# The Aryne Phosphate Reaction**

**DOI:** 10.1002/anie.202113231

**Published:** 2021-11-22

**Authors:** Thomas M. Haas, Stefan Wiesler, Tobias Dürr‐Mayer, Alexander Ripp, Paraskevi Fouka, Danye Qiu, Henning J. Jessen

**Affiliations:** ^1^ Institute of Organic Chemistry Albert-Ludwigs University Freiburg Albertstraße 21 79102 Freiburg im Breisgau Germany; ^2^ DFG Cluster of Excellence “Living, Adaptive and Energy-Autonomous Materials Systems” (livMatS) 79110 Freiburg Germany

**Keywords:** aryne chemistry, metaphosphates, oligophosphorylation, phosphorylation, polyphosphates

## Abstract

Condensed phosphates are a critically important class of molecules in biochemistry. Non‐natural analogues are important for various applications, such as single‐molecule real‐time DNA sequencing. Often, such analogues contain more than three phosphate units in their oligophosphate chain. Consequently, investigations into phosphate reactivity enabling new ways of phosphate functionalization and oligophosphorylation are essential. Here, we scrutinize the potential of phosphates to act as arynophiles, paving the way for follow‐up oligophosphorylation reactions. The aryne phosphate reaction is a powerful tool to—depending on the perspective—(oligo)phosphorylate arenes or arylate (oligo‐cyclo)phosphates. Based on Kobayashi‐type o‐silylaryltriflates, the aryne phosphate reaction enables rapid entry into a broad spectrum of arylated products, like monophosphates, diphosphates, phosphodiesters and polyphosphates. The synthetic potential of these new transformations is demonstrated by efficient syntheses of nucleotide analogues and an unprecedented one‐flask octaphosphorylation.

## Graphical abstract

The ability of phosphates to act as efficient arynophiles is demonstrated. The resulting aryne phosphate reaction is a versatile tool for arene phosphorylation or phosphate arylation. A broad scope of variously arylated phosphate products is culminating in novel nucleotide analogues and a benchmarking octaphosphorylation.

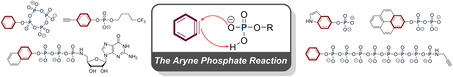

## Introduction

**Figure 1:**
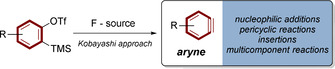
Aryne generation relying on Kobayashi's approach.

Arynes are “highly reactive, short‐lived intermediates that can undergo a variety of chemical transformations”^[1]^ The simplest representative is 1,2‐didehydrobenzene also known as benzyne (Figure 1). The central structural motif of arynes is a bent triple‐bond that is part of a strained aromatic (poly)‐cycle.[^2^, ^3^] This special bonding situation results in a low‐energy LUMO,^[4]^ leading to significant electrophilic reactivity.^[5]^ Consequently, arynes are captured efficiently by arynophiles in diverse reactions, like nucleophilic additions, pericyclic reactions, insertions or multicomponent processes.^[6]^ Due to this broad applicability, aryne‐based transformations are a powerful toolbox in chemical synthesis, underlined by their use in complex synthetic sequences towards natural products.[^7^, ^8^]

Because of their high reactivity and short lifetime,^[9]^ arynes are commonly generated in situ from stable precursors. Many strategies for aryne synthesis are available,^[10]^ and Kobayashi‐precursors based on the *o*‐silylaryltriflate functionality are particularly well developed.^[11]^ In this approach, fluoride ions induce aryne formation from bench‐stable precursors in a temperature range in between 0 °C to 100 °C.^[8]^

**Scheme 1:**
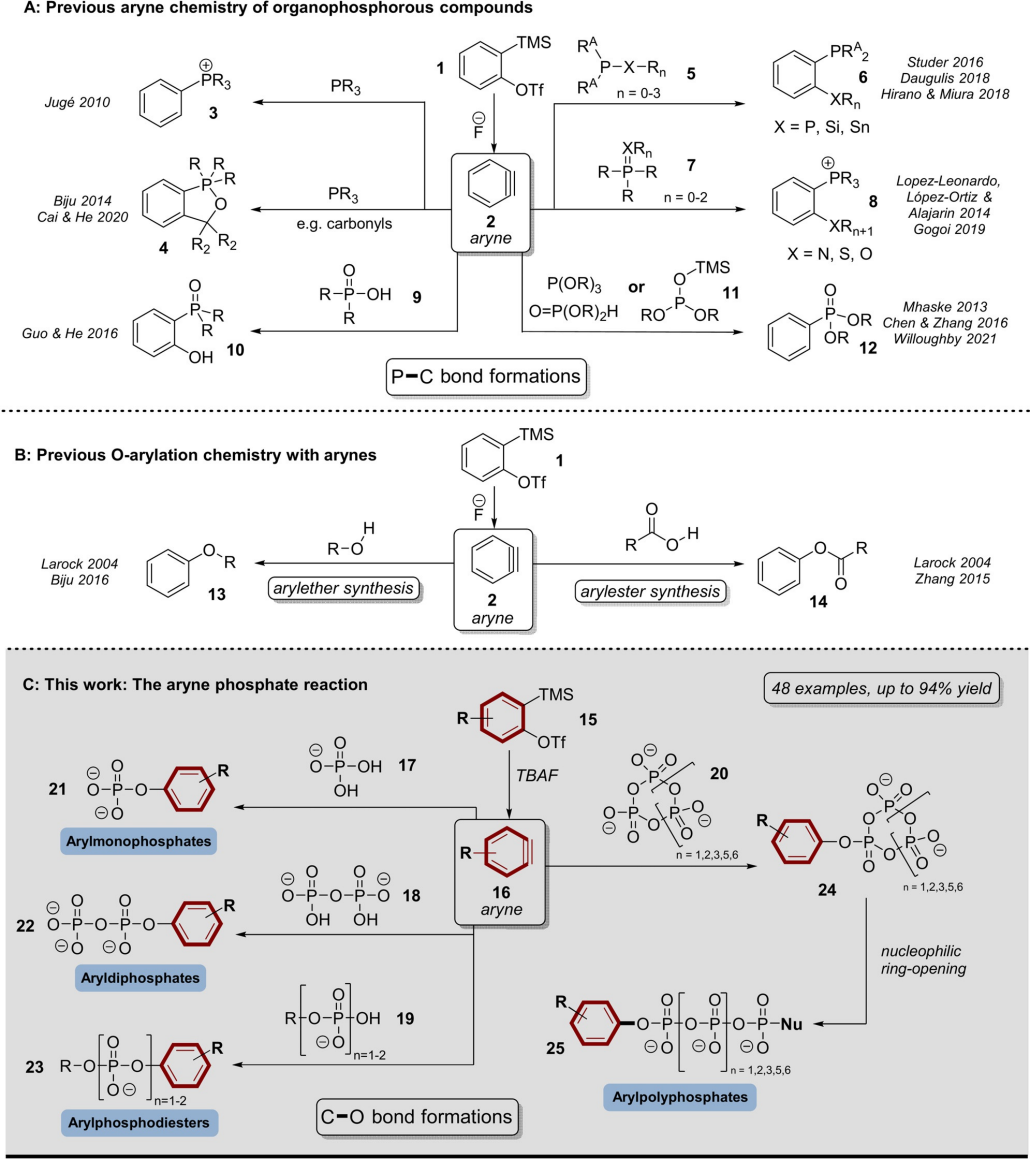
A) Previously reported aryne chemistry of organophosphorous compounds based on *o*‐silylphenyltriflates. B) Previously reported O‐arylation chemistry of arynes based on o‐silylaryltriflates. C) Synthetic concept of the aryne phosphate reaction including potentially accessible product groups.

Arylated organophosphorous compounds are important building blocks in organic synthesis including various applications in medicinal chemistry,^[12]^ polymer science^[13]^ or as ligands in transition metal catalysis.[^14^, ^15^] Aryne‐based methodologies have played a significant role in targeting such aryl‐phosphorous compounds (Scheme 1 A).[^16^, ^17^] In 2010, Jugé et al. presented a nucleophilic addition of phosphines to arynes leading to arylphosphonium salts (**3**).^[18]^ Biju, Cai and He demonstrated later in 2014 and 2016 how this reactivity can be applied in multicomponent reactions with carbonyls or CO_2_.[^19^, ^20^] Furthermore, Studer, Daugulis, Hirano and Miura developed aryne induced σ‐P^III^−X bond insertions accessing *ortho*‐stannylated, silylated or phosphinylated arylphosphines (**6**).[^21^, ^22^, ^23^] Analogously, Lopez‐Leonardo, López‐Ortiz, Alajarin and Gogoi presented π‐P^V^=X bond insertions leading to *ortho*‐aminated, thiolated or hydroxylated arylphosphonium products (**8**).[^24^, ^25^] Between 2013 and 2021 Mhaske, Chen, Zhang and Willoughby applied phosphites, H‐phosphonates and silylphosphates (**11**) to generate arylphosphonates (**12**).[^26^, ^27^, ^28^] Moreover, He and Guo presented a σ‐P‐O‐bond insertion of arynes into organophosphinic acids (**9**).^[29]^ In summary, the broad spectrum of aryne reactivity can be exploited with phosphorous functionalities towards diversely arylated products enabling C‐P‐bond formation. However, no exclusive aryne‐based O‐arylation has ever been described in the synthesis of organophosphorous compounds. More generally, “there are limited examples of O‐arylation transformations”^[6]^ using arynes (Scheme 1, B), overall restricted to few hydroxyl and carboxyl‐group arylation protocols since 2004.[^30^, ^31^, ^32^]

Despite the many studies scrutinizing the reactivity of diverse arynophiles, the ability of phosphates to engage in reactions with arynes is absent from the literature. This is remarkable, as the urgent need to access probes for interrogation of phosphate functions, for example, in analytical biochemistry, has driven innovative synthesis strategy development in the past years.[^33^, ^34^, ^35^, ^36^, ^37^]

Here, we present a detailed study of the phosphate aryne reaction. We demonstrate that phosphates, phosphate esters and anhydrides are arynophiles, which can be converted into O‐arylated phosphate derivatives. Starting from various substituted Kobayashi aryne precursors, we reacted the derived arynes with inorganic phosphate (P_i_, **17**) and pyrophosphate (PP_i_, **18**) towards arylated mono‐ (**21**) and diphosphates (**22**). In addition, phosphomonoesters (**19**) are selectively transformed into arylphosphodiesters (**23**). Notably, the method is applicable to cyclic condensed phosphates (**20**, also known as metaphosphates), leading to storable arylated cyclophosphate species (**24**) of controllable ring‐size, which have rarely been described.[^38^, ^39^] These species in turn are versatile oligophosphorylation reagents, linearizable by for example, amine nucleophiles. We evaluate this concept up to an unprecedented one‐flask octaphosphorylation reaction, but this appears not to be the limit. Overall, the scope of the aryne phosphate reaction is demonstrated in 48 examples covering a broad range of phosphates and aryne precursors.

## Results and Discussion

Initial experiments were performed with Kobayashi's 2‐(trimethylsilyl)phenyltrifluoromethanesulfonate (**1**) and PP_i_ (**18**) as a potential arynophile. An overview of the reaction optimization is shown in the SI (supporting Table 1). In short, MeCN was identified as the optimal solvent, by enabling complete dissolution of the phosphates as tetrabutylammonium‐salts (TBA) while maintaining aryne reactivity. TBAF (1 M in THF) was the ideal fluoride source as inorganic fluoride salts like CsF induced the precipitation of insoluble pyrophosphate metal salts. The rate and order of reagent addition turned out to play a critical role during reaction optimization. The highest yields were achieved, when TBAF was added slowly within 1 h via a syringe‐pump to a solution of phosphate and aryne‐precursor in MeCN. Notably, the reaction can be performed under ambient conditions without exclusion of moisture.

**Scheme 2:**
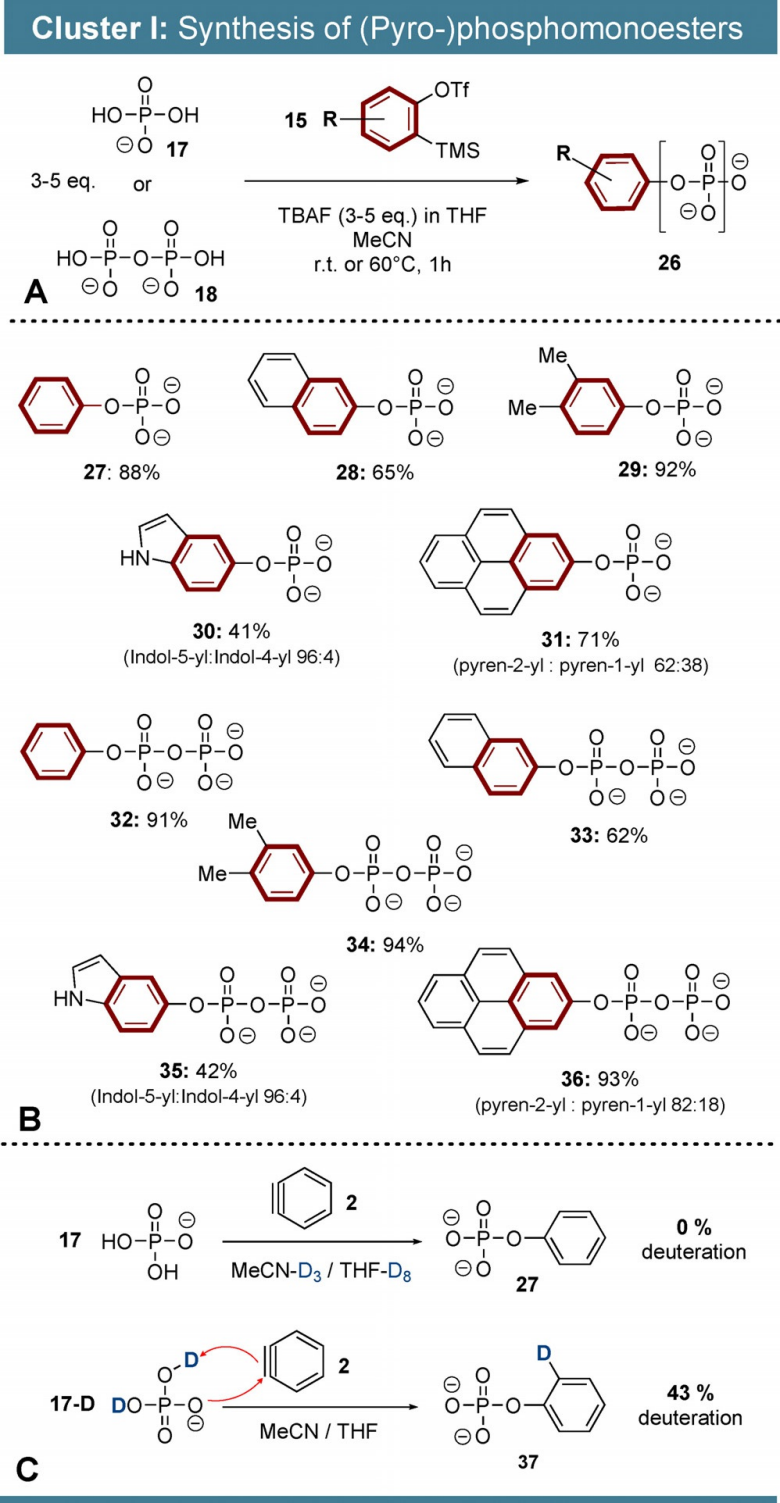
Reaction cluster I. Synthesis of (pyro‐)phosphomonoesters. A) Synthetic concept. The reactions were performed on 300 μmol scales. Monophosphate syntheses were performed at 60 °C, diphosphate syntheses at rt. P_i_ and PP_i_ were used as TBA salts. The products were isolated as TEA and TBA salts or mixtures thereof. B) Substrate scope. C) Deuteration experiments.

These optimized reaction conditions were applied in a first cluster of transformations: arynes derived from *o*‐silylaryltriflates were reacted with P_i_ (**17**) and PP_i_ (**18**) leading to (pyro‐)phosphomonoesters (**26**, Scheme 2 A). An excess of P_i_ and PP_i_ was applied in these transformations, as overreactions towards phosphodiesters were observed otherwise (see supporting Figures 3 & 4). In the case of P_i_, 60 °C led to the highest yields, but PP_i_ reactions required room temperature to avoid anhydride hydrolysis and over‐arylation. The products (Scheme 2 B) were purified chromatographically and usually isolated as TBA or TEA salts. Following this procedure, phenylphosphate **27** was synthesized in 88 % yield. 2‐Naphtylphosphate **28** was accessed from the corresponding naphtyne precursor in 65 % yield and 3,4‐dimethylphenylphosphate (**29**) was isolated in a yield of 92 %. Garg's indole 4,5‐indolyne‐precursor was transformed to indolylphosphate **30** in 41 % yield, in a regioisomeric ratio of 96:4 (5‐**30**:4‐**30**). The observed regioisomeric preference towards C5‐**30** is in accordance with literature precedence and can be explained by Garg's *aryne distortion model*.^[40]^

Furthermore, we developed a new pyrene‐derived aryne‐precursor (**SI‐19**) that was transformed successfully into the corresponding pyrenyl‐phosphate **31** in 71 % yield. The pyren‐2‐yl regioisomer was preferentially formed in a ratio of 62:38. Presumably steric effects as well as aryne distortion synergistically direct the phosphate to pyrene's C2 position,^[41]^ further supported by an increase in selectivity for the bulkier PP_i_. We envision the reaction mechanism as a proton‐coupled nucleophilic addition of phosphates (Scheme 2 C) to arynes. Inter‐ and intramolecular proton transfer both seem plausible, as both residual phosphate protons from the TBA salt and water content in TBAF solutions, are suitable H^+^‐sources. Consistently, the application of MeCN‐D_3_ and THF‐D8 as solvents did not lead to deuterium incorporation into the product (Scheme 2 C). In contrast, arylation of isotopically labelled phosphate 17‐D led to 43 % product deuteration.

While arguably arylmonophosphates are also accessible by alternative methods,[^42^, ^43^] the aryne phosphate reaction enables the straight‐forward synthesis of otherwise difficult to access monoarylpyrophosphates. In fact, PP_i_ proved to be a very efficient arynophile, leading to consistently high yields (Scheme 2 B). To ensure turnover at lowered reaction temperature, the PP_i_ equivalents had to be raised compared to the P_i_ case. Phenyldiphosphate **32**, 3,4‐dimethylphenyl‐diphosphate **34** and pyrenyldiphosphate **36** were isolated in >90 % yields. 2‐Naphtyldiphosphate **33** was obtained in 62 % yield and indolyldiphosphate **35** was accessed in 42 % yield.

**Scheme 3:**
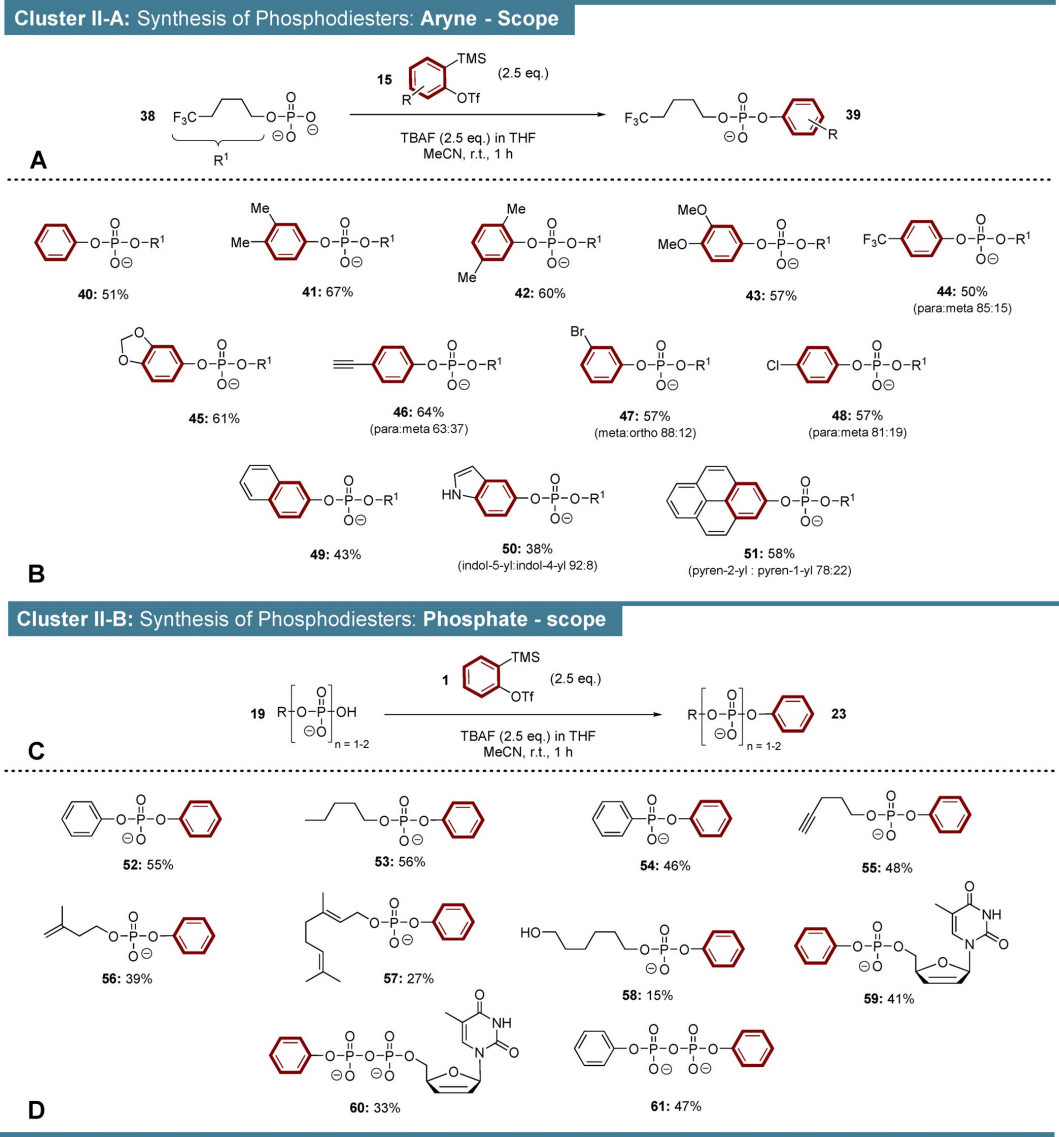
Reaction cluster II. Synthesis of arylphosphodiesters. Phosphates were introduced as TBA salts. The products are isolated as TEA or TBA salts. A) Synthetic concept of aryne scope investigation. The reactions were performed on 300 μmol scales and at concentrations of 200 mM. B) Aryne substrate scopes. C) Synthetic concept of phosphate monoester scope investigation. The reactions were performed on 150–500 μmol scales and at concentrations of 200 mM. D) Phosphate monoester substrate scope.

In a further cluster of experiments, we investigated the aryne phosphate reaction using phosphomonoesters as starting materials (Scheme 3 A). These transformations lead to phosphodiester products. 5,5,5‐Trifluoropentylphosphate (**38**) was chosen as standard substrate in the aryne scope delineation (Scheme 3 A+B), as the CF_3_‐moiety can be used as an analytical handle for NMR‐detection of products. **38** was reacted with 2.5 equivalents of *o*‐silylaryltriflates and TBAF following the procedure described above. Only little overreaction to phosphate triesters was generally observed (<10 %) and these triesters usually decomposed during chromatographic purification. Under the applied conditions, **38** was successfully transformed into arylalkyldiester **40** in 51 % yield. Dimethylphenyldiesters **41** and **42** were isolated in 67 % and 60 % yield, respectively. The reaction tolerates donor‐ and acceptor‐substituted arynes delivering for example, dimethoxyphenyl‐ and trifluoromethylphenyl diesters **43** and **44** yields of 57 % and 50 %. In the case of unsymmetric **44**, the *para*‐product was preferred in a ratio of 85:15. Sesamol‐derived diester **45** was isolated in 61 % yield. An alkynylated aryne precursor (**SI‐23**) was developed and successfully applied generating **46** in 64 % yield and a *para*:*meta* ratio of 84:16. Such clickable phosphordiester can be further functionalized by copper catalysed Huisgen‐type cycloadditions.^[44]^ Br‐ and Cl‐substituted diesters (**47**, **48**) were synthesized in comparable yields of 57 %, while the Br‐substituent induced a higher regioselectivity. Furthermore, naphtyldiester **49** was isolated in 43 % yield and indolyl‐diester **50** was generated in a yield of 38 % with a C5 positional preference of 85:15. Finally the pyryne was transformed to diester **51** in 58 % yield and with a C2‐preference of 78:22.

Subsequently, the phosphate scope was investigated using several organophosphates under the conditions described above (Scheme 3 C+D). Diphenylphosphate **52** was synthesized in 55 % yield. Similarly, pentylphenylphosphate **53** was isolated in 56 % yield and alkynylated derivative **55** was obtained in 48 %. Notably, the reaction extends to phosphonates as demonstrated by the O‐arylation of phenylphosphonate in 46 % yield (**54**). In addition, terpenoide phosphates were successfully arylated leading to isoprenol derivative **56** in 39 % yield and the challenging geraniol‐based allylphosphate **57** in 27 % yield.

The chemoselectivity regarding hydroxy‐ and phosphate‐groups—both O‐nucleophiles—was studied using bifunctional 6‐hydroxy‐hexylphosphate as a model substrate. Under the described reaction conditions, the phosphate group could however not be arylated selectively to give **58**. Hydroxyarylation occurred to a comparable extent, leading to a modest yield of 15 %. This also explains that a highly selective phosphate arylation of unprotected nucleotides like adenosine monophosphate could not be achieved. In contrast, nucleotide analogue d4T‐monophosphate was arylated successfully, generating **59** in 41 % yield. Furthermore, also organodiphosphates were arylated, as demonstrated by d4T‐diphosphate arylation to derivative **60** in 33 % yield. Finally, diphenylpyrophosphate (**61**) was isolated in 47 % yield.

**Scheme 4:**
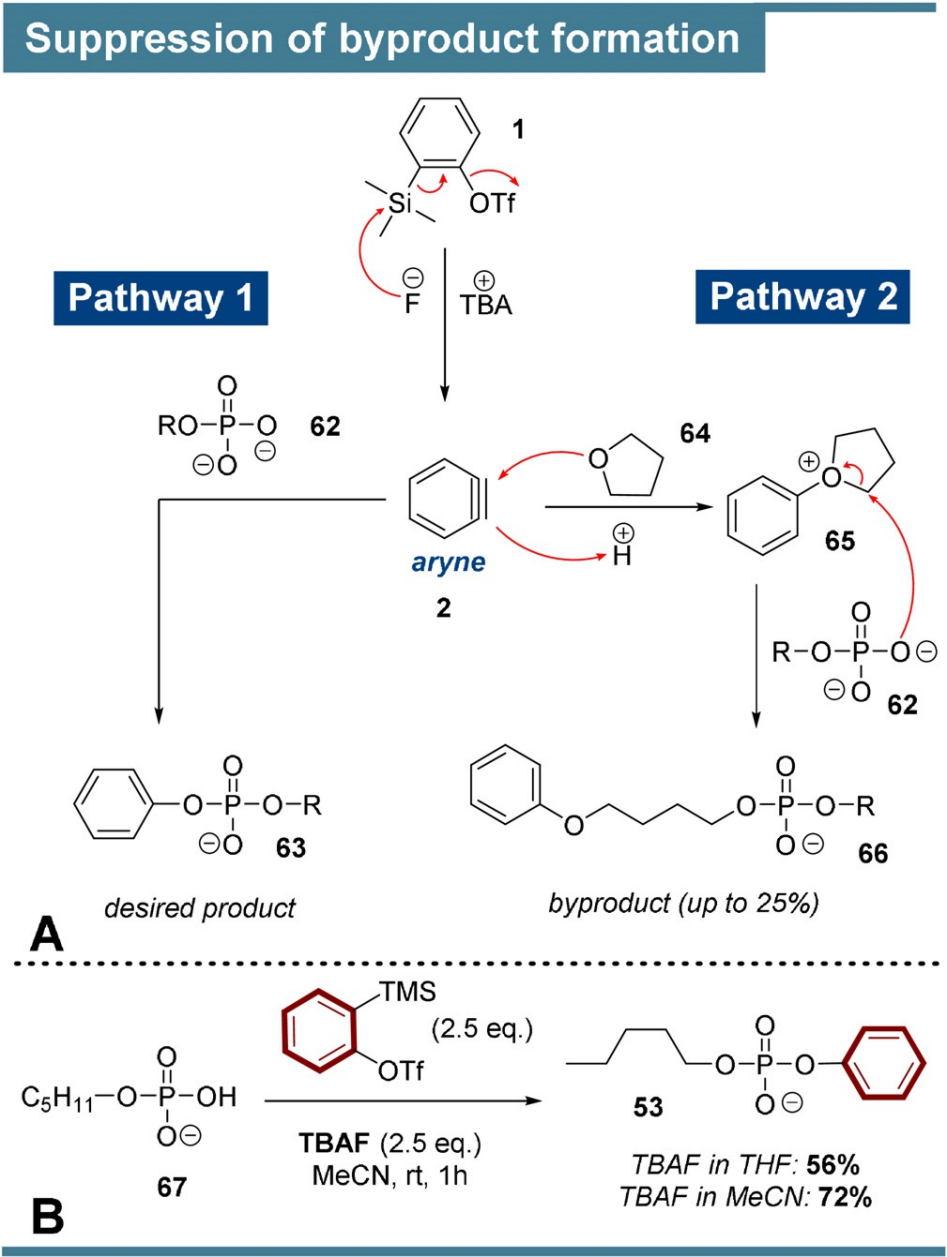
A) Depiction of product formation (pathway 1) and byproduct formation (pathway 2). B) Successful byproduct suppression by TBAF‐solvent exchange increases the yield significantly.

While overall good results were obtained in our cluster II studies, an obvious reduction in yields was observed as compared to cluster I. We now understand this limitation, as many reactions shown in Scheme 3 also generated distinct byproducts, which can be explained mechanistically as outlined in Scheme 4: In addition to pathway 1 leading to the desired products, byproduct formation according to pathway 2 was observed on the order of 25 %. In this case, the TBAF‐solvent tetrahydrofuran (THF, **64**) intercepts the aryne (**2**) under formation of cyclic oxonium‐ion **65**. Subsequently, the corresponding phosphate **62** attacks **65** in an S_N_2‐process leading to byproduct **66**. The non‐innocence of THF towards arynes was already reported in 1970^[45]^ and is sometimes exploited synthetically in multicomponent approaches.^[46]^ Suppression of pathway 2, by changing TBAF‐solvent from THF to MeCN (Scheme 4 B), should therefore increase the yields significantly. We demonstrate this with pentylphosphate (**67**), where solvent exchange led to the substantially improved yield of 72 % as compared to 56 % when the medium contained THF. Therefore, it should be possible to increase the yields of the reactions summarized in Scheme 3 at the expense of an additional solvent exchange procedure. Of note, the THF‐derived byproducts were not observed for cluster I reactions (Scheme 2), as in these cases P_i_ and PP_i_ were applied in excess accompanied by reduced TBAF‐equivalents.

**Scheme 5:**
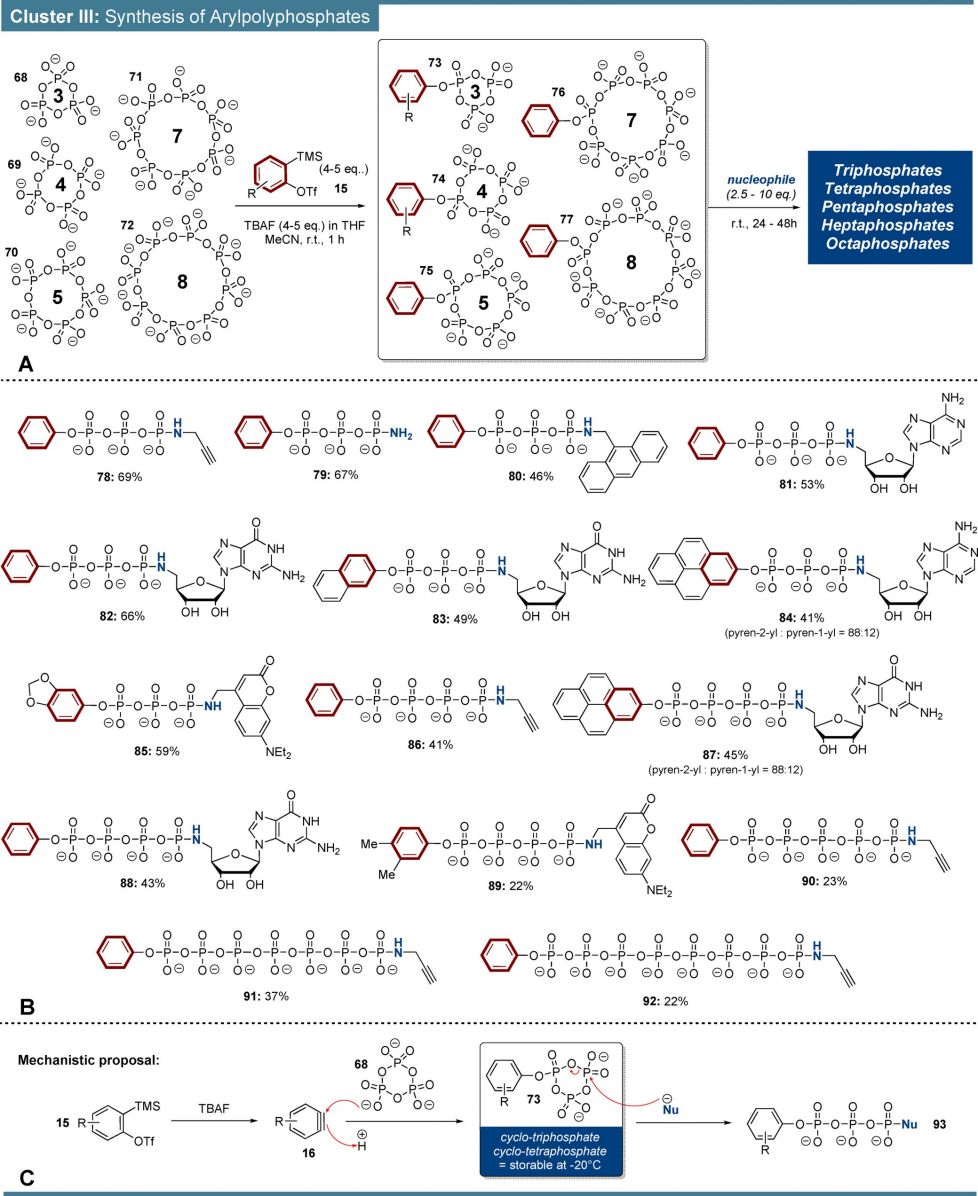
A) Synthetic concept of reaction cluster III. Arylation of cyclophosphates followed by nucleophilic ring‐opening. The reactions were performed on 100 μmol scales and at concentrations of 70 mM. For P_5_‐P_8_ syntheses, the amount of aryne precursor (4.0 equiv.) and nucleophile (20 equiv.) differed from P_3_‐P_4_ syntheses [aryne precursor (5.0 equiv.), nucleophile (2.5 equiv.)]. B) Substrate scope of arylpolyphosphate synthesis. C) Mechanistic proposal of nucleophilic ring‐openings.

In a third cluster of aryne phosphate reactions, the reactivity of arynes towards cyclophosphates was explored (Scheme 5 A). Initial experiments with trimetaphosphate (**68**) using optimized conditions from Scheme 2 proved the formation of arylcyclotriphosphate **73**, but 4–5 equiv. of aryne precursor were necessary to ensure complete turnover. Despite the aryne excess, bisarylations were marginally observed. Further reaction conditions were kept constant. Notably, activation of cyclophosphates by arylation is not restricted to trimetaphosphate (**68**), but can also be applied to tetra‐ (69), penta‐ (70), hepta‐, (71) and octametaphosphate (72). Monoarylcyclophosphates **73** and **74** could be isolated as oils after precipitation and stored as reagents for several weeks at −20 °C without substantial decomposition. Hence, **73** and **74** are storable tri‐ and tetraphosphorylation reagents.

All arylcyclophosphates **73**–**77** were subsequently linearized using N‐nucleophiles, based on an S_N_ type mechanism (Scheme 5 C).^[35]^ In accordance with literature, no branched products were detected.^[36]^ The reaction sequence enabled the isolation of various arylated polyphosphate chains (P_3_‐P_8_, Scheme 5 B). Most polyphosphate products were purified by automated strong‐ion exchange chromatography (SAX) and isolated as Na salts.

Using Kobayashi's *o*‐silylphenyltriflate (**1**), trimetaphosphate (**68**) was smoothly transformed into phenyltrimetaphosphate (**73**), which was then ring‐opened by several amine nucleophiles generating the terminally modified triphosphates **78**–**85**. For example, propargylamidotriphosphate **78** and amidotriphosphate **79** were isolated in overall yields of 69 % and 67 % from trimetaphosphate. Ring‐opening with anthracenylmethanamine led to triphosphate **80** in 46 % yield. Furthermore, phenylcyclotriphosphate could be linearized with 5′‐aminonucleosides, such as aminoadenosine **SI‐1** and aminoguanosine **SI‐2** leading to the nucleotide analogues **81** and **82** in 53 % and 66 % yield, respectively. Alternative aryne precursors were coupled analogously with trimetaphosphate, exemplified in naphtylated GTP analogue **83**, accessed in 49 % yield. The ATP‐derived pyrenyltriphosphate **84** was isolated in 41 % yield. The higher steric demand of cyclotriphosphate compared to P_i_ (Scheme 2) is reflected in an increased regioisomeric C2‐preference of 88:12. In addition, sesamolcyclotriphosphate was opened with amino‐DEACM (**SI‐26**) towards the fluorescent triphosphate analog **85** in 59 % yield.

Likewise, tetrametaphosphate was efficiently arylated using *o*‐silylaryltriflate precursors. Propargylamidotetraphosphate **86** was synthesized in 41 % yield. Ring‐opening of phenyltetraphosphate with aminoguanosine generated G4P‐analogue **88** in 43 % yield. Furthermore, the synthesis of pyrenylated G4P (**87**) was achieved in 45 % yield. The regioselectivity was similar to cyclotriphosphate example **84**. Fluorescent dimethylphenyltetraphosphate **89** was accessed via amino‐DEACM in 22 % yield.

Propargylamidopentaphosphate **90** was isolated in 23 % yield from pentametaphosphate (**70**), by ring‐opening of phenylcyclopenta‐phosphate **75**. Analogously, heptaphosphate **91** and octaphosphate **92** were isolated in yields of 37 % and 22 %, respectively, from their corresponding metaphosphate precursors. In the cases of polyphosphates P_>4_ the nucleophile equivalents were increased from 2.5 to 10, to ensure smooth ring‐opening.

## Conclusion

In 2017 Garg stated about arynes that “their high reactivity has seemingly steered chemists away from using them to assemble […] complex scaffolds. [But] arynes can and should be used strategically to enable the synthesis of complex molecules with motifs that have conventionally been viewed as challenging.”^[47]^ In the present paper, we meet this suggestion by introducing the aryne phosphate reaction that accesses highly challenging arylated organophosphorous compounds with Kobayashi‐type precursors. We demonstrate that inorganic phosphates, organophosphates and cyclophosphates are efficient arynophiles in O‐arylation reactions. An extensive portfolio of products was presented, ranging from arylmonophosphates and ‐diphosphates to arylphosphodiesters and ‐polyphosphates. Yet, there are countless more combinations that can be envisioned to further expand the repertoire of the aryne phosphate reaction. The versatility of this novel transformation is further demonstrated by synthetically unlocking penta‐, hepta‐ and octaphosphorylations in one‐flask operations. Polyphosphorylation reagents introducing more than four phosphate units in a single reaction have never been reported before and consequently, the disclosed penta‐, hepta‐ and octaphosphorylation procedures set new benchmarks in polyphosphorylation chemistry. It is apparent that longer polyP chains could potentially be accessible by either extending the ring‐size or by using phosphate nucleophiles on arylated cyclophosphates. We are confident that the aryne phosphate reaction will stimulate tool design in nucleotide and polyP research and—more generally—enable us to venture into the largely uncharted and fascinating realm of condensed phosphates.^[48]^

## Conflict of interest

The authors declare no conflict of interest.

## Supporting information

As a service to our authors and readers, this journal provides supporting information supplied by the authors. Such materials are peer reviewed and may be re‐organized for online delivery, but are not copy‐edited or typeset. Technical support issues arising from supporting information (other than missing files) should be addressed to the authors.

Supporting Information
