# Supplementary material for: The Aryne Phosphate Reaction
Source: Angew Chem Int Ed Engl. 2021 Nov 22;61(1):e202113231. doi: 10.1002/anie.202113231 (PMC9299019; doi:10.1002/anie.202113231)
Supplement: Supplementary file 1 — Supporting Information [file ANIE-61-0-s001.pdf]

Angewandte Chemie
Eine Zeitschrift der Gesellschaft Deutscher Chemiker

Supporting Information

The Aryne Phosphate Reaction**

*Thomas M. Haas[+], Stefan Wiesler[+], Tobias Dürr-Mayer, Alexander Ripp, Paraskevi Fouka, Danye Qiu, and Henning J. Jessen**

anie_202113231_sm_miscellaneous_information.pdf

Table of content

1. General remarks
2. Screening overview
3. Syntheses adapted from literature
4. **Cluster 1:** Synthesis of (Pyro-)phosphomonoesters
5. **Cluster 2A:** Synthesis of (Pyro-)phosphodiesters (aryne-Scope)
6. **Cluster 2B:** Synthesis of (Pyro-)phosphodiesters (phosphate-Scope)
7. **Cluster 3:** Synthesis of Arylpolyphosphates (cyclophosphate reactions)
8. Synthesis of phosphate starting materials without literature precedence
9. Synthesis of aryne precursors without literature precedence
10. Synthesis of Amino-DEACM
11. Supporting references
12. NMR-spectra
13. MS – spectra
14. Structure Tables (X-ray data)

Abbreviations

d4T	1-[(2R,5S)-5-(Hydroxymethyl)-2,5-dihydrofurane-2-yl]-5-methylpyrimidine-2,4-dion
DBU	1,8-Diazabicyclo[5.4.0]undec-7-ene
DEACM	7-(diethylamino)-4-(hydroxymethyl)-coumarine
DCM	Dichloromethane
DMF	Dimethylformamide
DMSO	Dimethyl sulfoxide
Et_2O	Diethyl ether
ETT	5-(Ethylthio)-1*H*-tetrazole
Fm	Fluorenylmethyl
*m*CPBA	*meta*-Chloroperoxybenzoic acid
MeCN	Acetonitrile
HPLC	Reverse phase high-performance liquid chromatography
HRMS	High resolution mass spectrometry
Pi	Inorganic phosphate
PPi	Inorganic pyrophosphate
qNMR	Quantitative NMR
RP-MPLC	Reverse phase medium pressure liquid chromatography
SAX	Strong anion exchange
TBA	Tetrabutylammonium
TBAF	Tetrabutylammoniumfluoride
TEA	Triethylammonium
TEAA	Triethylammonium acetate
TMS	Trimethylsilyl

1. General remarks

Reactions were carried out using glassware magnetically stirred, unless noted otherwise. Air- and moisture-sensitive liquids and solutions were transferred via syringe or stainless steel canula.

Reagents were purchased from commercial suppliers (Acros, Aldrich, Fluka, TCI) and used without further purification, unless noted otherwise.

Solvents were obtained in analytical grade and used as received for extractions, precipitation and solid washing.

Dry solvents for reactions were purchased in a dry form from Sigma and stored over molecular sieves as well as under the atmosphere of dry N_2.

Deuterated solvents for NMR and reactions were obtained from Armar Chemicals, Switzerland and euriso-top, Germany, in the indicated purity grade and used as received for NMR spectroscopy.

Strong ion-exchange chromatography was performed using an automated Äkta® – system. Q-Sepharose was purchased from Aldrich. Buffer solutions were produced manually using milliQ H_2O.

TBA-salt preparations were performed by either using DowexH^+ followed by TBA(OH) addition or Chelex®100 (preloaded with TBA). In both cases, the TBA salts were obtained after lyophilization.

Commercially available phosphates (e.g. phenylphosphate, phenylphosphonate) were transformed into their corresponding TBA-salts as described above.

Commercially available aryne precursors (2-(trimethylsilyl)phenyl triflate, 2-Bromo-6-(trimethylsilyl)phenyl triflate, Garg 4,5,-indolyne precursor) were purchased from Sigma and used without further purification.

Preparative RP-MPLC was performed using an automated Interchim® - system. The C18AQ-solid phase was purchased from Interchim.

Lyophilizations were done with Christ Freeze Dryer Alpha 1-4 LDplus and Christ Freeze Dryer Alpha 1-2 LDplus.

^{1}H-NMR spectra were recorded on Bruker 300 MHz spectrometers, Bruker 400 MHz (with cryoprobe) and Bruker 500 MHz spectrometers in the indicated deuterated solvent. Data are reported as follows: chemical shift (δ, ppm), multiplicity (s, singlet; d, doublet; t, triplet; q, quartet; m, multiplet; br. s, broad signal), coupling constant(s) (*J*, Hz), integration. All signals were referenced to the internal solvent signal as standard (D_2O, δ 4.79; MeCN-d_3, δ 1.94, DMSO-d_6, δ 2.50, $CDCl_3$, δ 7.26).
In some phosphate products there is still acetate (mostly as TBA-salt) present after RP-MPLC followed by lyophilization. These buffer residues were considered for yield determination. After $NaClO_4$ – purification acetone residues were present in the products. These were also considered for yield determination.

^{13}C{^{1}H}-NMR spectra were recorded with ^{1}H-decoupling on Bruker 126 MHz, Bruker 101 MHz (with cryoprobe) spectrometers at 298K in the indicated deuterated solvent. If possible, signals were referred to the internal solvent signal as standard (MeCN-d_3, δ 1.32, DMSO-d_6, δ 39.52, $CDCl_3$, δ 77.16).

^{31}P{^{1}H}-NMR spectra and ^{31}P-NMR spectra were recorded with ^{1}H-decoupling or ^{1}H coupling, respectively, on Bruker 202 MHz , 162 MHz (with cryoprobe) and Bruker 122 MHz spectrometers in the indicated deuterated solvent. All signals were referenced to an internal standard (PPP).

Mass spectra were recorded by C. Warth (Mass spectrometry service of the University of Freiburg) on a Thermo LCQ Advantage [spray voltage: 2.5 – 4.0 kV, spray current: 5 µA, ion transfer tube: 250 (150) °C, evaporation temperature: 50 – 400°C.

2. Screening overview

The TBA-salt of pyrophosphate and 2-(trimethylsilyl)phenyl trifluoromethanesulfonate were used as a starting point for optimization of the aryne phosphate coupling (see supporting figure 1). The ratios between starting material and the arylated products was used as “reactivity” parameter: the higher the arylation rate, the better the reaction conditions. The turnover was determined by ^{31}P-NMR analysis (see supporting figure 2). In summary, TBAF was the superior fluoride source. MeCN proved the most efficient solvent. A slow addition of the fluoride source was crucial for high turnover. A PP_i concentration of 80 mM was suitable and TBAF excess was not necessary. The optimized conditions are presented in table entry 12.

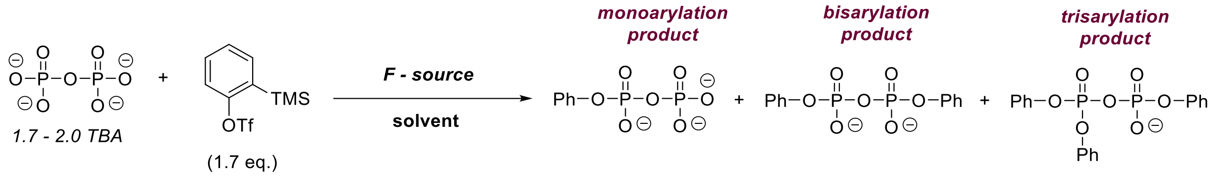

Supporting figure 1: model reaction used for optimizing F-source, solvent, temperature, addition time and concentration.

Supporting table 1: selected reaction conditions and corresponding turnover results: SM = starting material, PP_i, 1 = monoarylation product, 2 = bisarylation product, 3 = trisarylation product.

varied Parameters	Nr.	**solvent (conc.)**	**F-Source (eq.)**	**Temp.[°C]**	**Addition time (F-source)**	**SM:1:2:3 [%]**
F-sources	1	MeCN (80 mM)	CsF (3 eq.)	25	-	54:42:4:0
	2	MeCN (80 mM)	KF (3 eq.) 12-crown-4	25	-	50:50:0:0
	3	MeCN (80 mM)	TBAF (3 eq.)	25	1 min	36:54:10:0
solvents	4	Acetone (80 mM)	TBAF (3 eq.)	25	1 min	41:53:6:0
	5	DCM (80 mM)	TBAF (3 eq.)	25	1 min	76:23:1:0
	6	THF (80 mM)	TBAF (3 eq.)	25	1 min	61:29:10:0
	7	DME (80 mM)	TBAF (3 eq.)	25	1 min	93:4:3:0
addition speed	8	MeCN (80 mM)	TBAF (3 eq.)	25	60 min	21:43:29:7
temperature	9	MeCN (80 mM)	TBAF (3 eq.)	0	60 min	39:47:17:0
concentration	10	MeCN (110 mM)	TBAF (3 eq.)	25	60 min	44:48:8:0
	11	MeCN (40 mM)	TBAF (3.0 eq.)	25	60 min	23:42:28:7
F-source (equ.)	**12**	**MeCN (80 mM)**	**TBAF (1.7 eq.)**	**25**	**60 min**	**21:43:30:6**

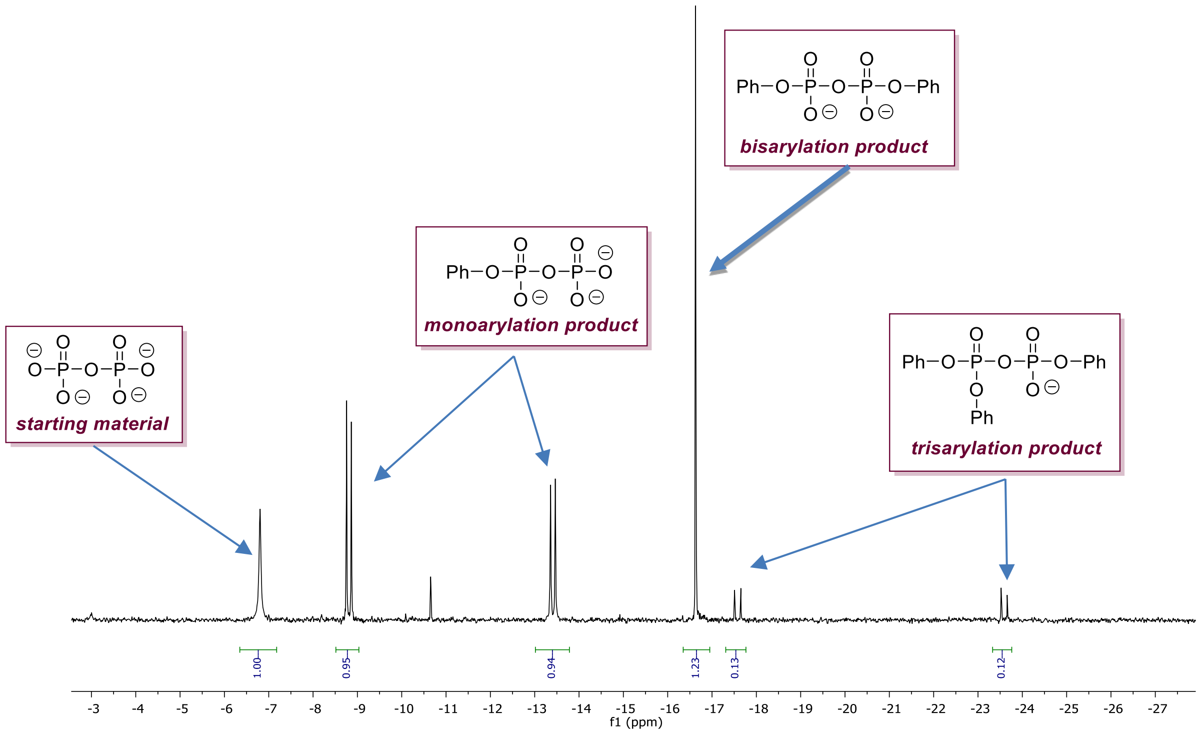

Supporting figure 2: exemplified ^{31}P-NMR determination of starting material to product ratios during reaction optimization.

3. Syntheses adapted from literature

Synthesis of 5'-Deoxy-5'-aminoadenosine (SI-1)

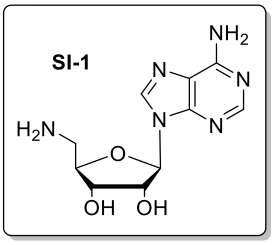

Compound **SI-1** was synthesized according to Ugarkar et al. The analytical data are in accordance with literature.[1]

Synthesis of 5'-Deoxy-5'-aminoguanosine (SI-2)

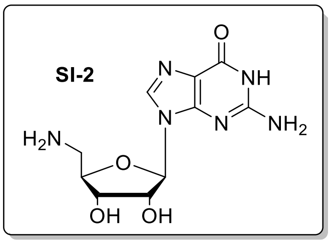

Compound **SI-2** was synthesized according to Dean. The analytical data are in accordance with literature.[2]

Synthesis of Pent-4-yn-1-ylphosphate (SI-3)

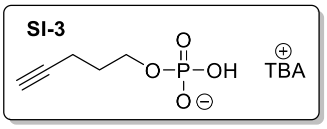

Compound **SI-3** was synthesized according to Singh et al. The analytical data are in accordance with literature.[3] Cations were changed to TBA as described above.

Synthesis of $(FmO)_2P$-N(*i*Pr)$_2$ (SI-4)

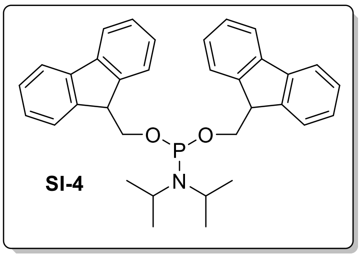

Compound **SI-4** was synthesized according to BIALY et al. The analytical data are in accordance with literature.[4]

d4T-monophosphate (SI-5)

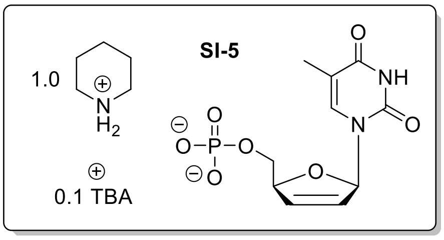

d4T (500 mg, 2.32 mmol) and ETT (725 mg, 5.58 mmol, 2.5 eq.) were dissolved in DMF. $(FmO)_2P$-N*i*Pr$_2$ (1.51 g, 2.90 mmol, 1.3 eq.) was added as solution in DMF (10 mL) and the resulting mixture was stirred for 1 h at rt. The solution was cooled to 0 °C and *m*CPBA (77%, 1.07 g, 4.35 mmol, 1.5 eq.) was added. The solution was stirred for 15 min at 0 °C and piperidine (10 vol%) was added. The solution was stirred for 40 min at rt. The solvent was removed under reduced pressure. The crude product was purified by RP-MPLC [C18-AQ, dryload on celite, elution with H_2O/MeCN/ TEAA (10 mM)]. The product (**SI-5**, 407 mg, 804 µmol, 36%) was isolated as white solid. NMR and HRMS data were in accordance with literature.[5]

^{1}H-NMR (400 MHz, D_2O, δ/ppm): 7.63 (q, *J* = 1.2 Hz, 1H), 6.96 (ddd, *J* = 3.3, 1.9, 1.5 Hz, 1H), 6.49 (dt, *J* = 6.1, 1.7 Hz, 1H), 5.95 (ddd, *J* = 6.2, 2.4, 1.4 Hz, 1H), 5.13 – 5.07 (m, 1H), 4.05 (dd, *J* = 5.7, 3.2 Hz, 2H), 1.89 (d, *J* = 1.2 Hz, 3H).* **^{31}P{^{1}H}-NMR** (162 MHz, D_2O, δ/ppm): 0.41. **HRMS** (ESI) m/z for $C_{10}H_{12}O_7N_2P$ [M-H]$^-$: calcd. 303.0388, found 303.0388.
*piperidinium and TBA-signals are not reported.

Synthesis of 4,5-dimethyl-2-(trimethylsilyl)phenyl trifluoromethanesulfonate (SI-6)

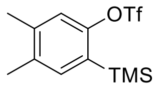

SI-6

Compound **SI-6** was synthesized according to UETA et al. The analytical data matched the previously published values.[6]

Synthesis of 3,6-dimethyl-2-(trimethylsilyl)phenyl trifluoromethanesulfonate (SI-7)

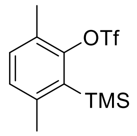

SI-7

Compound **SI-7** was synthesized according to WANG et al. The analytical data matched the previously published values.[7]

Synthesis of 4,5-dimethoxy-2-(trimethylsilyl)phenyl trifluoromethanesulfonate (SI-8)

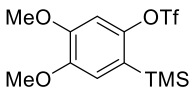

SI-8

Compound **SI-8** was synthesized according to XU et al. The analytical data matched the previously published values.[8]

Synthesis of 6-(trimethylsilyl)benzo[*d*][1,3]dioxol-5-yl trifluoromethanesulfonate (SI-9)

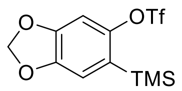

SI-9

Compound **SI-9** was synthesized according to UETA et al. The analytical data matched the previously published values.[6]

Synthesis of 5-chloro-2-(trimethylsilyl)phenyl trifluoromethanesulfonate (SI-10)

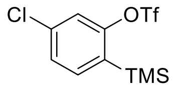

SI-10

Compound **SI-10** was synthesized according to PEÑA et al. The analytical data matched the previously published values.[9]

Synthesis of 4-(trifluoromethyl)-2-(trimethylsilyl)phenyl trifluoromethanesulfonate (SI-11)

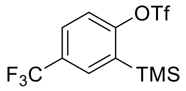

11

Compound **SI-11** was synthesized according to GHOTEKAR et al. The analytical data matched the previously published values.[10]

Synthesis of 3-(trimethylsilyl)naphthalen-2-yl trifluoromethanesulfonate (SI-12)

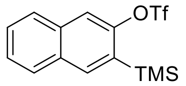

SI-12

Compound **SI-12** was synthesized according to UETA et al. The analytical data matched the previously published values.[6]

4. Cluster 1: Synthesis of (Pyro-)phosphomonoesters

Preliminary experiments

When cluster I reactions were performed with comparable molarity in P_i (**17**) and aryne - precursor **15** (supporting figure 3), substantial overreaction towards diphenylphosphate **52** was observed. This is underlined by the $^{31}P\{^1H\}$-NMR spectrum of the corresponding crude product mixture shown in supporting figure 4. The reason is a similar reactivity of P_i (**17**) and phosphomonoester **27** towards arynes. To suppress this overreaction and enable a coherent product formation, cluster I reactions were performed with an excess of P_i or PP_i.

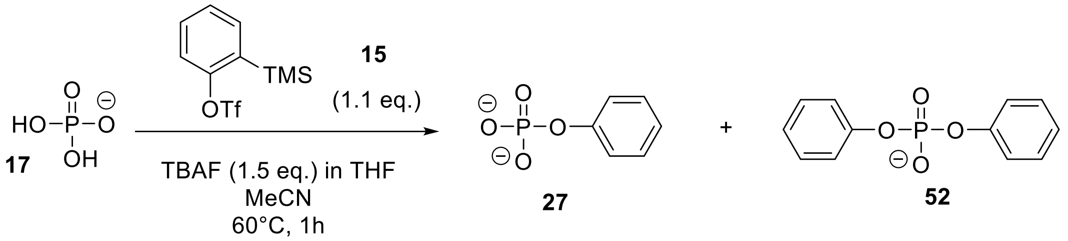

Supporting figure 3: reaction between P_i (**17**) and a slight excess of aryne precursor **15**.

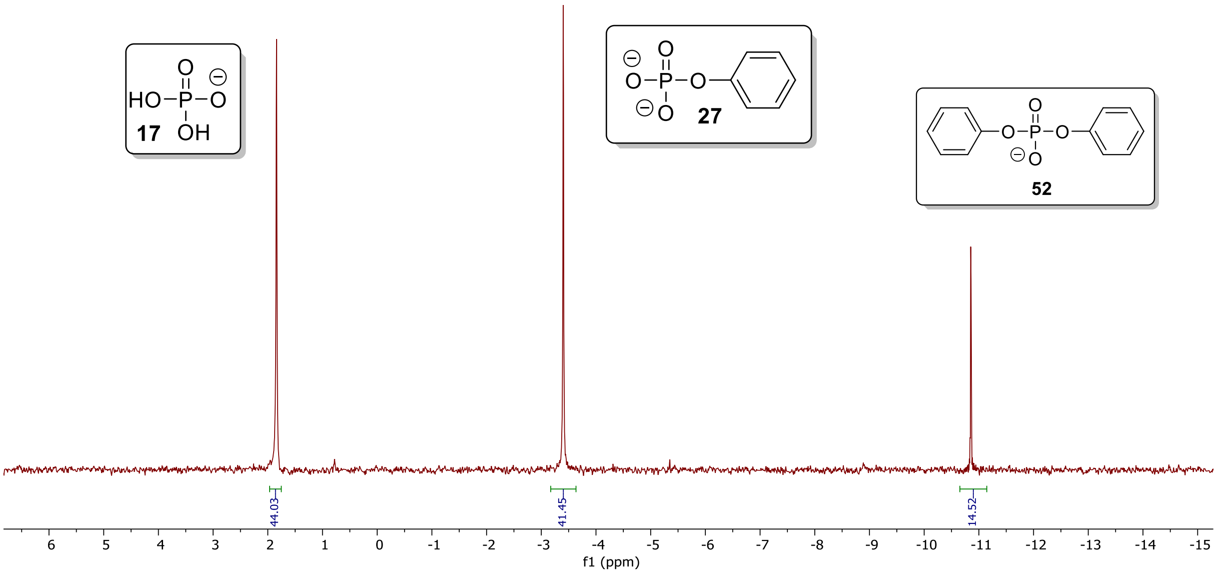

Supporting figure 4: ^{31}P-NMR spectrum of crude product mixture from reaction conditions according to supporting figure 3. Substantial overreaction towards diester **52** is observed.

General procedure A for the synthesis of phosphomonoesters:

The phosphate x TBA salt (900 µmol, 3.0 eq) was dissolved in dry MeCN (67 mM) before the aryne-precursor (300 µmol) was added. Subsequently the mixture was heated to 60 °C and TBAF (1 M in THF, 1.1 eq.) was added dropwise via syringe pump (1 h, 60°C, needle tip is below solvent surface). After removing the oil bath, the reaction mixture was cooled to rt and was then directly applied to a silica column (h = 6 cm, d = 2 cm, preconditioned with acetone). The column was washed with acetone (200 mL) and the product was eluted with acetone / AcOH (9/1, 50 mL). The solvent was removed, and the crude product was further purified by RP-MPLC [C18AQ, H_2O/MeCN/TEAA (10 mM)]. The product containing fractions were identified by LC-MS and the products were isolated after lyophilization.

Synthesis of phenyl phosphate (27)

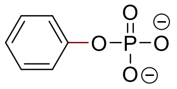
27

2.63 TBA / 1.66 acetate

The compound was synthesized according to the general procedure A from phosphate x 1.05 TBA (**17**, 316 mg, 900 µmol, 3.0 eq) and 2-(trimethylsilyl)phenyl trifluoromethanesulfonate (**1**, 90 mg, 300 µmol). The product (**27**, 241 mg, 265 µmol, 88%) was isolated as a colorless oil.

^{1}H-NMR (400 MHz, D_2O, δ/ppm): 7.48 – 7.32 (m, 2H), 7.26 – 7.11 (m, 3H), 3.22 – 3.14 (m, 21H), 1.63 (ddd, *J* = 12.0, 10.0, 6.2 Hz, 21H), 1.35 (h, *J* = 7.4 Hz, 21H), 0.94 (t, *J* = 7.4 Hz, 32H). **^{13}C{^{1}H}-NMR** (101 MHz, D_2O, δ/ppm): 152.22 (d, *J* = 6.6 Hz), 129.64, 123.91, 120.45 (d, *J* = 4.3 Hz), 58.13 – 58.01 (m), 23.10, 19.12, 12.80. **^{31}P{^{1}H}-NMR** (162 MHz, D_2O, δ/ppm): -3.53. **HRMS** (ESI) m/z for $C_6H_6O_4P$ $[M-H]^-$: calcd. 173.0009, found 173.0010.

Synthesis of 2-naphthalen-2-yl phosphate (28)

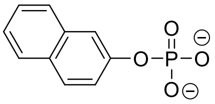
28

2.41 TBA / 1.33 acetate

The compound was synthesized according to the general procedure A from phosphate x 1.05 TBA (**17**, 316 mg, 900 µmol, 3.0 eq) and 3-(trimethylsilyl)naphthalen-2-yl

trifluoromethanesulfonate (**SI-12**, 105 mg, 300 µmol). The product (**28**, 173 mg, 195 µmol, 65%) was isolated as a colorless oil.

^{1}H-NMR (400 MHz, D_2O, δ/ppm): 7.95 – 7.73 (m, 3H), 7.67 (t, *J* = 2.1 Hz, 1H), 7.60 – 7.33 (m, 3H), 3.12 – 2.89 (m, 19H), 1.66 – 1.44 (m, 19H), 1.29 (h, *J* = 7.4 Hz, 19H), 0.91 (t, *J* = 7.4 Hz, 29H). **^{13}C{^{1}H}-NMR** (101 MHz, D_2O, δ/ppm): 150.43 (d, *J* = 6.8 Hz), 133.88, 129.97, 129.45, 127.71, 127.32, 126.71, 125.09, 121.55 (d, *J* = 4.6 Hz), 116.11 (d, *J* = 4.6 Hz), 62.59 – 45.12 (m), 23.02, 19.08, 12.82. **^{31}P{^{1}H}-NMR** (162 MHz, D_2O, δ/ppm): -3.43. **HRMS** (APCI) m/z for $C_{10}H_8O_4P$ [M-H]$^-$: calcd. 223.0166, found 223.0166.

Synthesis of 3,4-dimethylphenyl phosphate (29)

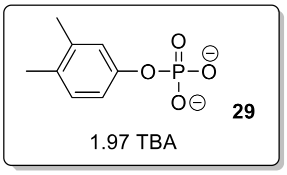

The compound was synthesized according to the general procedure A from phosphate x 1.05 TBA (**17**, 316 mg, 900 µmol, 3.0 eq) and 4,5-dimethyl-2-(trimethylsilyl)phenyl trifluoromethanesulfonate (**SI-6**, 98 mg, 300 µmol). The product (**29**, 204 mg, 277 µmol, 92%) was isolated as a colorless oil.

^{1}H-NMR (400 MHz, D_2O, δ/ppm): 7.17 – 7.10 (m, 1H), 7.01 (ddd, *J* = 2.4, 1.1, 0.5 Hz, 1H), 6.93 (dddd, *J* = 8.3, 2.6, 1.3, 0.6 Hz, 1H), 3.19 – 3.07 (m, 16H), 2.23 (s, 3H), 2.20 (s, 3H), 1.74 – 1.47 (m, 16H), 1.34 (h, *J* = 7.4 Hz, 16H), 1.07 – 0.78 (m, 24H). **^{13}C{^{1}H}-NMR** (101 MHz, D_2O, δ/ppm): 150.19 (d, *J* = 6.8 Hz), 138.26, 132.23 (d, *J* = 1.3 Hz), 130.25, 121.46 (d, *J* = 4.3 Hz), 117.54 (d, *J* = 4.2 Hz), 64.51 – 53.21 (m), 23.08, 19.17 – 19.07 (m), 19.01, 18.13, 12.83. **^{31}P{^{1}H}-NMR** (162 MHz, D_2O, δ/ppm): -3.68. **HRMS** (APCI) m/z for $C_8H_{10}O_4P$ [M-H]$^-$: calcd. 201.0322, found 201.0323.

Synthesis of 1*H*-indol-5-yl phosphate (30)

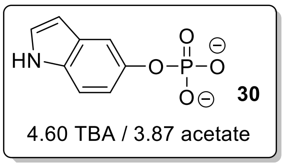

The compound was synthesized according to the general procedure A from phosphate x 1.05 TBA (**17**, 316 mg, 900 µmol, 3.0 eq) and 4-(trimethylsilyl)-1*H*-indol-5-yl

trifluoromethanesulfonate (101 mg, 300 µmol). The crude product was obtained as a 81:19 mixture (5-**30**:4-**30**). The product (**30**, 190 mg, 122 µmol, 41%) was isolated as a 96:4 mixture (5-**30**:4-**30**) as a light green solid. The NMR data are given for the major isomer.

^{1}H-NMR (400 MHz, D_2O, δ/ppm): 7.48 – 7.43 (m, 3H), 7.40 (s, 1H), 7.09 – 7.04 (m, 1H), 3.28 – 2.98 (m, 37H), 1.80 – 1.51 (m, 37H), 1.34 (h, *J* = 7.4 Hz, 37H), 0.94 (t, *J* = 7.4 Hz, 55H). **^{13}C{^{1}H}-NMR** (101 MHz, D_2O, δ/ppm): 145.54 (d, *J* = 7.0 Hz), 132.73, 127.62, 126.69, 115.62 (d, *J* = 3.8 Hz), 111.97, 110.97 (d, *J* = 4.1 Hz), 100.98, 60.10 – 56.30 (m), 23.07, 19.10, 12.80. **^{31}P{^{1}H}-NMR** (122 MHz, D_2O, δ/ppm): -3.28, -3.59. **HRMS** (ESI) m/z for $C_8H_7O_4NP$ $[M-H]^-$: calcd. 212.0118, found 212.0118.

Synthesis of pyren-2-yl phosphate (31)

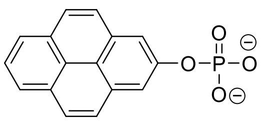
31

4.75 TBA / 2.87 acetate

The compound was synthesized according to the general procedure A from phosphate x 1.05 TBA (**17**, 316 mg, 900 µmol, 3.0 eq) and 2-(trimethylsilyl)pyren-1-yl trifluoromethanesulfonate (**SI-19**, 127 mg, 300 µmol). The crude product was obtained as a 76:24 mixture (2-**31**:1-**31**). The product (**31**, 334 mg, 214 µmol, 71%) was isolated as a 62:38 mixture (2-**31**:1-**31**) as a light green oil. The ^{1}H NMR and ^{31}P NMR data are given for the mixture and the ^{13}C NMR data are given for the major isomer.

^{1}H-NMR (400 MHz, CD_3CN, δ/ppm): 8.53 (d, *J* = 9.2 Hz, 0.6H), 8.29 (dd, *J* = 8.5, 1.0 Hz, 0.6H), 8.20 – 8.13 (m, 4H), 8.13 (dd, *J* = 7.6, 1.2 Hz, 0.6H), 8.10 – 8.03 (m, 1.3H), 8.00 (s, 4H), 7.99 – 7.90 (m, 3.6H), 3.17 – 2.82 (m, 38H), 1.66 – 1.48 (m, 38H), 1.38 – 1.17 (m, 38H), 0.93 (t, *J* = 7.3 Hz, 57H). **^{13}C{^{1}H}-NMR** (101 MHz, CD_3CN, δ/ppm): 153.51 (d, *J* = 7.1 Hz), 133.12, 131.35, 128.57, 127.97, 126.29, 126.03, 117.74 (d, *J* = 5.1 Hz), 62.39 – 57.17 (m), 24.16, 22.20 – 18.70 (m), 13.70. **^{31}P{^{1}H}-NMR** (122 MHz, CD_3CN, δ/ppm): -3.31, -3.75. **HRMS** (ESI) m/z for $C_{16}H_{10}O_4P$ $[M-H]^-$: calcd. 297.0322, found 297.0320.

Deuteration experiment using deuterated P_i (17-D)

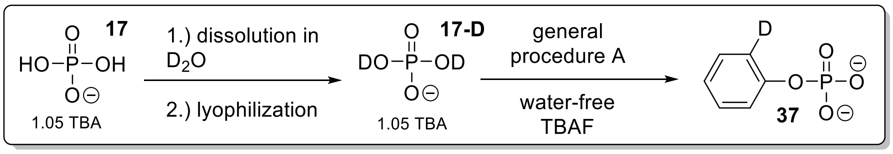

P_i x 1.05 TBA (500 mg) was dissolved in D_2O (3.0 ml) and the resulting solution was incubated for 30 min at room temperature. Subsequently the solution was lyophilized to dryness. The resulting solid was applied as starting material in general procedure A. In this case, the TBAF-solution was stored over molecular sieves (3 Å) for 5 h before the reaction to reduce the water content. The deuteration ratio of the product was determined by HRMS.

HRMS (ESI) m/z for $C_6H_5{}^2HO_4P$ $[M-H]^-$: calcd. 174.0072, found 174.0072.

General procedure B for the synthesis of pyrophosphomonoesters:

The pyrophosphate x TBA salt (1.50 mmol, 5.0 eq) was dissolved in dry MeCN (67 mM) before the aryne-precursor (300 µmol) was added. Subsequently TBAF (1 M in THF, 1.1 eq.) was added dropwise via syringe pump (1 h, rt, needle tip is below solvent surface). The reaction mixture was stirred 15 min at rt and was then diluted with Et_2O (15 mL) and H_2O (15 mL) and transferred to a separation funnel. The layers were separated, and the aqueous layer was washed with Et_2O (2 × 10 mL). Then, the combined organic layers were back-extracted with H_2O (5 × 10 mL) and the combined aqueous layers were lyophilized. The residue was further purified by RP-MPLC [C18AQ, H_2O/MeCN/TEAA (10 mM)]. The product containing fractions were identified by LC-MS and the products were isolated after lyophilization.

Synthesis of phenyl diphosphate (32)

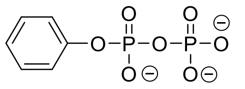
32

3.80 TBA / 0.14 TEA / 2.16 acetate

The compound was synthesized according to the general procedure B from pyrophosphate x 2.39 TBA (**18**, 1.13 g, 1.50 mmol, 5.0 eq) and 2-(trimethylsilyl)phenyl trifluoromethanesulfonate (**1**, 90 mg, 300 µmol). The product (**32**, 358 mg, 272 µmol, 91%) was isolated as a colorless oil.

^{1}H-NMR (400 MHz, D_2O, δ/ppm): 7.50 – 7.36 (m, 2H), 7.32 – 7.15 (m, 3H), 3.51 – 2.96 (m, 27H), 1.98 (s, 5H), 1.81 – 1.57 (m, 31H), 1.37 (h, *J* = 7.7 Hz, 31H), 1.30 – 1.25 (m, 1H), 0.96 (t, *J* = 7.3 Hz, 46H). **^{13}C{^{1}H}-NMR** (101 MHz, D_2O, δ/ppm): 151.84 (d, *J* = 7.2 Hz), 129.64, 124.25 (d, *J* = 1.3 Hz), 120.60 (d, *J* = 4.4 Hz), 59.88 – 54.25 (m), 46.61, 23.10, 20.81 – 17.49 (m), 12.80, 8.20. **^{31}P{^{1}H}-NMR** (122 MHz, D_2O, δ/ppm): -10.90 (d, *J* = 20.6 Hz), -15.80 (d, *J* = 20.7 Hz). **HRMS** (ESI) m/z for $C_6H_7O_7P_2$ $[M-H]^-$: calcd. 252.9672, found 252.9675.

Synthesis of 2-naphthalen-2-yl diphosphate (33)

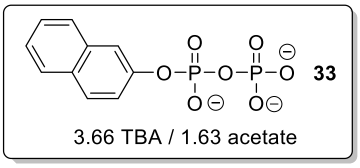

The compound was synthesized according to the general procedure B from pyrophosphate x 2.30 TBA (**18**, 1.10 g, 1.50 mmol, 5.0 eq) and 3-(trimethylsilyl)naphthalen-2-yl trifluoromethanesulfonate (**SI-12**, 105 mg, 300 µmol). The product (**33**, 239 mg, 186 µmol, 62%) was isolated as a colorless oil.

^{1}H-NMR (400 MHz, D_2O, δ/ppm): 8.03 – 7.90 (m, 3H), 7.75 (t, *J* = 2.1 Hz, 1H), 7.66 – 7.39 (m, 3H), 3.34 – 2.82 (m, 29H), 1.73 – 1.45 (m, 29H), 1.32 (h, *J* = 7.4 Hz, 29H), 0.92 (t, *J* = 7.4 Hz, 44H). **^{13}C{^{1}H}-NMR** (101 MHz, D_2O, δ/ppm): 149.86 (d, *J* = 7.3 Hz), 133.81, 130.21, 129.52, 127.73, 127.49, 126.76, 125.31, 121.47 (d, *J* = 4.7 Hz), 116.54 (d, *J* = 4.7 Hz), 59.80 – 53.72 (m), 23.05, 19.10 (t, *J* = 1.6 Hz), 12.82. **^{31}P{^{1}H}-NMR** (122 MHz, D_2O, δ/ppm): -10.84 (d, *J* = 20.7 Hz), -15.94 (d, *J* = 20.7 Hz). **HRMS** (ESI) m/z for $C_{10}H_9O_7P_2$ [M-H]$^-$: calcd. 302.9829, found 302.9830.

Synthesis of 3,4-dimethylphenyl diphosphate (34)

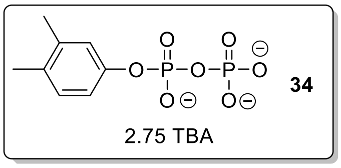

The compound was synthesized according to the general procedure B from pyrophosphate x 2.39 TBA (**18**, 1.13 g, 1.50 mmol, 5.0 eq) and 4,5-dimethyl-2-(trimethylsilyl)phenyl trifluoromethanesulfonate (**SI-6**, 98 mg, 300 µmol). The product (**34**, 281 mg, 281 µmol, 94%) was isolated as a colorless oil.

^{1}H-NMR (400 MHz, D_2O, δ/ppm): 7.19 – 7.13 (m, 1H), 7.07 (ddd, *J* = 2.5, 1.5, 0.9 Hz, 1H), 6.99 (dddd, *J* = 8.3, 2.7, 1.2, 0.6 Hz, 1H), 3.31 – 3.04 (m, 22H), 2.25 (s, 3H), 2.22 (s, 3H), 1.75 – 1.51 (m, 22H), 1.35 (h, *J* = 7.4 Hz, 22H), 1.08 – 0.72 (m, 33H). **^{13}C{^{1}H}-NMR** (101 MHz, D_2O, δ/ppm): 149.84 (d, *J* = 7.2 Hz), 138.31, 132.59 (d, *J* = 1.4 Hz), 130.24, 121.56 (d, *J* = 4.5 Hz), 117.66 (d, *J* = 4.3 Hz), 59.22 – 56.56 (m), 23.09, 19.12 (t, *J* = 1.6 Hz), 18.99, 18.15, 12.83. **^{31}P{^{1}H}-NMR** (162 MHz, D_2O, δ/ppm): -10.90 (d, *J* = 20.5 Hz), -15.72 (d, *J* = 21.1 Hz). **HRMS** (ESI) m/z for $C_8H_{11}O_7P_2$ [M-H]$^-$: calcd. 280.9985, found 280.9987.

Synthesis of 1*H*-indol-5-yl diphosphate (35)

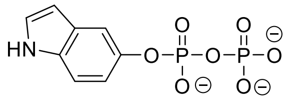

35

5.00 TBA / 0.14 TEA / 3.27 acetate

The compound was synthesized according to the general procedure B from pyrophosphate x 2.30 TBA (**18**, 1.10 g, 1.50 mmol, 5.0 eq) and 4-(trimethylsilyl)-1*H*-indol-5-yl trifluoromethanesulfonate (101 mg, 300 µmol). The crude product was obtained as a 88:12 mixture (5-**35**:4-**35**). The product (**35**, 216 mg, 126 µmol, 42%) was isolated as a 96:4 mixture (5-**35**:4-**35**) as a light green oil. The NMR data are given for the major isomer.

^{1}**H-NMR** (300 MHz, D_2O, δ/ppm): 7.51 (ddd, *J* = 2.3, 1.6, 0.6 Hz, 1H), 7.50 – 7.45 (m, 1H), 7.45 – 7.39 (m, 1H), 7.14 (dddd, *J* = 8.8, 2.4, 1.2, 0.4 Hz, 1H), 6.58 (dd, *J* = 3.1, 0.9 Hz, 1H), 3.30 – 2.89 (m, 41H), 1.63 (dq, *J* = 11.7, 7.7 Hz, 40H), 1.36 (h, *J* = 7.4 Hz, 40H), 1.26 (t, *J* = 7.3 Hz, 1H), 0.95 (t, *J* = 7.3 Hz, 60H). ^{13}**C{^{1}H}-NMR** (101 MHz, D_2O, δ/ppm): 145.44 (d, *J* = 7.4 Hz), 132.80, 127.58, 126.62, 115.72 (d, *J* = 3.9 Hz), 111.92, 111.18 (d, *J* = 4.3 Hz), 101.30, 59.29 – 55.57 (m), 23.07, 19.11 (t, *J* = 1.5 Hz), 12.82. ^{31}**P{^{1}H}-NMR** (162 MHz, D_2O, δ/ppm): -10.74 (d, *J* = 20.5 Hz), -15.01 (d, *J* = 20.4 Hz). **HRMS** (ESI) m/z for $C_8H_7{}^2HO_7P_2$ [M-H]$^-$: calcd. 292.9844, found 292.9845.

Synthesis of pyren-2-yl diphosphate (36)

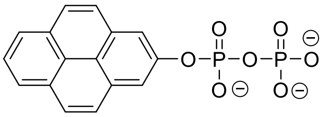

36

3.77 TBA / 1.21 acetate

The compound was synthesized according to the general procedure B from pyrophosphate x 2.39 TBA (**18**, 1.13 g, 1.50 mmol, 5.0 eq) and 2-(trimethylsilyl)pyren-1-yl trifluoromethanesulfonate (**SI-19**, 127 mg, 300 µmol). The crude product was obtained as a 86:14 mixture (2-**36**:1-**36**). The product (**36**, 379 mg, 278 µmol, 93%) was isolated as an 82:18 mixture (2-**36**:1-**36**) as a light green oil. The NMR data are given for the major isomer.

^{1}**H-NMR** (400 MHz, D_2O, δ/ppm): 8.17 – 8.13 (m, 2H), 8.07 (d, *J* = 9.1 Hz, 2H), 7.91 (t, *J* = 8.8 Hz, 4H), 7.71 (t, *J* = 7.6 Hz, 1H), 2.81 – 2.58 (m, 30H), 1.52 – 1.22 (m, 30H), 1.14 (h, *J* = 7.3 Hz, 30H), 0.81 (t, *J* = 7.3 Hz, 45H). ^{13}**C{^{1}H}-NMR** (101 MHz, D_2O, δ/ppm): 150.68 (d, *J* = 6.8 Hz), 132.08, 130.20, 127.85, 127.46, 125.76, 125.27, 123.82, 120.80, 117.11 (d, *J* = 4.9 Hz),

57.68 (t, J = 2.8 Hz), 22.83, 18.96, 12.80. **^{31}P{^{1}H}-NMR** (162 MHz, D_2O, δ/ppm): -10.63 (d, J = 19.4 Hz), -16.18 (d, J = 19.9 Hz). **HRMS** (ESI) m/z for $C_{16}H_{11}O_7P_2$ [M-H]$^-$: calcd. 376.9985, found 376.9987.

5. Cluster 2A: Synthesis of (Pyro-)phosphodiesters (aryne-Scope)

General procedure C for the synthesis of phosphodiesters:

The phosphate x TBA salt (150 - 500 µmol) was dissolved in dry MeCN (200 mM) before the aryne-precursor (2.5 eq.) was added. Subsequently TBAF (1 M in THF, 2.5 eq.) was added dropwise via syringe pump (1 h, rt, needle tip is below solvent surface). Subsequently the reaction mixture is stirred for 15 min at rt and directly applied to a silica column (h = 6 cm, d = 2 cm, preconditioned with acetone). The column was washed with acetone (200 mL) and the product was eluted with acetone / AcOH (9/1, 50 mL). The solvent was removed, and the crude product was further purified by RP-MPLC [C18AQ, H_2O/MeCN/TEAA (10 mM)]. The product containing fractions were identified by LC-MS and the products were isolated after lyophilization.

5,5,5-Trifluoropentyl-phenylphosphate (40)

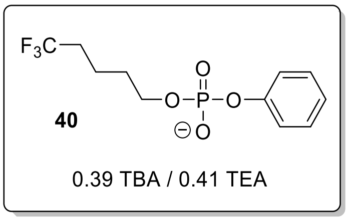

The compound was synthesized according to the general procedure C from 5,5,5,-trifluoropentylphosphate x 1.5 TBA (**38**, 173 mg, 300 µmol) and 2-(trimethylsilyl)phenyl trifluoromethanesulfonate (**1**, 182 µl, 223 mg, 750 µmol, 2.5 eq.). The product (**40**, 66.0 mg, 152 µmol, 51%) was isolated as colorless oil.

^{1}H-NMR (400 MHz, CD_3CN, δ/ppm): 7.32 – 7.24 (m, 2H), 7.22 – 7.16 (m, 2H), 7.08 – 7.01 (m, 1H), 3.91 (dt, J = 6.8, 6.0 Hz, 2H), 3.13 – 3.04 (m, 3H), 2.95 (q, J = 7.3 Hz, 2H), 2.21 – 2.07 (m, 2H), 1.69 – 1.52 (m, 7H), 1.40 – 1.29 (m, 3H), 1.19 (t, J = 7.3 Hz, 4H), 0.96 (t, J = 7.4 Hz, 4H). **^{13}C{^{1}H}-NMR** (101 MHz, CD_3CN, δ/ppm): 154.36 (d, J = 6.5 Hz), 130.12, 123.82, 121.14 (d, J = 5.0 Hz), 65.94 (d, J = 6.1 Hz), 59.31, 46.53, 33.58 (q, J = 28.0 Hz), 30.25 (d, J = 7.5

Hz), 24.31, 20.34, 19.24 (q, *J* = 3.3 Hz), 13.79, 8.88. **^{19}F NMR** (377 MHz, CD_3CN, δ/ppm): -66.98 (t, *J* = 11.4 Hz). **^{31}P{^{1}H}-NMR** (162 MHz, CD_3CN, δ/ppm): -6.72. **HRMS** (ESI) m/z for $C_{11}H_{13}F_3O_4P$ [M-H]$^-$: calcd. 297.0509, found 297.0507.

Synthesis of 3,4-dimethylphenyl (5,5,5-trifluoropentyl) phosphate (41)

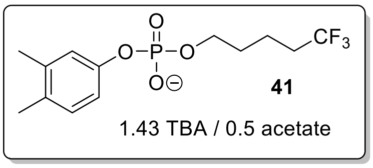

The compound was synthesized according to the general procedure C from 5,5,5-trifluoropentyl phosphate x 1.22 TBA (**38**, 155 mg, 300 µmol) and 4,5-dimethyl-2-(trimethylsilyl)phenyl trifluoromethanesulfonate (**SI-6**, 245 mg, 750 µmol, 2.5 eq). The product (**41**, 142 mg, 201 µmol, 67%) was isolated as a colorless oil.

^{1}H-NMR (400 MHz, CD_3CN, δ/ppm): 7.00 – 6.94 (m, 2H), 6.93 – 6.85 (m, 1H), 3.93 – 3.72 (m, 2H), 3.23 – 2.93 (m, 11H), 2.19 (s, 3H), 2.16 (s, 3H), 2.16 – 2.06 (m, 2H), 1.69 – 1.49 (m, 15H), 1.34 (h, *J* = 7.4 Hz, 11H), 0.96 (t, *J* = 7.3 Hz, 17H). **^{13}C{^{1}H}-NMR** (101 MHz, CD_3CN, δ/ppm): 153.58 (d, *J* = 6.6 Hz), 137.71, 130.47, 130.35, 128.74 (q, *J* = 275.5 Hz), 122.10 (d, *J* = 4.7 Hz), 118.16, 64.85 (d, *J* = 6.1 Hz), 60.82 – 54.61 (m), 33.58 (q, *J* = 27.9 Hz), 30.47 (d, *J* = 7.3 Hz), 24.26, 21.63 – 19.39 (m), 19.90, 19.36 (q, *J* = 3.1 Hz), 18.90, 13.74. **^{19}F NMR** (377 MHz, CD_3CN, δ/ppm): -66.97 (t, *J* = 11.1 Hz). **^{31}P{^{1}H}-NMR** (162 MHz, CD_3CN, δ/ppm): -5.40. **HRMS** (ESI) m/z for $C_{13}H_{17}F_3O_4P$ [M-H]$^-$: calcd. 325.0822, found 325.0820.

Synthesis of 2,5-dimethylphenyl (5,5,5-trifluoropentyl) phosphate (42)

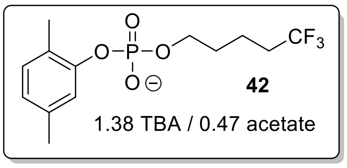

The compound was synthesized according to the general procedure C from 5,5,5-trifluoropentyl phosphate x 1.47 TBA (**38**, 173 mg, 300 µmol) and 3,6-dimethyl-2-(trimethylsilyl)phenyl trifluoromethanesulfonate (**SI-7**, 245 mg, 750 µmol, 2.5 eq). The product (**42**, 124 mg, 180 µmol, 60%) was isolated as a colorless oil.

^{1}H-NMR (400 MHz, CD_3CN, δ/ppm): 7.22 (s, 1H), 6.96 (d, *J* = 7.5 Hz, 1H), 6.70 – 6.59 (m, 1H), 4.03 – 3.56 (m, 2H), 3.15 – 3.04 (m, 11H), 2.23 (d, *J* = 0.7 Hz, 3H), 2.22 – 2.07 (m, 5H),

1.71 – 1.48 (m, 15H), 1.44 – 1.16 (m, 11H), 0.96 (t, *J* = 7.3 Hz, 17H). **^{13}C{^{1}H}-NMR** (101 MHz, CD_3CN, δ/ppm): 153.71 (d, *J* = 6.7 Hz), 136.61, 130.74, 130.13 (q), 126.44 (d, *J* = 6.4 Hz), 123.03, 121.45 (d, *J* = 2.5 Hz), 64.93 (d, *J* = 6.3 Hz), 60.62 – 54.40 (m), 33.62 (q, *J* = 27.9 Hz), 30.53 (d, *J* = 7.3 Hz), 24.25, 21.15, 20.64 – 19.62 (m), 19.41 (q, *J* = 3.2 Hz), 16.51, 13.74. **^{19}F NMR** (377 MHz, CD_3CN, δ/ppm): -66.95 (t, *J* = 11.4 Hz). **^{31}P{^{1}H}-NMR** (162 MHz, CD_3CN, δ/ppm): -5.26. **HRMS** (ESI) m/z for $C_{13}H_{17}F_3O_4P$ [M-H]$^-$: calcd. 325.0822, found 325.0821.

Synthesis of 3,4-dimethoxyphenyl (5,5,5-trifluoropentyl) phosphate (43)

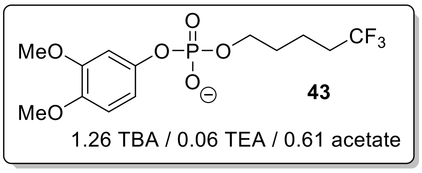

The compound was synthesized according to the general procedure C from 5,5,5-trifluoropentyl phosphate x 1.22 TBA (**38**, 155 mg, 300 µmol) and 4,5-dimethoxy-2-(trimethylsilyl)phenyl trifluoromethanesulfonate (**SI-8**, 269 mg, 750 µmol, 2.5 eq). The product (**43**, 121 mg, 172 µmol, 57%) was isolated as a colorless oil.

^{1}H-NMR (400 MHz, CD_3CN, δ/ppm): 6.87 (dd, *J* = 2.6, 0.9 Hz, 1H), 6.77 (d, *J* = 8.7 Hz, 1H), 6.70 (ddd, *J* = 8.7, 2.6, 1.1 Hz, 1H), 3.84 – 3.76 (m, 2H), 3.75 (s, 3H), 3.73 (s, 3H), 3.13 – 3.01 (m, 11H), 2.26 – 2.05 (m, 2H), 1.68 – 1.48 (m, 15H), 1.44 – 1.24 (m, 11H), 0.96 (t, *J* = 7.4 Hz, 15H), 0.89 (t, *J* = 7.2 Hz, 0.5H). **^{13}C{^{1}H}-NMR** (101 MHz, CD_3CN, δ/ppm): 150.26, 149.80 (d, *J* = 6.5 Hz), 145.07, 128.74 (q, *J* = 275.6 Hz), 113.15, 111.93 (d, *J* = 4.7 Hz), 106.22 (d, *J* = 4.9 Hz), 64.95 (d, *J* = 6.3 Hz), 61.17 – 57.30 (m), 56.80, 56.16, 33.59 (q, *J* = 27.9 Hz), 30.48 (d, *J* = 7.3 Hz), 24.25, 21.75 – 19.05 (m), 19.36 (q, *J* = 3.2 Hz), 13.73. **^{19}F NMR** (377 MHz, CD_3CN, δ/ppm): -66.95 (t, *J* = 11.4 Hz). **^{31}P{^{1}H}-NMR** (162 MHz, CD_3CN, δ/ppm): -5.36. **HRMS** (ESI) m/z for $C_{13}H_{17}F_3O_6P$ [M-H]$^-$: calcd. 357.0720, found 357.0721.

Synthesis of 4-(trifluoromethyl)phenyl (5,5,5-trifluoropentyl) phosphate (44)

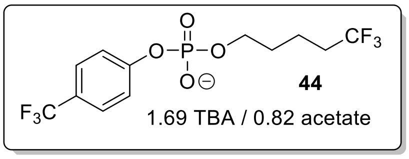

The compound was synthesized according to the general procedure C from 5,5,5-trifluoropentyl phosphate x 1.22 TBA (**38**, 155 mg, 300 µmol) and 5-(trifluoromethyl)-2-(trimethylsilyl)phenyl trifluoromethanesulfonate (**SI-11**, 275 mg, 750 µmol, 2.5 eq). The crude product was obtained as a 68:32 mixture (*para*:*meta*). The product (**44**, 116 mg, 149 µmol, 50%) was isolated as a 85:15 mixture (*para*:*meta*) as a colorless oil. The NMR data are given for the major isomer.

^{1}H-NMR (400 MHz, CD_3CN, δ/ppm): 7.60 – 7.52 (m, 2H), 7.48 – 7.33 (m, 2H), 3.92 – 3.77 (m, 2H), 3.17 – 3.03 (m, 14H), 2.24 – 2.04 (m, 2H), 1.70 – 1.47 (m, 18H), 1.46 – 1.23 (m, 14H), 0.96 (t, *J* = 7.3 Hz, 20H). **^{13}C{^{1}H}-NMR** (101 MHz, CD_3CN, δ/ppm): 158.46 (d, *J* = 6.1 Hz), 128.68 (q, *J* = 275.5 Hz), 127.27 – 127.10 (m), 125.74 (q, *J* = 270.4 Hz), 123.98 (q, *J* = 32.2 Hz), 121.10 (d, *J* = 5.0 Hz), 65.40 (d, *J* = 6.3 Hz), 59.79 – 54.70 (m), 35.90 – 32.26 (m), 30.29 (d, *J* = 7.4 Hz), 24.25, 21.09 – 19.13 (m), 19.26 (q, *J* = 3.3 Hz), 13.73. **^{19}F-NMR** (377 MHz, CD_3CN, δ/ppm): -61.94 (*para*), -62.98 (*meta*), -66.94 – -67.13 (m, both). **^{31}P{^{1}H}-NMR** (162 MHz, CD_3CN, δ/ppm): -1.32. **HRMS** (ESI) m/z for $C_{12}H_{12}F_6O_4P$ [M-H]$^-$: calcd. 365.0383, found 365.0381.

Synthesis of benzo[*d*][1,3]dioxol-5-yl (5,5,5-trifluoropentyl) phosphate (45)

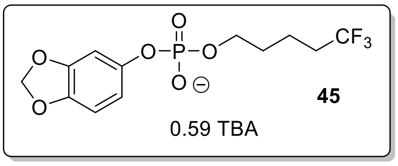

The compound was synthesized according to the general procedure C from 5,5,5-trifluoropentyl phosphate x 1.22 TBA (**38**, 155 mg, 300 µmol) and 6-(trimethylsilyl)benzo[*d*][1,3]dioxol-5-yl trifluoromethanesulfonate (**SI-9**, 257 mg, 750 µmol, 2.5 eq). The product (**45**, 89 mg, 184 µmol, 61%) was isolated as a colorless oil.

^{1}H-NMR (400 MHz, CD_3CN, δ/ppm): 6.79 (dd, *J* = 2.2, 0.8 Hz, 1H), 6.70 (d, *J* = 8.4 Hz, 1H), 6.64 (ddd, *J* = 8.4, 2.3, 1.1 Hz, 1H), 5.91 (s, 2H), 4.22 – 3.79 (m, 2H), 3.25 – 2.89 (m, 5H), 2.26 – 2.03 (m, 2H), 1.71 – 1.49 (m, 9H), 1.46 – 1.27 (m, 5H), 0.96 (t, *J* = 7.3 Hz, 7H). **^{13}C{^{1}H}-**

NMR (101 MHz, CD_3CN, δ/ppm): 148.76 (d, *J* = 6.7 Hz), 148.64, 144.11, 128.69 (q, *J* = 275.6 Hz), 113.20 (d, *J* = 4.9 Hz), 108.38, 103.50 (d, *J* = 4.5 Hz), 102.47, 66.01 (d, *J* = 6.0 Hz), 63.00 – 54.55 (m), 33.52 (q, *J* = 28.0 Hz), 30.17 (d, *J* = 7.4 Hz), 24.24, 21.30 – 19.63 (m), 19.14 (q, *J* = 3.3 Hz), 13.72. ^{19}**F NMR** (377 MHz, CD_3CN, δ/ppm): -66.98 (t, *J* = 11.4 Hz). ^{31}**P{^{1}H}-NMR** (162 MHz, CD_3CN, δ/ppm): -6.49. **HRMS** (ESI) m/z for $C_{12}H_{13}F_3O_6P$ [M-H]$^-$: calcd. 341.0407, found 341.0403.

Synthesis of 4-ethynylphenyl (5,5,5-trifluoropentyl) phosphate (46)

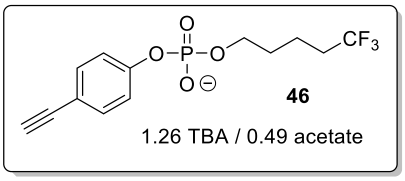

The compound was synthesized according to the general procedure C from 5,5,5-trifluoropentyl phosphate x 1.22 TBA (**38**, 155 mg, 300 µmol) and 2-(trimethylsilyl)-4-((trimethylsilyl)ethynyl)phenyl trifluoromethanesulfonate (**SI-23**, 253 mg, 750 µmol, 2.5 eq). The crude product was obtained as a 84:16 mixture (*para*:*meta*). The product (**46**, 127 mg, 193 µmol, 64%) was isolated as a 63:37 mixture (*para*:*meta*) as a colorless oil. The NMR data are given for the mixture, an assignment to the isomers was made if possible.

^{1}**H-NMR** (400 MHz, CD_3CN, δ/ppm): 7.39 – 7.31 (m, 1.5H, both), 7.22 – 7.16 (m, 1.9H, both), 7.11 – 7.04 (m, 0.4H, *meta*), 3.85 – 3.76 (m, 2H, both), 3.33 (s, 0.4H, *meta*), 3.26 (s, 0.6H, *para*), 3.16 – 3.03 (m, 10H, both), 2.23 – 2.02 (m, 2H, both), 1.68 – 1.46 (m, 14H, both), 1.44 – 1.24 (m, 10H, both), 1.05 – 0.90 (m, 15H, both). ^{13}**C{^{1}H}-NMR** (101 MHz, CD_3CN, δ/ppm): 156.48 (d, *J* = 6.4 Hz, *para*), 155.69 (d, *J* = 6.4 Hz, *meta*), 133.70 (*para*), 130.00 (*meta*), 128.73 (q, *J* = 275.6 Hz, both), 126.02 (*para*), 123.18 (*meta*), 122.02 (d, *J* = 5.1 Hz, *meta*), 120.94 (d, *J* = 5.2 Hz, *para*), 115.59, 84.50 (*para*), 84.23 (*meta*), 78.24 (*meta*), 77.21 (*para*), 65.06 (d, *J* = 6.3 Hz, both), 62.17 – 57.65 (m, both), 33.58 (qd, *J* = 28.0, 2.3 Hz, both), 30.42 (dd, *J* = 7.3, 3.1 Hz, both), 24.25 (both), 21.89 – 19.93 (m, both), 19.99 – 18.48 (m, both), 13.73 (both). ^{19}**F NMR** (377 MHz, CD_3CN, δ/ppm): -66.84 – -67.09 (m, both). ^{31}**P{^{1}H}-NMR** (162 MHz, CD_3CN, δ/ppm): -5.61 (*meta*), -5.77 (*para*). **HRMS** (ESI) m/z for $C_{13}H_{13}F_3O_4P$ [M-H]$^-$: calcd. 321.0509, found 321.0507.

Synthesis of 3-bromophenyl (5,5,5-trifluoropentyl) phosphate (47)

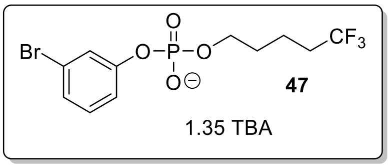

The compound was synthesized according to the general procedure C from 5,5,5-trifluoropentyl phosphate x 1.22 TBA (**38**, 155 mg, 300 µmol) and 2-bromo-6-(trimethylsilyl)phenyl trifluoromethanesulfonate (283 mg, 750 µmol, 2.5 eq). The crude product was obtained as a 88:12 mixture (*meta*:*ortho*). The product (**47**, 121 mg, 172 µmol, 57%) was isolated as a colorless oil in a similar regioisomeric ratio. Redundant TBA counterions are assumed to be hydroxide.

^{1}H-NMR (400 MHz, CD_3CN, δ/ppm): 7.45 (ddd, *J* = 1.7, 1.7, 0.9 Hz, 1H), 7.22 – 7.08 (m, 3H), 3.90 – 3.66 (m, 2H), 3.25 – 2.96 (m, 11H), 2.26 – 2.05 (m, 2H), 1.70 – 1.48 (m, 15H), 1.41 – 1.28 (m, 11H), 0.96 (t, *J* = 7.3 Hz, 16H). **^{13}C{^{1}H}-NMR** (101 MHz, CD_3CN, δ/ppm): 155.64 (d, *J* = 6.6 Hz), 130.29, 127.72 (q, *J* = 275.5 Hz), 124.48, 123.01 (d, *J* = 4.8 Hz), 121.33, 118.97 (d, *J* = 5.2 Hz), 64.28 (d, *J* = 6.1 Hz), 64.21 – 49.20 (m), 32.64 (q, *J* = 28.0 Hz), 29.44 (d, *J* = 7.3 Hz), 23.31, 21.02 – 18.82 (m), 18.37 (q, *J* = 3.1 Hz), 12.79. **^{19}F-NMR** (377 MHz, CD_3CN, δ/ppm): -66.99 (t, *J* = 11.3 Hz). **^{31}P{^{1}H}-NMR** (162 MHz, CD_3CN, δ/ppm): -6.02. **HRMS** (ESI) m/z for $C_{11}H_{12}BrF_3O_4P$ $[M-H]^-$: calcd. 374.9614, found 374.9613.

Synthesis of 4-chlorophenyl (5,5,5-trifluoropentyl) phosphate (48)

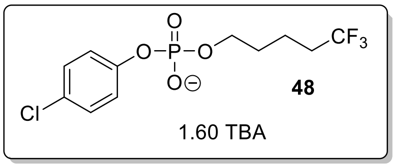

The compound was synthesized according to the general procedure C from 5,5,5-trifluoropentyl phosphate x 1.22 TBA (**38**, 155 mg, 300 µmol) and 5-chloro-2-(trimethylsilyl)phenyl trifluoromethanesulfonate (**SI-10**, 250 mg, 750 µmol, 2.5 eq). The crude product was obtained as a 78:22 mixture (*para*:*meta*). The product (**48**, 123 mg, 171 µmol, 57%) was isolated as a 81:19 mixture (*para*:*meta*) as a colorless oil. The NMR data are given for the major isomer. Redundant TBA-counterions are assumed to be hydroxide.

^{1}H-NMR (400 MHz, CD_3CN, δ/ppm): 7.20 (s, 4H), 3.86 – 3.71 (m, 2H), 3.16 – 3.02 (m, 13H), 2.20 – 2.06 (m, 2H), 1.68 – 1.46 (m, 17H), 1.45 – 1.20 (m, 13H), 0.96 (t, *J* = 7.3 Hz, 19H). **^{13}C{^{1}H}-NMR** (101 MHz, CD_3CN, δ/ppm): 154.67 (d, *J* = 6.6 Hz), 129.48, 128.73 (q, *J* = 275.6

Hz), 122.37 (d, J = 5.0 Hz), 122.23, 65.03 (d, J = 6.3 Hz), 61.89 – 53.86 (m), 33.57 (q, J = 28.0 Hz), 30.41 (d, J = 7.3 Hz), 24.25, 21.72 – 19.36 (m), 19.31 (q, J = 3.2 Hz), 13.73. **^{19}F NMR** (377 MHz, CD_3CN, δ/ppm): -66.99 (t, J = 11.4 Hz). **^{31}P{^{1}H}-NMR** (162 MHz, CD_3CN, δ/ppm): -5.53. **HRMS** (ESI) m/z for $C_{11}H_{12}ClF_3O_4P$ [M-H]$^-$: calcd. 331.0119, found 331.0119.

Synthesis of naphthalen-2-yl (5,5,5-trifluoropentyl) phosphate (49)

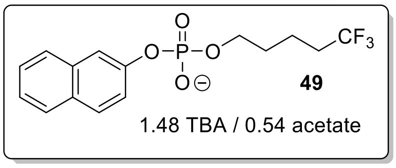

The compound was synthesized according to the general procedure C from 5,5,5-trifluoropentyl phosphate x 1.22 TBA (**38**, 155 mg, 300 µmol) and 3-(trimethylsilyl)naphthalen-2-yl trifluoromethanesulfonate (**SI-12**, 261 mg, 750 µmol, 2.5 eq). The product (**49**, 95 mg, 129 µmol, 43%) was isolated as a colorless oil.

^{1}H-NMR (400 MHz, CD_3CN, δ/ppm): 7.84 – 7.71 (m, 3H), 7.68 – 7.62 (m, 1H), 7.48 – 7.38 (m, 2H), 7.34 (ddd, J = 8.1, 6.8, 1.3 Hz, 1H), 3.90 – 3.82 (m, 2H), 3.13 – 3.03 (m, 12H), 2.20 – 2.02 (m, 2H), 1.71 – 1.47 (m, 16H), 1.48 – 1.24 (m, 12H), 0.95 (t, J = 7.3 Hz, 18H). **^{13}C{^{1}H}-NMR** (101 MHz, CD_3CN, δ/ppm): 153.45 (d, J = 6.4 Hz), 135.30, 130.36, 129.34, 128.70 (q, J = 275.5 Hz), 128.34, 127.79, 126.81, 124.70, 122.71 (d, J = 5.4 Hz), 115.88 (d, J = 5.0 Hz), 65.07 (d, J = 6.3 Hz), 61.18 – 55.22 (m), 33.56 (q, J = 27.8 Hz), 30.45 (d, J = 7.5 Hz), 24.23, 21.85 – 19.03 (m), 19.34 (q, J = 3.3 Hz), 13.73. **^{19}F NMR** (377 MHz, CD_3CN, δ/ppm): -66.98 (t, J = 11.4 Hz). **^{31}P{^{1}H}-NMR** (162 MHz, CD_3CN, δ/ppm): -5.44. **HRMS** (ESI) m/z for $C_{15}H_{15}F_3O_4P$ [M-H]$^-$: calcd. 347.0666, found 347.0666.

Synthesis of 1*H*-indol-5-yl (5,5,5-trifluoropentyl) phosphate (50)

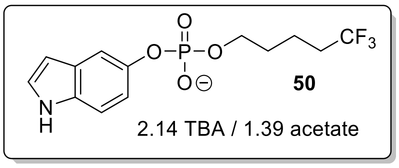

The compound was synthesized according to the general procedure C from 5,5,5-trifluoropentyl phosphate x 1.22 TBA (**38**, 155 mg, 300 µmol) and 4-(trimethylsilyl)-1*H*-indol-5-yl trifluoromethanesulfonate (253 mg, 750 µmol, 2.5 eq). The crude product was obtained as a 85:15 mixture (5-**50**:4-**50**). The product (**50**, 107 mg, 114 µmol, 38%) was isolated as a 92:8 mixture (5-**50**:4-**50**) as a light green solid. The NMR data are given for the major isomer.

^{1}H-NMR (400 MHz, CD_3CN, δ/ppm): 9.78 (s, 1H), 7.35 (td, *J* = 1.4, 0.6 Hz, 1H), 7.25 (dd, *J* = 8.7, 0.8 Hz, 1H), 7.18 (ddd, *J* = 3.0, 2.4, 0.4 Hz, 1H), 6.97 (dddd, *J* = 8.8, 2.3, 1.0, 0.4 Hz, 1H), 6.33 (ddd, *J* = 3.0, 2.0, 0.9 Hz, 1H), 3.89 – 3.77 (m, 2H), 3.17 – 2.96 (m, 17H), 2.24 – 2.05 (m, 2H), 1.66 – 1.49 (m, 21H), 1.42 – 1.24 (m, 17H), 0.96 (t, *J* = 7.3 Hz, 26H). **^{13}C{^{1}H}-NMR** (101 MHz, CD_3CN, δ/ppm): 149.00 (d, *J* = 6.8 Hz), 133.03, 129.08, 128.78 (q, *J* = 275.5 Hz), 126.20, 116.55 (d, *J* = 4.9 Hz), 111.79, 110.93 (d, *J* = 4.3 Hz), 101.92, 64.81 (d, *J* = 6.1 Hz), 33.62 (q, *J* = 27.9 Hz), 30.58 (d, *J* = 7.3 Hz), 24.25, 21.45 – 18.68 (m), 19.38 (q, *J* = 3.3 Hz), 13.73. **^{19}F NMR** (377 MHz, CD_3CN, δ/ppm): -66.96 (t, *J* = 11.3 Hz). **^{31}P{^{1}H}-NMR** (162 MHz, CD_3CN, δ/ppm): -4.70. **HRMS** (ESI) m/z for $C_{13}H_{14}F_3NO_4P$ $[M-H]^-$: calcd. 336.0618, found 336.0617.

Synthesis of pyren-2-yl (5,5,5-trifluoropentyl) phosphate (51)

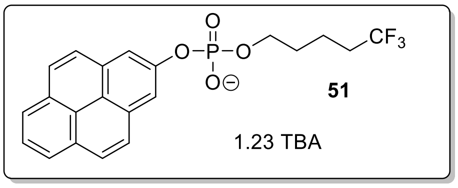

The compound was synthesized according to the general procedure C from 5,5,5-trifluoropentyl phosphate x 1.22 TBA (**38**, 155 mg, 300 µmol) and 2-(trimethylsilyl)pyren-1-yl trifluoromethanesulfonate (**SI-19**, 317 mg, 750 µmol, 2.5 eq). The crude product was obtained as a 78:22 mixture (2-**51**:1-**51**). The product (**51**, 125 mg, 174 µmol, 58%) was isolated as a 78:22 mixture (2-**51**:1-**51**) as a light-yellow oil. The NMR data are given for the major isomer. Redundant TBA counterions are assumed to be hydroxide.

^{1}H-NMR (400 MHz, CD_3CN, δ/ppm): 8.27 – 7.88 (m, 9H), 4.11 – 3.84 (m, 2H), 3.26 – 2.92 (m, 10H), 2.21 – 2.02 (m, 2H), 1.72 – 1.50 (m, 14H), 1.42 – 1.22 (m, 10H), 0.96 (t, *J* = 7.3 Hz, 15H). **^{13}C{^{1}H}-NMR** (101 MHz, CD_3CN, δ/ppm): 153.66 (d, *J* = 6.4 Hz), 133.09, 131.35, 128.67 (q, *J* = 275.5 Hz), 128.47, 128.01, 126.20, 125.97, 117.55 (d, *J* = 5.1 Hz), 65.39 (d, *J* = 6.0 Hz), 60.51 – 53.67 (m), 35.11 – 32.44 (m), 30.42 (d, *J* = 7.4 Hz), 24.23, 22.07 – 18.77 (m), 19.33 (q, *J* = 3.2 Hz), 13.72. **^{19}F NMR** (377 MHz, CD_3CN, δ/ppm): -67.05 (t, *J* = 11.3 Hz). **^{31}P{^{1}H}-NMR** (162 MHz, CD_3CN, δ/ppm): -5.93. **HRMS** (ESI) m/z for $C_{21}H_{17}F_3O_4P$ $[M-H]^-$: calcd. 421.0822, found 421.0819.

6. Cluster 2B: Synthesis of (Pyro-)phosphodiesters (phosphate-Scope)

Diphenylphosphate (52)

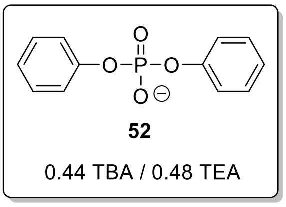

The compound was synthesized according to the general procedure C from phenylphosphate x 1.0 TBA (200 mg, 467 µmol) and 2-(trimethylsilyl)phenyl trifluoromethanesulfonate (**1**, 2.5 eq.). The product (**52**, 102 mg, 254 µmol, 55%) was isolated as colorless oil.

^{1}H-NMR (400 MHz, D_2O, δ/ppm): δ 7.45 – 7.38 (m, 4H), 7.27 – 7.20 (m, 6H), 3.27 – 3.12 (m, 6H), 1.71 – 1.59 (m, 4H), 1.48 – 1.32 (m, 3H), 1.28 (t, J = 7.4 Hz, 4H), 0.95 (t, J = 7.4 Hz, 5H). **^{13}C{^{1}H}-NMR** (101 MHz, D_2O, δ/ppm): 151.62 (d, J = 7.2 Hz), 129.80, 124.52 (d, J = 1.2 Hz), 120.22 (d, J = 4.6 Hz), 58.72 – 57.16 (m), 46.64, 23.10, 20.47 – 18.36 (m), 12.79, 8.19. **^{31}P{^{1}H}-NMR** (162 MHz, D_2O, δ/ppm): -8.85. **HRMS** (ESI) m/z for $C_{12}H_{10}O_4P$ $[M-H]^-$: calcd. 249.0322, found 249.0322.

Pentyl -phenylphosphate (53)

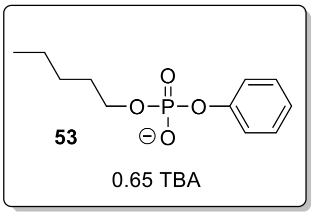

The compound was synthesized according to the general procedure C from pentylphosphate x 1.0 TBA (**67**, 123 mg, 300 µmol) and 2-(trimethylsilyl)phenyl trifluoromethanesulfonate (**1**, 2.5 eq.). The product (**53**, 0.65 TBA, 67.0 mg, 167 µmol, 56%) was isolated as colorless oil.

Alternative procedure (avoiding THF-side reaction):

TBAF (1 M in THF, 750 µL, 750 µmol, 2.5 eq.) was dried under high vacuum and dissolved in dry MeCN (750 µL). This was repeated twice and the resulting solution was then added to a solution of Pentylphosphate x 1.1 TBA (**67**, 130 mg, 300 mmol) and 2-(trimethylsilyl)phenyl trifluoromethanesulfonate (**1**, 182 µL, 750 µmol, 2.5 eq.) in MeCN (1.5 mL) with a syringe pump (1 h, needle tip is below solvent surface). Purification was done according to the general

procedure C. The product (**53**, 0.65 TEA, 67.2 mg, 216 µmol, 72%) was isolated as colorless oil.

^{1}H-NMR (400 MHz, CD_3CN, δ/ppm): 7.29 – 7.23 (m, 2H), 7.22 – 7.17 (m, 2H), 7.06 – 6.99 (m, 1H), 3.87 (q, *J* = 6.6 Hz, 2H), 3.15 – 3.04 (m, 5H), 1.65 – 1.50 (m, 2H), 1.41 – 1.22 (m, 9H), 0.96 (t, *J* = 7.3 Hz, 7H), 0.89 – 0.84 (m, 3H). **^{13}C{^{1}H}-NMR** (101 MHz, CD_3CN, δ/ppm): 154.47 (d, *J* = 6.5 Hz), 130.02, 123.61, 121.15 (d, *J* = 5.0 Hz), 66.66 (d, *J* = 6.3 Hz), 59.74 – 58.85 (m), 31.15 (d, *J* = 7.3 Hz), 28.74, 24.32, 23.09, 21.39 – 19.80 (m), 14.34, 13.82. **^{31}P{^{1}H}-NMR** (162 MHz, CD_3CN, δ/ppm): -6.80. **HRMS** (ESI) m/z for $C_{11}H_{16}O_4P$ $[M-H]^-$: calcd. 243,0792, found 243,0791.

Phenyl-phenylphosphonate (54)

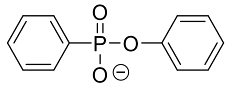

54

1.17 TBA / 0.52 acetate

The compound was synthesized according to the general procedure C from phenylphosphonate x 1.0 TBA (201 mg, 500 µmol) and 2-(trimethylsilyl)phenyl trifluoromethanesulfonate (**1**, 2.5 eq.). The product (**54**, 127 mg, 229 µmol, 46%) was isolated as colorless oil.

^{1}H-NMR (400 MHz, D_2O, δ/ppm): 7.81 – 7.73 (m, 2H), 7.61 – 7.55 (m, 1H), 7.54 – 7.47 (m, 2H), 7.34 – 7.27 (m, 2H), 7.18 – 7.12 (m, 1H), 7.04 – 6.99 (m, 2H), 3.25 – 3.11 (m, 9H), 1.70 – 1.57 (m, 9H), 1.36 (h, *J* = 7.4 Hz, 9H), 0.95 (t, *J* = 7.3 Hz, 14H). **^{13}C{^{1}H}-NMR** (101 MHz, D_2O, δ/ppm): 151.51 (d, *J* = 7.1 Hz), 132.66 (d, *J* = 181.1 Hz), 131.39 (d, *J* = 3.0 Hz), 131.18 (d, *J* = 9.5 Hz), 129.58, 128.38 (d, *J* = 14.1 Hz), 124.19 (d, *J* = 1.3 Hz), 121.02 (d, *J* = 3.8 Hz), 58.69 – 57.62 (m), 23.09, 19.51 – 17.77 (m), 12.79. **^{31}P{^{1}H}-NMR** (162 MHz, D_2O, δ/ppm): 12.99. **HRMS** (ESI) m/z for $C_{12}H_{10}O_3P$ $[M-H]^-$: calcd. 233.0373, found 233.0373.

Pent-4-yn-1-yl-phenylphosphate (55)

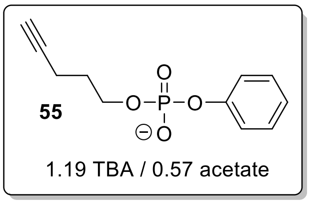

The compound was synthesized according to the general procedure C from pent-4-yn-1-ylphosphate x 1.0 TBA (**SI-3**, 203 mg, 500 µmol) and 2-(trimethylsilyl)phenyl trifluoromethanesulfonate (**1**, 2.5 eq.). The product (**55**, 136 mg, 242 µmol, 48%) was isolated as colorless oil.

^{1}H-NMR (400 MHz, D_2O/MeCN-d3, δ/ppm): 7.66 – 7.58 (m, 2H), 7.46 – 7.38 (m, 3H), 4.24 (q, *J* = 6.3 Hz, 2H), 3.46 – 3.33 (m, 10H), 2.58 (t, *J* = 2.7 Hz, 1H), 2.50 (td, *J* = 7.1, 2.7 Hz, 2H), 2.04 (ttd, *J* = 7.1, 6.1, 0.9 Hz, 2H), 1.85 (ddd, *J* = 11.8, 10.0, 6.3 Hz, 10H), 1.59 (h, *J* = 7.4 Hz, 10H), 1.19 (t, *J* = 7.4 Hz, 14H). **^{13}C{^{1}H}-NMR** (101 MHz, D_2O/MeCN-d3, δ/ppm): 152.43 (d, *J* = 6.8 Hz), 129.85, 124.09, 120.40 (d, *J* = 4.6 Hz), 84.85, 69.82, 65.00 (d, *J* = 5.9 Hz), 58.94 – 57.12 (m), 29.12 (d, *J* = 7.6 Hz), 23.36, 20.48 – 18.45 (m), 14.43, 13.09. **^{31}P{^{1}H}-NMR** (162 MHz, D_2O/MeCN-d3, δ/ppm): -4.34. **HRMS** (ESI) m/z for $C_{11}H_{12}O_4P$ [M-H]$^-$: calcd. 239.0479, found 239.0480.

Isoprenyl-phenylphosphate (56)

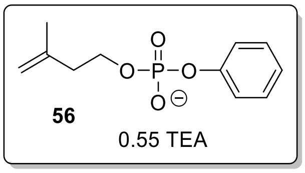

The compound was synthesized according to the general procedure C from isoprenylphosphate x 1.25 TBA (**SI-15**, 134 mg, 288 µmol) and 2-(trimethylsilyl)phenyl trifluoromethanesulfonate (**1**, 2.5 eq.). The product (**56**, 33.3 mg, 112 µmol, 39%) was isolated as colorless oil.

^{1}H-NMR (400 MHz, CD_3CN, δ/ppm): 7.33 – 7.27 (m, 2H), 7.22 – 7.17 (m, 2H), 7.12 – 7.06 (m, 1H), 4.77 (dqt, *J* = 2.2, 1.5, 0.7 Hz, 1H), 4.72 (dq, *J* = 2.2, 1.2 Hz, 1H), 4.04 (q, *J* = 6.8 Hz, 2H), 2.96 (qd, *J* = 7.3, 4.7 Hz, 3H), 2.32 (tdd, *J* = 6.8, 1.2, 0.6 Hz, 2H), 1.71 (td, *J* = 1.0, 0.5 Hz, 3H), 1.18 (t, *J* = 7.3 Hz, 5H). **^{13}C{^{1}H}-NMR** (101 MHz, CD_3CN, δ/ppm): 153.75 (d, *J* = 6.7 Hz), 143.49, 130.28, 124.40, 121.22 (d, *J* = 4.7 Hz), 112.50, 65.39 (d, *J* = 6.2 Hz), 46.75, 39.20 (d, *J* = 7.7 Hz), 22.56, 8.94. **^{31}P{^{1}H}-NMR** (162 MHz, CD_3CN, δ/ppm): -6.95. **HRMS** (ESI) m/z for $C_{11}H_{14}O_4P$ [M-H]$^-$: calcd. 241.0635, found 241.0636.

Geranyl-phenylphosphate (57)

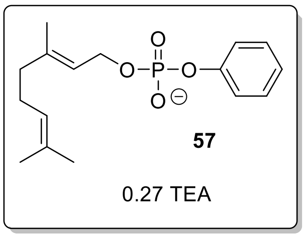

The compound was synthesized according to the general procedure C from geranylphosphate x 1.4 TBA (**SI-14**, 158 mg, 275 µmol) and 2-(trimethylsilyl)phenyl trifluoromethanesulfonate (**1**, 2.5 eq.). In this case the purification procedure required modification. After the reaction, the solvent was removed, and the residue was dissolved in TEAA-buffer (10 mM). Insolubles were removed and the solution was directly applied to RP-MPLC (see general procedure C). The product (**57**, 25.1 mg, 74.7 µmol, 27%) was isolated as colorless oil.

^{1}H-NMR (400 MHz, CD_3CN, δ/ppm): 7.30 – 7.24 (m, 2H), 7.21 – 7.16 (m, 2H), 7.07 – 7.01 (m, 1H), 5.32 (tq, *J* = 6.7, 1.3 Hz, 1H), 5.08 (tdt, *J* = 5.7, 2.9, 1.4 Hz, 1H), 4.41 (m, 2H), 2.96 (q, *J* = 7.3 Hz, 2H), 1.66 – 1.64 (m, 3H), 1.61 (d, *J* = 1.3 Hz, 3H), 1.59 – 1.57 (m, 3H), 1.19 (t, *J* = 7.3 Hz, 3H). **^{13}C{^{1}H}-NMR** (101 MHz, CD_3CN, δ/ppm): 154.32 (d, *J* = 5.3 Hz), 140.90, 132.47, 130.21, 124.94, 123.93, 121.87 (d, *J* = 6.7 Hz), 121.16 (d, *J* = 3.2 Hz), 63.48 (d, *J* = 3.6 Hz), 46.65, 40.14, 27.12, 25.82, 17.79, 16.53, 8.92. **^{31}P{^{1}H}-NMR** (162 MHz, CD_3CN, δ/ppm): -5.83. **HRMS** (ESI) m/z for $C_{16}H_{22}O_4P$ $[M-H]^-$: calcd. 309.1261, found 309.1266.

6-Hydroxyhexyl-phenylphosphate (58)

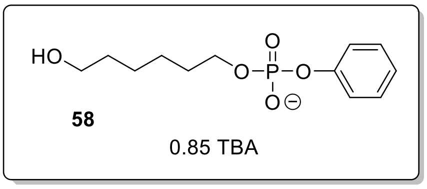

The compound was synthesized according to the general procedure C from 6-hydroxyhexylphosphate x 1.20 TBA (**SI-13**, 183 mg, 377 µmol) and 2-(trimethylsilyl)phenyl trifluoromethanesulfonate (**1**, 2.5 eq.). The product (**58**, 28.0 mg, 55.8 µmol, 15%) was isolated as colorless oil.

^{1}H-NMR (400 MHz, D_2O/MeCN-d3, δ/ppm): 7.58 – 7.51 (m, 2H), 7.38 – 7.31 (m, 3H), 4.09 (q, *J* = 6.5 Hz, 2H), 3.69 (t, *J* = 6.8 Hz, 2H), 3.34 – 3.26 (m, 7H), 1.83 – 1.71 (m, 9H), 1.65 (p, *J* = 6.8 Hz, 2H), 1.57 – 1.44 (m, 11H), 1.41 (t, *J* = 7.3 Hz, 1H), 1.11 (t, *J* = 7.4 Hz, 10H).

^{13}**C{^{1}H}-NMR** (101 MHz, D_2O/MeCN-d3, δ/ppm): 152.97 (d, *J* = 6.8 Hz), 130.54, 124.84, 120.99 (d, *J* = 4.6 Hz), 67.43 (d, *J* = 6.3 Hz), 62.52, 59.63 – 57.79 (m), 47.46, 32.23, 30.63 (d, *J* = 7.2 Hz), 25.63 (d, *J* = 8.8 Hz), 23.99, 21.10 – 19.15 (m), 13.74. ^{31}**P{^{1}H}-NMR** (162 MHz, D_2O/MeCN-d3, δ/ppm): -4.22. **HRMS** (ESI) m/z for $C_{12}H_{18}O_5P$ $[M-H]^-$: calcd. 273.0897, found 273.0899.

D4T-phenylphosphate (59)

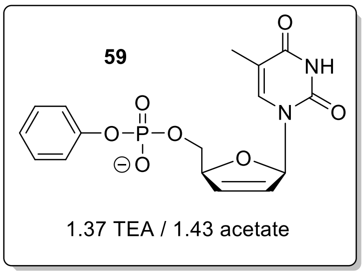

The compound was synthesized according to the general procedure C from d4T-monophosphate x 1.44 TBA (**SI-5**, 120 mg, 184 µmol) and 2-(trimethylsilyl)phenyl trifluoromethanesulfonate (**1**, 2.5 eq.). The product (**59**, 45.4 mg, 74.5 µmol, 41%) was isolated as colorless oil.

^{1}**H-NMR** (400 MHz, D_2O, δ/ppm): 7.33 (q, *J* = 1.2 Hz, 1H), 7.25 (dd, *J* = 8.4, 7.3 Hz, 2H), 7.12 – 7.02 (m, 3H), 6.90 (dt, *J* = 3.2, 1.7 Hz, 1H), 6.49 (dt, *J* = 6.2, 1.8 Hz, 1H), 5.90 (dt, *J* = 6.2, 2.0 Hz, 1H), 5.21 – 5.12 (m, 1H), 4.25 (dt, *J* = 11.6, 2.8 Hz, 1H), 4.09 (dt, *J* = 11.6, 3.6 Hz, 1H), 3.21 (q, *J* = 7.3 Hz, 11H), 1.60 (d, *J* = 1.1 Hz, 3H), 1.29 (t, *J* = 7.3 Hz, 16H). ^{13}**C{^{1}H}-NMR** (101 MHz, D_2O, δ/ppm): δ 166.43, 152.19, 151.42 (d, *J* = 7.2 Hz), 138.22, 134.22, 129.09, 125.04, 124.38, 120.21 (d, *J* = 4.4 Hz), 110.72, 89.95, 85.65 (d, *J* = 10.4 Hz), 66.19 (d, *J* = 5.5 Hz), 46.63, 11.28, 8.20. ^{31}**P{^{1}H}-NMR** (162 MHz, D_2O, δ/ppm): -4.85. **HRMS** (ESI) m/z for $C_{16}H_{16}N_2O_7P$ $[M-H]^-$: calcd. 379.0701, found 379.0706.

D4T-phenylpyrophosphate (60)

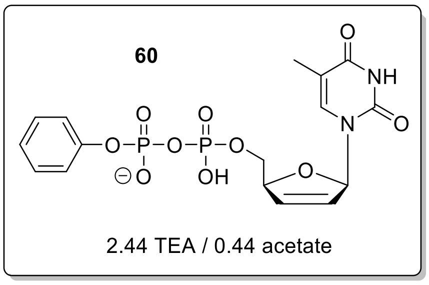

The compound was synthesized according to the general procedure C from d4T-monophosphate x 2.37 TBA (**SI-17**, 150 mg, 157 µmol) and 2-(trimethylsilyl)phenyl trifluoromethanesulfonate (**1**, 2.5 eq.). In this case the purification procedure required modification. After the reaction, the crude product was precipitated by the addition of Et_2O/pentene (1/1, 40 mL). The precipitate was separated by centrifugation, washed with Et_2O/pentane (1/1, 2 x 30 mL) and dried over high vac. The resulting crude product was dissolved in TEAA-buffer (10 mM) and the solution was directly applied to RP-MPLC (see general procedure C). The product (**60**, 38.8 mg, 52.1 µmol, 33%) was isolated as colorless oil.

^{1}H-NMR (400 MHz, D_2O, δ/ppm): 7.52 (q, *J* = 1.2 Hz, 1H), 7.35 – 7.26 (m, 2H), 7.18 – 7.09 (m, 3H), 6.97 – 6.91 (m, 1H), 6.45 (dt, *J* = 6.1, 1.8 Hz, 1H), 5.88 (ddd, *J* = 6.2, 2.4, 1.4 Hz, 1H), 5.13 – 5.05 (m, 1H), 4.22 – 4.10 (m, 2H), 3.19 (q, *J* = 7.3 Hz, 15H), 1.78 (dd, *J* = 1.2, 0.4 Hz, 3H), 1.27 (t, *J* = 7.3 Hz, 23H). **^{13}C{^{1}H}-NMR** (101 MHz, D_2O, δ/ppm): 166.42, 152.09, 151.71 (d, *J* = 7.2 Hz), 138.24, 134.06, 129.39, 125.34, 124.09 (d, *J* = 1.2 Hz), 120.19 (d, *J* = 4.8 Hz), 111.31, 89.71, 85.89 (d, *J* = 9.7 Hz), 66.53 (d, *J* = 5.9 Hz), 46.61, 11.41, 8.20. **^{31}P{^{1}H}-NMR** (162 MHz, D_2O, δ/ppm): -11.95 (d, *J* = 22.0 Hz), -16.41 (d, *J* = 22.0 Hz). **HRMS** (ESI) m/z for $C_{16}H_{17}N_2O_{10}P_2$ $[M-H]^-$: calcd. 459.0364, found 459.0365.

Diphenylpyrophosphate (61)

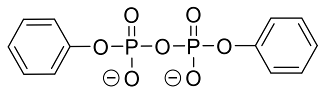

61 1.52 TBA / 0.33 TEA

The compound was synthesized according to the general procedure C from Phenylpyrophosphate x 2.4 TBA (**SI-16**, 215 mg, 260 µmol) and 2-(trimethylsilyl)phenyl trifluoromethanesulfonate (**1**, 2.5 eq.). In this case the purification procedure required modification. After the reaction, the solvent was removed and the residue was dissolved in TEAA-buffer (10 mM). Insolubles were removed and the solution was directly applied to RP-MPLC (see general procedure C). The product (**61**, 89.4 mg, 123 µmol, 47%) was isolated as colorless oil.

^{1}H-NMR (400 MHz, D_2O, δ/ppm): 7.42 – 7.34 (m, 4H), 7.24 – 7.17 (m, 6H), 3.25 – 3.14 (m, 14H), 1.72 – 1.59 (m, 12H), 1.36 (h, J = 7.4 Hz, 12H), 1.28 (t, J = 7.3 Hz, 3H), 0.95 (t, J = 7.4 Hz, 18H). **^{13}C{^{1}H}-NMR** (101 MHz, D_2O, δ/ppm): 151.68 (t, J = 3.7 Hz), 129.63, 124.35, 120.55 (t, J = 2.2 Hz), 58.09, 46.63, 23.10, 19.12, 12.79, 8.19. **^{31}P{^{1}H}-NMR** (162 MHz, D_2O, δ/ppm): -16.13. **HRMS** (ESI) m/z for $C_6H_{14}O_5P$ [M-H]$^-$: calcd. 197.0584, found 197.0584.

7. Cluster 3: Synthesis of Arylpolyphosphates (cyclophosphate reactions)

7.1. Preparation of cyclic metaphosphate (n = 4, 5, 7, 8) x TBA – salts:

Trimetaphosphate was commercially available. Tetrametaphosphate was synthesized as Na-salt on gram-scale according to Bell et al.[11]

A mixture of higher cyclic metaphosphates (n = 3 – 8) was prepared on multi-gram scale according to Glonek et al.[12] from crystalline orthophosphoric acid and DCC in TMU. Preparative separation was possible by automated SAX (Äkta-system, Q-Sepharose). Tri- to heptametaphosphate were eluted with Triethylammoniumbicarbonate-buffer (0.1 - 1M, pH 7.5). Octametaphosphate was eluted with NH_4HCO_3 – buffer. The sample qualities were polished during a second run of automated SAX (Äkta-system, Q-Sepharose, NH_4HCO_3 – buffer). The procedure delivered pentametaphosphate, heptametaphosphate and octametaphosphate as NH_4 – salts. Hexametaphosphate could not be isolated sufficient purity. The different ring-sizes were assigned by HRMS.

HRMS – data:

HRMS (ESI) m/z for $H_4O_{15}P_5$ [M-H]$^-$: calcd. 398.8244, found 398.8255.
HRMS (ESI) m/z for $H_6O_{21}P_7$ [M-H]$^-$: calcd. 558.7570, found 558.7587.
HRMS (ESI) m/z for $H_7O_{24}P_8$ [M-H]$^-$: calcd. 638.7234, found 638.7238.

For solubility reasons the cations had to be changed to TBA before subsequent reactions. This was achieved by using either DowexH$^+$ or Chelex®TBA$^+$ as described above. After lyophilization, the TBA-salts were dissolved in MeCN, passed through a syringe filter and evaporated to dryness. The isolated metaphosphate TBA – salts were isolated as white solids and could be stored for months in the fridge.

The metaphosphate / TBA – ratios were determined by the addition of tetramethylphosphonium bromide and qNMR measurements. The TBA-amounts were usually higher than expected according to phosphate units present. We hypothesize the surplus TBA-ions are part of hydroxide salts. Consequently, the following molecular weights were determined:

Trimetaphosphate (**68**, DowexH$^+$): 3MP x 3.9 TBA (MW = 1194 g/mol)

Tetrametaphosphate (**69**, Chelex®TBA$^{+)}$: 4MP x 6.0 TBA (MW = 1803 g/mol)

Pentametaphosphate (**70**, Chelex®TBA^{+}): 5MP x 5.3 TBA (MW = 1683 g/mol)

Heptametaphosphate (**71,** Chelex®TBA^{+}): 7MP x 8.2 TBA (MW = 2559 g/mol)

Octametaphosphate (**72**, Chelex®TBA^{+}): 8MP x 11.3 TBA. (MW = 3424 g/mol)

7.2. Synthesis of arylpolyphosphates

General procedure D for the synthesis of arylpolyphosphates:

The cyclophosphate x TBA salt (100 µmol) was dissolved in dry MeCN (ca. 70 mM) and the corresponding aryne-precursor (4.0 – 5.0 eq.) was added. Subsequently, TBAF (1 M in THF, 5.0 eq.) was added dropwise via syringe pump (1 h, rt, needle tip is below solvent surface). The reaction mixture was stirred for additional 15 min at rt before the amine-nucleophile (2.5 – 20 eq.) was added. The resulting solution was stirred 24 - 48 h, and the crude product was precipitated by pipetting the reaction mixture into a $NaClO_4$-solution (0.5 M in acetone, -20°C, 35 mL). The suspension was incubated for 20 min at -20°C and the precipitate was separated by centrifugation. The resulting pellet was washed with acetone (30 mL) and dried over high vacuum.

Purification method D1:

The crude product was purified by automated SAX (Äkta system, Q-Sepharose, $NaClO_4$-buffer). Product containing fractions (80 – 150 mM) were combined and lyophilized. The resulting solid was washed with acetone (3 x 30 mL), separated by centrifugation and dried over high vacuum. The products were isolated as Na – salts.

Purification method D2:

The crude product was purified by automated RP-MPLC (Interchim system, C18-AQ, H_2O/MeCN/TEAA [10 mM]). The product containing fractions were combined and lyophilized. The products were isolated as TEA – salts.

PhenylP$_3$-propargylamidate (78)

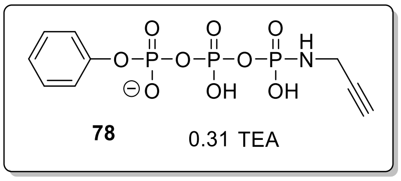

The compound was synthesized according to the general procedure D from trimetaphosphate x 3.9 TBA (**68**, 119 mg, 100 µmol) and 2-(Trimethylsilyl)phenyl-trifluoro-methanesulfonate (**1**, 121 µL, 149 mg, 500 µmol, 5.0 eq.). Propargylamine (16.0 µL, 13.8 mg, 250 µmol, 2.5 eq.) was applied for nucleophilic ring-opening (24 h). The crude product was purified according to purification method D2. The product (**78**, 32.6 mg, 68.7 µmol, 69%) was isolated as colorless oil.

^{1}H-NMR (400 MHz, D_2O, δ/ppm): 7.45 – 7.39 (m, 2H), 7.33 – 7.27 (m, 2H), 7.25 – 7.19 (m, 1H), 3.66 (dd, *J* = 9.9, 2.5 Hz, 2H), 3.21 (q, *J* = 7.3 Hz, 2H), 2.55 (t, *J* = 2.5 Hz, 1H), 1.29 (t, *J* = 7.4 Hz, 3H). **^{13}C{^{1}H}-NMR** (101 MHz, D_2O, δ/ppm): 151.75 (d, *J* = 7.2 Hz), 129.68, 124.33 (d, *J* = 1.4 Hz), 120.59 (d, *J* = 4.6 Hz), 82.96 (d, *J* = 11.7 Hz), 71.35, 46.64, 30.96, 8.20. **^{31}P{^{1}H}-NMR** (162 MHz, D_2O, δ/ppm): -2.83 (d, *J* = 20.6 Hz), -15.91 (d, *J* = 19.8 Hz), -23.02 (t, *J* = 20.1 Hz). **HRMS** (ESI) m/z for $C_9H_{11}NO_9P_3$ [M-H]$^-$: calcd. 369.9652, found 369.9652.

PhenylP$_3$-amidate (79)

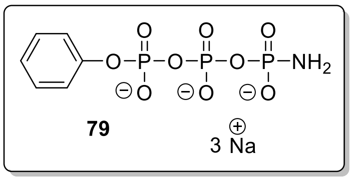

The compound was synthesized according to the general procedure D from trimetaphosphate x 3.9 TBA (**68**, 119 mg, 100 µmol) and 2-(Trimethylsilyl)phenyl-trifluoro-methanesulfonate (**1**, 121 µL, 149 mg, 500 µmol, 5.0 eq.). Aqu. NH_3 (25%, 17.0 µL, 250 µmol, 2.5 eq.) was applied for nucleophilic ring-opening (24 h). The crude product was purified according to purification method D1. The product (**79**, 26.7 mg, 66.9 µmol, 67%) was isolated as white solid.

^{1}H-NMR (400 MHz, D_2O, δ/ppm): 7.47 – 7.40 (m, 2H), 7.33 – 7.28 (m, 2H), 7.27 – 7.21 (m, 1H). **^{13}C{^{1}H}-NMR** (101 MHz, D_2O, δ/ppm): 151.70 (d, *J* = 7.2 Hz), 129.72, 124.44 (d, *J* = 1.5 Hz), 120.61 (d, *J* = 4.5 Hz). **^{31}P{^{1}H}-NMR** (162 MHz, D_2O, δ/ppm): -1.03 (d, *J* = 19.1 Hz), -

15.76 (d, *J* = 19.9 Hz), -22.68 (t, *J* = 19.4 Hz). **HRMS** (ESI) m/z for $C_6H_9NO_9NP_3$ $[M-H]^-$: calcd. 331.9496, found 331.9493.

PhenylP₃-anthracen-9-ylmethanamidate (80)

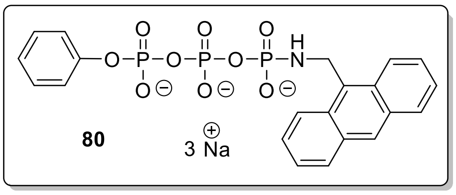

The compound was synthesized according to the general procedure D from trimetaphosphate x 3.9 TBA (**68**, 119 mg, 100 µmol) and 2-(Trimethylsilyl)phenyl-trifluoro-methanesulfonate (**1**, 121 µL, 149 mg, 500 µmol, 5.0 eq.). Anthracen-9-ylmethanamine (51.3 mg, 250 µmol, 2.5 eq.) was applied for nucleophilic ring-opening (24 h). The crude product was purified according to purification method D1. The product (**80**, 27.1 mg, 46.0 µmol, 46%) was isolated as white solid.

^{1}H-NMR (400 MHz, D_2O, δ/ppm): 8.56 (s, 1H), 8.51 (dd, *J* = 8.9, 1.0 Hz, 2H), 8.11 (ddd, *J* = 8.4, 1.6, 0.8 Hz, 2H), 7.63 (ddd, *J* = 8.9, 6.5, 1.5 Hz, 2H), 7.57 (ddd, *J* = 7.8, 6.6, 1.1 Hz, 2H), 7.29 – 7.22 (m, 2H), 7.20 – 7.13 (m, 2H), 6.96 – 6.89 (m, 1H), 4.98 (s, 2H). **^{13}C{^{1}H}-NMR** (101 MHz, D_2O, δ/ppm): 151.76 (d, *J* = 7.2 Hz), 131.27 (d, *J* = 12.7 Hz), 131.27, 129.61, 129.49, 128.80, 127.28, 126.57, 125.41, 124.47, 123.99, 120.35 (d, *J* = 4.7 Hz), 37.82. **^{31}P{^{1}H}-NMR** (162 MHz, D_2O, δ/ppm): -2.15 (d, *J* = 21.2 Hz), -15.91 (d, *J* = 19.4 Hz), -22.67 (t, *J* = 21.2 Hz). **HRMS** (ESI) m/z for $C_{21}H_{18}NO_9P_3$ $[M-H_2]^{2-}$: calcd. 260.5102, found 260.5102.

PhenylP₃-5’-deoxyadenosyl-5’-amidate (81)

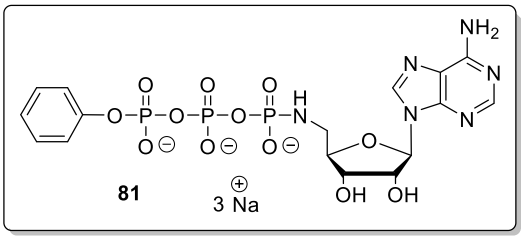

The compound was synthesized according to the general procedure D from trimetaphosphate x 3.9 TBA (**68**, 119 mg, 100 µmol) and 2-(Trimethylsilyl)phenyl-trifluoro-methanesulfonate (**1**, 121 µL, 149 mg, 500 µmol, 5.0 eq.). Nucleophilic ring-opening was induced by dropping the reaction mixture into a solution of 5’-Deoxy-5’-aminoadenosine (**SI-1**, 66.5 mg, 250 µmol, 2.5 eq.) and DBU (74.5 µL, 76.0 mg, 500 µmol, 5.0 eq.) in DMF

(1.5 mL). The resulting solution was stirred for 48 h. The crude product was purified according to purification method D1. The product (**81**, 34.5 mg, 53.2 µmol, 53%) was isolated as white solid.

^{1}H-NMR (400 MHz, D_2O, δ/ppm): 8.33 (s, 1H), 8.23 (d, *J* = 0.5 Hz, 1H), 7.24 – 7.17 (m, 2H), 7.12 (dddd, *J* = 7.9, 1.9, 1.3, 0.5 Hz, 2H), 7.00 (dp, *J* = 7.0, 0.8 Hz, 1H), 5.98 (d, *J* = 6.4 Hz, 1H), 4.78 – 4.75 (m, 1H), 4.41 (dd, *J* = 5.4, 3.3 Hz, 1H), 4.22 (q, *J* = 3.4 Hz, 1H), 3.34 – 3.17 (m, 2H). **^{13}C{^{1}H}-NMR** (101 MHz, D_2O, δ/ppm): 155.51, 152.79, 151.51 (d, *J* = 7.1 Hz), 148.87, 140.21, 129.32, 123.98, 120.27 (d, *J* = 4.6 Hz), 118.90, 87.11, 85.44 (d, *J* = 8.9 Hz), 73.27, 70.79, 43.14. **^{31}P{^{1}H}-NMR** (162 MHz, D_2O, δ/ppm): -1.58 (d, *J* = 21.0 Hz), -15.79 (d, *J* = 19.0 Hz), -22.78 (t, *J* = 19.2 Hz). **HRMS** (ESI) m/z for $C_{16}H_{20}N_6O_{12}P_3$ [M-H]$^-$: calcd. 581.0358, found 581.0361.

PhenylP$_3$-5'-deoxyguanosyl-5'-amidate (82)

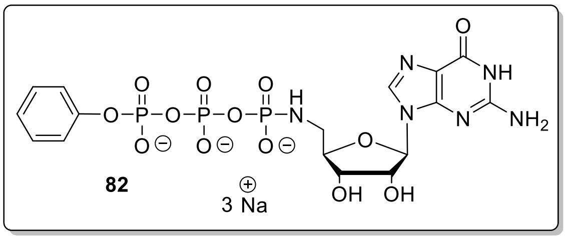

The compound was synthesized according to the general procedure D from trimetaphosphate x 3.9 TBA (**68**, 119 mg, 100 µmol) and 2-(Trimethylsilyl)phenyl-trifluoro-methanesulfonate (**1**, 121 µL, 149 mg, 500 µmol, 5.0 eq.). Nucleophilic ring-opening was induced by dropping the reaction mixture into a solution of 5'-Deoxy-5'-aminoguanosine (**SI-2**, 56.4 mg, 200 µmol, 2.0 eq.) in DMSO/DMF (1/1, 1.5 mL) and stirring the resulting solution for 48 h. The crude product was purified according to purification method D1. The product (**82**, 43.9 mg, 66.1 µmol, 66%) was isolated as white solid.

^{1}H-NMR (400 MHz, D_2O, δ/ppm): 7.83 (s, 1H), 7.17 – 7.02 (m, 4H), 6.91 (ddt, *J* = 7.3, 6.3, 1.3 Hz, 1H), 5.73 (d, *J* = 8.1 Hz, 1H), 5.17 (dd, *J* = 8.0, 5.5 Hz, 1H), 4.45 (dd, *J* = 5.5, 1.4 Hz, 1H), 4.33 – 4.28 (m, 1H), 3.38 (ddd, *J* = 14.1, 5.0, 3.0 Hz, 1H), 3.25 (ddd, *J* = 14.1, 9.3, 2.5 Hz, 1H). **^{13}C{^{1}H}-NMR** (101 MHz, D_2O, δ/ppm): 159.37, 153.76, 151.50 (d, *J* = 7.0 Hz), 151.26, 139.68, 129.07, 123.68, 120.16 (d, *J* = 4.5 Hz), 117.25, 88.70, 86.60 (d, *J* = 9.8 Hz), 71.47, 71.07, 43.36. **^{31}P{^{1}H}-NMR** (162 MHz, D_2O, δ/ppm): -1.59 (d, *J* = 22.9 Hz), -15.76 (d, *J* = 19.2 Hz), -22.87 (dd, *J* = 22.6, 19.5 Hz). **HRMS** (ESI) m/z for $C_{16}H_{20}N_6O_{13}P_3$ [M-H]$^-$: calcd. 597.0307, found 597.0309.

Napht-2-ylP3-5'-deoxyguanosyl-5'-amidate (83)

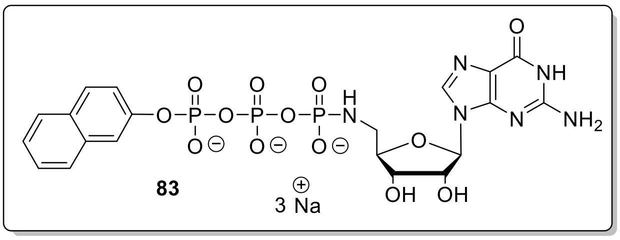

The compound was synthesized according to the general procedure D from trimetaphosphate x 3.9 TBA (**68**, 119 mg, 100 µmol) and 3-(Trimethylsilyl)naphthalen-2-yl trifluoromethanesulfonate (**SI-12**, 174 mg, 500 µmol, 5.0 eq.). Nucleophilic ring-opening was induced by dropping the reaction mixture into a solution of 5'-Deoxy-5'-aminoguanosine (**SI-2**, 56.4 mg, 200 µmol, 2.0 eq.) in DMSO/DMF (1/1, 1.5 mL) and stirring the resulting solution for 48 h. The crude product was purified according to purification method D2 and D1 subsequently. The product (**83**, 34.9 mg, 48.9 µmol, 49%) was isolated as white solid.

^{1}H-NMR (400 MHz, D_2O, δ/ppm): 7.69 (d, J = 9.1 Hz, 1H), 7.67 (s, 1H), 7.65 – 7.57 (m, 2H), 7.55 (d, J = 2.1 Hz, 1H), 7.34 – 7.30 (m, 1H), 7.29 – 7.22 (m, 2H), 5.64 (d, J = 8.0 Hz, 1H), 5.02 (dd, J = 7.9, 5.5 Hz, 1H), 4.40 (dd, J = 5.5, 1.5 Hz, 1H), 4.33 (t, J = 2.3 Hz, 1H), 3.48 (ddd, J = 14.0, 4.1, 2.9 Hz, 1H), 3.29 (ddd, J = 14.0, 9.1, 2.4 Hz, 1H). **^{13}C{^{1}H}-NMR** (101 MHz, D_2O, δ/ppm): 158.22, 152.77, 150.69, 149.24 (d, J = 7.6 Hz), 139.30, 133.25, 129.81, 129.03, 127.10 (d, J = 3.3 Hz), 125.95, 124.87, 120.85, 120.81, 116.71, 116.37 (d, J = 4.8 Hz), 88.80, 86.52 (d, J = 10.5 Hz), 71.43, 71.07, 43.43. **^{31}P{^{1}H}-NMR** (162 MHz, D_2O, δ/ppm): -1.35 (d, J = 23.0 Hz), -15.81 (d, J = 20.1 Hz), -22.43 (dd, J = 23.0, 20.0 Hz). **HRMS** (ESI) m/z for $C_{20}H_{21}N_6NaO_{13}P_3$ [M-H]$^-$: calcd. 669.0283, found 669.0285.

Pyren-2-ylP3-5'-deoxyadenosyl-5'-amidate (84)

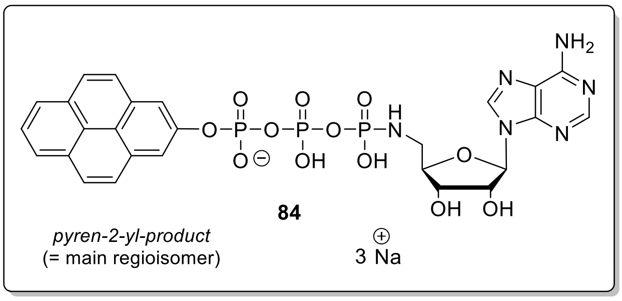

The compound was synthesized according to the general procedure D from trimetaphosphate x 3.9 TBA (**68**, 119 mg, 100 µmol) and 1-(Trimethylsilyl)pyren-2-yl trifluoromethanesulfonate (**SI-19**, 212 mg, 500 µmol, 5.0 eq.). Nucleophilic ring-opening was induced by dropping the reaction mixture into a solution of 5'-Deoxy-5'-aminoadenosine (**SI-1**, 66.5 mg, 250 µmol, 2.5 eq.) and DBU (74.5 µL, 76.0 mg, 500 µmol, 5.0 eq.) in DMF (1.5 mL). The resulting solution was stirred for 48 h. The crude product was purified according to purification method D1. The product (**84**, 31.9 mg, 41.1 µmol, 41%) was isolated as white solid.

The product is formed in an 88:12 (2-**84**:1-**84**) regioisomeric ratio. ^{1}H- and ^{13}C{^{1}H}-NMR signals from the major product are given.

^{1}H-NMR (400 MHz, D_2O, δ/ppm): 8.10 (d, *J* = 7.7 Hz, 2H), 7.98 – 7.89 (m, 7H), 7.51 (s, 1H), 7.42 (s, 1H), 5.33 (d, *J* = 5.0 Hz, 1H), 4.16 – 4.05 (m, 3H), 3.32 (ddd, *J* = 14.0, 7.0, 4.0 z, 1H), 3.23 (ddd, *J* = 14.1, 9.5, 4.5 Hz, 1H). **^{13}C{^{1}H}-NMR** (101 MHz, D_2O, δ/ppm): 153.85, 151.27, 149.50 (d, *J* = 6.8 Hz), 146.99, 138.55, 131.74, 129.95, 127.74, 126.72, 125.65, 125.21, 123.16, 120.48, 117.41, 116.28 (d, *J* = 4.9 Hz), 87.03, 84.38 (d, *J* = 9.7 Hz), 73.90, 70.36, 43.29. **^{31}P{^{1}H}-NMR** (162 MHz, D_2O, δ/ppm): -1.62 (d, *J* = 22.0 Hz), -15.82 (d, *J* = 19.9 Hz), -22.75 (dd, *J* = 22.0, 19.2 Hz). **HRMS** (ESI) m/z for $C_{26}H_{24}N_6O_{12}P_3$ $[M-H]^-$: calcd. 705.0671, found 705.0677.

Benzo[1,3]dioxol-5-ylP$_4$-((7-(diethylamino)-2-oxo-2H-chromen-4-yl)methylamidate (85)

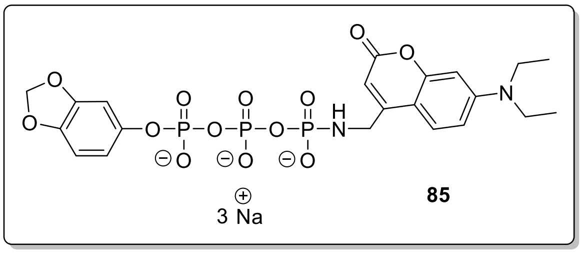

The compound was synthesized according to the general procedure D from trimetaphosphate x 3.9 TBA (**68**, 119 mg, 100 µmol) and 6-(Trimethylsilyl)benzo[1,3]dioxol-5-yl trifluoromethanesulfonate (**SI-9**, 171 mg, 500 µmol, 5.0 eq.). Amino-DEACM (**SI-26**, 61.3 mg, 250 µmol, 2.5 eq.) was applied for nucleophilic ring-opening (24 h). The crude product was purified according to purification method D1. The product (**85**, 39.7 mg, 59.1 µmol, 59%) was isolated as white solid.

^{1}H-NMR (400 MHz, D_2O, δ/ppm): 7.35 (d, *J* = 9.1 Hz, 1H), 6.69 (dd, *J* = 9.2, 2.5 Hz, 1H), 6.67 (ddd, *J* = 2.4, 1.0, 0.5 Hz, 1H), 6.64 – 6.60 (m, 1H), 6.58 – 6.54 (m, 2H), 6.26 – 6.23 (m, 1H), 5.75 (s, 2H), 4.18 (dd, *J* = 8.6, 1.5 Hz, 2H), 3.44 (q, *J* = 7.1 Hz, 4H), 1.19 (t, *J* = 7.1 Hz, 6H). **^{13}C{^{1}H}-NMR** (101 MHz, D_2O, δ/ppm): 166.19, 158.12 (d, *J* = 9.7 Hz), 155.16, 151.02, 146.98, 146.27 (d, *J* = 7.6 Hz), 143.09, 125.03, 112.67 (d, *J* = 4.9 Hz), 109.95, 107.82, 106.89, 103.25, 102.57 (d, *J* = 4.7 Hz), 101.36, 96.95, 44.43, 41.71, 11.55. **^{31}P{^{1}H}-NMR** (162 MHz, D_2O, δ/ppm): -2.28 (d, *J* = 21.2 Hz), -15.87 (d, *J* = 20.0 Hz), -22.92 (t, *J* = 20.6 Hz). **HRMS** (ESI) m/z for $C_{21}H_{24}N_2O_{13}P_3$ [M-H]$^-$: calcd. 605.0497, found 605.0500.

Synthesis of PhenylP$_4$-propargylamidate (86)

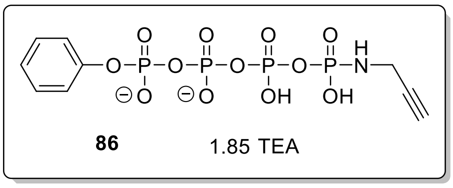

The compound was synthesized according to the general procedure D from tetrametaphosphate x 6.0 TBA (**69**, 180 mg, 100 µmol) and 2-(Trimethylsilyl)phenyl-trifluoromethanesulfonate (**1**, 121 µL, 149 mg, 500 µmol, 5.0 eq.). Propargylamine (16.0 µL, 13.8 mg, 250 µmol, 2.5 eq.) was applied for nucleophilic ring-opening (24 h). The crude product was purified according to purification method D2. The product (**86**, 35.1 mg, 40.9 µmol, 41%) was isolated as colorless oil.

^{1}H-NMR (400 MHz, D_2O, δ/ppm): 7.48 – 7.38 (m, 2H), 7.30 (dq, *J* = 7.7, 1.2 Hz, 2H), 7.26 – 7.19 (m, 1H), 3.70 (dd, *J* = 10.2, 2.5 Hz, 2H), 3.21 (q, *J* = 7.3 Hz, 11H), 2.56 (t, *J* = 2.5 Hz, 1H), 1.29 (t, *J* = 7.3 Hz, 16H). **^{13}C{^{1}H}-NMR** (101 MHz, D_2O, δ/ppm): 151.73 (d, *J* = 7.1 Hz), 129.71, 124.36, 120.65 (d, *J* = 4.6 Hz), 83.11 (d, *J* = 11.4 Hz), 71.32, 46.64, 30.99, 8.21. **^{31}P{^{1}H}-NMR** (162 MHz, D_2O, δ/ppm): -2.52 (d, *J* = 19.4 Hz, 1P), -15.70 (d, *J* = 18.0 Hz, 1P), -22.45 – -23.22 (m, 2P). **HRMS** (ESI) m/z for $C_9H_{11}NO_{12}P_4$ $[M-H_2]^{2-}$: calcd. 224.4621, found 224.4623.

Pyren-2-ylP4-5'-deoxyguanosyl-5'-amidate (87)

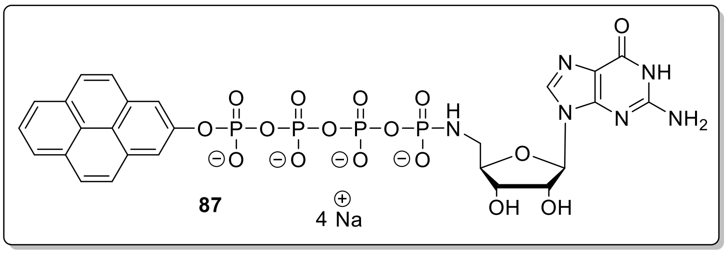

The compound was synthesized according to the general procedure D from tetrametaphosphate x 6.0 TBA (**69**, 180 mg, 100 µmol) and 1-(Trimethylsilyl)pyren-2-yl trifluoromethanesulfonate (**SI-19**, 212 mg, 500 µmol, 5.0 eq.). Nucleophilic ring-opening was induced by dropping the reaction mixture into a solution of 5'-Deoxy-5'-aminoguanosine (**SI-2**, 56.4 mg, 200 µmol, 2.0 eq.) in DMSO/DMF (1/1, 1.5 mL) and stirring the resulting solution for 48 h. The crude product was purified according to purification method D1. The product (**87**, 40.5 mg, 45.1 µmol, 45%) was isolated white solid.

The product is formed in an 88:12 (2-**87**:1:**87**) regioisomeric ratio. ^{1}H- and ^{13}C{^{1}H}-NMR signals from the major product are given.

^{1}H-NMR (400 MHz, D_2O, δ/ppm): 8.10 – 7.92 (m, 8H), 7.90 – 7.83 (m, 1H), 7.20 (s, 1H), 5.21 (d, *J* = 7.6 Hz, 1H), 4.55 (dd, *J* = 7.7, 5.4 Hz, 1H), 4.17 (dd, *J* = 5.5, 1.7 Hz, 1H), 4.12 (q, *J* = 2.5 Hz, 1H), 3.33 (dt, *J* = 14.0, 3.7 Hz, 1H), 3.15 (ddd, *J* = 14.0, 9.3, 2.5 Hz, 1H). **^{13}C{^{1}H}-NMR** (101 MHz, D_2O, δ/ppm): 157.23, 151.96, 149.79, 149.67 (d, *J* = 7.4 Hz), 138.26, 131.98, 130.02, 127.95, 126.88, 125.63, 125.13, 123.25, 120.75, 116.61 (d, *J* = 4.6 Hz), 115.70, 88.34, 85.98 (d, *J* = 10.4 Hz), 71.49, 71.23, 43.01. **^{31}P{^{1}H}-NMR** (162 MHz, D_2O, δ/ppm): -1.08 (d, *J* = 22.3 Hz), -15.45 (d, *J* = 18.0 Hz), -21.99 (dd, *J* = 22.2, 13.9 Hz), -22.59 (dd, *J* = 17.8, 13.8 Hz). **HRMS** (ESI) m/z for $C_{26}H_{23}N_6O_{16}P_4$ $[M-H_3]^{3-}$: calcd. 266.3379, found 266.3379.

Synthesis of PhenylP$_4$-5'-deoxyguanosyl-5'-amidate (88)

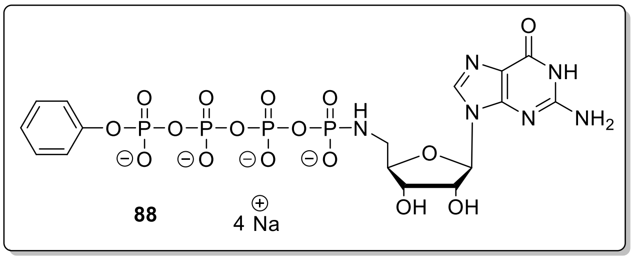

The compound was synthesized according to the general procedure D from tetrametaphosphate x 6.0 TBA (**69**, 180 mg, 100 µmol) and 2-(Trimethylsilyl)phenyl-trifluoromethanesulfonate (**1**, 121 µL, 149 mg, 500 µmol, 5.0 eq.). Nucleophilic ring-opening was induced by dropping the reaction mixture into a solution of 5'-Deoxy-5'-aminoguanosine (**SI-2**, 56.4 mg, 200 µmol, 2.0 eq.) in DMSO/DMF (1/1, 1.5 mL) and stirring the resulting solution for 48 h. The crude product was purified according to purification method D2 and D1 subsequently. The product (**88**, 32.6 mg, 42.5 µmol, 43%) was isolated as white solid.

^{1}H-NMR (400 MHz, D_2O, δ/ppm): 7.67 (s, 1H), 7.10 – 6.94 (m, 4H), 6.80 – 6.69 (m, 1H), 5.57 (d, *J* = 8.0 Hz, 1H), 4.97 (dd, *J* = 7.9, 5.5 Hz, 1H), 4.32 (dd, *J* = 5.5, 1.5 Hz, 1H), 4.17 – 4.09 (m, 1H), 3.23 – 3.04 (m, 2H). **^{13}C{^{1}H}-NMR** (101 MHz, D_2O, δ/ppm): 158.96, 153.47, 151.51 (d, *J* = 7.2 Hz), 151.27, 139.68, 129.29, 123.83, 120.36 (d, *J* = 4.3 Hz), 117.15, 88.68, 86.60 (d, *J* = 9.7 Hz), 71.40, 71.23, 43.17. **^{31}P{^{1}H}-NMR** (162 MHz, D_2O, δ/ppm): -1.22 (d, *J* = 21.1 Hz), -15.50 (d, *J* = 17.5 Hz), -22.29 (dd, *J* = 21.4, 15.2 Hz), -22.90 (dd, *J* = 17.9, 15.1 Hz). **HRMS** (ESI) m/z for $C_{16}H_{19}N_6O_{16}P_4$ [M-H_3]$^{3-}$: calcd. 224.9941, found 224.9942.

3,4-DimethylphenylP$_4$-(7-(diethylamino)-2-oxo-2H-chromen-4-yl)methylamidate (89)

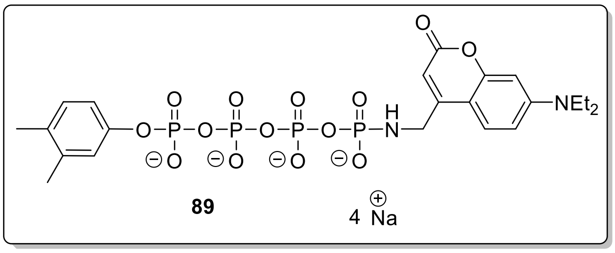

The compound was synthesized according to the general procedure D from tetrametaphosphate x 6.0 TBA (**69**, 180 mg, 100 µmol) and 4,5-Dimethyl-2-(trimethylsilyl)phenyl-trifluoromethanesulfonate (**SI-6**, 163 mg, 500 µmol, 5.0 eq.). Amino-DEACM (**SI-26**, 61.3 mg, 250 µmol, 2.5 eq.) was applied for nucleophilic ring-opening (24 h). The crude product was purified according to purification method D1. The product (**89**, 16.6 mg, 22.0 µmol, 22%) was isolated as white solid.

^{1}H-NMR (400 MHz, D_2O, δ/ppm): 7.34 (d, *J* = 9.1 Hz, 1H), 6.68 (d, *J* = 1.4 Hz, 2H), 6.66 (dd, *J* = 9.1, 2.6 Hz, 1H), 6.61 (s, 1H), 6.48 (d, *J* = 2.5 Hz, 1H), 6.05 (d, *J* = 1.2 Hz, 1H), 4.11 (d, *J* = 8.9 Hz, 2H), 3.37 (q, *J* = 7.0 Hz, 4H), 1.85 (s, 3H), 1.82 (s, 3H), 1.12 (t, *J* = 7.1 Hz, 6H). **^{13}C{^{1}H}-NMR** (101 MHz, D_2O, δ/ppm): 166.32, 158.76 (d, *J* = 8.5 Hz), 155.03, 151.04, 149.33, 137.94, 132.15, 129.87, 125.31, 120.77 (d, *J* = 5.1 Hz), 116.92 (d, *J* = 4.2 Hz), 110.15, 107.07, 103.17, 96.95, 44.44, 41.79, 18.68, 17.87, 11.61. **^{31}P{^{1}H}-NMR** (162 MHz, D_2O, δ/ppm): -2.06 (d, *J* = 21.8 Hz, 1P), -15.66 (d, *J* = 17.8 Hz, 1P), -22.06 – -23.01 (m, 2P). **HRMS** (ESI) m/z for $C_{22}H_{29}N_2O_{14}P_4$ [M-H]$^-$: calcd. 669.0575, found 669.0579.

Synthesis of PhenylP_5-propargylamidate (90)

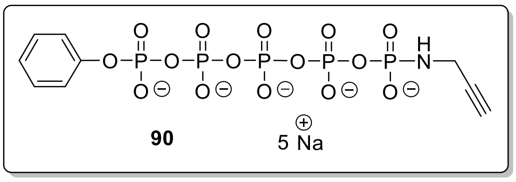

The compound was synthesized according to the general procedure D from pentametaphosphate x 5.3 TBA (**70**, 168 mg, 100 µmol) and 2-(Trimethylsilyl)phenyl-trifluoromethanesulfonate (**1**, 121 µL, 149 mg, 500 µmol, 5.0 eq.). Propargylamine (64.0 µL, 55.0 mg, 1.00 mmol, 10 eq.) was applied for nucleophilic ring-opening (48 h). The crude product was purified according to purification method D1. The product (**90**, 15.2 mg, 23.1 µmol, 23%) was isolated as white solid.

^{1}H-NMR (400 MHz, D_2O, δ/ppm): 7.47 – 7.39 (m, 2H), 7.34 – 7.27 (m, 2H), 7.26 – 7.20 (m, 1H), 3.73 (dd, *J* = 10.7, 2.4 Hz, 2H), 2.57 (td, *J* = 2.5, 0.5 Hz, 1H). **^{13}C{^{1}H}-NMR** (101 MHz, D_2O, δ/ppm): 151.70 (d, *J* = 7.2 Hz), 129.71, 124.39 (d, *J* = 1.4 Hz), 120.68 (d, *J* = 4.4 Hz), 68.14, 30.95. **^{31}P{^{1}H}-NMR** (162 MHz, D_2O, δ/ppm): -2.34 (d, *J* = 19.7 Hz, 1P), -15.55 (d, *J* = 16.9 Hz, 1P), -21.84 – -23.03 (m, 3P). **HRMS** (ESI) m/z for $C_9H_{13}NO_{15}P_5$ [M-H]$^-$: calcd. 529.8979, found 529.8980.

Synthesis of PhenylP$_7$-propargylamidate (91)

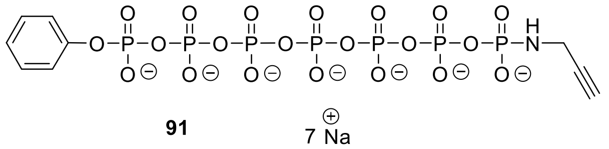

The compound was synthesized according to the general procedure D from heptametaphosphate x 8.2 TBA (**71**, 255 mg, 100 µmol) and 2-(Trimethylsilyl)phenyl-trifluoromethanesulfonate (**1**, 96.9 µL, 119 mg, 400 µmol, 4.0 eq.). Propargylamine (64.0 µL, 55.0 mg, 1.0 mmol, 10 eq.) was applied for nucleophilic ring-opening (48 h). The crude product was purified according to purification method D1. The product (**91**, 30.9 mg, 36.6 µmol, 37%) was isolated as white solid.

^{1}H-NMR (400 MHz, D_2O, δ/ppm): 7.51 – 7.40 (m, 2H), 7.36 – 7.31 (m, 2H), 7.30 – 7.24 (m, 1H), 3.76 (dd, *J* = 10.8, 2.5 Hz, 2H), 2.64 (t, *J* = 2.5 Hz, 1H). **^{13}C{^{1}H}-NMR** (101 MHz, D_2O, δ/ppm): 151.66 (d, *J* = 7.3 Hz), 129.80, 124.56, 120.67 (d, *J* = 4.5 Hz), 83.09 (d, *J* = 10.6 Hz), 71.49, 30.30. **^{31}P{^{1}H}-NMR** (162 MHz, D_2O, δ/ppm): -1.83 – -2.25 (m, 1P), -15.33 (d, *J* = 17.7 Hz, 1P), -21.62 – -22.03 (m, 4P), -22.20 (dd, *J* = 17.7, 13.7 Hz, 1P). **HRMS** (ESI) m/z for $C_9H_{15}NO_{21}P_7$ $[M-H]^-$: calcd. 689.8305, found 689.8306.

Synthesis of PhenylP$_8$-propargylamidate (92)

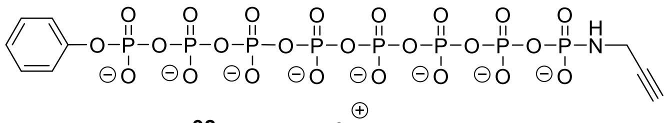

The compound was synthesized according to the general procedure D from octametaphosphate x 11.3 TBA (**72**, 342 mg, 100 µmol) and 2-(Trimethylsilyl)phenyl-trifluoromethanesulfonate (**1**, 96.9 µL, 119 mg, 400 µmol, 4.0 eq.). Propargylamine (64.0 µL, 55.0 mg, 1.0 mmol, 10 eq.) was applied for nucleophilic ring-opening (48 h). The crude product was purified according to purification method D1. The product (**92**, 21.2 mg, 22.4 µmol, 22%) was isolated as white solid.

^{1}H-NMR (400 MHz, D_2O, δ/ppm): 7.47 – 7.39 (m, 2H), 7.30 (dq, *J* = 7.8, 1.2 Hz, 2H), 7.26 – 7.20 (m, 1H), 3.73 (dd, *J* = 10.7, 2.5 Hz, 2H), 2.60 (t, *J* = 2.5 Hz, 1H). **^{13}C{^{1}H}-NMR** (101 MHz, D_2O, δ/ppm): 151.65 (d, *J* = 7.3 Hz), 129.73, 124.45, 120.65 (d, *J* = 4.6 Hz), 71.38, 30.93. **^{31}P{^{1}H}-NMR** (162 MHz, D_2O, δ/ppm): -2.24 (d, *J* = 20.1 Hz, 1P), -15.49 (d, *J* = 17.8 Hz, 1P), -21.71 – -22.25 (m, 5P), -22.33 – -22.62 (m, 1P). **HRMS** (ESI) m/z for $C_9H_{15}NO_{24}P_8$ $[M-H_2]^{2-}$: calcd. 384.3948, found 384.3946.

Phenyl-cyclotriphosphate as storable triphosphorylation reagent (73)

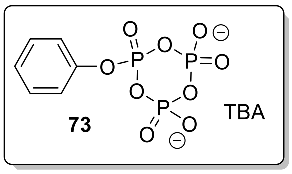

The cyclophosphate x TBA salt (**68**, 100 µmol) was dissolved in dry MeCN (ca. 70 mM) and 2-(Trimethylsilyl)phenyl-trifluoro-methanesulfonate (**1**, 121 µL, 149 mg, 500 µmol, 5.0 eq.) (4.0 – 5.0 eq.) was added. Subsequently TBAF (1 M in THF, 5.0 eq.) was added dropwise via syringe pump (1 h, rt, needle tip is below solvent surface). Then, the reaction mixture is stirred for 15 min at rt. The product is precipitated with Et_2O (40 mL) and the resulting oil is washed with Et_2O (2 x 30 mL). The phenyl-cyclotriphosphate **73** is dried over high vacuum and can be stored as triphosphorylation reagent in the fridge for weeks without substantial decomposition. Furthermore, storage as solution in MeCN at rt is possible for weeks.

^{31}P{^{1}H}-NMR (162 MHz, CD_3CN, δ/ppm): -23.61 (d, *J* = 25.1 Hz), -26.19 (t, *J* = 24.4 Hz). **HRMS** (ESI) m/z for $C_6H_5O_9P_3$ $[M-H_2]^{2-}$: calcd. 156.9578, found 156.9579.

Phenyl-cyclotetraphosphate as storable tetraphosphorylation reagent (74)

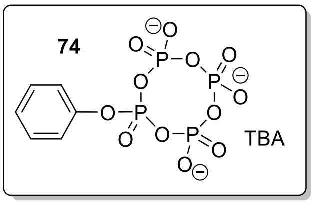

The cyclophosphate x TBA salt (**69**, 100 µmol) was dissolved in dry MeCN (ca. 70 mM) and 2-(Trimethylsilyl)phenyl-trifluoro-methanesulfonate (**1**, 121 µL, 149 mg, 500 µmol, 5.0 eq.) was added. Subsequently TBAF (1 M in THF, 5.0 eq.) was added dropwise via syringe pump (1 h, rt, needle tip is below solvent surface). Then, the reaction mixture is stirred for 15 min at rt. The product is precipitated with Et_2O (40 mL) and the resulting oil is washed with Et_2O (2 x 30 mL). The phenyl-cyclotetraphosphate **74** is dried over high vacuum and can be stored as tetraphosphorylation reagent in the fridge for weeks without substantial decomposition. Furthermore, storage as solution in MeCN at rt is possible for weeks.

^{31}P{^{1}H}-NMR (162 MHz, CD_3CN, δ/ppm): -25.03 – -25.65 (m, 3P), -29.37 – -29.89 (m, 1P). **HRMS** (ESI) m/z for $C_6H_7O_{12}P_4$ $[M-H]^-$: calcd. 394.8893, found 394.8892.

8. Synthesis of phosphate starting materials without literature precedence

General procedure E for the synthesis of mono- and diphosphates

Alcohol (or Monophosphate TBA-salt) and ETT were dissolved in DMF. $(FmO)_2P$-N*i*Pr_2 (**SI-4**) was added as solution in DMF and the resulting mixture was stirred for 1 h at rt. The solution was cooled to 0 °C and *m*CPBA (77%, 1.2 eq.) was added. The solution was stirred for 15 min at 0 °C and piperidine (10 vol%) was added. The solution was stirred for 40 min at rt.

Purification method E1:

The crude product was either precipitated with ether (40 mL) , washed with ether (40 mL) and dried over high vac. Purification was performed by automated SAX (Äkta-system, Q-Sepharose, NH_4HCO_3 – buffer). The product containing fractions were identified by NMR and the product was isolated after lyophilization.

Purification method E2:

The solvent was removed under reduced pressure. The crude product was purified by RP-MPLC [C18-AQ, dryload on celite, elution with H_2O/MeCN/ TEAA (10 mM)]. The product containing fractions were identified by NMR or HPLC and the product was isolated after lyophilization.

Afterwards cations were exchanged to TBA by Dowex or Chelex before application in subsequent reactions.

6-Hydroxyhexylphosphate (SI-13)

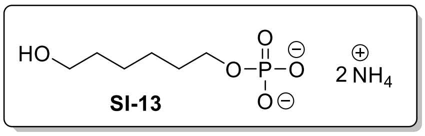

The compound was synthesized according to the general procedure E with 1,6-hexanediol (849 mg, 7.19 mmol, 5.0 eq.), $(FmO)_2P$-N*i*Pr_2 (**SI-14**, 750 mg, 1.44 mmol, 1.0 eq.), ETT (340 mg, 2.88 mmol, 2.0 eq.) and *m*CPBA (77%, 386 mg, 1.72 mmol, 1.2 eq.) in DMF (4.0 mL). Purification was performed by purification method E1 and the product (**SI-13**, 225 mg, 970 µmol, 67%) was isolated as white solid.

^{1}H-NMR (400 MHz, D_2O, δ/ppm): 3.82 (dt, *J* = 6.6 Hz, 2H), 3.59 (t, *J* = 6.6 Hz, 2H), 1.68 – 1.50 (m, 4H), 1.44 – 1.30 (m, 4H). **^{31}P{^{1}H}-NMR** (162 MHz, D_2O, δ/ppm): 1.34. **^{13}C{^{1}H}-NMR** (101 MHz, D_2O, δ/ppm): 65.48 (d, *J* = 5.4 Hz), 61.72, 31.15, 29.83 (d, *J* = 6.8 Hz), 24.68, 24.66. **HRMS** (ESI) m/z for $C_6H_{14}O_5P$ $[M-H]^-$: calcd. 197.0584, found 197.0584.

Geranylphosphate (SI-14)

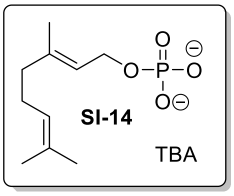

The compound was synthesized according to the general procedure E with Geraniol (210 mg, 236 µL, 1.36 mmol), $(FmO)_2P$-N*i*Pr_2 (**SI-4**, 782 mg, 1.50 mmol, 1.1 eq.), ETT (355 mg, 2.73 mmol, 2.0 eq.) and *m*CPBA (77%, 402 mg, 1.63 mmol, 1.2 eq.) in DMF (10 mL). Purification was performed by method E2 and the product (**SI-14**, 268 mg, 615 µmol, 45%) was isolated as 2.0 TEAA salt.
As the TEAA-salt was not stable in solution (decomposition by phosphate elimination) the cations were immediately changed to TBA by chelex.

^{1}H-NMR (400 MHz, D_2O, δ/ppm): 5.50 – 5.39 (m, 1H), 5.22 (tdd, *J* = 5.5, 2.9, 1.4 Hz, 1H), 4.37 (ddd, *J* = 7.2, 6.2, 0.8 Hz, 2H), 3.25 – 3.16 (m, 11H), 2.23 – 2.08 (m, 4H), 1.74 – 1.59 (m, 20H), 1.37 (h, *J* = 7.4 Hz, 11H), 0.95 (t, *J* = 7.4 Hz, 17H). **^{31}P{^{1}H}-NMR** (162 MHz, D_2O, δ/ppm): 1.61. **^{13}C{^{1}H}-NMR** (101 MHz, D_2O, δ/ppm): 142.18, 133.69, 124.13, 120.13 (d, *J* = 7.8 Hz), 61.74 (d, *J* = 4.9 Hz), 59.15 – 56.63 (m), 38.75, 25.56, 24.79, 23.11, 19.87 – 18.66 (m), 16.93, 15.46, 12.80. **HRMS** (ESI) m/z for $C_{10}H_{18}O_4P$ $[M-H]^-$: calcd. 233.0948, found 233.0946.

Isoprenylphosphate (SI-15)

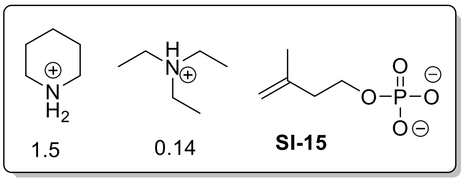

The compound was synthesized according to the general procedure E with Isoprenol (130 mg, 151 µL, 1.51 mmol), $(FmO)_2P\text{-}N\mathit{i}Pr_2$ (**SI-4**, 1.02 g, 1.97 mmol, 1.3 eq.), ETT (491 mg, 3.78 mmol, 2.5 eq.) and *m*CPBA (77%, 724 mg, 2.95 mmol, 1.5 eq.) in DMF (5.0 mL). Purification was performed by purification method E2. The product (**SI-15**, 175 mg, 573 µmol, 38%) was isolated as white solid.

^{1}H-NMR (400 MHz, D_2O, δ/ppm): 4.89 – 4.86 (m, 1H), 4.84 – 4.82 (m, 1H), 3.99 (td, *J* = 6.7, 6.6 Hz, 2H), 3.19 – 3.13 (m, 7H), 3.12 – 3.04 (m, 1H), 2.38 (d, *J* = 2549.5 Hz, 1H), 1.85 – 1.71 (m, 10H), 1.70 – 1.63 (m, 3H), 1.27 (t, *J* = 7.3 Hz, 1H). **^{13}C{^{1}H}-NMR** (101 MHz, D_2O, δ/ppm): 143.62, 111.52, 63.71 (d, *J* = 5.2 Hz), 44.51, 37.92 (d, *J* = 7.2 Hz), 27.12, 22.18, 21.55, 21.45, 14.47. **^{31}P{^{1}H}-NMR** (162 MHz, D_2O, δ/ppm): 0.49. **HRMS** (ESI) m/z for $C_5H_{10}O_4P$ $[M\text{-}H]^-$: calcd. 165.0322, found 165.0323.

Pentylphosphate (67)

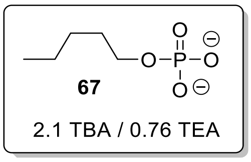

The compound was synthesized according to the general procedure E with pentan-1-ol (309 mg, 380 µL, 3.52 mmol), $(FmO)_2P\text{-}N\mathit{i}Pr_2$ (**SI-4**, 2.02 g, 3.87 mmol, 1.1 eq.), ETT (916 mg, 7.04 mmol, 2.0 eq.) and *m*CPBA (77%, 1.13 g, 5.06 mmol, 1.4 eq.) in MeCN (15 mL). Purification was performed by purification method E2. Cations were directly changed to TBA. The product (**67**, 2.65 g, 3.52 mmol, quant) was isolated colorless oil. Redundant anions are assumed to be hydroxide.

^{1}H-NMR (400 MHz, D_2O, δ/ppm): 3.85 (q, *J* = 6.7 Hz, 2H), 3.23 – 3.04 (m, 21H), 1.69 – 1.53 (m, 19H), 1.42 – 1.25 (m, 26H), 0.92 (t, *J* = 7.4 Hz, 25H), 0.89 – 0.84 (m, 3H). **^{13}C{^{1}H}-NMR** (101 MHz, D_2O, δ/ppm): 65.97 (d, *J* = 5.5 Hz), 58.58 – 56.88 (m), 46.49, 29.55 (d, *J* = 6.8 Hz), 27.15, 26.98, 23.07, 21.67, 19.24 – 18.75 (m), 14.36, 13.29, 12.80. **^{31}P{^{1}H}-NMR** (162 MHz, D_2O, δ/ppm): 0.46. **HRMS** (ESI) m/z for $C_5H_{12}O_4P$ $[M\text{-}H]^-$: calcd. 167.0479, found 167.0480.

5,5,5-Trifluorpentylphosphate (38)

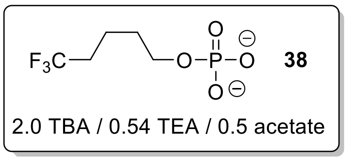

The compound was synthesized according to the general procedure E with 5,5,5-trifluoropentan-1-ol (500 mg, 370 µL, 3.52 mmol), $(FmO)_2P$-N*i*Pr_2 (**SI-4**, 2.02 g, 3.87 mmol, 1.1 eq.), ETT (916 mg, 7.04 mmol, 2.0 eq.) and *m*CPBA (77%, 1.13 g, 5.06 mmol, 1.4 eq.) in MeCN (15 mL). Purification was performed by purification method E2. Cations were directly changed to TBA. The product (**38**, 2.28 g, 2.97 mmol, 84%) was isolated colorless oil.

^{1}H-NMR (400 MHz, D_2O, δ/ppm): 3.88 (q, *J* = 6.3 Hz, 2H), 3.19 – 3.10 (m, 19H), 2.31 – 2.13 (m, 2H), 1.77 – 1.53 (m, 20H), 1.41 – 1.27 (m, 21H), 0.93 (t, *J* = 7.6 Hz, 24H). **^{13}C{^{1}H}-NMR** (101 MHz, D_2O, δ/ppm): 127.62 (q, *J* = 275.8 Hz), 65.11 (d, *J* = 5.4 Hz), 59.59 – 57.42 (m), 46.48, 32.32 (q, *J* = 27.9 Hz), 28.86 (d, *J* = 7.1 Hz), 26.99, 23.08, 17.82 (q, *J* = 3.3 Hz), 14.32, 12.79. **^{19}F-NMR** (377 MHz, D_2O, δ/ppm): -66.06 (t, *J* = 11.4 Hz). **^{31}P{^{1}H}-NMR** (162 MHz, D_2O, δ/ppm): 0.41. **HRMS** (ESI) m/z for $C_5H_9F_3O_4P$ $[M-H]^-$: calcd. 221.0196, found 221.0196.

Phenylpyrophosphat (SI-16)

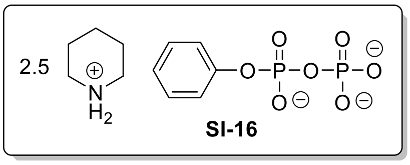

The compound was synthesized according to the general procedure E with phenylphosphate x 1.0 TBA (800 mg, 1.93 mmol), $(FmO)_2P$-N*i*Pr_2 (**SI-4**, 1.11 g, 2.13 mmol, 1.1 eq.), ETT (570 mg, 4.83 mmol, 2.5 eq.) and *m*CPBA (77%, 691 mg, 3.09 mmol, 1.6 eq.) in DMF (10 mL). The compound was purified according to the general procedure E2. The product (**SI-16**, 445 mg, 989 µmol, 51%) was isolated as white solid.

^{1}H-NMR (400 MHz, D_2O, δ/ppm): 7.45 – 7.38 (m, 2H), 7.29 – 7.25 (m, 2H), 7.24 – 7.20 (m, 1H), 3.21 – 3.11 (m, 10H), 1.83 – 1.73 (m, 10H), 1.73 – 1.59 (m, 5H). **^{13}C{^{1}H}-NMR** (101 MHz, D_2O, δ/ppm): 151.79 (d, *J* = 7.2 Hz), 129.65, 124.30 (d, *J* = 1.4 Hz), 120.55 (d, *J* = 4.3 Hz), 44.50, 22.18, 21.45. **^{31}P{^{1}H}-NMR** (162 MHz, D_2O, δ/ppm): -10.80 (d, *J* = 21.0 Hz), -15.71 (d, *J* = 20.9 Hz). **HRMS** (ESI) m/z for $C_6H_7O_7P_2$ $[M-H]^-$: calcd. 252.9672, found 252.9674.

D4T-diphosphate (SI-17)

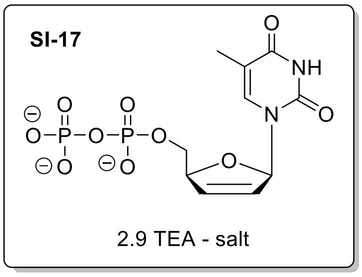

The compound was synthesized according to the general procedure E with d4T-monophosphate x 1.4 TBA (**SI-5**, 275 mg, 426 µmol), $(FmO)_2P$-N*i*Pr_2 (**SI-4**, 288 mg, 554 µmol, 1.3 eq.), ETT (139 mg, 1.07 mmol, 2.5 eq.) and *m*CPBA (77%, 204 mg, 913 µmol, 2.1 eq.) in DMF (10 mL). The crude product was precipitated with ether and subsequently purified according to the general procedure E2. The product (**SI-17**, 177 mg, 263 µmol, 62%) was isolated as white solid.

^{1}H-NMR (400 MHz, D_2O, δ/ppm): 7.62 (q, *J* = 1.4 Hz, 1H), 7.00 – 6.94 (m, 1H), 6.53 (dt, *J* = 6.2, 1.8 Hz, 1H), 5.94 (ddd, *J* = 6.5, 2.6, 1.6 Hz, 1H), 5.15 – 5.09 (m, 1H), 4.14 (ddd, *J* = 6.2, 3.4, 1.9 Hz, 2H), 3.19 (q, *J* = 7.4 Hz, 18H), 1.90 (d, *J* = 1.3 Hz, 3H), 1.28 (t, *J* = 7.3 Hz, 27H). **^{13}C{^{1}H}-NMR** (101 MHz, D_2O, δ/ppm): 166.77, 152.26, 138.18, 134.33, 125.10, 111.52, 89.95, 85.97 (d, *J* = 8.5 Hz), 66.27 (d, *J* = 5.7 Hz), 46.63, 11.43, 8.19. **^{31}P{^{1}H}-NMR** (162 MHz, D_2O, δ/ppm): -10.20 (d, *J* = 20.9 Hz), -11.42 (d, *J* = 20.7 Hz). **HRMS** (ESI) m/z for $C_{10}H_{13}N_2O_{10}P_2$ [M-H]$^-$: calcd. 383.0051, found 383.0042.

9. Synthesis of aryne precursors without literature precedence

2-bromopyrene-1-ol (SI-18)

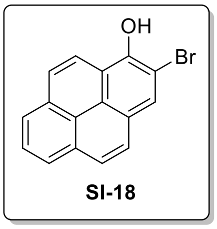

Compound **SI-18** was synthesized according to GHOTEKAR et al. The analytical data matched the previously published values.[13]

2-(trimethylsilyl)pyren-1-yl trifluoromethanesulfonate (SI-19)

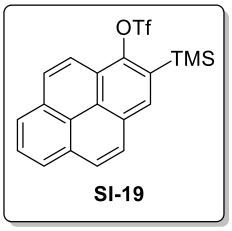

A flame-dried, argon-filled 50 mL three-necked flask equipped with a magnetic stir bar and a reflux condenser was charged with a solution of 2-bromopyrene-1-ol (**SI-18**, 1.52 g, 5.12 mmol) in THF (17 mL). Then HMDS (1.16 mL, 5.63 mmol, 1.1 eq) was added and the mixture was heated to reflux for 5 h. After cooling to rt, the solvent was removed under reduced pressure. The crude product was used in the next step without further purification.

A flame-dried, argon-filled 100 mL Schlenk flask equipped with a magnetic stir bar was charged with a solution of the crude product [((2-bromopyren-1-yl)oxy)trimethylsilane] in THF (23 mL). The solution was cooled to –78°C and *n*-BuLi was added dropwise. After stirring for 1 h at –78°C, Tf_2O was added dropwise and stirring was continued for 1 h at –78°C. An aqueous saturated $NaHCO_3$ solution (15 mL) was then added at –78°C and the mixture was allowed to warm to rt. The layers were separated, and the aqueous layer was extracted with Et_2O (3 × 20 mL). The combined organic layers were dried over Na_2SO_4 and the solvent was removed under reduced pressure. The product was purified by column chromatography (cyclohexane/ ethyl acetate, 100:1) and obtained as a yellowish solid (**SI-19**, 1.37 g, 3.23 mmol, 63%). Crystallization from hot chloroform (50°C) at room temperature gave single crystals suitable for a X-ray analysis (see below for the report).

R*f* (cyclohexane/ ethyl acetate, 100:1) = 0.45. **^{1}H-NMR** (400 MHz, $CDCl_3$, δ/ppm): 8.30 (s, 1H), 8.27 (d, *J* = 9.3 Hz, 1H), 8.20 – 8.11 (m, 3H), 8.07 – 7.90 (m, 3H), 0.66 (s, 9H). **^{13}C{^{1}H}-NMR** (101 MHz, $CDCl_3$, δ/ppm): 145.59, 132.20, 132.17, 131.16, 130.78, 130.52, 129.09, 128.55, 127.10, 126.89, 126.61, 126.28, 125.97, 123.96, 123.91, 120.40 (q, *J* = 1.7 Hz), 119.02 (q, *J* = 320.2 Hz), 0.63. **^{19}F NMR** (377 MHz, $CDCl_3$, δ/ppm): -72.59. **HRMS** (APCI) m/z for $C_{20}H_{16}F_3O_3SSi$ $[M\text{-}H]^-$: calcd. 421.0547, found 421.0550.

4-((trimethylsilyl)ethynyl)phenol (SI-20)

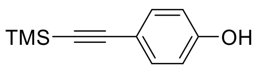

SI-20

Compound **SI-20** was synthesized according to HUDSON et al. The analytical data matched the previously published values.[14]

4-(trimethylsilyl)ethynyl)phenyl isopropylcarbamate (SI-21)

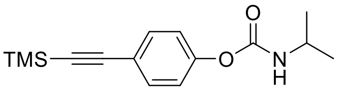

SI-21

A flame-dried, argon-filled 100 mL Schlenk flask equipped with a magnetic stir bar was charged with a solution of 4-((trimethylsilyl)ethynyl)phenol (**SI-20**, 1.90 g, 10.0 mmol) in CH_2Cl_2 (33 mL). Subsequently *i*-PrNCO (1.28 g, 15.0 mmol, 1.5 eq) was added, followed by NEt_3 (274 µL, 2.00 mmol, 0.2 eq) and then the mixture was stirred for 2 h at rt. After that the solvent was removed under reduced pressure. The residue was purified by column chromatography (cyclohexane/ ethyl acetate, 5:1) and the product (**SI-21**, 2.60 g, 9.45 mmol, 95%) was obtained as a colorless solid.

R*f* (cyclohexane/ ethyl acetate, 5:1) = 0.35. **^{1}H-NMR** (400 MHz, $CDCl_3$, δ/ppm): 7.49 – 7.40 (m, 2H), 7.16 – 7.04 (m, 2H), 4.83 (d, *J* = 7.9 Hz, 1H), 4.01 – 3.79 (m, 1H), 1.23 (d, *J* = 6.6 Hz, 6H), 0.24 (s, 9H). **^{13}C{^{1}H}-NMR** (101 MHz, $CDCl_3$, δ/ppm): 153.28, 151.24, 133.16, 121.57, 120.13, 104.61, 94.04, 43.65, 23.05, 0.12. **HRMS** (ESI) m/z for $C_{15}H_{22}NO_2Si$ $[M+H]^+$: calcd. 276.1414, found 276.1416.

2-(trimethylsilyl)-4-((trimethylsilyl)ethynyl)phenyl isopropylcarbamate (SI-22)

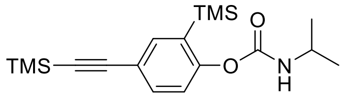

SI-22

A flame-dried, argon-filled 250 mL Schlenk flask equipped with a magnetic stir bar was charged with 4-(trimethylsilyl)ethynyl)phenyl isopropylcarbamate (**SI-21**, 2.60 g, 9.45 mmol) and Et_2O (94.5 mL). After cooling to 0°C, TMEDA (1.56 mL, 10.4 mmol, 1.1 eq) was added, followed by a solution of TBSOTf in *n*-pentane (8 mL, 1.3 M, 10.4 mmol, 1.1 eq) and then the mixture was stirred for 5 min at 0°C and further 30 min at rt. Additional TMEDA (2.83 mL, 18.9 mmol, 2.0 eq) was added and the mixture was cooled to –78°C. Then *n*-BuLi (7.60 mL, 2.48 M in *n*-hexane, 18.9 mmol, 2.0 eq) was added dropwise over 60 min. After an additional hour at –78°C, TMSCl was added dropwise over 35 min and the mixture was stirred for a further 85 min at –78°C. An aqueous saturated $NaHSO_4$ solution (40 mL) was then added at –78°C and the mixture was allowed to warm to rt. The layers were separated, and the organic layer was washed with aqueous saturated $NaHSO_4$ solution (60 mL) and brine (60 mL). The organic layer was dried over Na_2SO_4, and the solvent was removed under reduced pressure. The product was purified by column chromatography (cyclohexane/ ethyl acetate, 50:1 to 20:1) and obtained as a colorless solid (**SI-22**, 1.91 g, 5.50 mmol, 58%).

$\mathbf{R}_f$ (cyclohexane/ ethyl acetate, 10:1) = 0.20. **^{1}H-NMR** (400 MHz, $CDCl_3$, δ/ppm): 7.53 (d, *J* = 2.1 Hz, 1H), 7.46 (dd, *J* = 8.4, 2.1 Hz, 1H), 7.06 (d, *J* = 8.4 Hz, 1H), 4.86 (d, *J* = 8.0 Hz, 1H), 3.90 (dp, *J* = 8.0, 6.5 Hz, 1H), 1.22 (d, *J* = 6.6 Hz, 6H), 0.28 (s, 9H), 0.25 (s, 9H). **^{13}C{^{1}H}-NMR** (101 MHz, $CDCl_3$, δ/ppm): 155.66, 153.44, 138.64, 134.02, 132.08, 122.18, 119.97, 105.04, 93.82, 43.57, 23.01, 0.13, -0.87. **HRMS** (ESI) m/z for $C_{18}H_{30}NO_2Si_2$ $[M+H]^+$: calcd. 348.1810, found 348.1808.

Synthesis of 2-(trimethylsilyl)-4-((trimethylsilyl)ethynyl)phenyl trifluoromethane-sulfonate (SI-23)

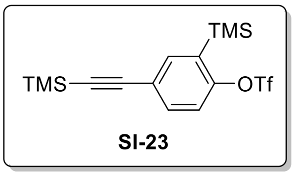

A flame-dried, argon-filled 250 mL Schlenk flask equipped with a magnetic stir bar was charged with a solution of 2-(trimethylsilyl)-4-((trimethylsilyl)ethynyl)phenyl isopropylcarbamate (**SI-22**, 1.51 g, 4.36 mmol) in MeCN (44 mL). Then DBU (980 µL, 6.54 mmol, 1.5 eq), and Et_2NH (540 µL, 5.23 mmol, 1.2 eq) were added, and the mixture was heated to 40°C and stirred for 45 min. The reaction mixture was cooled to rt and a solution of $PhNTf_2$ (2.34 g, 6.54 mmol, 1.5 eq) in MeCN (13 mL) was added dropwise and stirred for 2 h. After that a saturated aqueous $NaHSO_4$ solution (30 mL) was added and the mixture was diluted with ethyl acetate (30 mL). The layers were separated, and the organic layer was washed with saturated aqueous $NaHSO_4$ solution (30 mL) and aqueous NaOH (10%, 2 × 30 mL). The organic layer was dried over Na_2SO_4 and the solvent was removed under reduced pressure. The residue was purified by column chromatography (*n*-pentane) and the product (**SI-23**, 1.43 g, 3.62 mmol, 83%) was isolated as a colorless liquid.

^{1}H-NMR (400 MHz, $CDCl_3$, δ/ppm): 7.59 (dd, *J* = 2.2, 0.4 Hz, 1H), 7.51 (dd, *J* = 8.6, 2.2 Hz, 1H), 7.28 (d, *J* = 8.6 Hz, 1H), 0.37 (s, 9H), 0.26 (s, 9H). **^{13}C{^{1}H}-NMR** (101 MHz, $CDCl_3$, δ/ppm): 154.51, 139.87, 134.77, 133.08, 123.01, 119.51 (d, *J* = 1.7 Hz), 118.61 (q, *J* = 320 Hz), 103.46, 96.26, 0.00, -0.79. **^{19}F NMR** (377 MHz, $CDCl_3$, δ/ppm): -73.83. **HRMS** (APCI) m/z for $C_{15}H_{22}F_3O_3SSi_2$ $[M+H]^+$: calcd. 395.0775, found 395.0775.

10. Synthesis of Amino-DEACM

The synthetic route towards amino-DEACM (**SI-26**) is shown in the supporting figure 5 below. Mesylate **SI-24** was synthesized according to Wong et al.[15] Analytical data were in accordance with literature.

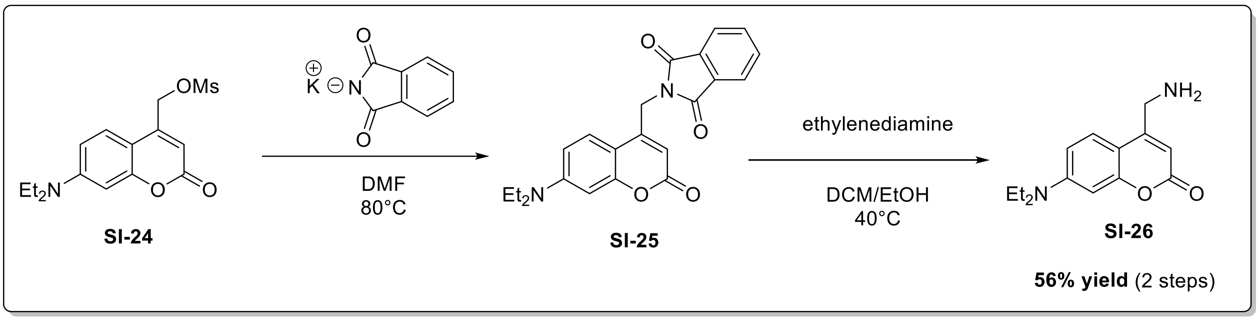

Supporting figure 5: Synthesis of Amino-DEACM **SI-26** from Mesylate **SI-24**.

Step 1: 2-((7-(diethylamino)-2-oxo-2H-chromen-4-yl)methyl)isoindoline-1,3-dione (**SI-25**)

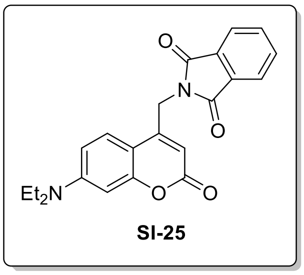

Potassium phthalimide (990 mg, 5.30 mmol, 1.2 eq.) was added to a solution of (7-(diethylamino)-2-oxo-2H-chromen-4-yl)methyl methanesulfonate (**SI-24**, 1.50 g, 4.61 mmol, 1.0 eq.) in DMF (90 mL) and it was stirred for 2 h at 80 °C. Afterwards the reaction mixture was poured into ice water (450 mL). The yellow precipitate was collected *via* Büchner funnel and was dried in a desiccator, over $CaCl_2$ and under vacuum, for 3 days. The dry solid was purified by recrystallization (toluene, 45 mL) and the title compound (**SI-25**, 950 mg, 2.52 mmol, 57%) was obtained as dark yellow crystals.

^{1}H-NMR (400 MHz, $CDCl_3$, δ/ppm): 7.95 – 7.86 (m, 2H), 7.81 – 7.74 (m, 2H), 7.55 (d, *J* = 9.0 Hz, 1H), 6.62 (dd, *J* = 9.0, 2.6 Hz, 1H), 6.51 (d, *J* = 2.6 Hz, 1H), 5.86 (t, *J* = 1.2 Hz, 1H), 4.94 (d, *J* = 1.2 Hz, 2H), 3.42 (q, *J* = 7.1 Hz, 4H), 1.21 (t, *J* = 7.1 Hz, 6H). **^{13}C{^{1}H}-NMR** (101 MHz, $CDCl_3$, δ/ppm): 167.61, 161.79, 156.34, 150.76, 149.14, 134.47, 131.83, 124.66, 123.74, 108.73, 106.80, 106.57, 97.88, 44.78, 37.61, 12.45. **HRMS** (APCI) m/z for $[C_{22}H_{21}N_2O_4]^+$: calcd. 377.1496, found 377.1491. **R_f** = 0.54 (silica gel, CH:EA 1:1).

Step 2: 4-(aminomethyl)-7-(diethylamino)-2H-chromen-2-one (Amino-DEACM) (**SI-26**)

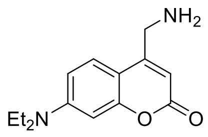

SI-26

Ethylenediamine (220 µL, 198 mg, 3.3 mmol, 5.0 eq.) was added to a solution of 2-((7-(diethylamino)-2-oxo-2H-chromen-4-yl)methyl)isoindoline-1,3-dione (**SI-25**, 250 mg, 664 µmmol, 1.0 eq.) in DCM/EtOH (1:1, 25 mL). The solution was stirred for 5 h at 40 °C. Afterwards, the reaction mixture was directly dry loaded on deactivated silica (ca. 4 spatulas) and purified by flash chromatography (deactivated silica gel, DCM:MeOH 95:5). *Attention:* Solvent removal should be carried out below 35°C. The title compound (**SI-26**, 160 mg, 650 µmol, 98%) was obtained as a yellow oil.

^{1}H-NMR (300 MHz, $CDCl_3$, δ/ppm): 7.37 (d, J = 9.0 Hz, 1H), 6.57 (dd, J = 9.0, 2.6 Hz, 1H), 6.50 (d, J = 2.6 Hz, 1H), 6.18 (t, J = 1.3 Hz, 1H), 3.98 (d, J = 1.3 Hz, 2H), 3.40 (q, J = 7.1 Hz, 4H), 1.53 (br. s, 2H), 1.19 (t, J = 7.1 Hz, 6H). **^{13}C{^{1}H}-NMR** (101 MHz, $CDCl_3$, δ/ppm): 162.51, 156.60, 156.24, 150.47, 124.39, 108.50, 107.10, 105.68, 97.87, 44.74, 42.27, 12.46. **HRMS** (ESI) m/z for $[C_{14}H_{19}N_2O_2]^+$: calcd. 247.1441 found 247.1442. **R_f** = 0.10 (deactivated silica gel, DCM:MeOH 95:5).

11.Supporting references

[1] B. G. Ugarkar, A. J. Castellino, J. S. DaRe, M. Ramirez-Weinhouse, J. J. Kopcho, S. Rosengren, M. D. Erion, J. *Med. Chem.* **2003**, *46*, 22, 4750-4760.

[2] D. K. Dean, Synth. Commun. 2002, 32, 1517-1521.

[3] J. Singh, N. Steck, D. De, A. Hofer, A. Ripp, I. Captain, M. Keller, P. A. Wender, R. Bhandari, H. J. Jessen, *Angew. Chem. Int. Ed.* **2019**, *58*, 3928-3933.

[4] L. Bialy, H. Waldmann, *Angew. Chem.* **2002**, 114, 1819-1822.

[5] S. Yang, C. Pannecouque, E. Lescrinier, A. Giraut, P. Herdewijn, *Org. Biomol. Chem.* **2012**, *10*, 146-153.

[6] Y. Ueta, K. Mikami, S. Ito, *Angew. Chem. Int. Ed.* **2016**, *55*, 7525-7529.

[7] Y. Wang, A. D. Stretton, M. C. McConnell, P. A. Wood, S. Parsons, J. B. Henry, A. R. Mount, T. H. Galow, *J. Am. Chem. Soc.* **2007**, *129*, 13193-13200.

[8] D. Xu, Y. Zhao, D. Song, Z. Zhong, S. Feng, X. Xie, X. Wang, X. She, *Org. Lett.* **2017**, *19*, 3600-3603.

[9] D. Peña, A. Cobas, D. Pérez, E. Guitián, *Synthesis* **2002**, *10*, 1454-1458.

[10] G. S. Ghotekar, A. C. Shaikh, M. Muthukrishnan, *J. Org. Chem.* **2019**, *84*, 2269-2276.

[11] R. N. Bell, L. F. Audrieth, O. F. Hill, *Ind. Eng. Chem. Res.* **1952**, *44*, 568-572.

[12] T. Glonek, J. R. van Wazer, M. Mudgett, T. Myers, *Inorg. Chem.* **1972**, *11*, 567-570.

[13] A. H. G. David, R. Casares, J. M. Cuerva, A. G. Campaña, V. Blanco, *J. Am. Chem. Soc.* **2019**, *141*, 18064-18074.

[14] S. A. Hudson, K. J. McLean, S. Surade, Y.-Q. Yang, D. Leys, A. Ciulli, A. W. Munro, C. Abell, *Angew. Chem. Int. Ed.* **2012**, *51*, 9311-9316.

[15] P. T. Wong, E. W. Roberts, S. Tang, J. Mukherjee, J. Cannon, A. J. Nip, K. Corbin, M. F. Krummel, S. K. Choi, *ACS Chem. Biol.* **2017**, *12*, 1001.

12. NMR - spectra

(aligned according to molecule numbering)

Compound 27, phenyl phosphate, ¹H - NMR (D_2O, 400 MHz)

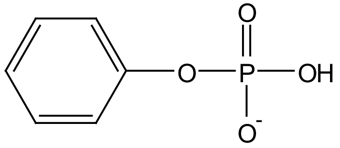

TBA salt

27

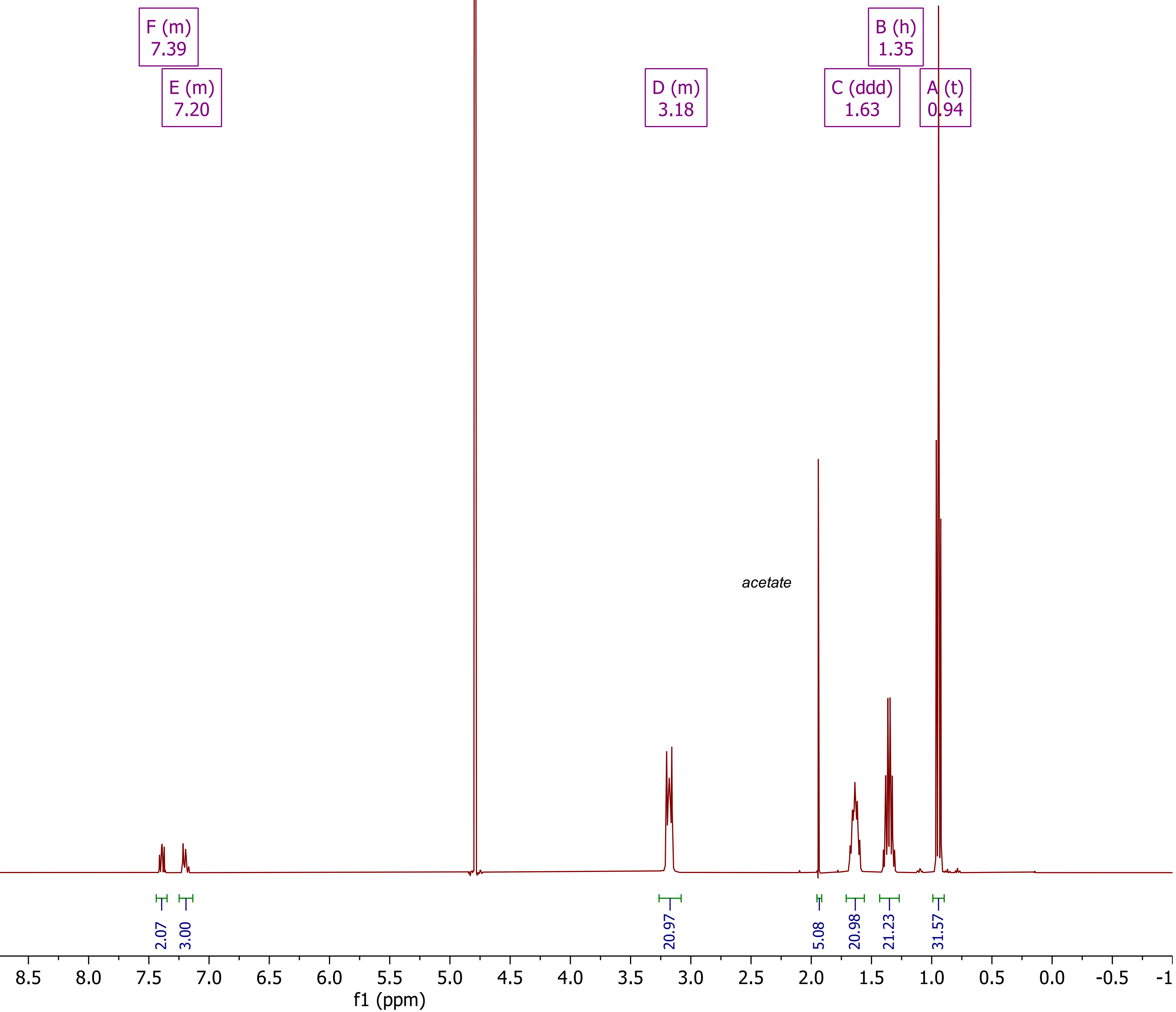

Compound 27, phenyl phosphate, $^{31}P\{^1H\}$ - NMR (D_2O, 162 MHz)

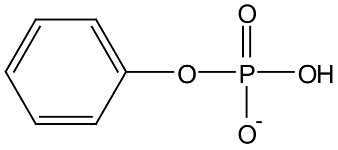

TBA salt

27

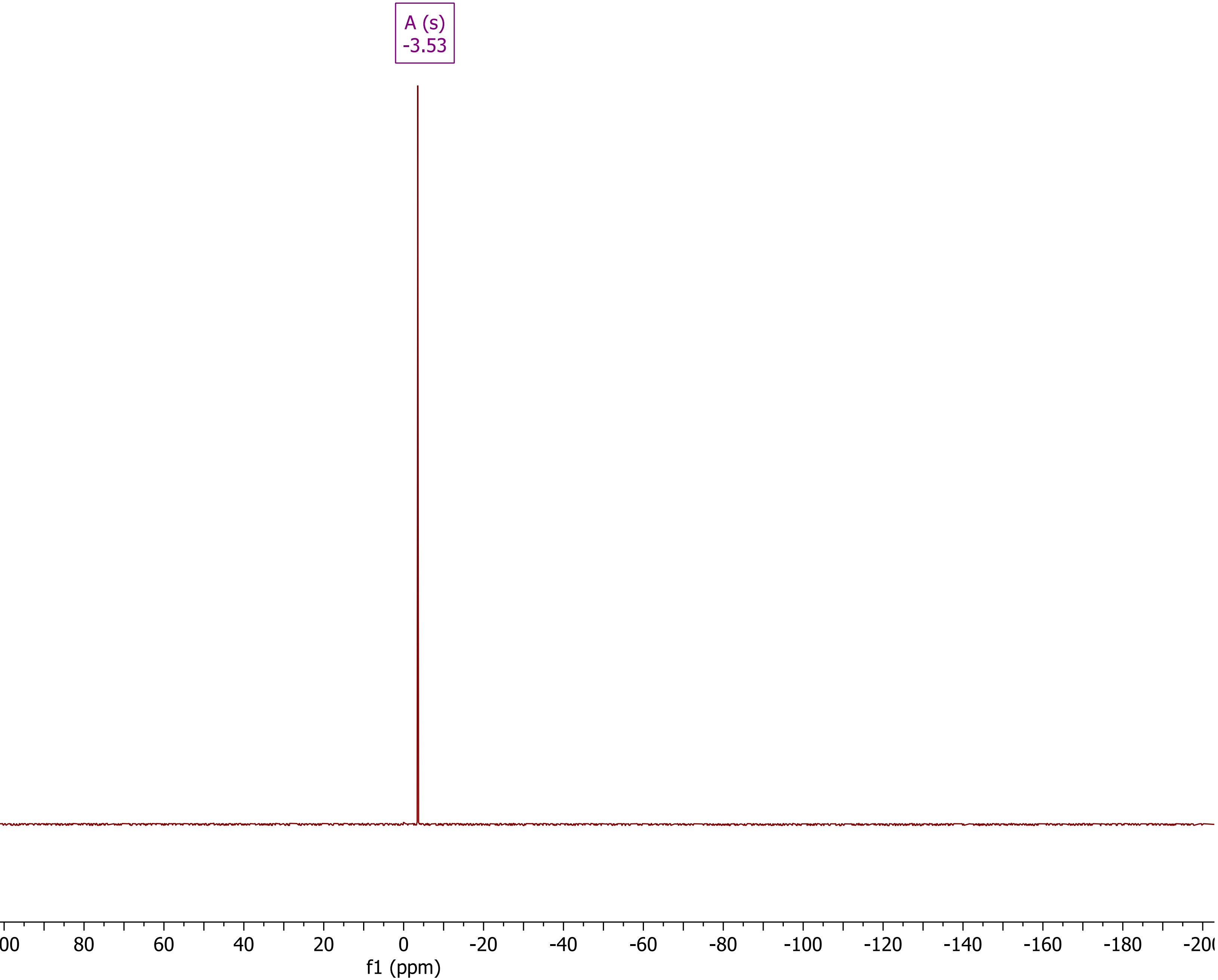

Compound 27, phenyl phosphate, ^{13}C - NMR (D_2O, 101 MHz)

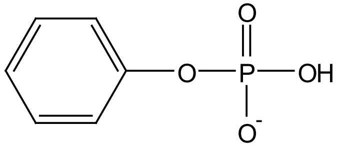

TBA salt

27

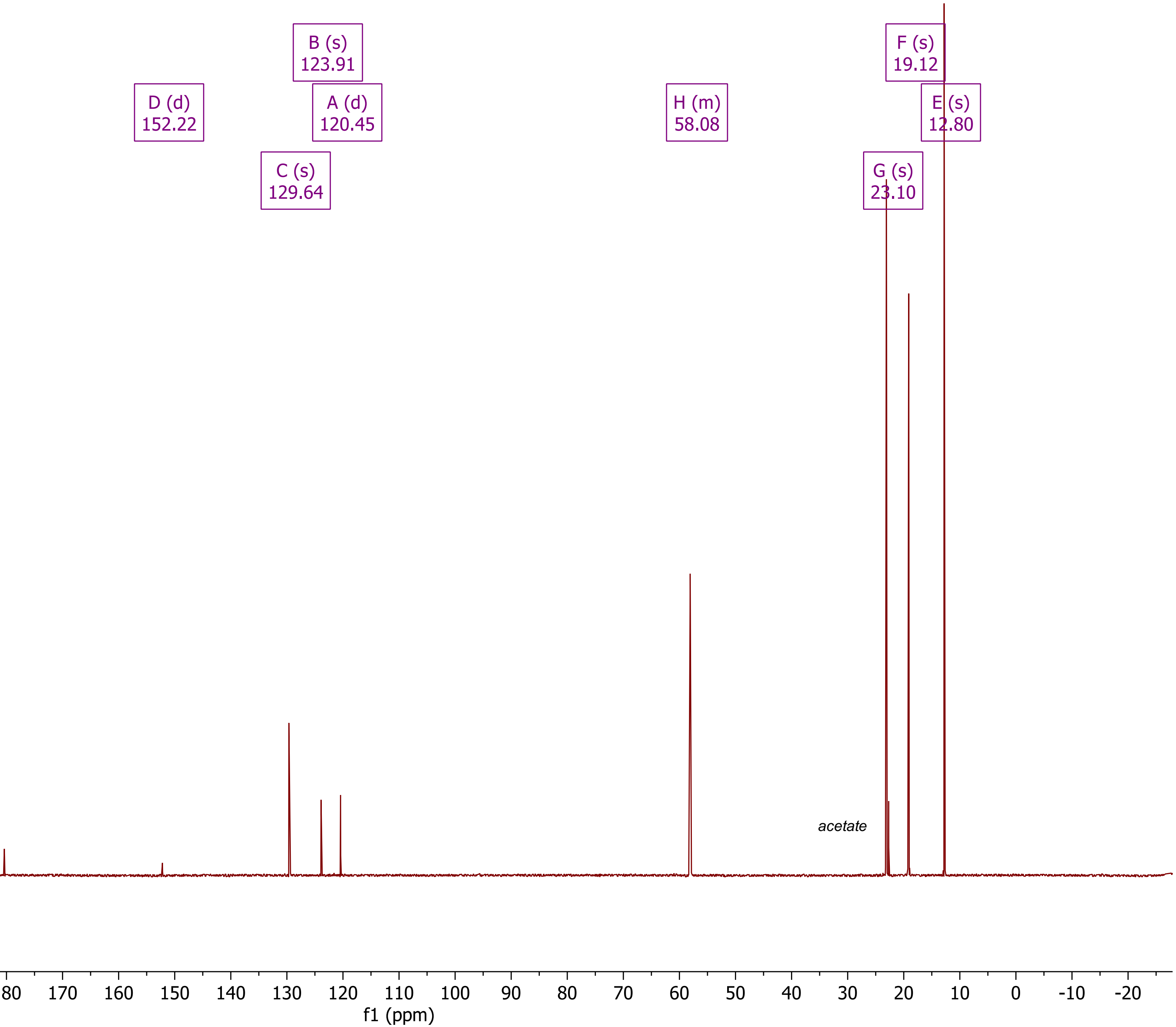

Compound 28, 2-napthalen-2-yl phosphate, ^{1}H - NMR (D_2O, 400 MHz)

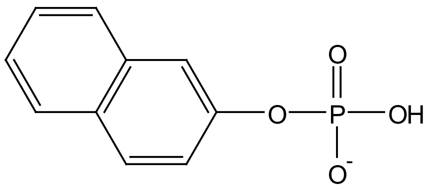

TBA salt

28

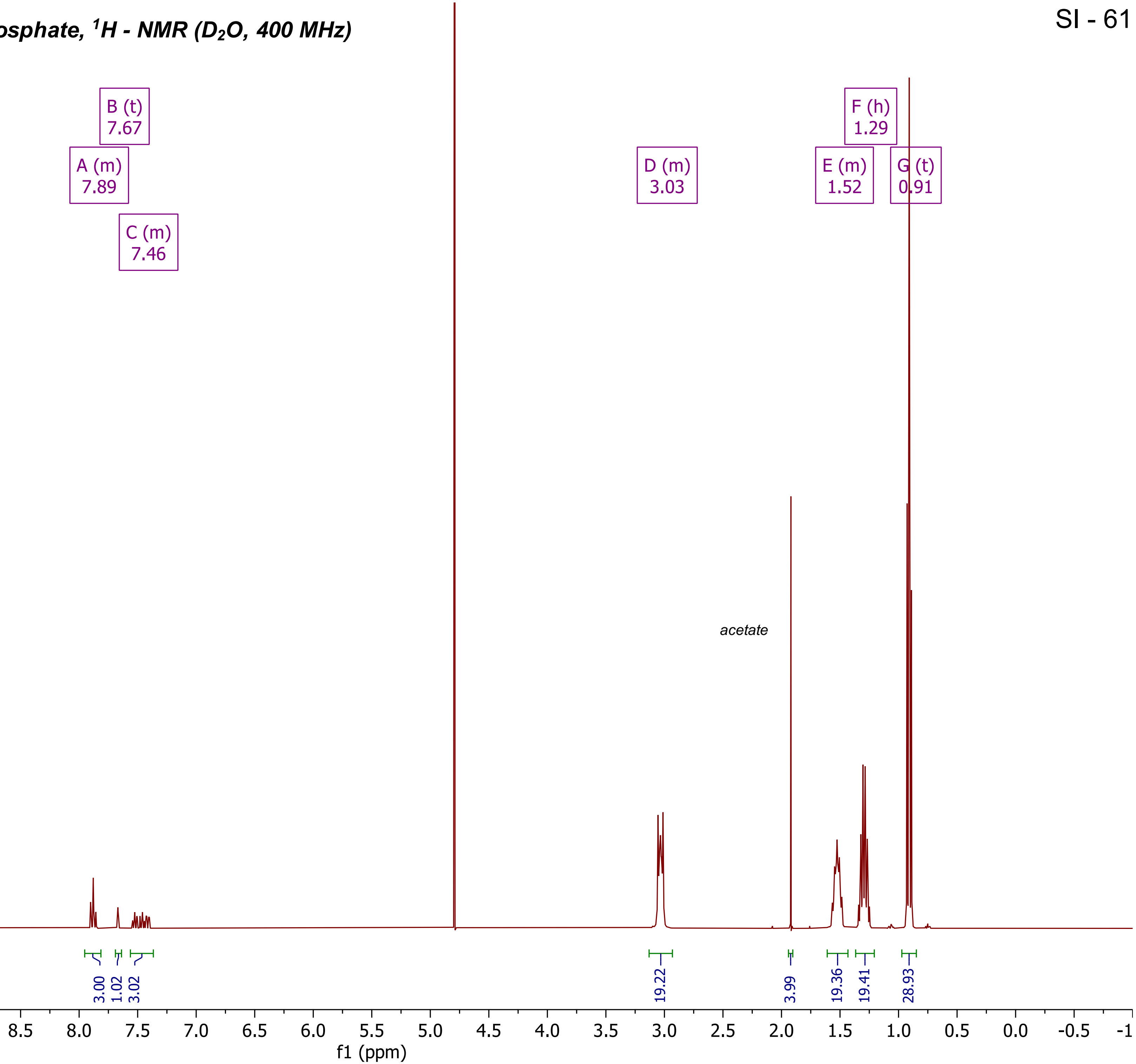

Compound 28, 2-napthalen-2-yl phosphate, $^{31}P\{^1H\}$ - NMR (D_2O, 162 MHz)

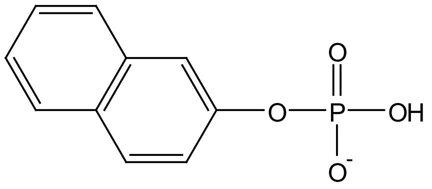

TBA salt

28

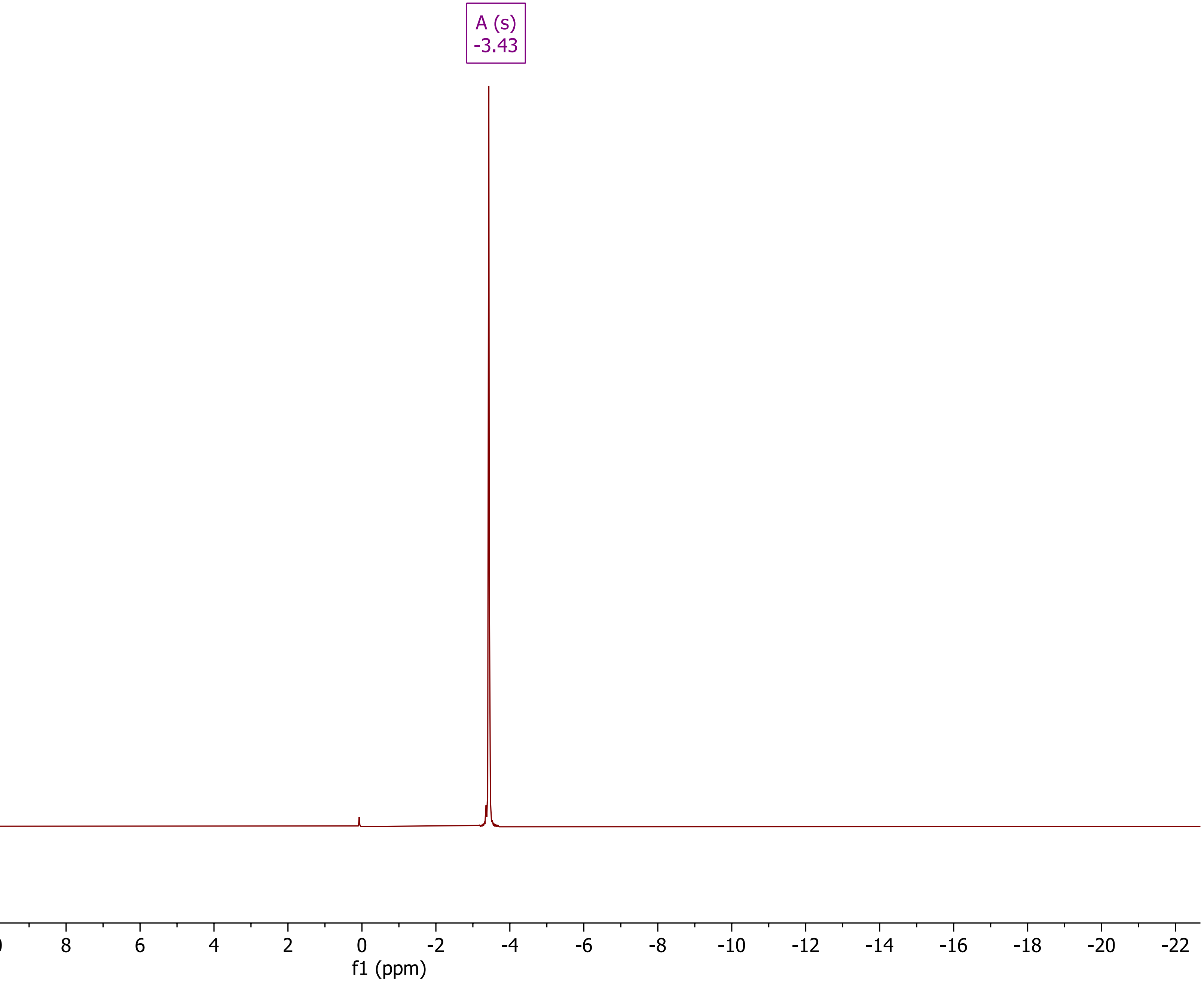

Compound 28, 2-napthalen-2-yl phosphate, ^{13}C - NMR (D_2O, 101 MHz)

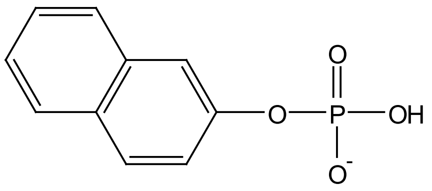

TBA salt

28

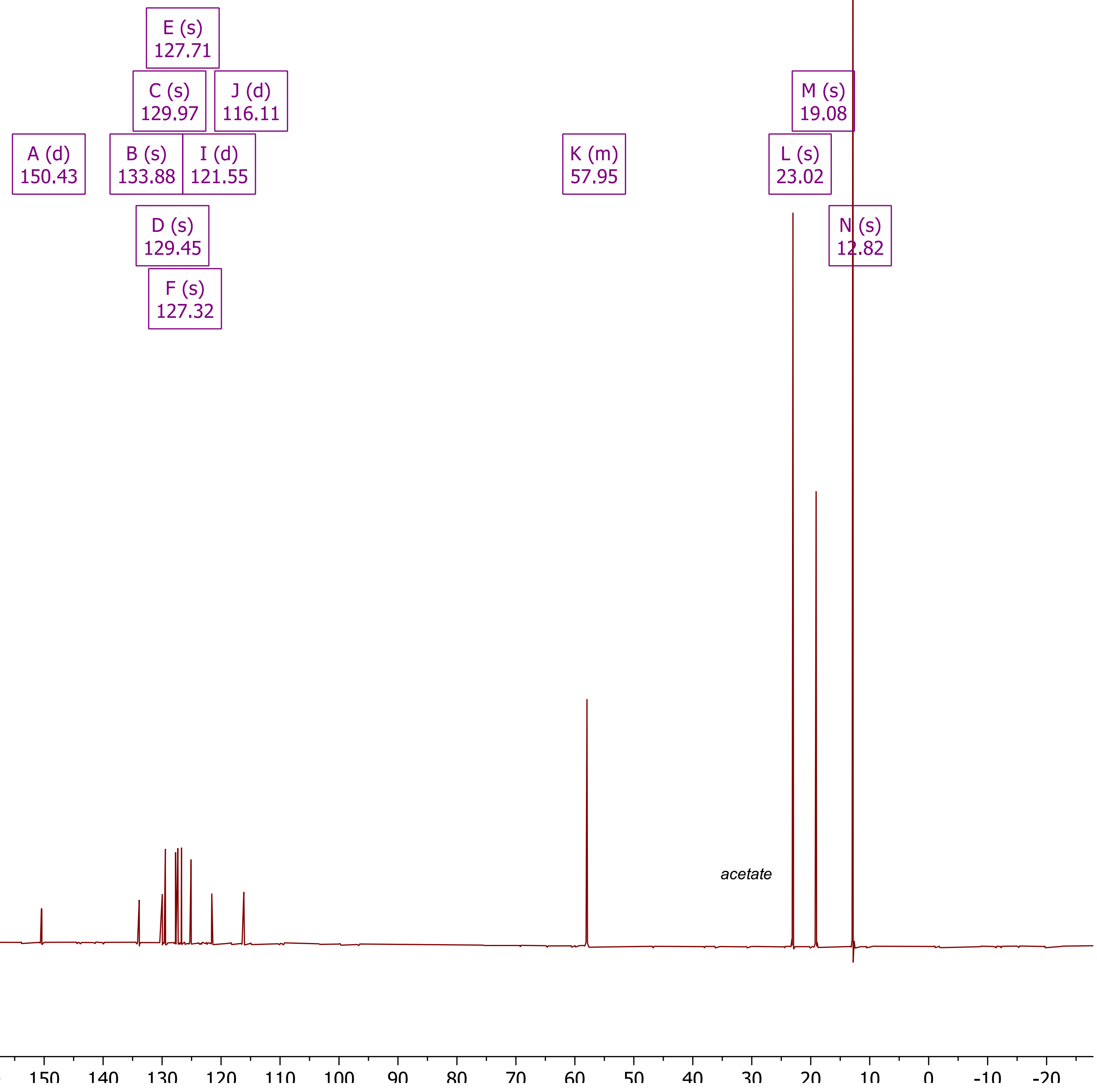

Compound 29, 3,4-dimethylphenyl phosphate, ^{1}H - NMR (D_2O, 400 MHz)

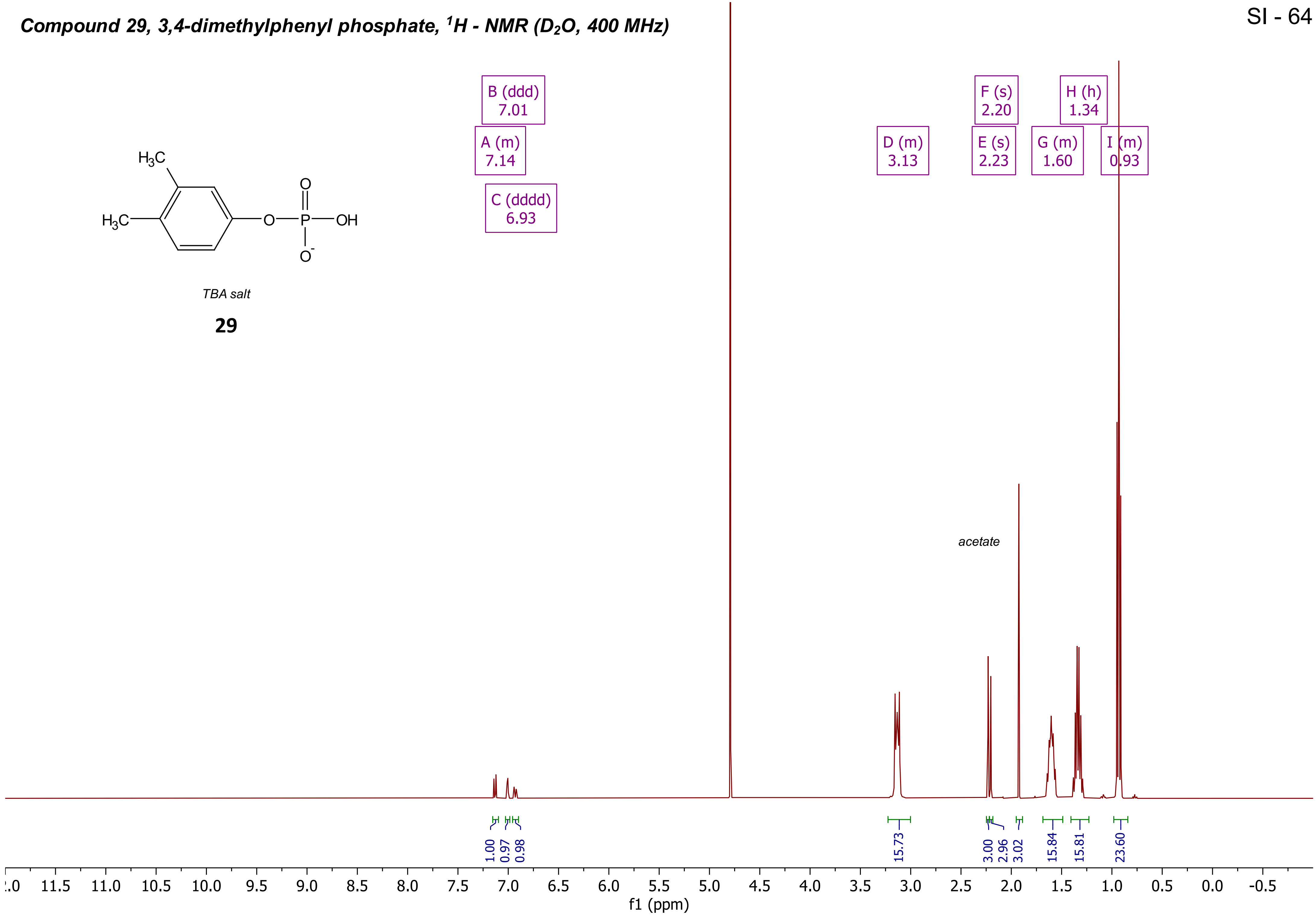

Compound 29, 3,4-dimethylphenyl phosphate, $^{31}P\ \{^{1}H\}$ - NMR (D_2O, 162 MHz)

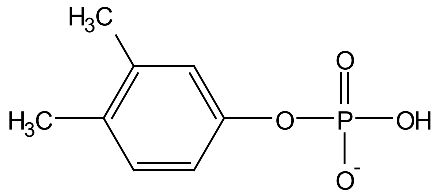

TBA salt

29

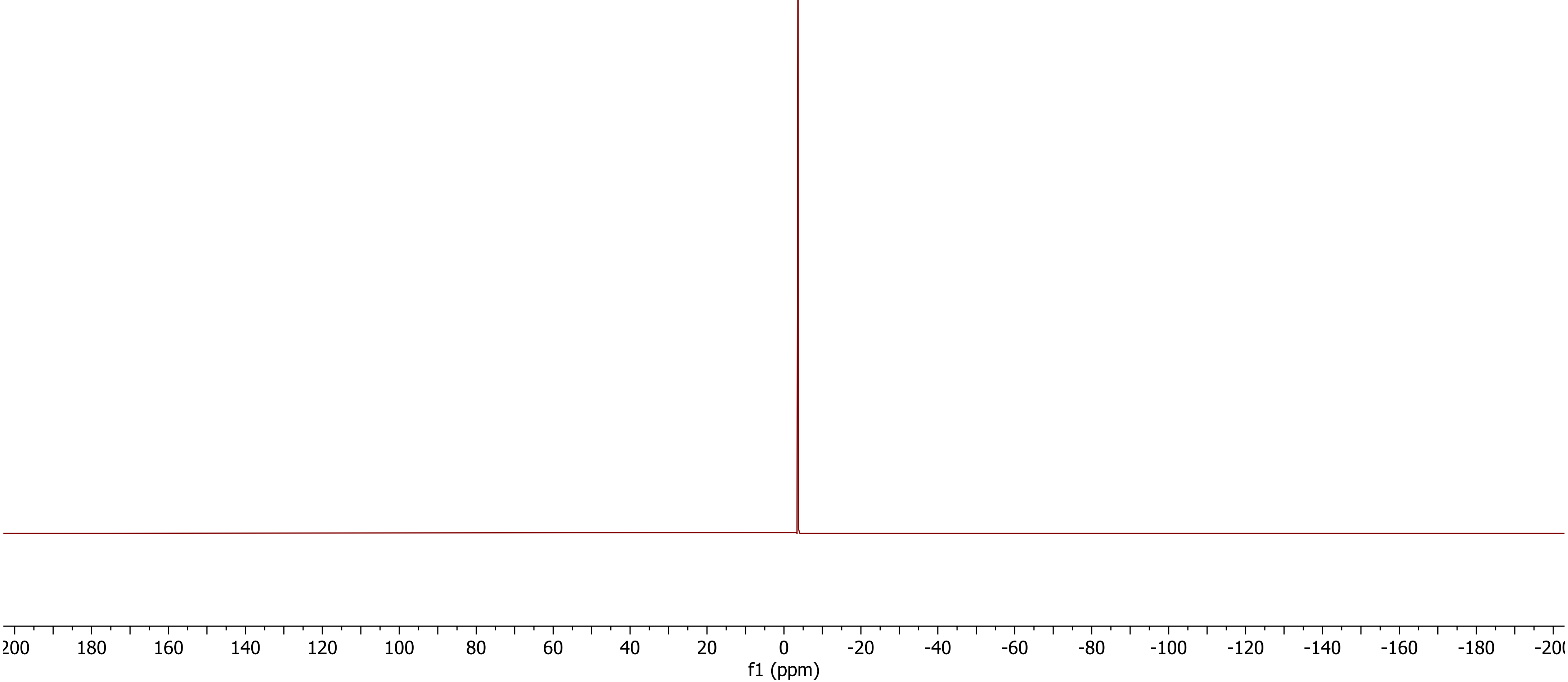

Compound 29, 3,4-dimethylphenyl phosphate, ^{13}C - NMR (D_2O, 101 MHz)

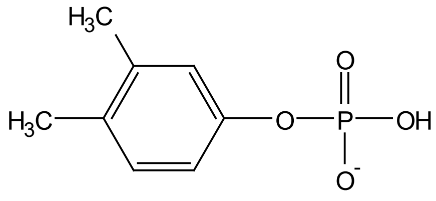

TBA salt

29

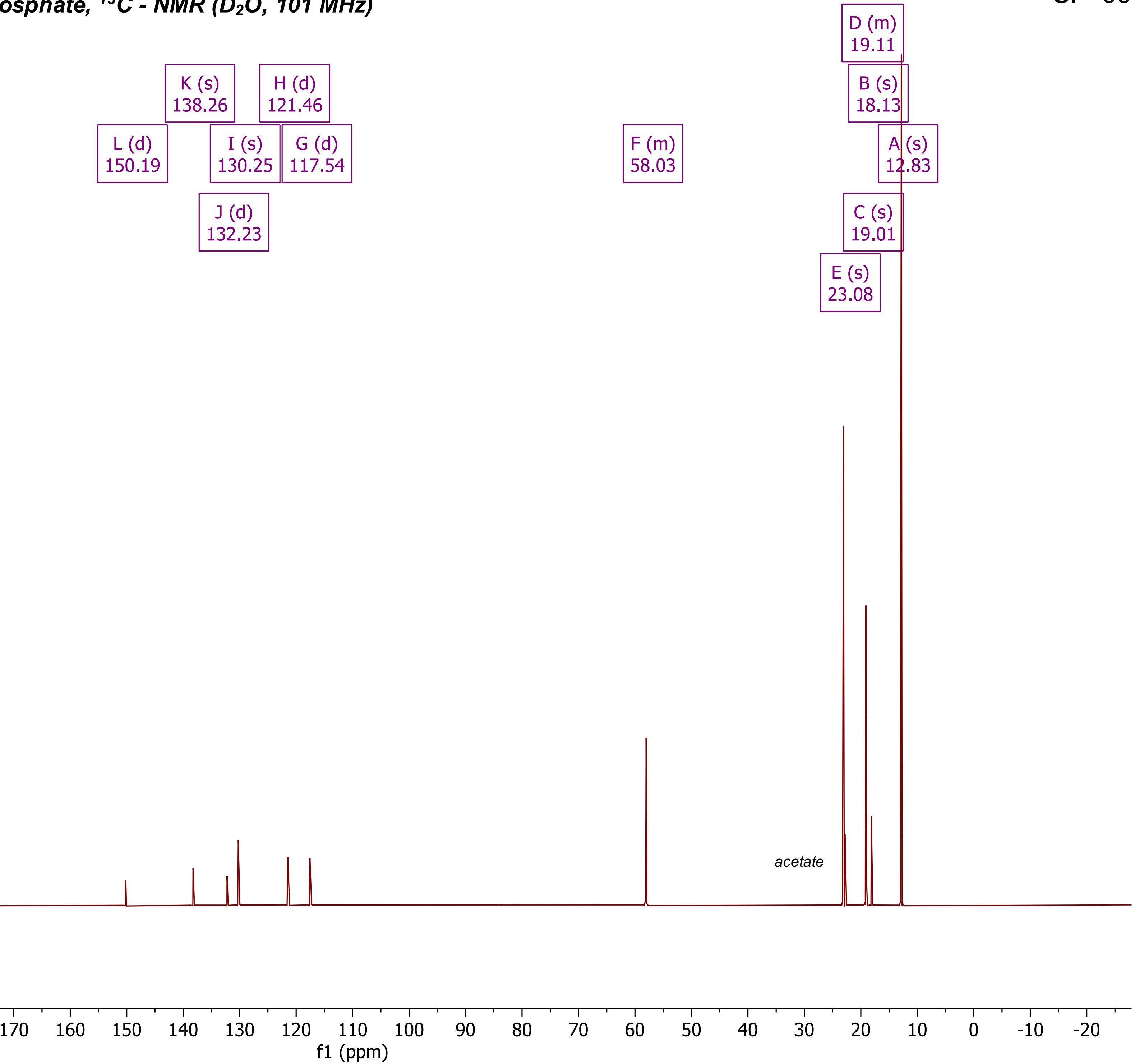

Compound 30, 1H-indol-5-yl phosphate, ^{1}H - NMR (D_2O, 400 MHz)

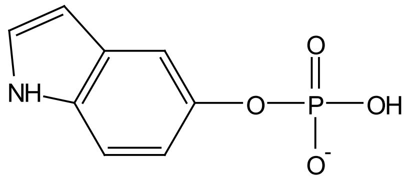

TBA salt

30

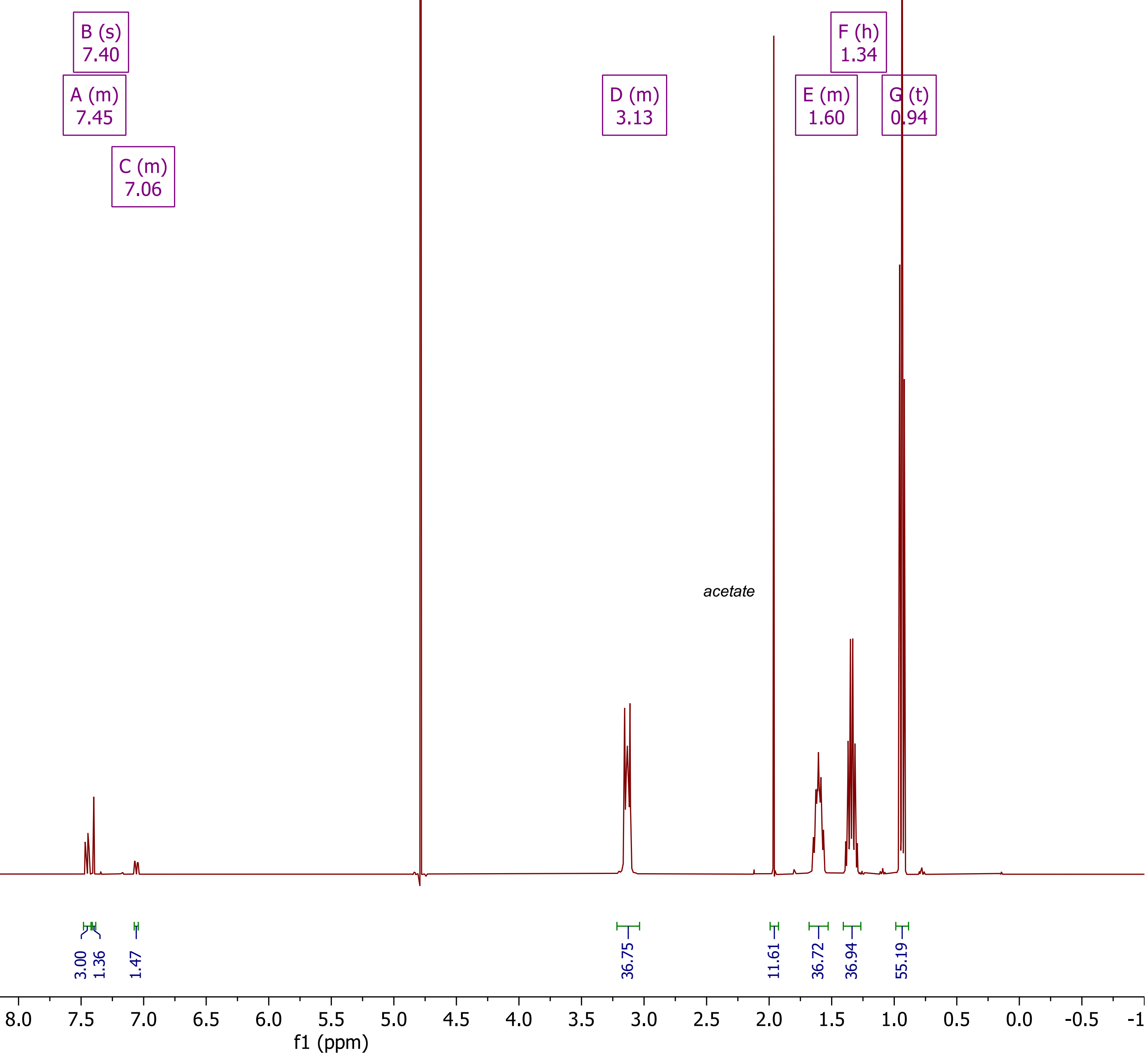

Compound 30, 1H-indol-5-yl phosphate, $^{31}P\{^{1}H\}$ - NMR (D_2O, 162 MHz)

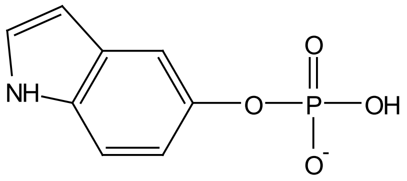

TBA salt

30

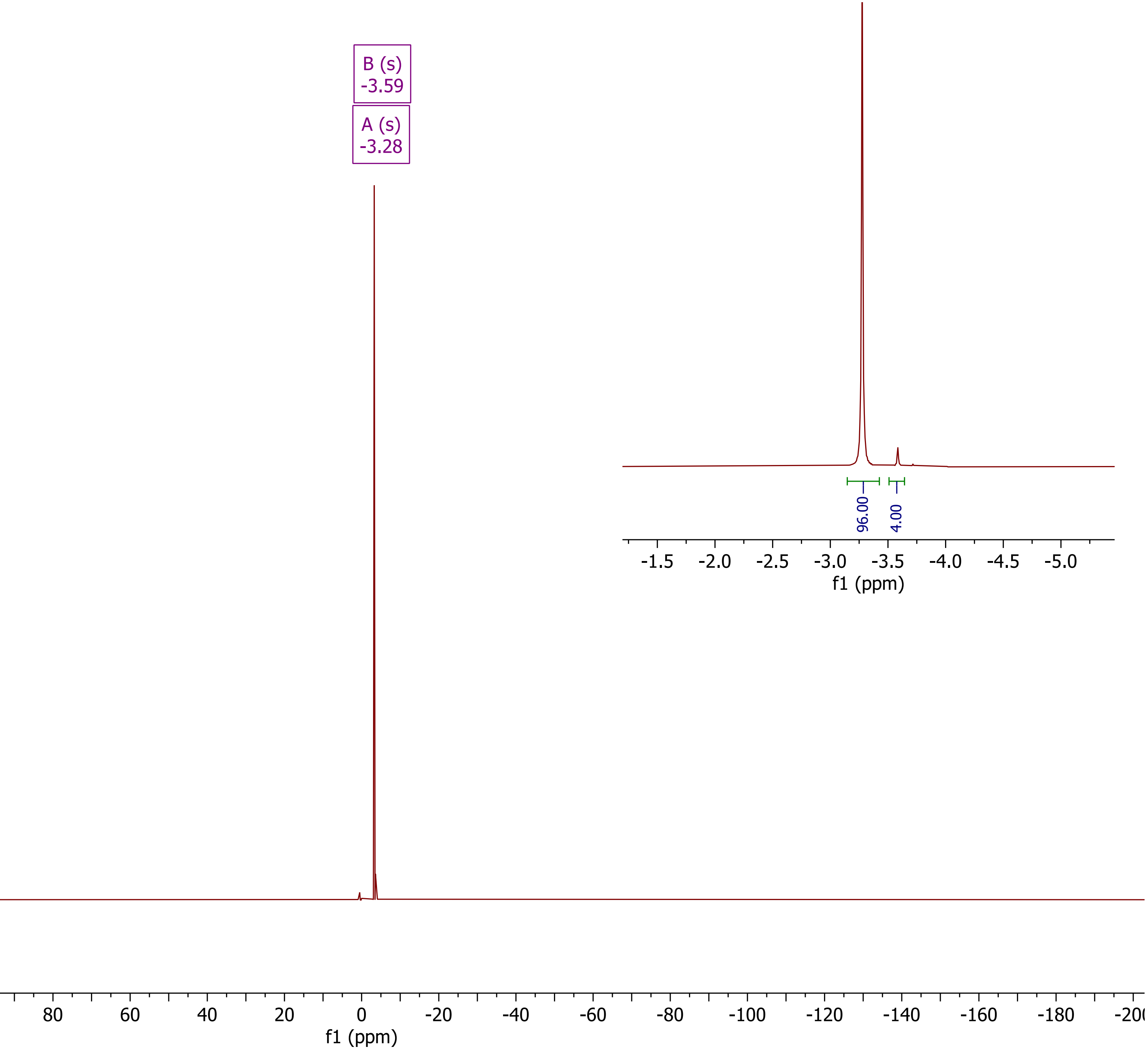

Compound 30, 1H-indol-5-yl phosphate, ^{13}C - NMR (D_2O, 101 MHz)

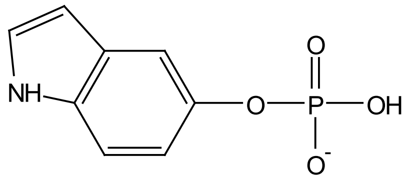

TBA salt

30

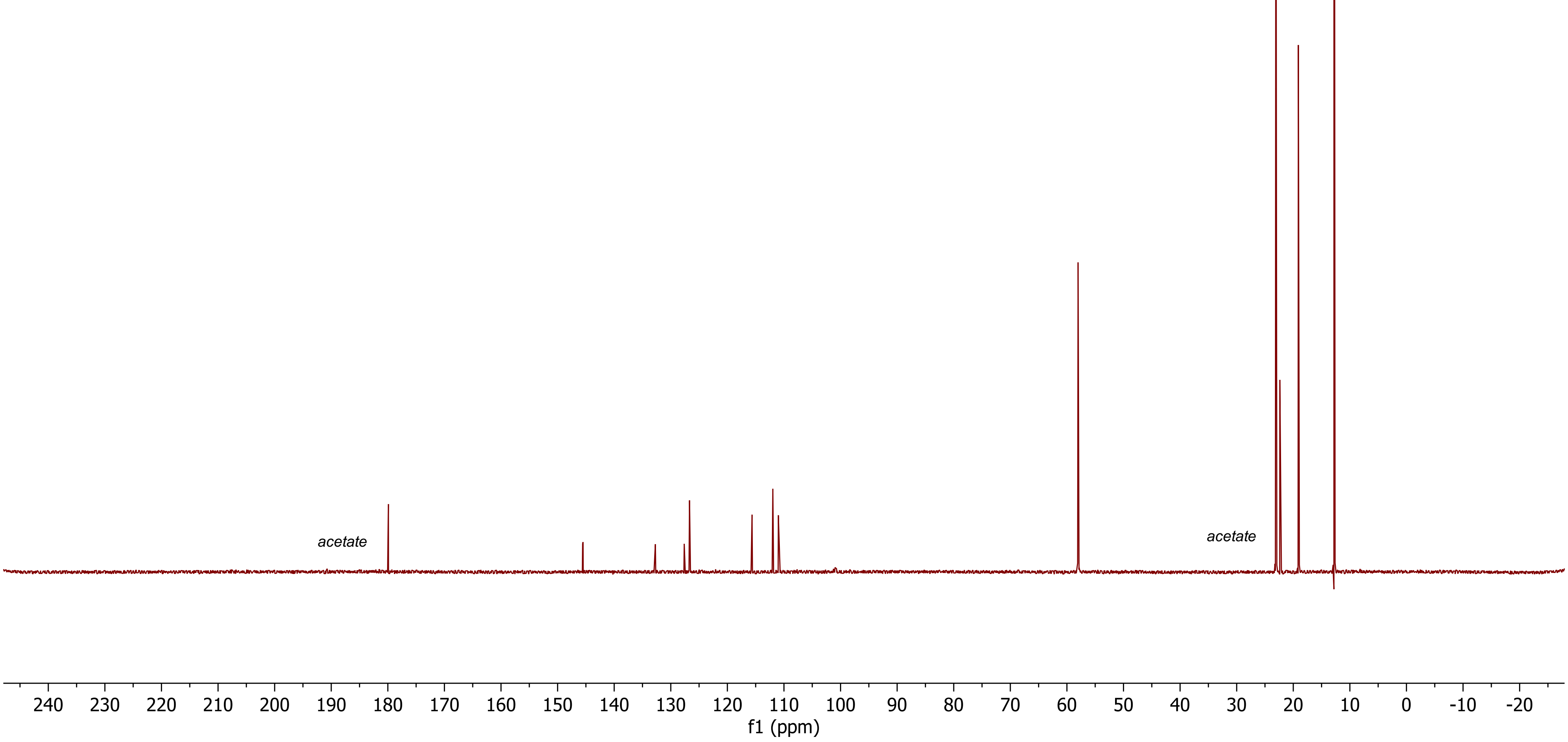

Compound 31, pyren-2-yl phosphate, ¹H - NMR (CD_3CN, 400 MHz)

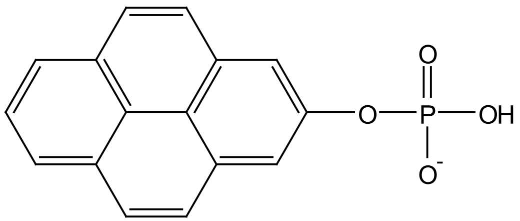

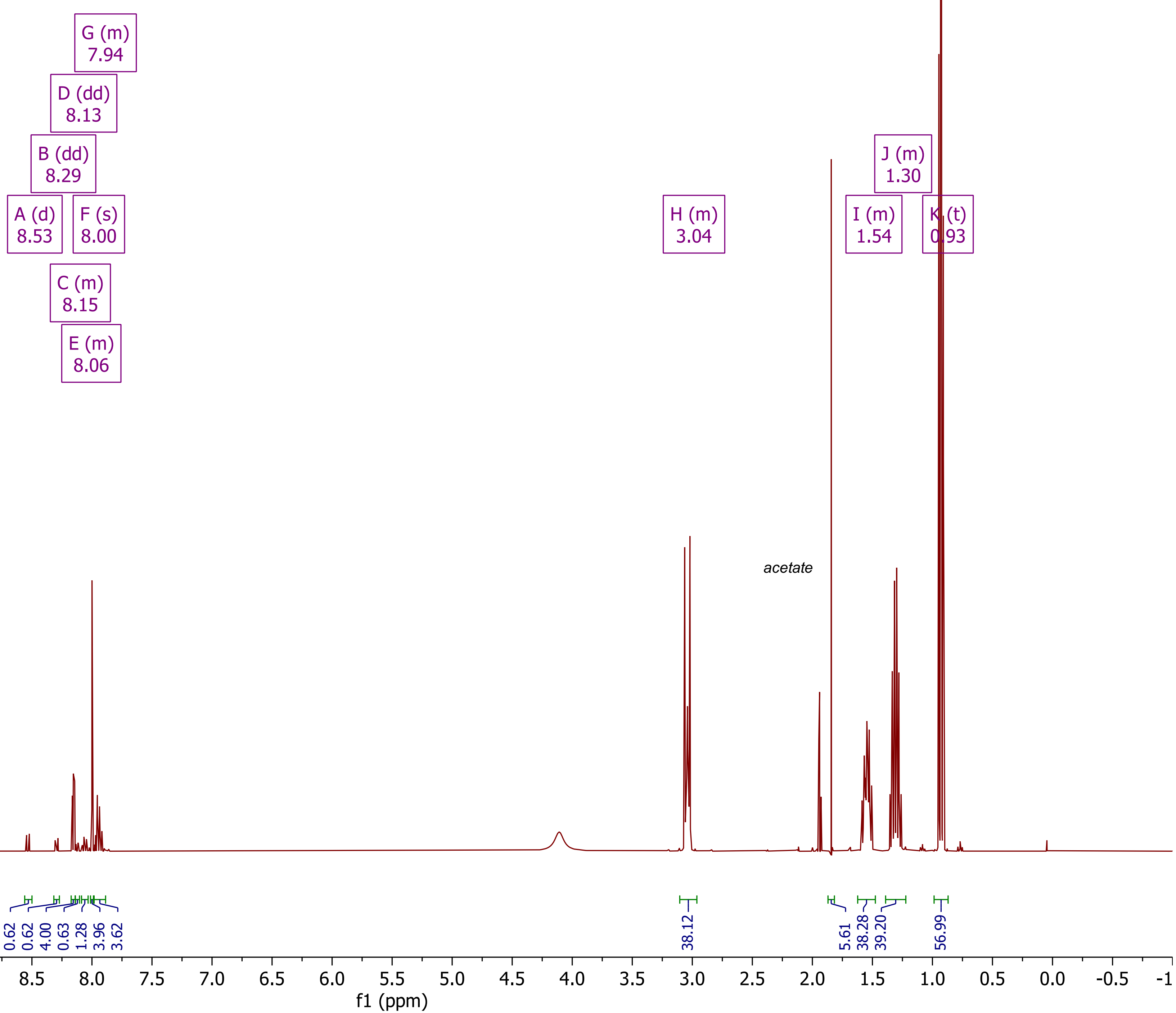

Compound 31, pyren-2-yl phosphate, $^{31}P\ \{^1H\}$ - NMR (CD_3CN, 122 MHz)

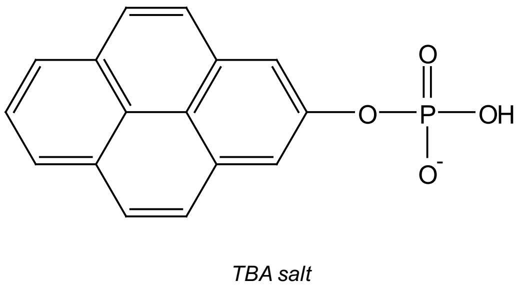

31

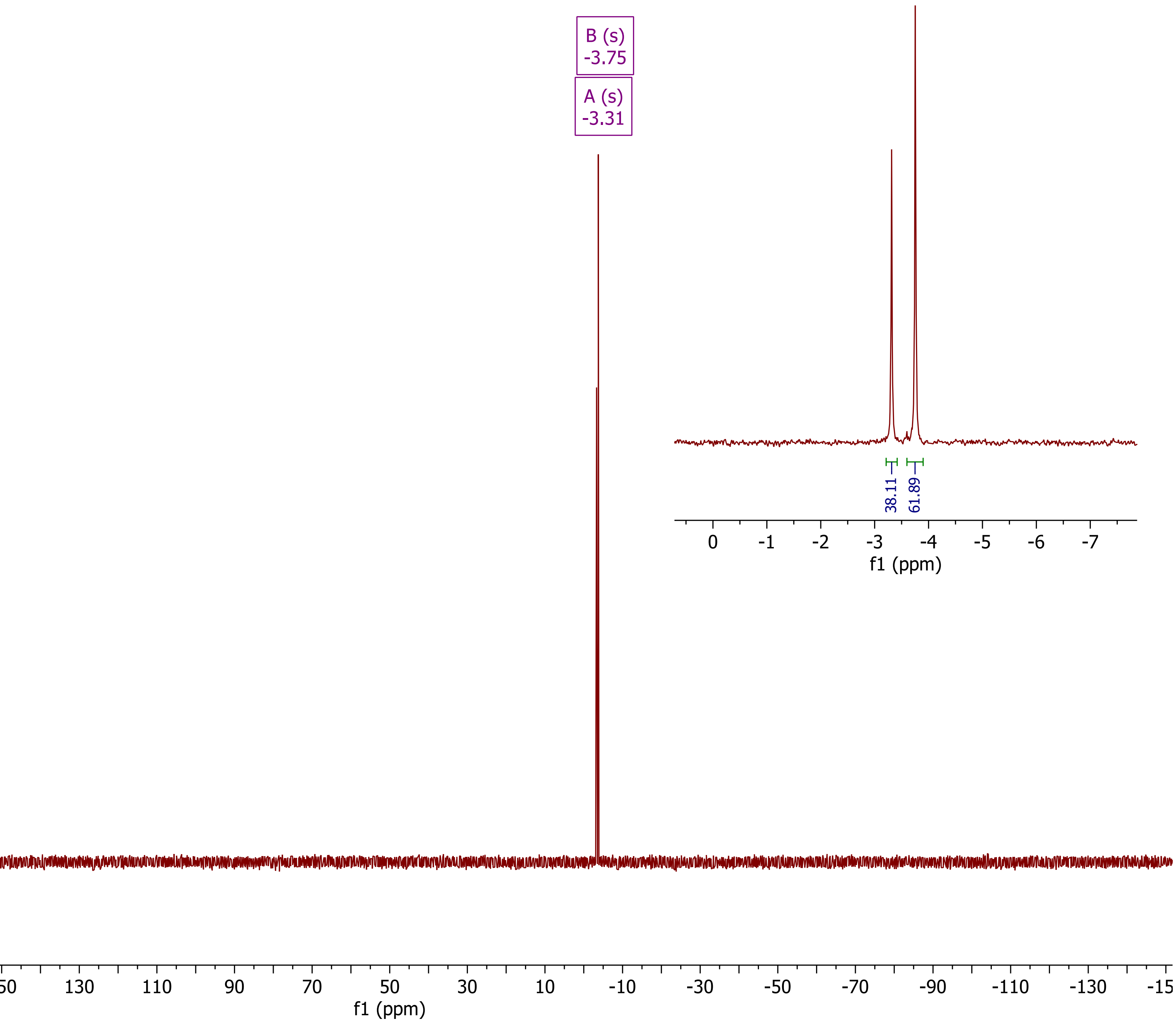

Compound 31, pyren-2-yl phosphate, ^{13}C - NMR (CD_3CN, 101 MHz)

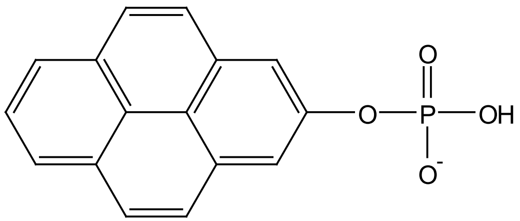

TBA salt

31

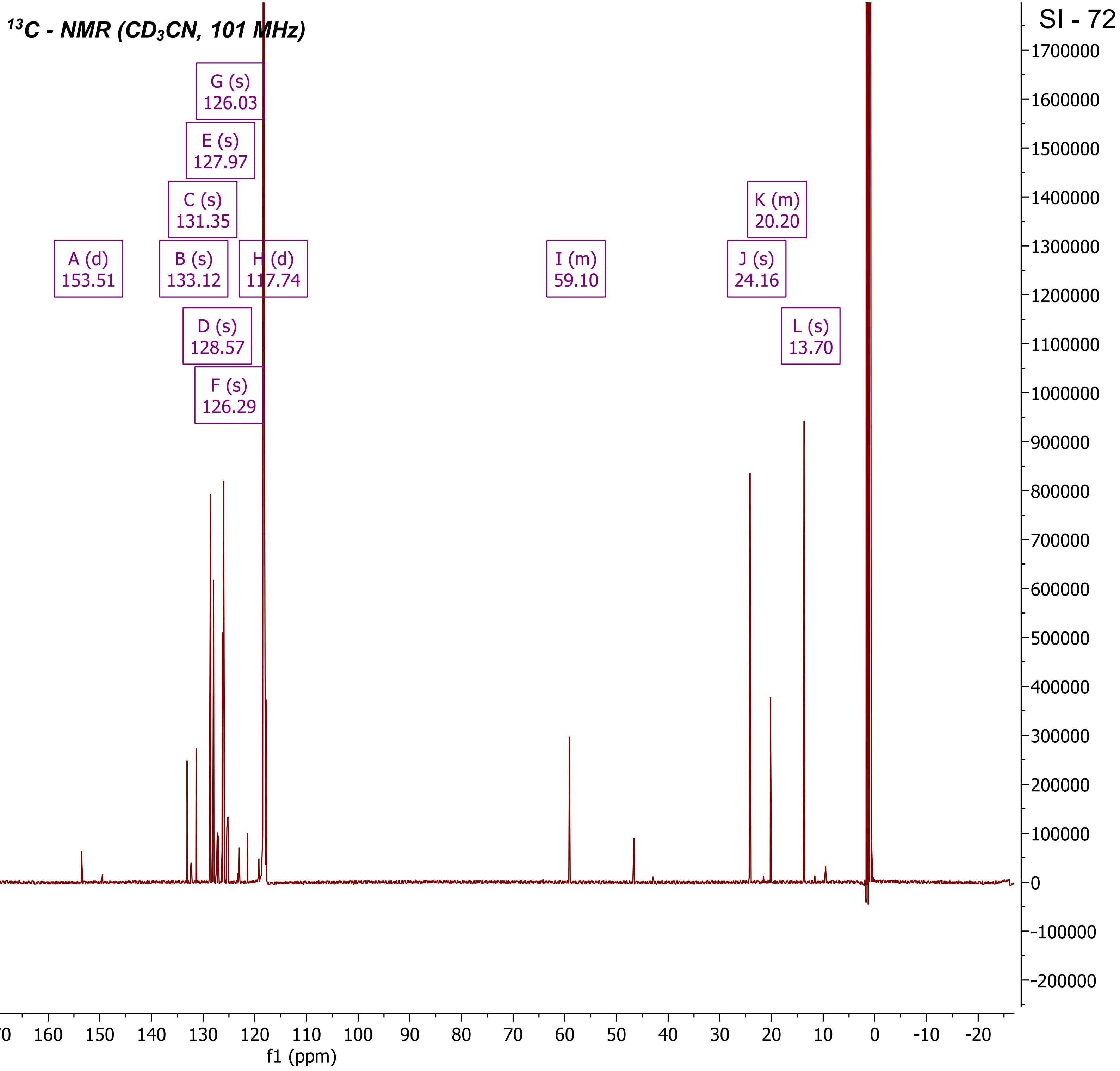

Compound 32, phenyl diphosphate, ^{1}H - NMR (D_2O, 400 MHz)

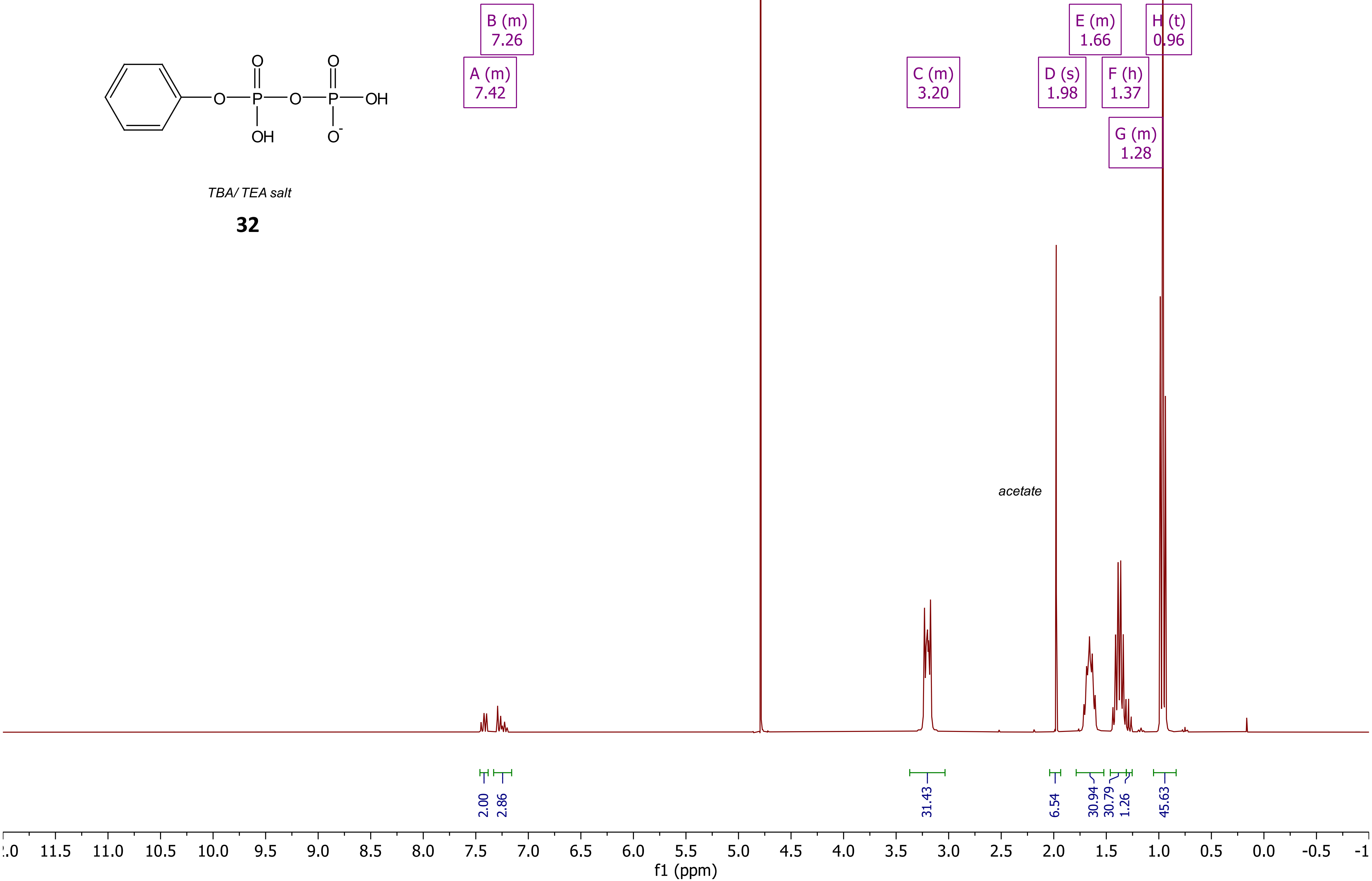

Compound 32, phenyl diphosphate, ^{31}P {^{1}H} - NMR (D_2O, 122 MHz)

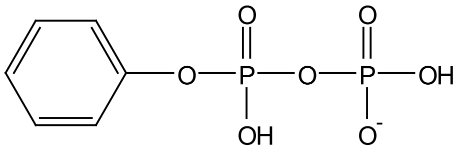

TBA/ TEA salt

32

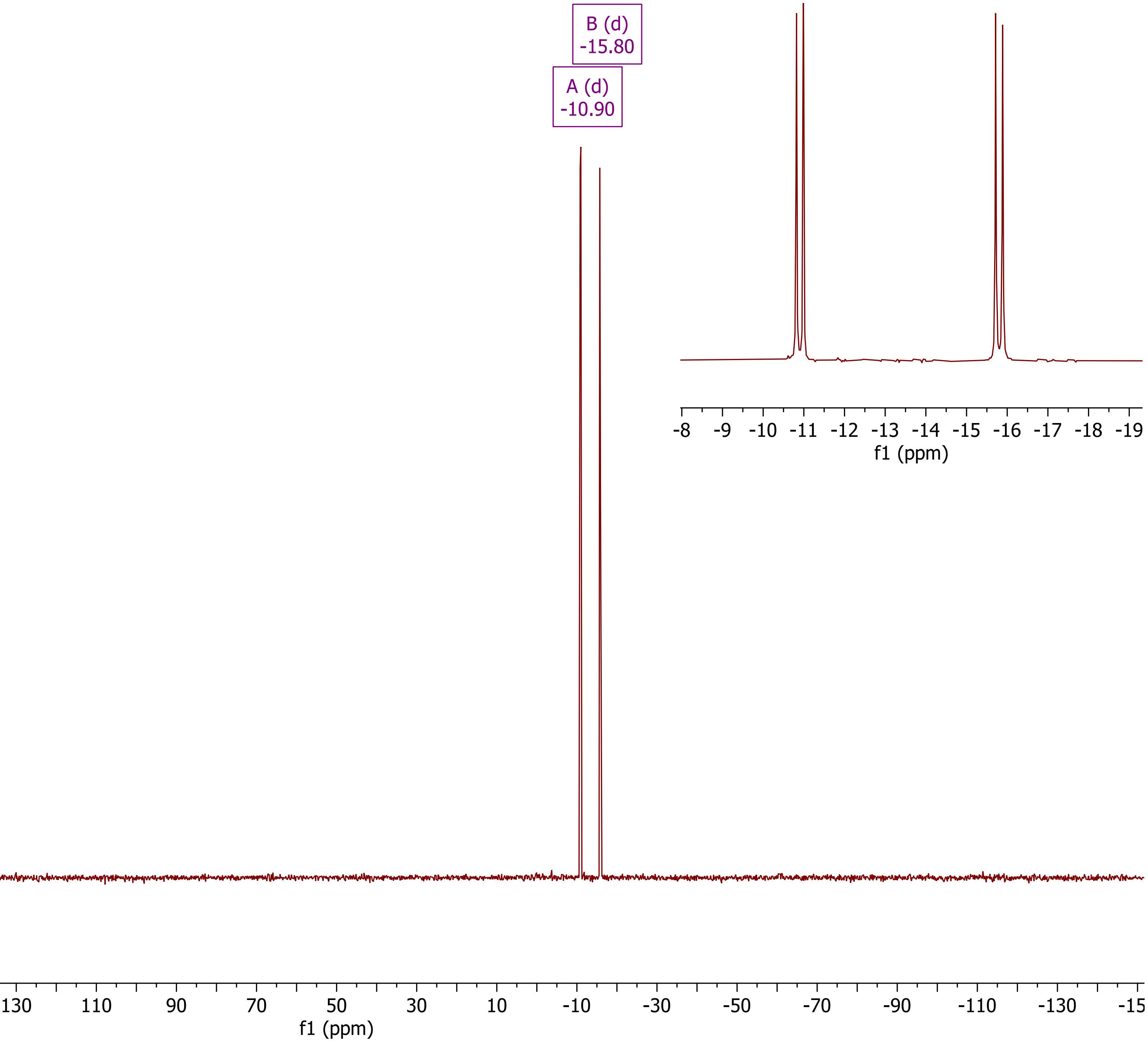

Compound 32, phenyl diphosphate, ^{13}C - NMR (D_2O, 101 MHz)

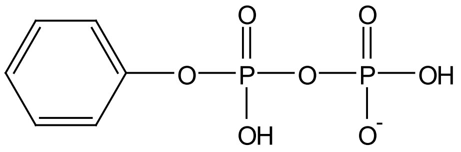

TBA/ TEA salt

32

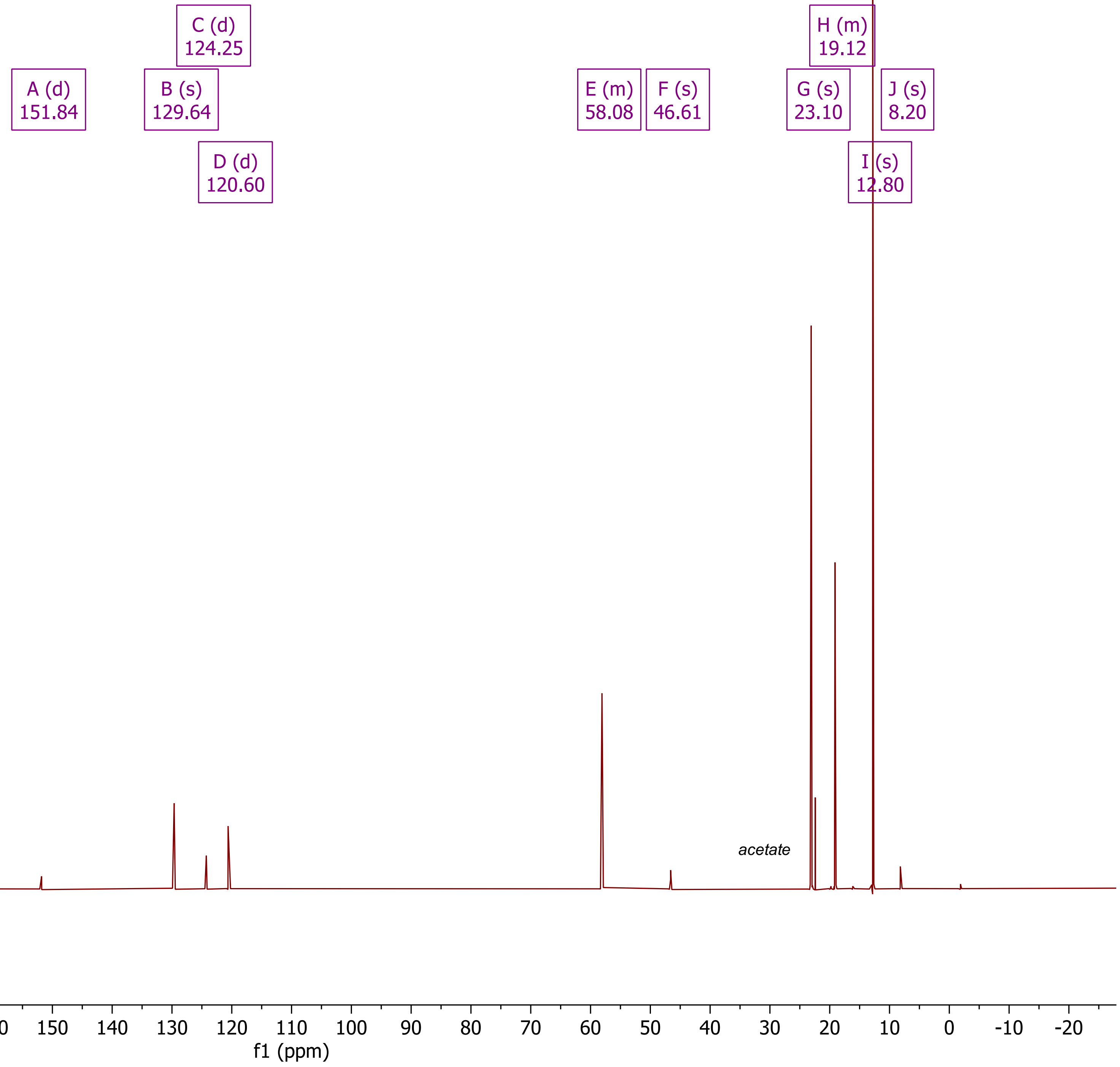

Compound 33, 2-naphthalen-2-yl diphosphate, ^{1}H - NMR (D_2O, 400 MHz)

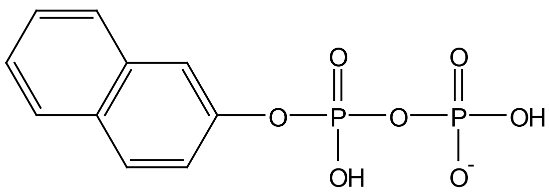

TBA salt

33

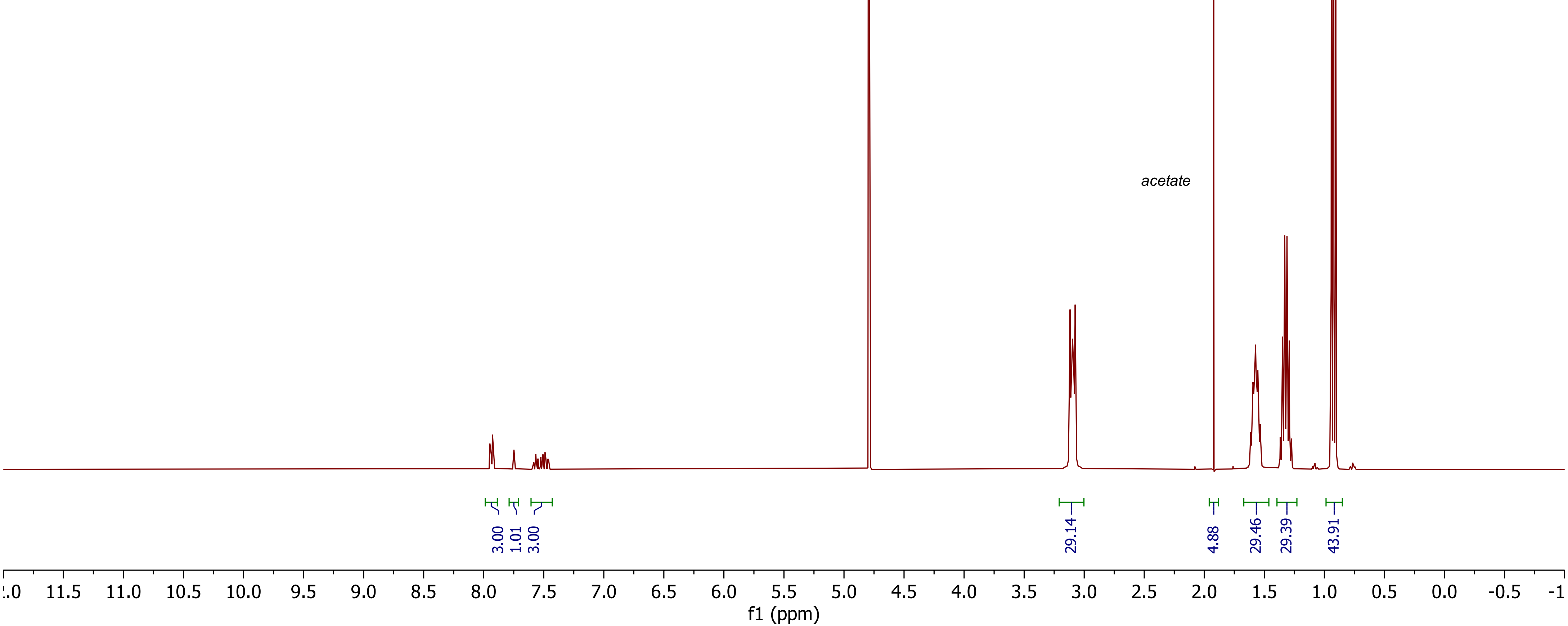

Compound 33, 2-naphthalen-2-yl diphosphate, $^{31}P\{^1H\}$ - NMR (D_2O, 122 MHz)

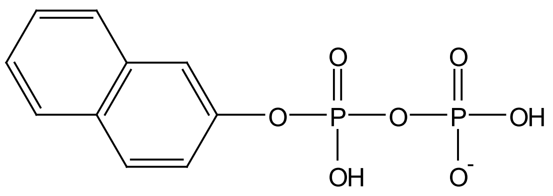

TBA salt

33

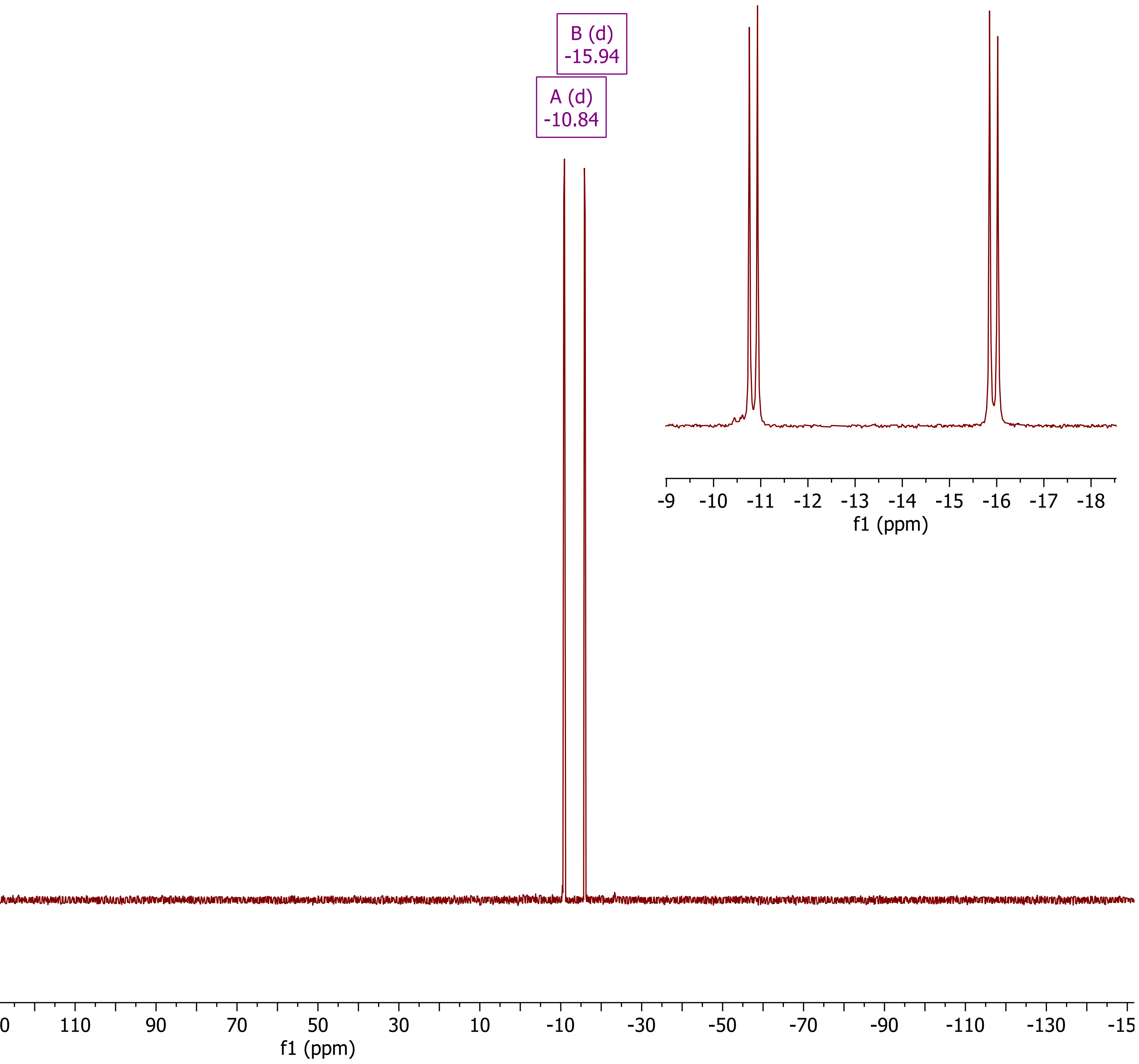

Compound 33, 2-naphthalen-2-yl diphosphate, ^{13}C - NMR (D_2O, 101 MHz)

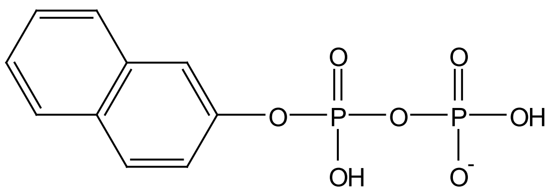

TBA salt

33

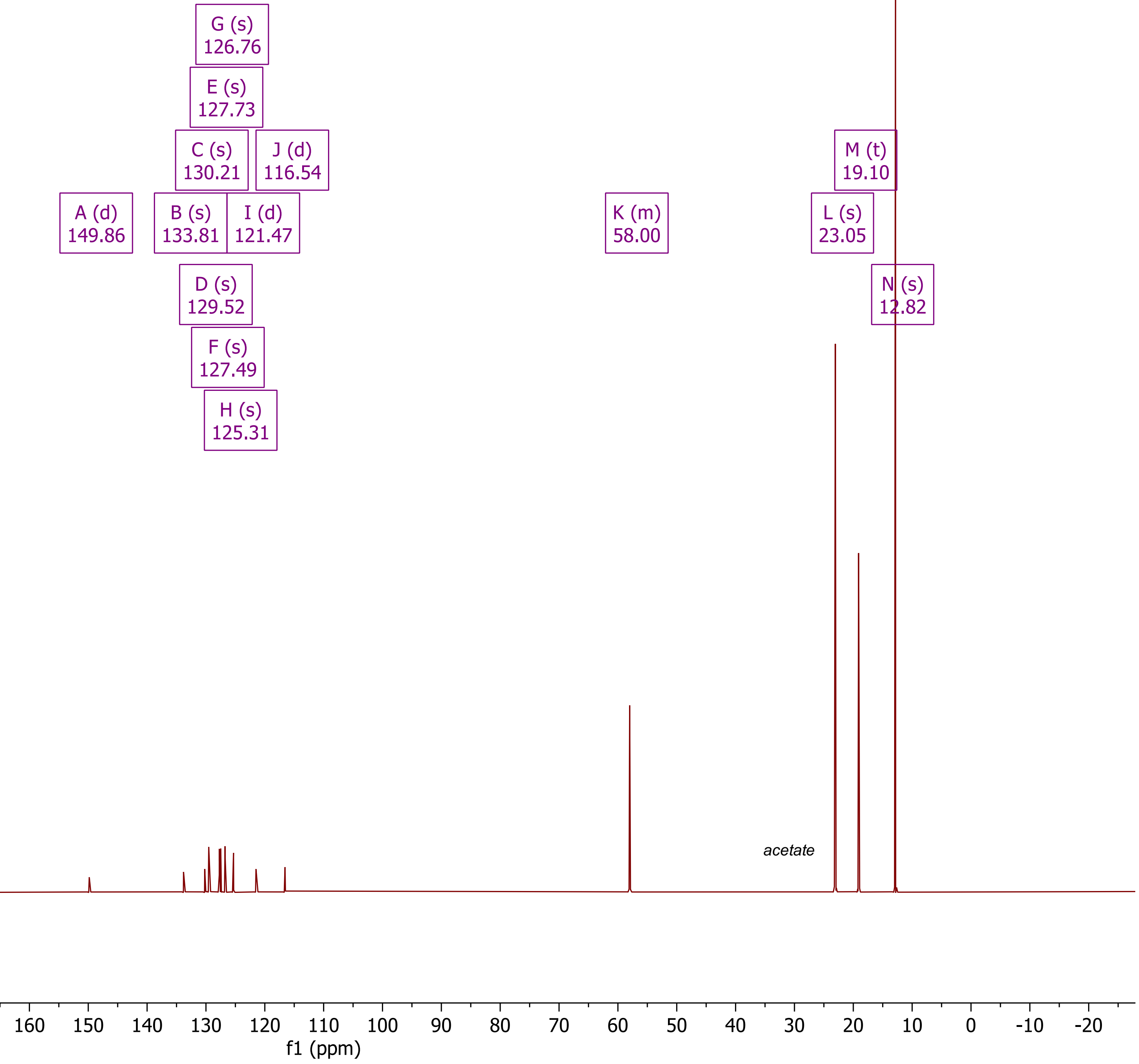

Compound 34, 3,4-dimethylphenyl diphosphate, ¹H - NMR (D_2O, 400 MHz)

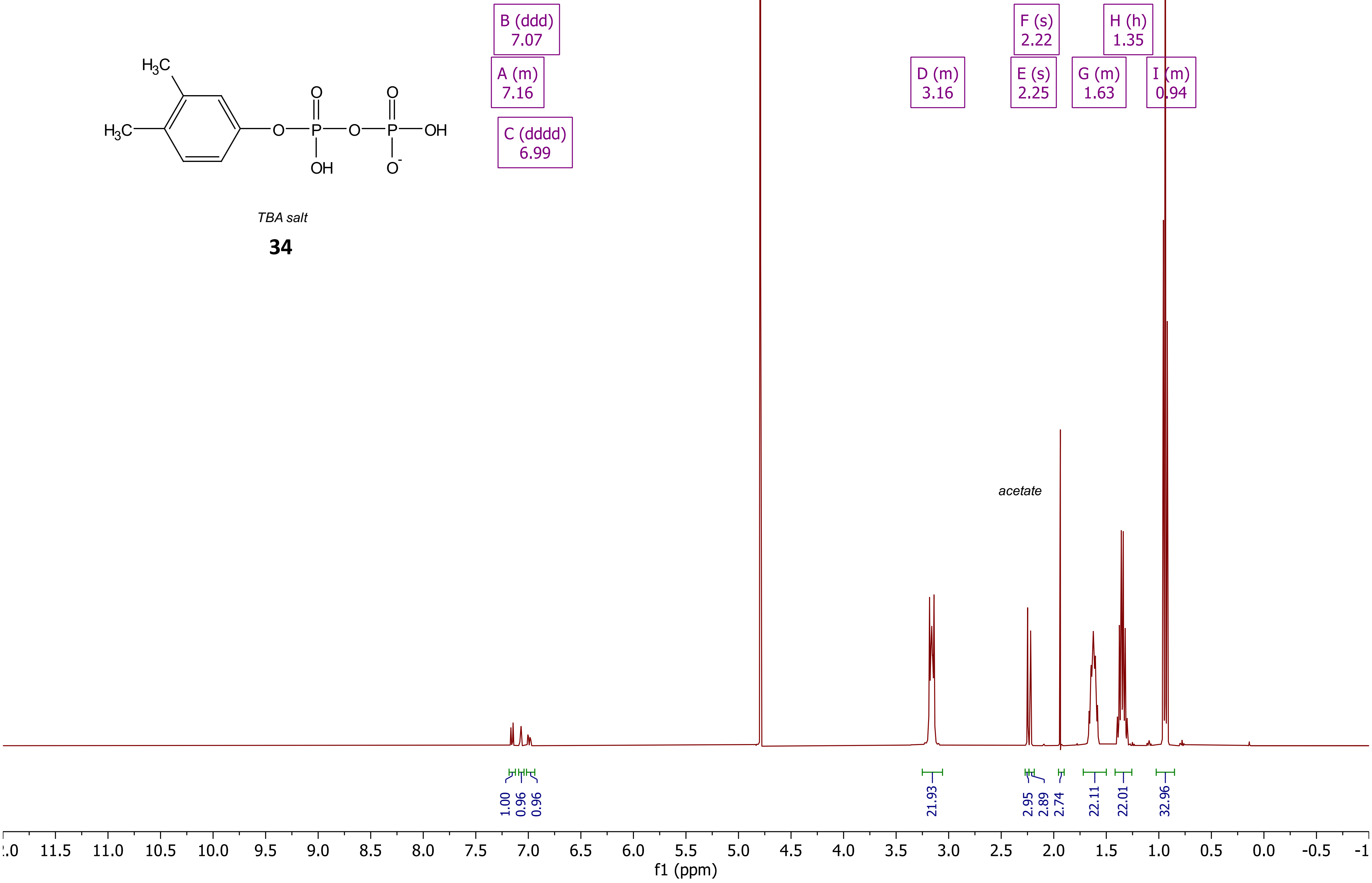

Compound 34, 3,4-dimethylphenyl diphosphate, $^{31}P\{^1H\}$ - NMR (D_2O, 162 MHz)

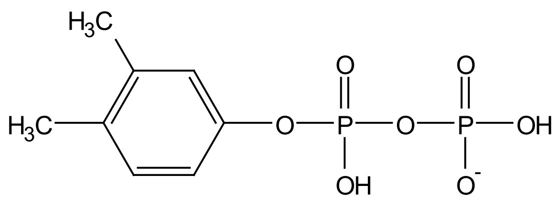

TBA salt

34

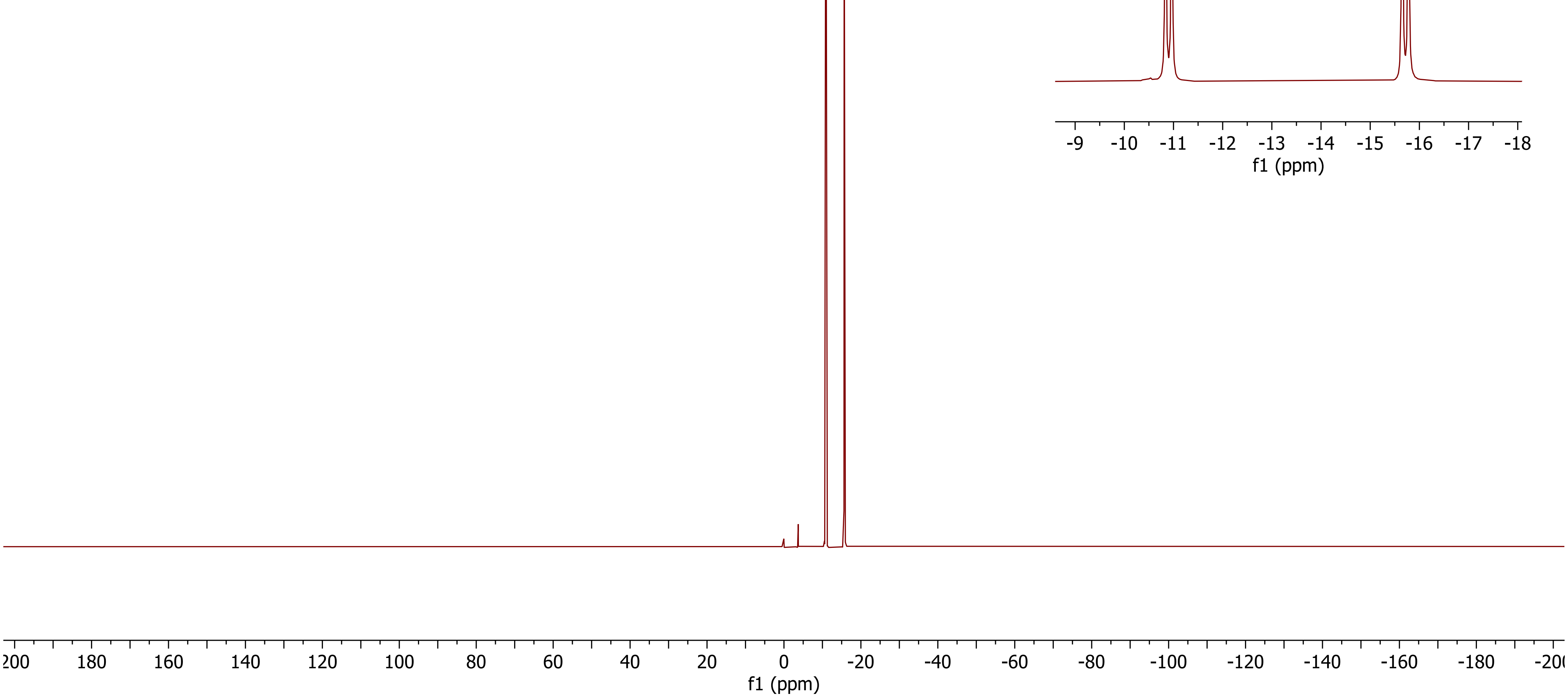

Compound 34, 3,4-dimethylphenyl diphosphate, ^{13}C - NMR (D_2O, 101 MHz)

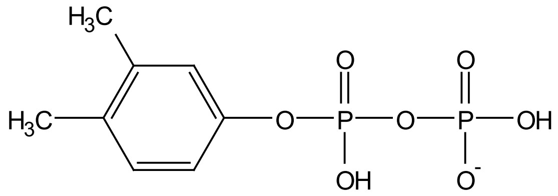

TBA salt

34

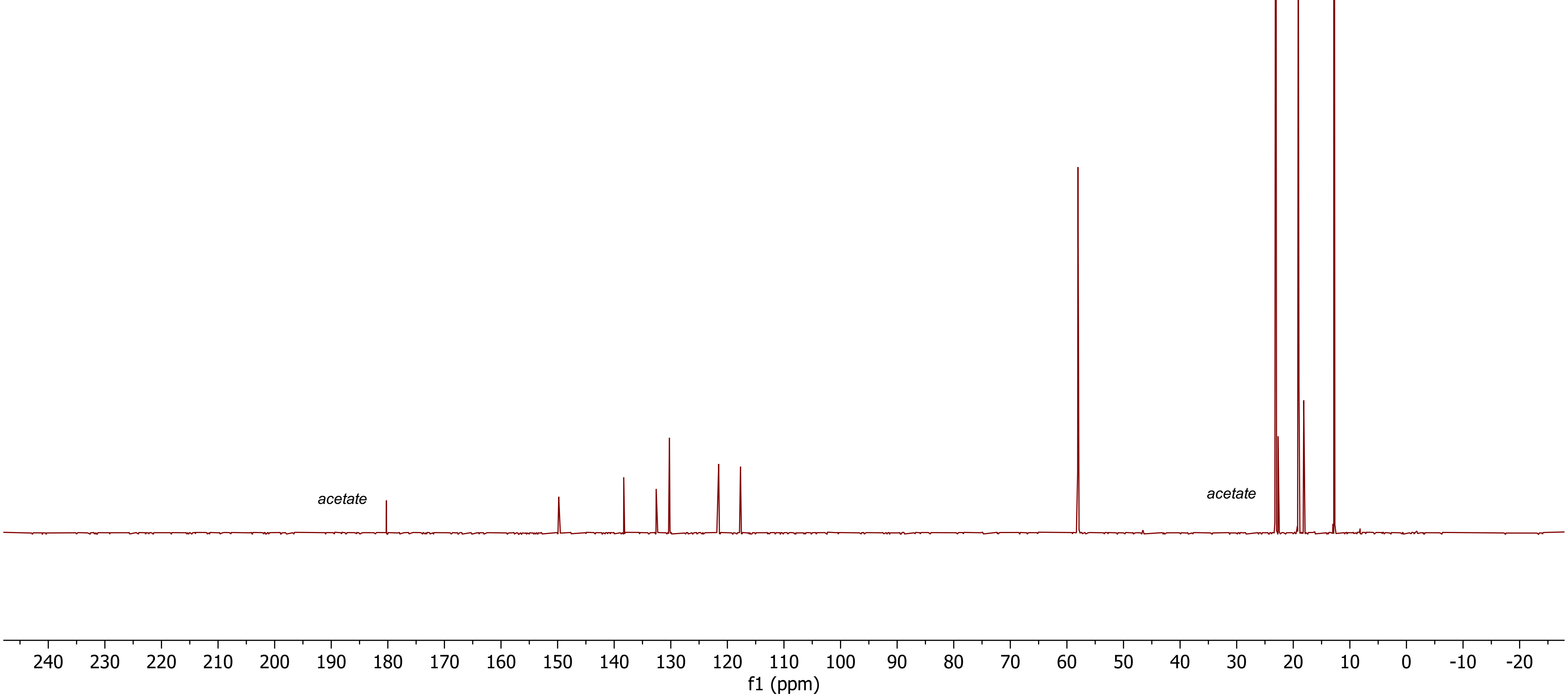

Compound 35, 1H-indol-5-yl diphosphate, ¹H - NMR (D_2O, 300 MHz)

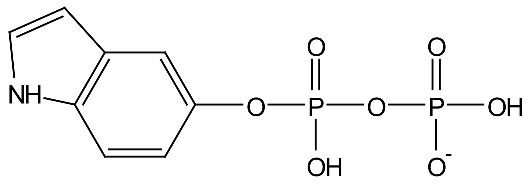

TBA/ TEA salt

35

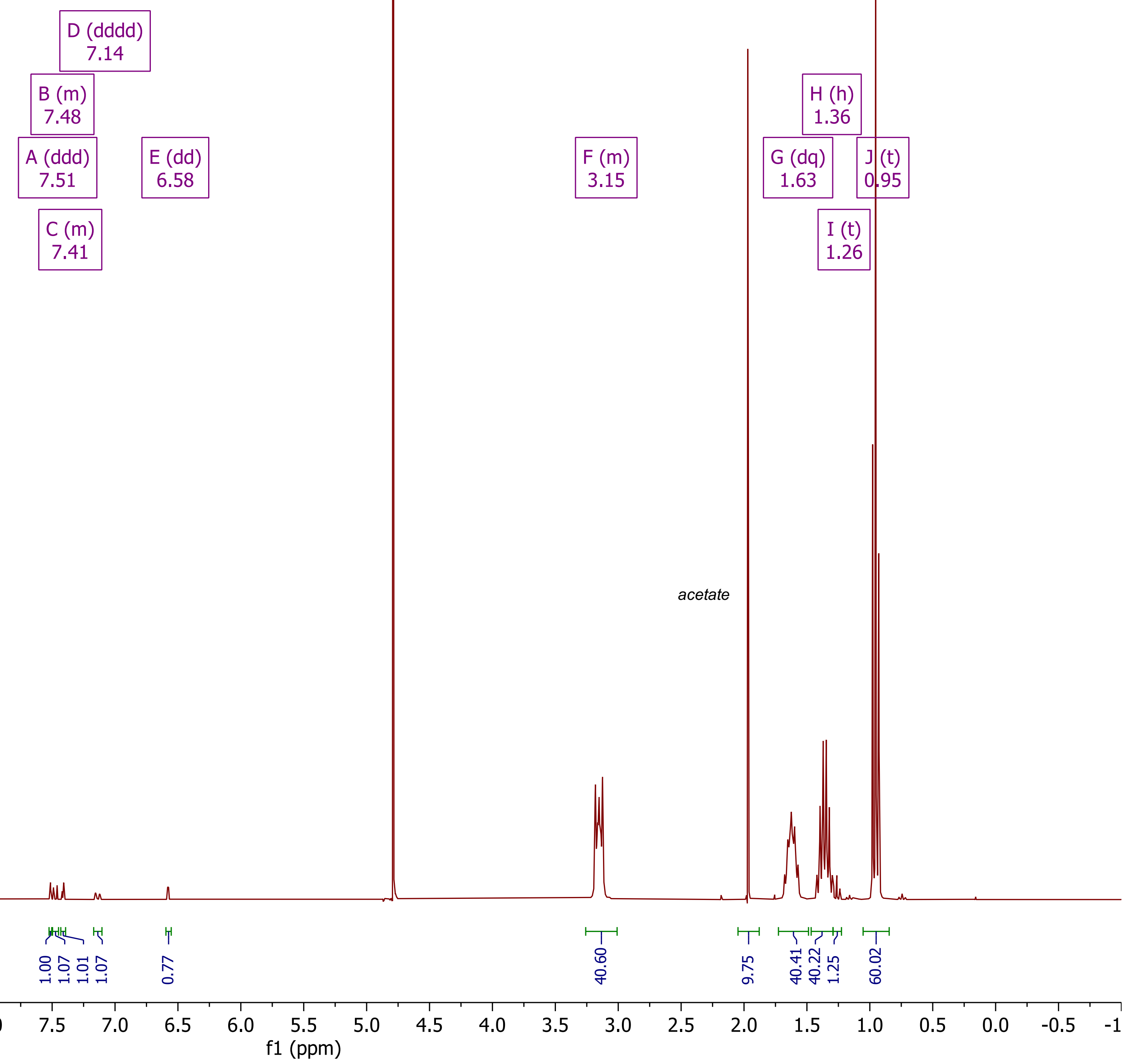

Compound 35, 1H-indol-5-yl diphosphate, $^{31}P\{^{1}H\}$ - NMR (D_2O, 162 MHz)

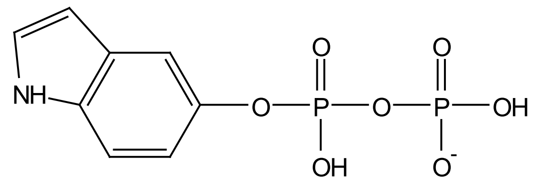

TBA/ TEA salt

35

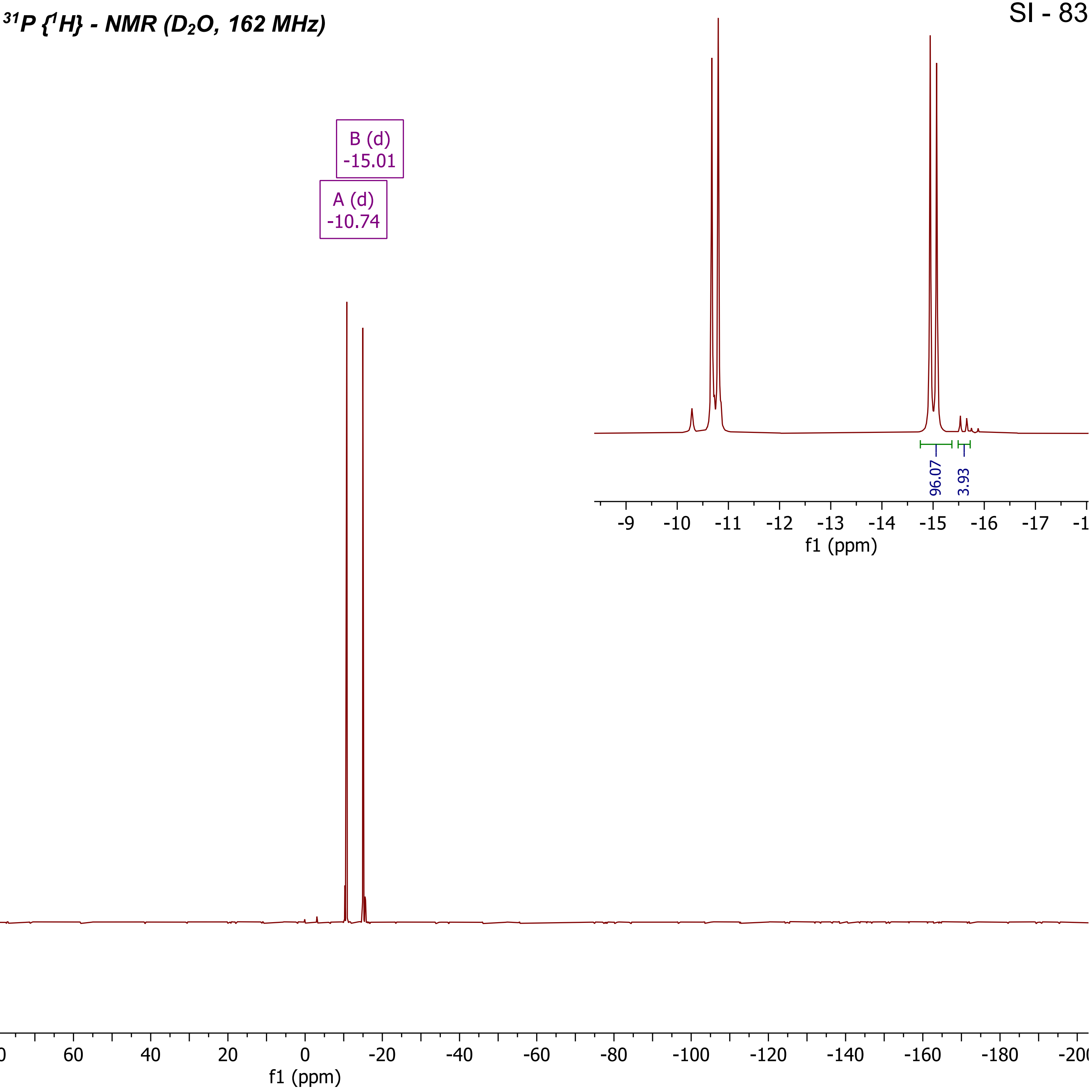

Compound 35, 1H-indol-5-yl diphosphate, ^{13}C - NMR (D$_2$O, 101 MHz)

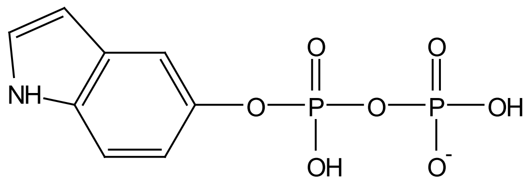

TBA/ TEA salt

35

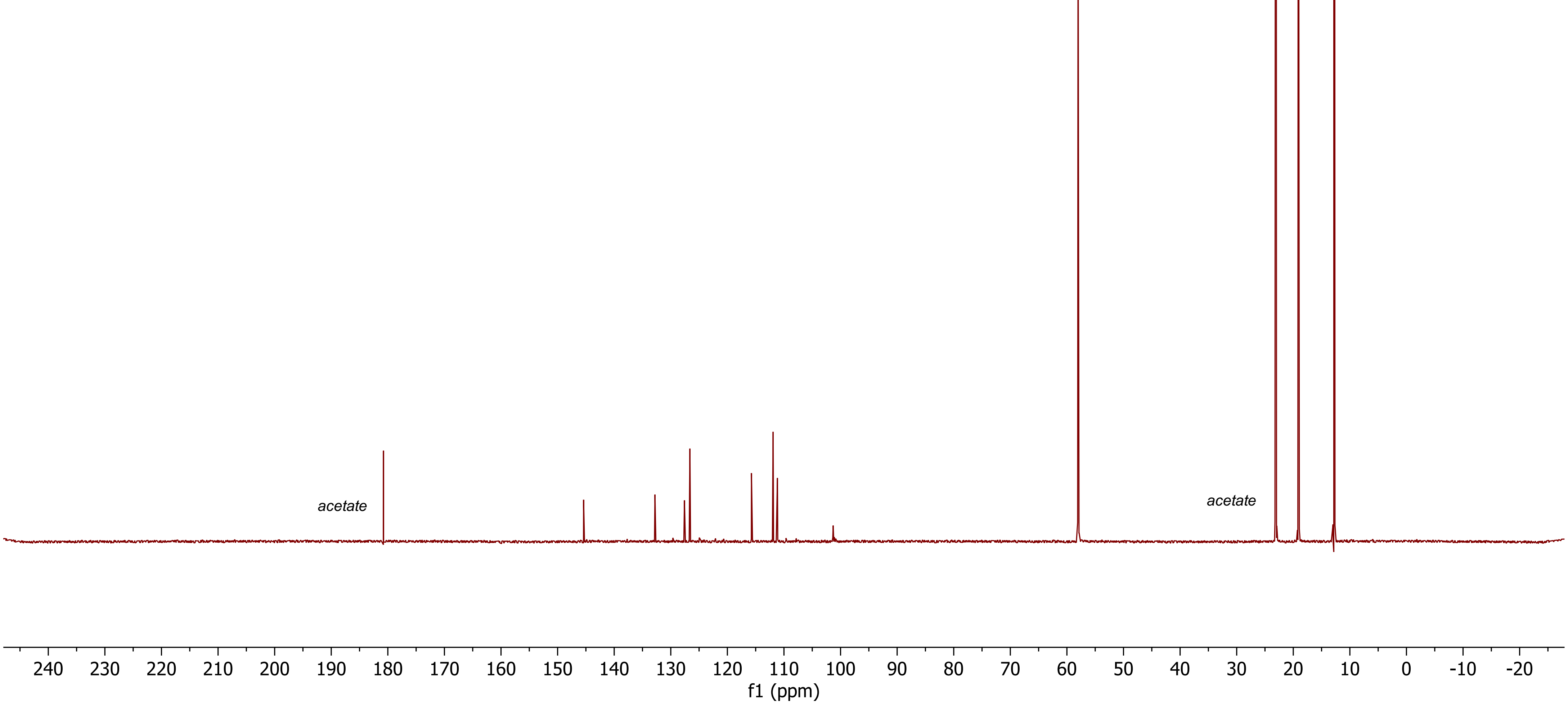

Compound 36, pyren-2-yl diphosphate, ^{1}H - NMR (D_2O, 400 MHz)

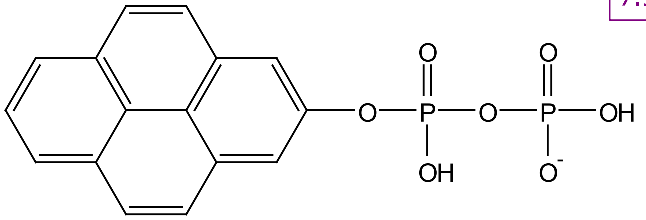

TBA salt

36

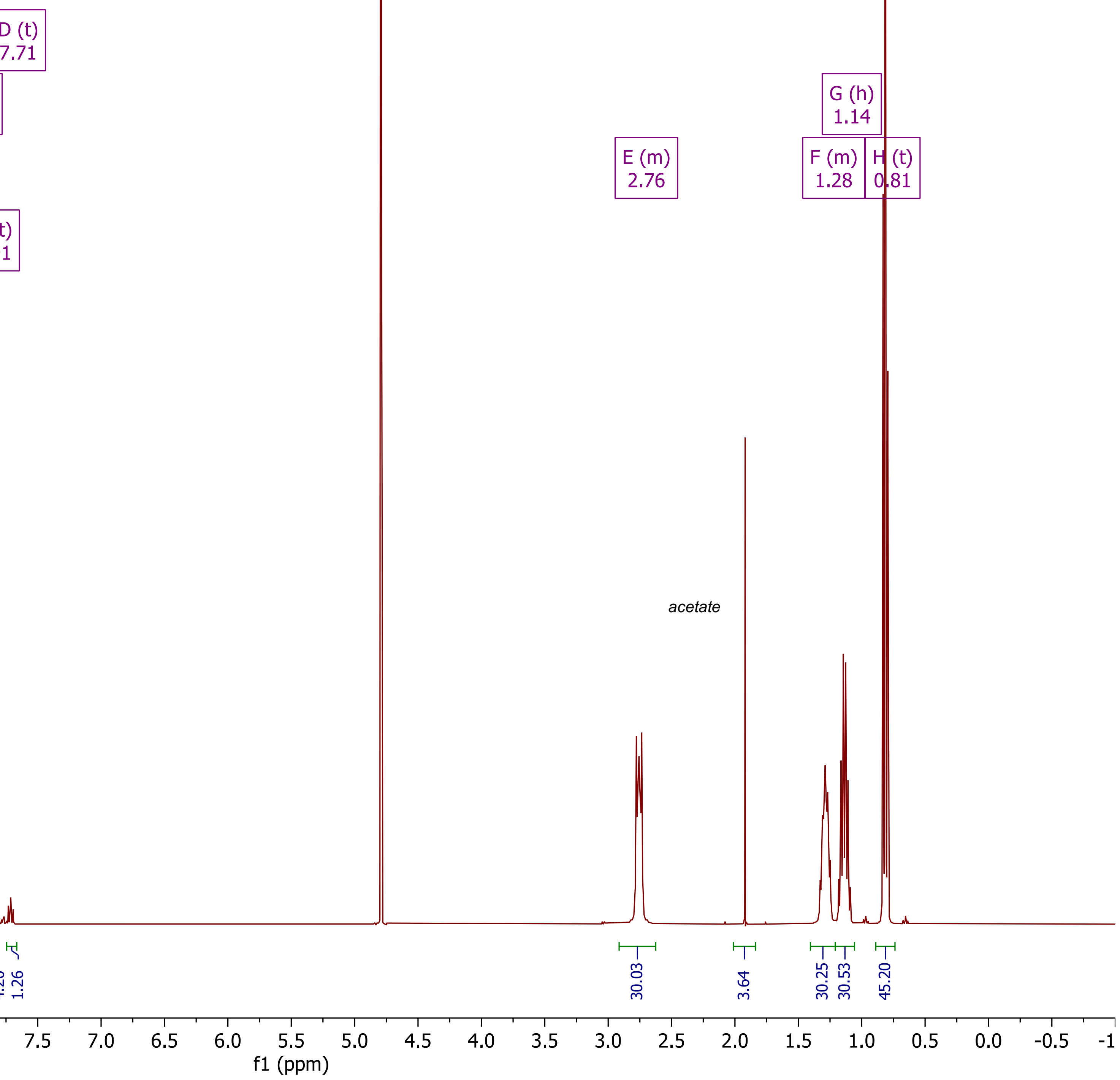

Compound 36, pyren-2-yl diphosphate, $^{31}P\{^1H\}$ - NMR (D_2O, 162 MHz)

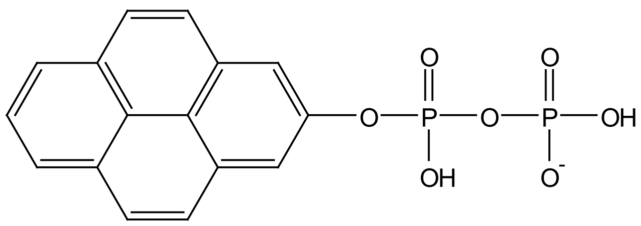

TBA salt

36

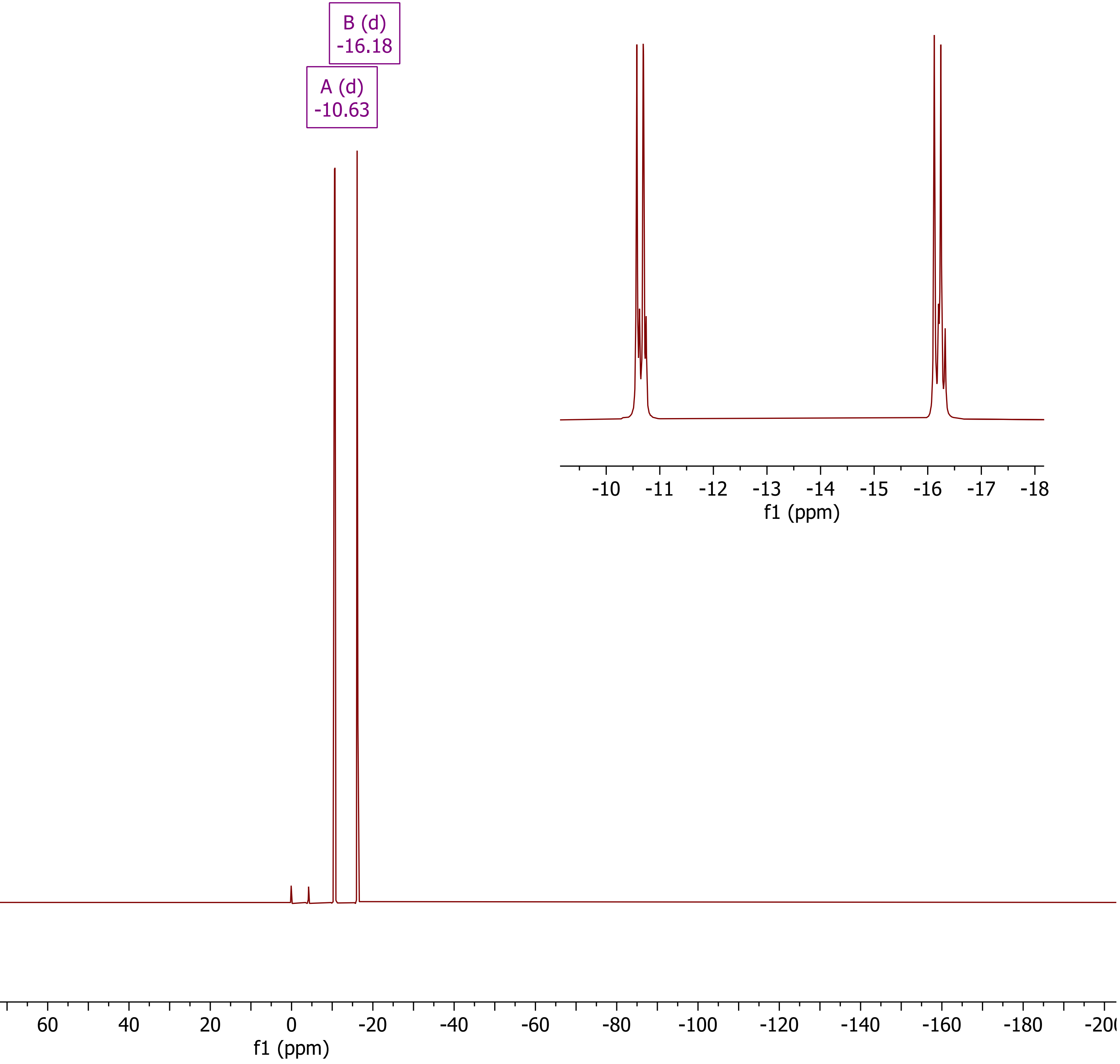

Compound 36, pyren-2-yl diphosphate, ^{13}C - NMR (D_2O, 101 MHz)

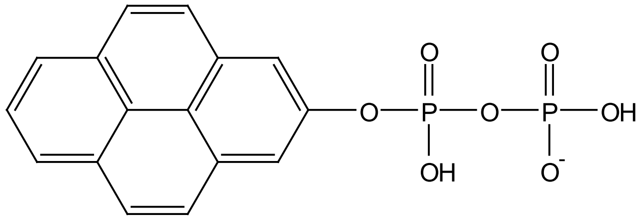

TBA salt

36

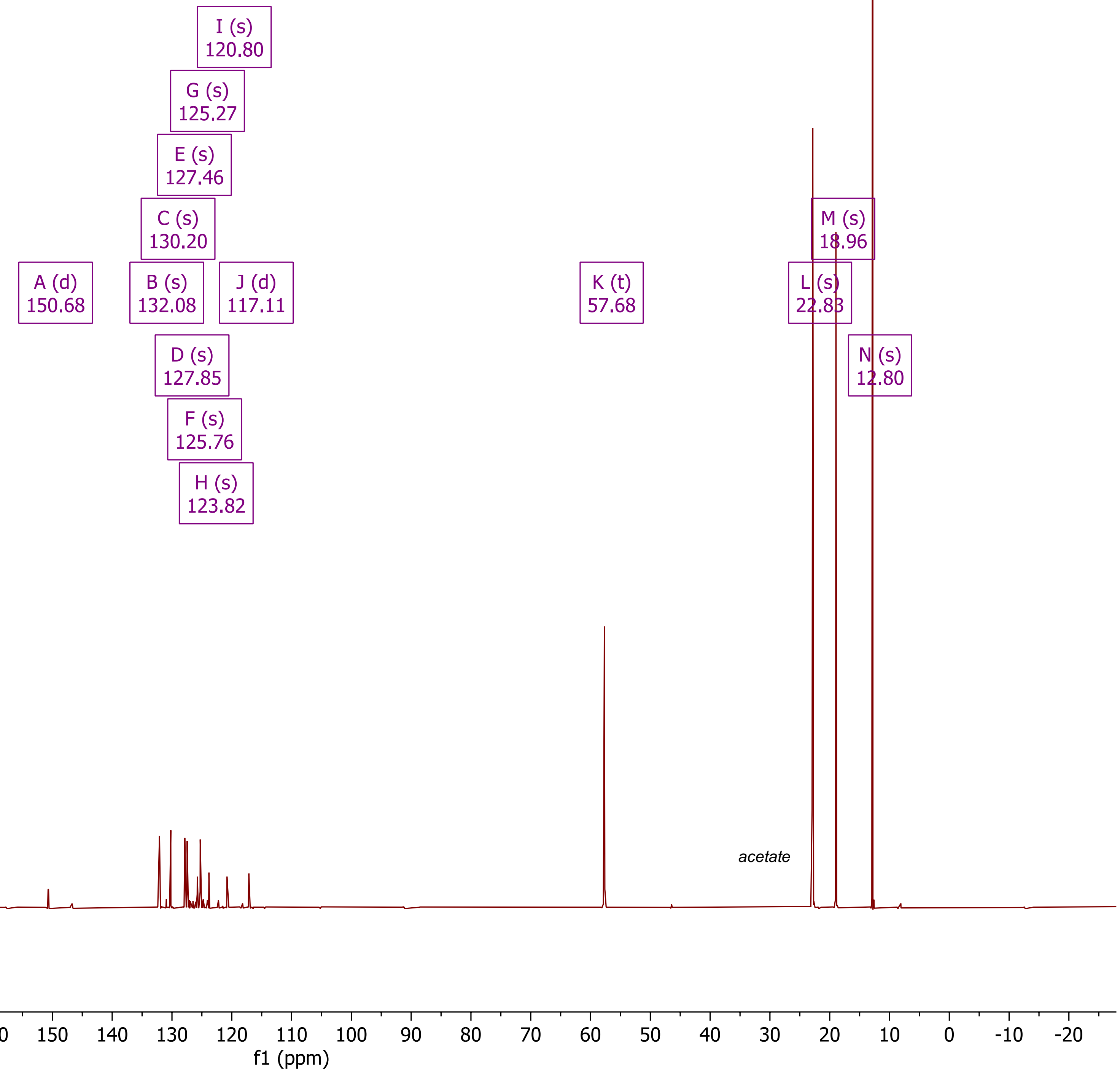

Compound 38, 5,5,5-Trifluoropentylphosphate, ^1H - NMR (D_2O, 400 MHz)

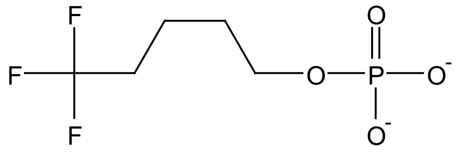

TBA/TEA - salt

38

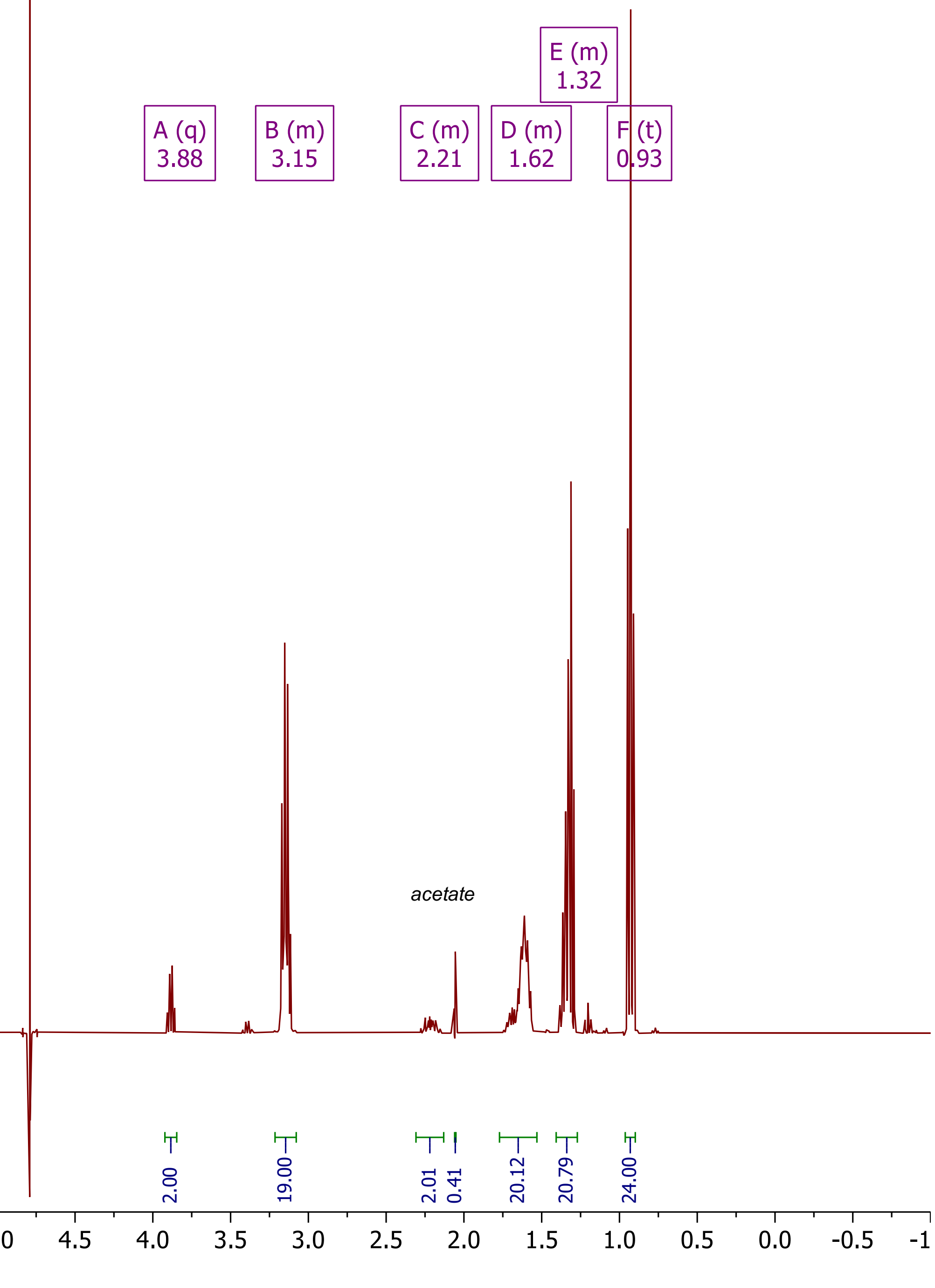

Compound 38, 5,5,5-Trifluoropentylphosphate, ^{19}F - NMR (D2O, 377 MHz)

TBA/TEA - salt

38

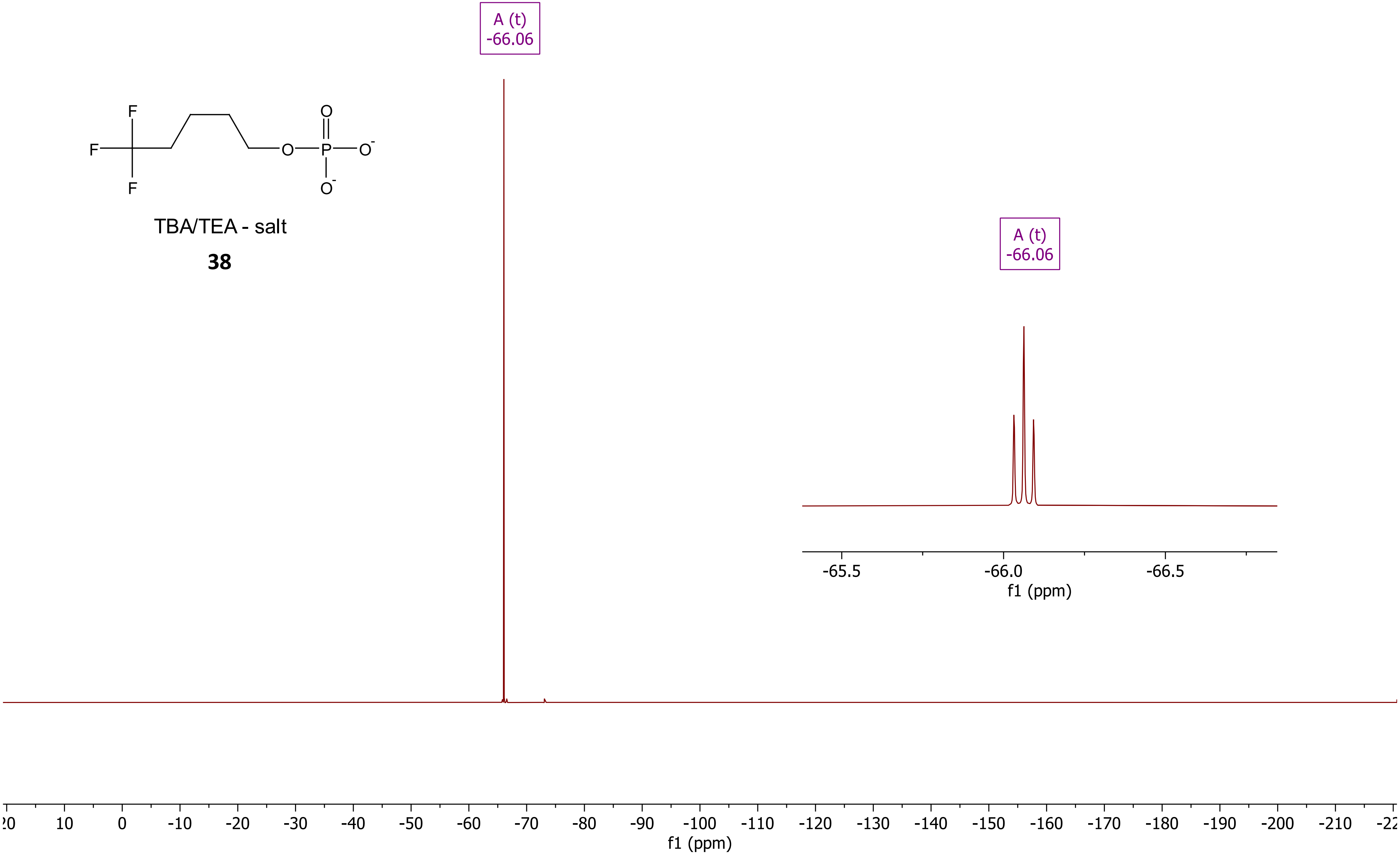

Compound 38, 5,5,5-Trifluoropentylphosphate, $^{31}P\{^{1}H\}$ - NMR (D2O, 162 MHz)

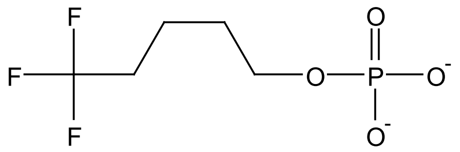

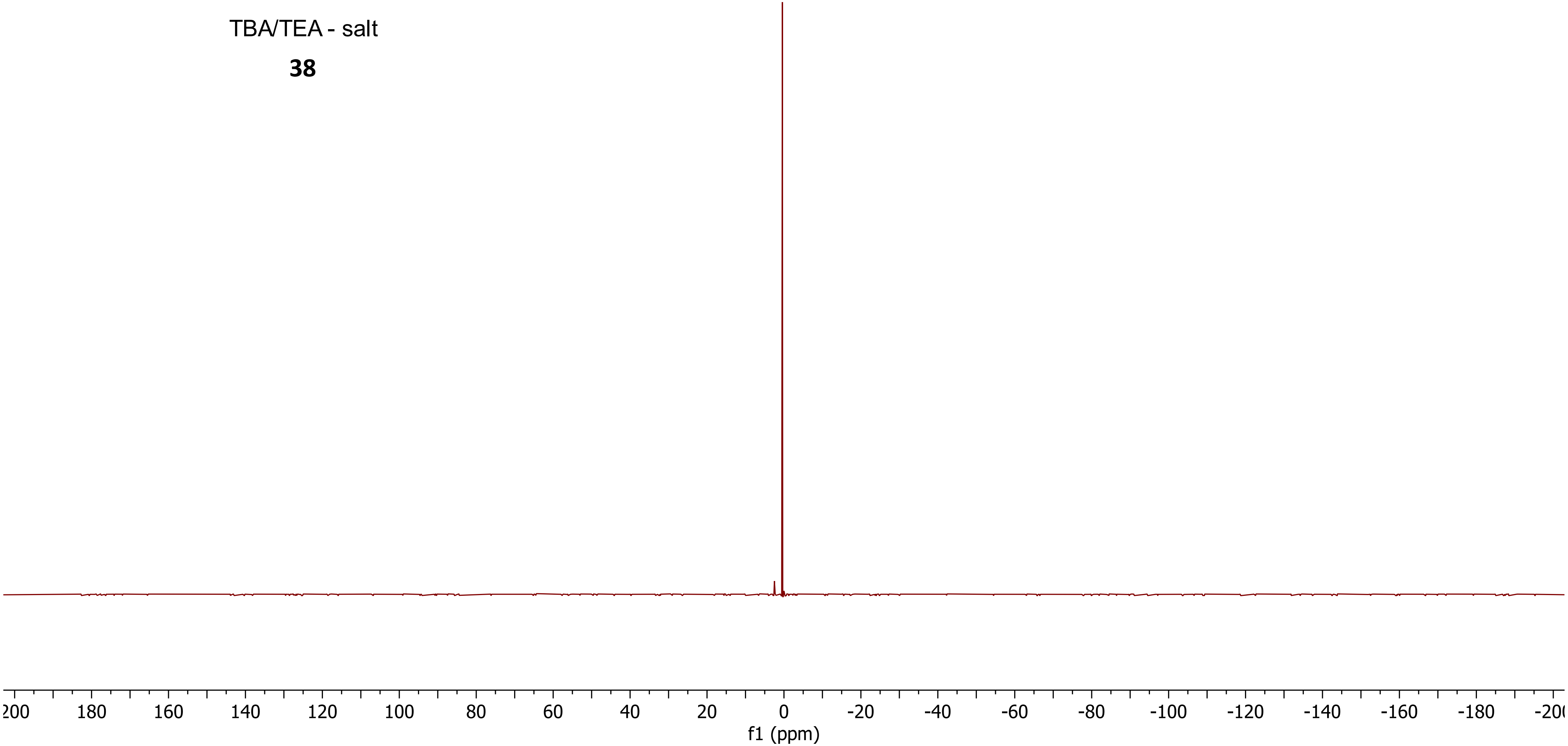

Compound 38, 5,5,5-Trifluoropentylphosphate, $^{13}C\{^{1}H\}$ - NMR (D2O, 101 MHz)

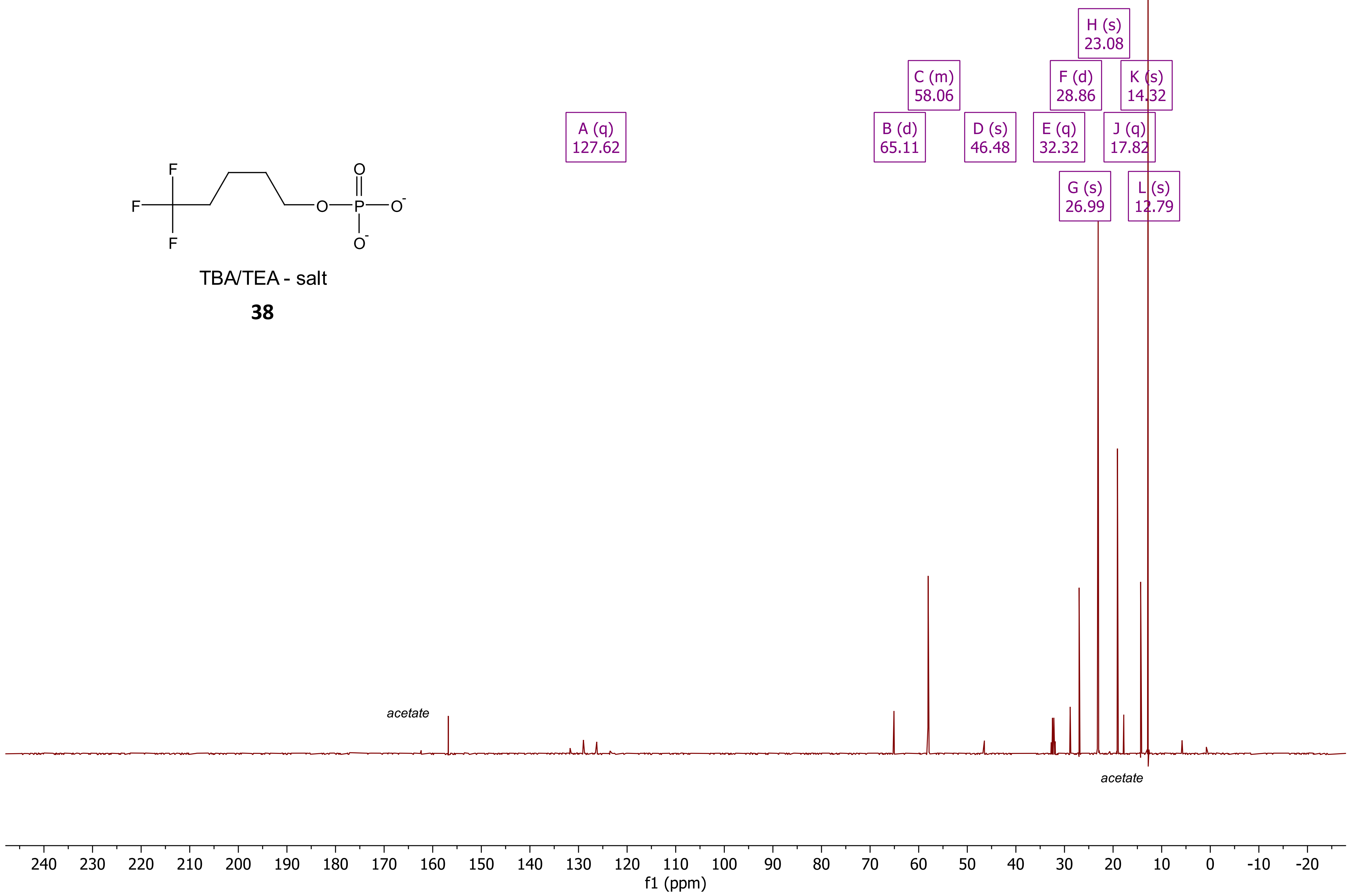

Compound 39, 5,5,5-Trifluoropentyl-phenylphosphate, ^{1}H - NMR (MeCN-d3, 400 MHz)

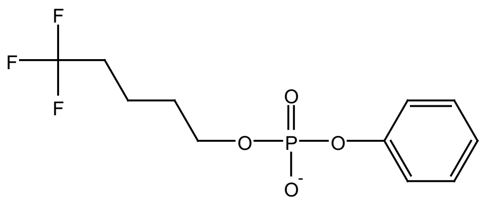

TBA / TEA - salt

39

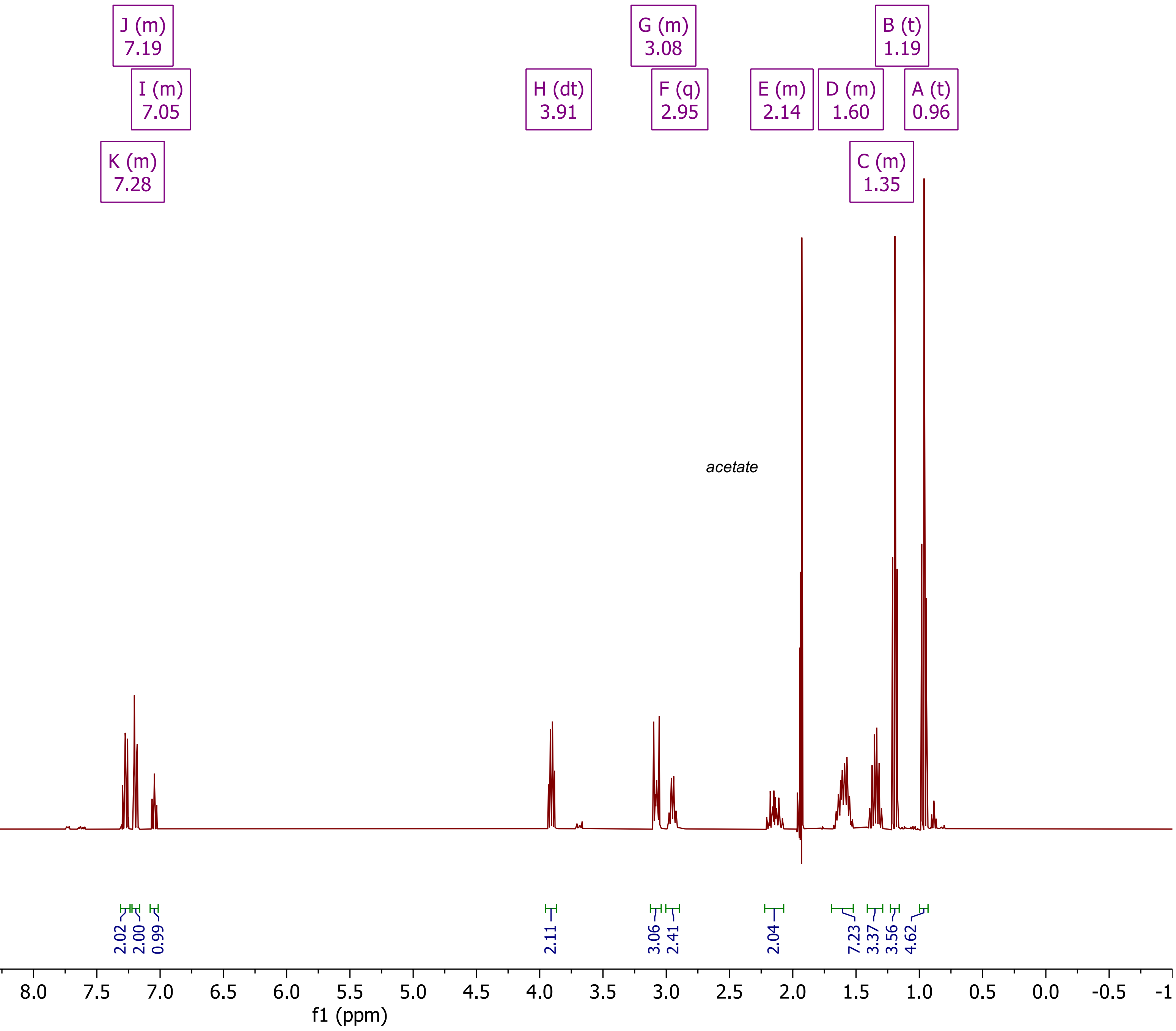

Compound 39, 5,5,5-Trifluoropentyl-phenylphosphate, ^{19}F - NMR (MeCN-d3, 377 MHz)

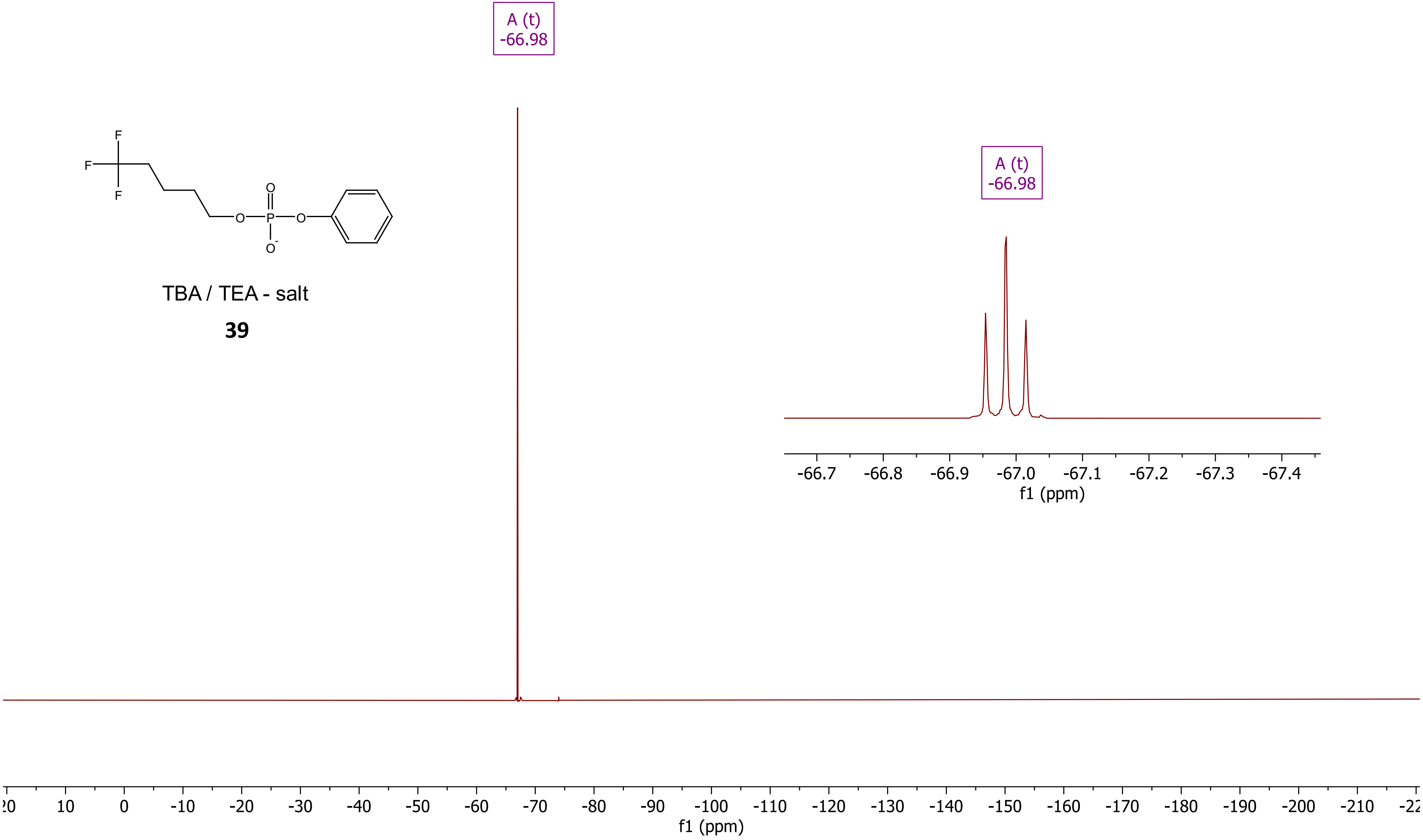

TBA / TEA - salt

39

Compound 39, 5,5,5-Trifluoropentyl-phenylphosphate, ^{31}P {^{1}H} - NMR (MeCN-d3, 162 MHz)

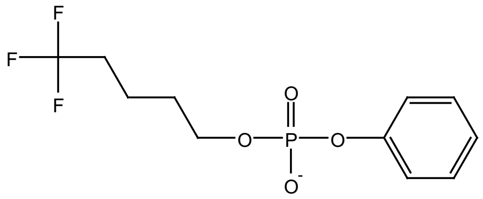

TBA / TEA - salt

39

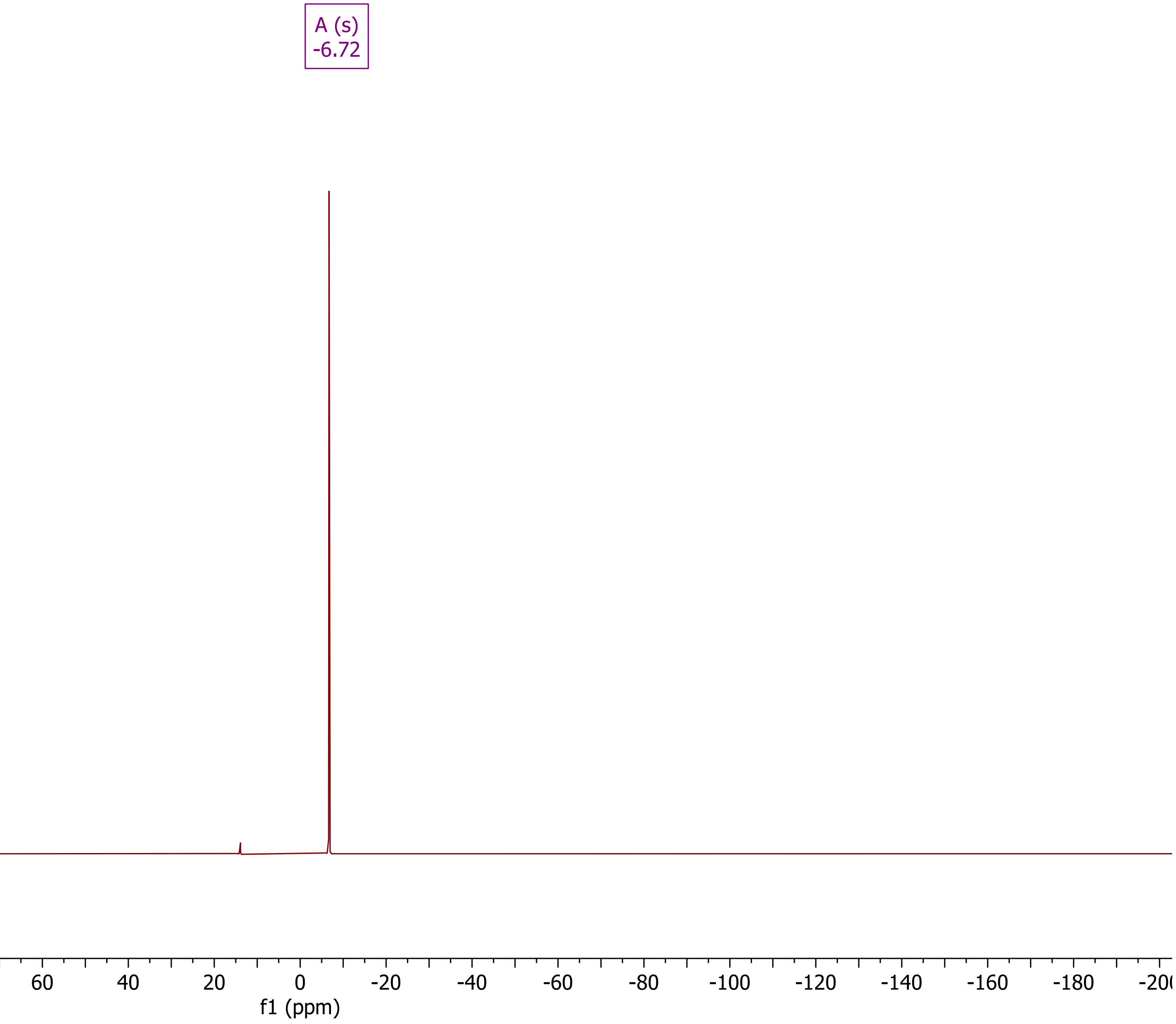

Compound 39, 5,5,5-Trifluoropentyl-phenylphosphate, $^{13}C\{^1H\}$ - NMR (MeCN-d3, 101 MHz)

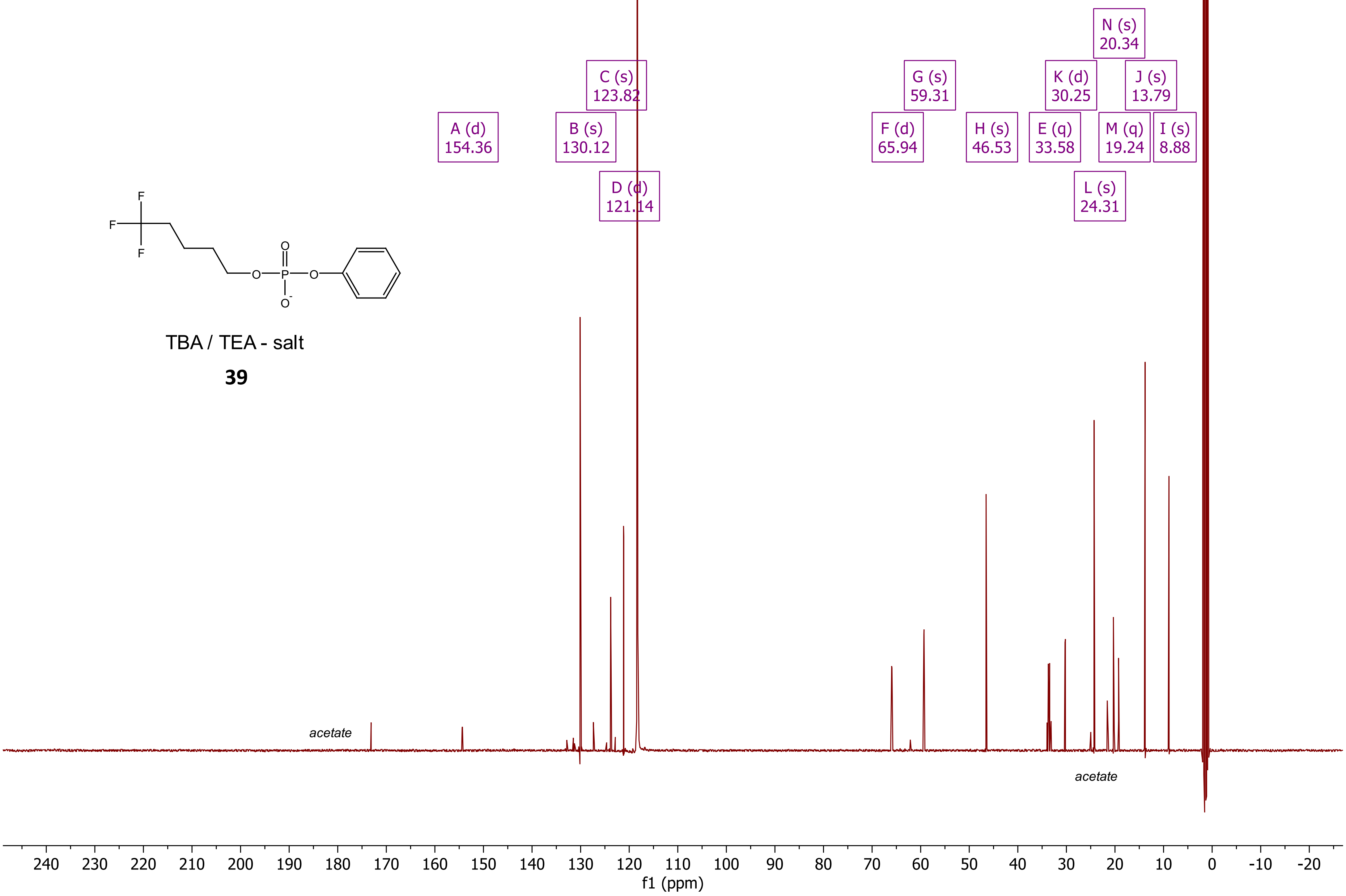

Compound 41, 3,4-dimethylphenyl (5,5,5-trifluoropentyl) phosphate, ^{1}H - NMR (CD_3CN, 400 MHz)

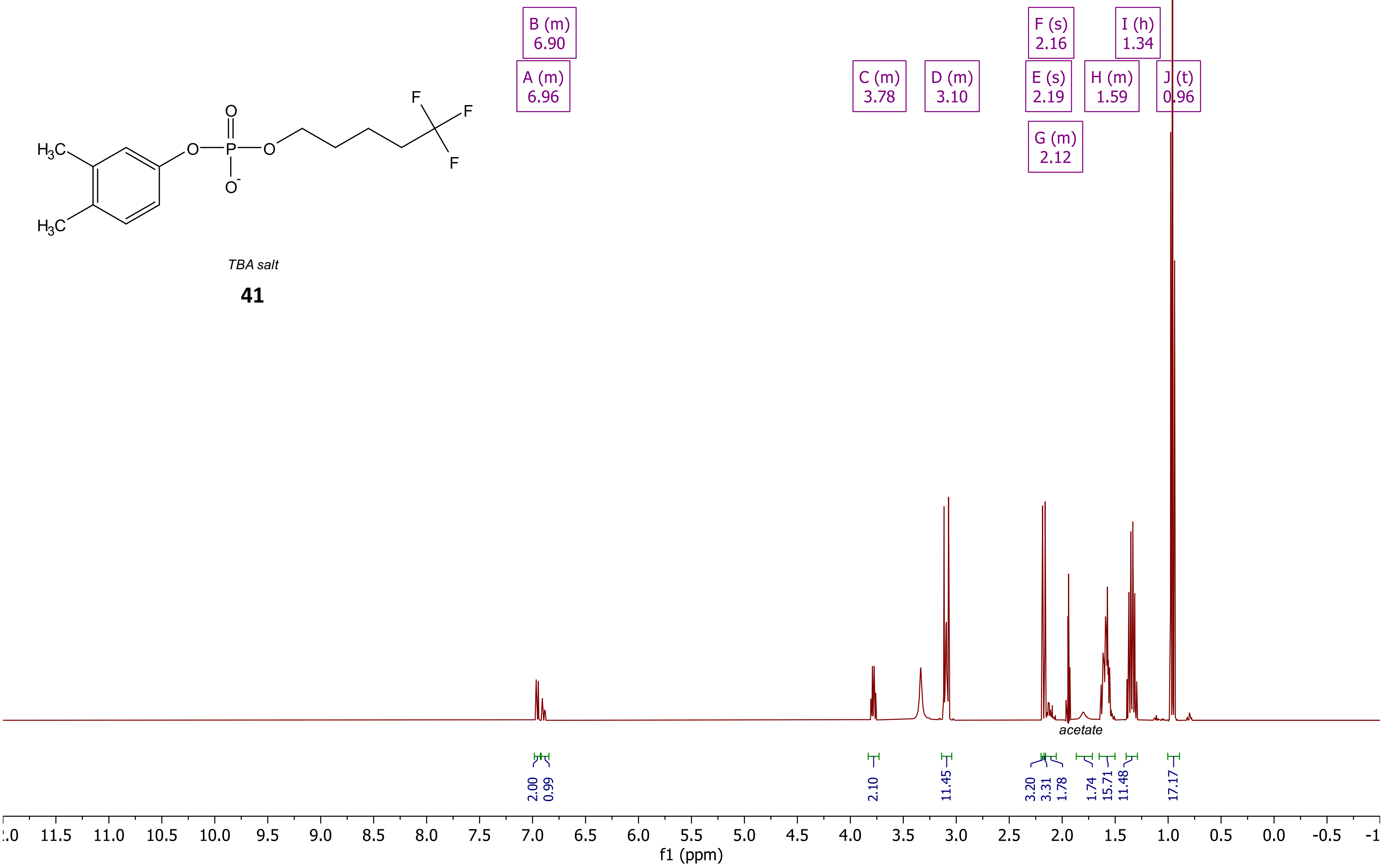

TBA salt

41

Compound 41, 3,4-dimethylphenyl (5,5,5-trifluoropentyl) phosphate, ^{19}F - NMR (CD_3CN, 377 MHz)

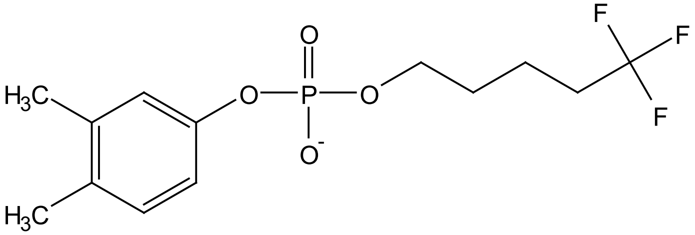

TBA salt

41

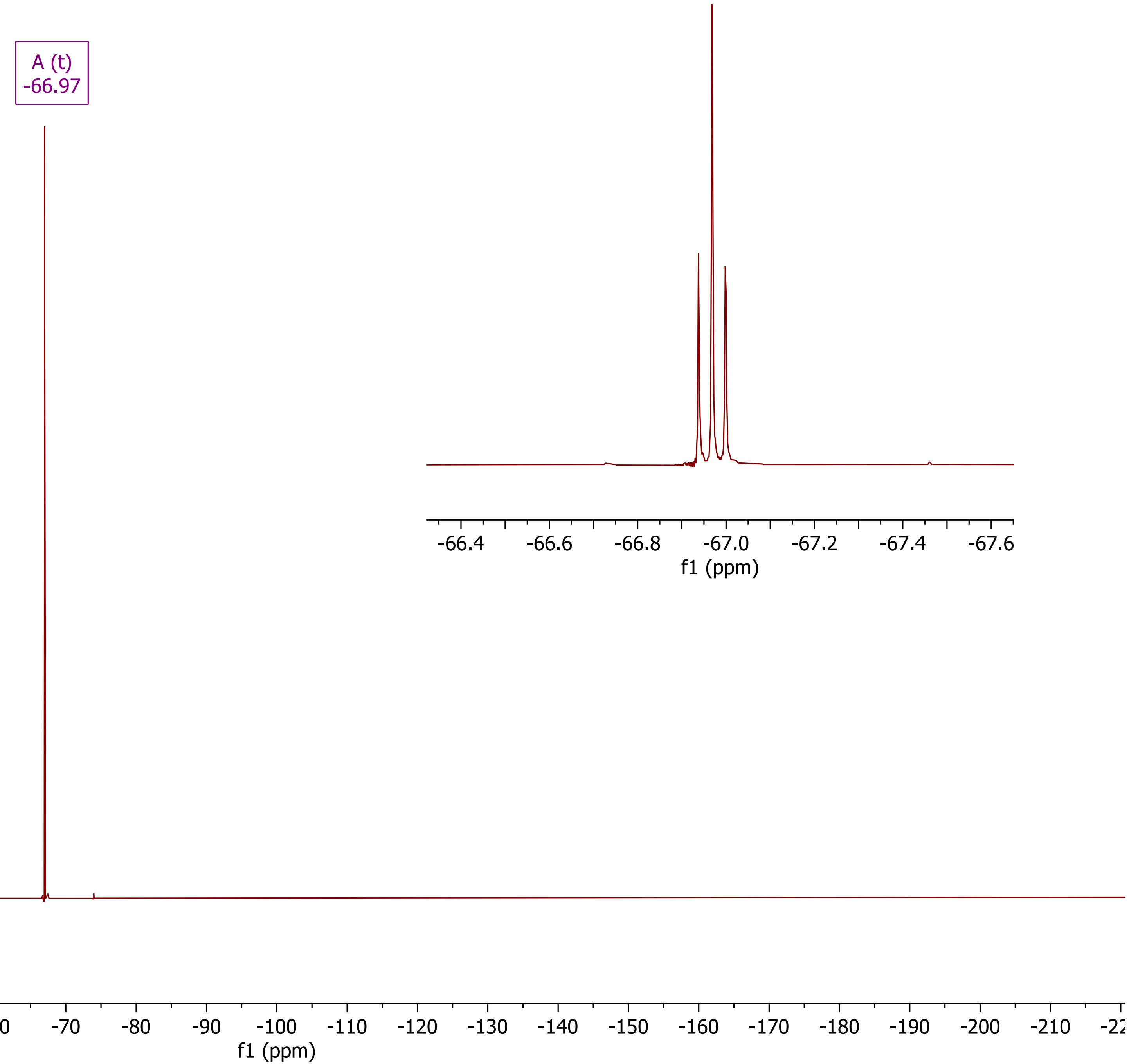

Compound 41, 3,4-dimethylphenyl (5,5,5-trifluoropentyl) phosphate, $^{31}P\{^{1}H\}$ - NMR (CD_3CN, 162 MHz)

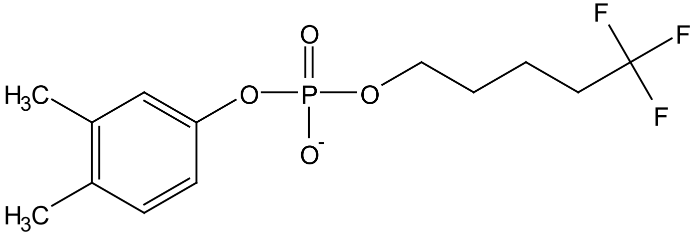

TBA salt

41

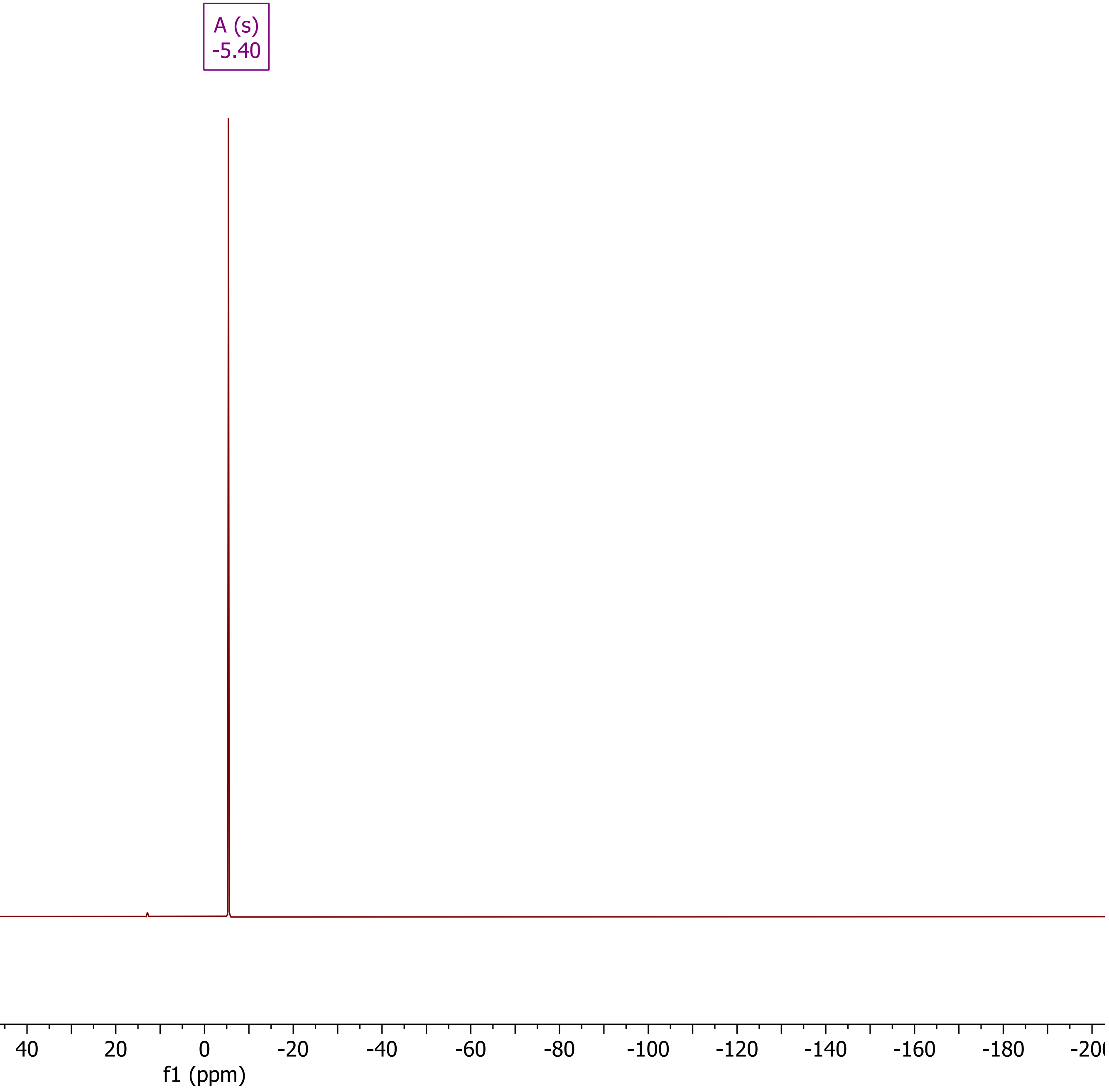

Compound 41, 3,4-dimethylphenyl (5,5,5-trifluoropentyl) phosphate, ^{13}C - NMR (CD_3CN, 101 MHz)

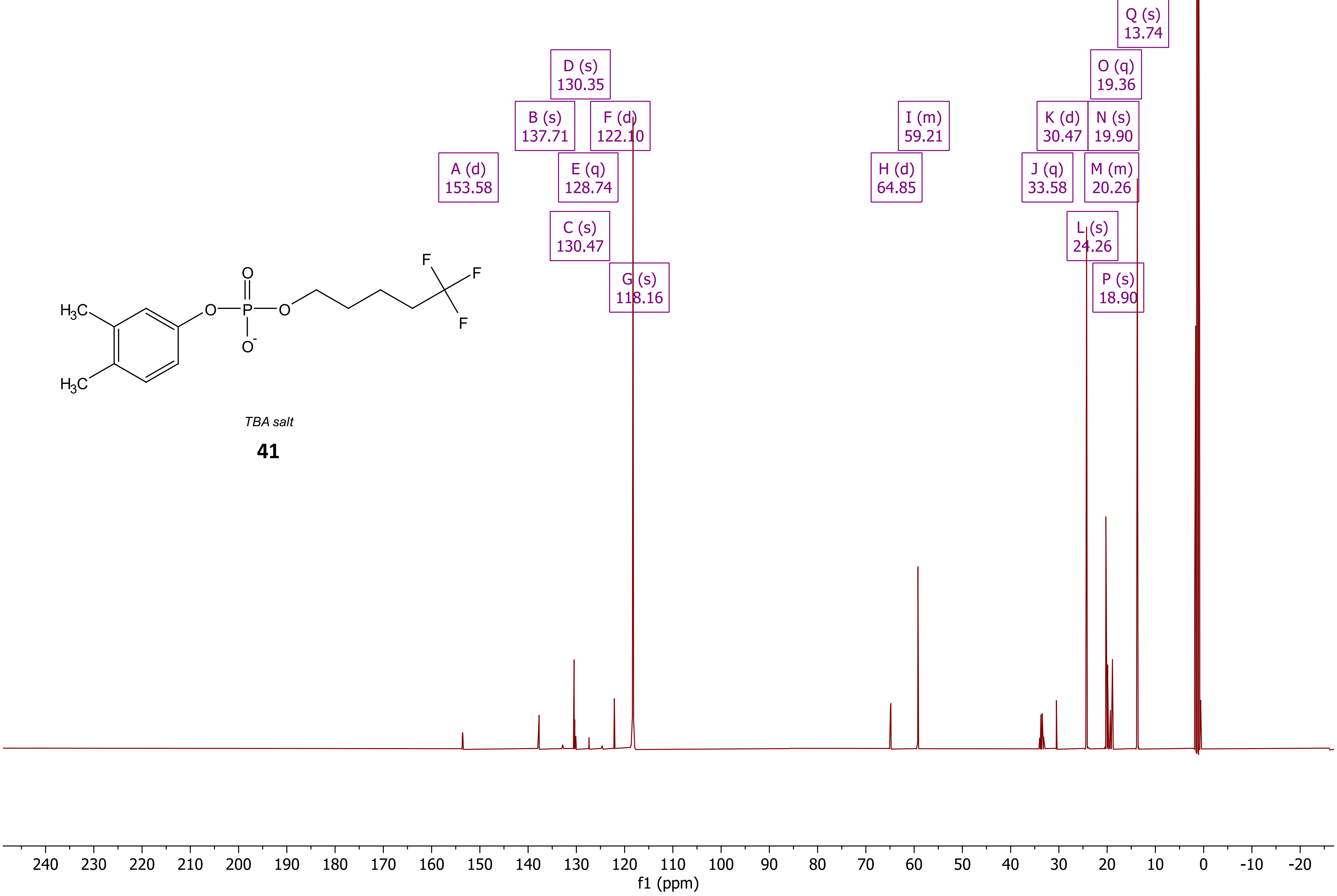

Compound 42, 2,5-dimethylphenyl (5,5,5-trifluoropentyl) phosphate, ^{1}H - NMR (CD_3CN, 400 MHz)

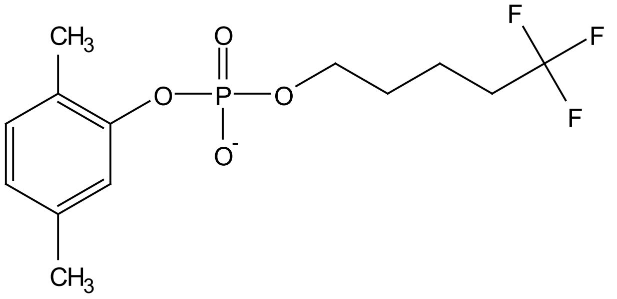

TBA salt

42

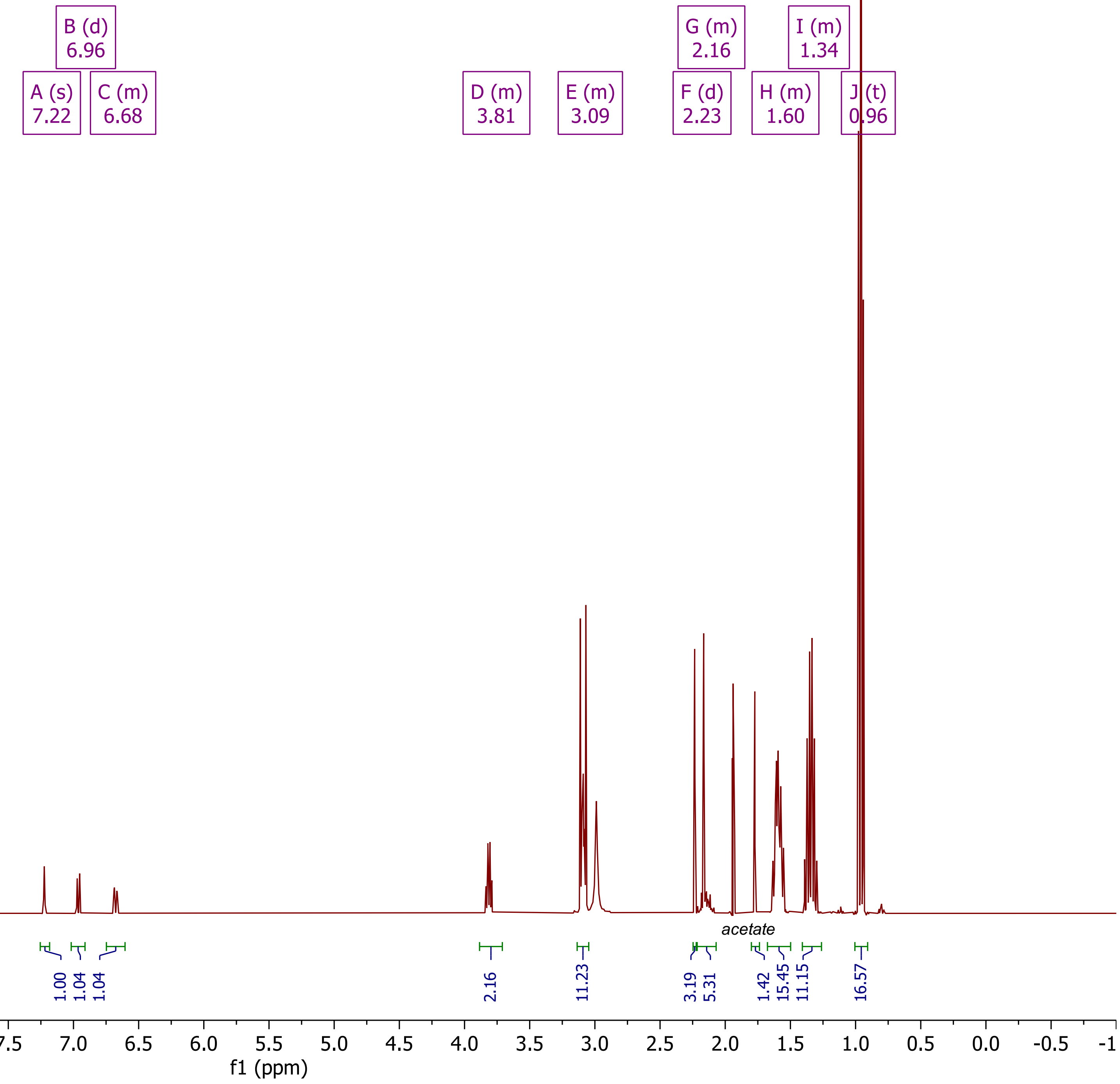

Compound 42, 2,5-dimethylphenyl (5,5,5-trifluoropentyl) phosphate, ^{19}F - NMR (CD_3CN, 377 MHz)

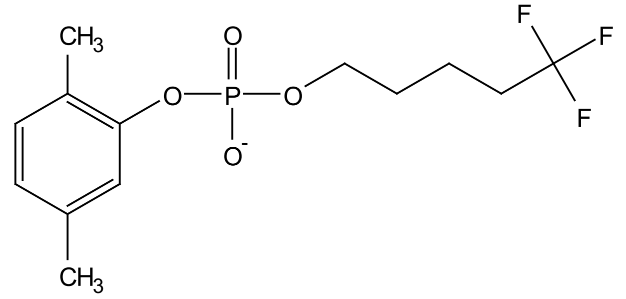

TBA salt

42

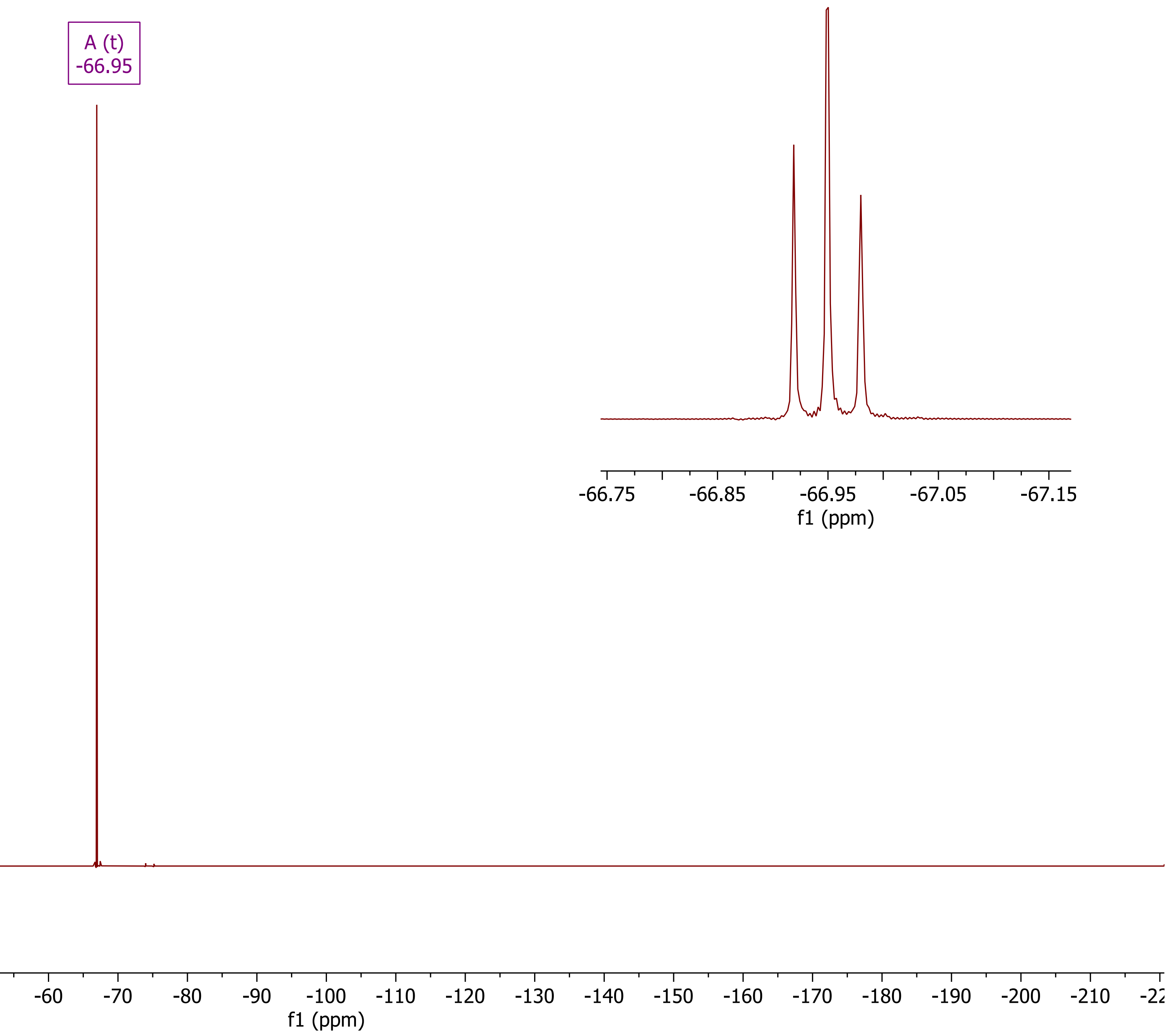

Compound 42, 2,5-dimethylphenyl (5,5,5-trifluoropentyl) phosphate, $^{31}P\ \{^1H\}$ - NMR (CD_3CN, 162 MHz)

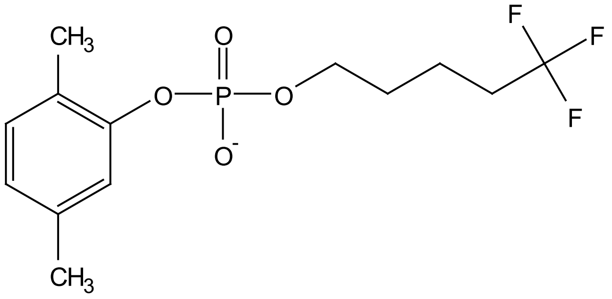

TBA salt

42

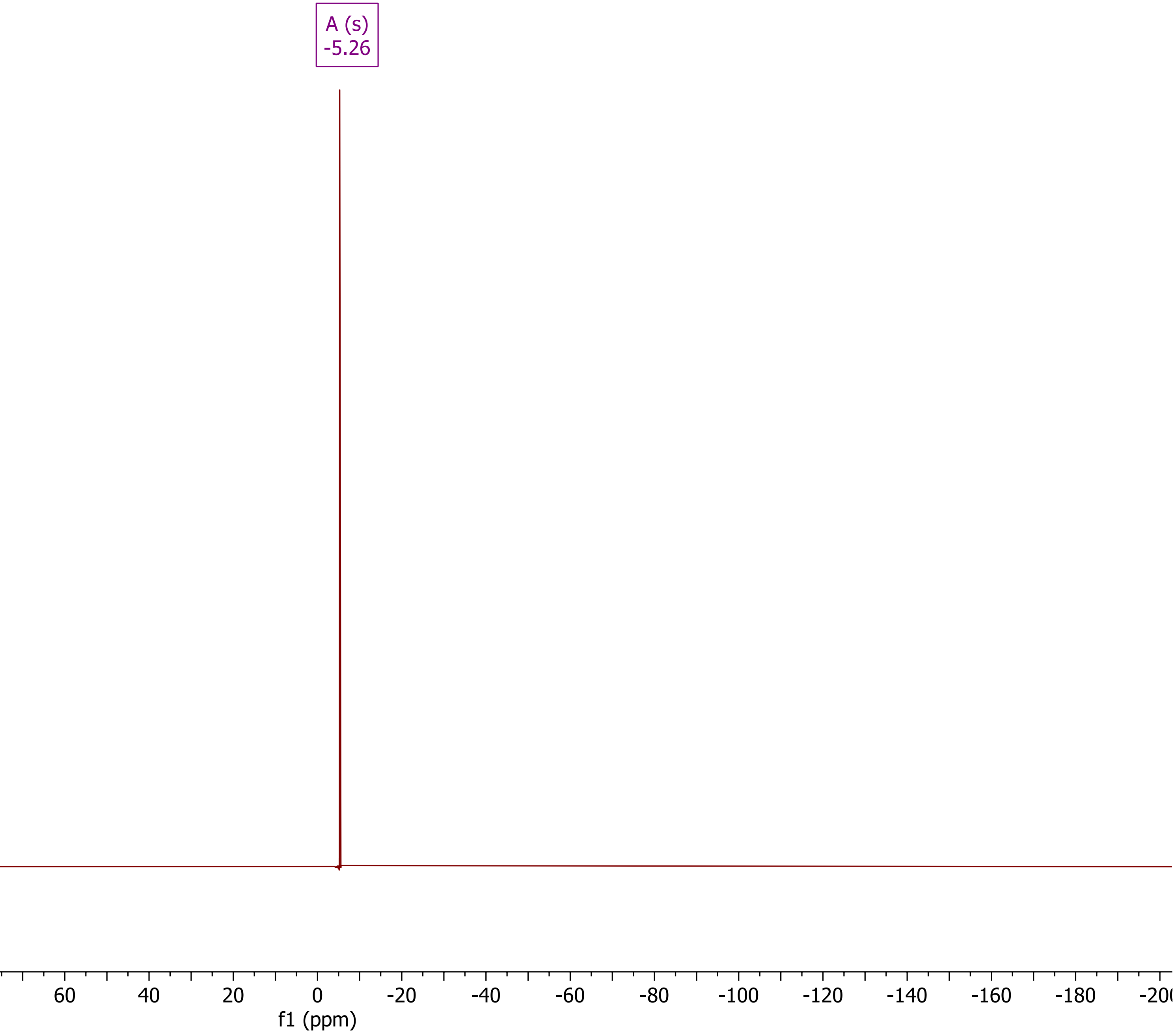

Compound 42, 2,5-dimethylphenyl (5,5,5-trifluoropentyl) phosphate, ^{13}C - NMR (CD_3CN, 101 MHz)

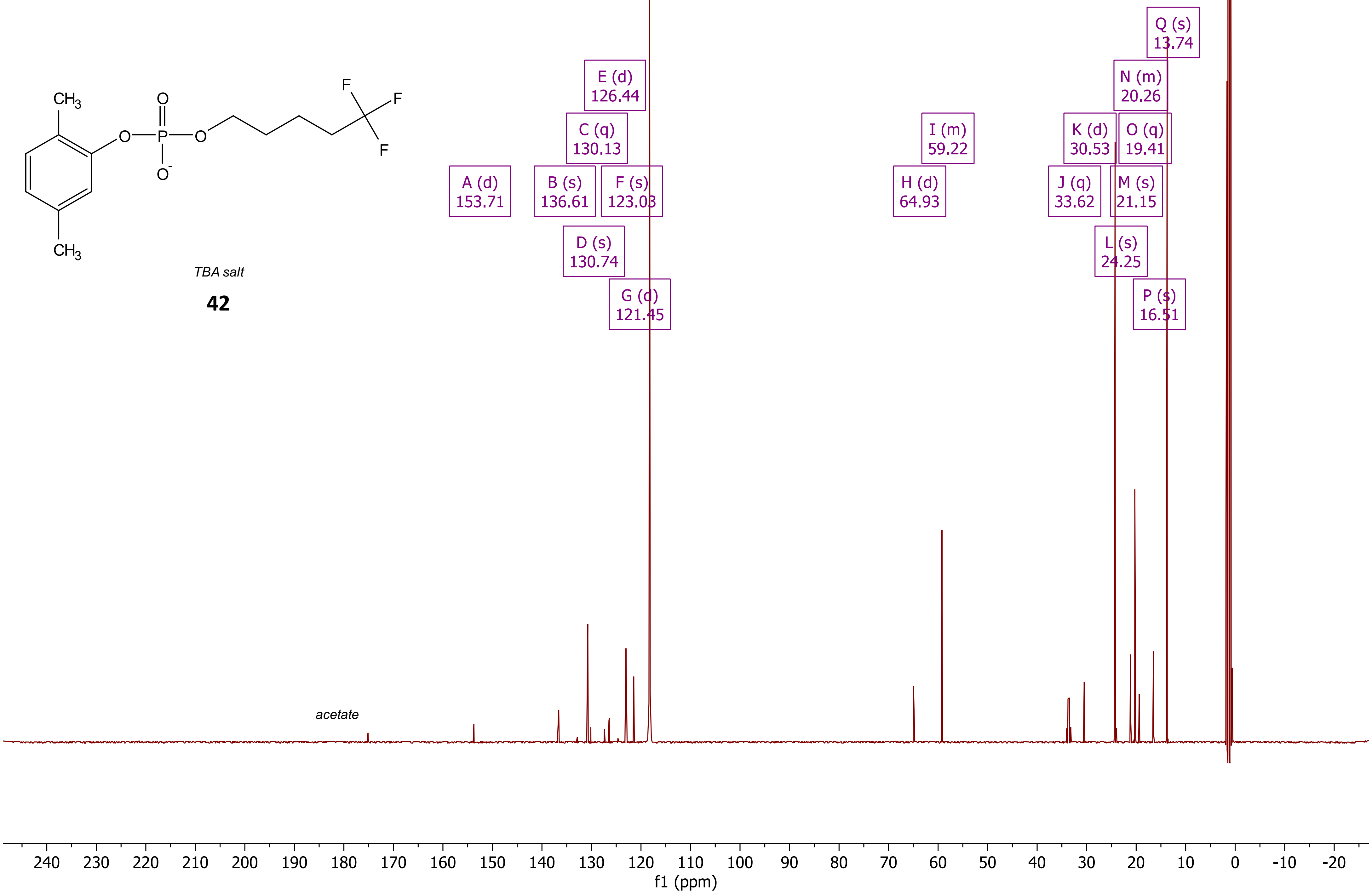

Compound 43, 3,4-dimethoxyphenyl (5,5,5-trifluoropentyl) phosphate, ^1H - NMR (CD_3CN, 400 MHz)

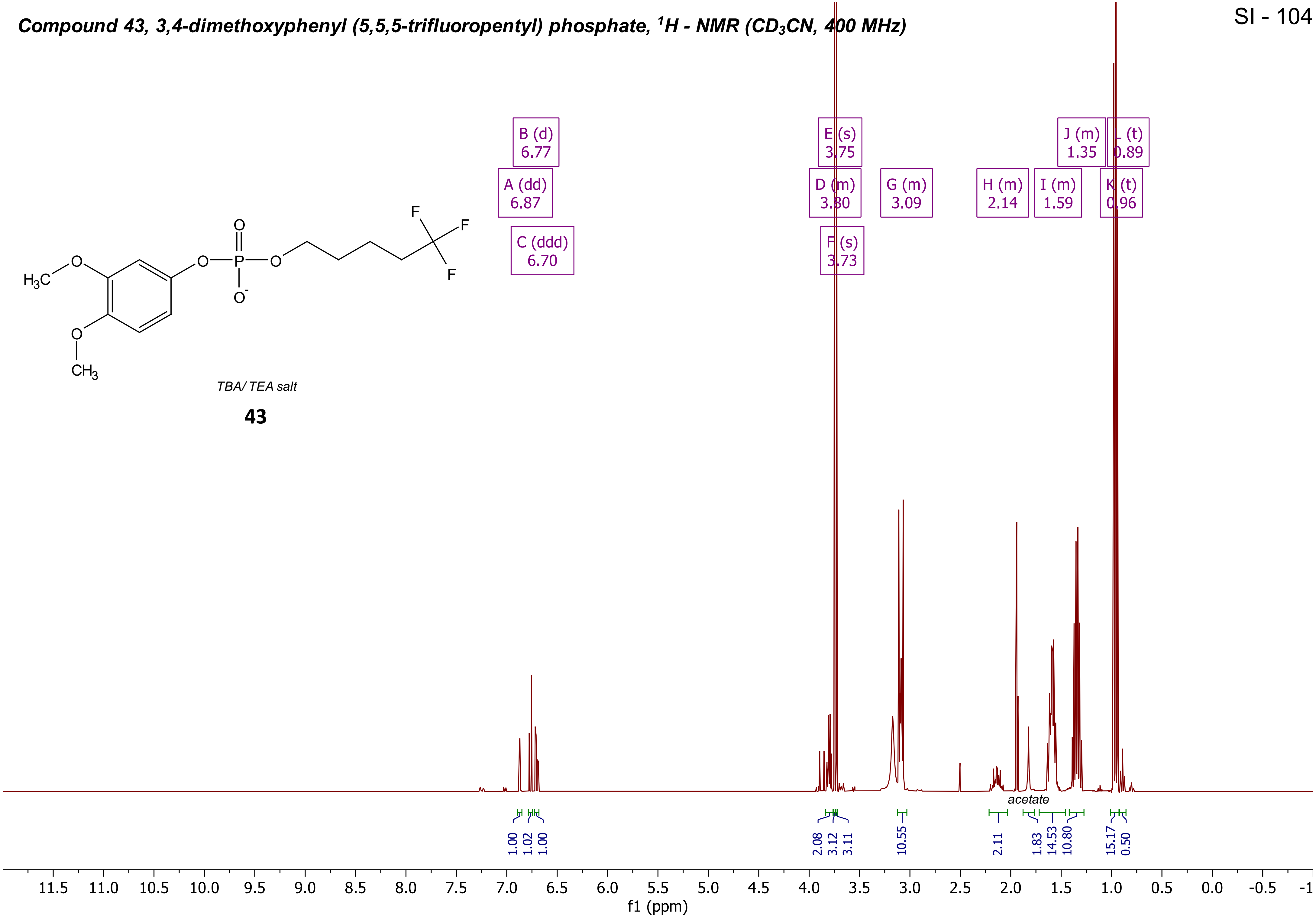

Compound 43, 3,4-dimethoxyphenyl (5,5,5-trifluoropentyl) phosphate, ^{19}F - NMR (CD_3CN, 377 MHz)

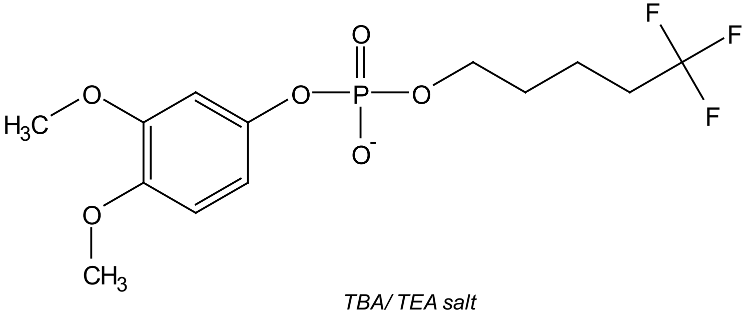

TBA/ TEA salt

43

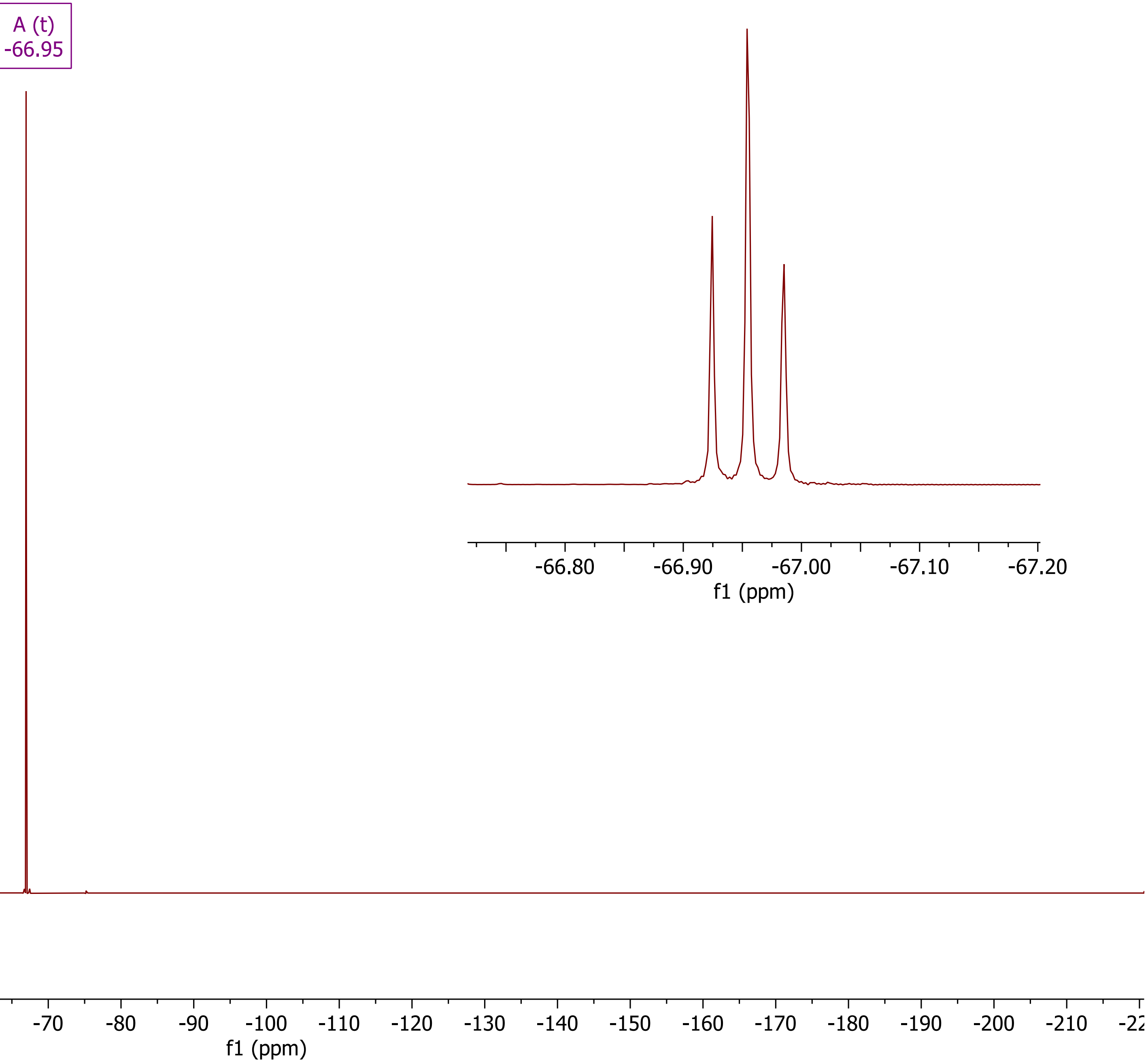

Compound 43, 3,4-dimethoxyphenyl (5,5,5-trifluoropentyl) phosphate, $^{31}P\ \{^{1}H\}$ - NMR (CD_3CN, 162 MHz)

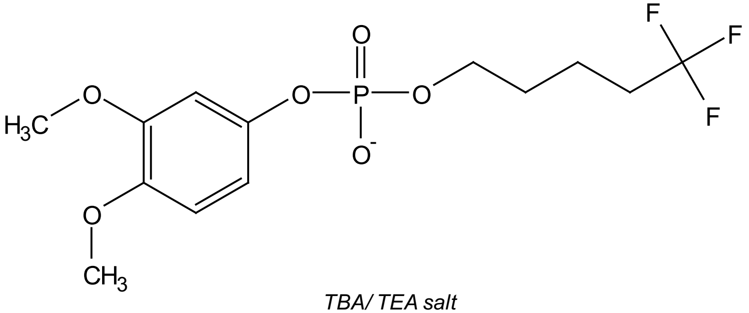

TBA/ TEA salt

43

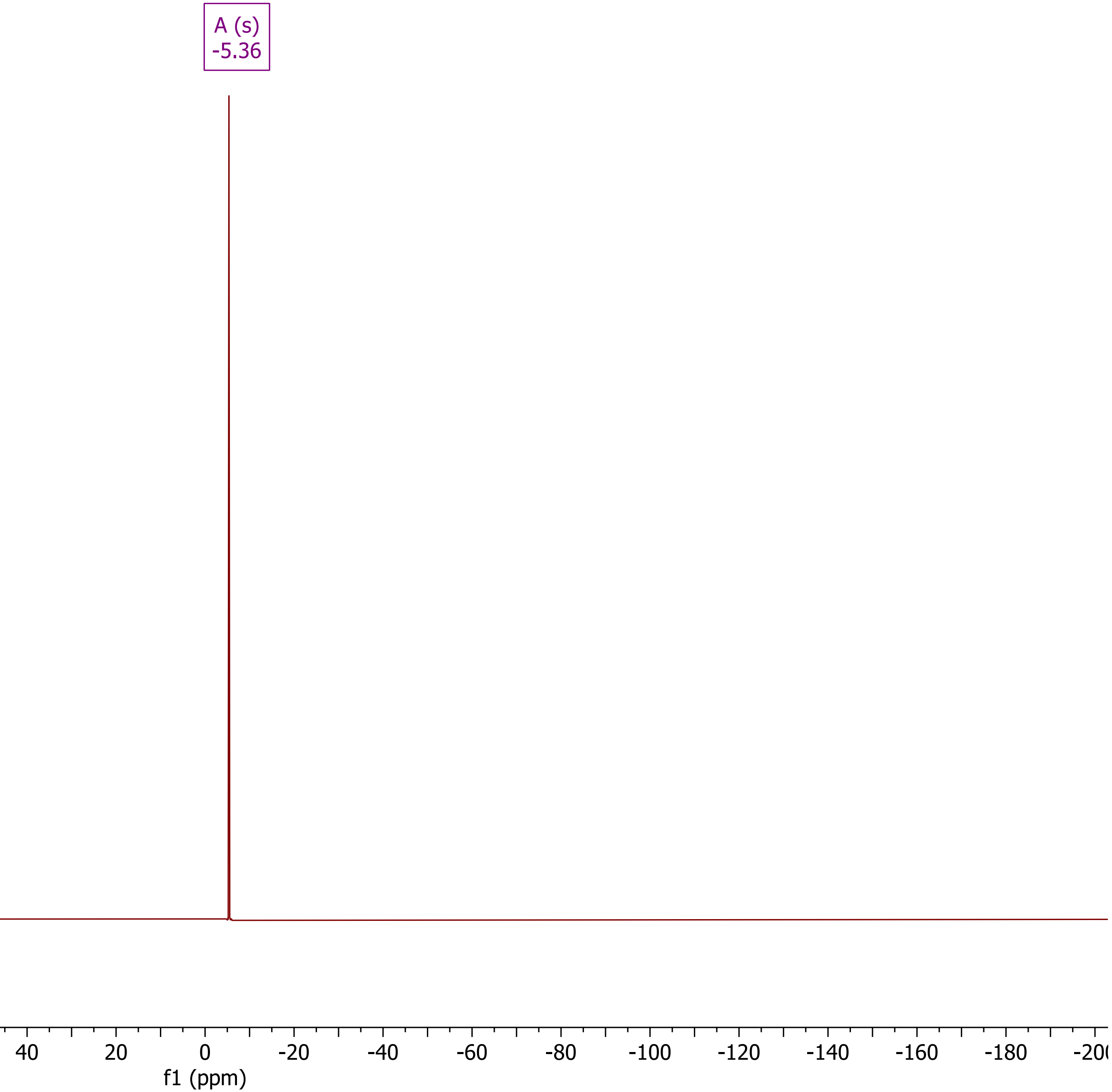

Compound 43, 3,4-dimethoxyphenyl (5,5,5-trifluoropentyl) phosphate, ^{13}C - NMR (CD_3CN, 101 MHz)

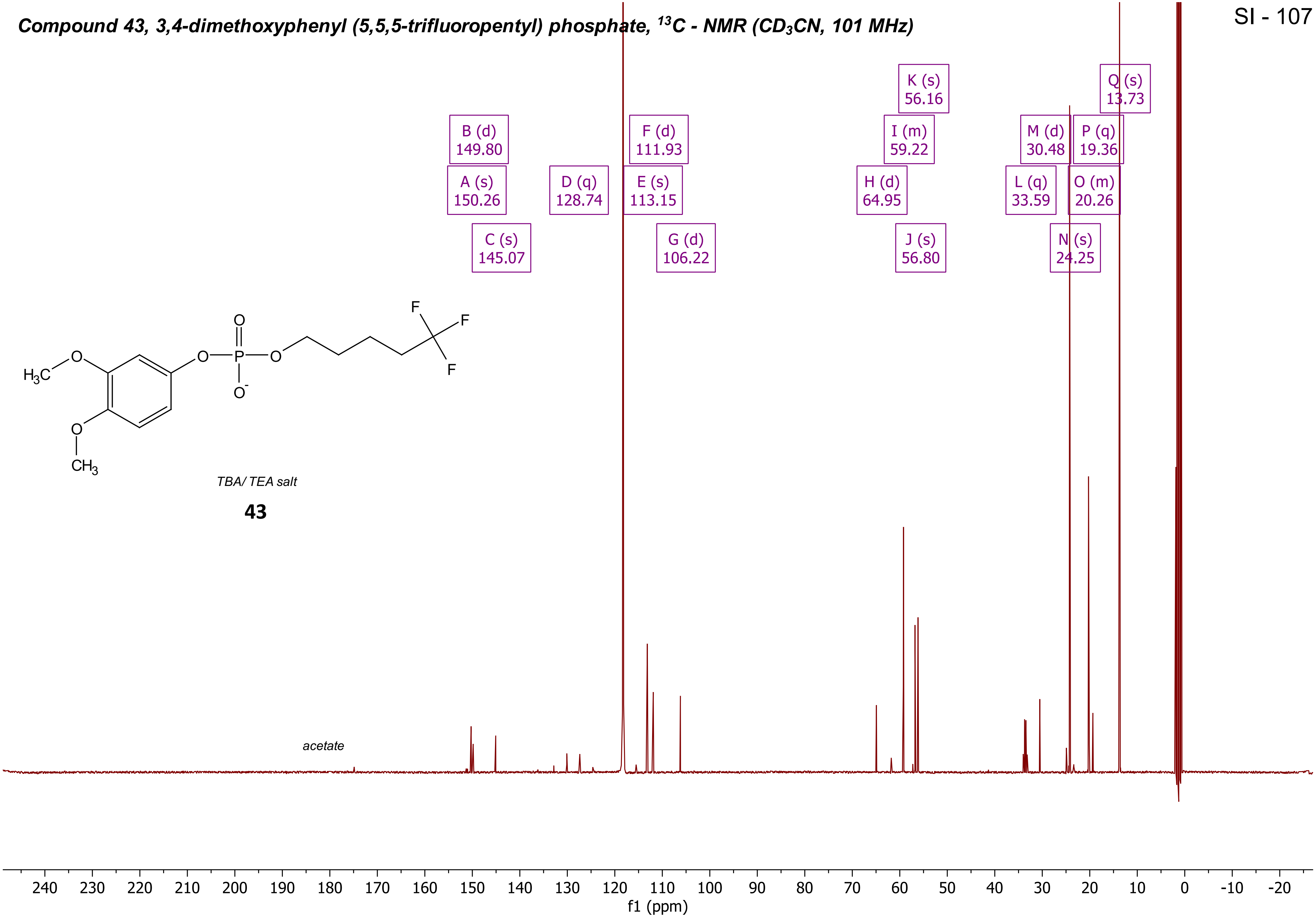

Compound 44, 4-(trifluoromethyl)phenyl (5,5,5-trifluoropentyl) phosphate, ^{1}H - NMR (CD_3CN, 400 MHz)

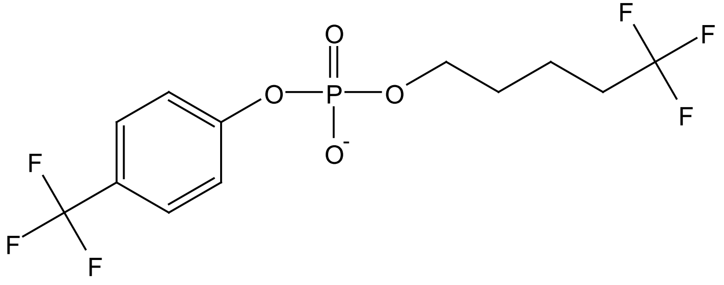

TBA salt

44

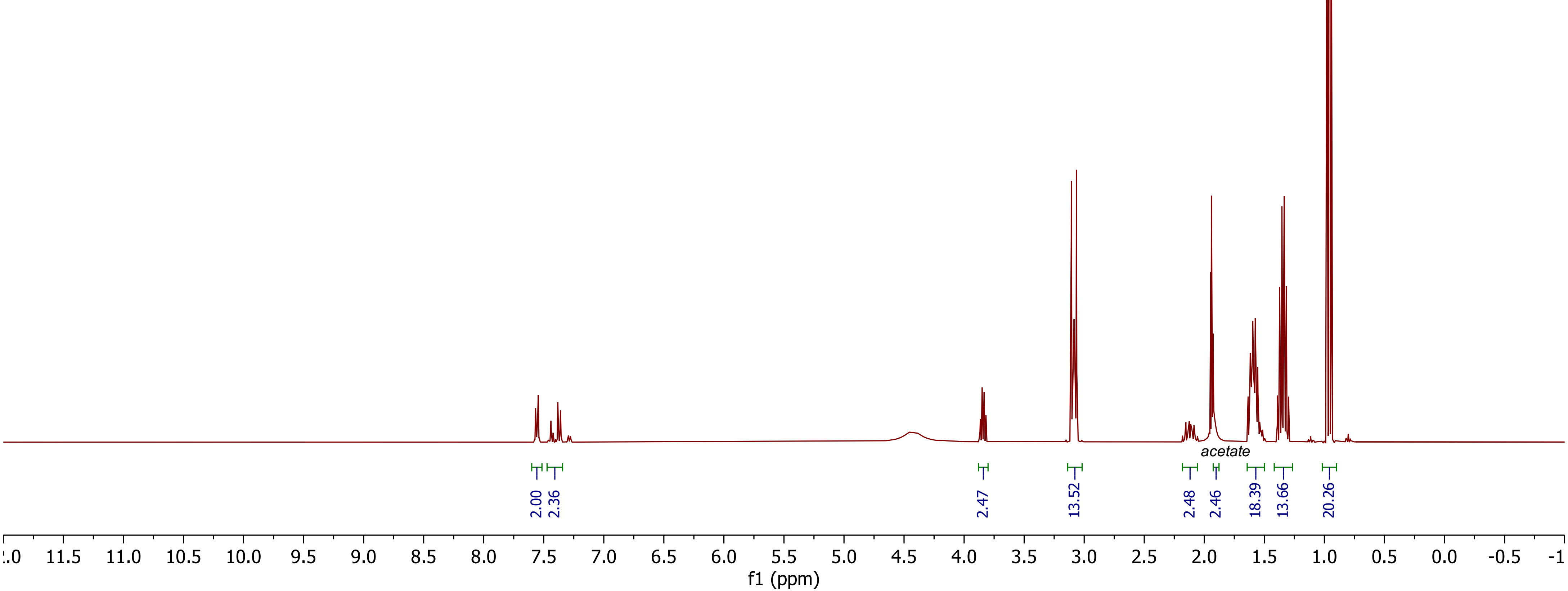

Compound 44, 4-(trifluoromethyl)phenyl (5,5,5-trifluoropentyl) phosphate, ^{19}F - NMR (CD_3CN, 377 MHz)

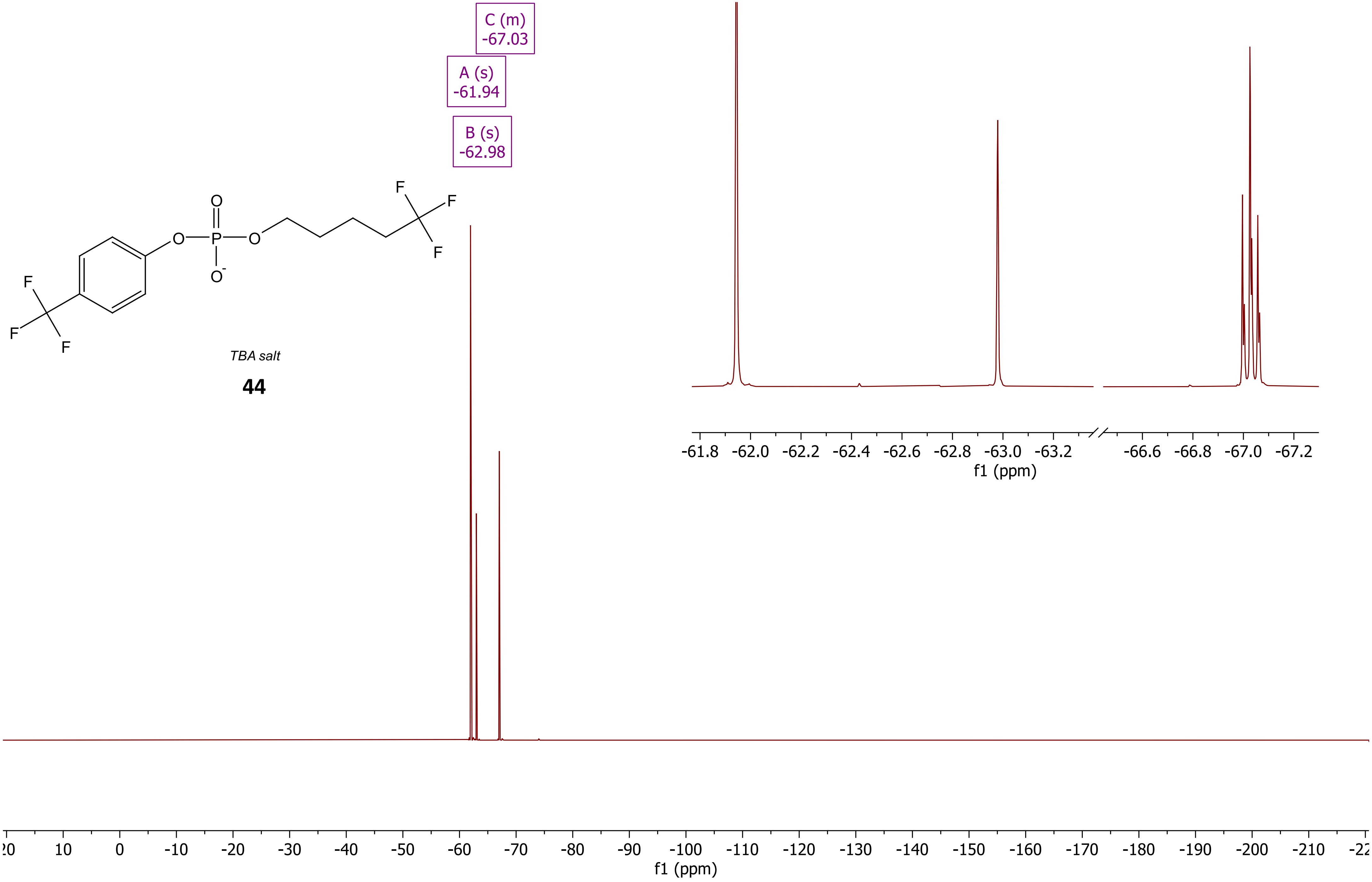

Compound 44, 4-(trifluoromethyl)phenyl (5,5,5-trifluoropentyl) phosphate, $^{31}P\{^1H\}$ - NMR (CD_3CN, 162 MHz)

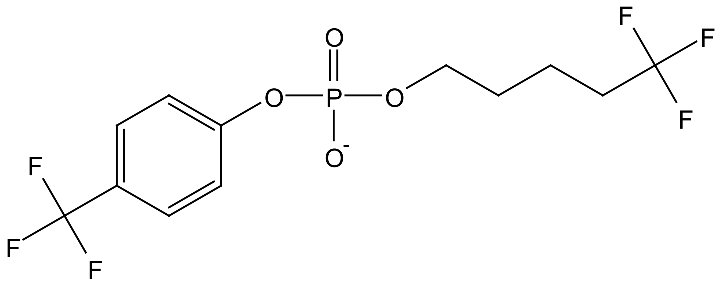

TBA salt

44

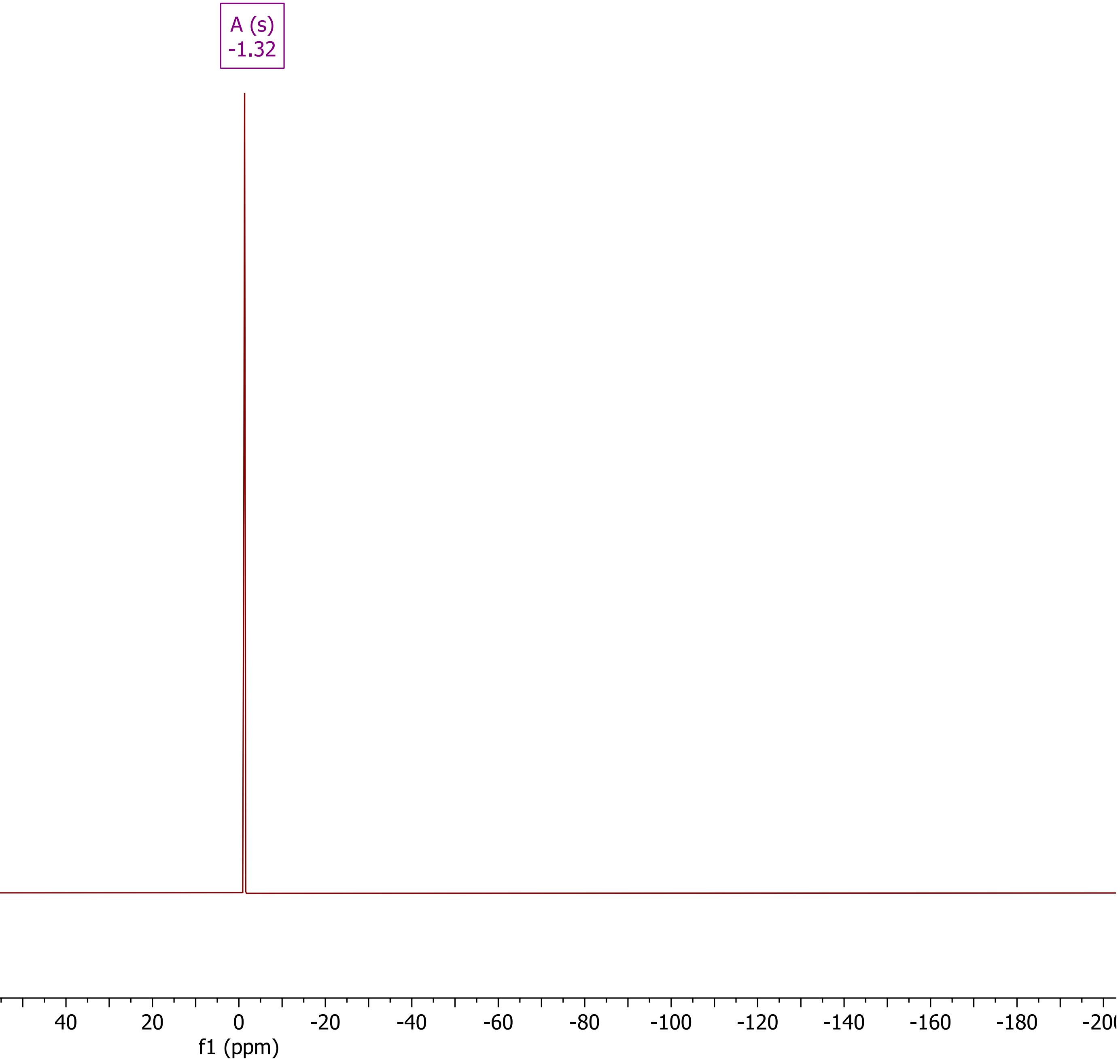

Compound 44, 4-(trifluoromethyl)phenyl (5,5,5-trifluoropentyl) phosphate, ^{13}C - NMR (CD_3CN, 101 MHz)

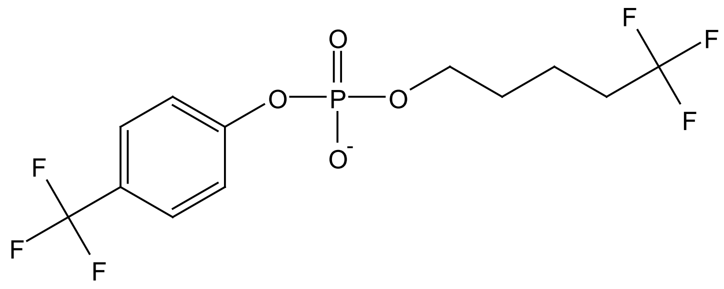

TBA salt

44

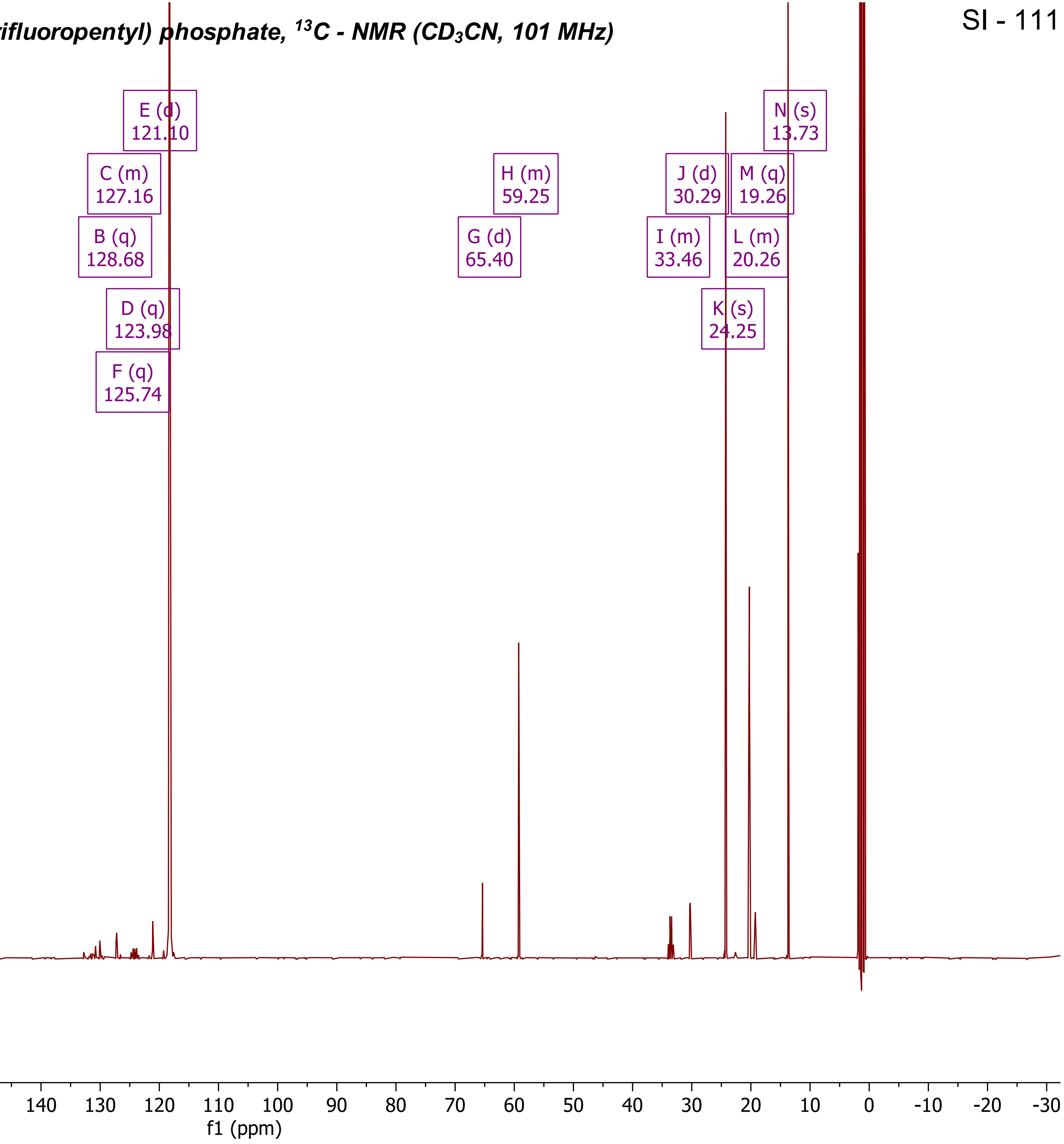

Compound 45, benzo[d][1,3]dioxol-5-yl (5,5,5-trifluoropentyl) phosphate, ^{1}H - NMR (CD_3CN, 400 MHz)

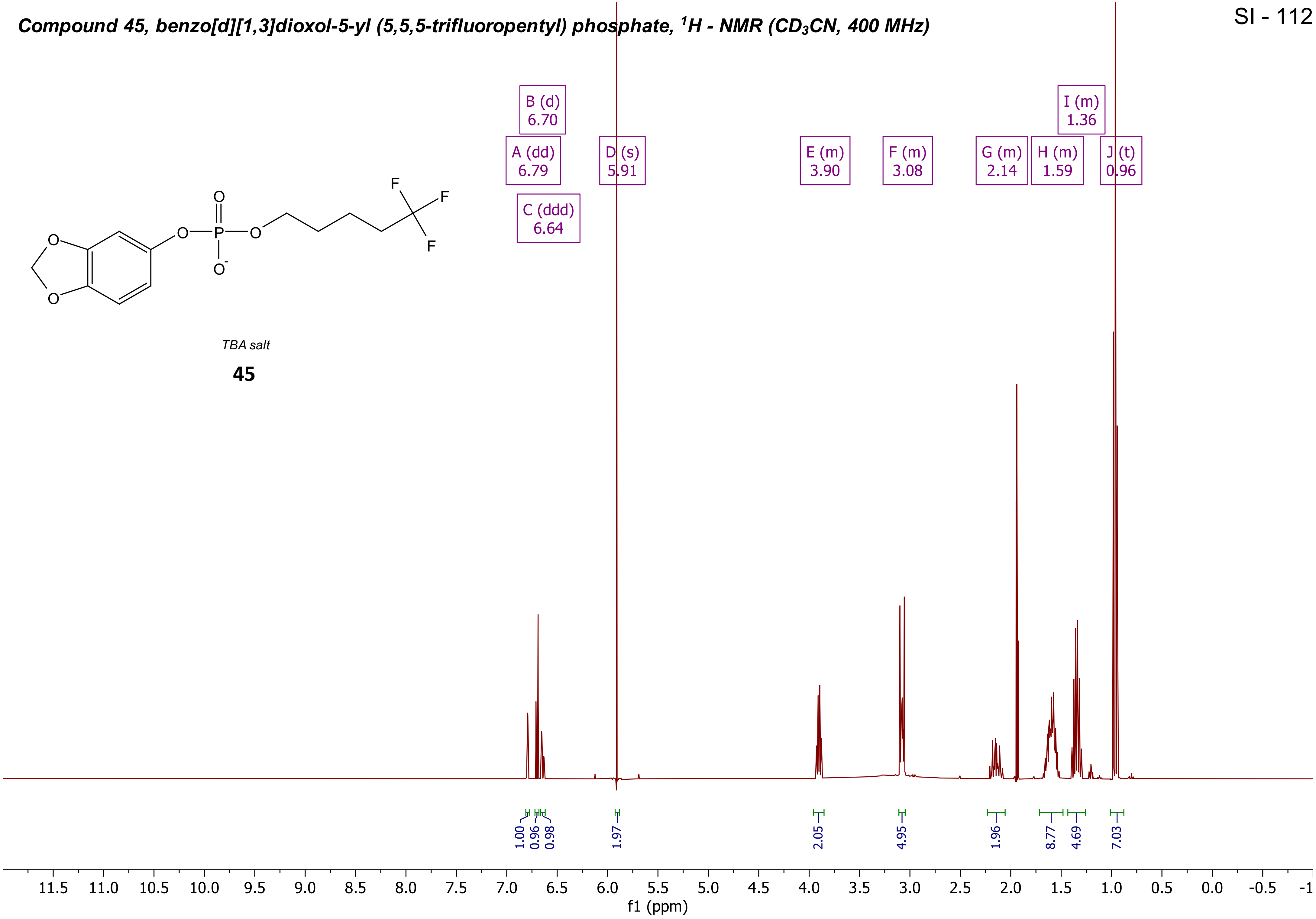

TBA salt

45

Compound 45, benzo[d][1,3]dioxol-5-yl (5,5,5-trifluoropentyl) phosphate, ^{19}F - NMR (CD_3CN, 377 MHz)

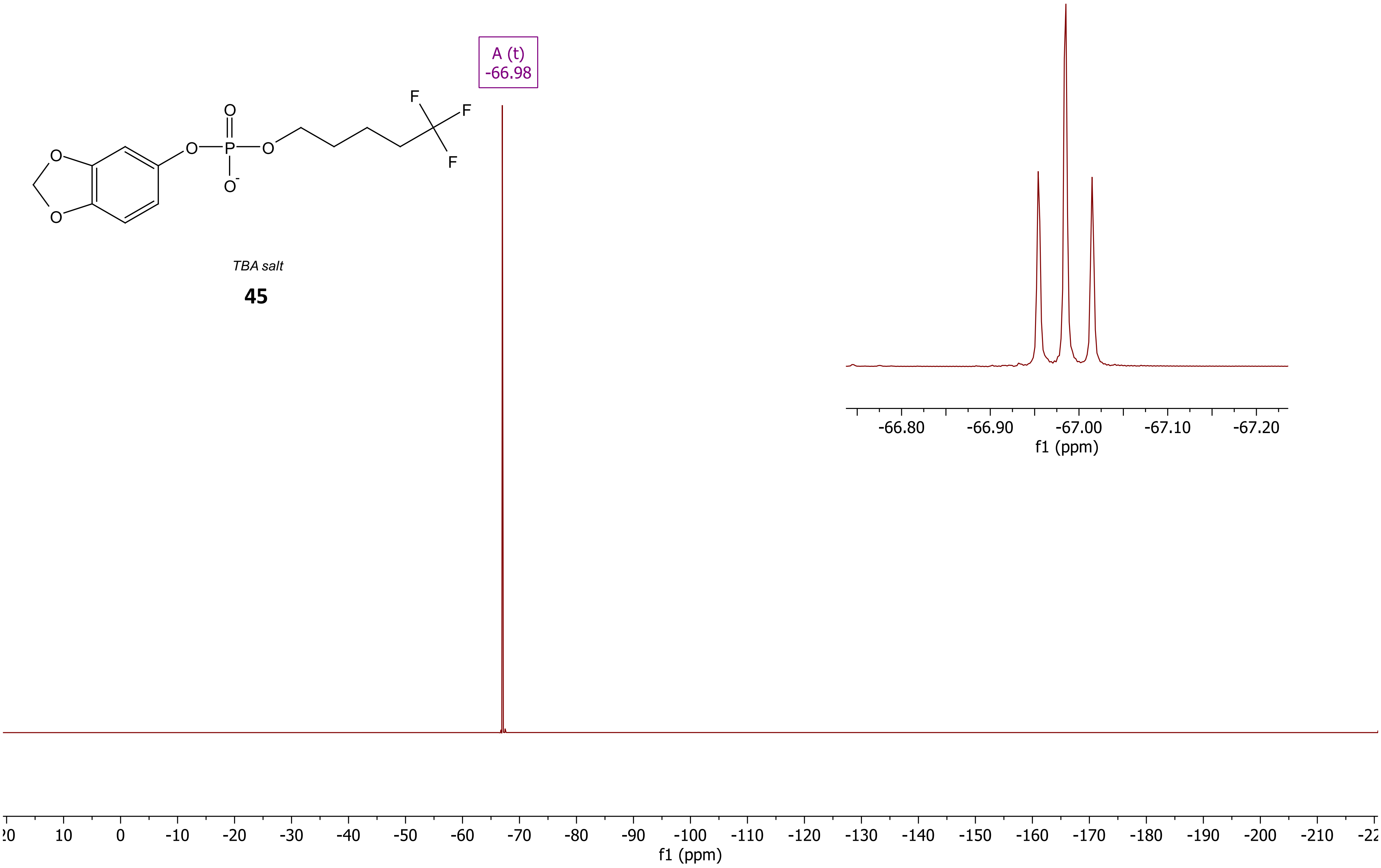

Compound 45, benzo[d][1,3]dioxol-5-yl (5,5,5-trifluoropentyl) phosphate, $^{31}P\ \{^{1}H\}$ - NMR (CD_3CN, 162 MHz)

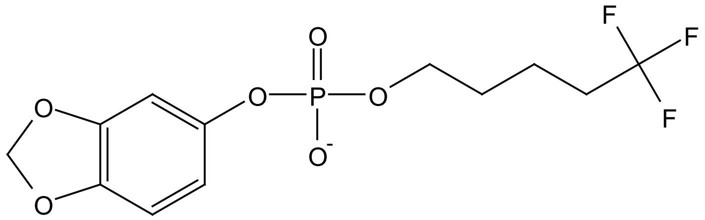

TBA salt

45

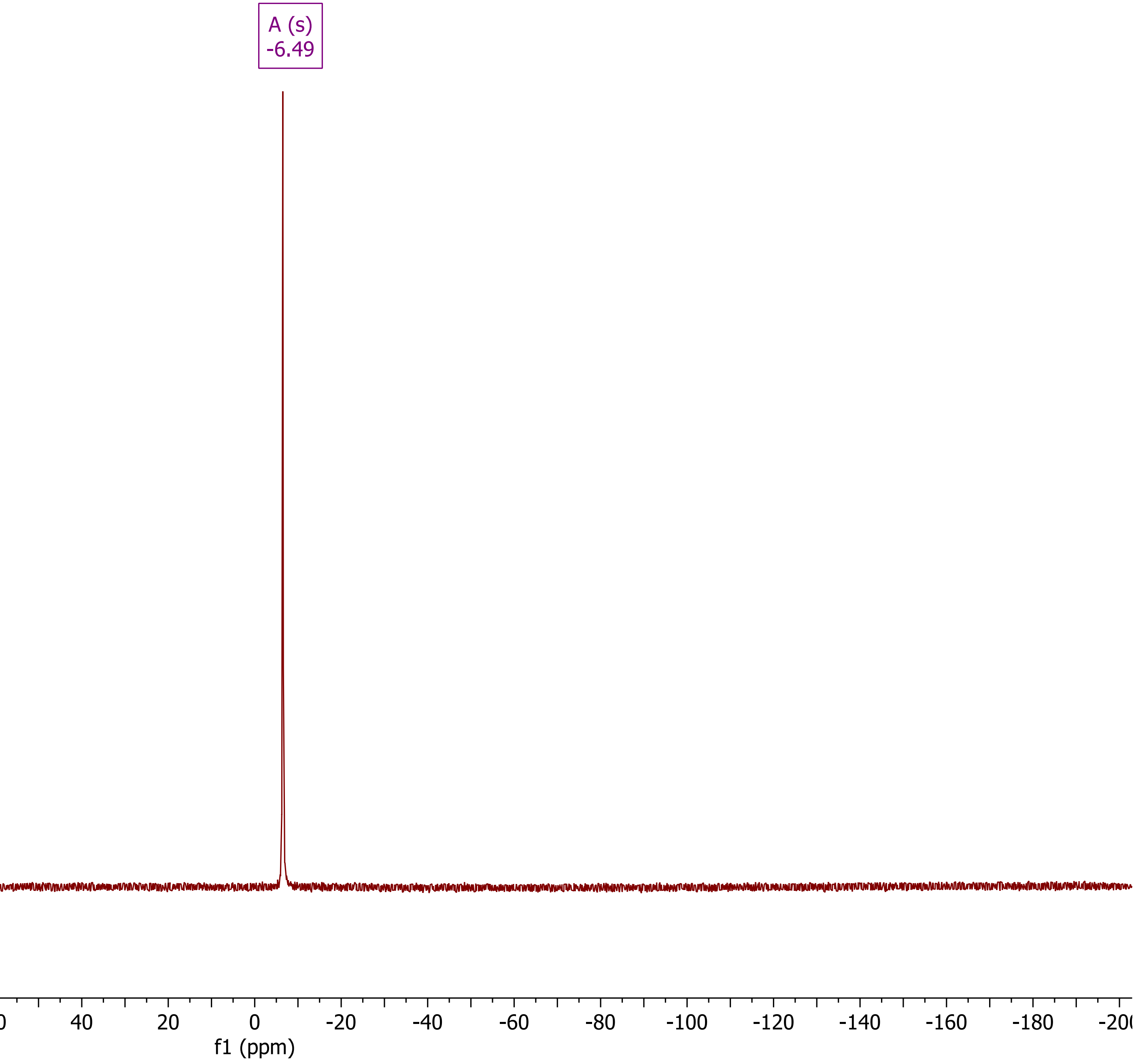

Compound 45, benzo[d][1,3]dioxol-5-yl (5,5,5-trifluoropentyl) phosphate, ^{13}C - NMR (CD_3CN, 101 MHz)

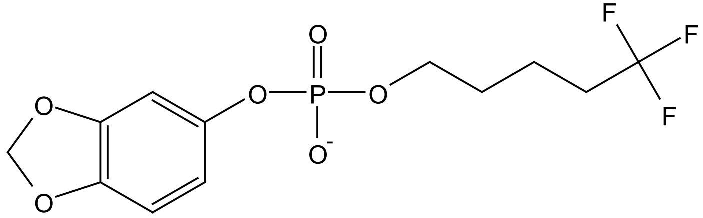

TBA salt

45

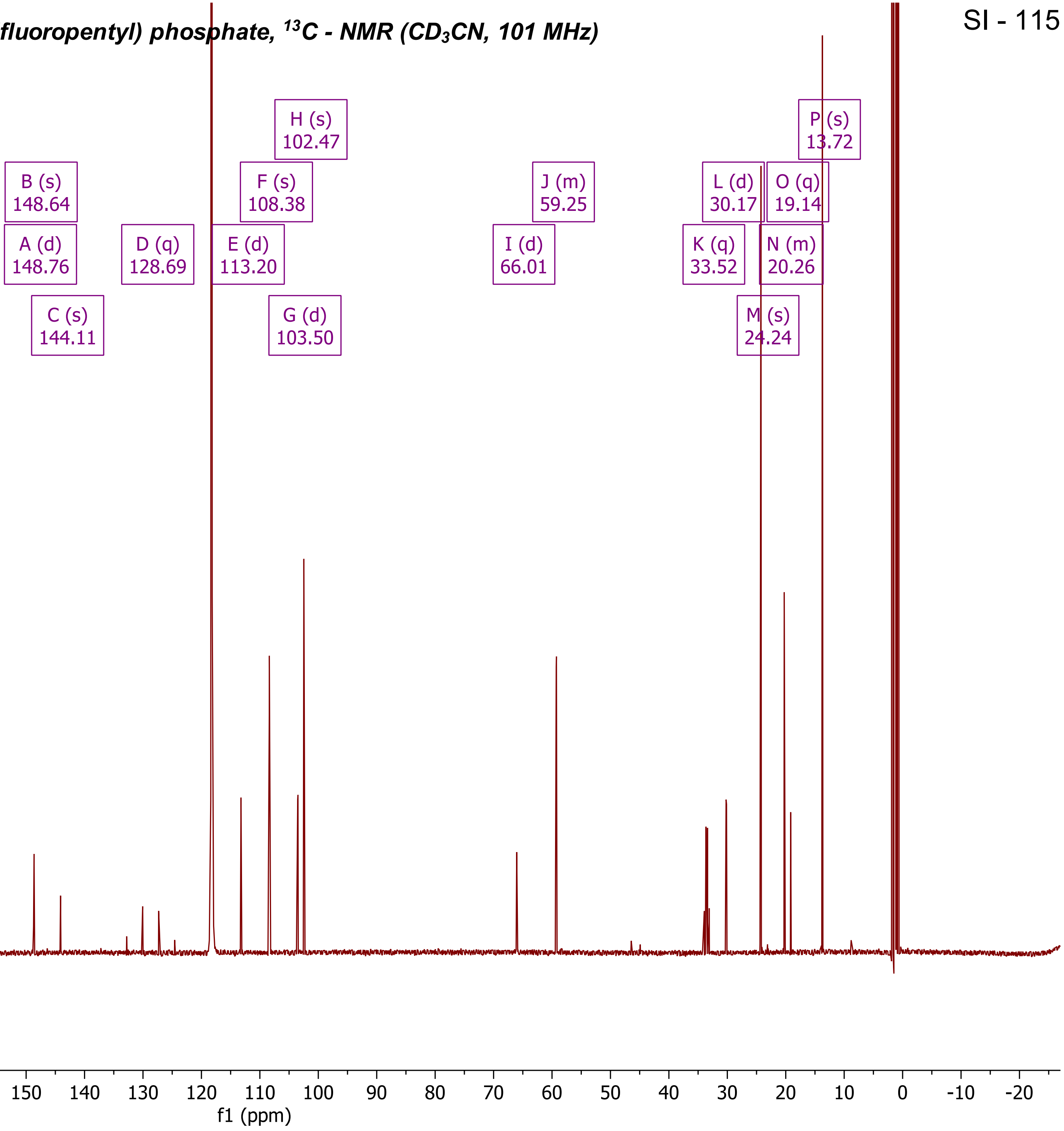

Compound 46, 4-ethynylphenyl (5,5,5-trifluoropentyl) phosphate, ^{1}H - NMR (CD_3CN, 400 MHz)

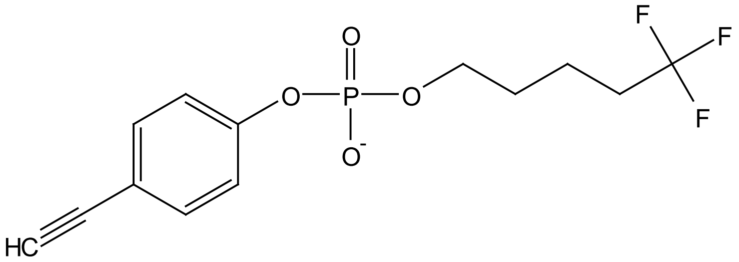

TBA salt

46

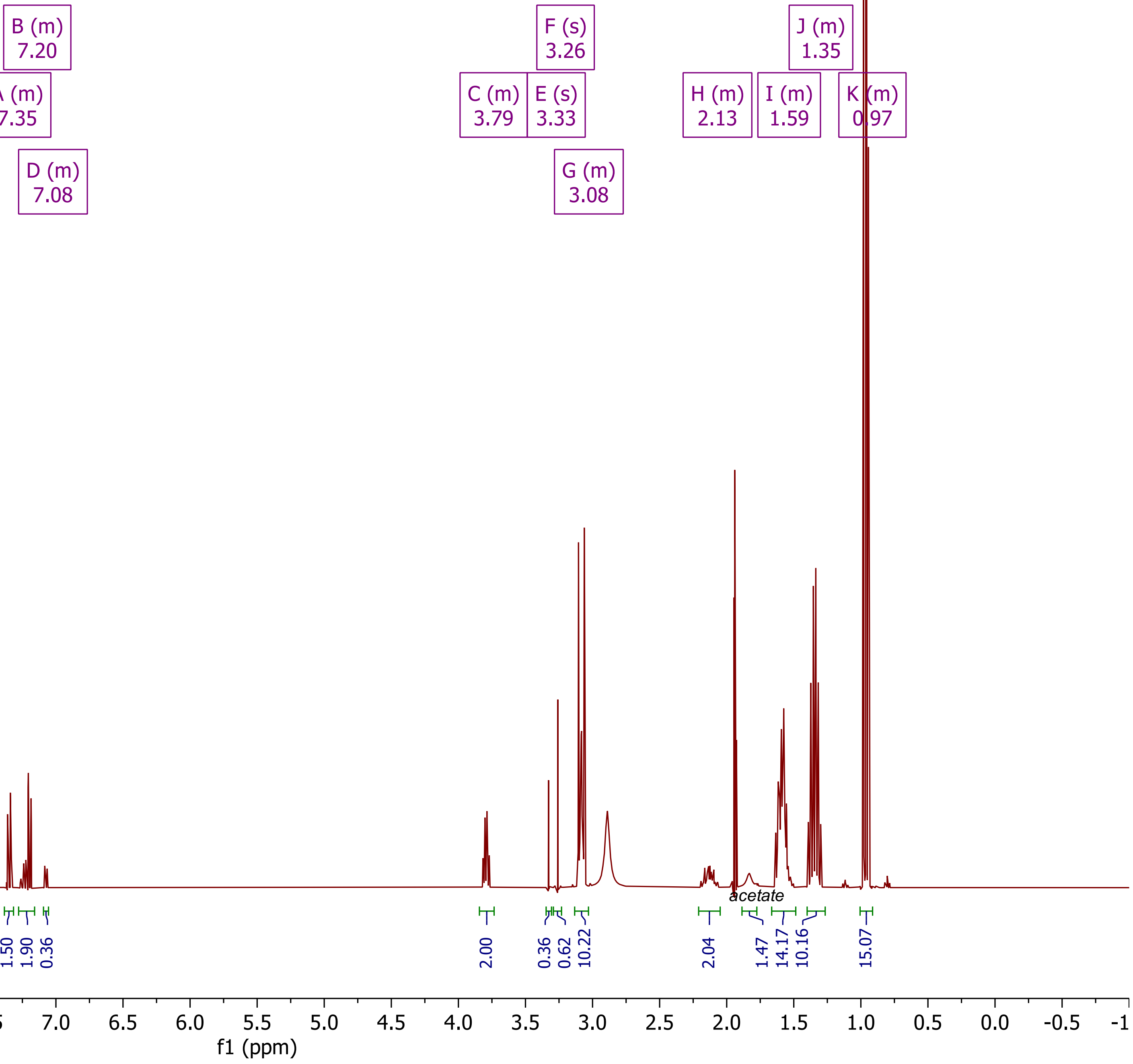

Compound 46, 4-ethynylphenyl (5,5,5-trifluoropentyl) phosphate, ^{19}F - NMR (CD_3CN, 377 MHz)

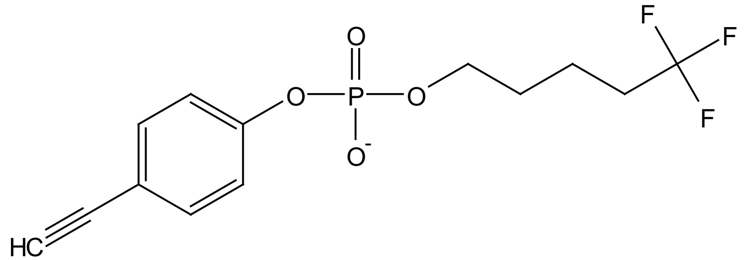

TBA salt

46

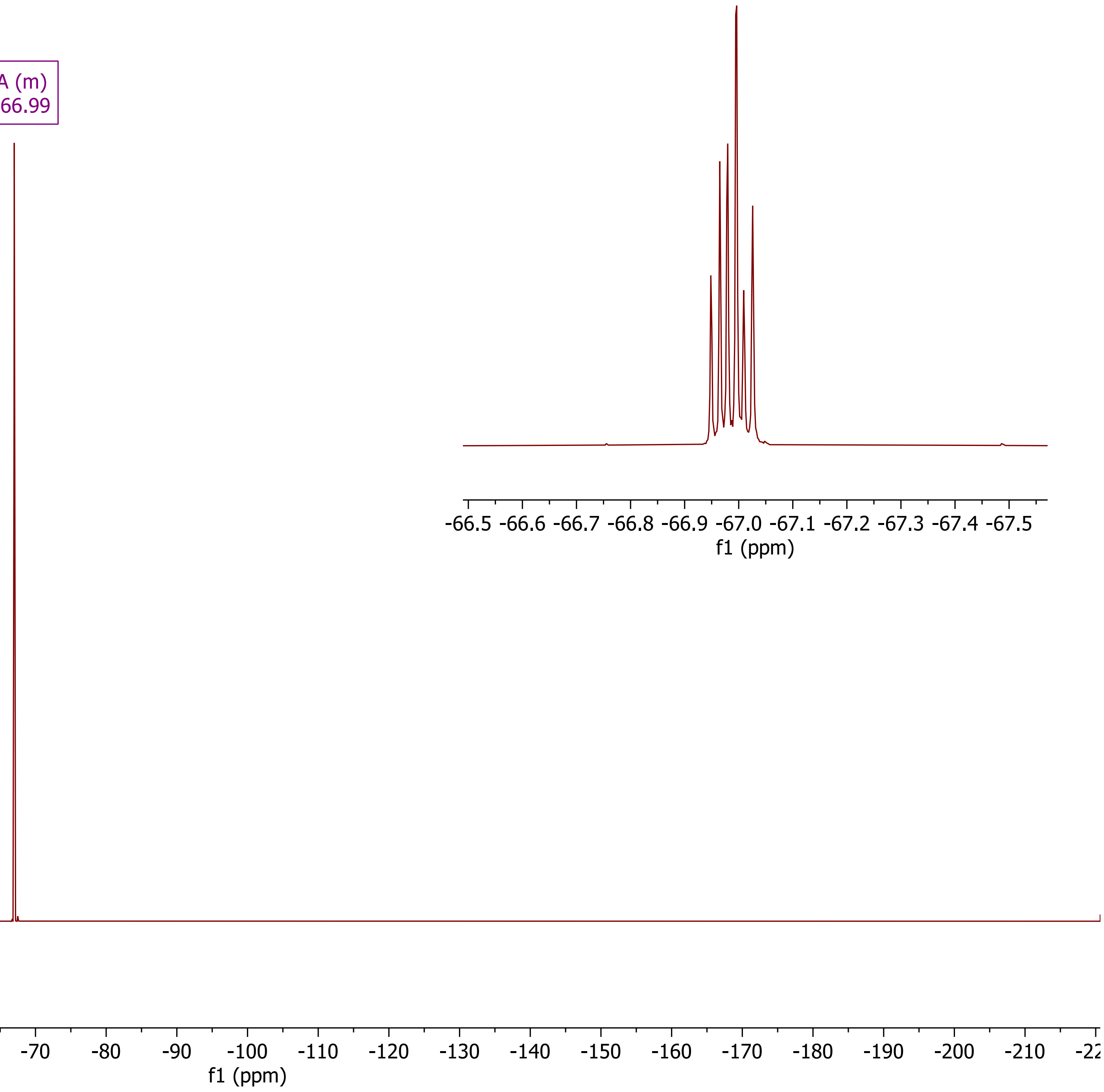

Compound 46, 4-ethynylphenyl (5,5,5-trifluoropentyl) phosphate, $^{31}P\ \{^{1}H\}$ - NMR (CD_3CN, 162 MHz)

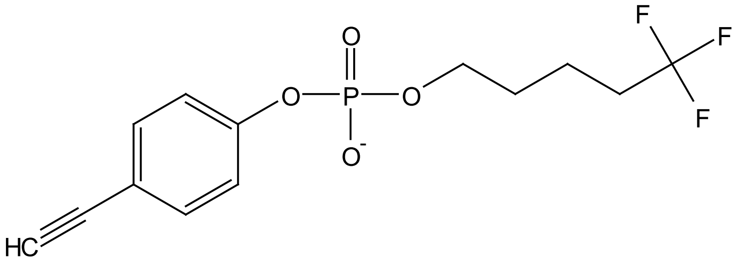

TBA salt

46

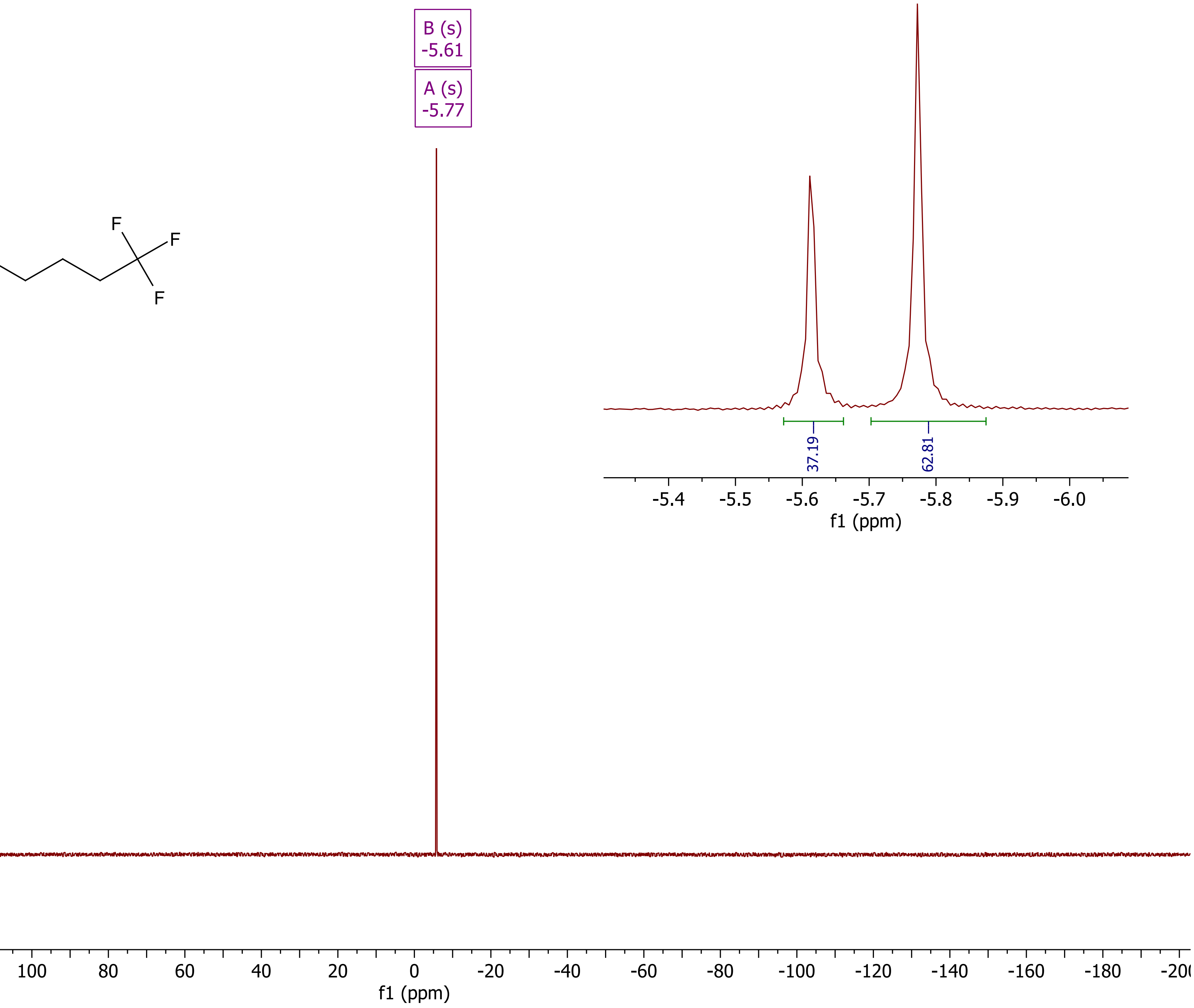

Compound 46, 4-ethynylphenyl (5,5,5-trifluoropentyl) phosphate, ^{13}C - NMR (CD_3CN, 101 MHz)

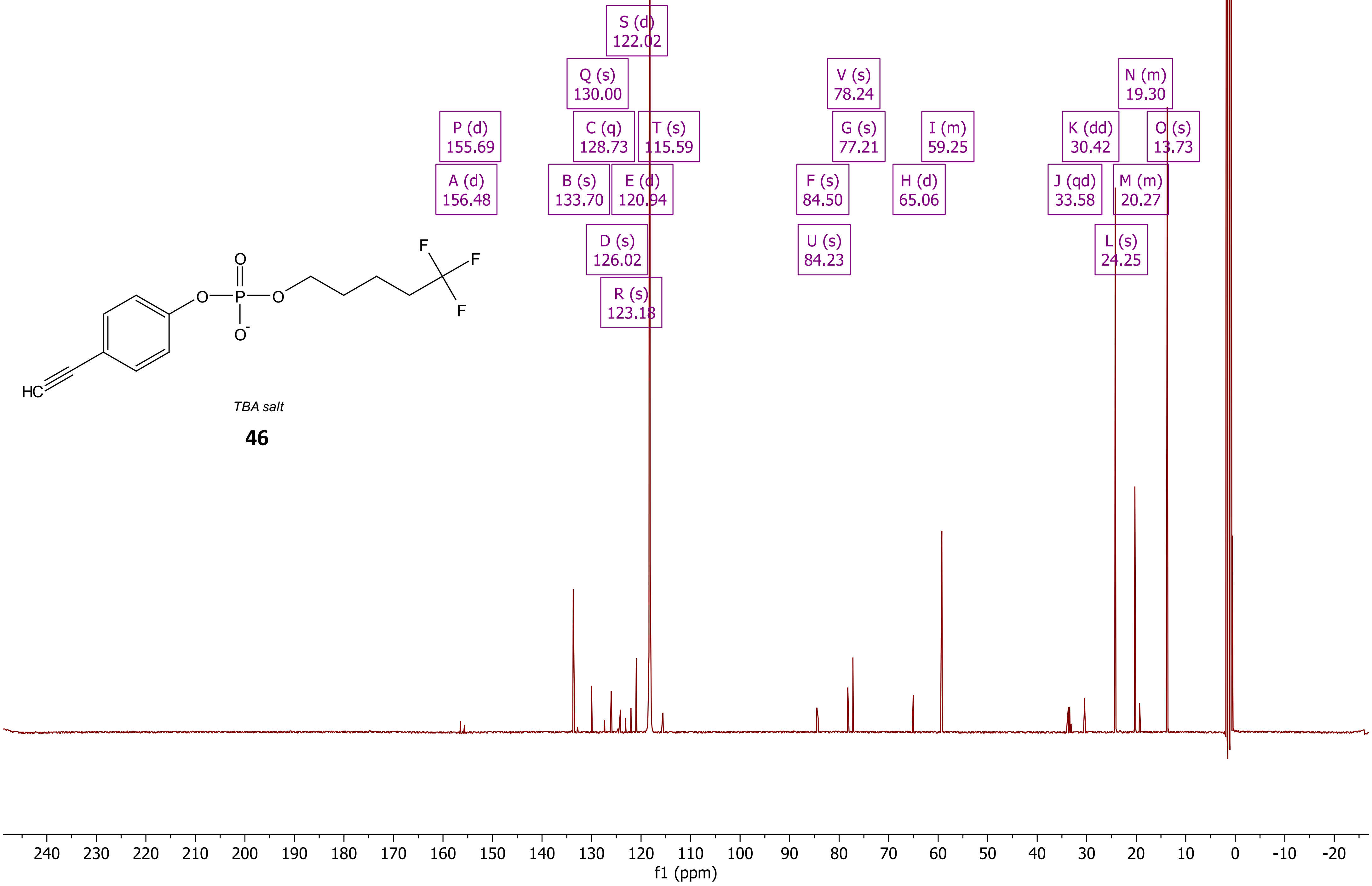

Compound 47, 3-bromophenyl (5,5,5-trifluoropentyl) phosphate, ^{1}H - NMR (CD_3CN, 400 MHz)

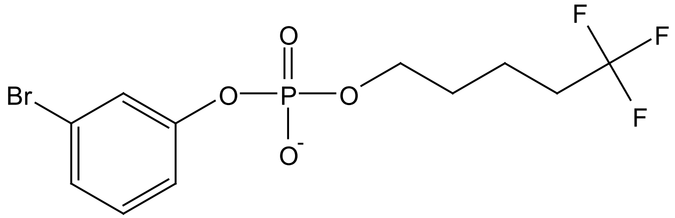

TBA salt

47

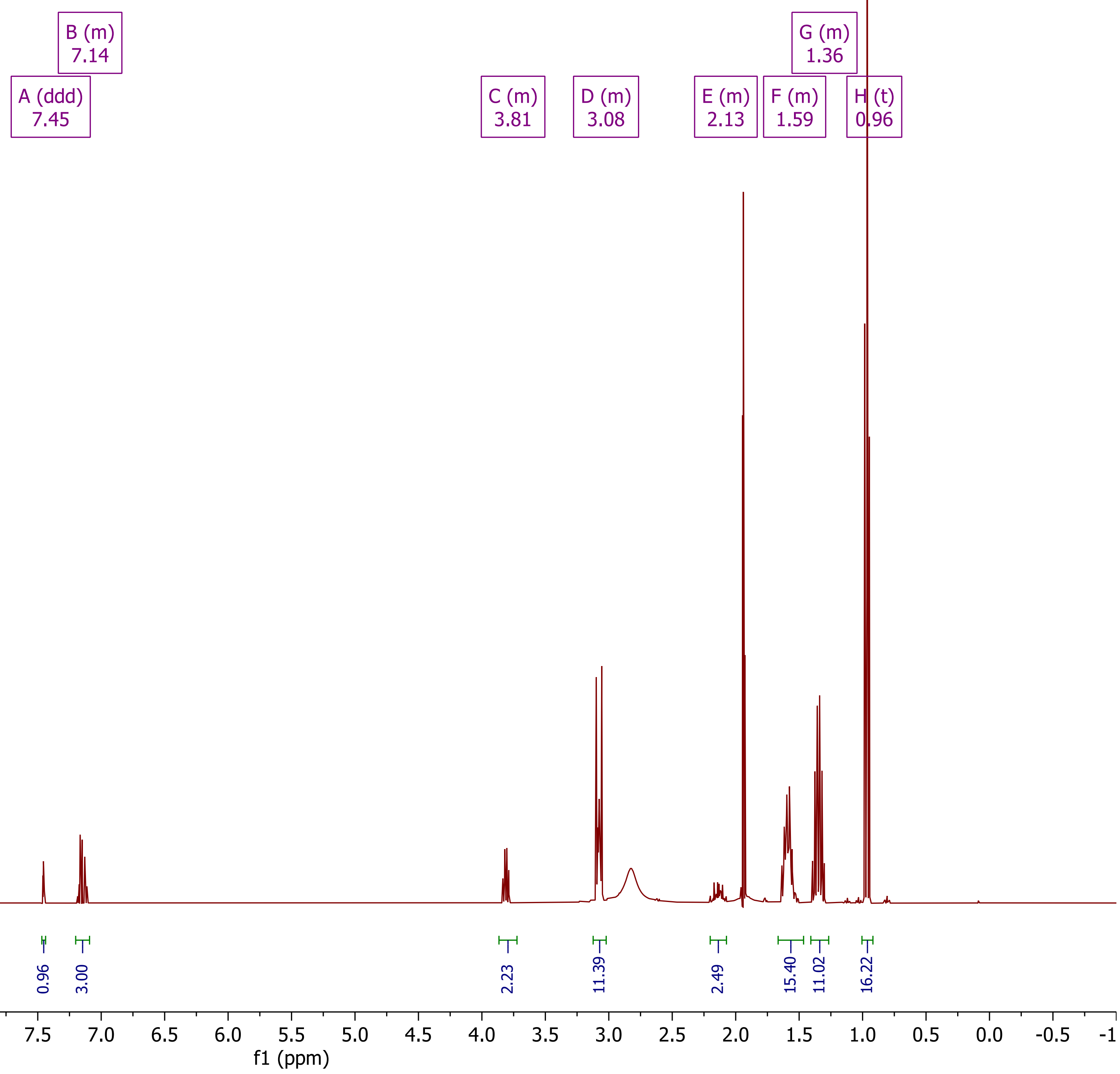

Compound 47, 3-bromophenyl (5,5,5-trifluoropentyl) phosphate, ^{19}F - NMR (CD_3CN, 377 MHz)

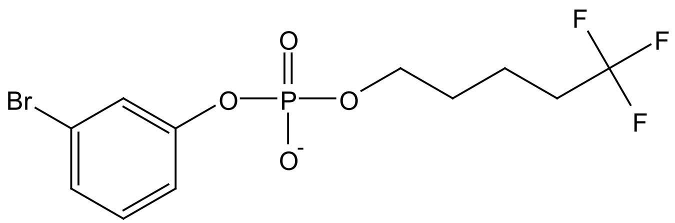

TBA salt

47

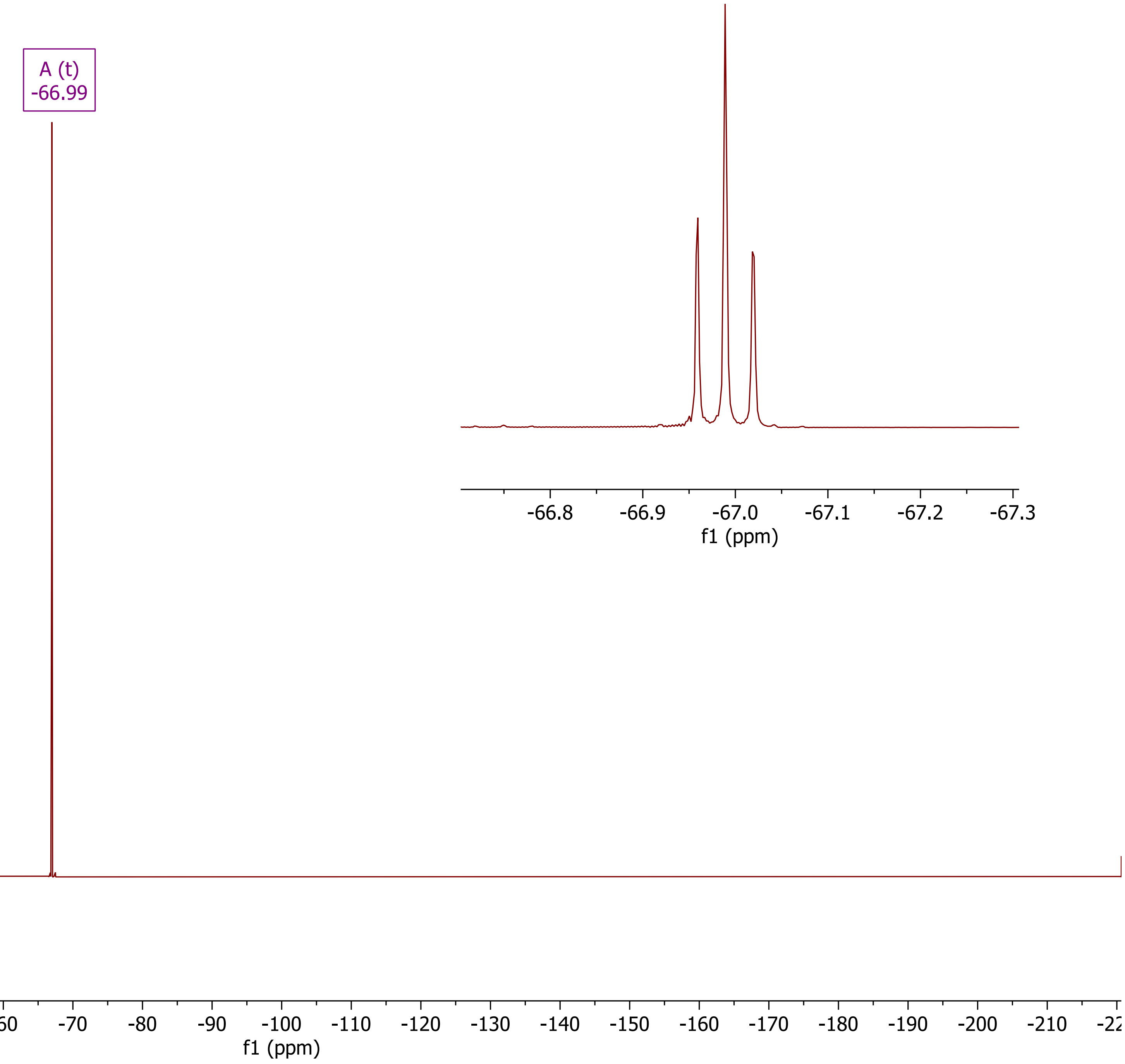

Compound 47, 3-bromophenyl (5,5,5-trifluoropentyl) phosphate, $^{31}P\{^{1}H\}$ - NMR (CD_3CN, 162 MHz)

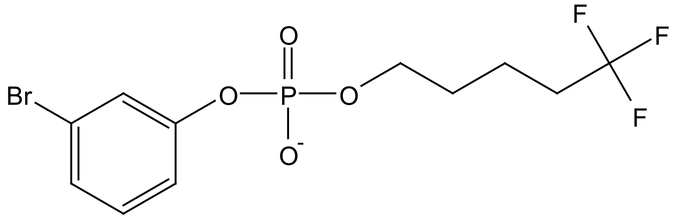

TBA salt

47

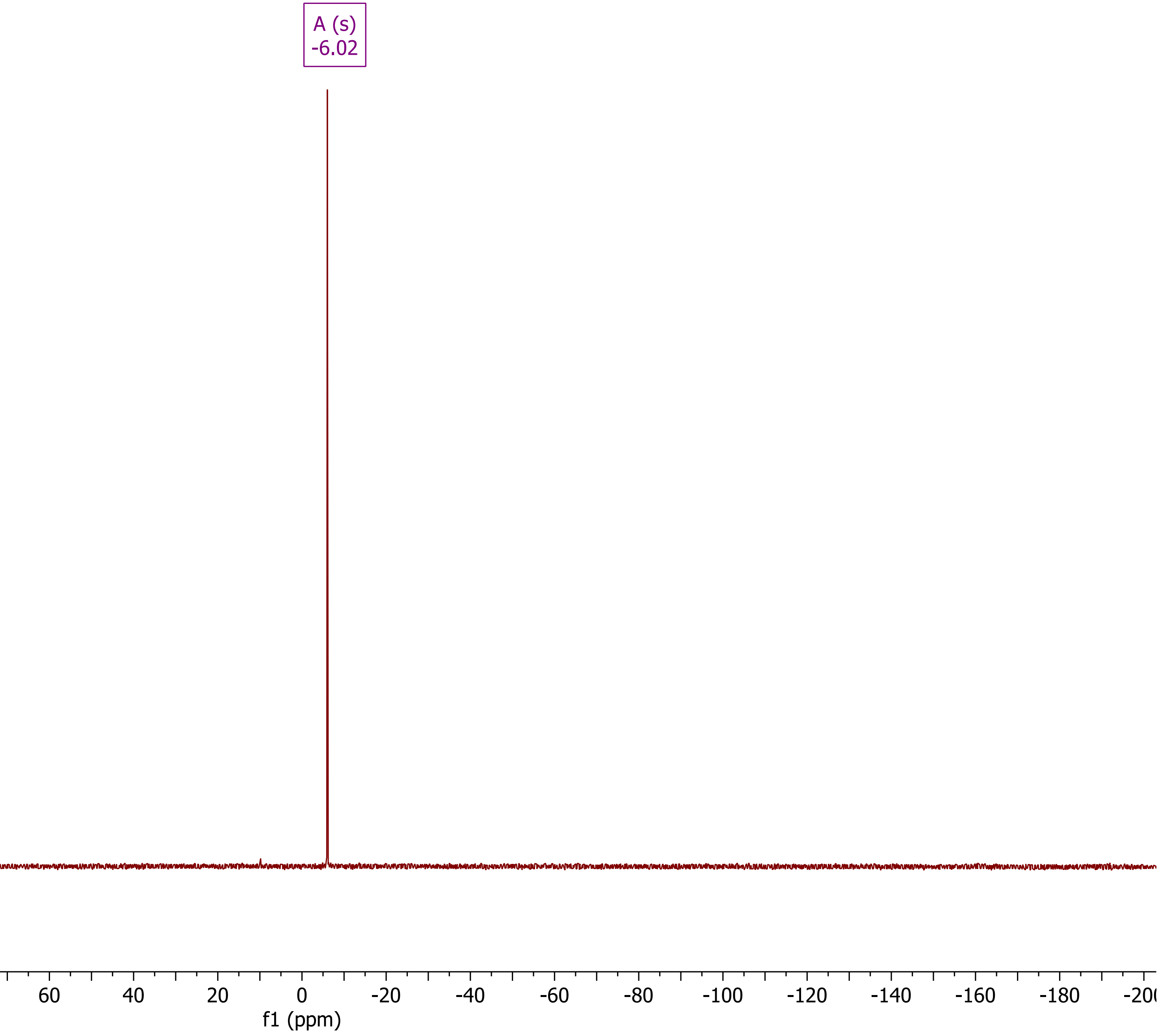

Compound 47, 3-bromophenyl (5,5,5-trifluoropentyl) phosphate, ^{13}C - NMR (CD_3CN, 101 MHz)

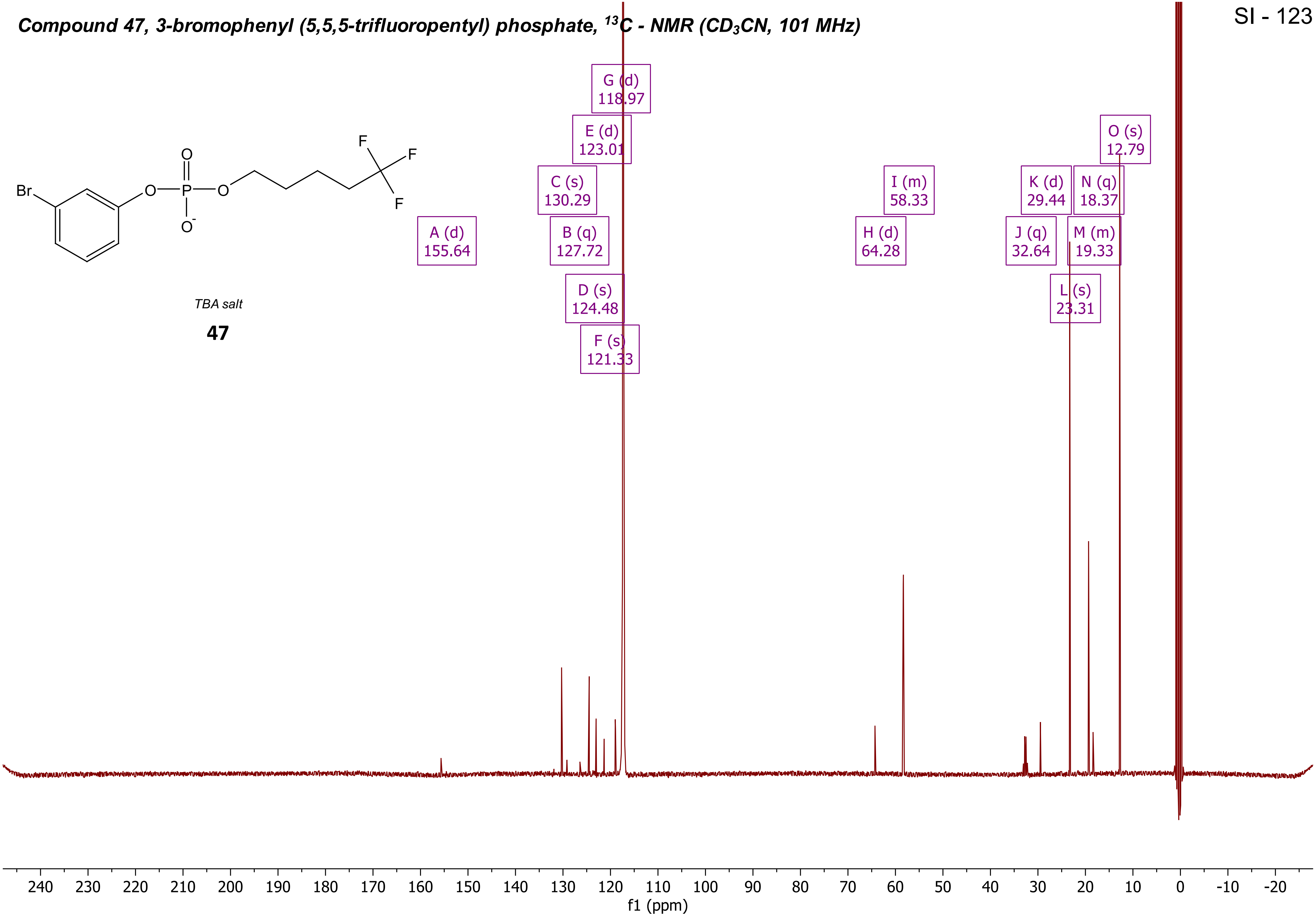

TBA salt

47

Compound 48, 4-chlorophenyl (5,5,5-trifluoropentyl) phosphate, ^{1}H - NMR (CD_3CN, 400 MHz)

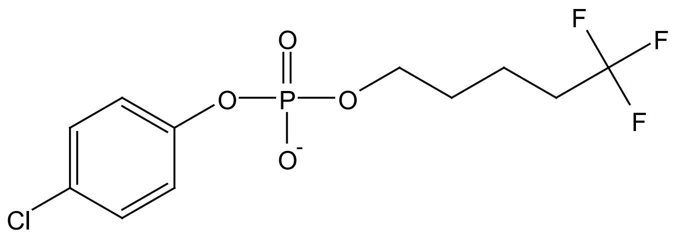

TBA salt

48

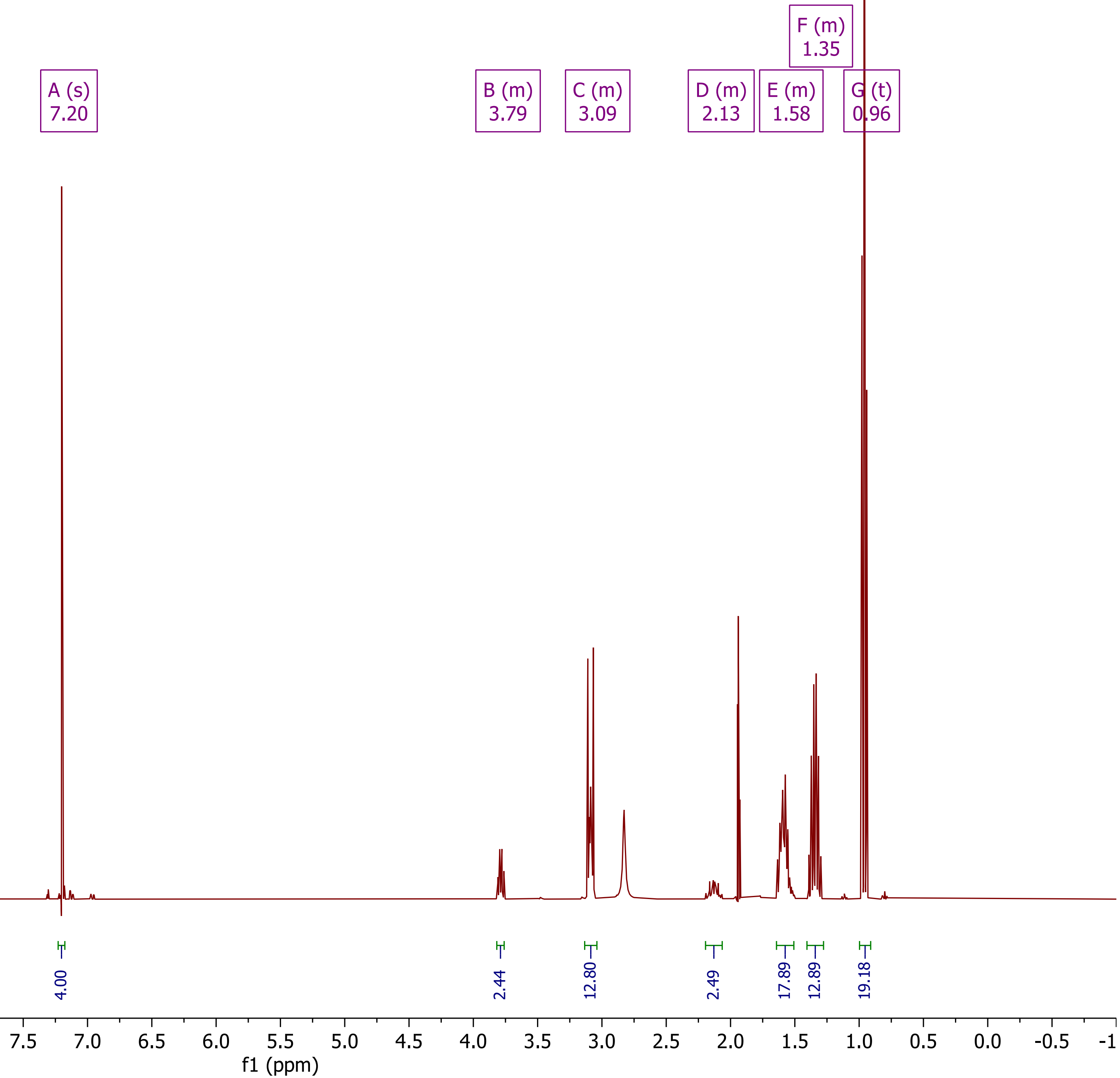

Compound 48, 4-chlorophenyl (5,5,5-trifluoropentyl) phosphate, ^{19}F - NMR (CD_3CN, 377 MHz)

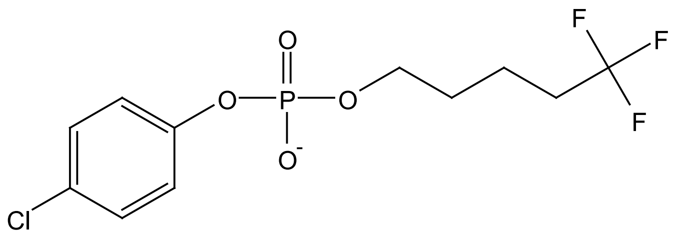

TBA salt

48

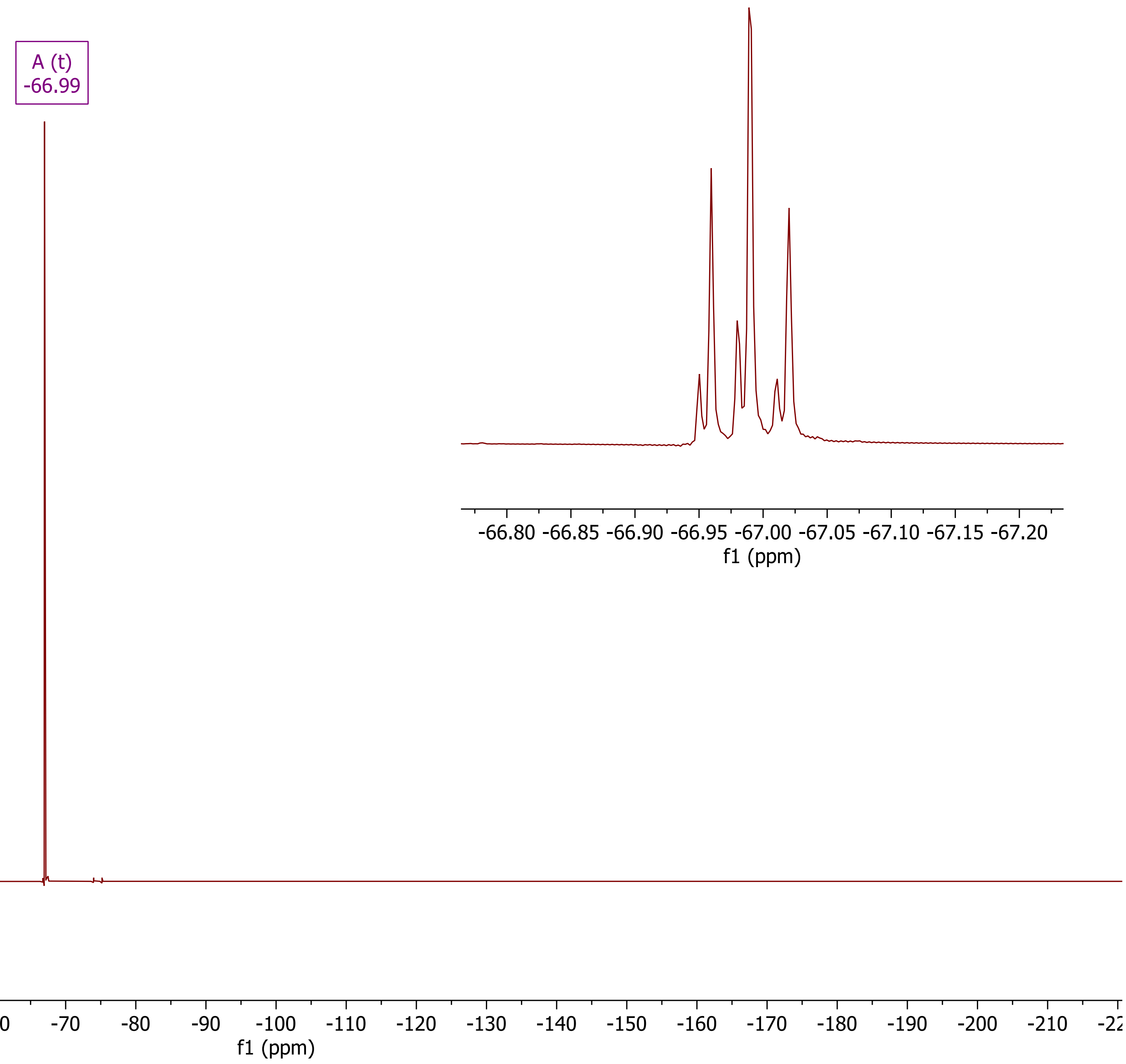

Compound 48, 4-chlorophenyl (5,5,5-trifluoropentyl) phosphate, $^{31}P\ \{^{1}H\}$ - NMR (CD_3CN, 162 MHz)

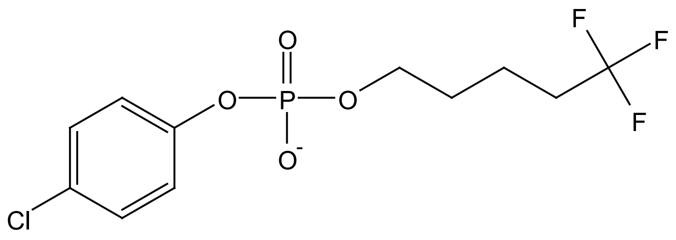

TBA salt

48

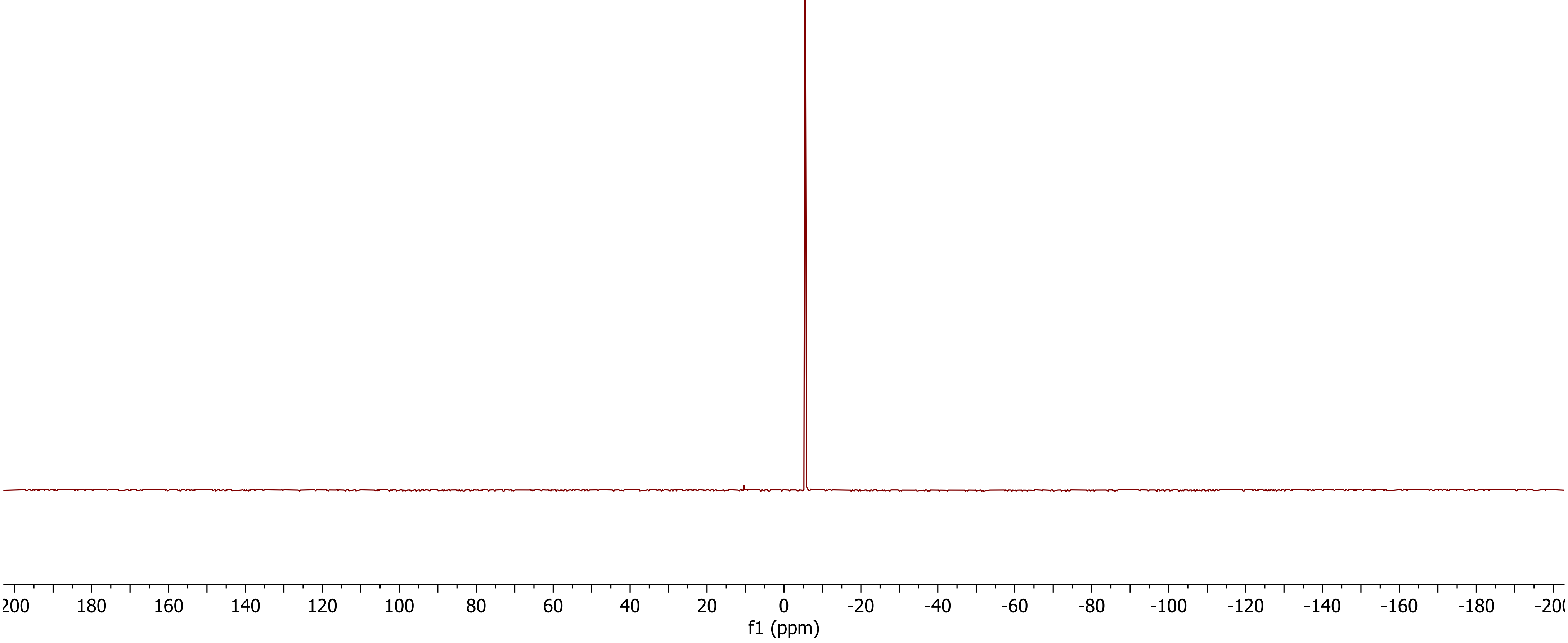

Compound 48, 4-chlorophenyl (5,5,5-trifluoropentyl) phosphate, ^{13}C - NMR (CD_3CN, 101 MHz)

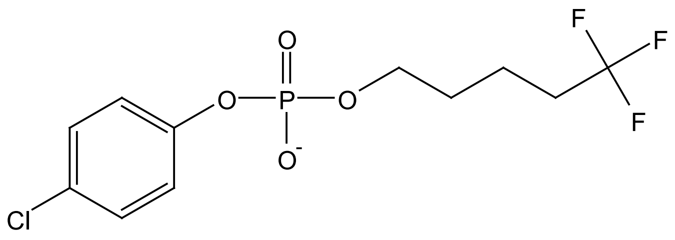

TBA salt

48

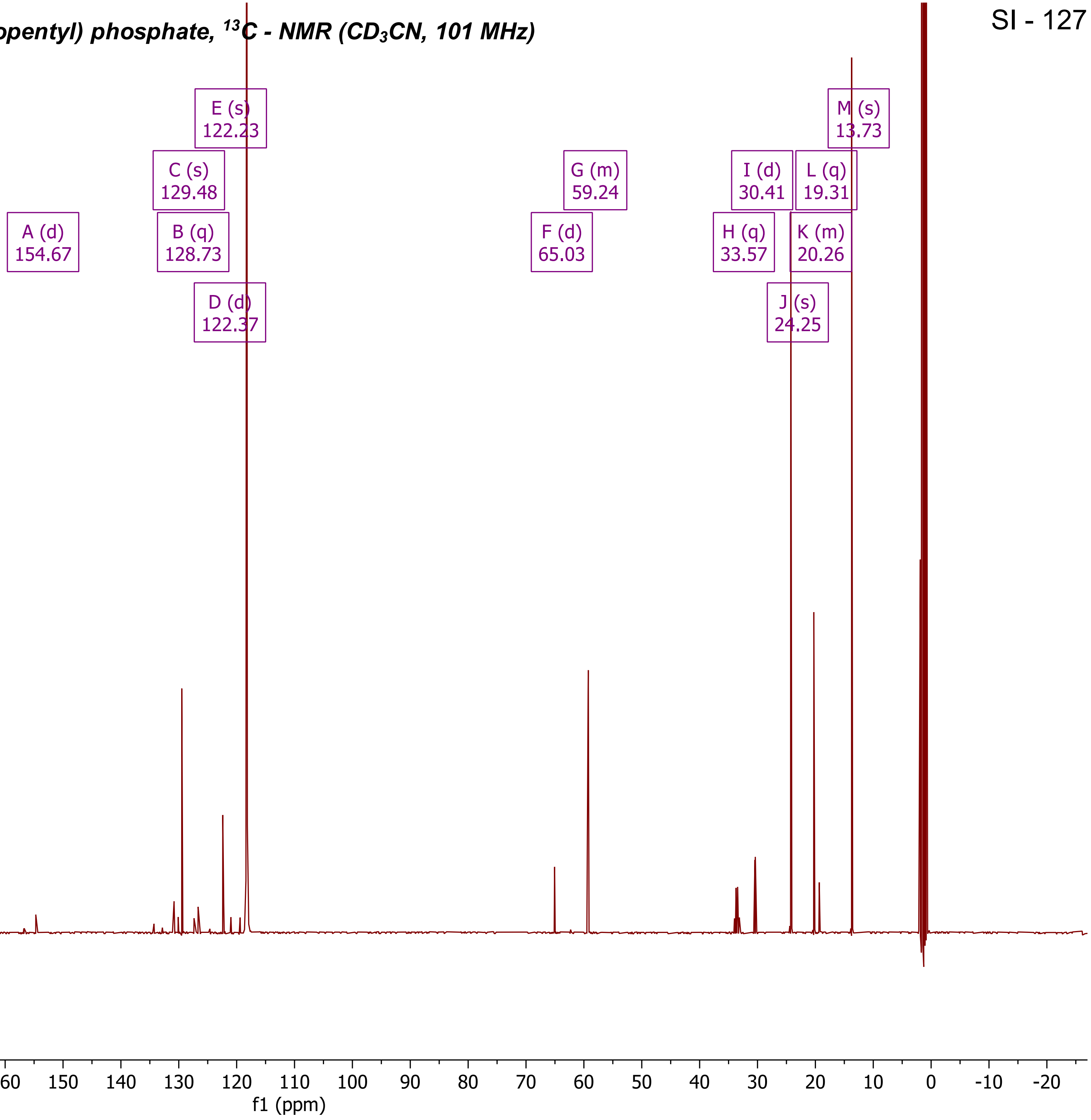

Compound 49, naphthalen-2-yl (5,5,5-trifluoropentyl) phosphate, ^{1}H - NMR (CD_3CN, 400 MHz)

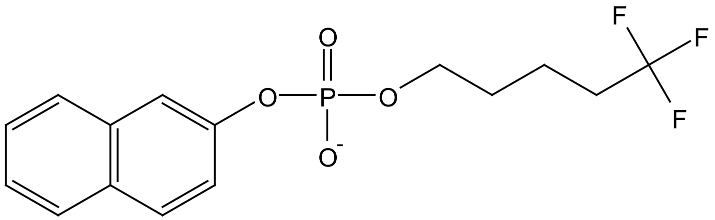

TBA salt

49

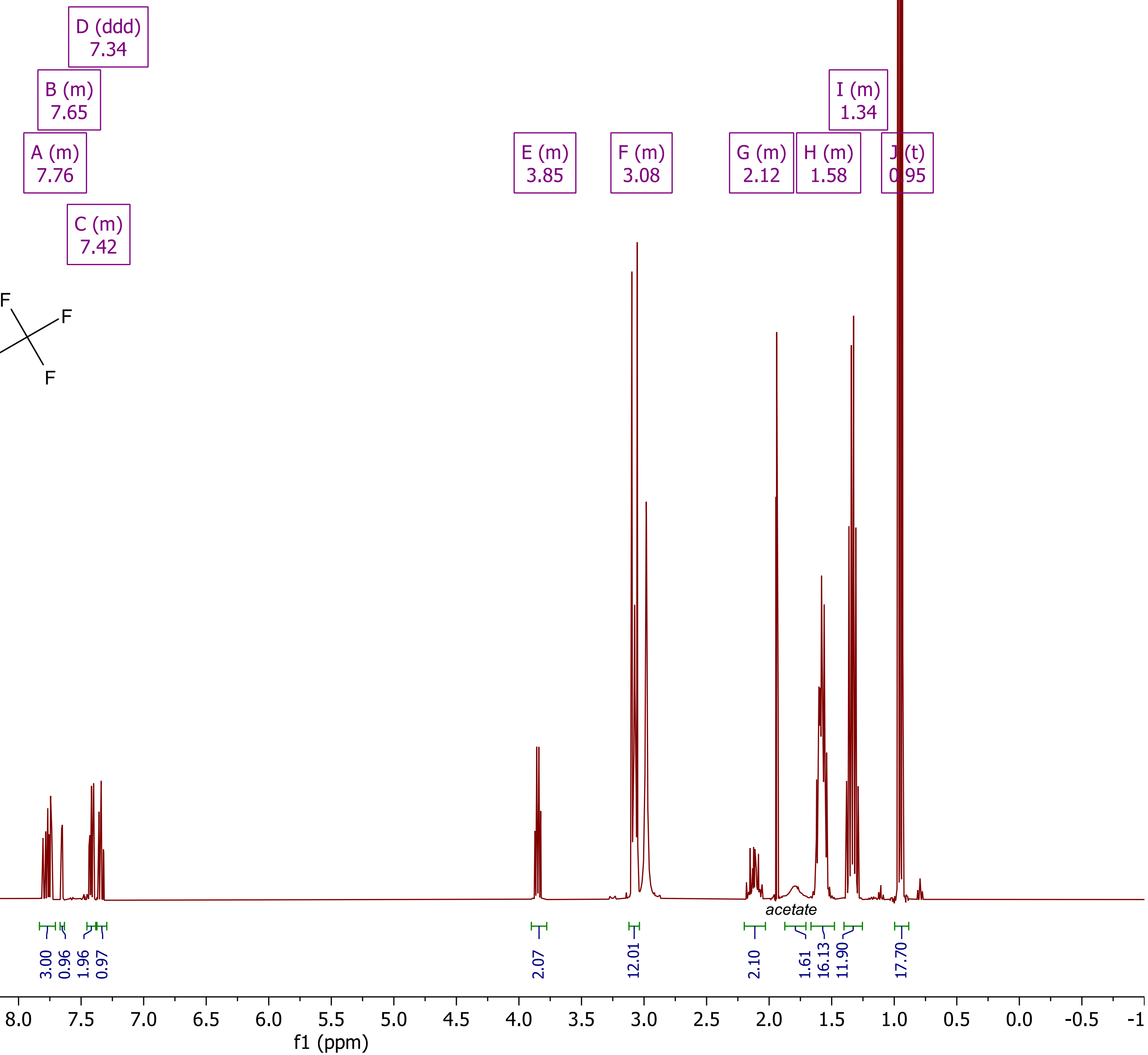

Compound 49, naphthalen-2-yl (5,5,5-trifluoropentyl) phosphate, ^{19}F - NMR (CD_3CN, 377 MHz)

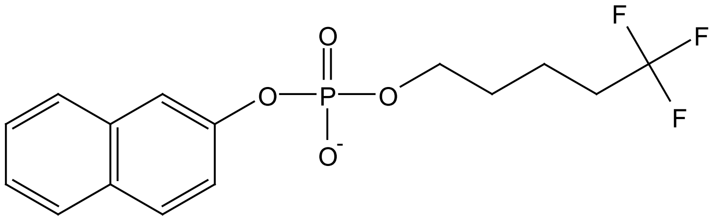

TBA salt

49

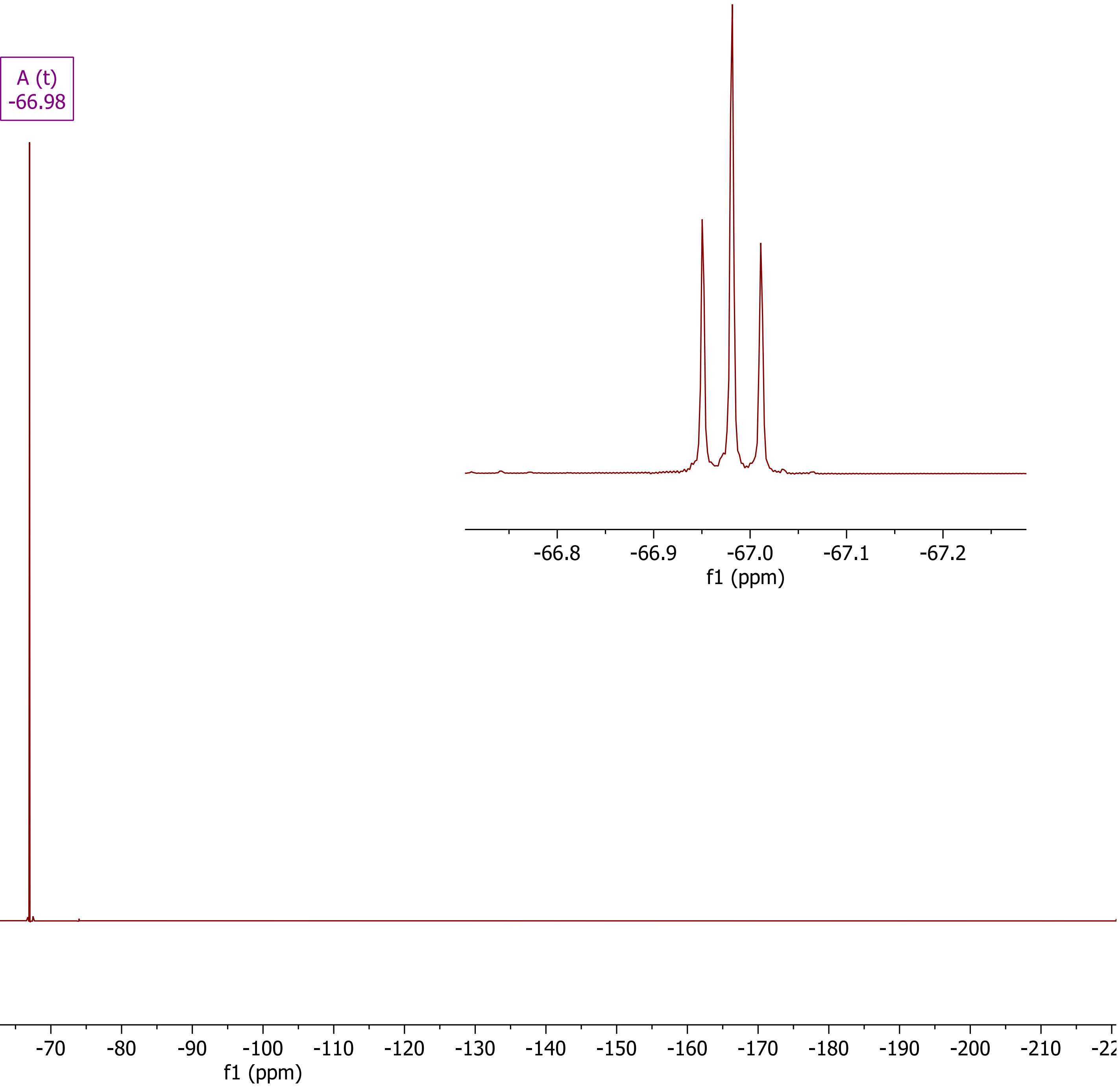

Compound 49, naphthalen-2-yl (5,5,5-trifluoropentyl) phosphate, $^{31}P\{^{1}H\}$ - NMR (CD_3CN, 162 MHz)

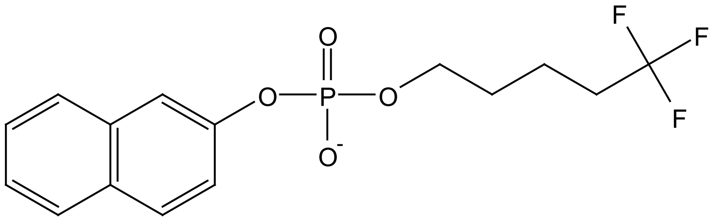

TBA salt

49

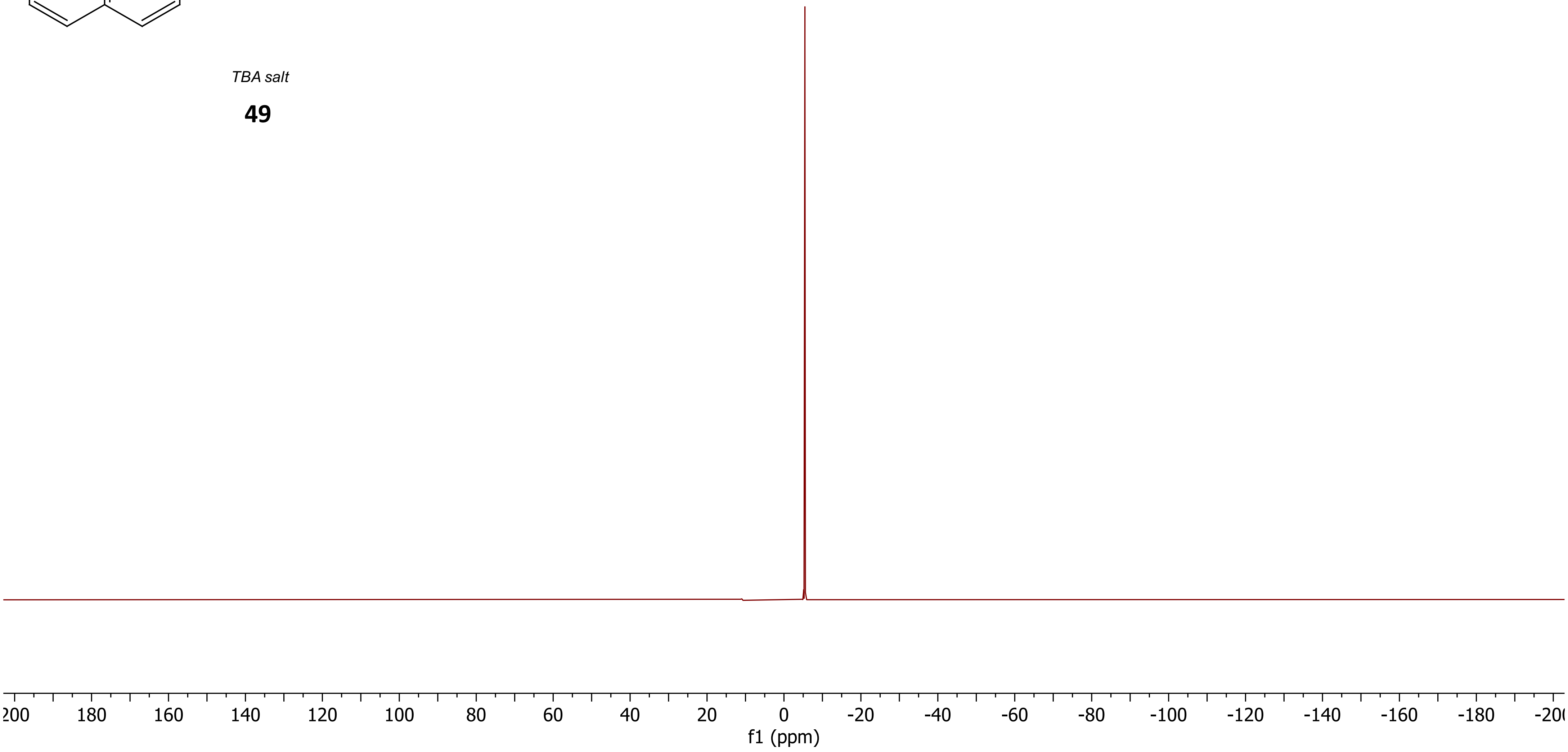

Compound 49, naphthalen-2-yl (5,5,5-trifluoropentyl) phosphate, ^{13}C - NMR (CD_3CN, 101 MHz)

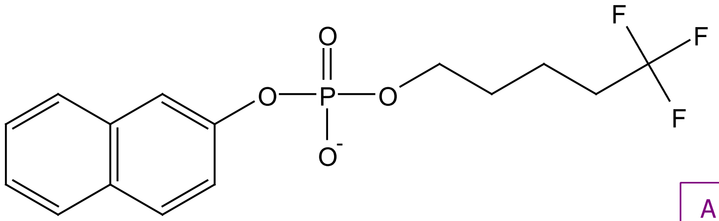

TBA salt

49

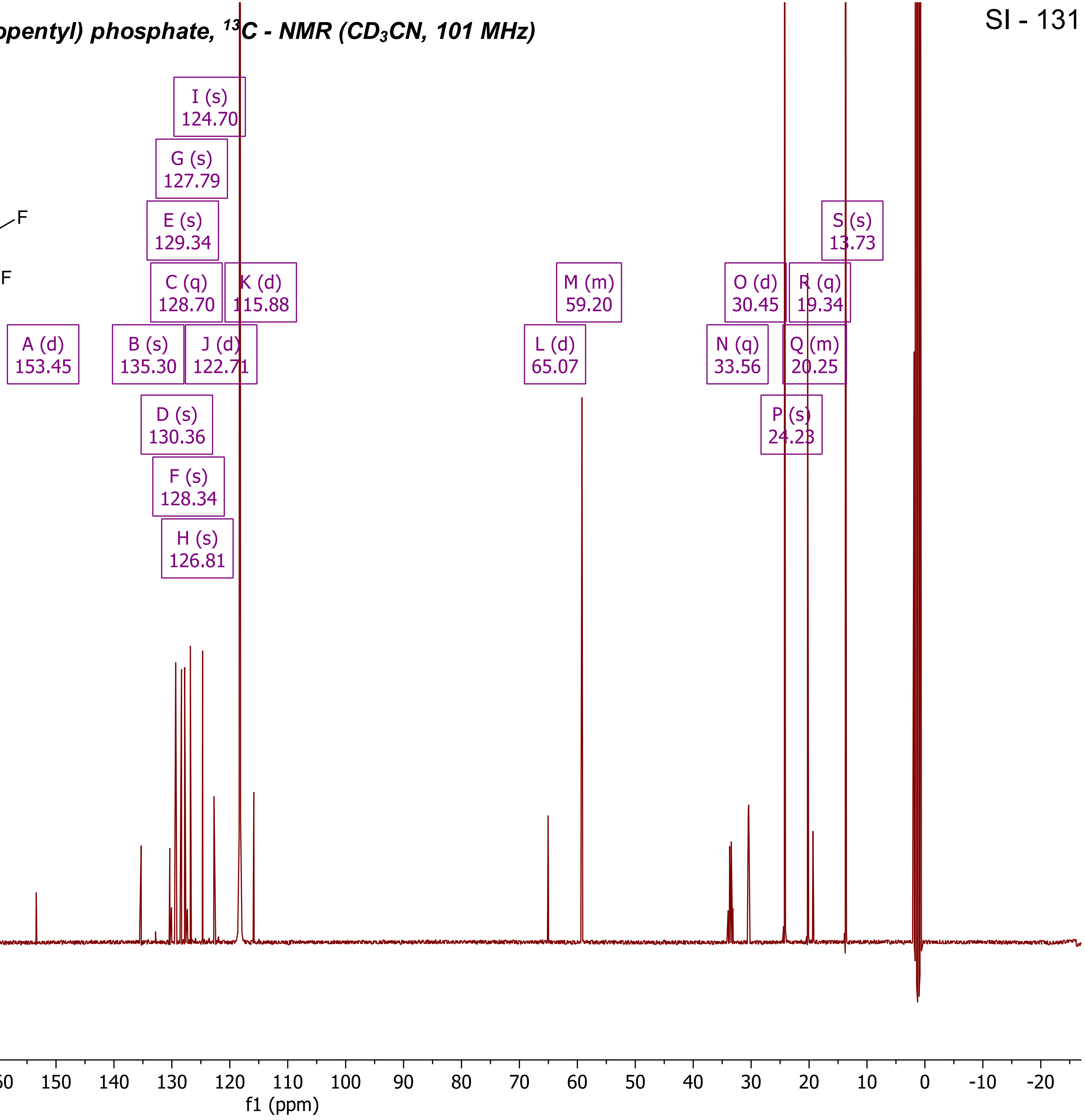

Compound 50, 1H-indol-5-yl (5,5,5-trifluoropentyl) phosphate, ^{1}H - NMR (CD_3CN, 400 MHz)

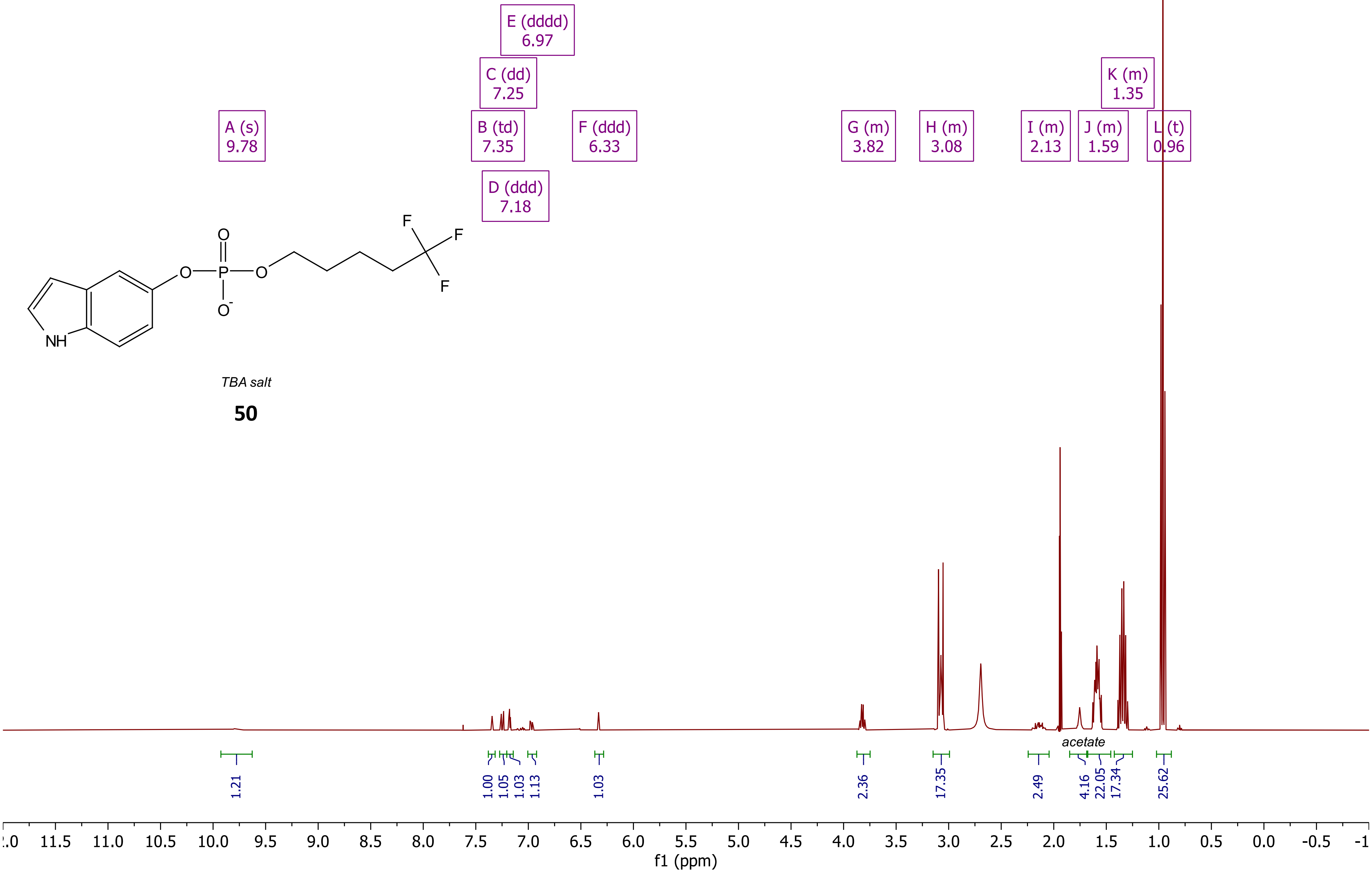

Compound 50, 1H-indol-5-yl (5,5,5-trifluoropentyl) phosphate, ^{19}F - NMR (CD_3CN, 377 MHz)

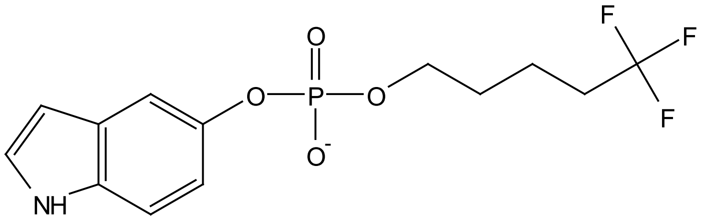

TBA salt

50

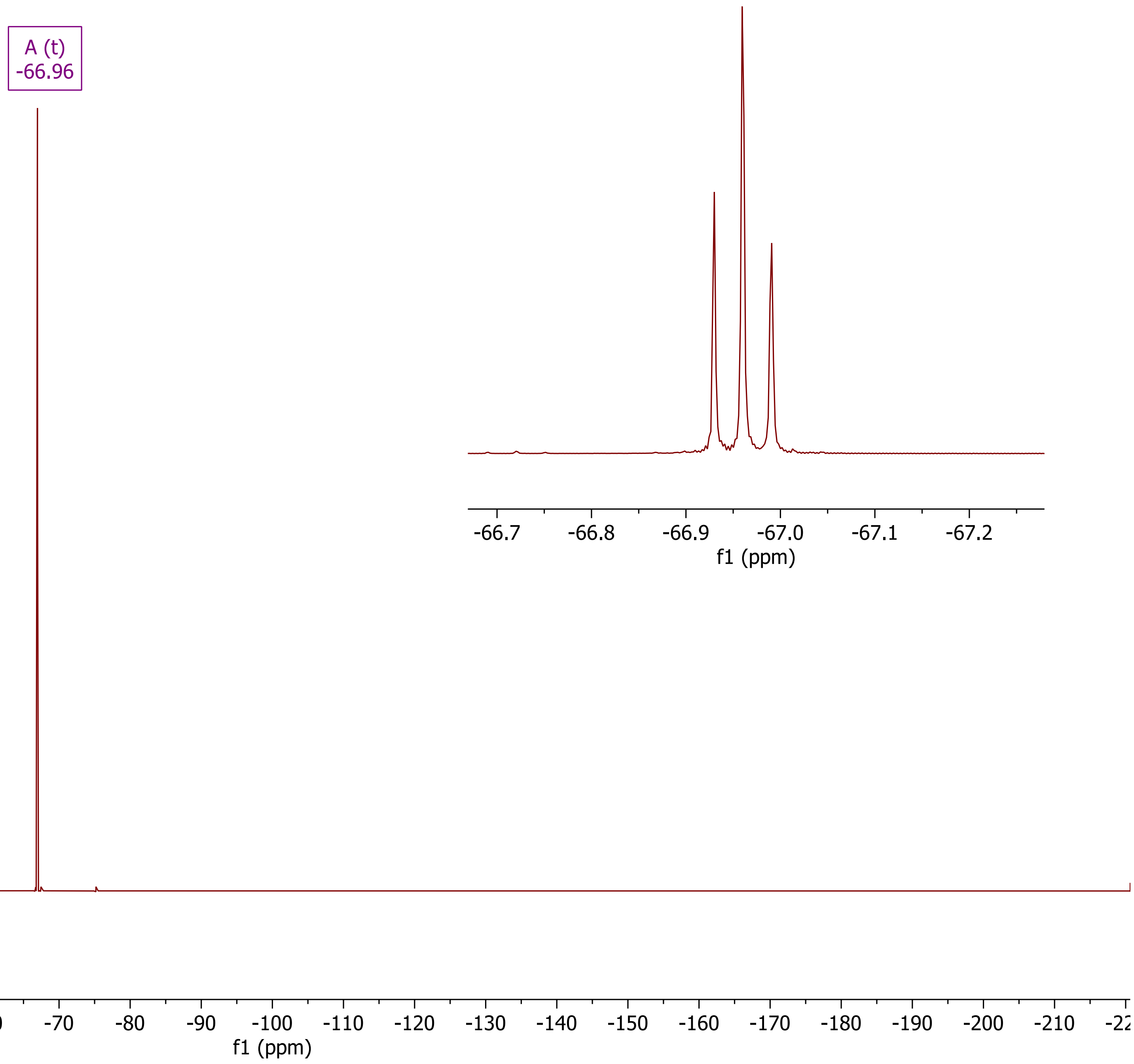

Compound 50, 1H-indol-5-yl (5,5,5-trifluoropentyl) phosphate, $^{31}P\{^1H\}$ - NMR (CD_3CN, 162 MHz)

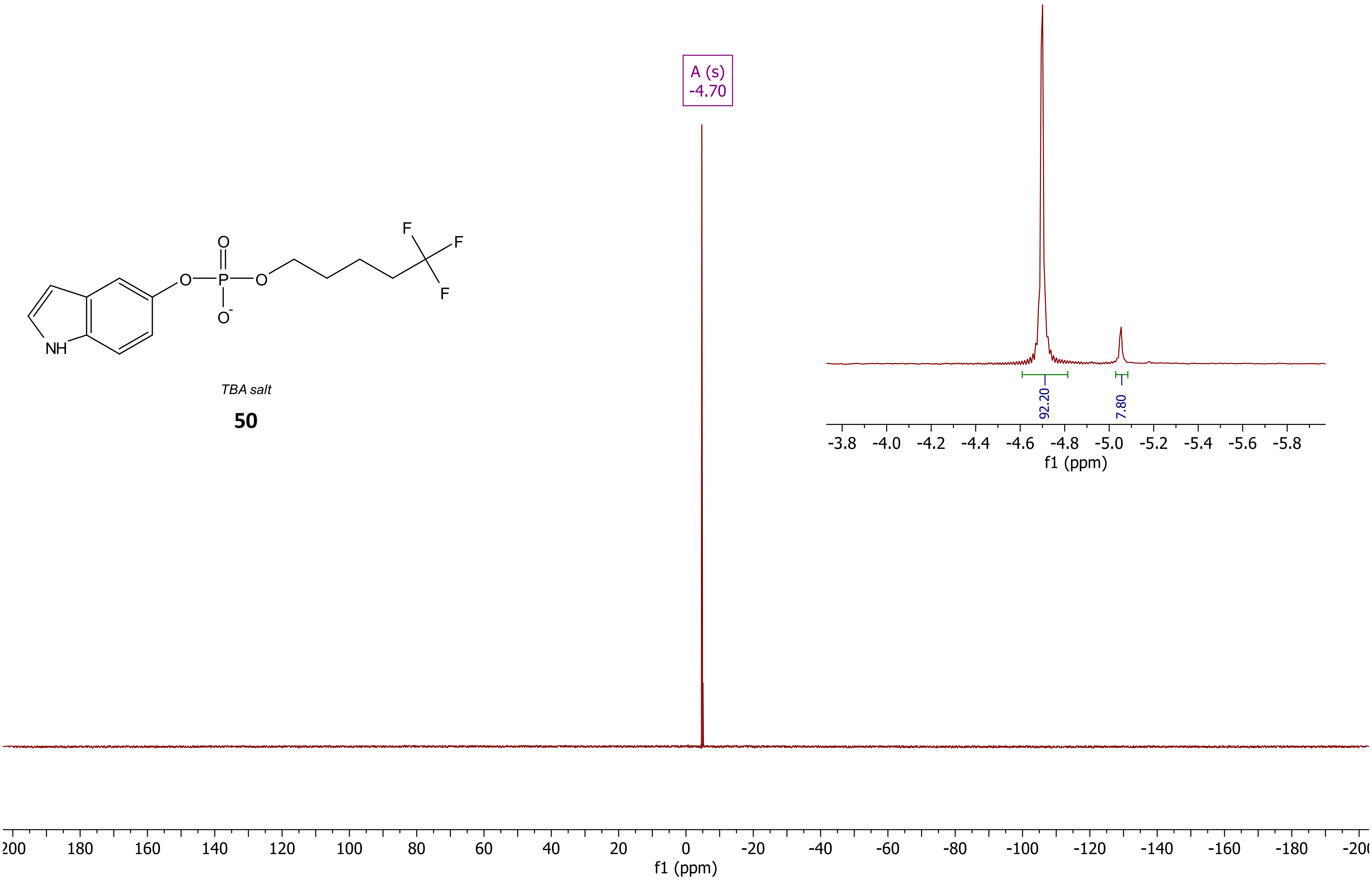

TBA salt

50

Compound 50, 1H-indol-5-yl (5,5,5-trifluoropentyl) phosphate, ^{13}C - NMR (CD_3CN, 101 MHz)

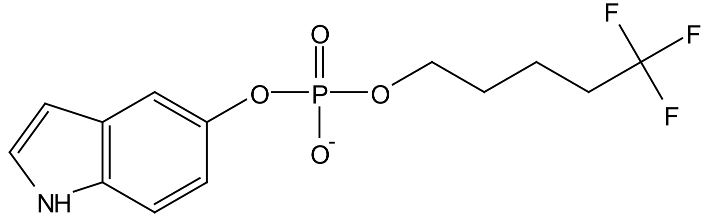

50

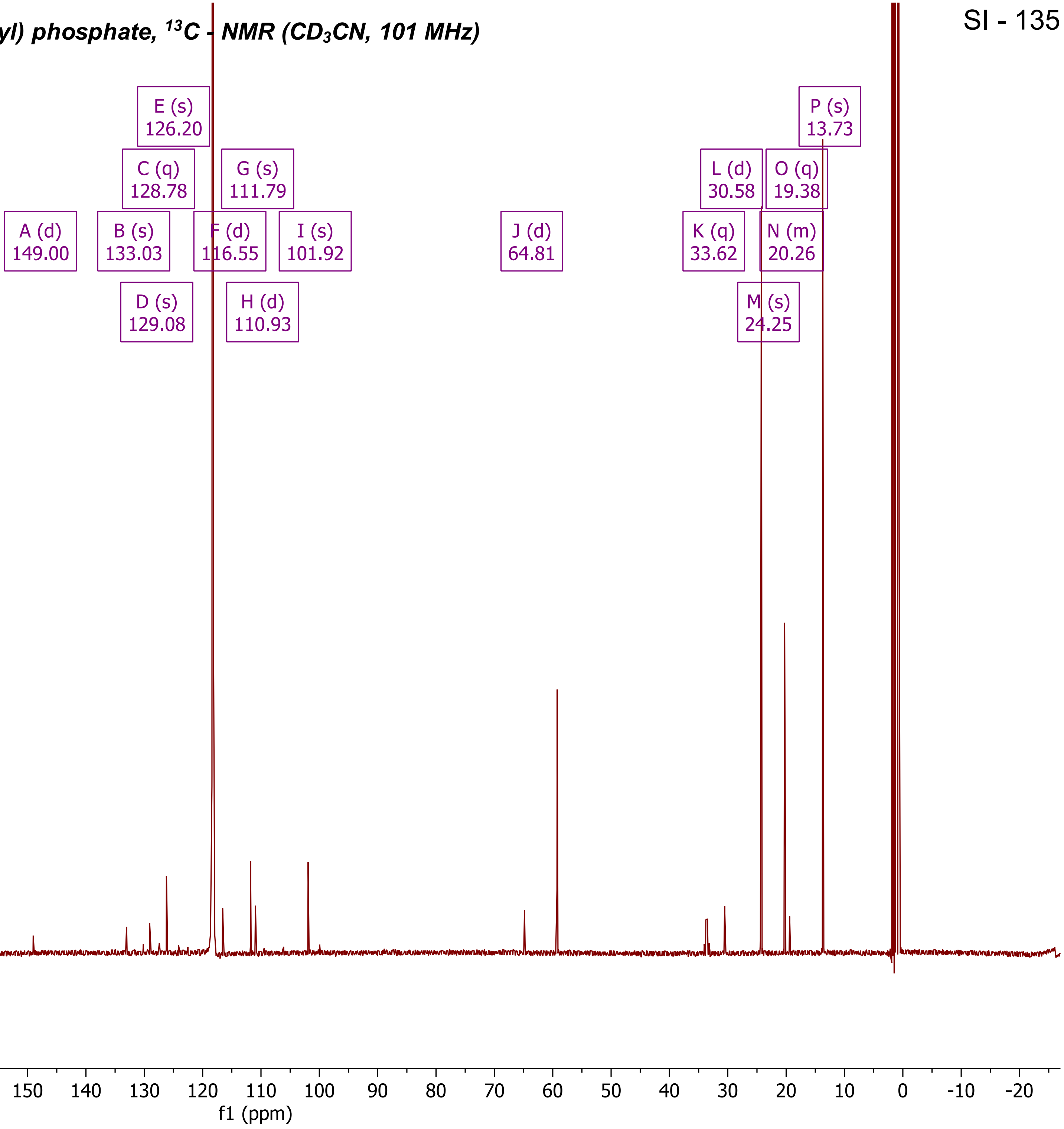

Compound 51, pyren-2-yl (5,5,5-trifluoropentyl) phosphate, ^1H - NMR (CD_3CN, 400 MHz)

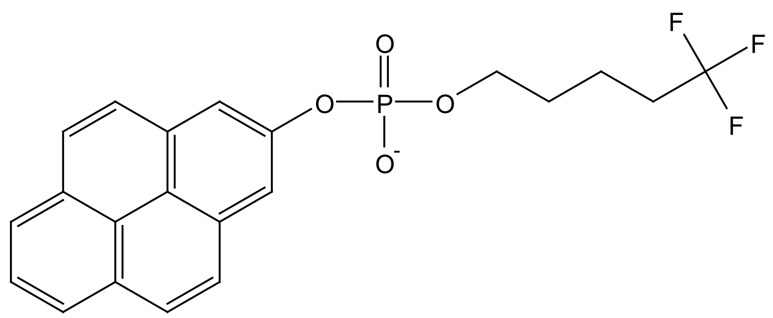

TBA salt

51

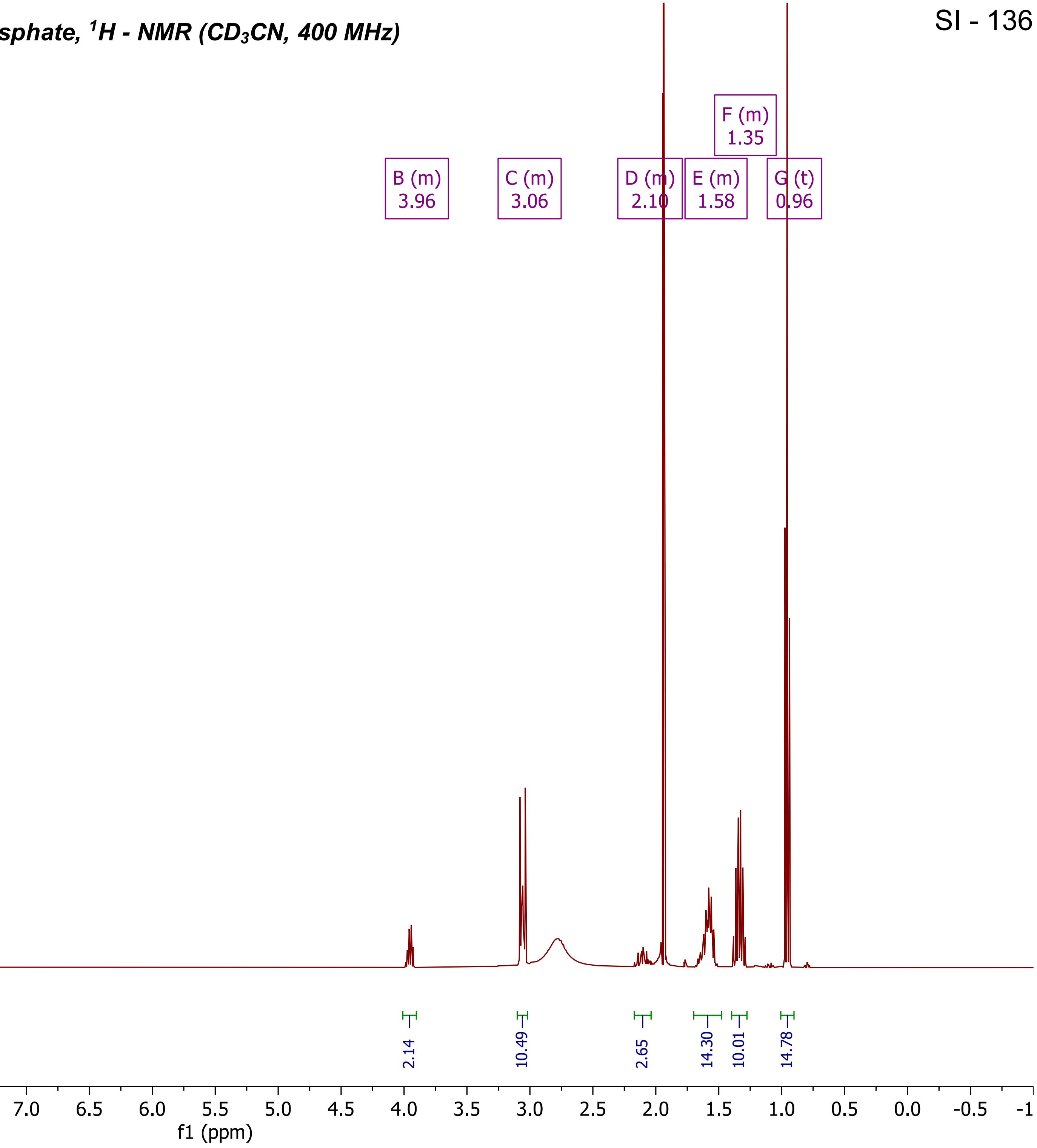

Compound 51, pyren-2-yl (5,5,5-trifluoropentyl) phosphate, ^{19}F - NMR (CD_3CN, 377 MHz)

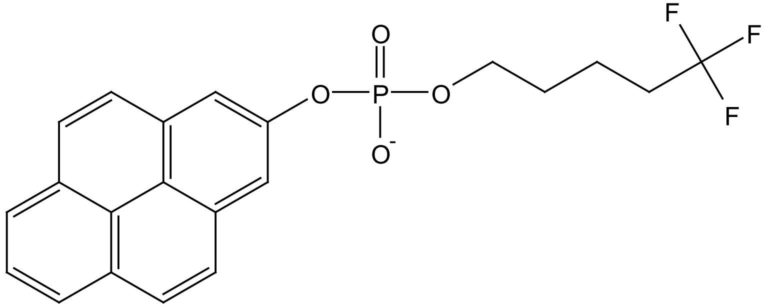

TBA salt

51

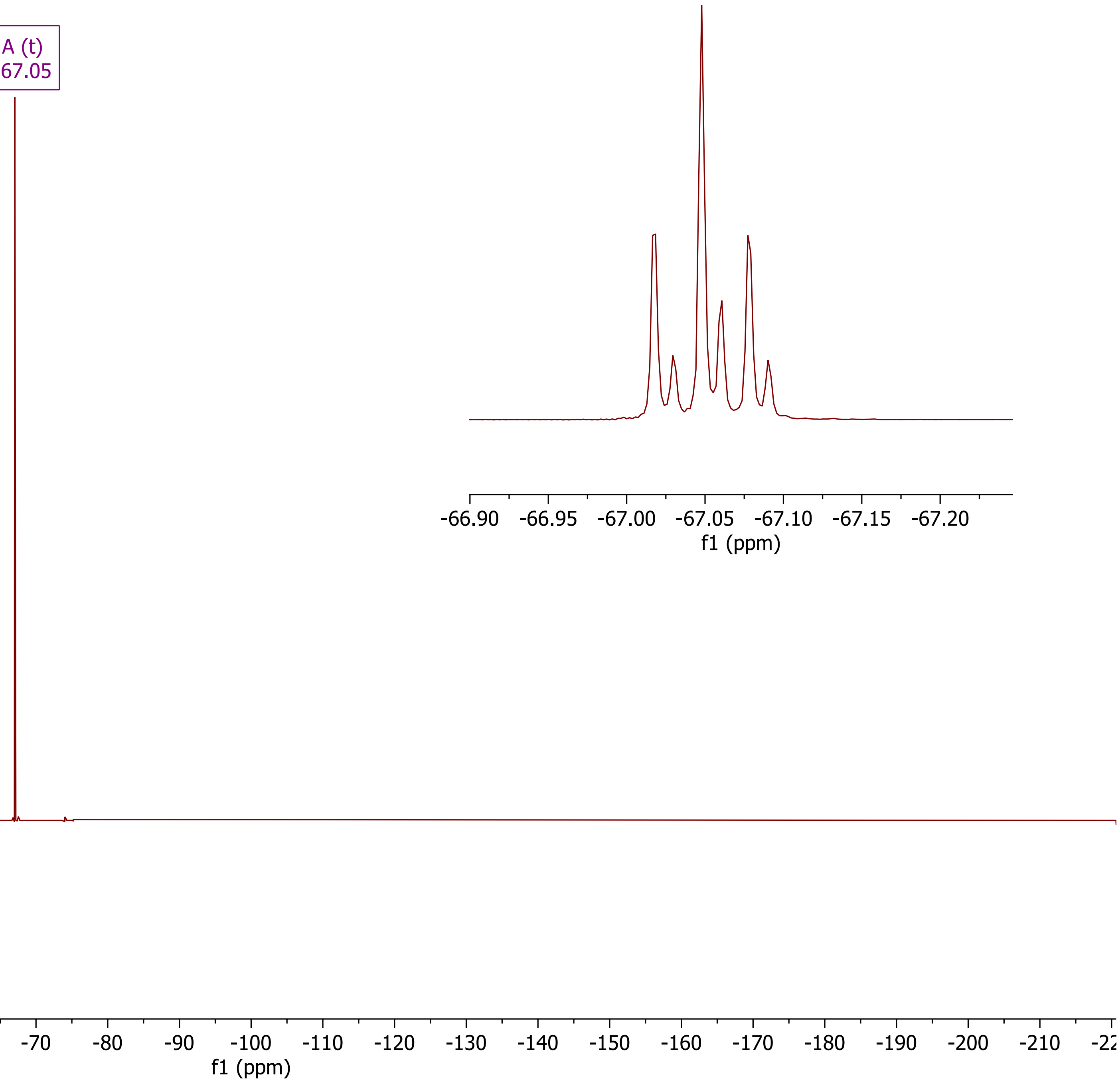

Compound 51, pyren-2-yl (5,5,5-trifluoropentyl) phosphate, $^{31}P\{^1H\}$ - NMR (CD_3CN, 162 MHz)

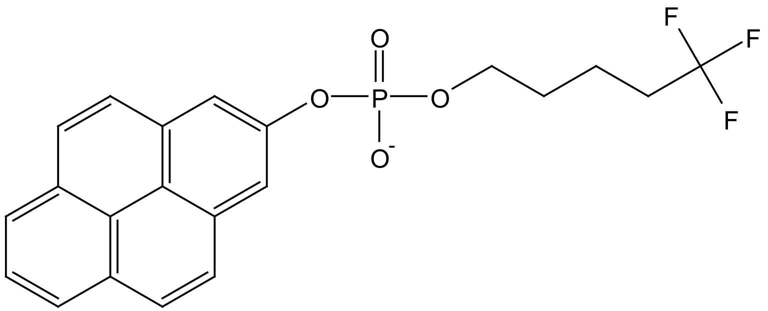

TBA salt

51

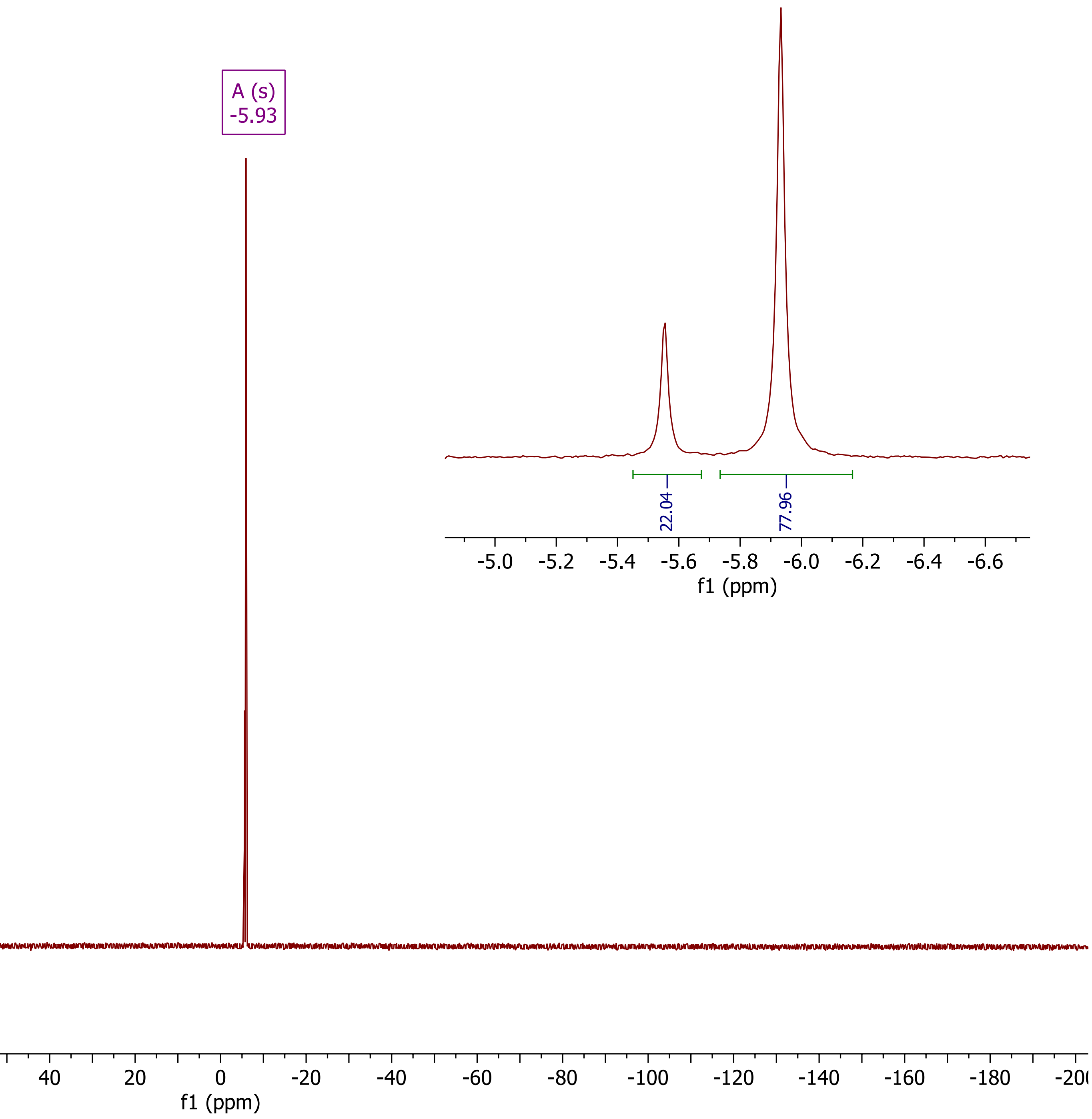

Compound 51, pyren-2-yl (5,5,5-trifluoropentyl) phosphate, ^{13}C - NMR (CD_3CN, 101 MHz)

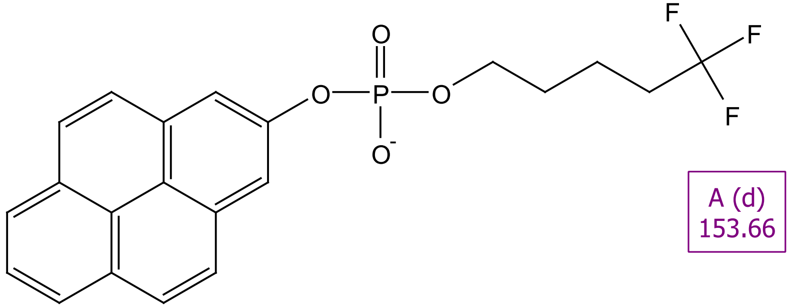

TBA salt

51

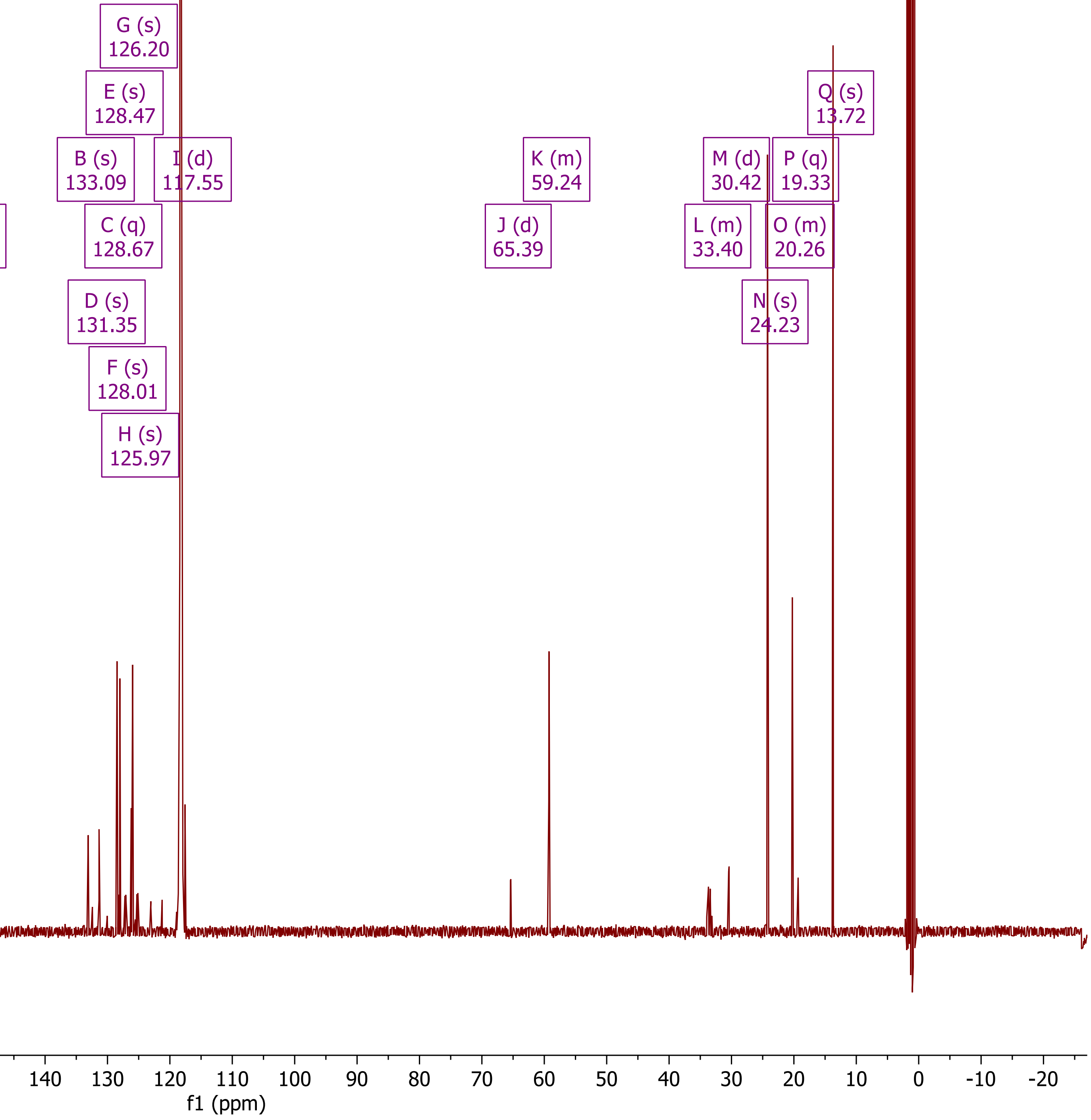

Compound 52, Diphenylphosphate, ¹H - NMR (D_2O, 400 MHz)

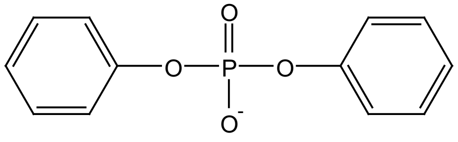

TBA / TEA - salt

52

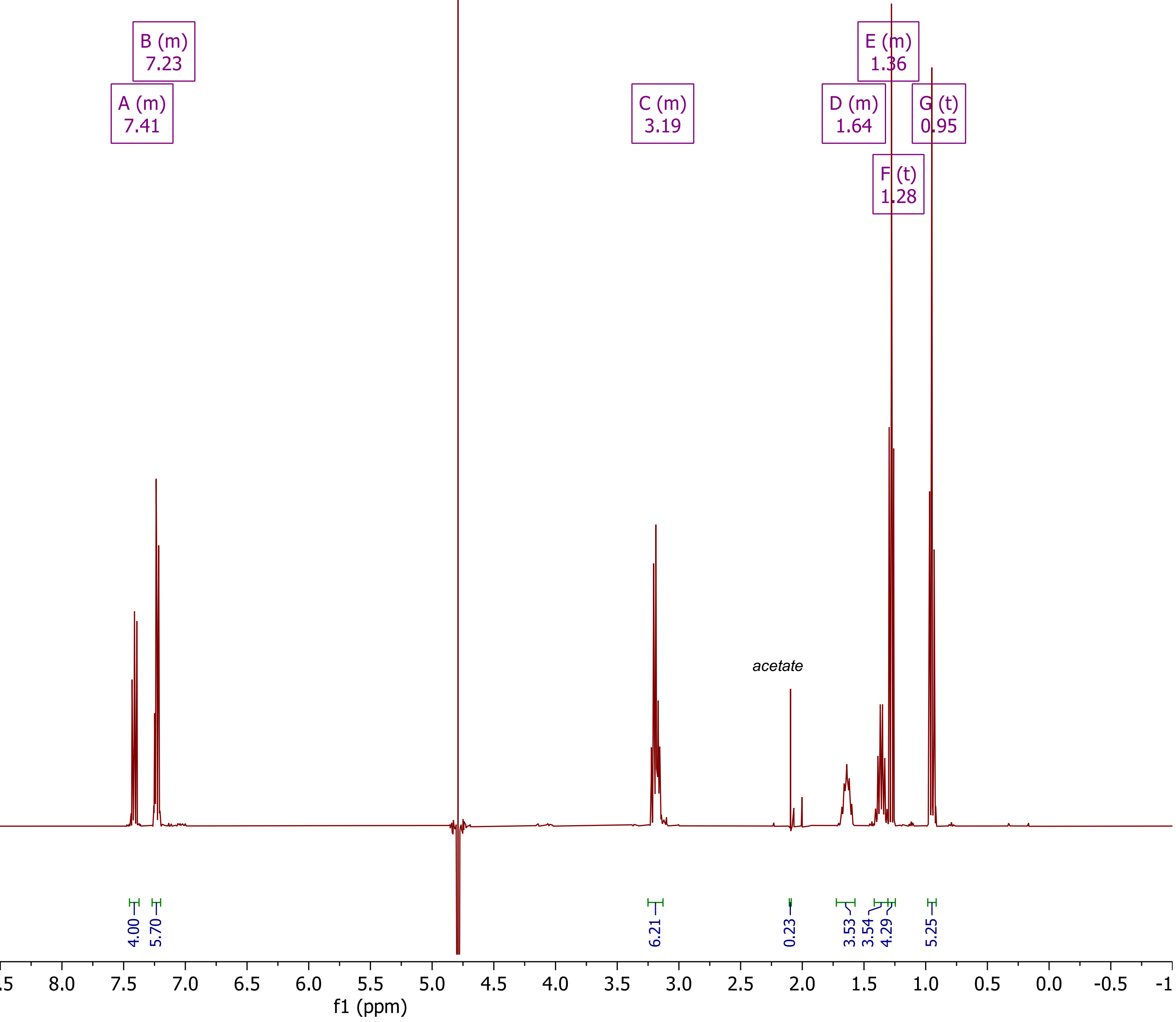

Compound 52, Diphenylphosphate, $^{31}P\{^1H\}$ - NMR (D2O, 162 MHz)

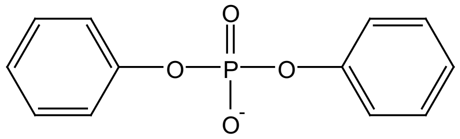

TBA / TEA - salt

52

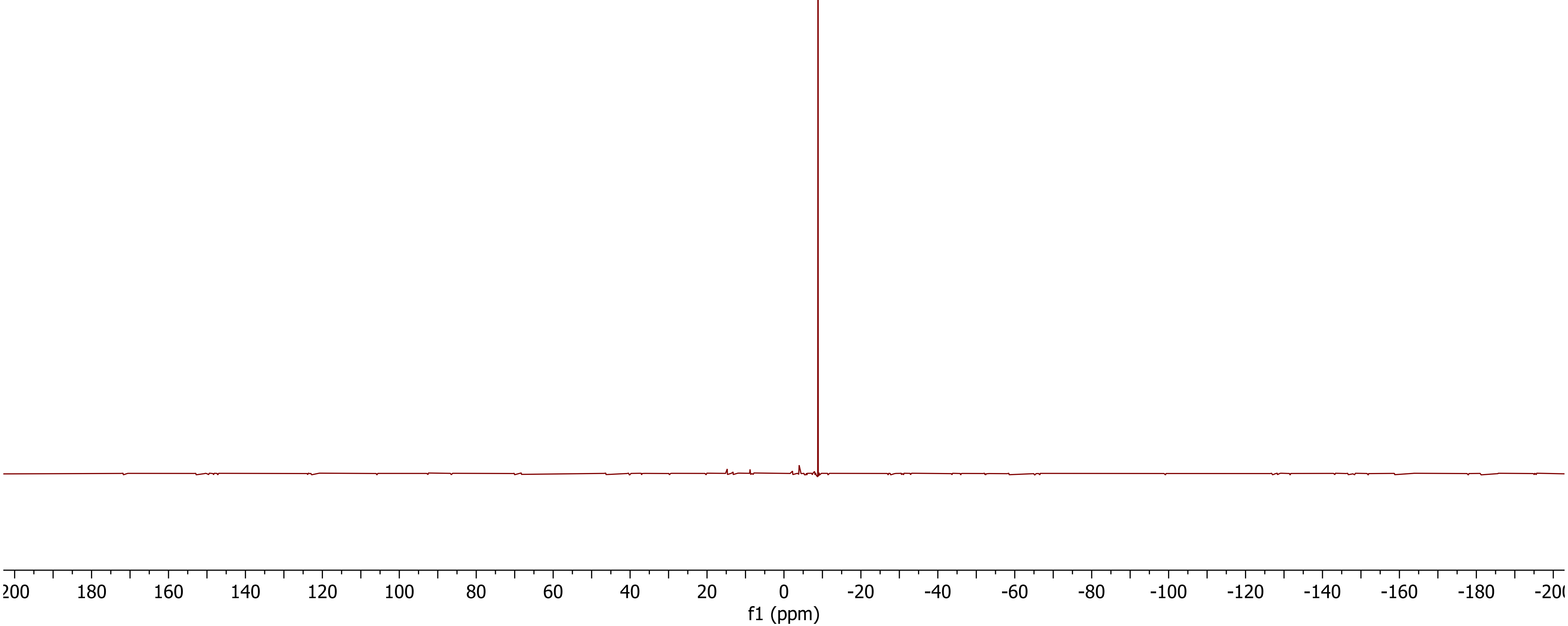

Compound 52, Phenylphenylphosphonate, ^{13}C - NMR (D2O, 101 MHz)

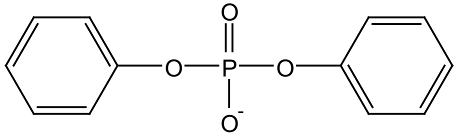

TBA / TEA - salt

52

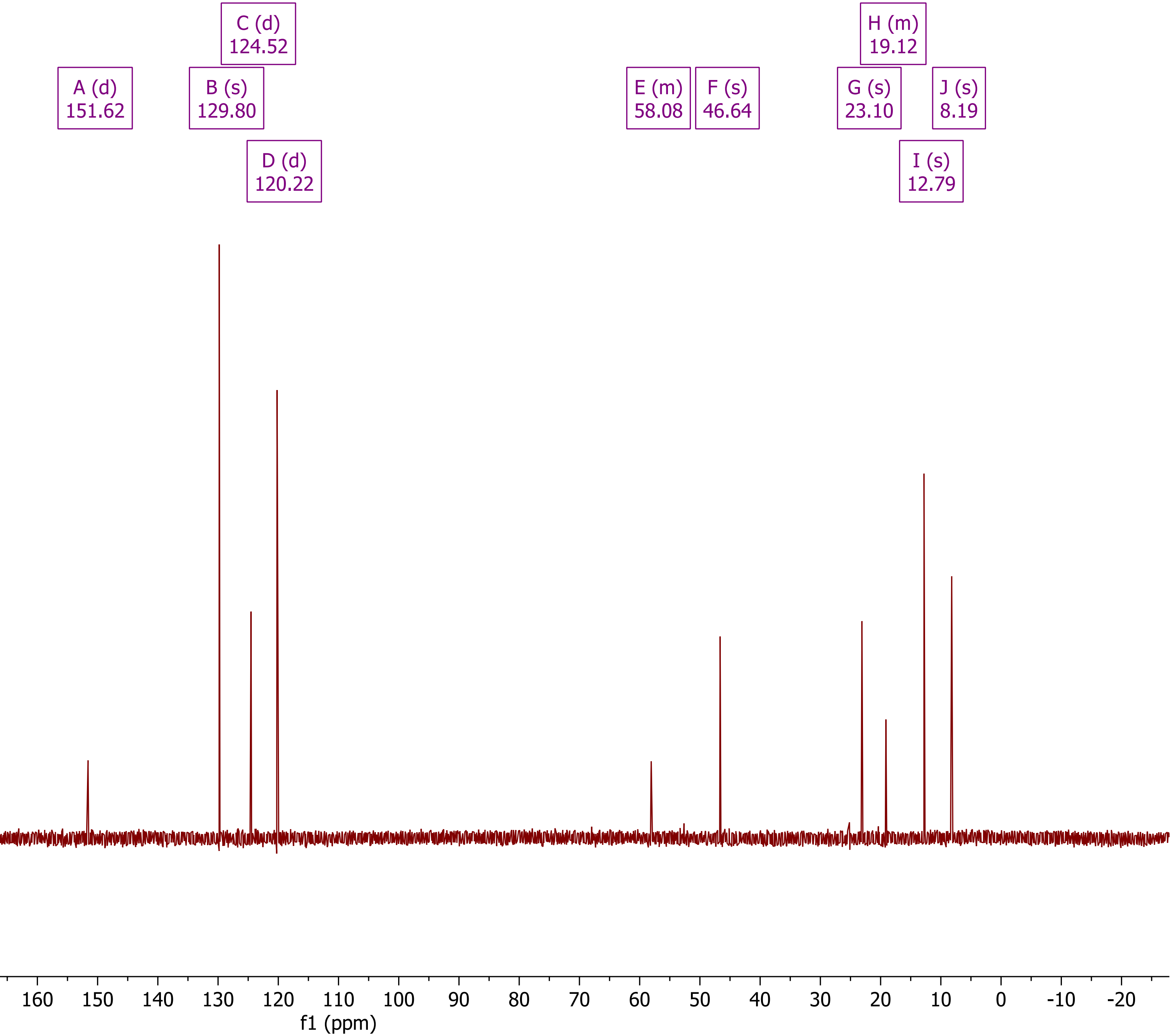

Compound 53, Pentyl-phenylphosphate, ^{1}H - NMR (MeCN-d3, 400 MHz)

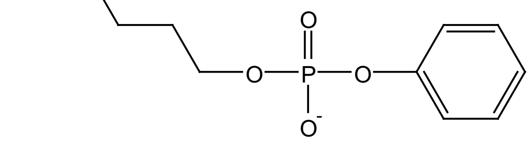

TBA - salt

53

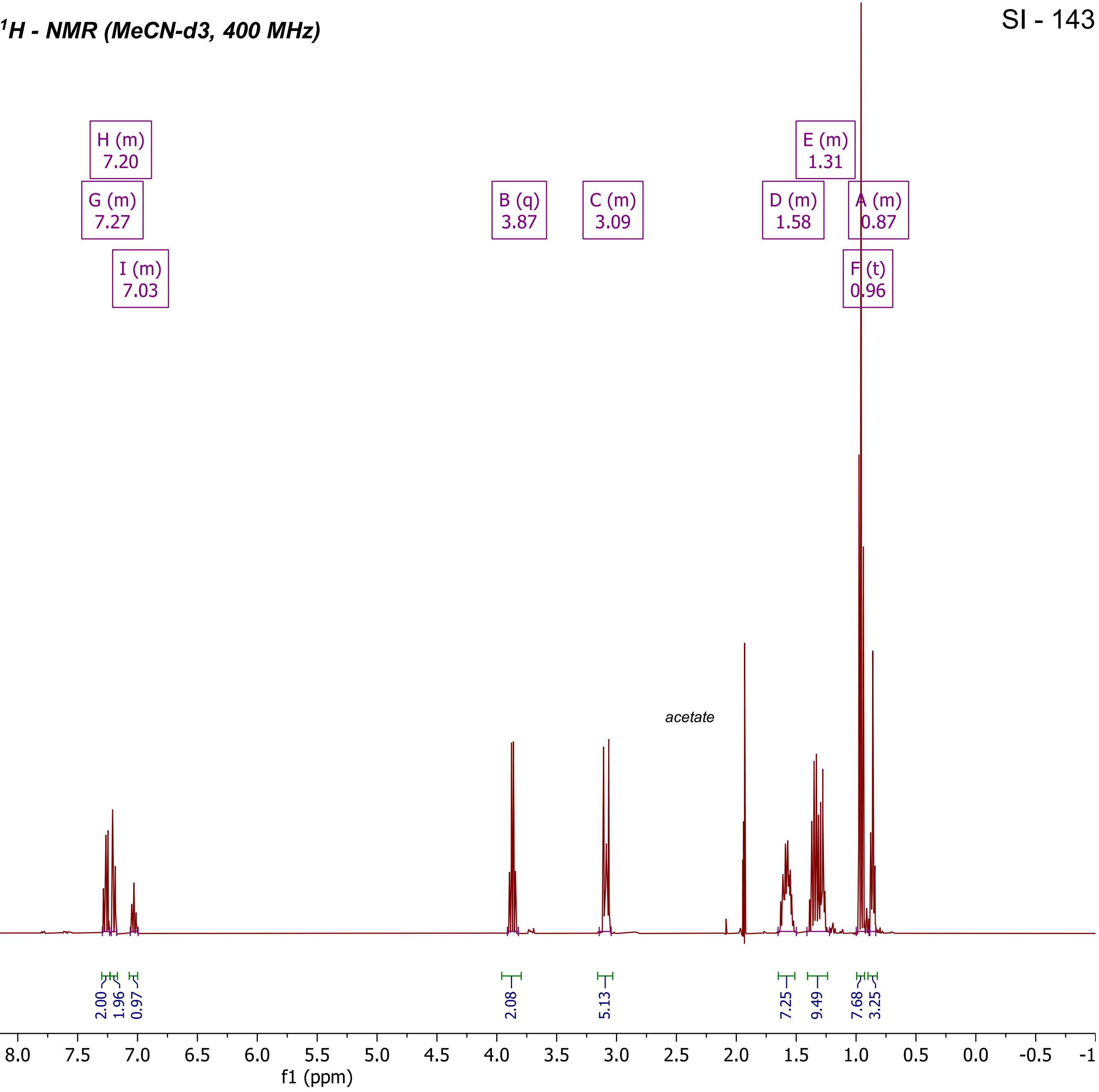

Compound 53, Pentyl-phenylphosphate, ^{31}P {^{1}H} - NMR (MeCN-d3, 162 MHz)

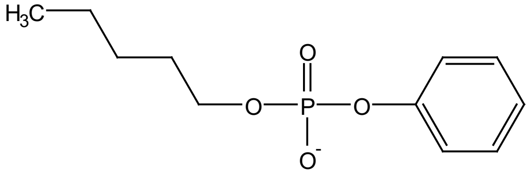

TBA - salt

53

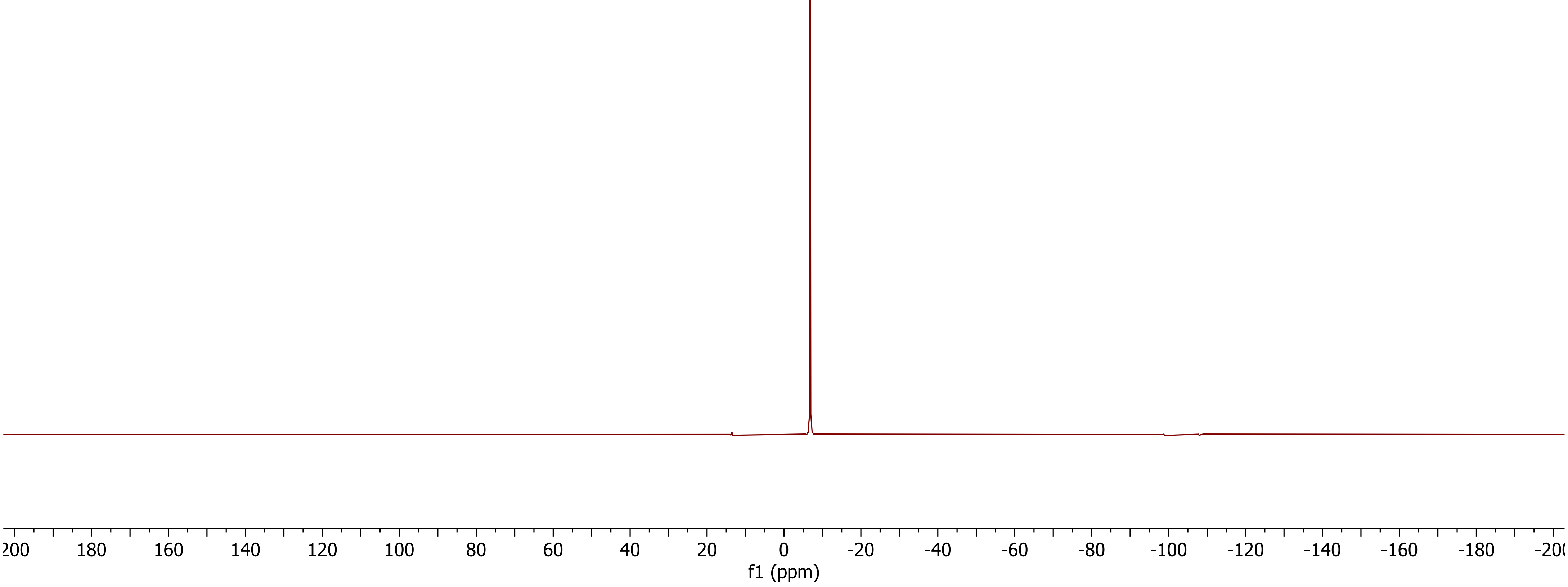

Compound 53, Pentyl-phenylphosphate, $^{13}C\{^{1}H\}$ - NMR (MeCN-d3, 101 MHz)

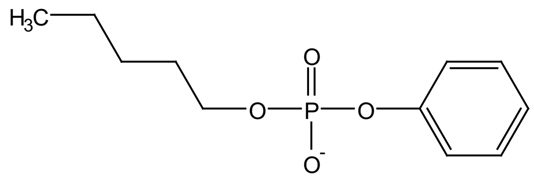

TBA - salt

53

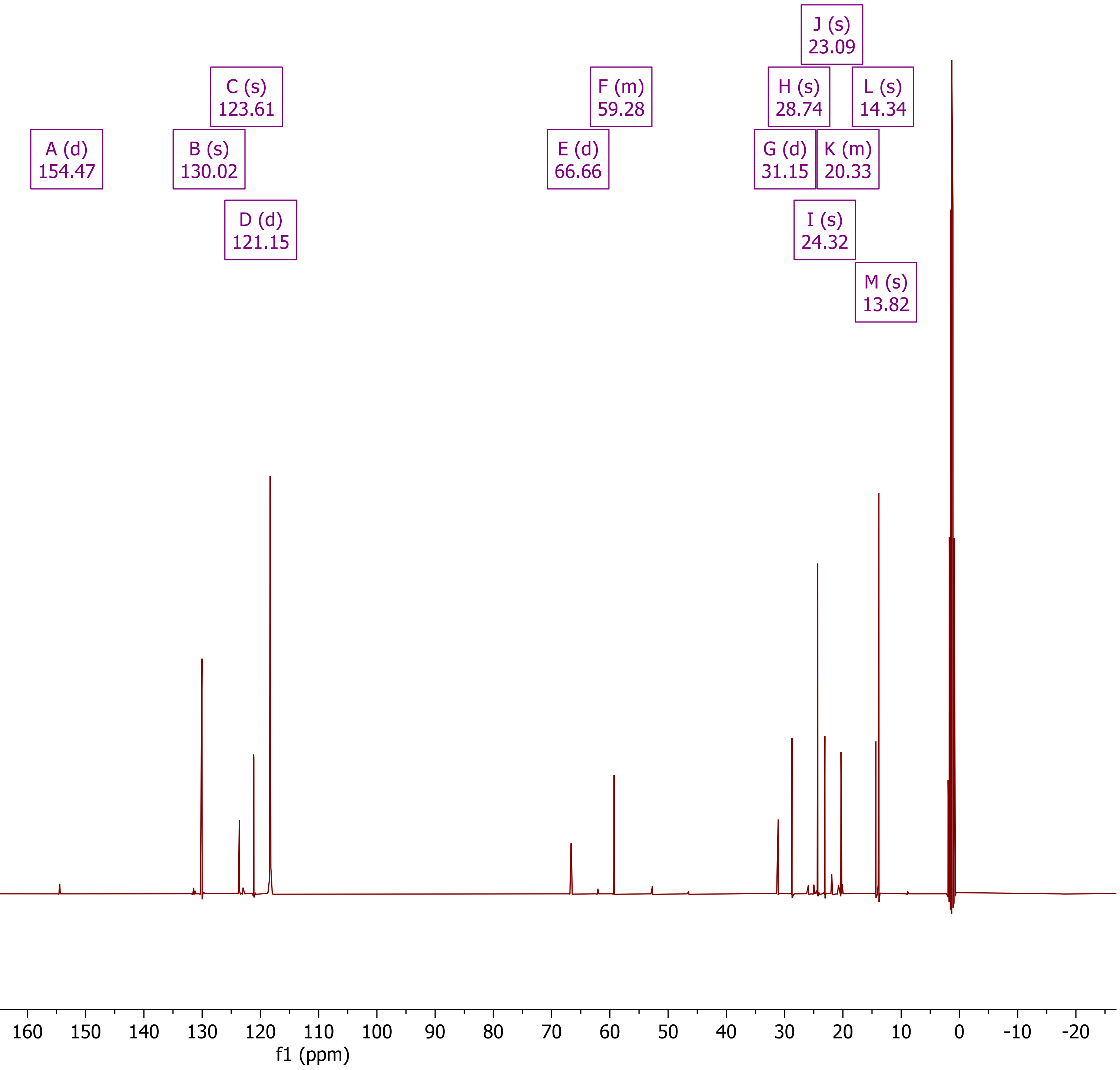

Compound 54, Phenylphenylphosphonate, ^{1}H - NMR (D_2O, 400 MHz)

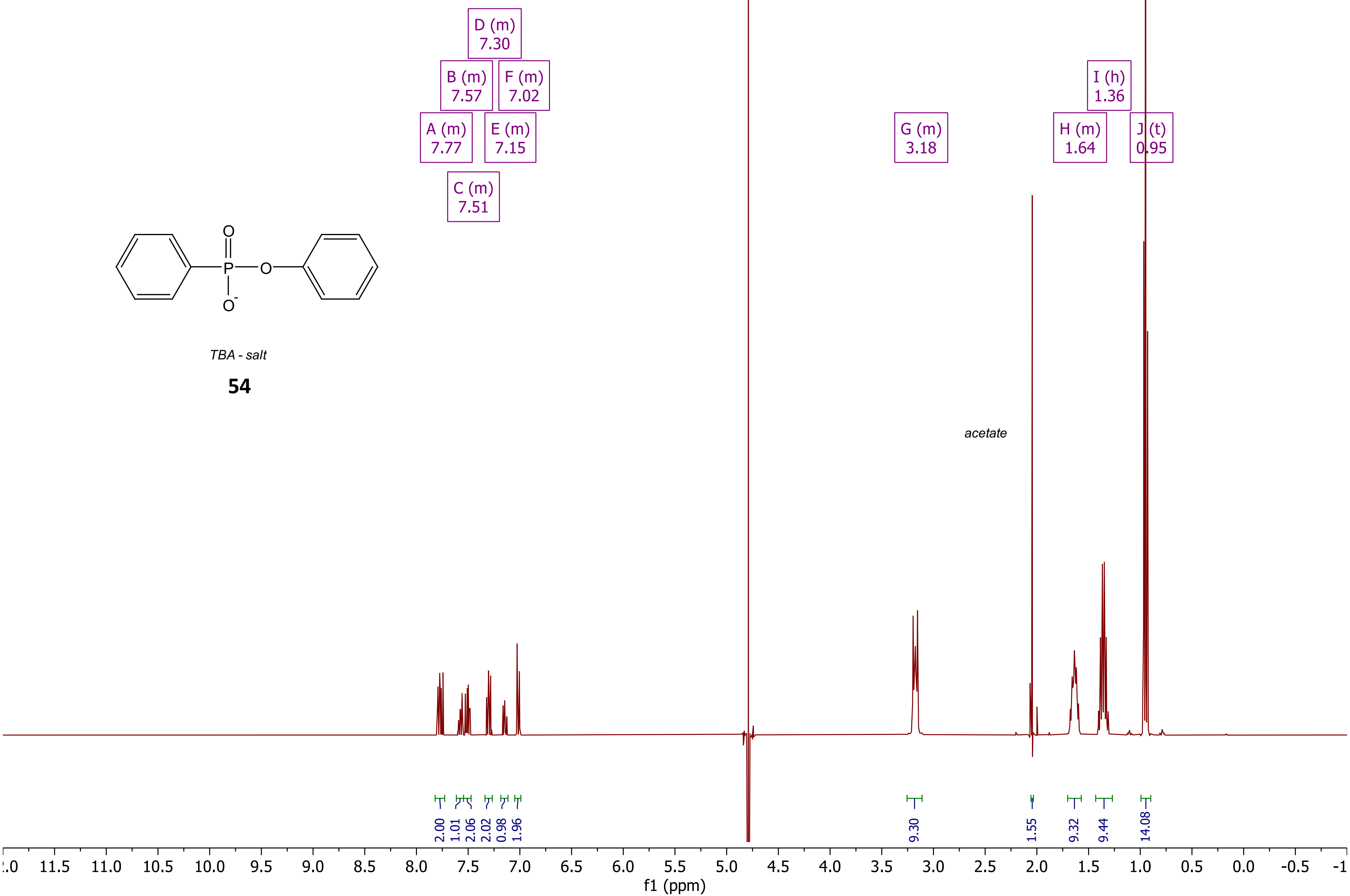

Compound 54, Phenylphenylphosphonate, $^{31}P\ \{^{1}H\}$ - NMR (D2O, 162 MHz)

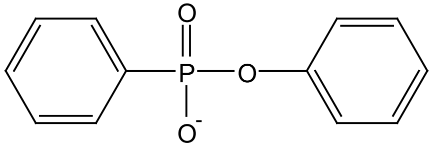

TBA - salt

54

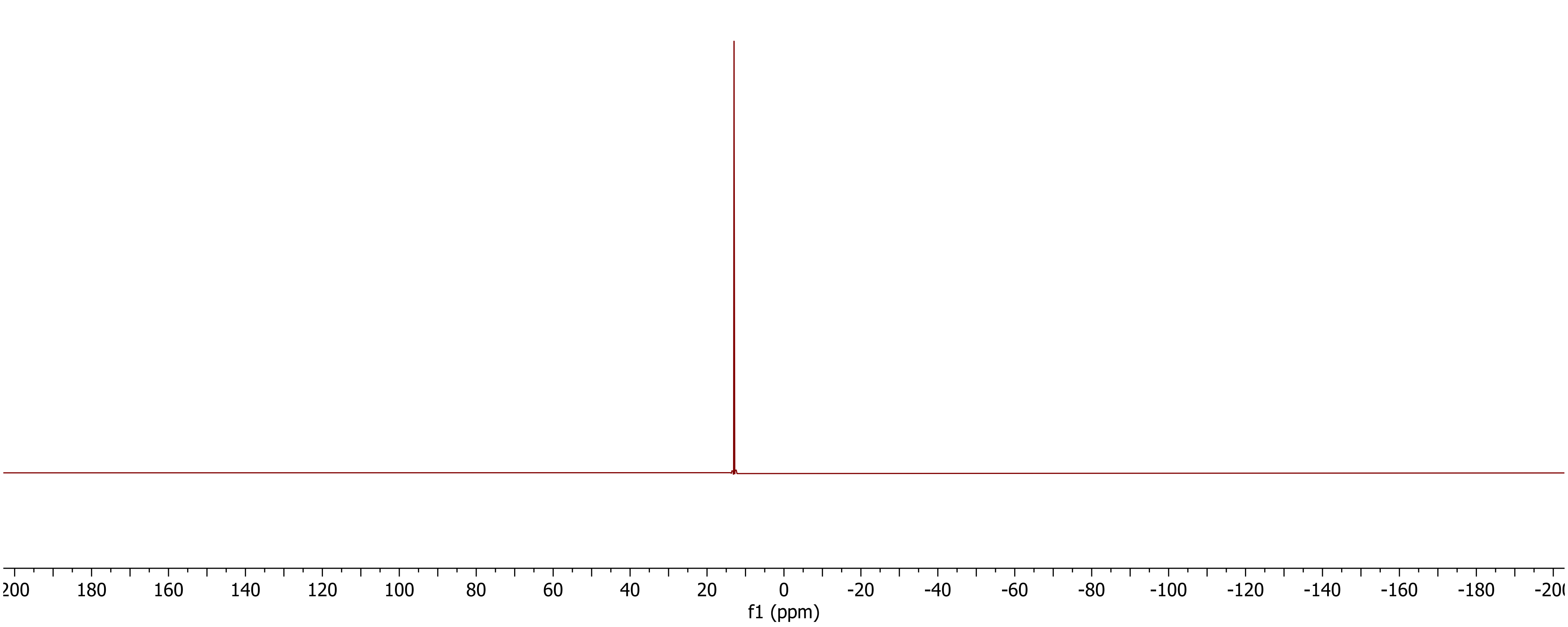

Compound 54, Phenylphenylphosphonate, ^{13}C - NMR (D2O, 101 MHz)

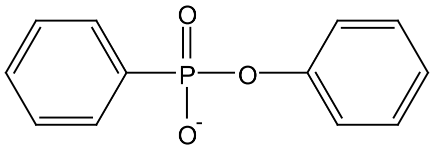

54

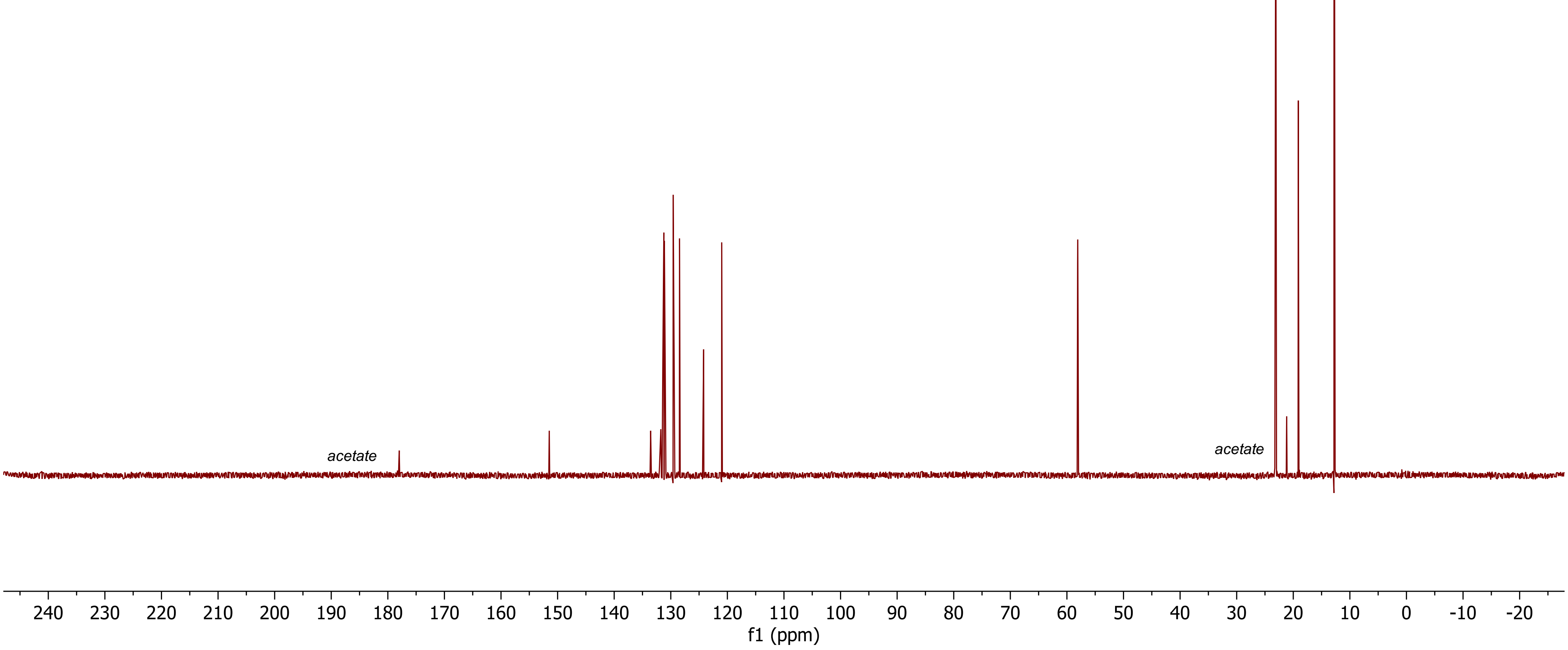

Compound 55, Pent-4-yn-1-yl-phenylphosphate, ^{1}H - NMR (D_2O / MeCN-d3, 400 MHz)

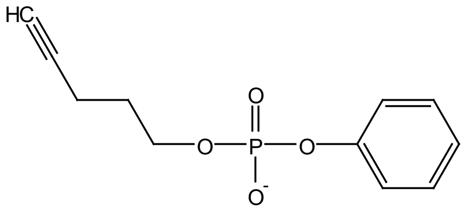

TBA - salt

55

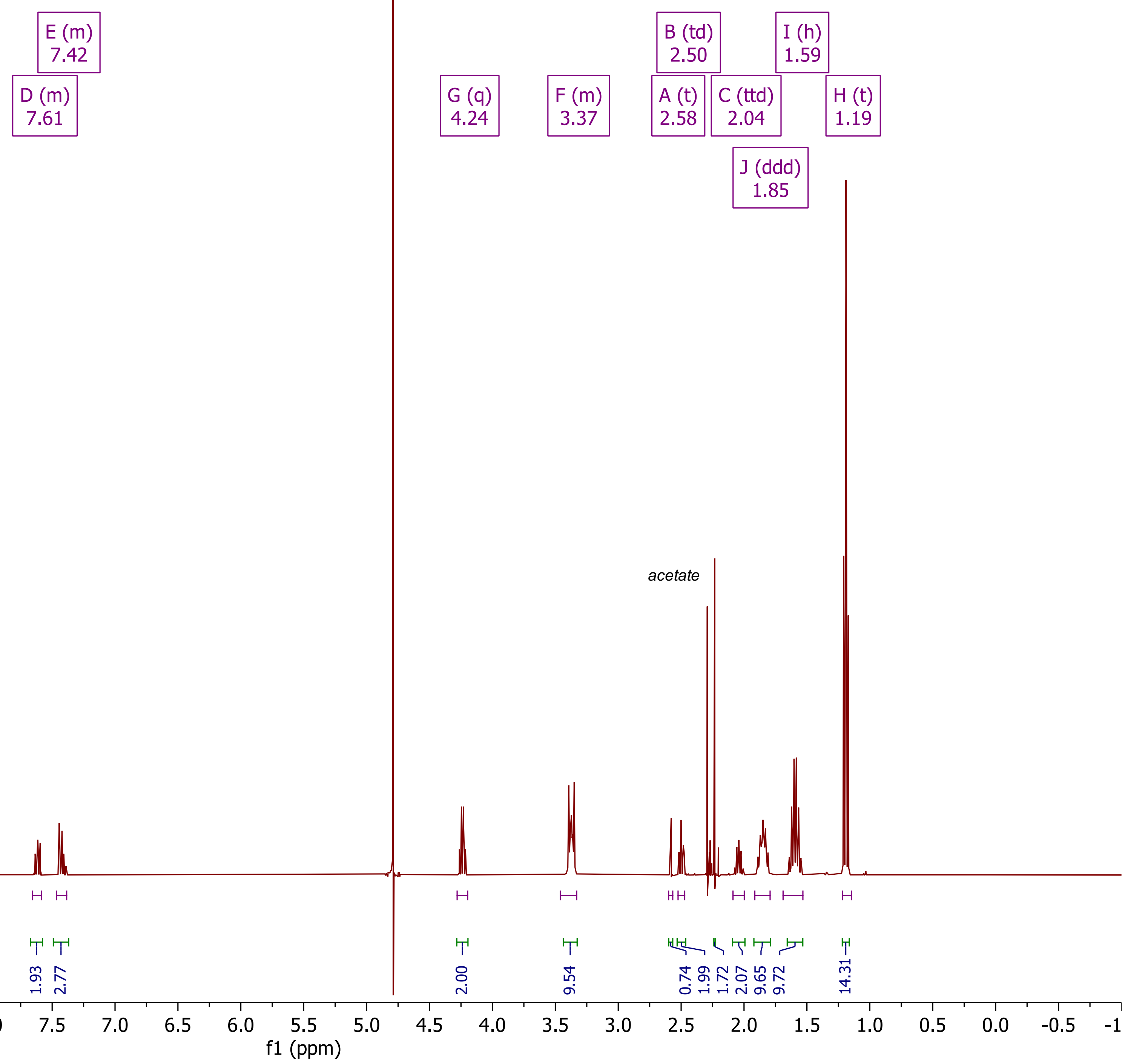

Compound 55, Pent-4-yn-1-yl-phenylphosphate, $^{31}P\ \{^{1}H\}$ - NMR (D2O/MeCN-d3, 162 MHz)

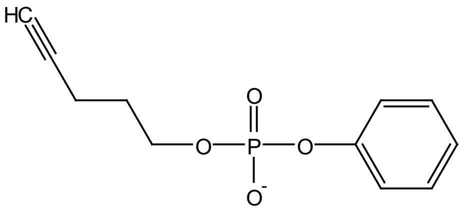

TBA - salt

55

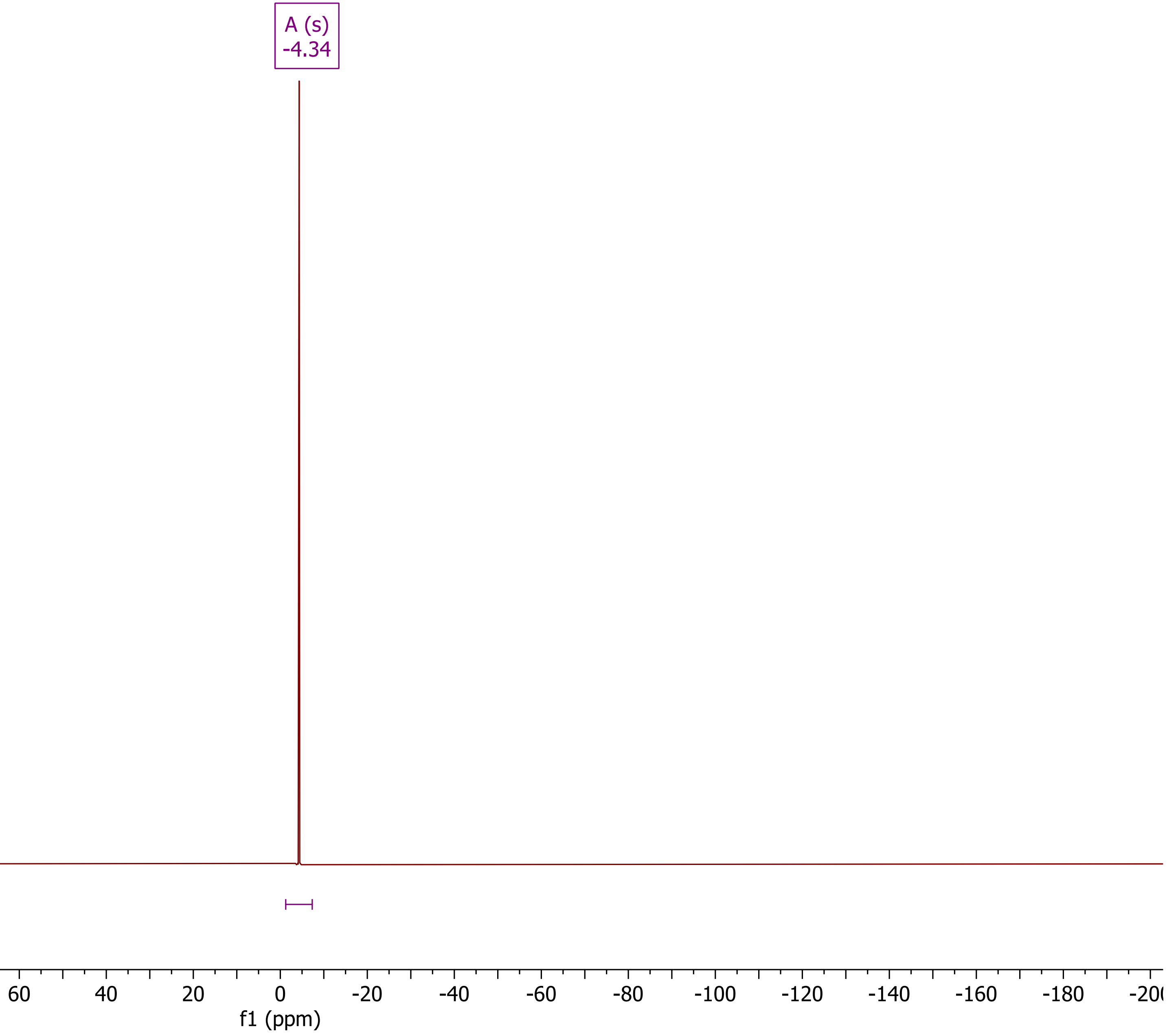

Compound 55, Pent-4-yn-1-yl-phenylphosphate, $^{13}C\ \{^1H\}$ - NMR (D_2O / MeCN-d3, 101 MHz)

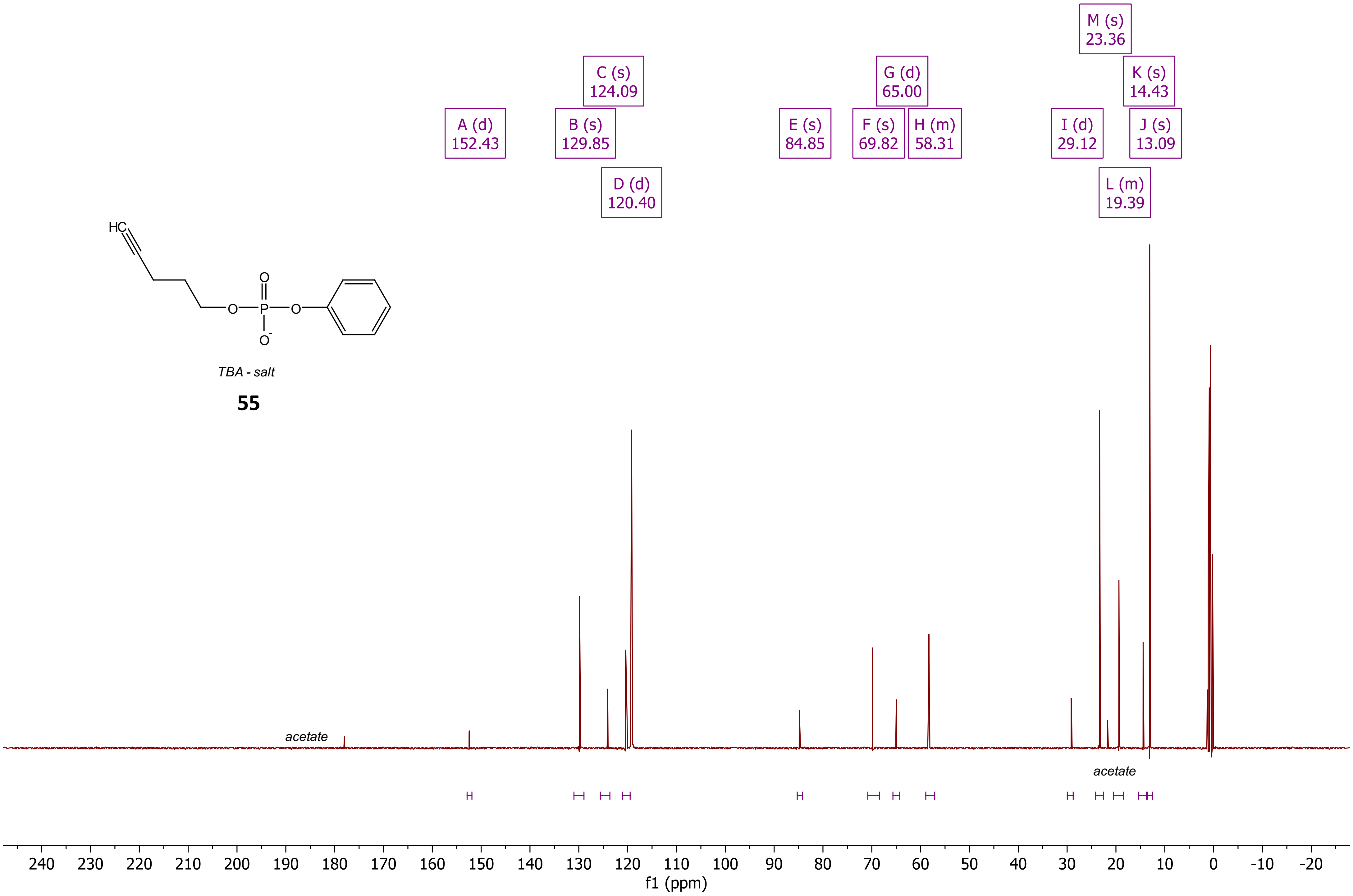

Compound 56, Isoprenyl-phenylphosphate, ¹H - NMR (MeCN-d3, 400 MHz)

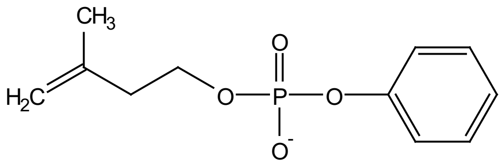

TEA- salt

56

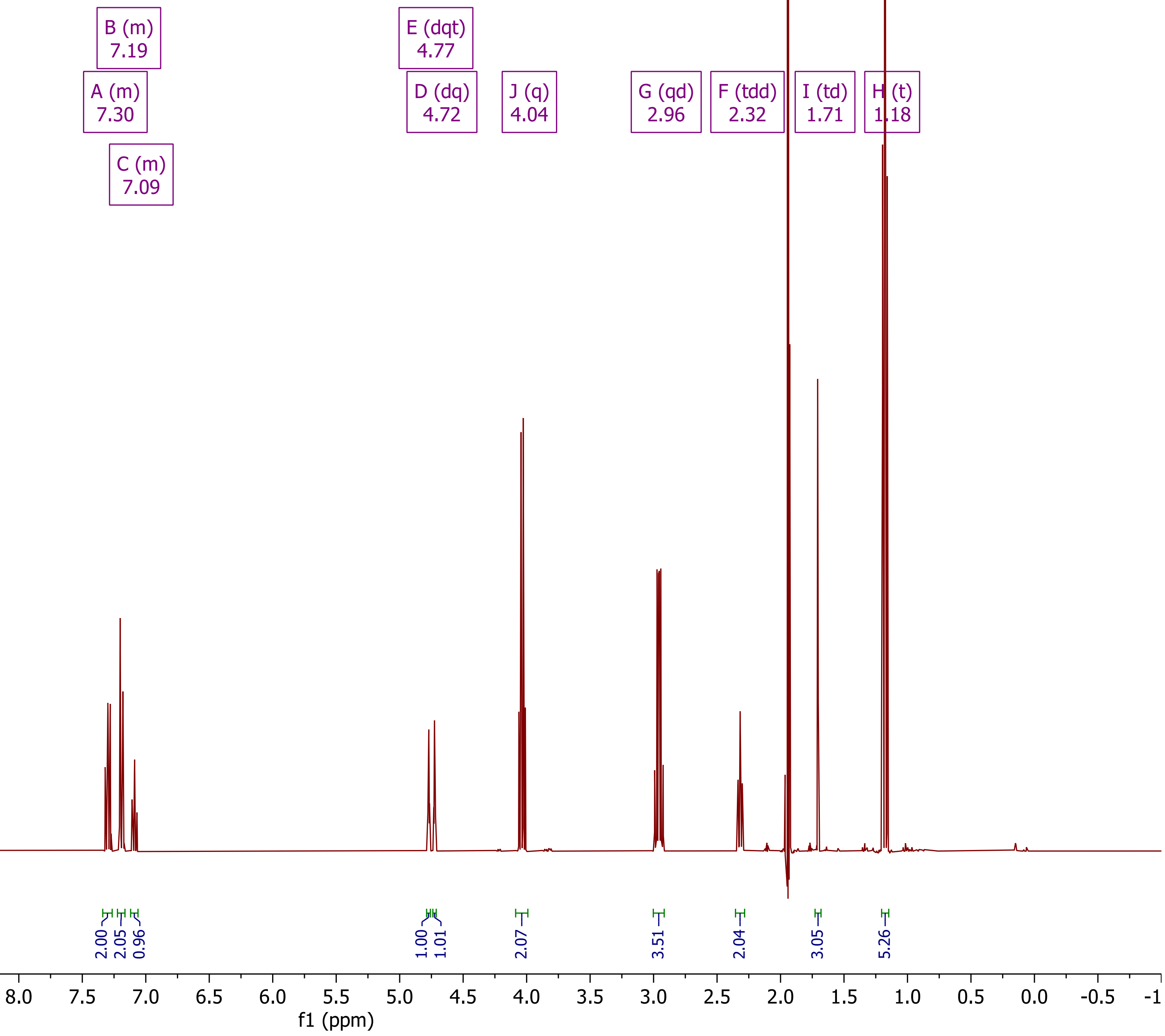

Compound 56, Isoprenyl-phenylphosphate, ^{31}P {^{1}H} - NMR (MeCN-d3, 162 MHz)

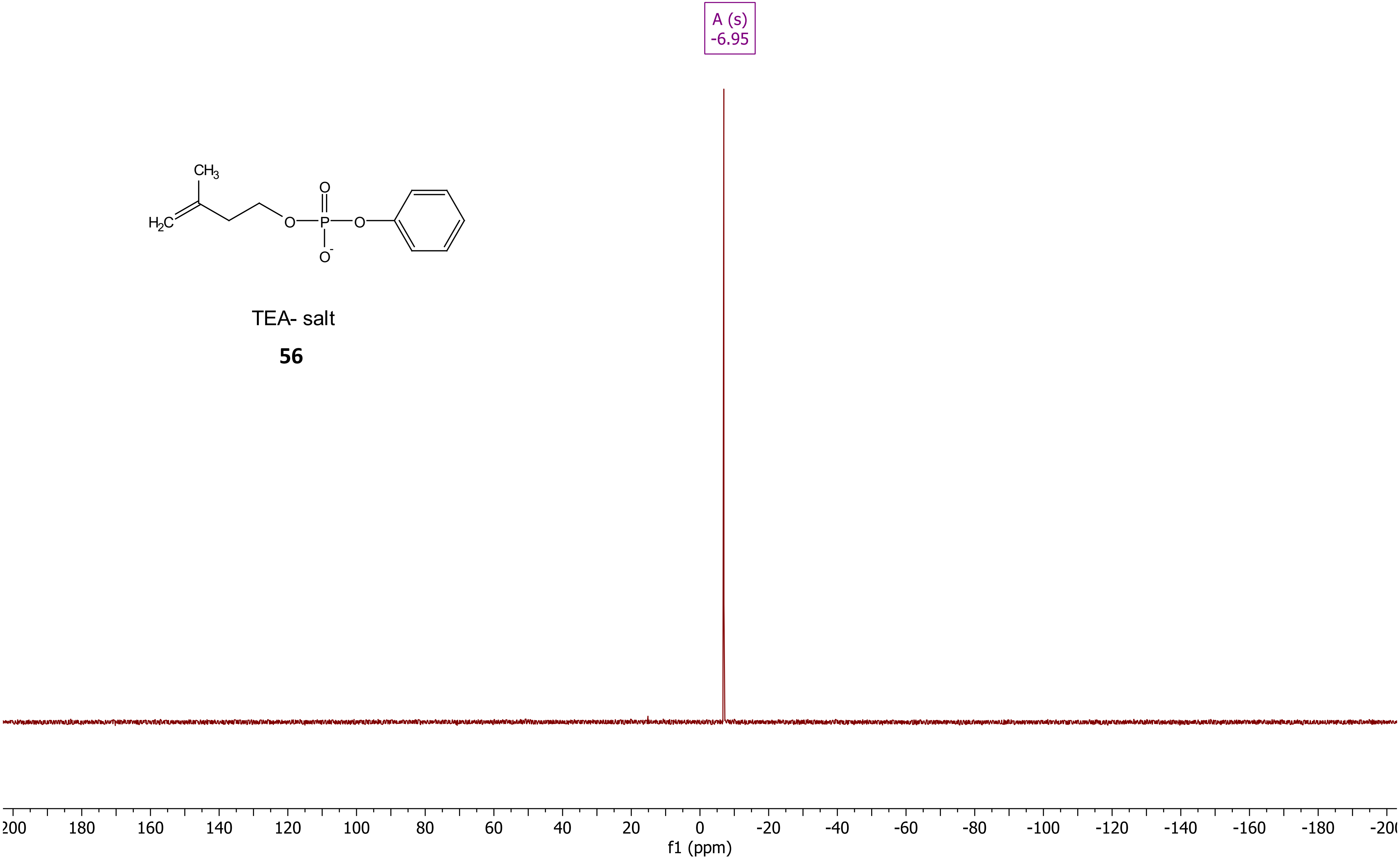

TEA- salt

56

Compound 56, Isoprenyl-phenylphosphate, ^{13}C {^{1}H} - NMR (MeCN-d3, 101 MHz)

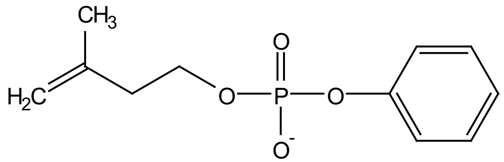

TEA- salt

56

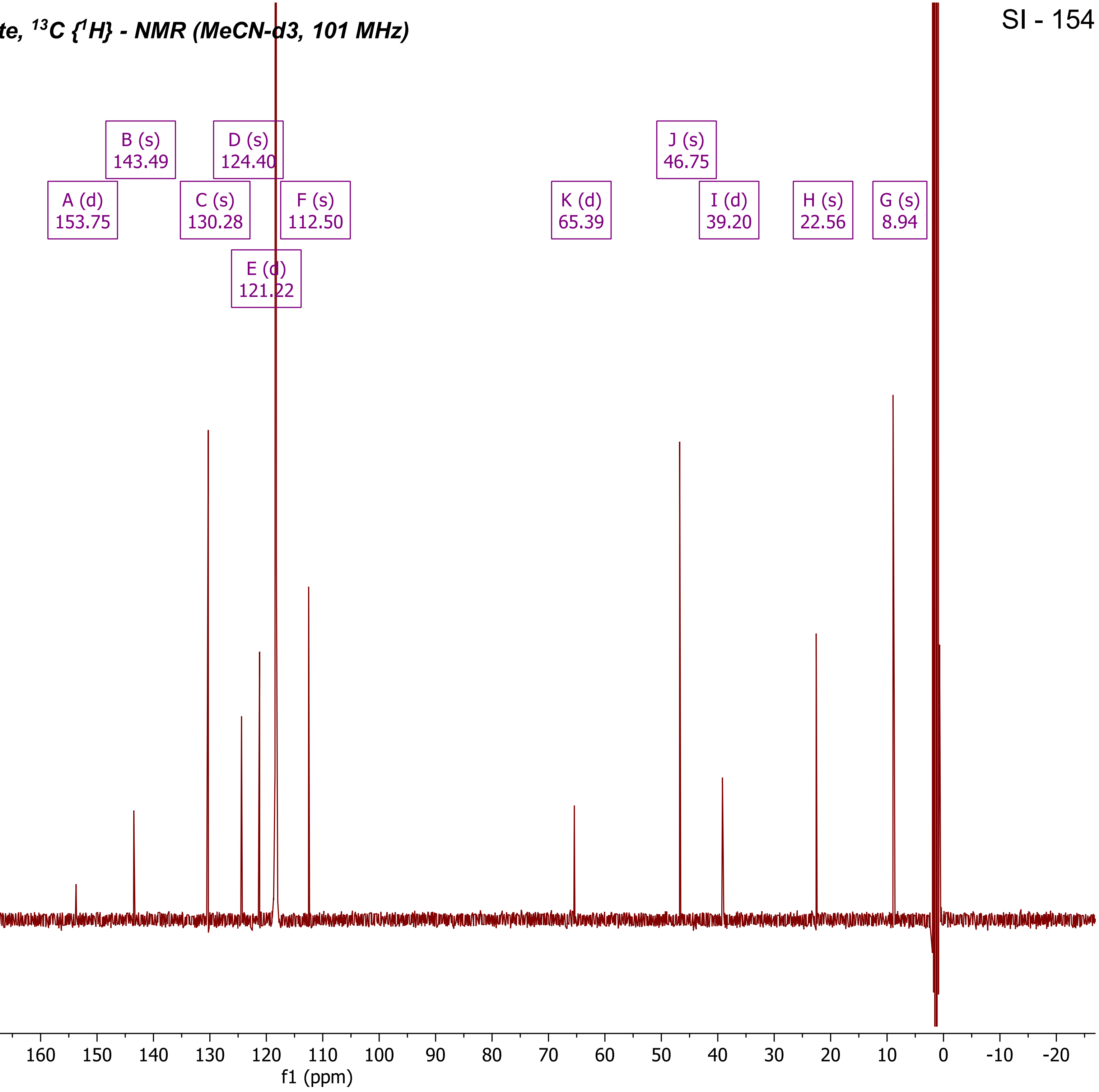

Compound 57, Geranyl-phenylphosphate, ^{1}H - NMR (MeCN-d3, 400 MHz)

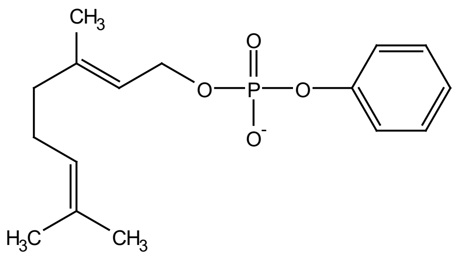

TEA - salt

57

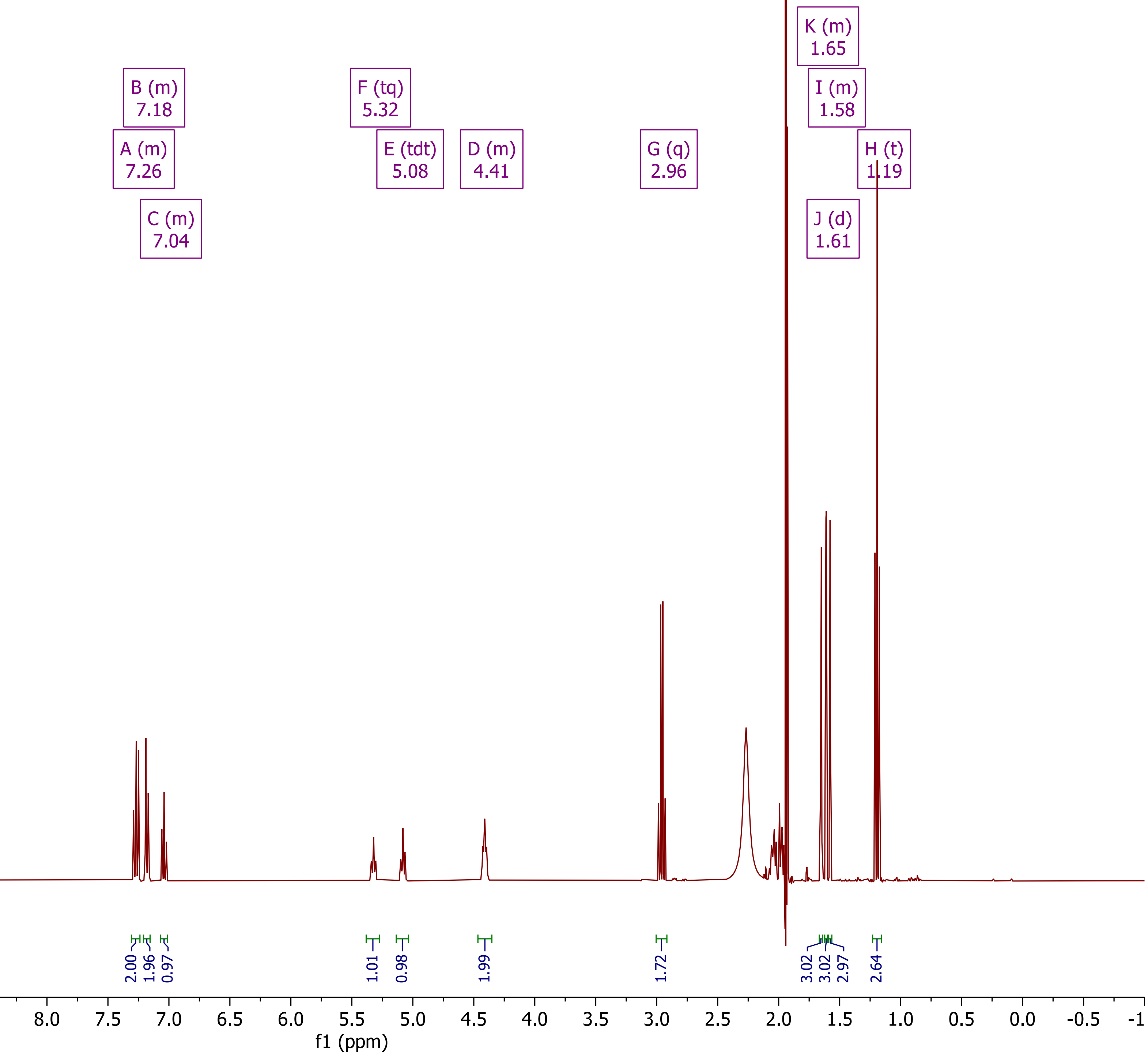

Compound 57, Geranyl-phenylphosphate, ^{31}P {^{1}H} - NMR (MeCN-d3, 162 MHz)

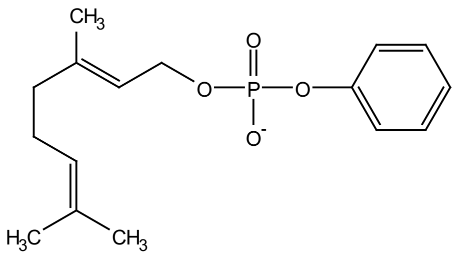

TEA - salt

57

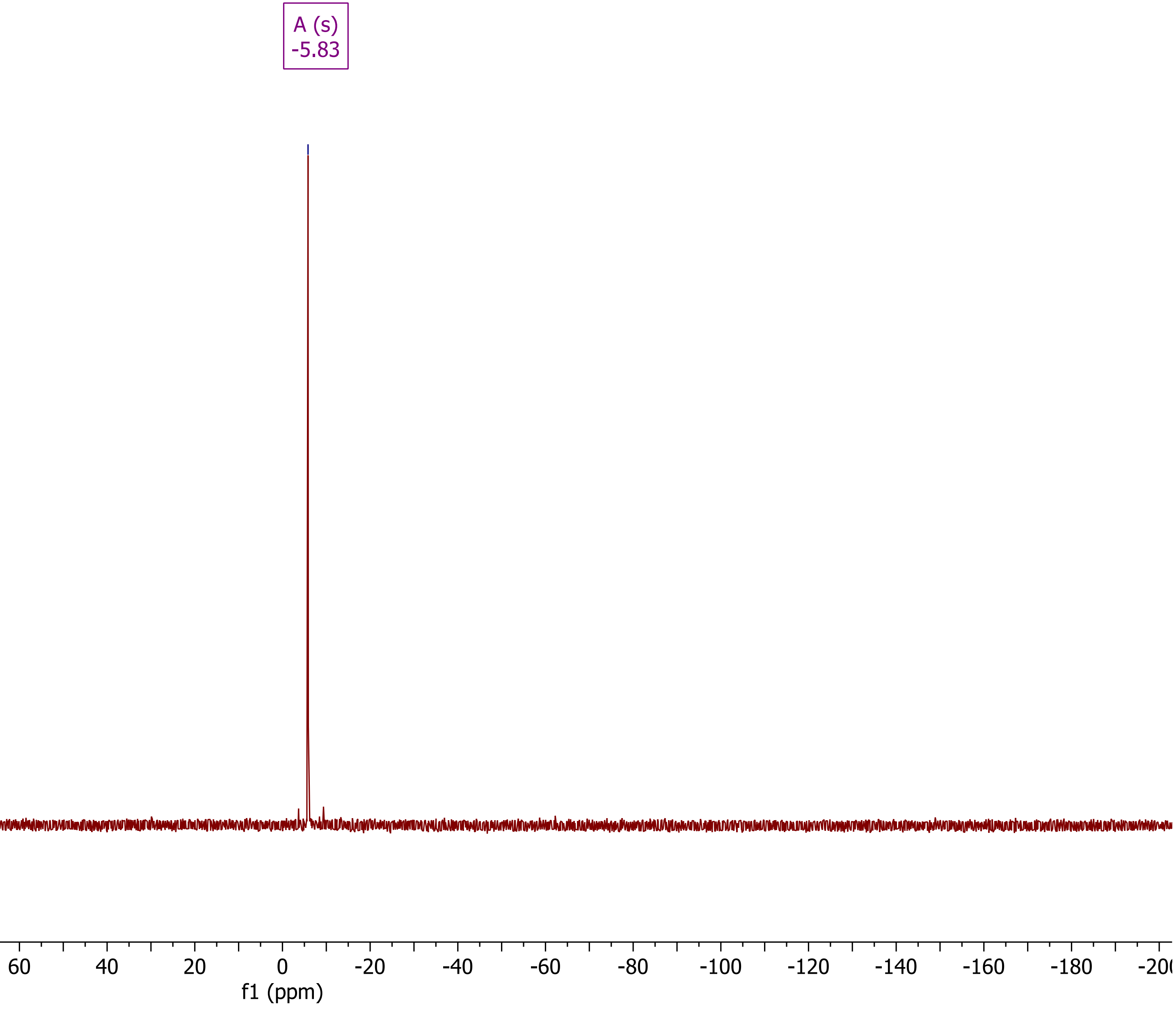

Compound 57, Geranyl-phenylphosphate, ^{31}P - NMR (MeCN-d3, 162 MHz)

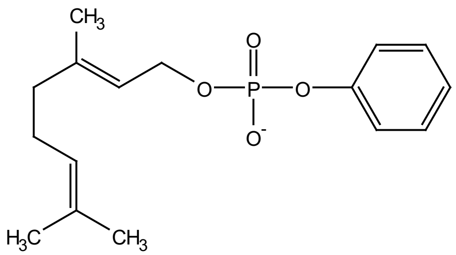

TEA - salt

57

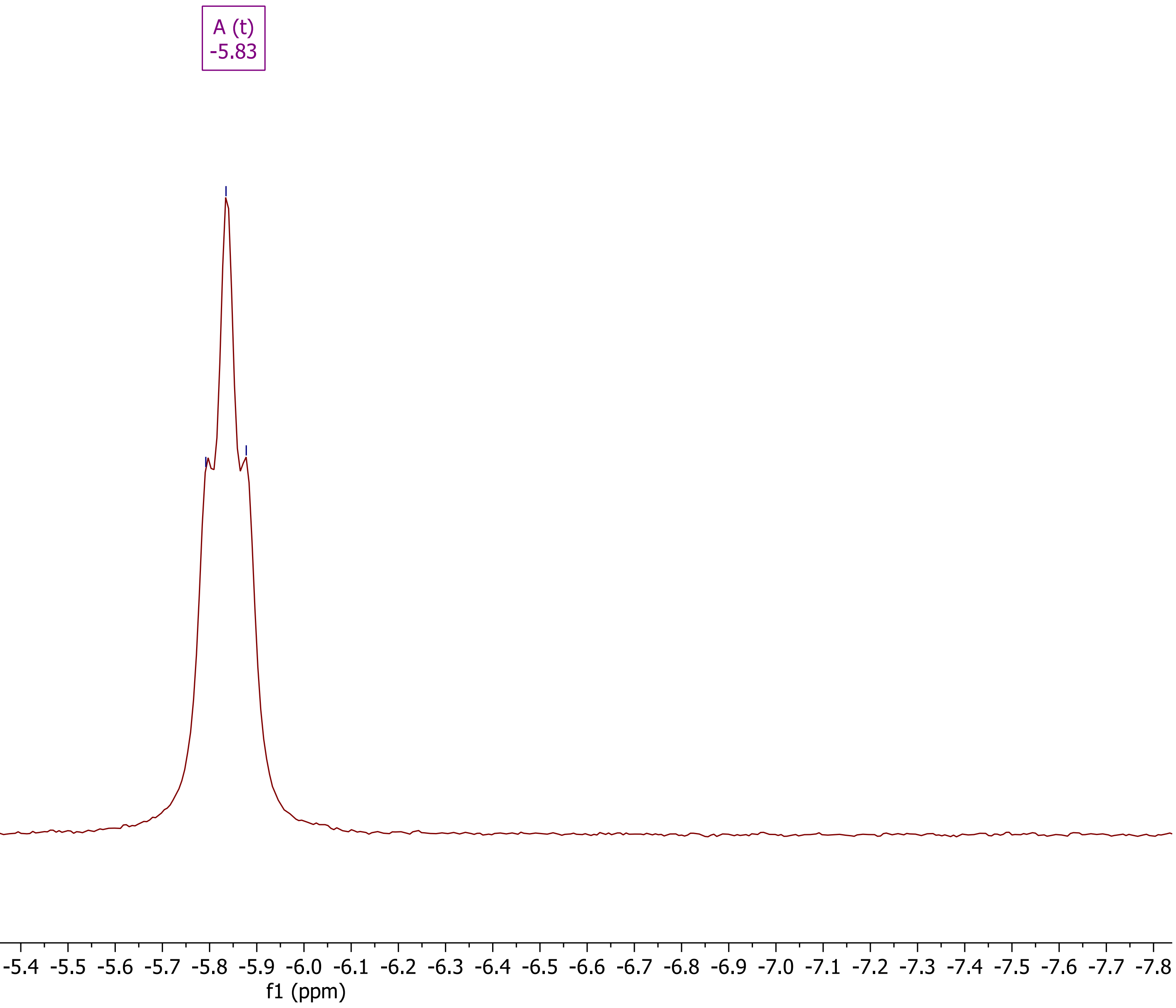

Compound 57, Geranyl-phenylphosphate, $^{13}C\ \{^1H\}$ - NMR (MeCN-d3, 101 MHz)

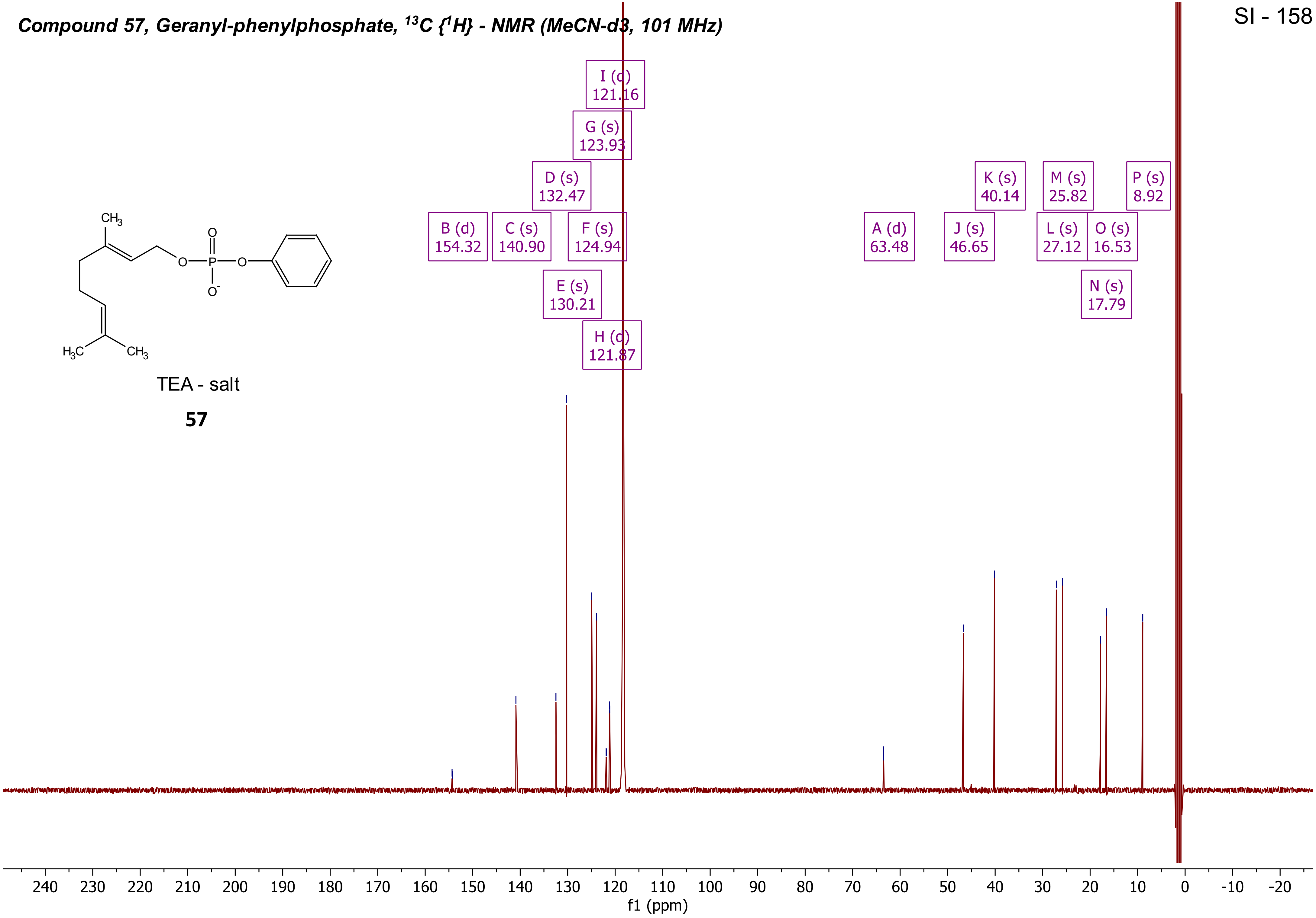

Compound 58, 6-Hydroxyhexyl-phenylphosphate, ^{1}H - NMR (D_2O / MeCN-d3, 400 MHz)

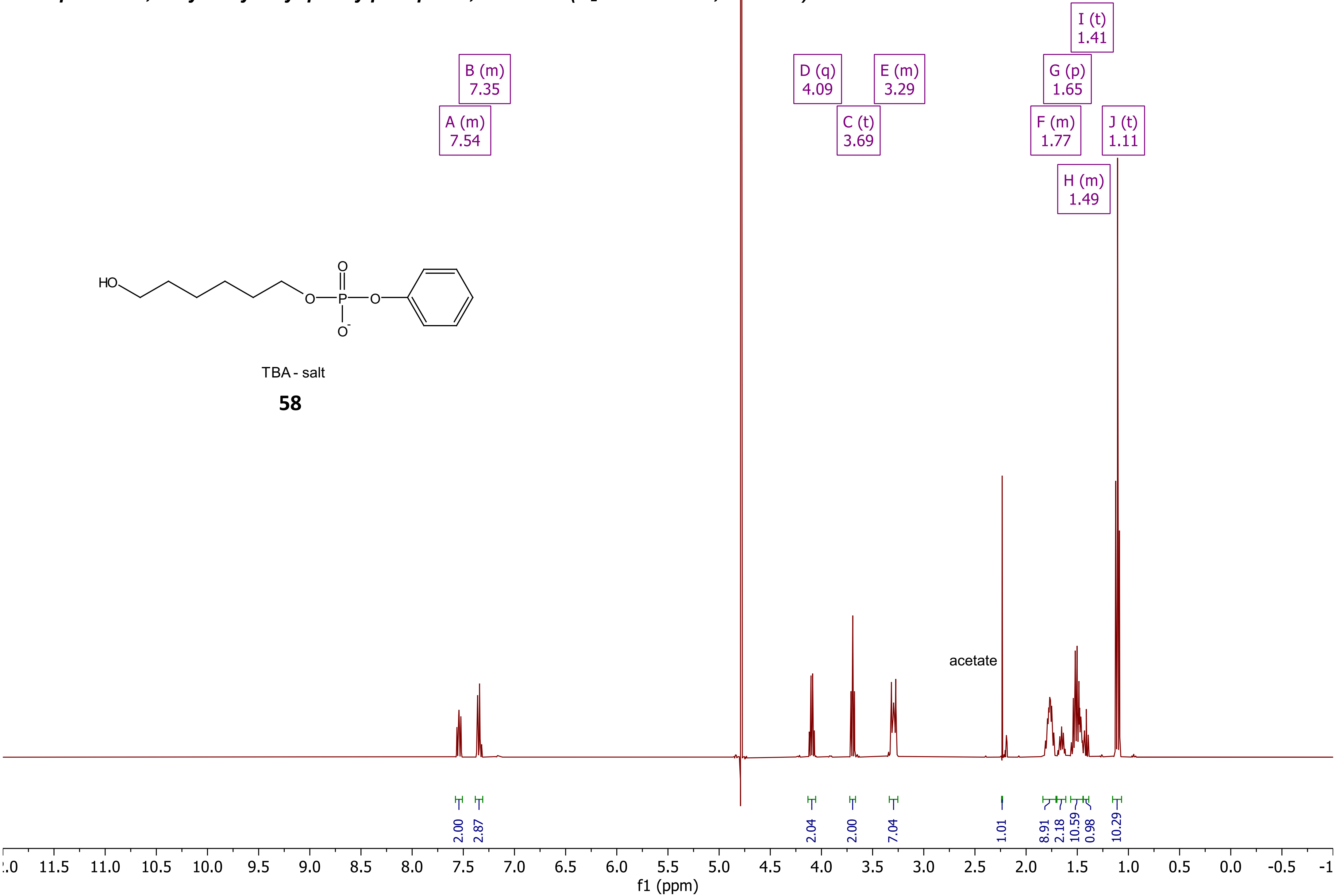

Compound 58, 6-Hydroxyhexyl-phenylphosphate, ^{31}P {^{1}H} - NMR (D_2O/MeCN-d3, 162 MHz)

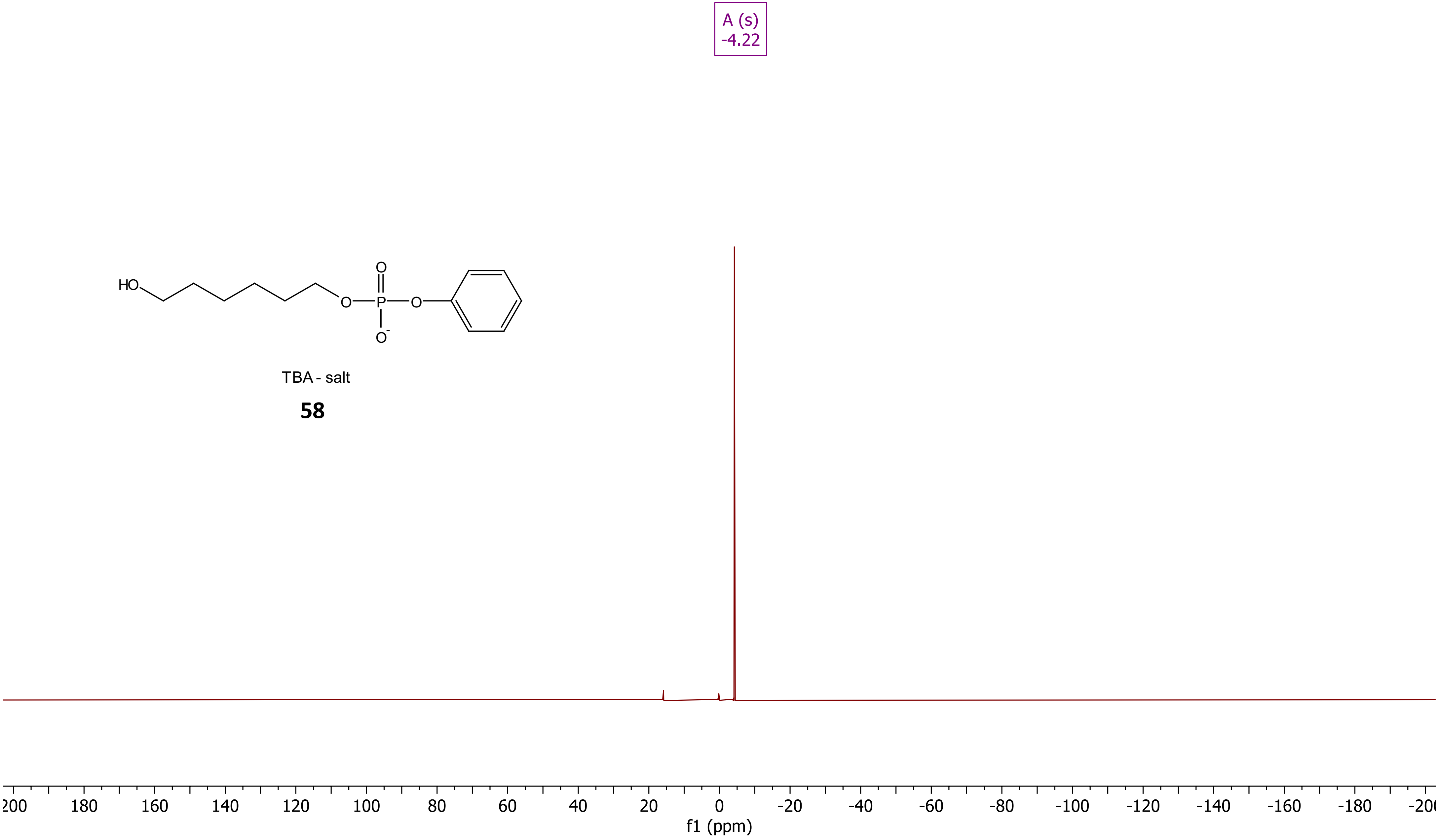

TBA - salt

58

Compound 58, 6-Hydroxyhexyl-phenylphosphate, ^{1}H - ^{31}P - HMBC (D_2O/MeCN-d3)

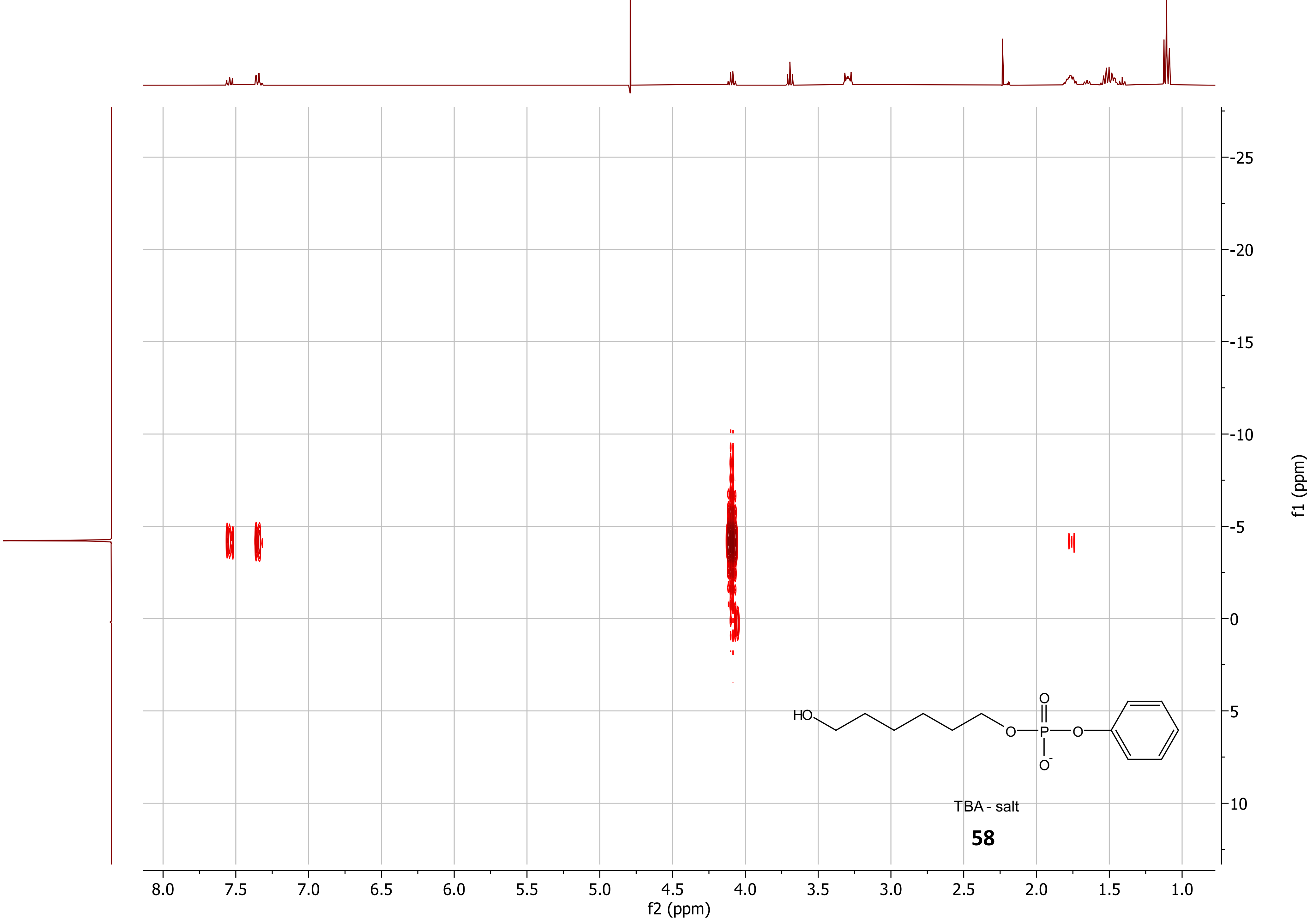

Compound 58, 6-Hydroxyhexyl-phenylphosphate, $^{13}C\{^1H\}$ - NMR (D_2O/MeCN-d3, 101 MHz)

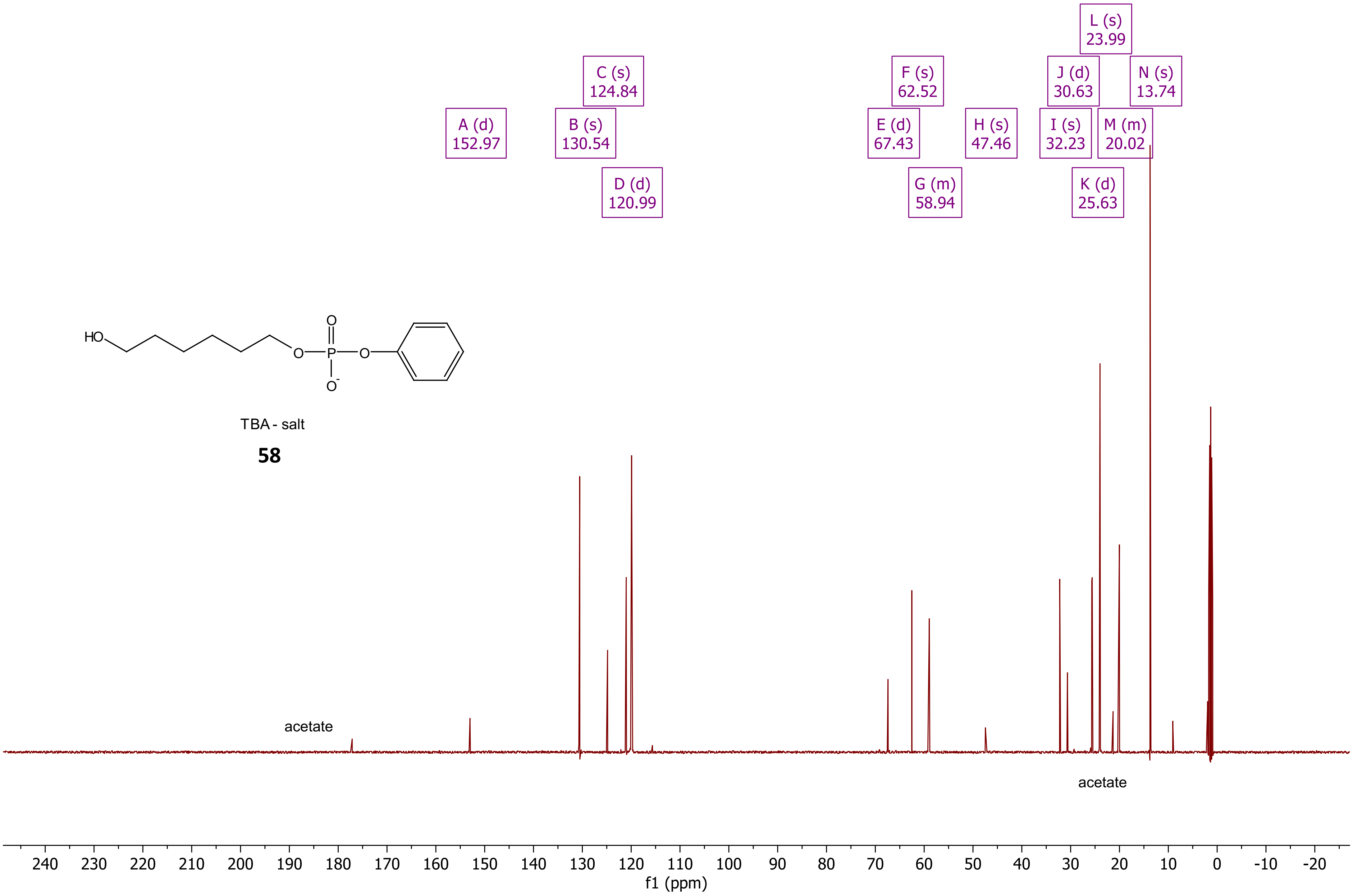

Compound 59, D4T-phenylphosphate, ^{1}H - NMR (D_2O, 400 MHz)

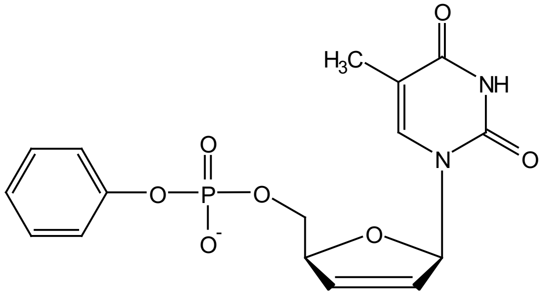

TEA - salt

59

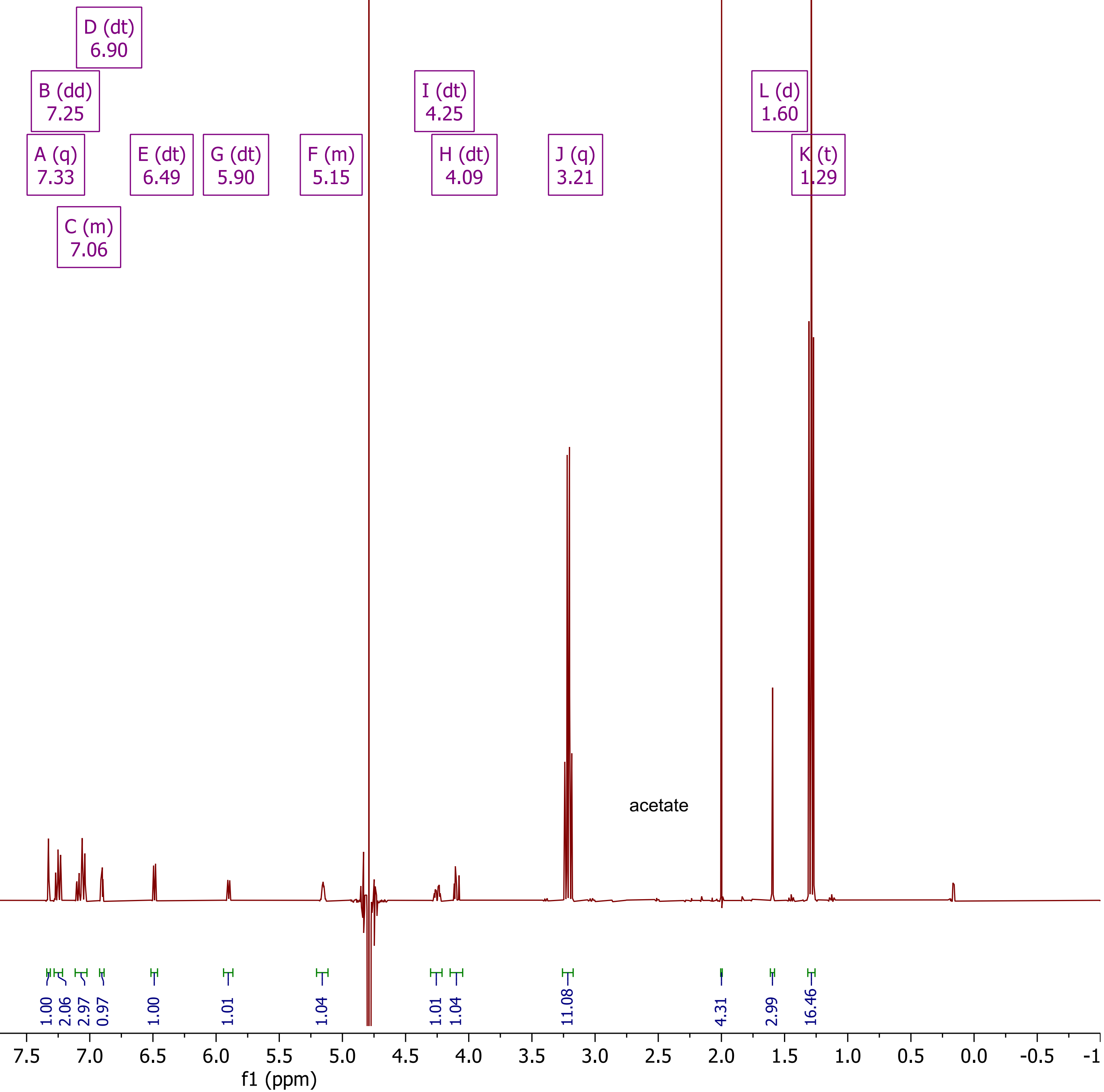

Compound 59, D4T-phenylphosphate, $^{31}P\ \{^{1}H\}$ - NMR (D_2O, 162 MHz)

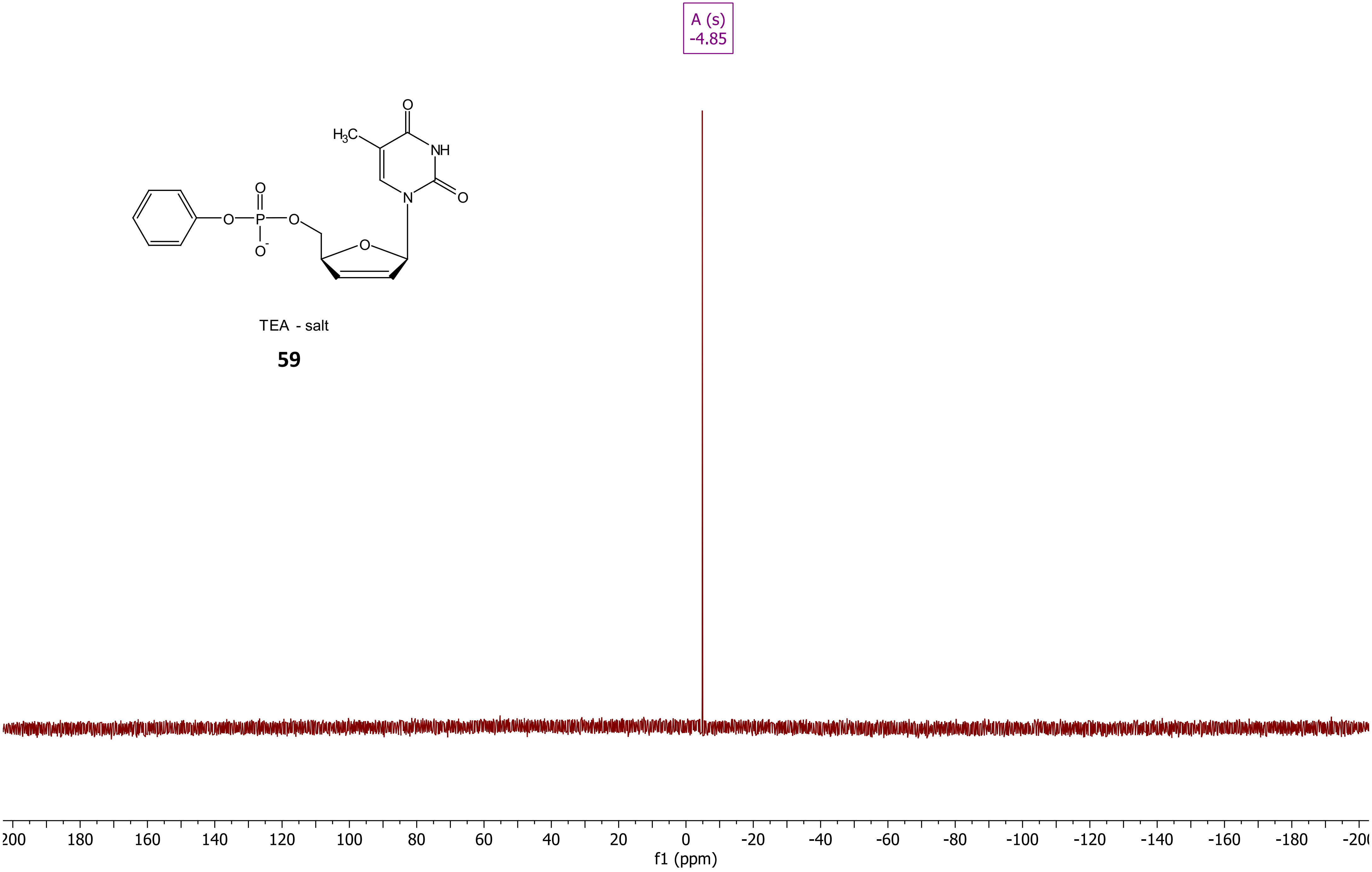

TEA - salt

59

Compound 59, D4T-phenylphosphate, $^{13}C\ \{^1H\}$ - NMR (D_2O, 101 MHz)

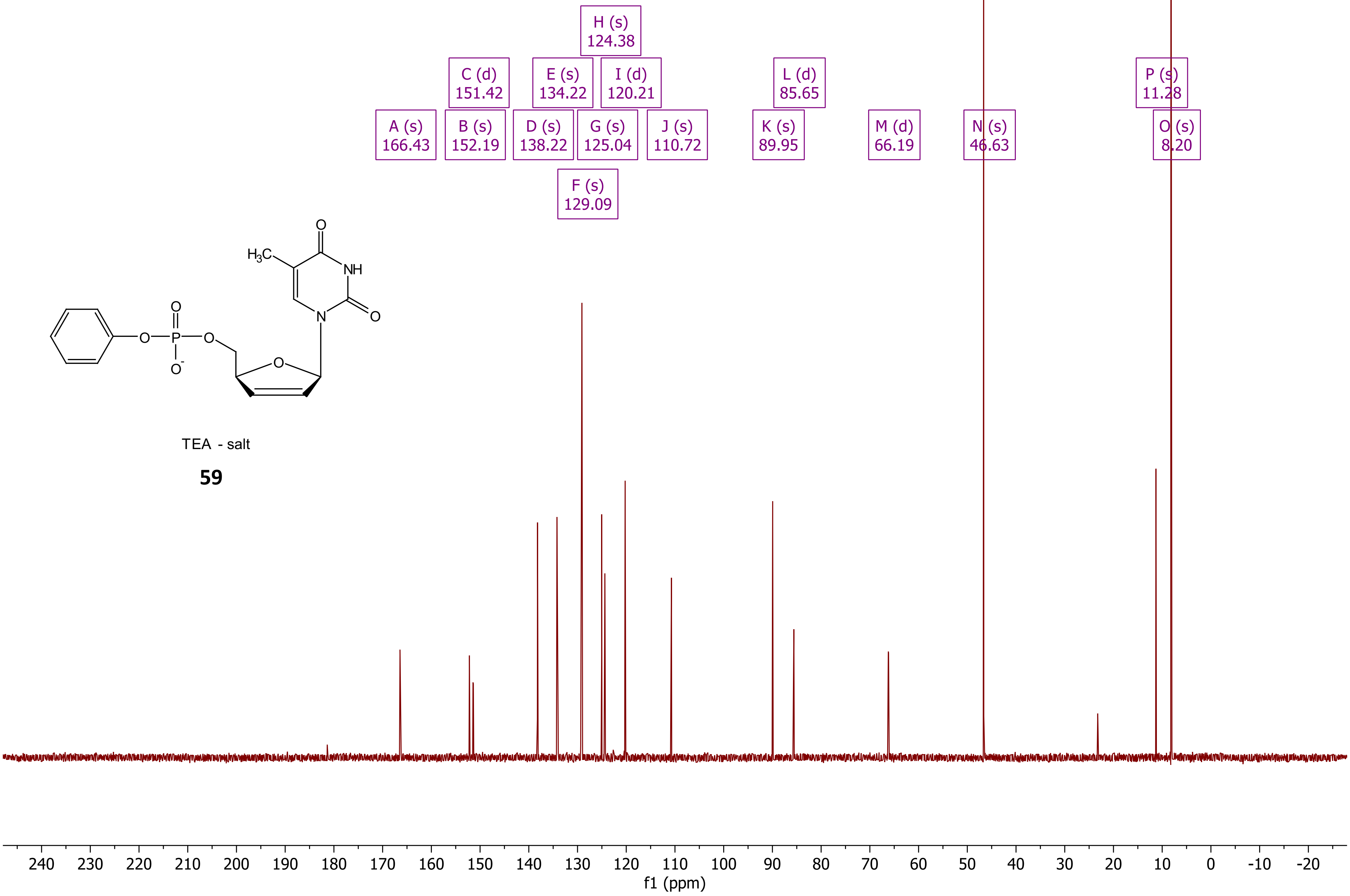

59

Compound 60, D4T-phenylpyrophosphate, ^{1}H - NMR (D_2O, 400 MHz)

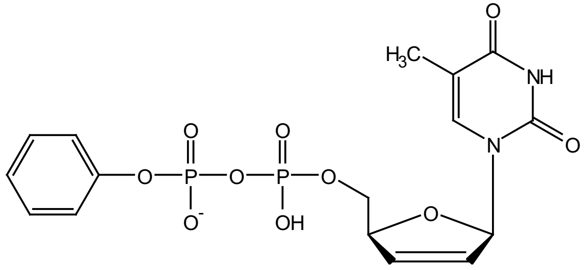

TEA - salt

60

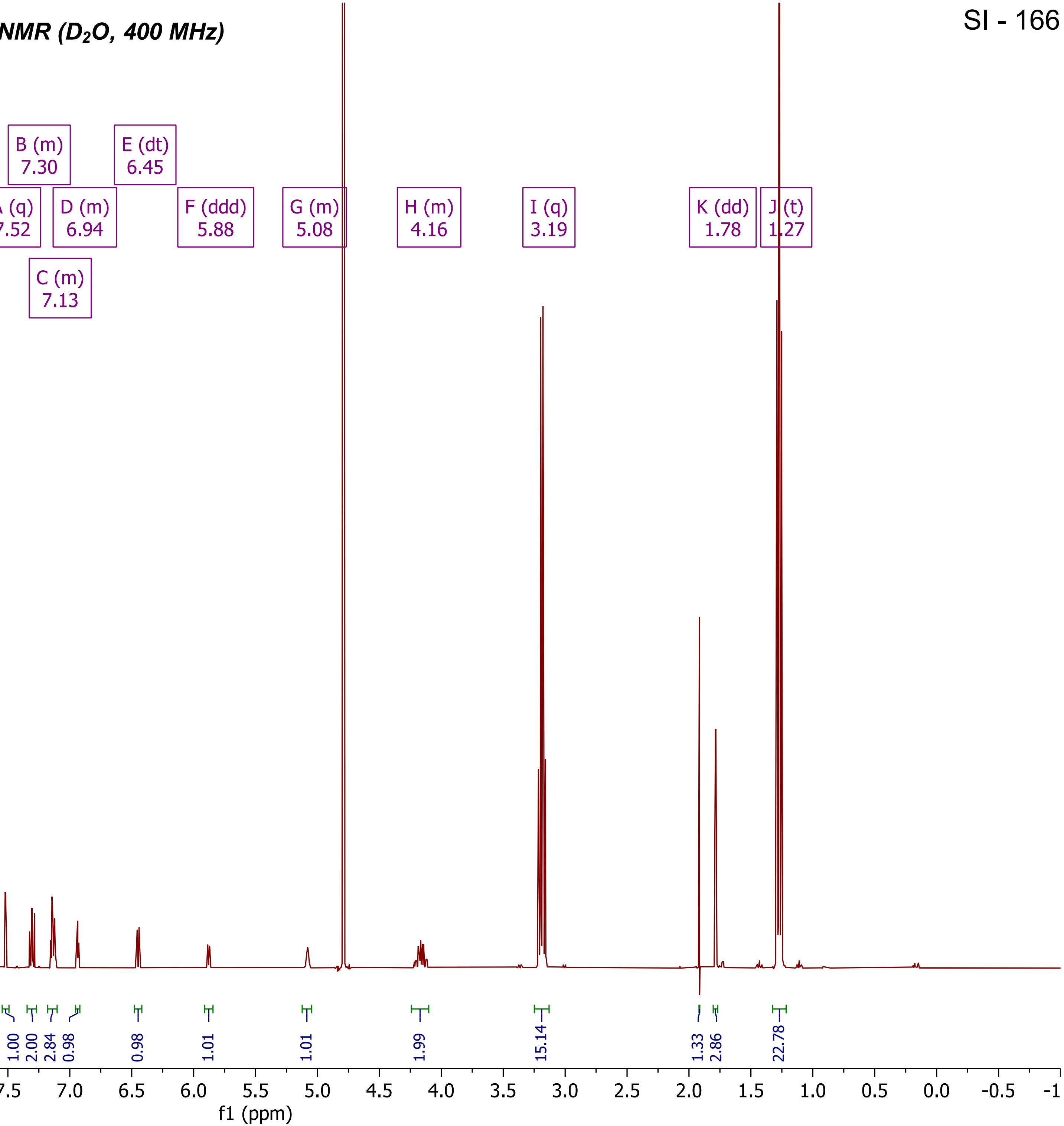

Compound 60, D4T-phenylpyrophosphate, $^{31}P\ \{^{1}H\}$ - NMR (D_2O, 162 MHz)

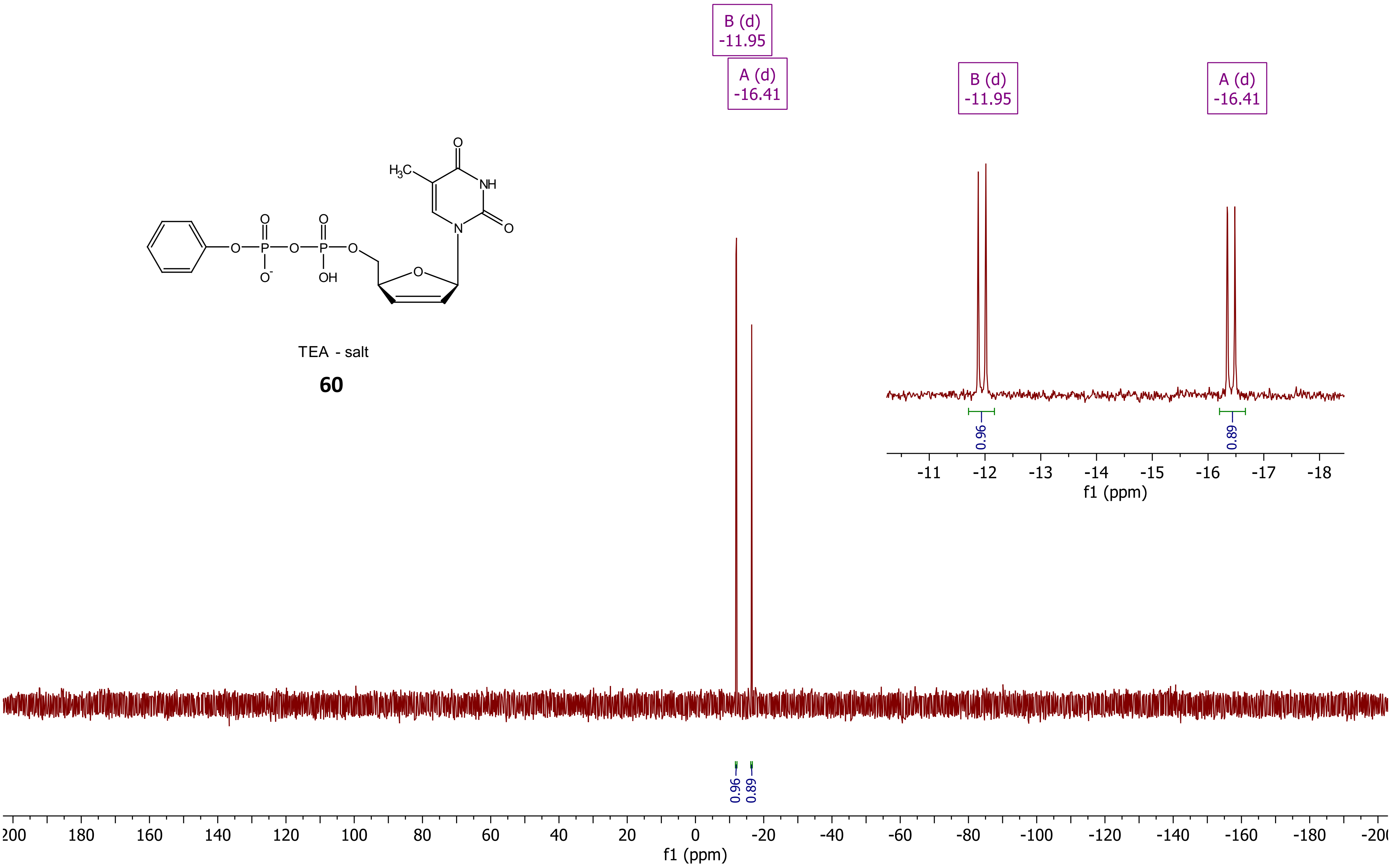

TEA - salt

60

Compound 60, D4T-phenylpyrophosphate, $^{13}C\{^{1}H\}$ - NMR (D_2O, 101 MHz)

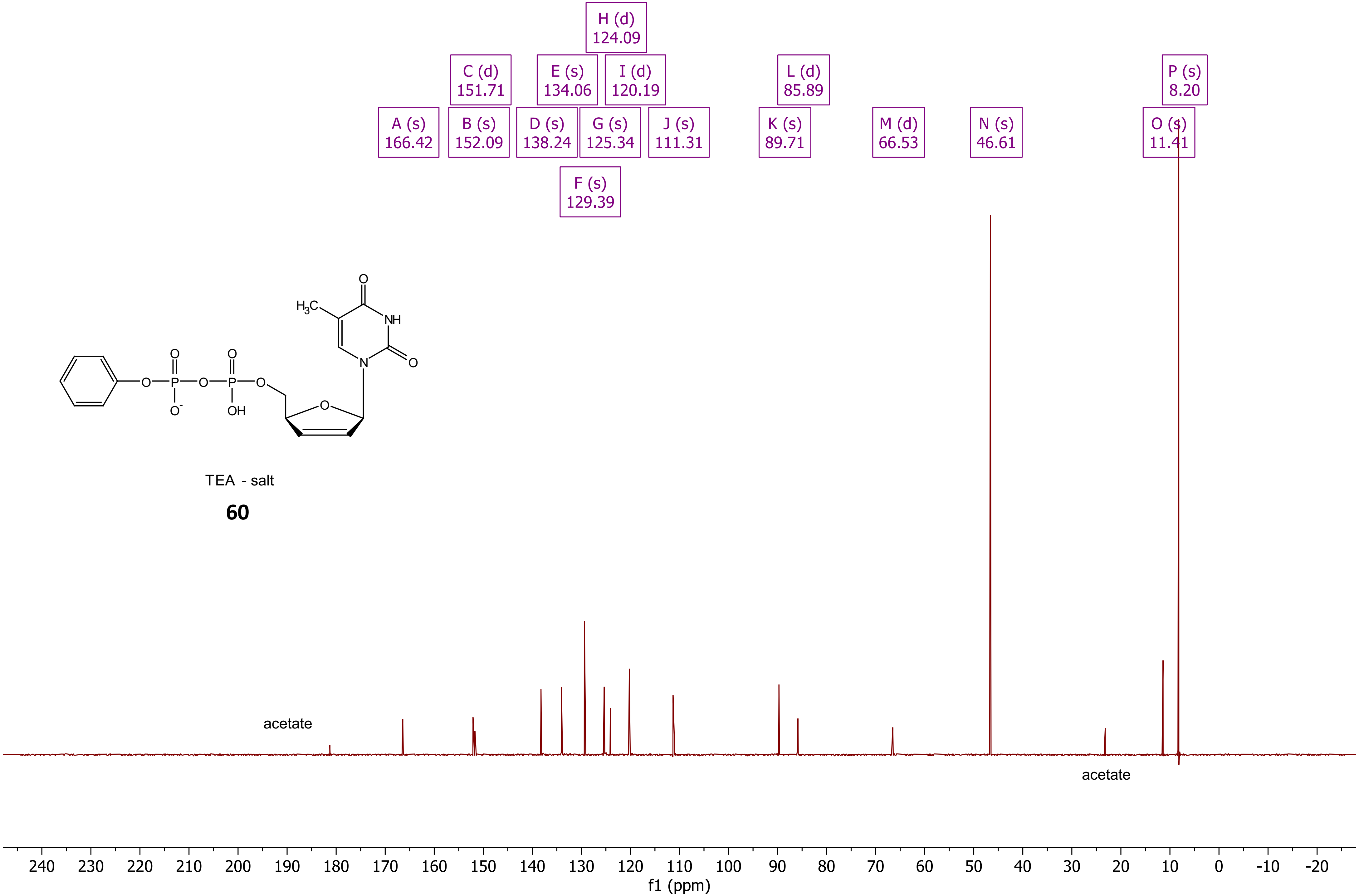

Compound 61, Diphenylpyrophosphate, ¹H - NMR (D_2O, 400 MHz)

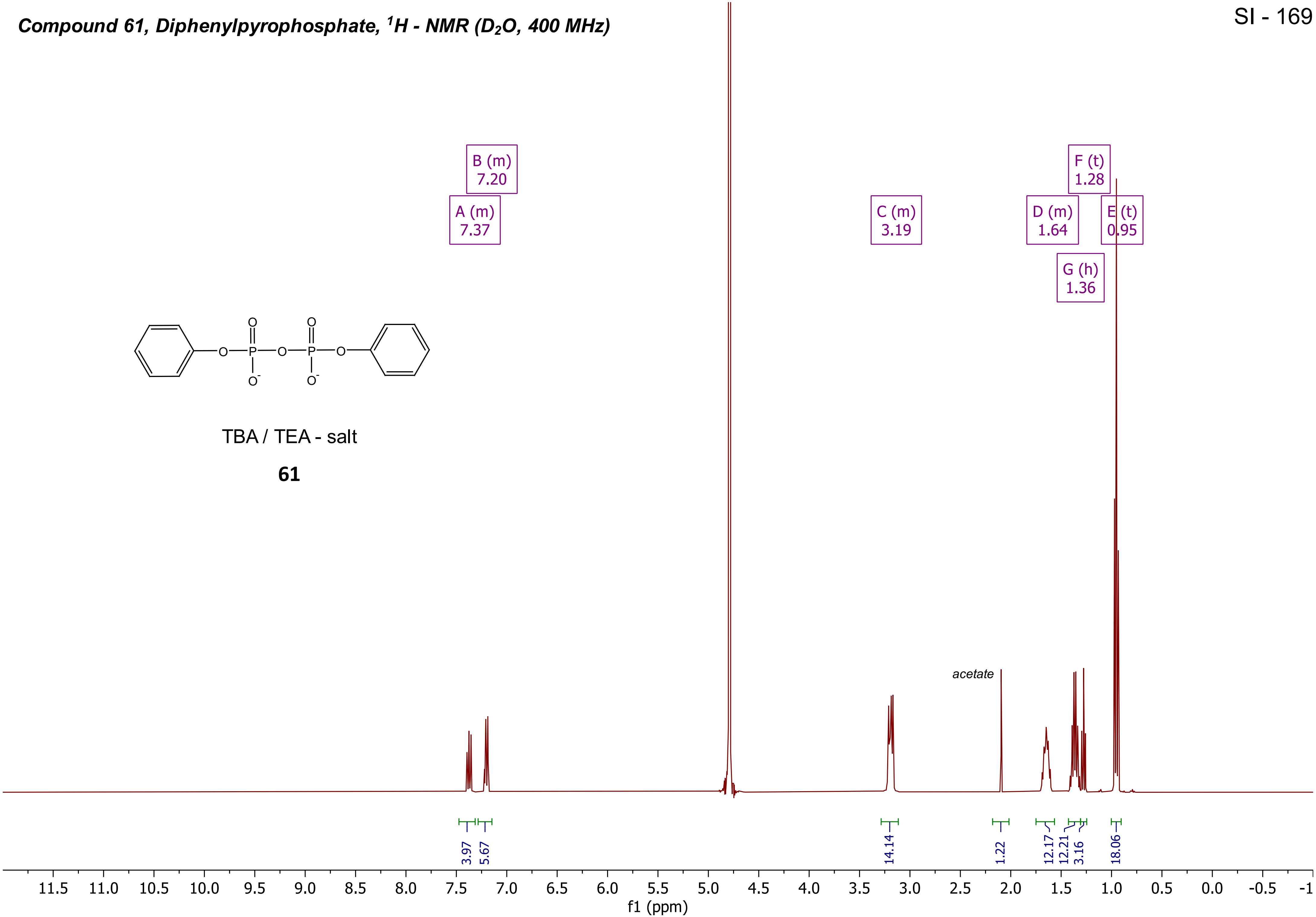

Compound 61, Diphenylpyrophosphate, ^{31}P {^{1}H} - NMR (D2O, 162 MHz)

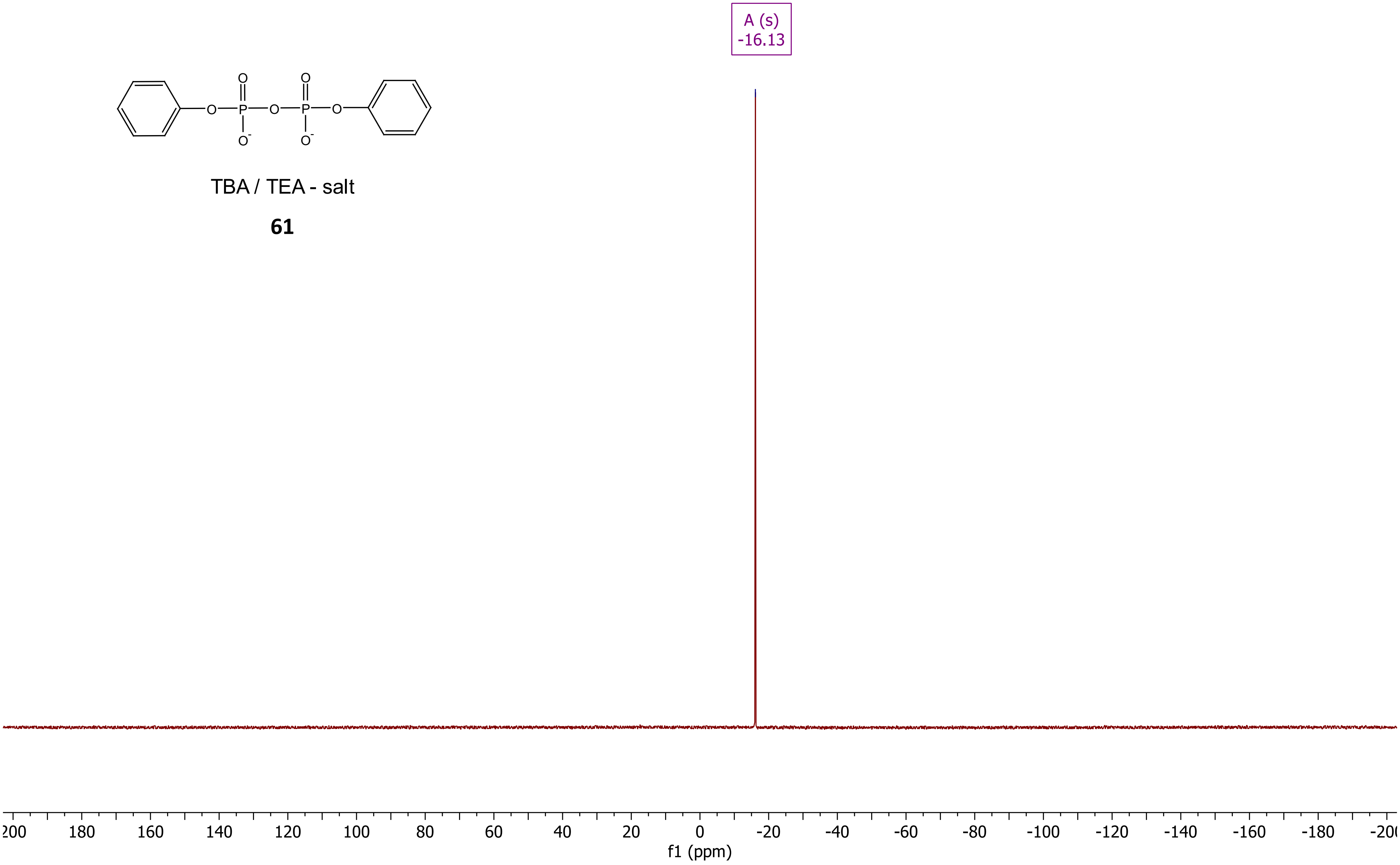

TBA / TEA - salt

61

Compound 61, Diphenylpyrophosphate, ^{13}C - NMR (D2O, 101 MHz)

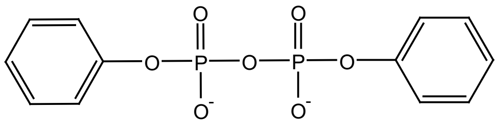

TBA / TEA - salt

61

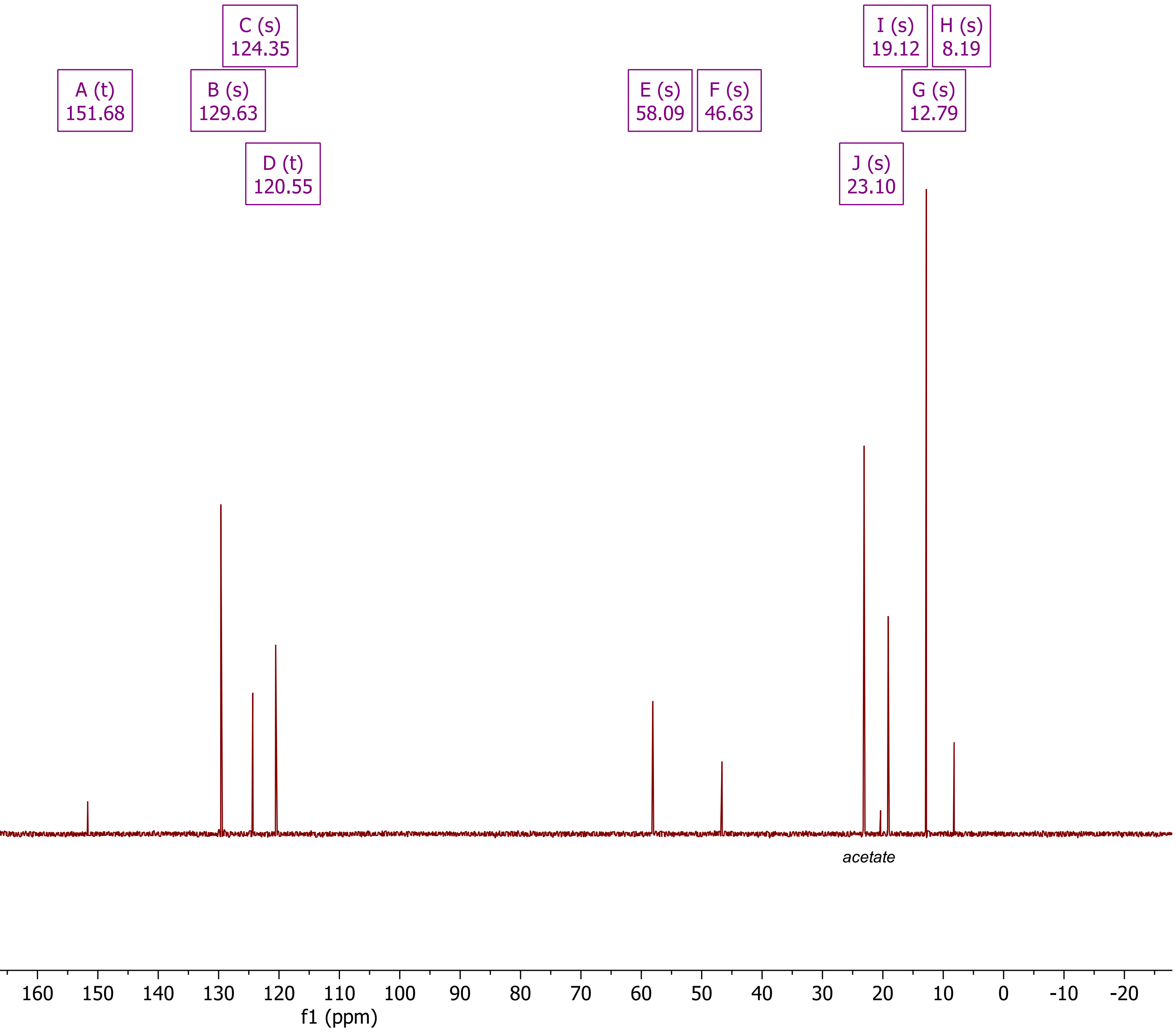

Compound 67, Pentylphosphate, ^{1}H - NMR (D_2O, 400 MHz)

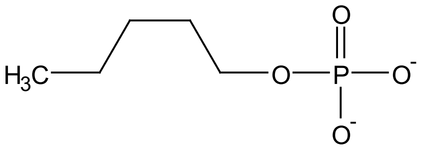

TBA/TEA - salt

67

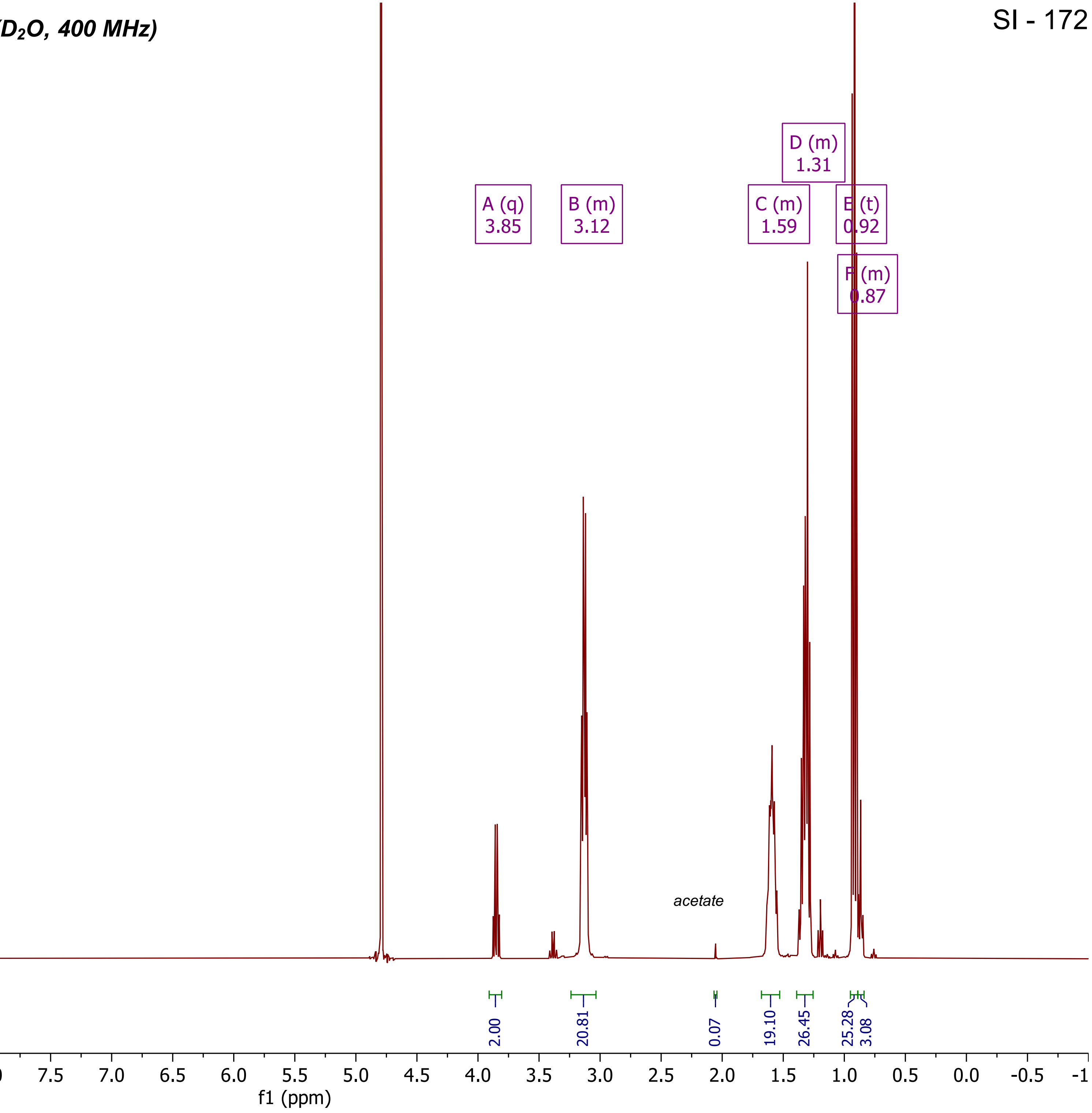

Compound 67, Pentylphosphate, $^{31}P\{^{1}H\}$ - NMR (D2O, 162 MHz)

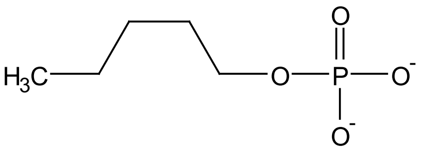

TBA/TEA - salt

67

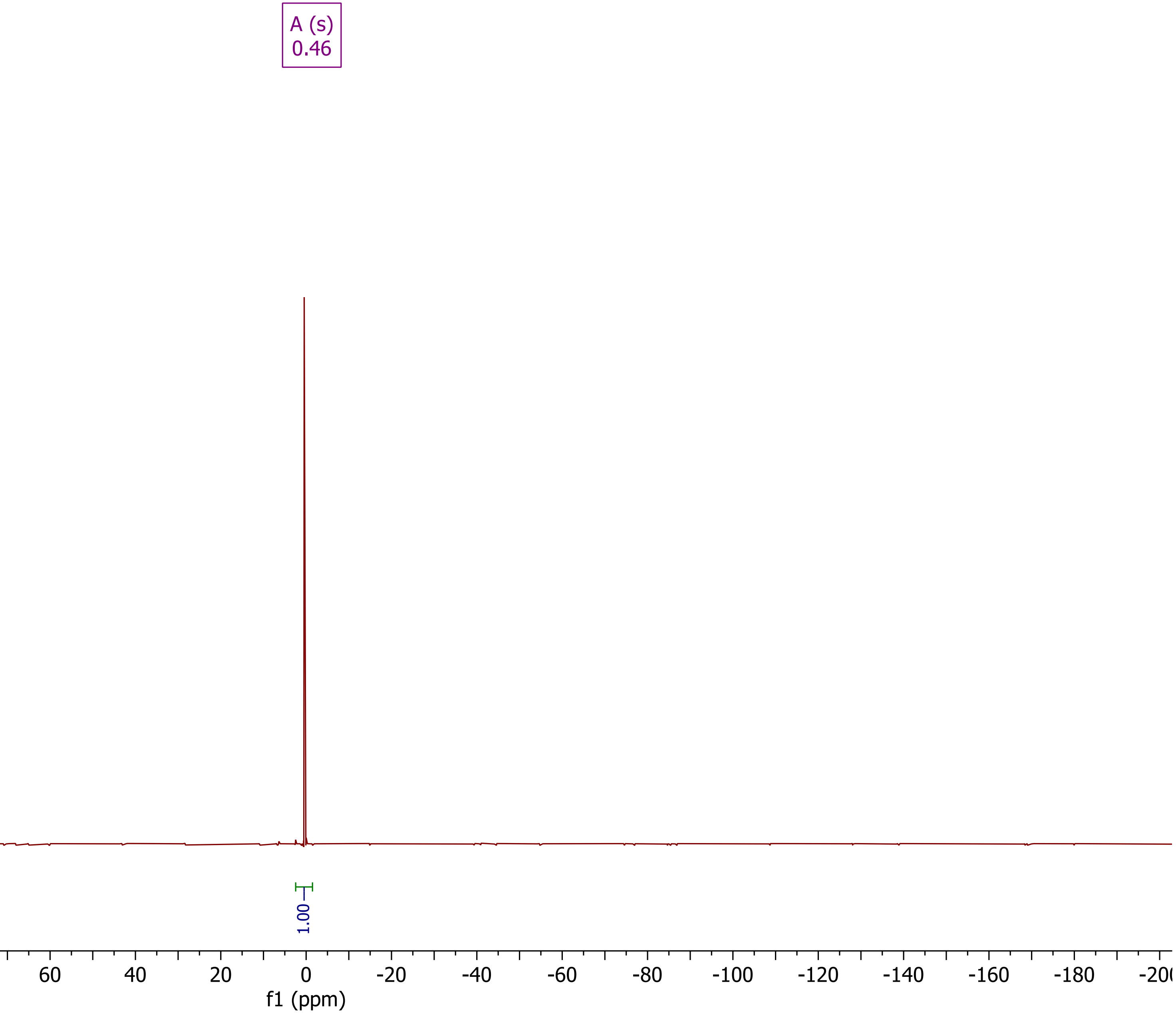

Compound 67, Pentylphosphate, $^{13}C\{^{1}H\}$ - NMR (D2O, 101 MHz)

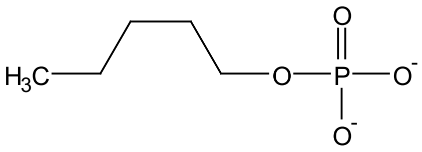

TBA/TEA - salt

67

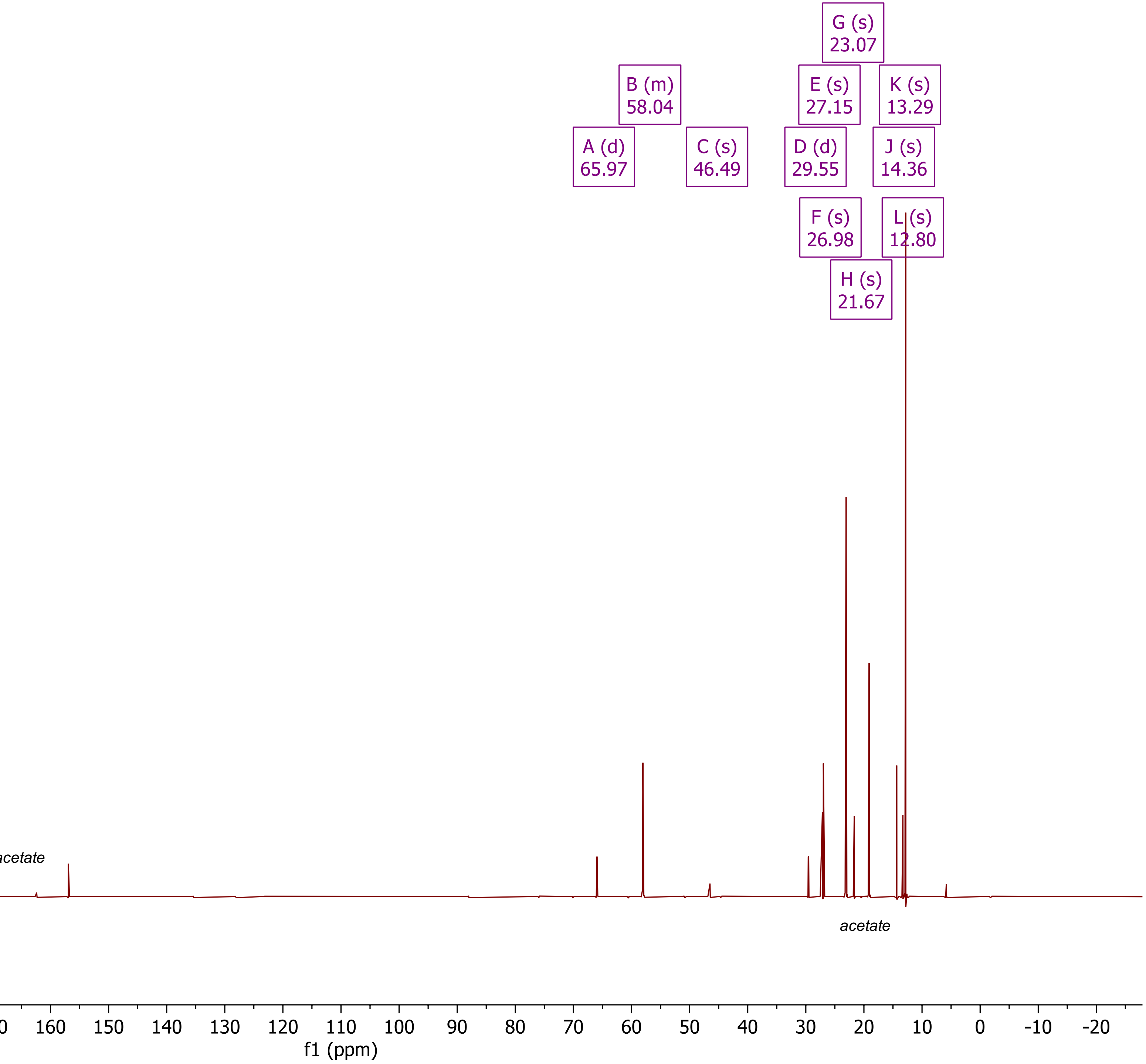

Compound 73, Phenylcyclotriphosphate, ^{31}P {^{1}H} - NMR (MeCN-d3, 162 MHz)

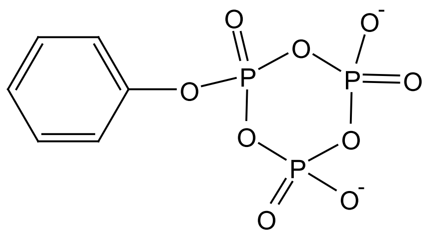

73

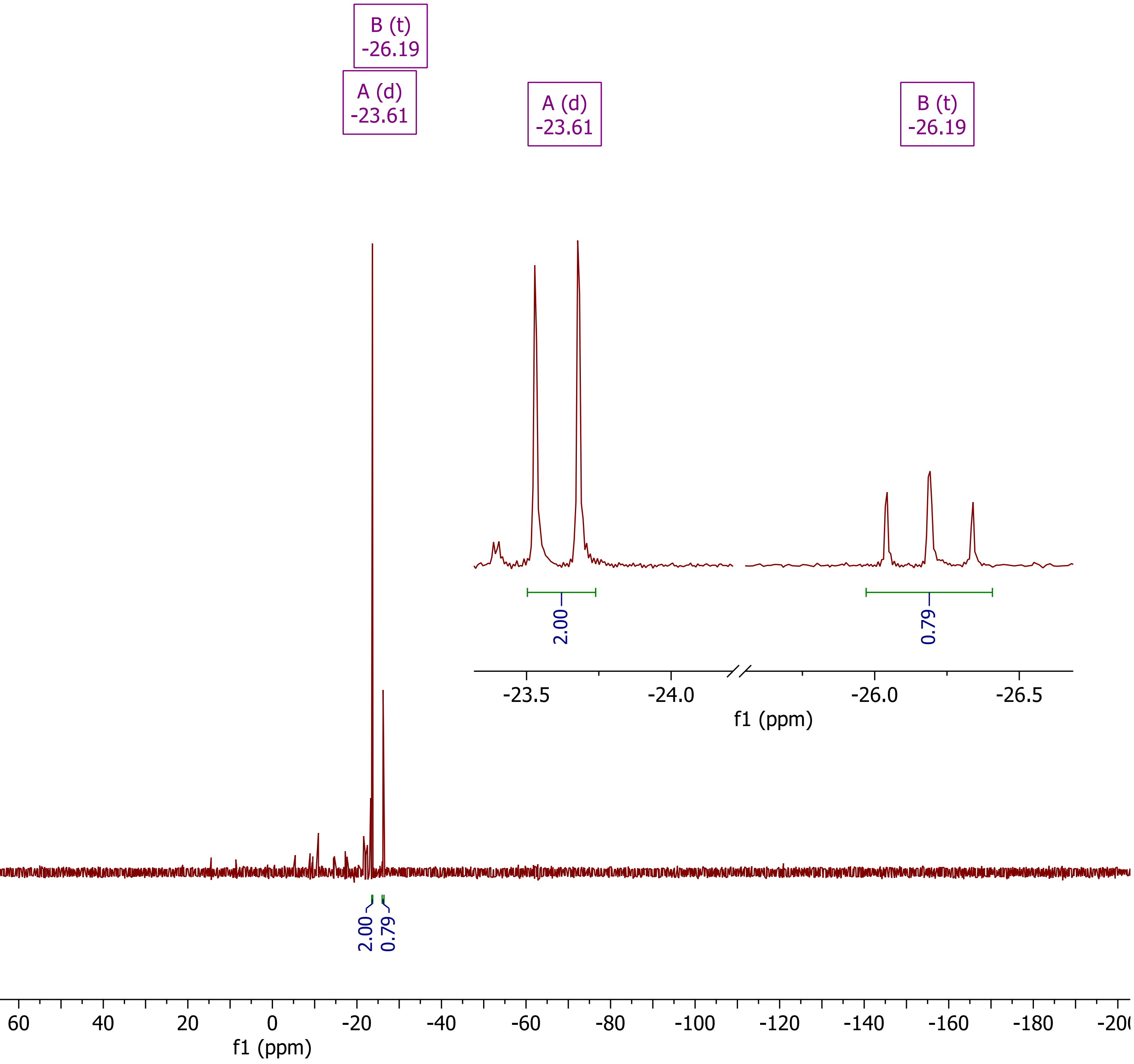

Compound 74, Phenylcyclotetraphosphate, $^{31}P\{^{1}H\}$ - NMR (MeCN-d3, 162 MHz)

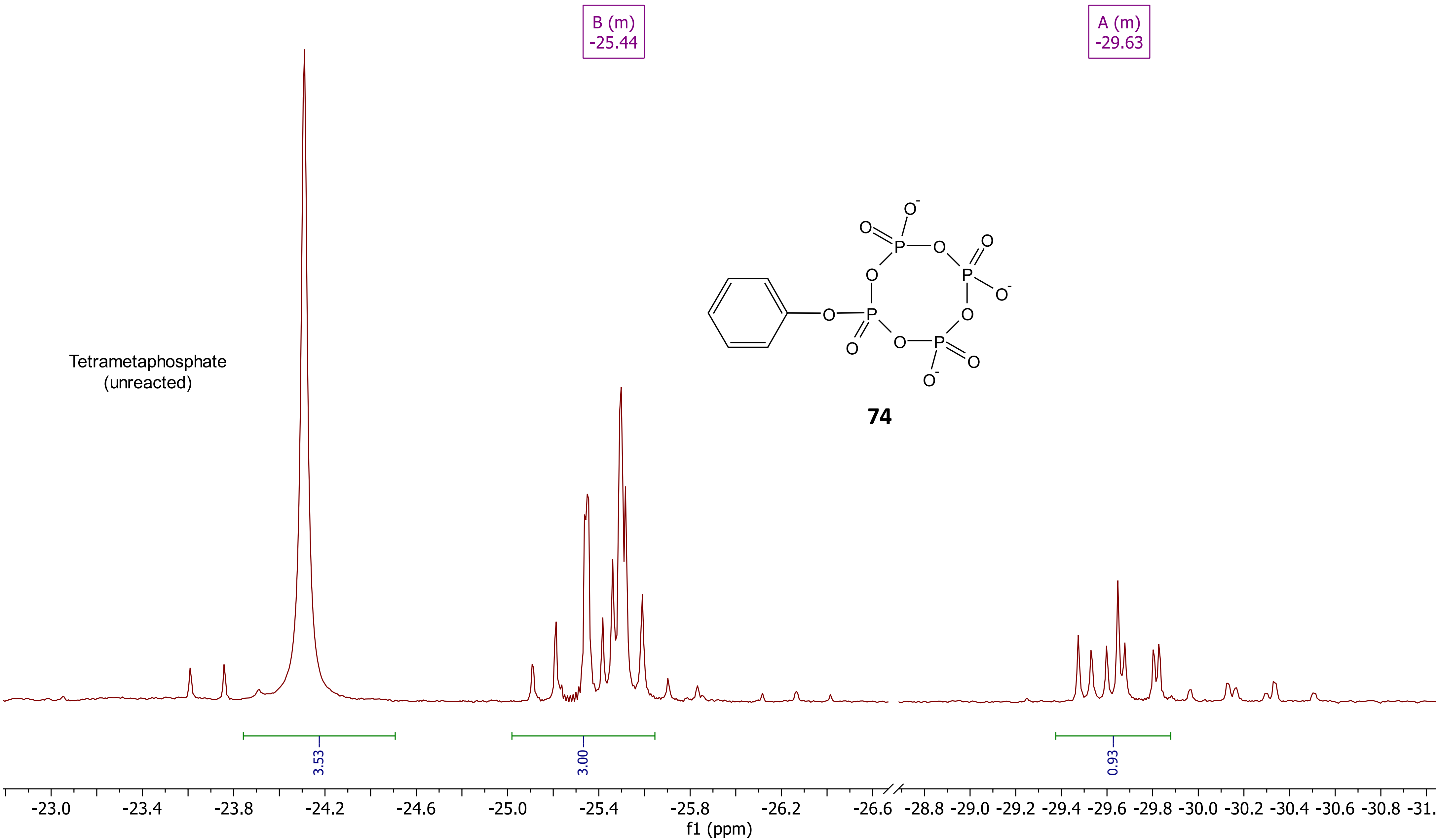

Compound 74, Phenylcyclotetraphosphate, $^{31}P\ \{^{1}H\}$ - NMR (MeCN-d3, 162 MHz)

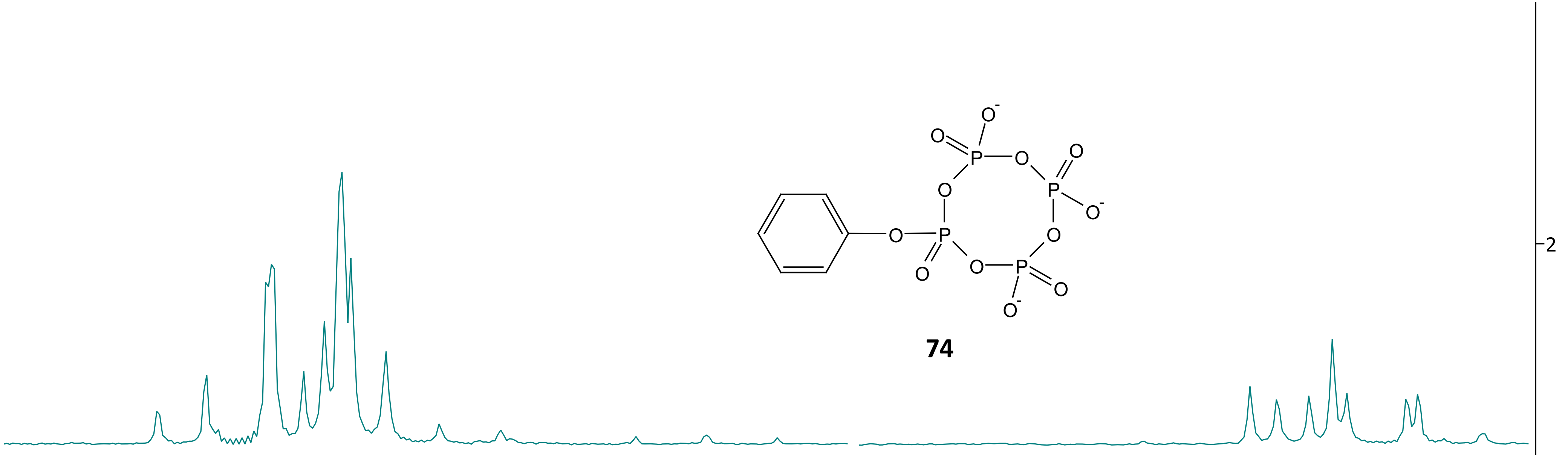

74

Compound 74, Phenylcyclotetraphosphate, $^{31}P\ \{^{1}H\}$ - NMR (spinsimulator)

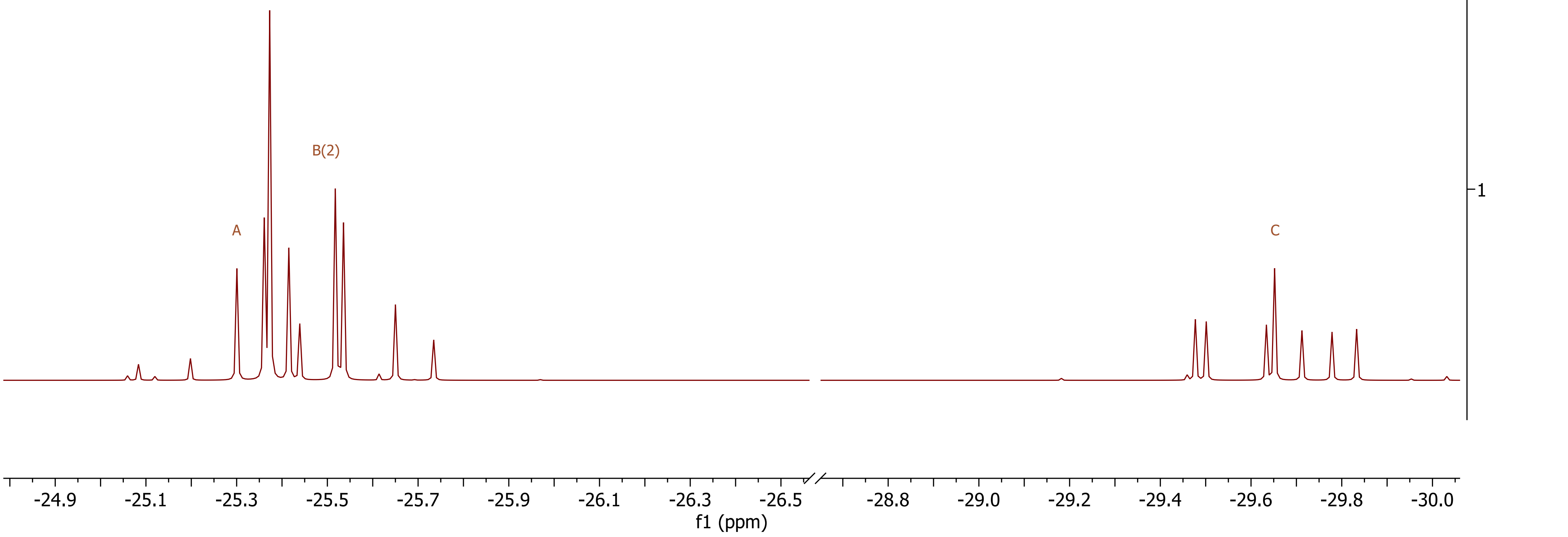

Compound 78, PhenylP$_3$-propargylamidate, ^{1}H - NMR (D_2O, 400 MHz)

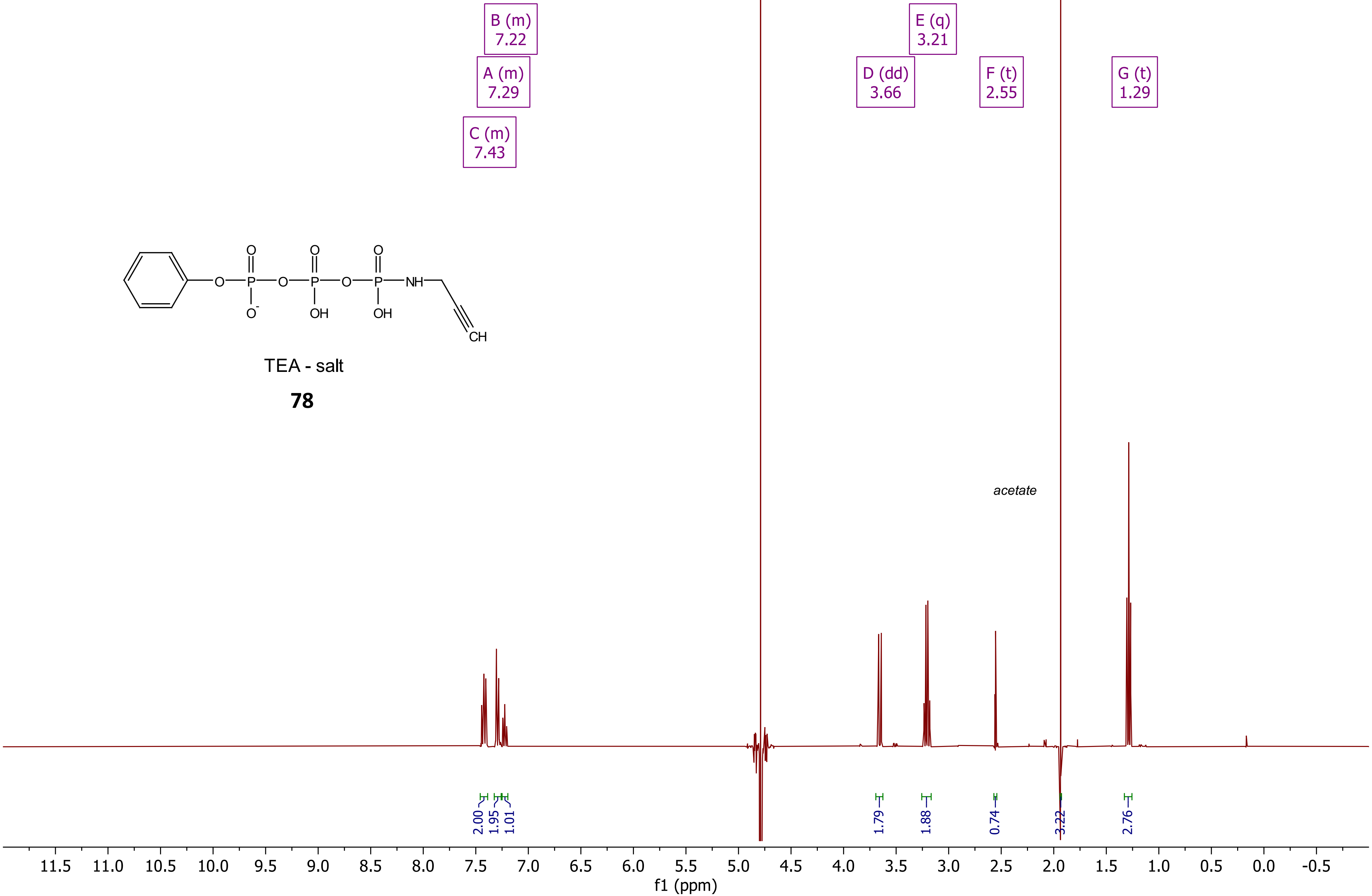

TEA - salt

78

Compound 78, PhenylP$_3$-propargylamidate, ^{31}P {^{1}H} - NMR (D2O, 162 MHz)

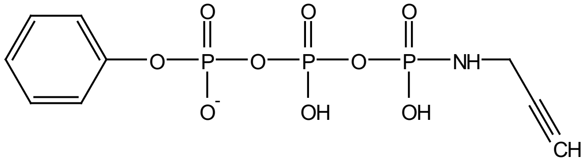

TEA - salt

78

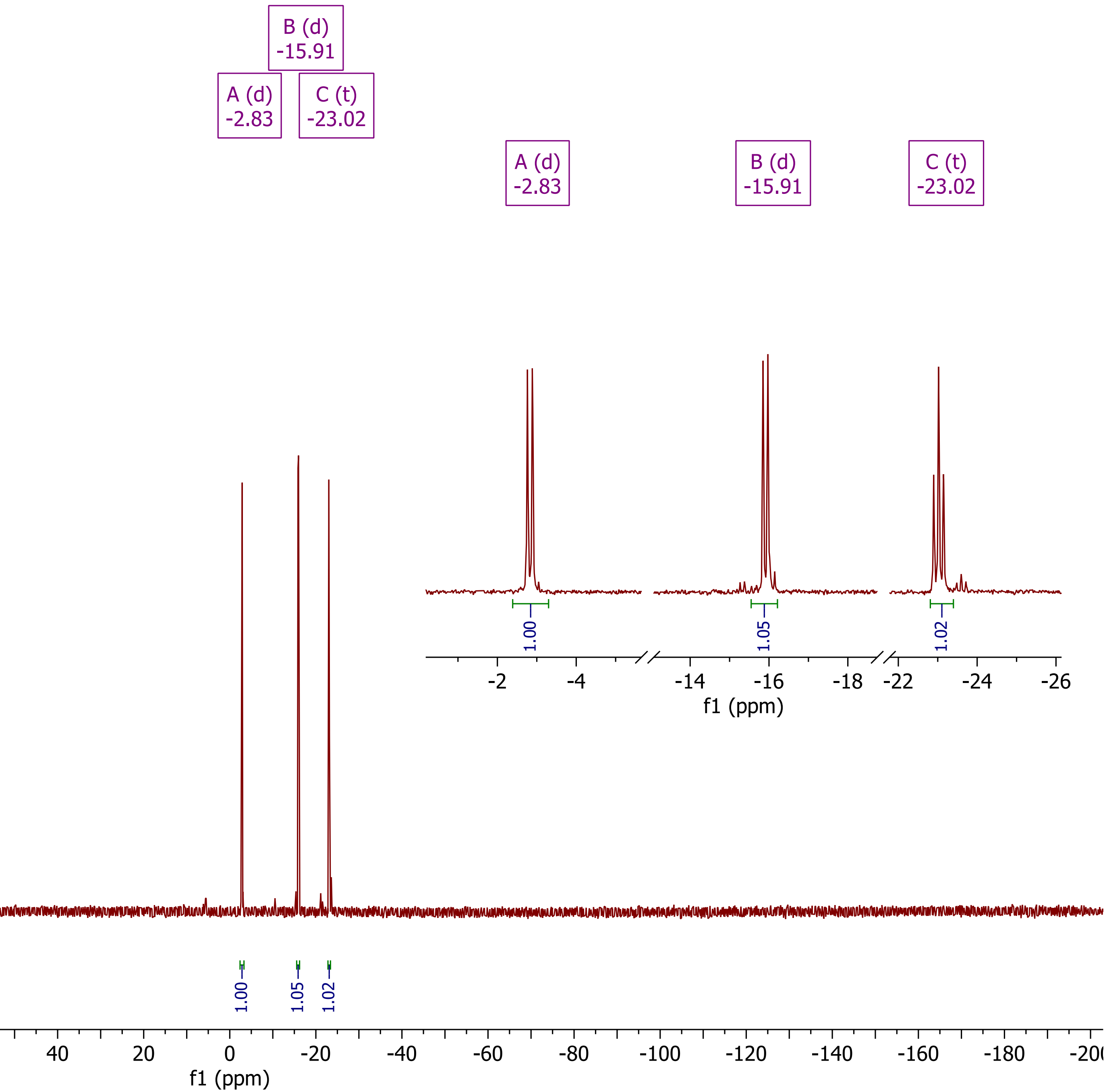

Compound 78, PhenylP$_3$-propargylamidate, ^1H - ^{31}P - HMBC (D2O MHz)

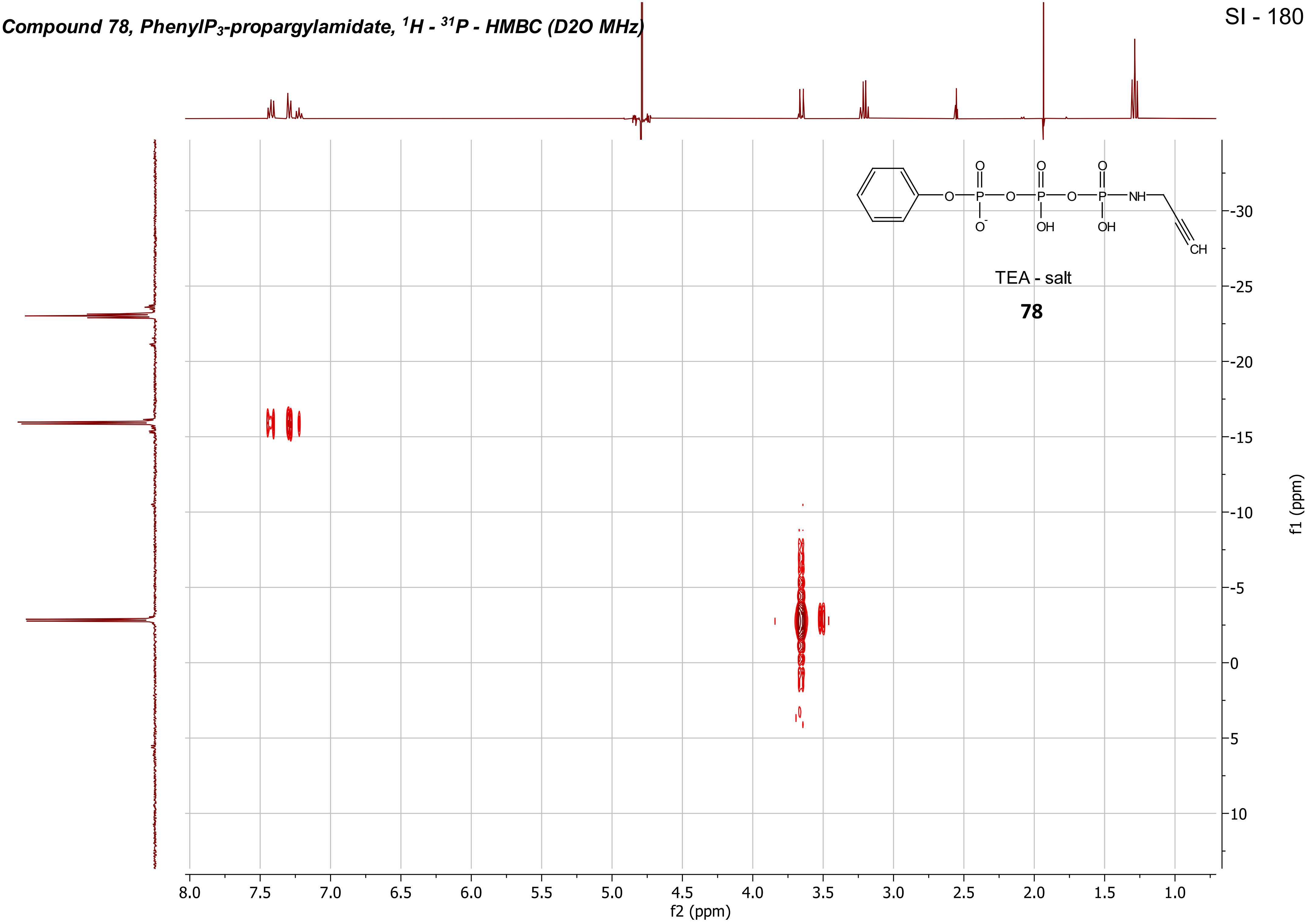

Compound 78, PhenylP$_3$-propargylamidate, $^{13}C\{^1H\}$ - NMR (D2O, 101 MHz)

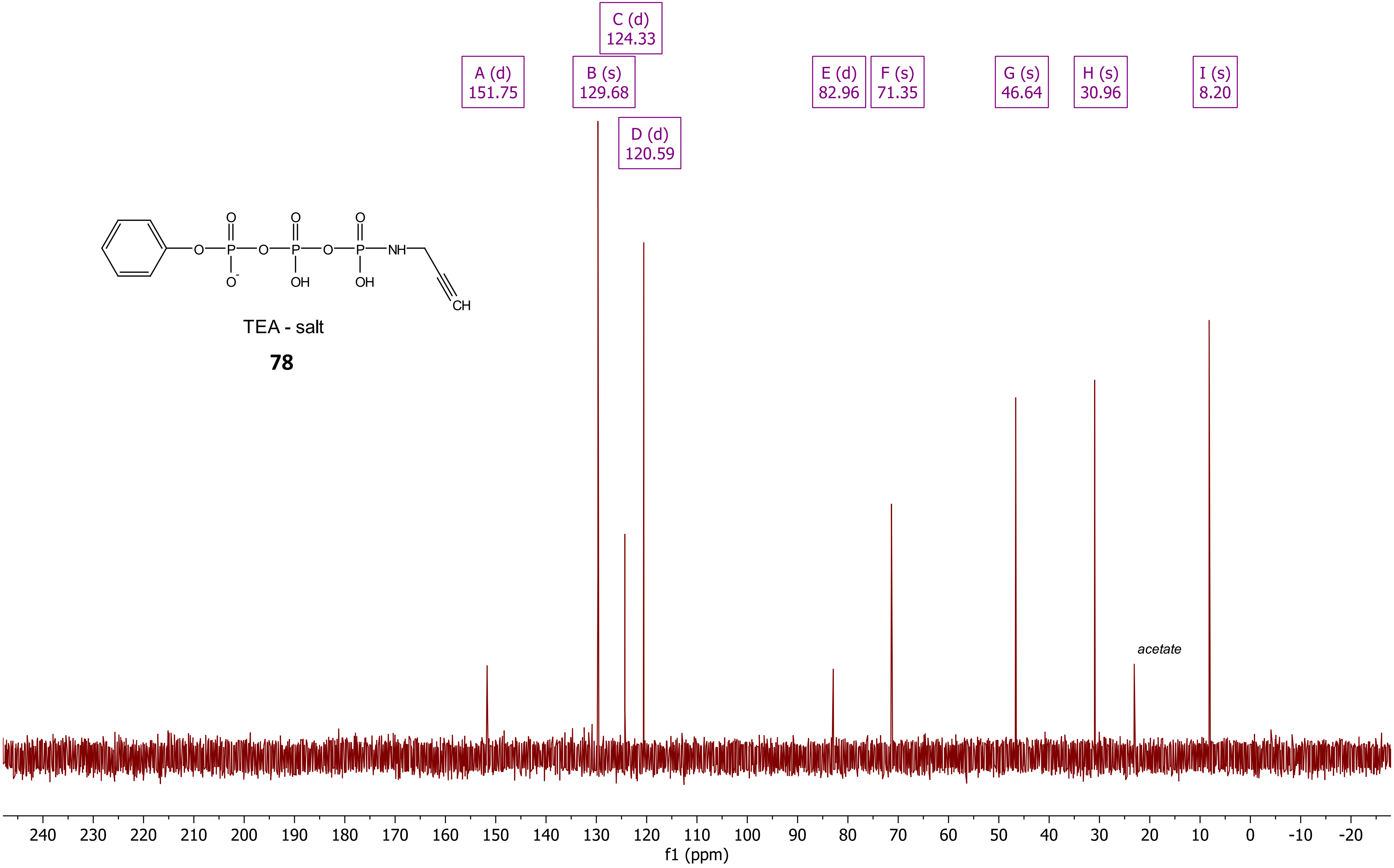

TEA - salt

78

Compound 79, PhenylP$_3$-amidate, ^{1}H - NMR (D2O, 400 MHz)

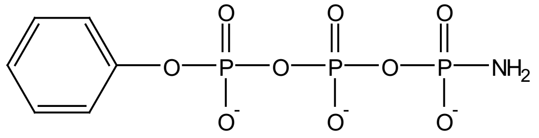

Na - salt

79

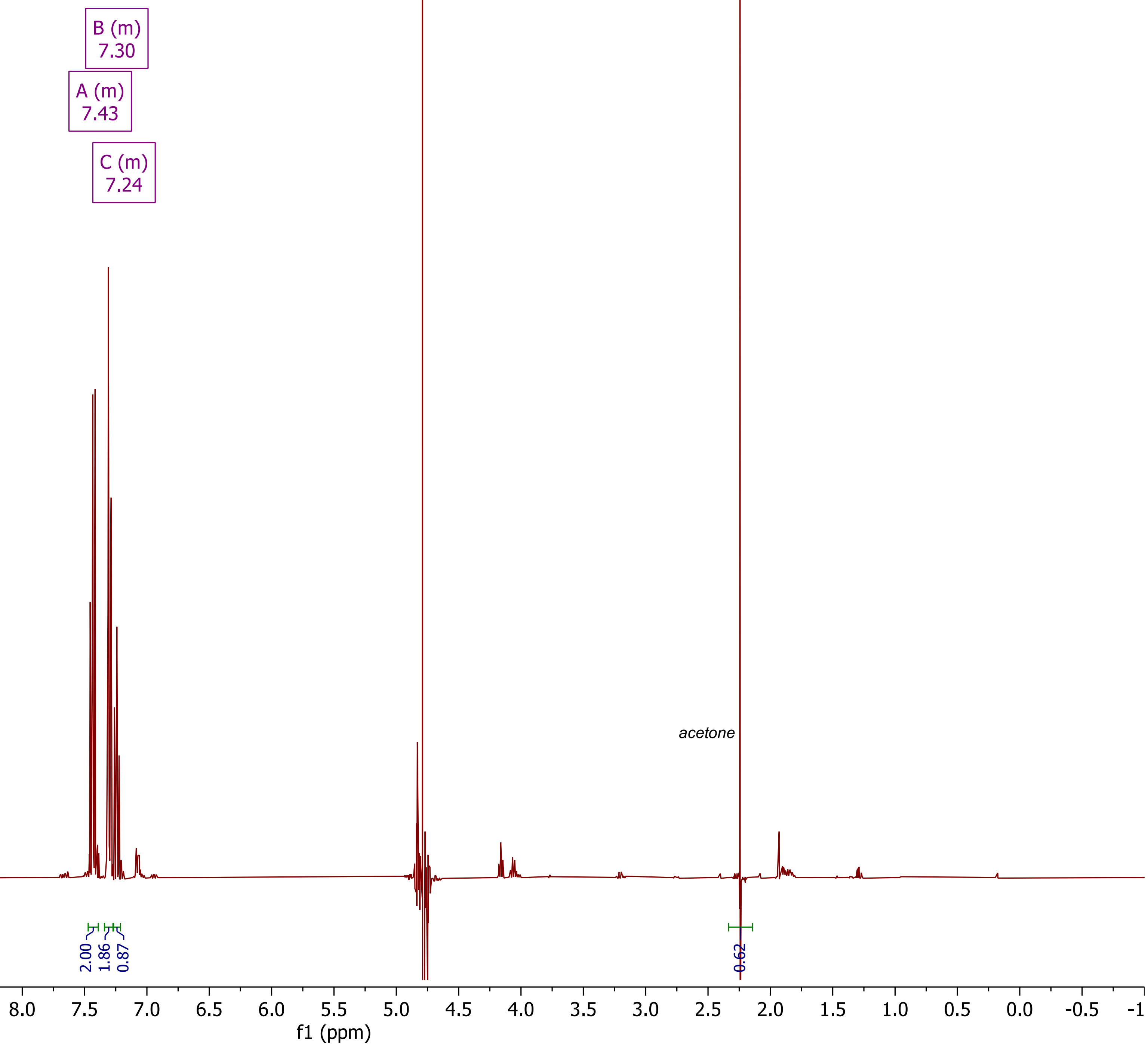

Compound 79, PhenylP$_3$-amidate, ^{31}P {^{1}H} - NMR (D2O, 162 MHz)

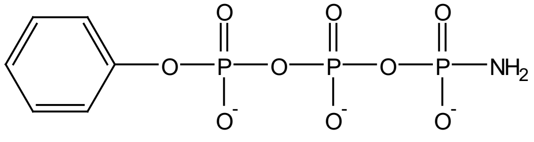

Na - salt

79

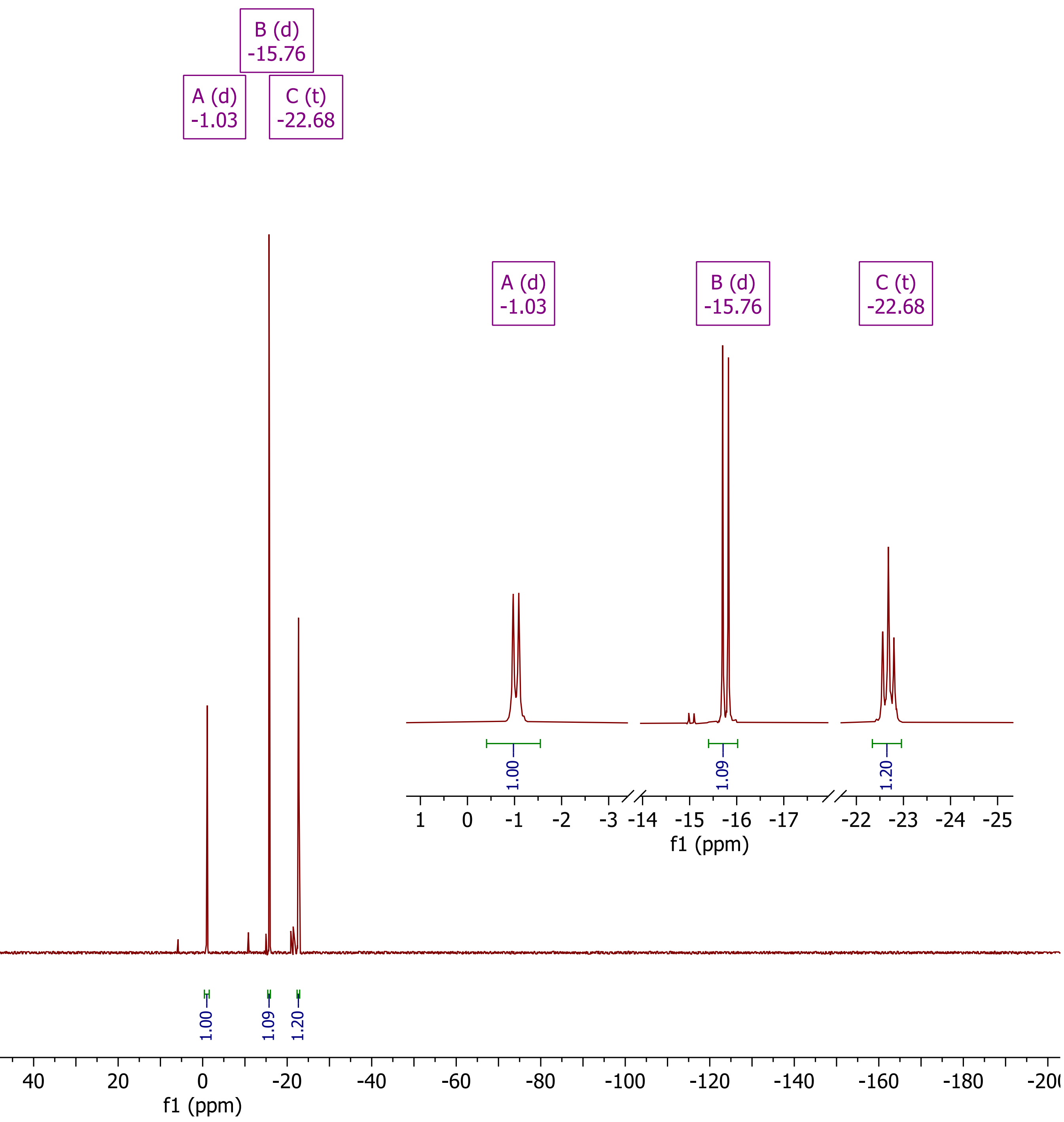

Compound 79, PhenylP$_3$-amidate, $^{13}C\{^{1}H\}$ - NMR (D2O, 101 MHz)

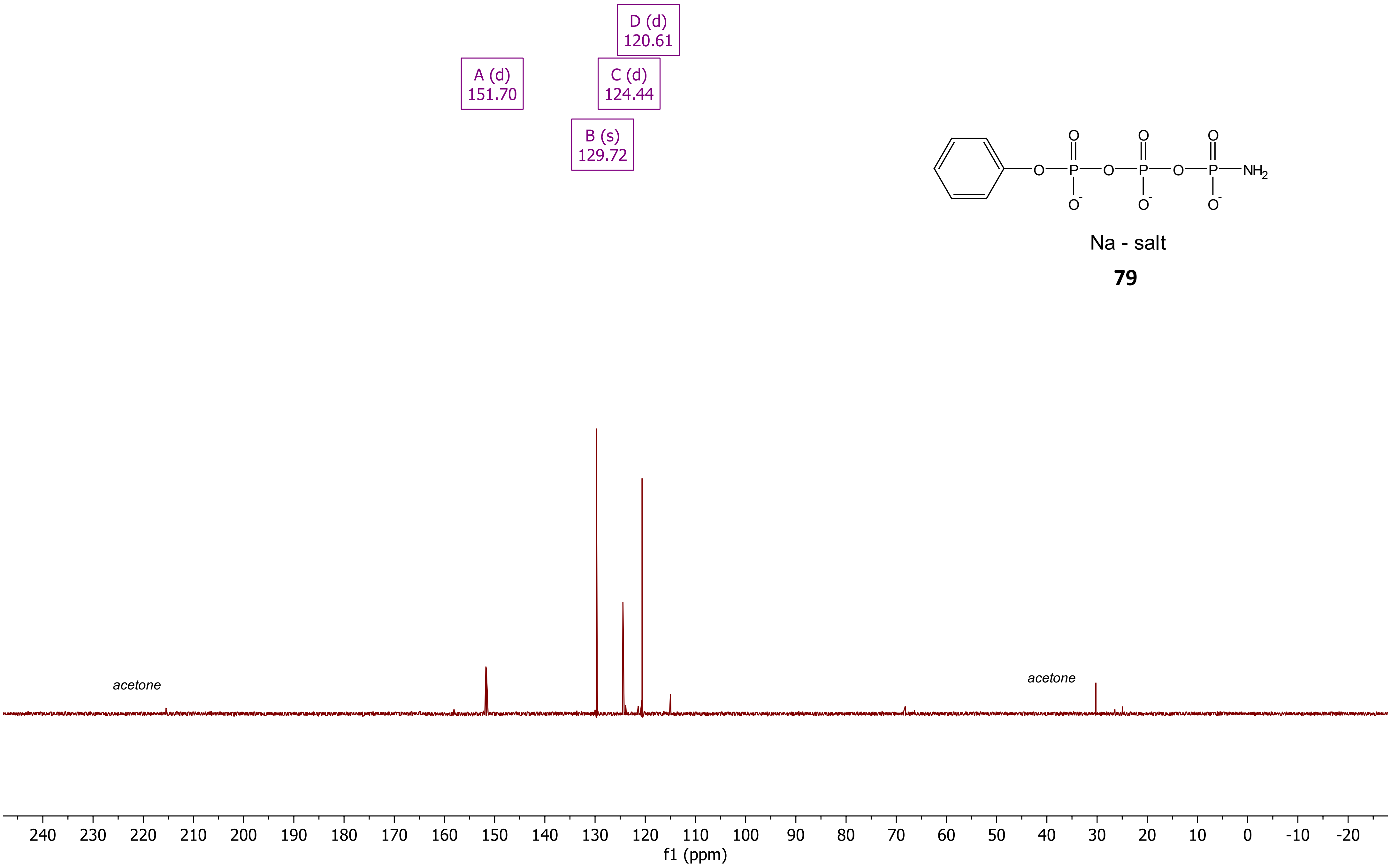

79

Compound 80, PhenylP$_3$- anthracen-9-ylmethanamidate, ^{1}H - NMR (D2O, 400 MHz)

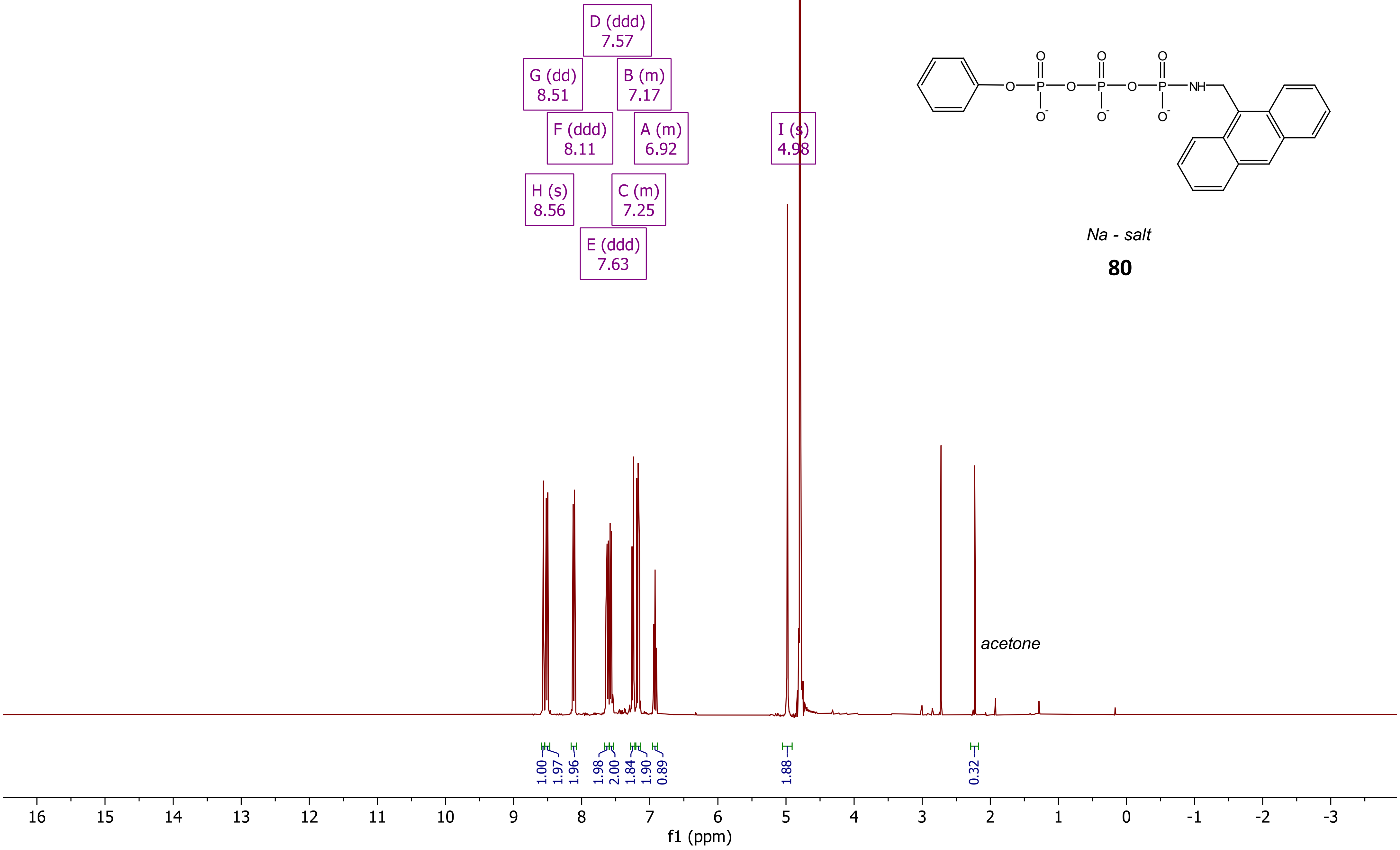

Compound 80, PhenylP$_3$- anthracen-9-ylmethanamidate, ^{31}P {^{1}H} - NMR (D2O, 162 MHz)

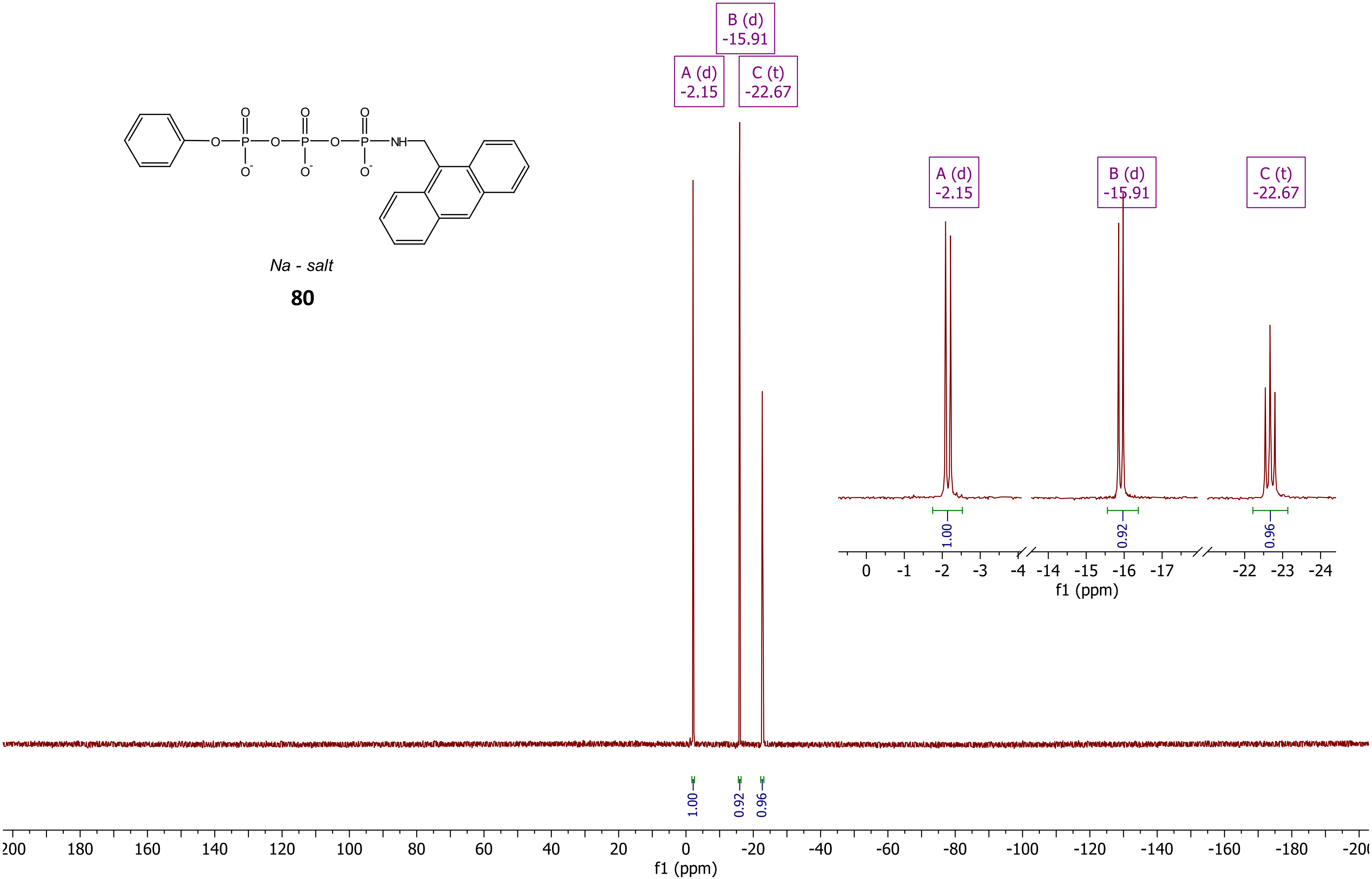

Compound 80, PhenylP$_3$- anthracen-9-ylmethanamidate, ^{13}C{^{1}H} - NMR (D2O, 101 MHz)

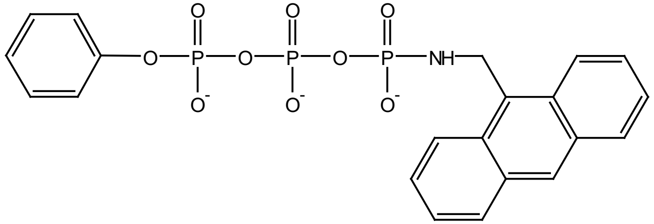

Na - salt

80

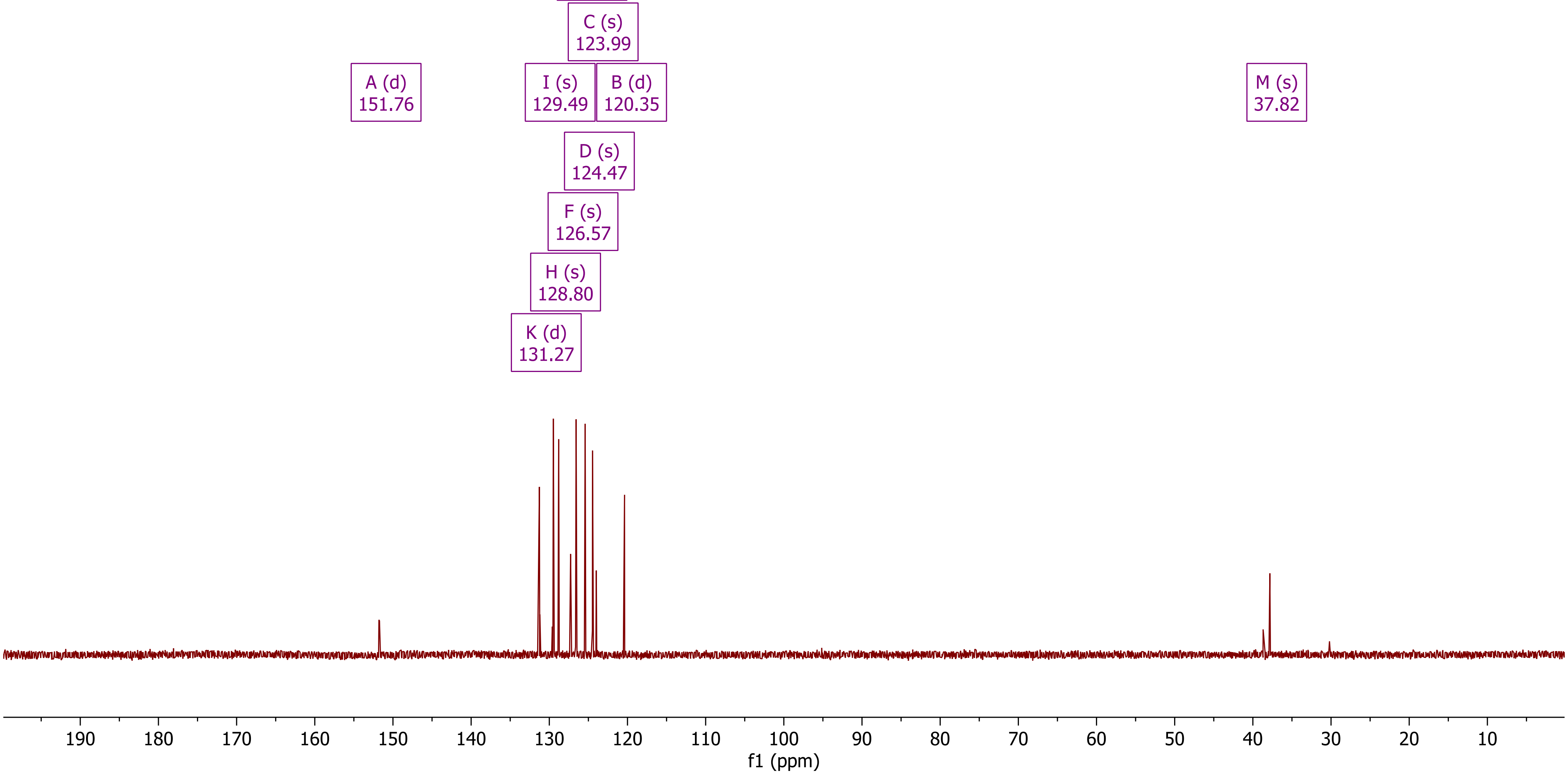

Compound 81, PhenylP$_3$-5'-deoxyadenosyl-5'-amidate, ^{1}H - NMR (D$_2$O, 400 MHz)

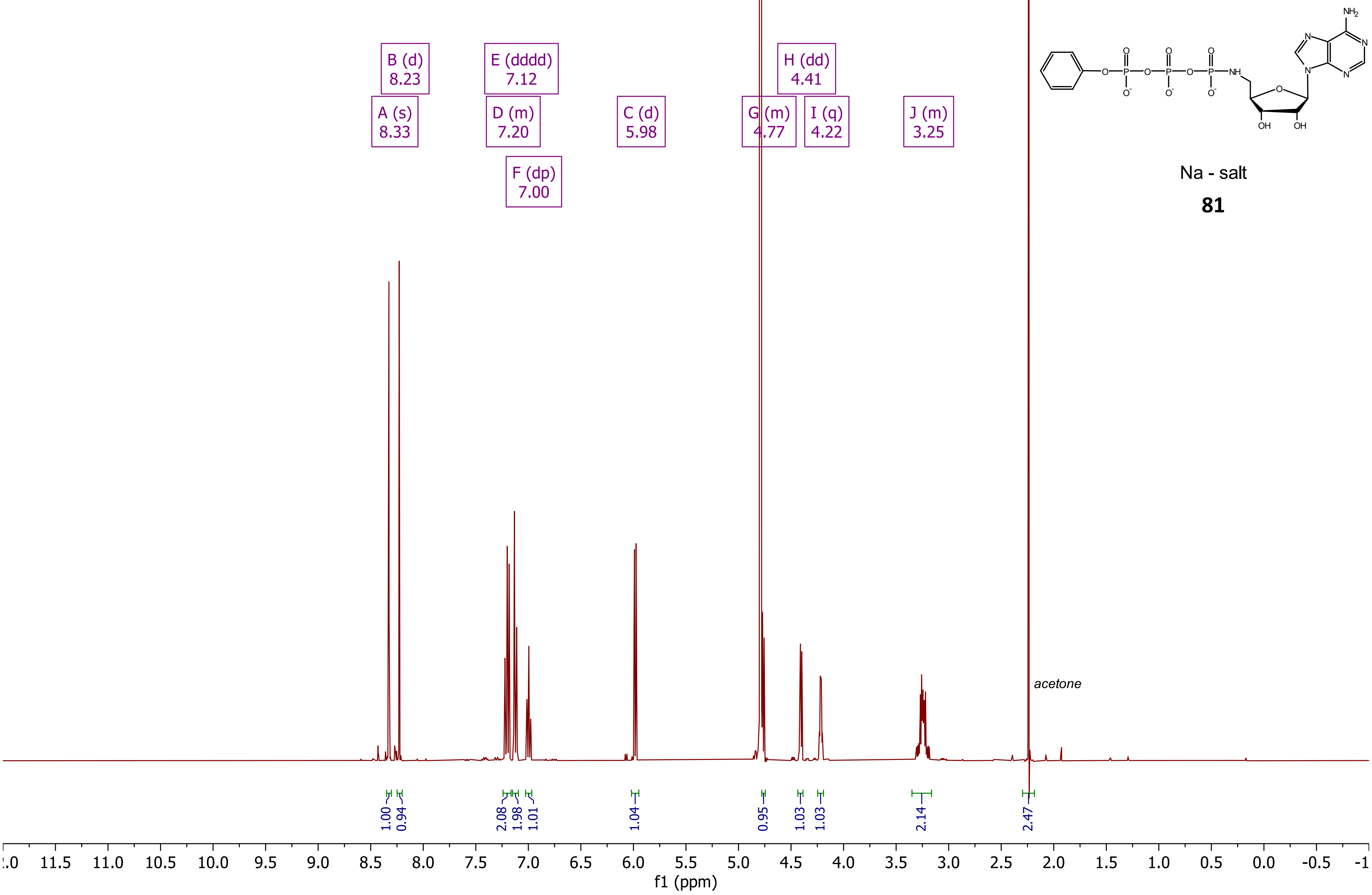

Compound 81, PhenylP$_3$-5'-deoxyadenosyl-5'-amidate, ^{31}P {^{1}H} - NMR (D2O, 162 MHz)

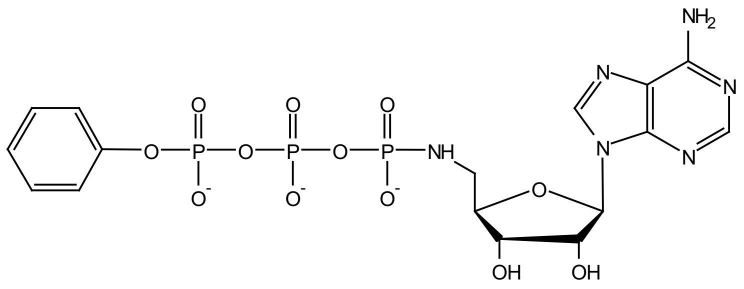

81

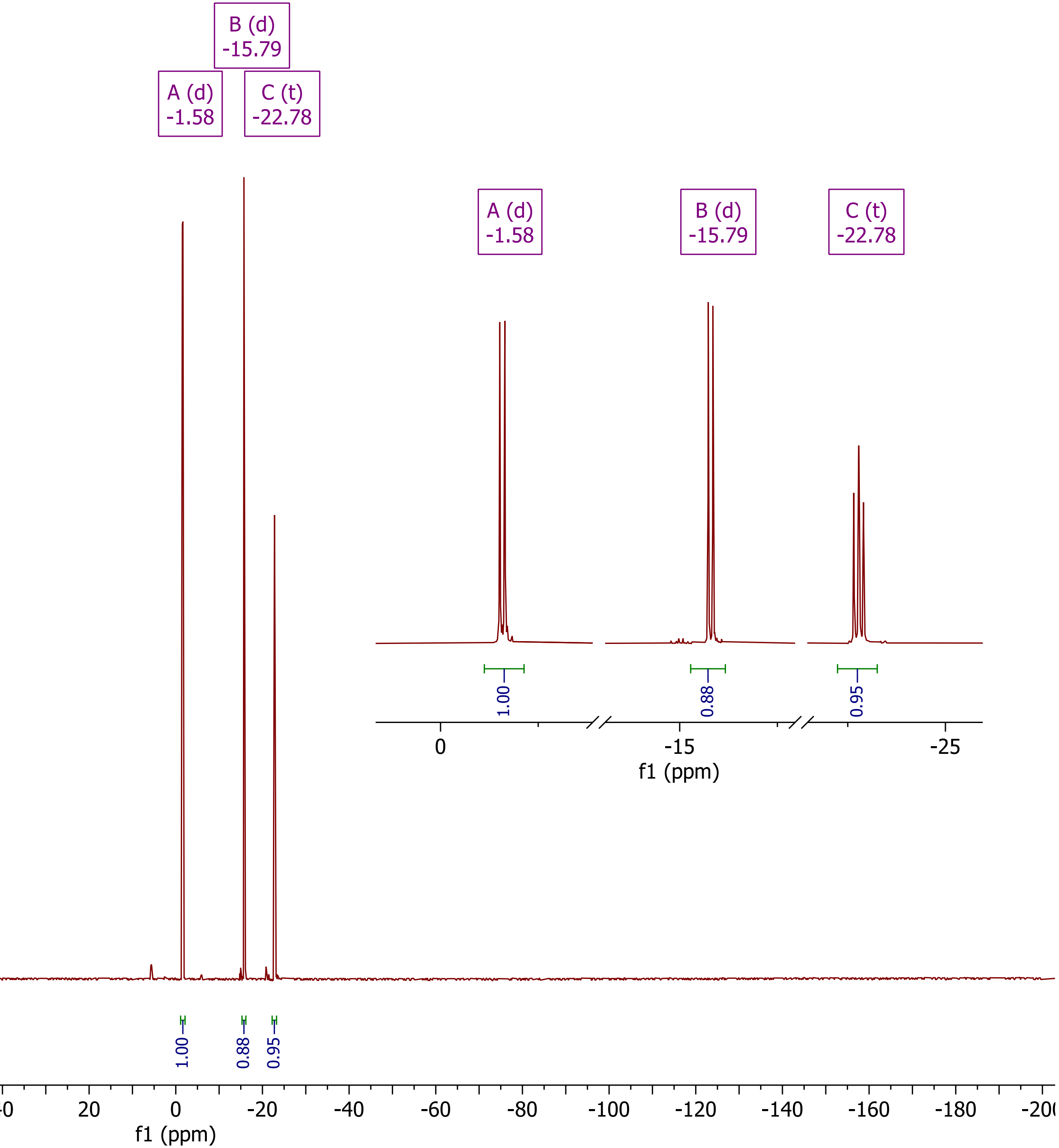

Compound 81, PhenylP$_3$-5'-deoxyadenosyl-5'-amidate, DQF-COSY (D2O)

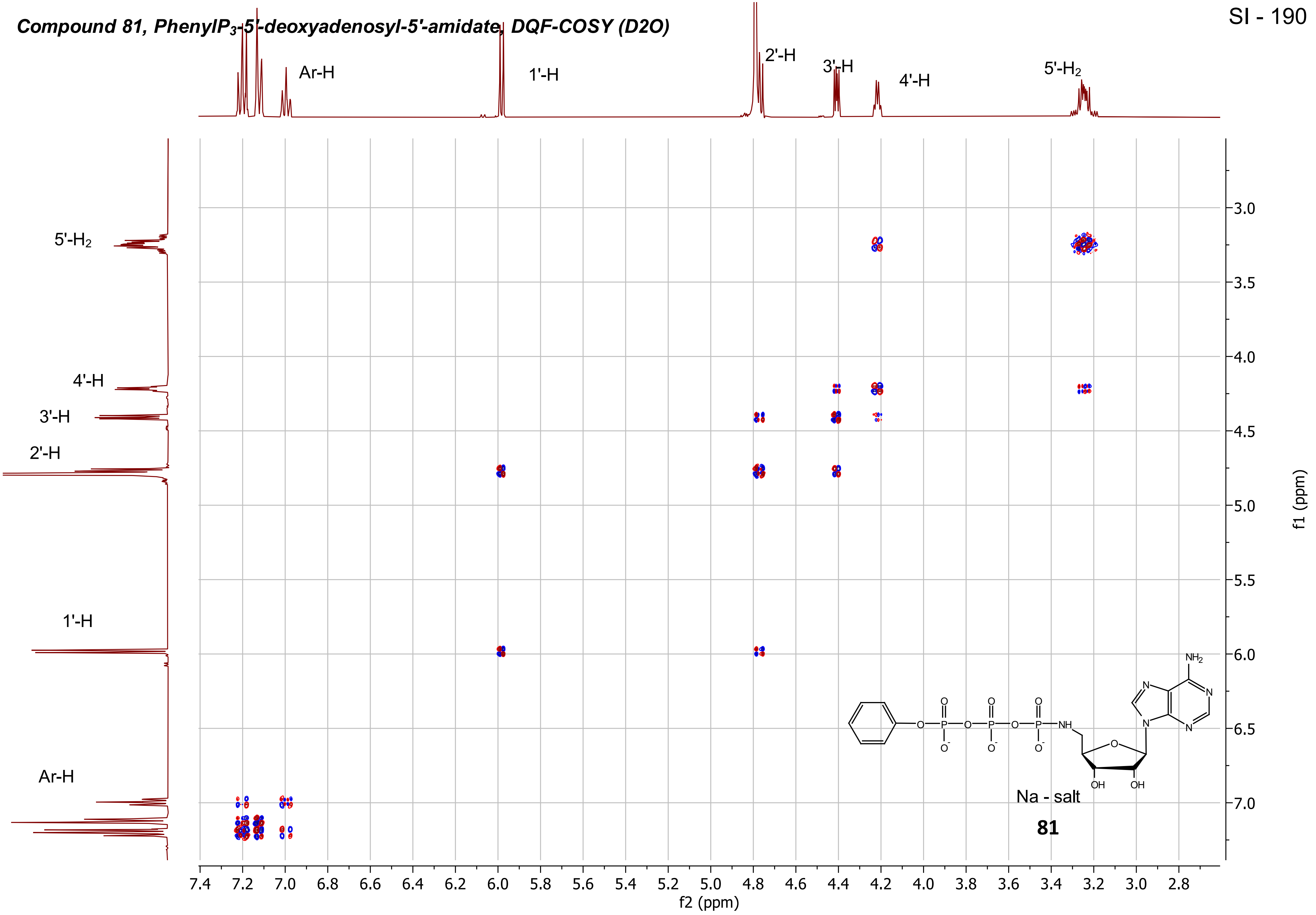

Compound 81, PhenylP$_3$-5'-deoxyadenosyl-5'-amidate, HSQC (D2O)

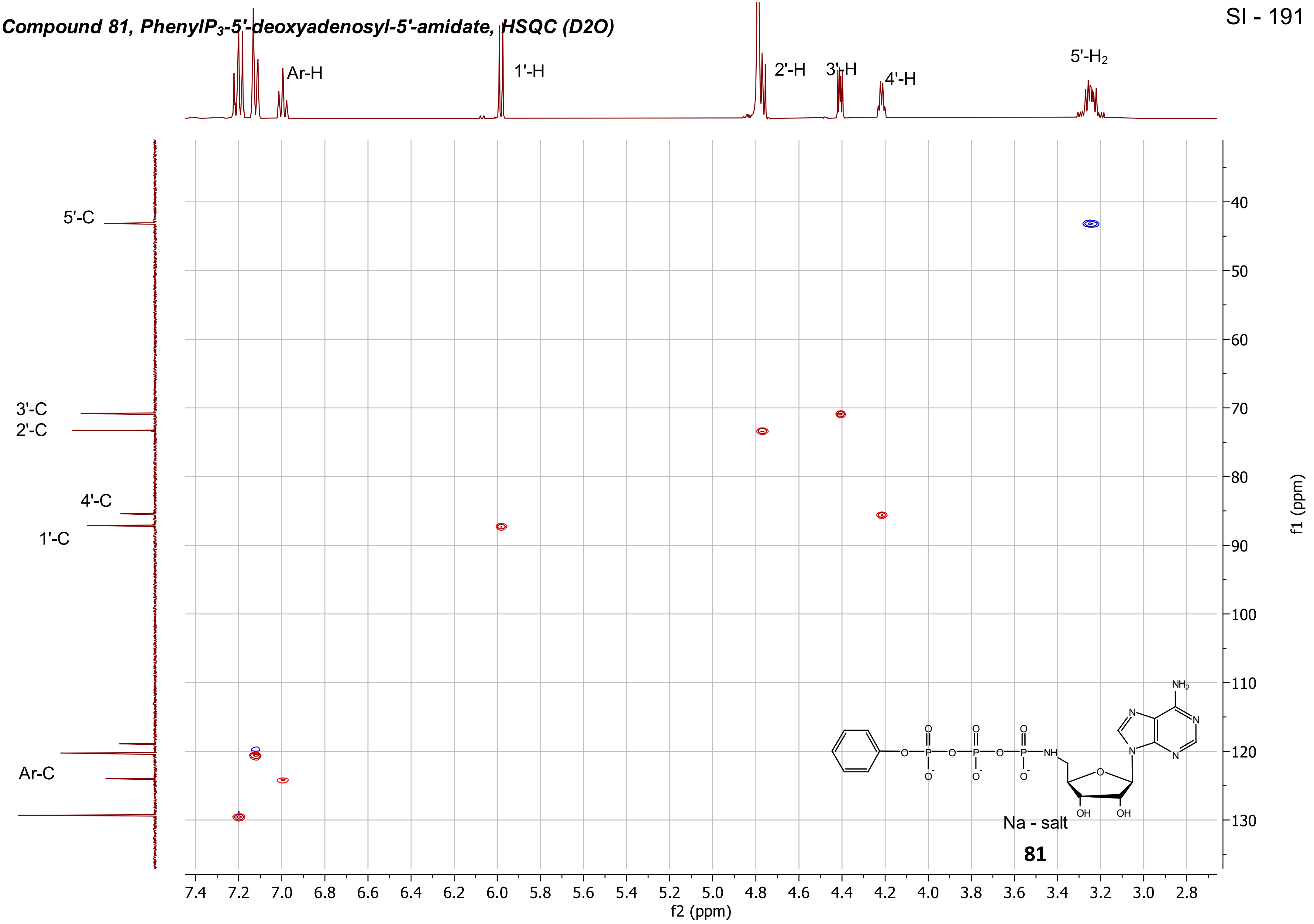

Compound 81, PhenylP$_3$-5'-deoxyadenosyl-5'-amidate, ^{1}H - ^{31}P - HMBC (D2O)

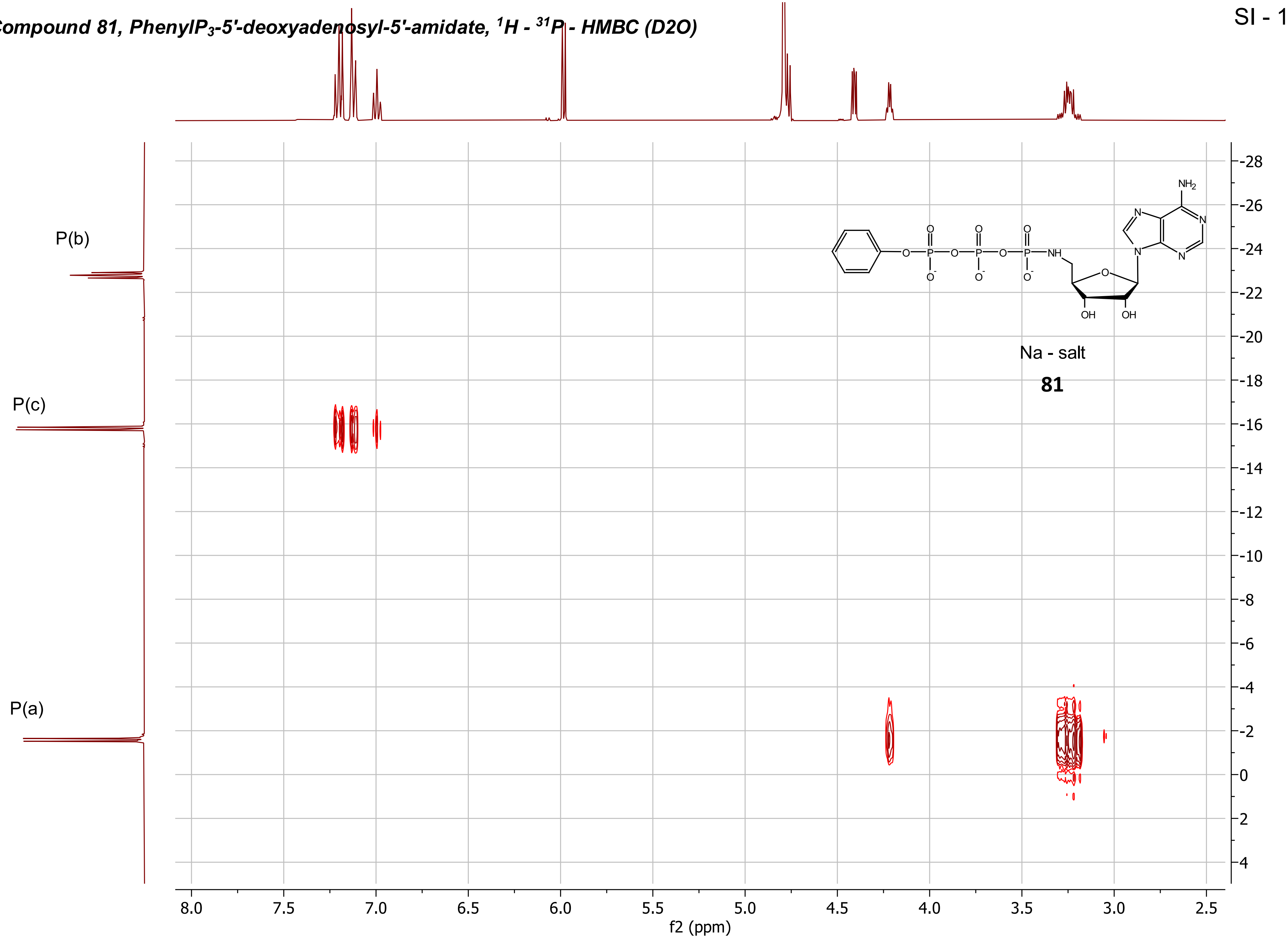

Compound 81, PhenylP$_3$-5'-deoxyadenosyl-5'-amidate, $^{13}C\{^1H\}$ - NMR (D2O, 101 MHz)

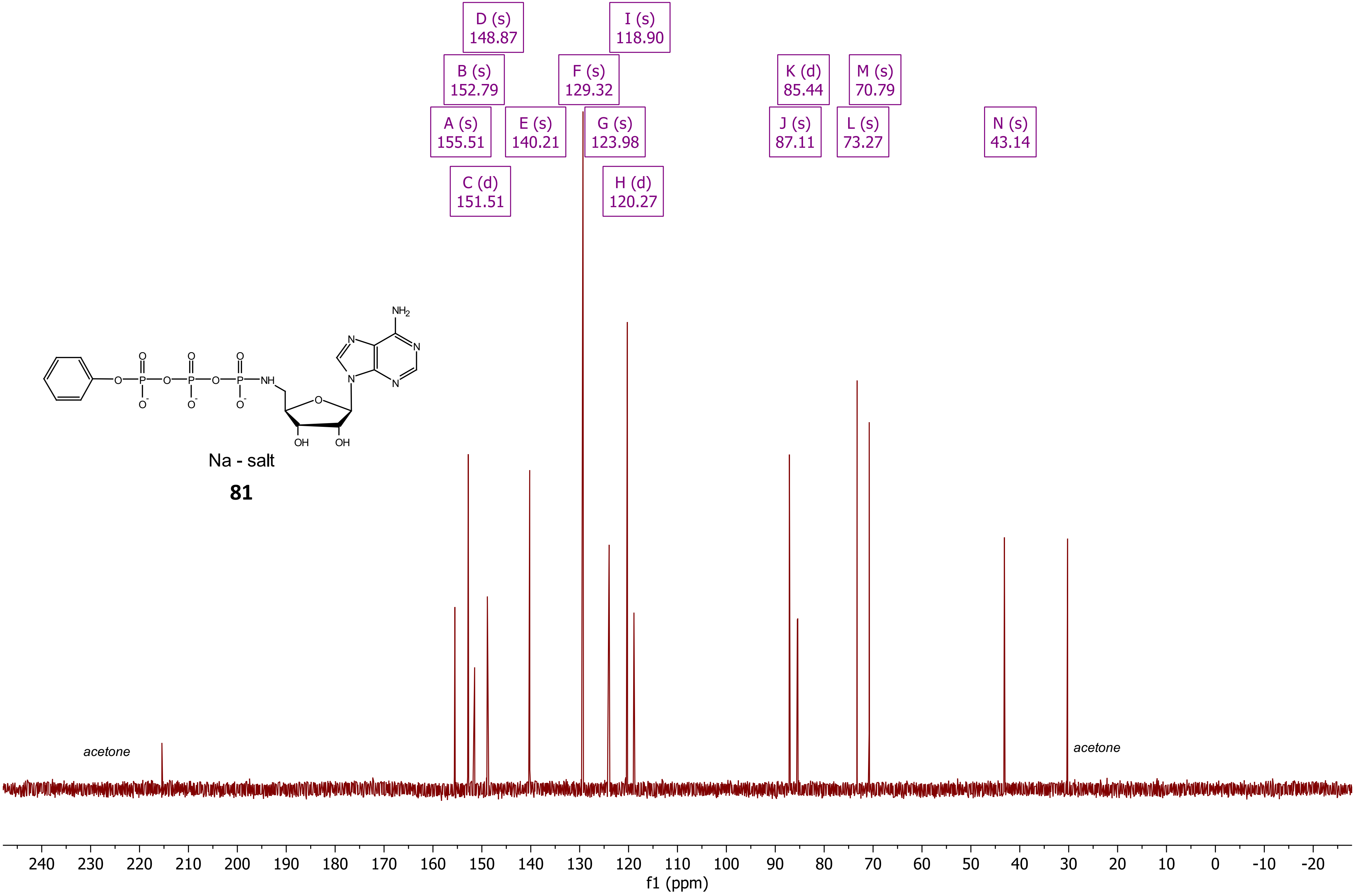

Na - salt

81

Compound 82, PhenylP$_3$-5'-deoxyguanosyl-5'-amidate, ^{1}H - NMR (D$_2$O, 400 MHz)

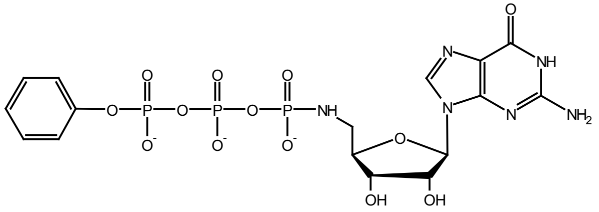

Na - salt

82

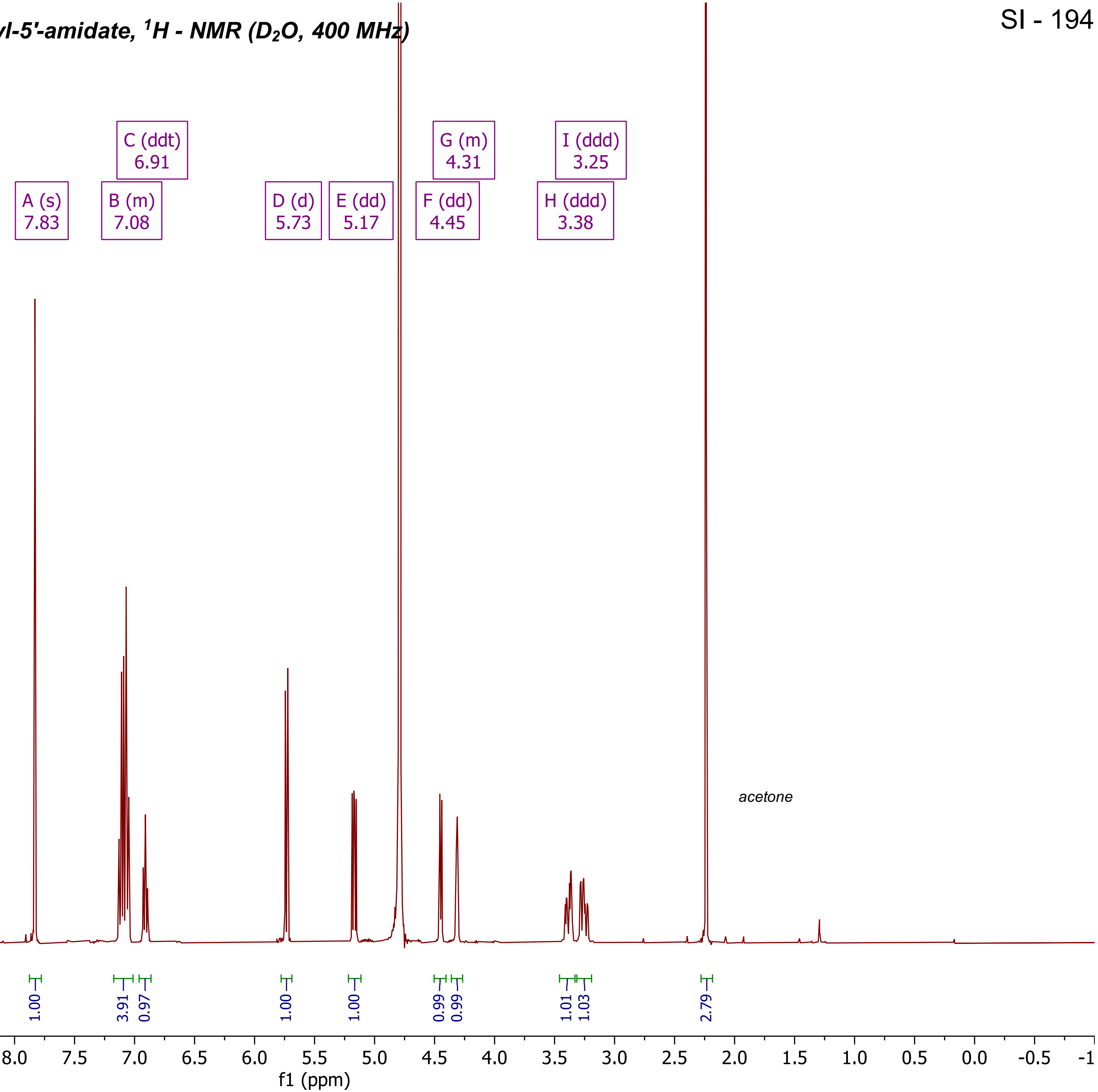

Compound 82, PhenylP$_3$-5'-deoxyguanosyl-5'-amidate, ^{31}P {^{1}H} - NMR (D2O, 162 MHz)

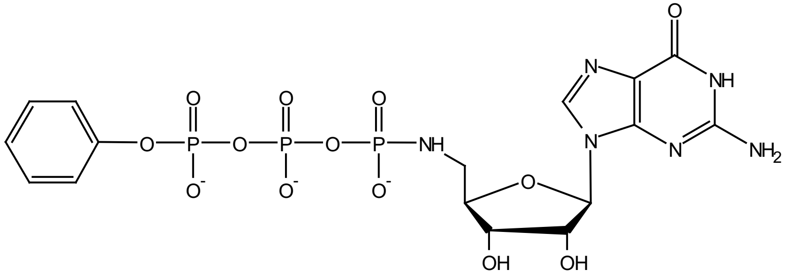

Na - salt

82

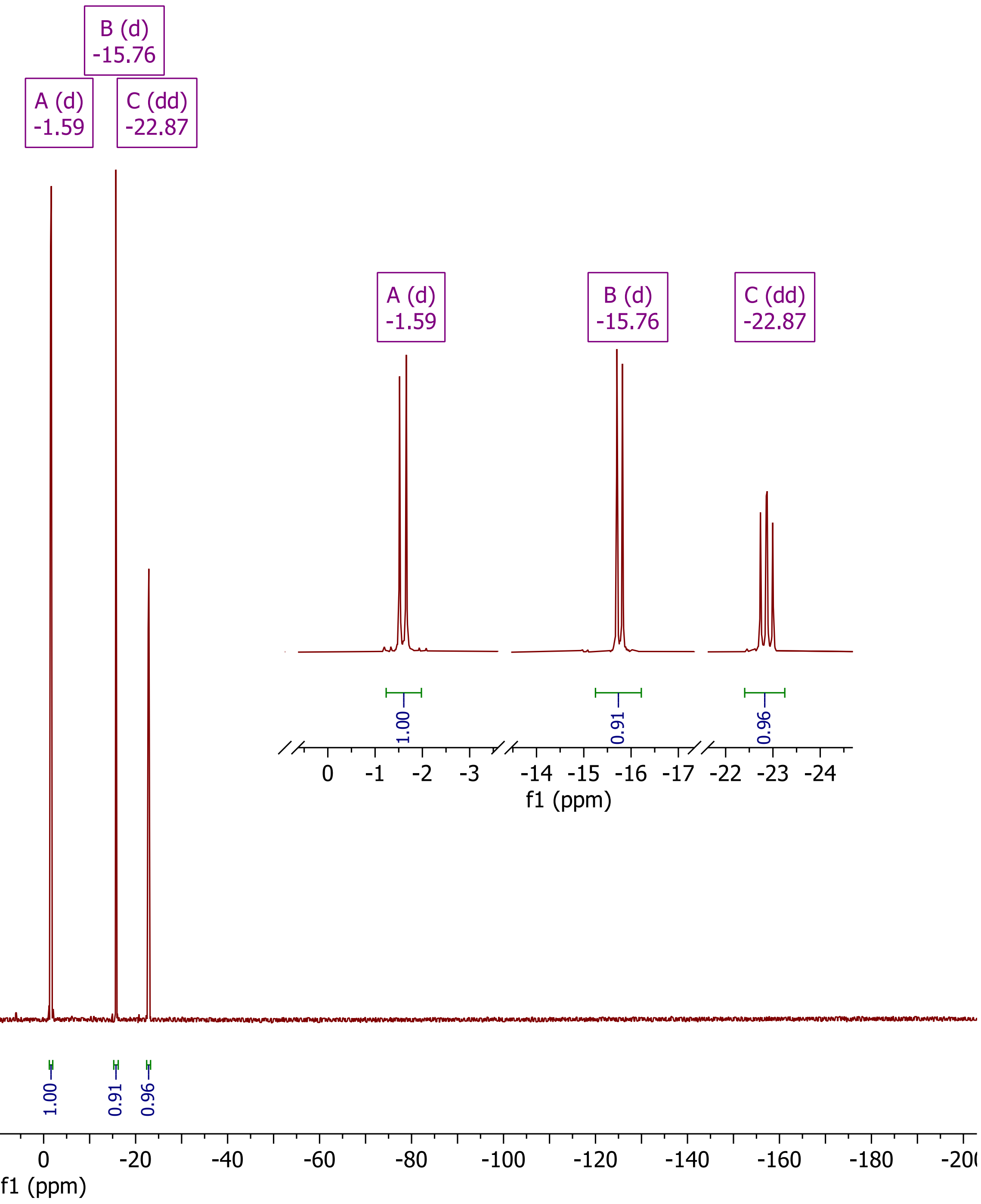

Compound 82, PhenylP$_3$-5'-deoxyguanosyl-5'-amidate, DQF-COSY (D2O)

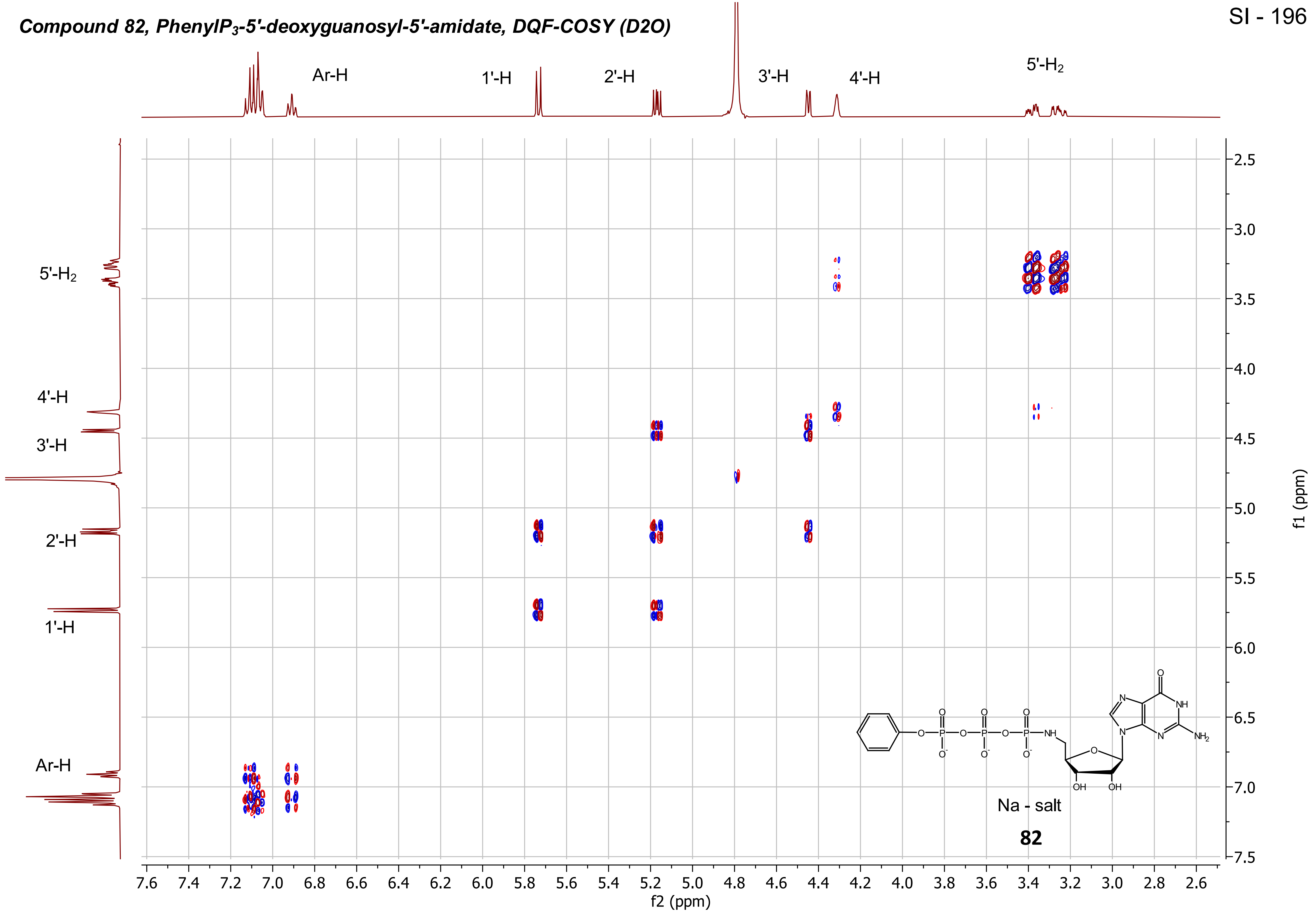

Compound 82, PhenylP$_3$-5'-deoxyguanosyl-5'-amidate, HSQC (D2O)

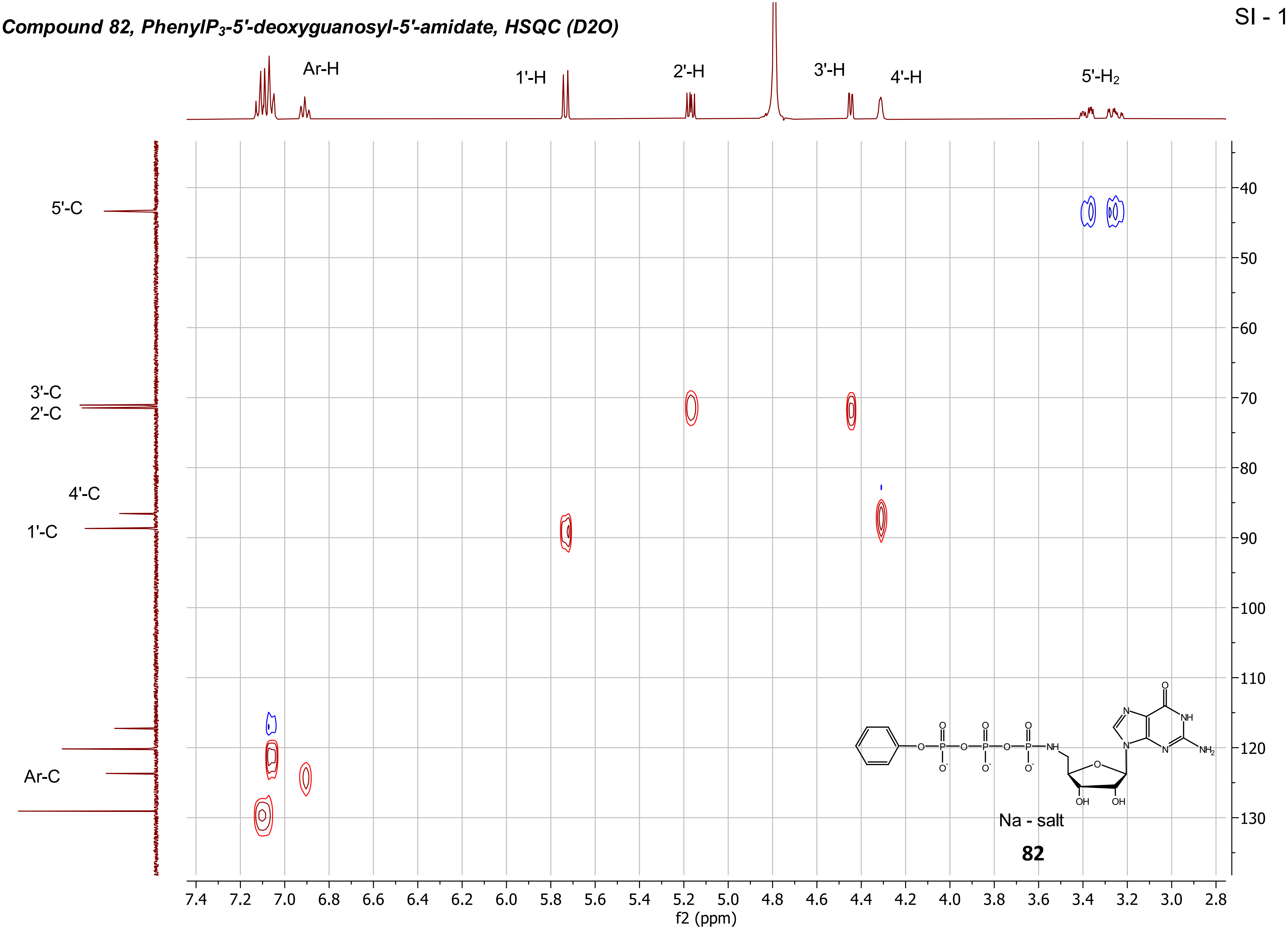

Compound 82, PhenylP$_3$-5'-deoxyguanosyl-5'-amidate, ^{1}H - ^{31}P - HMBC (D2O)

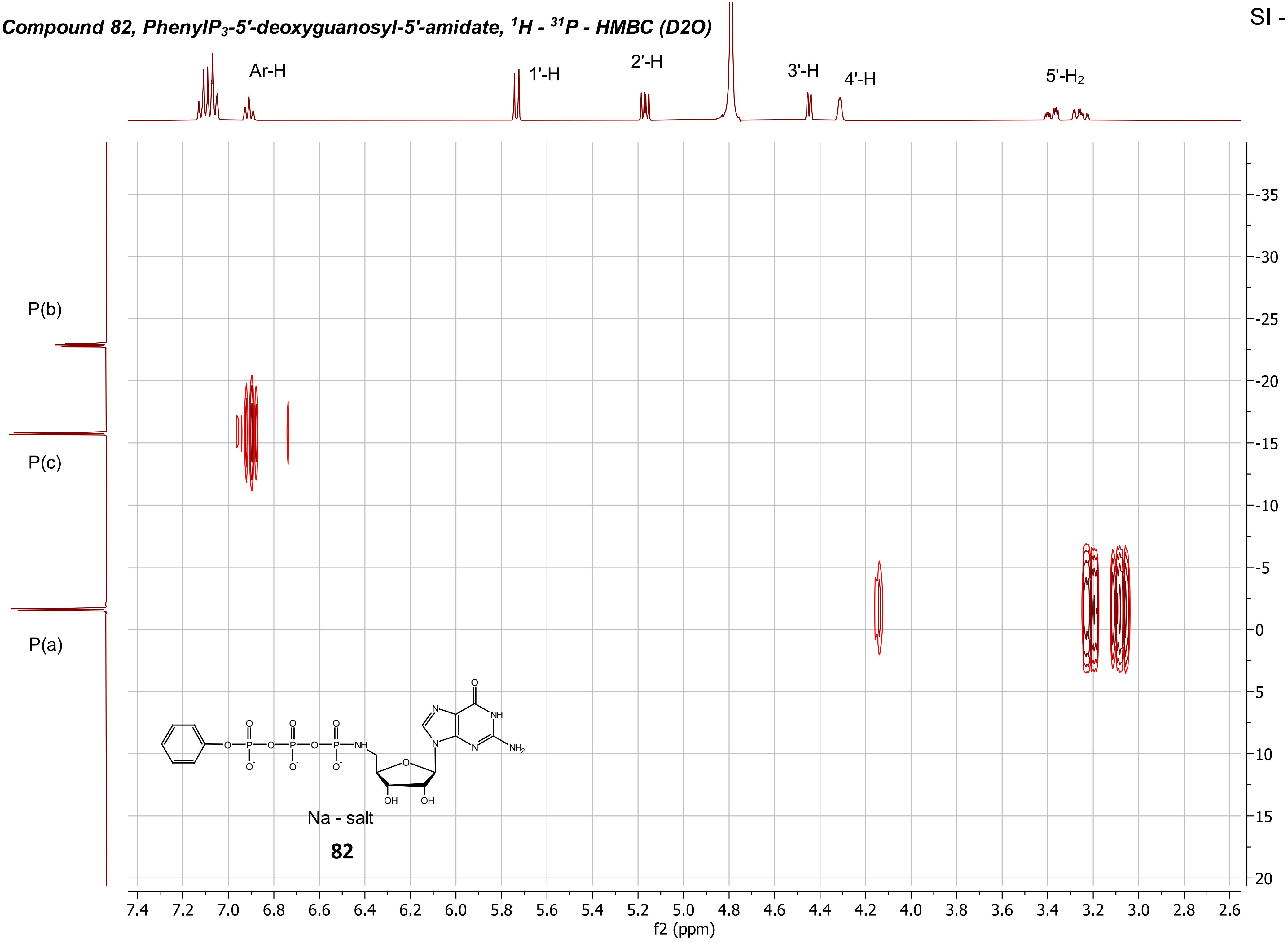

Compound 82, PhenylP$_3$-5'-deoxyguanosyl-5'-amidate, ^{13}C{^{1}H} - NMR (D2O, 101 MHz)

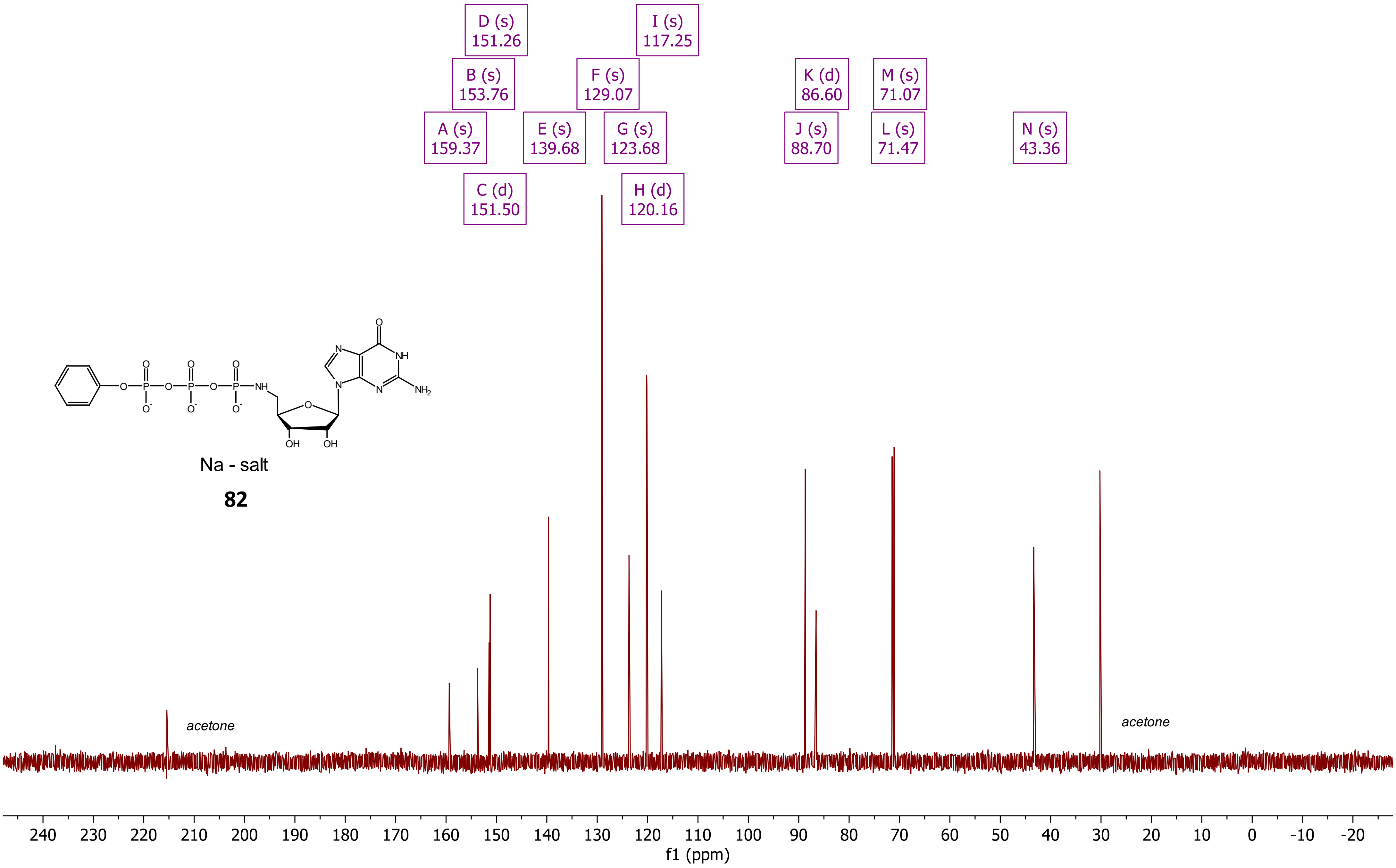

Compound 83, Napht-2-ylP$_3$-5'-deoxyguanosyl-5'-amidate, ^{1}H - NMR (D2O, 400 MHz)

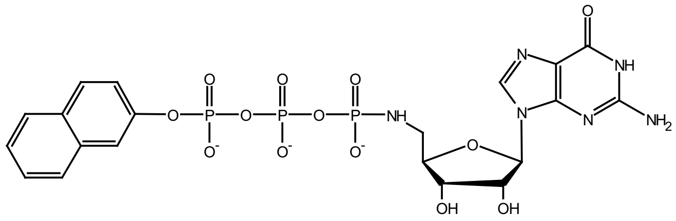

Na - salt

83

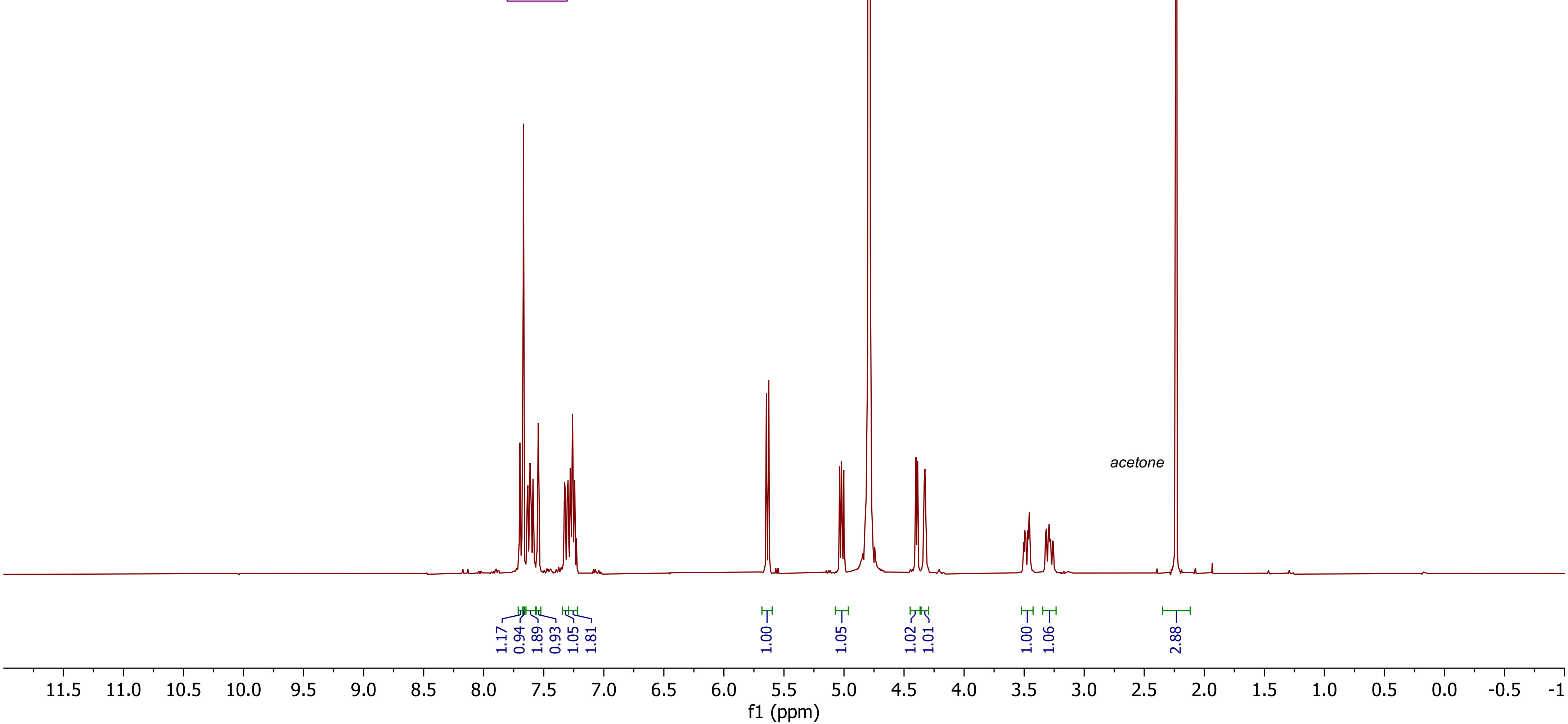

Compound 83, Napht-2-ylP$_3$-5'-deoxyguanosyl-5'-amidate, ^{31}P {^1H} - NMR (D2O, 162 MHz)

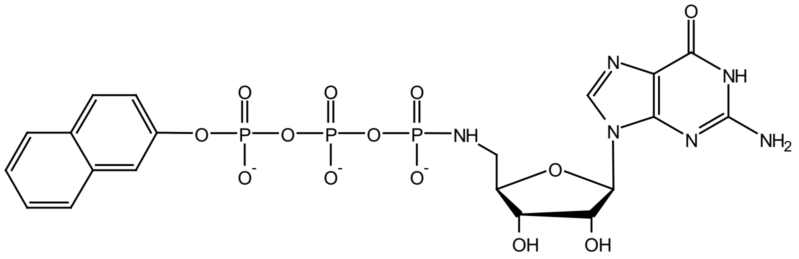

Na - salt

83

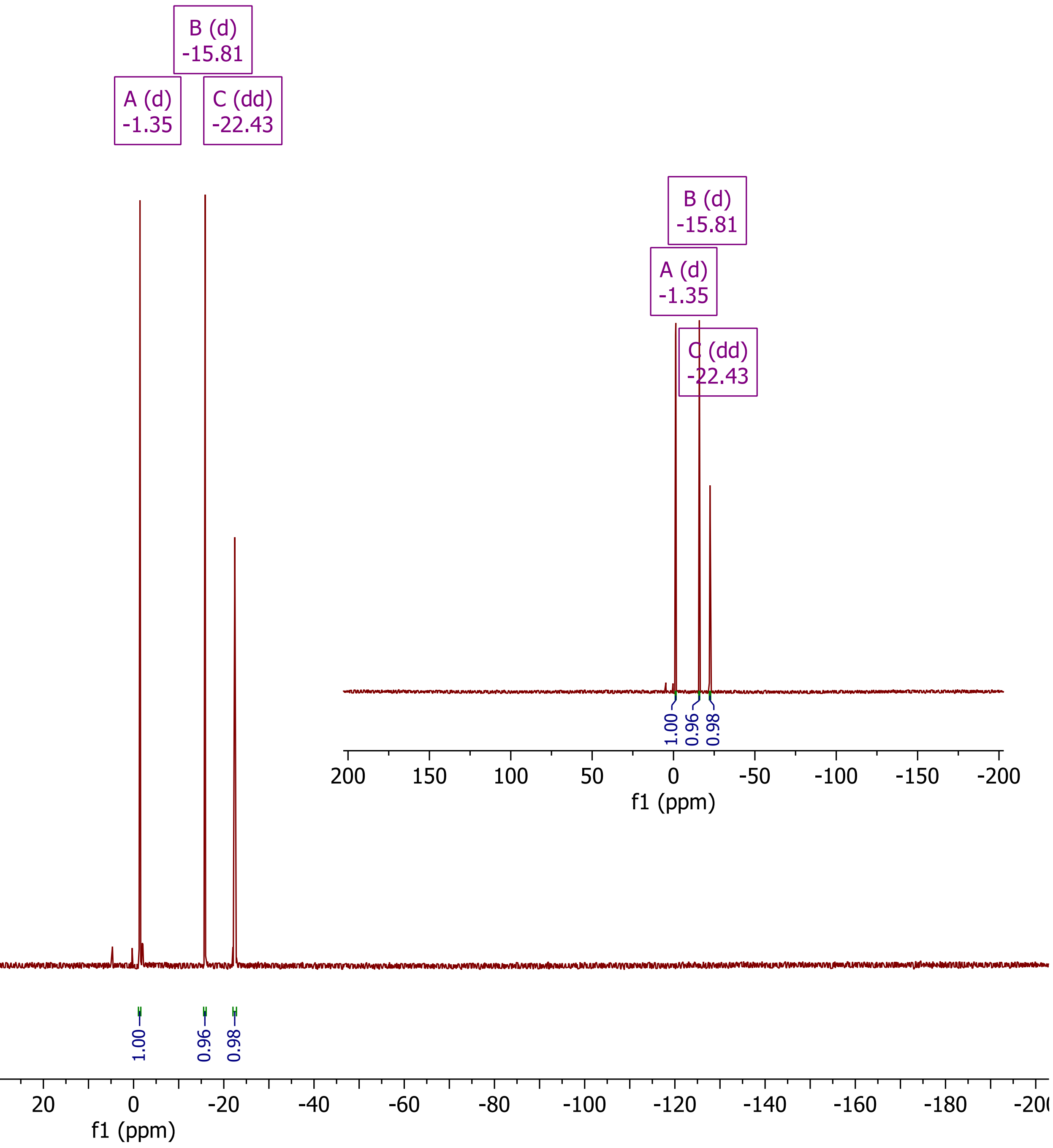

Compound 83, Napht-2-ylP$_3$-5'-deoxyguanosyl-5'-amidate, DQF-COSY (D2O)

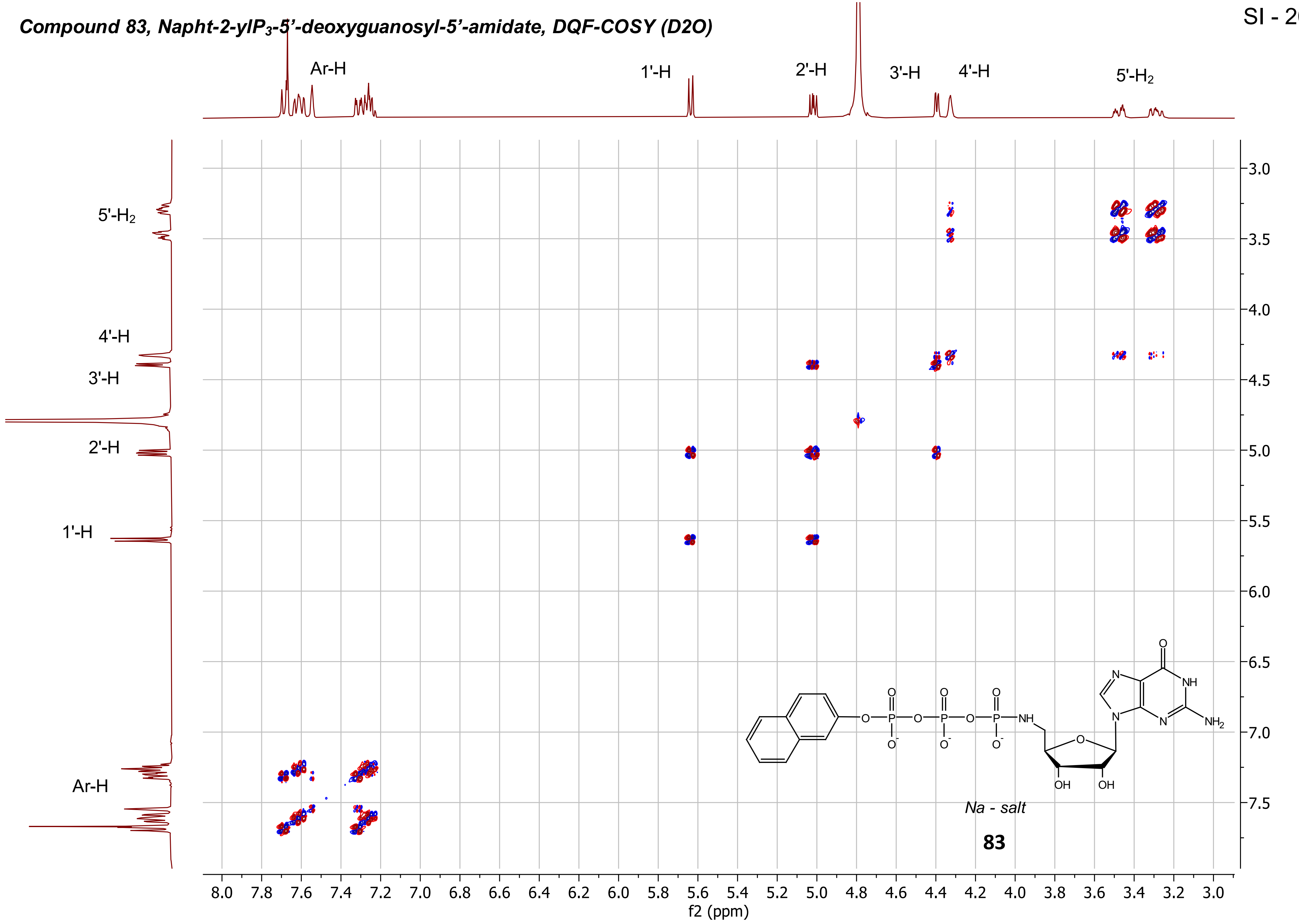

Compound 83, Napht-2-ylP$_3$-5'-deoxyguanosyl-5'-amidate, HSQC (D2O)

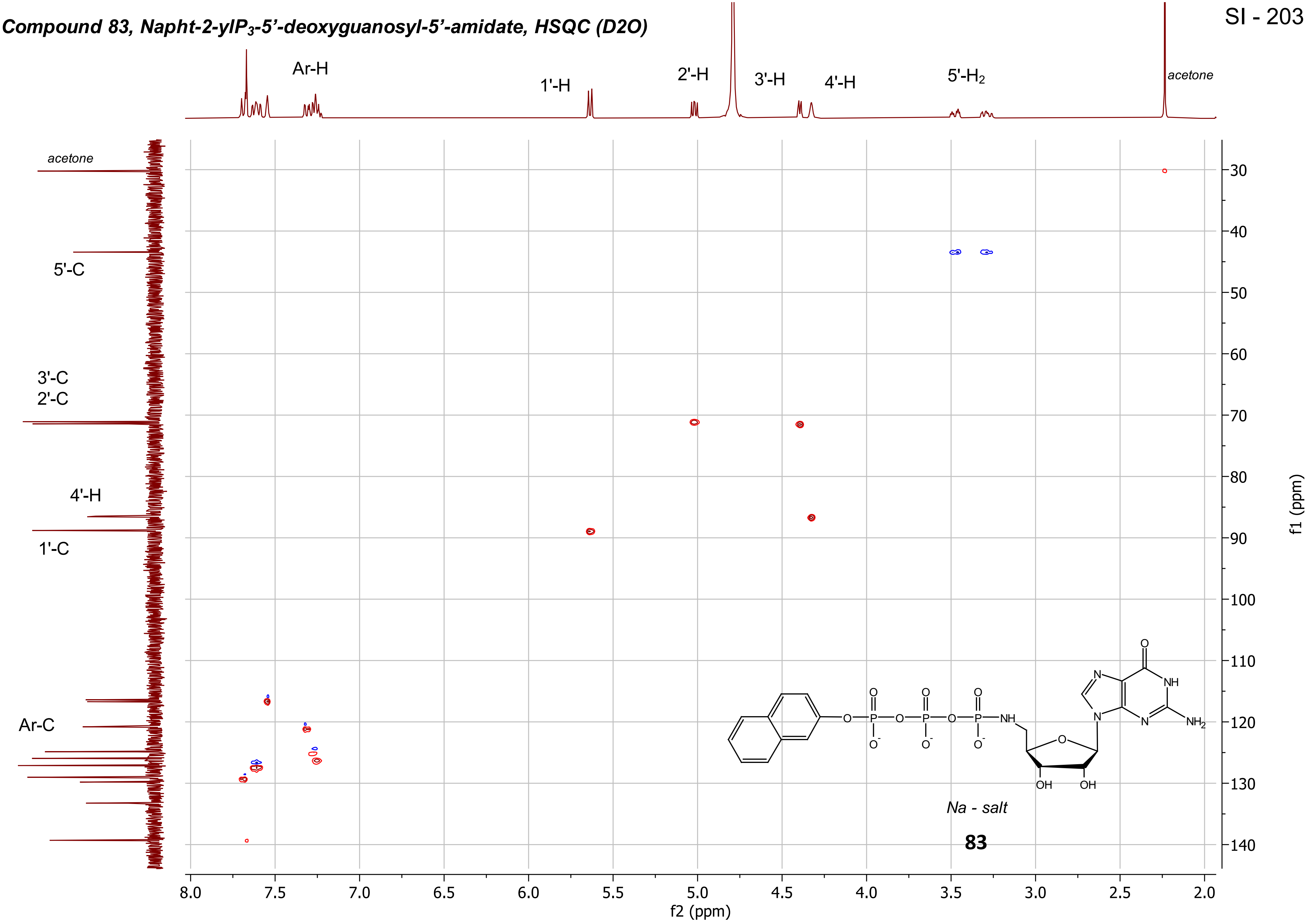

Compound 83, Napht-2-ylP$_3$-5'-deoxyguanosyl-5'-amidate, ^1H - ^{31}P - HMBC (D2O)

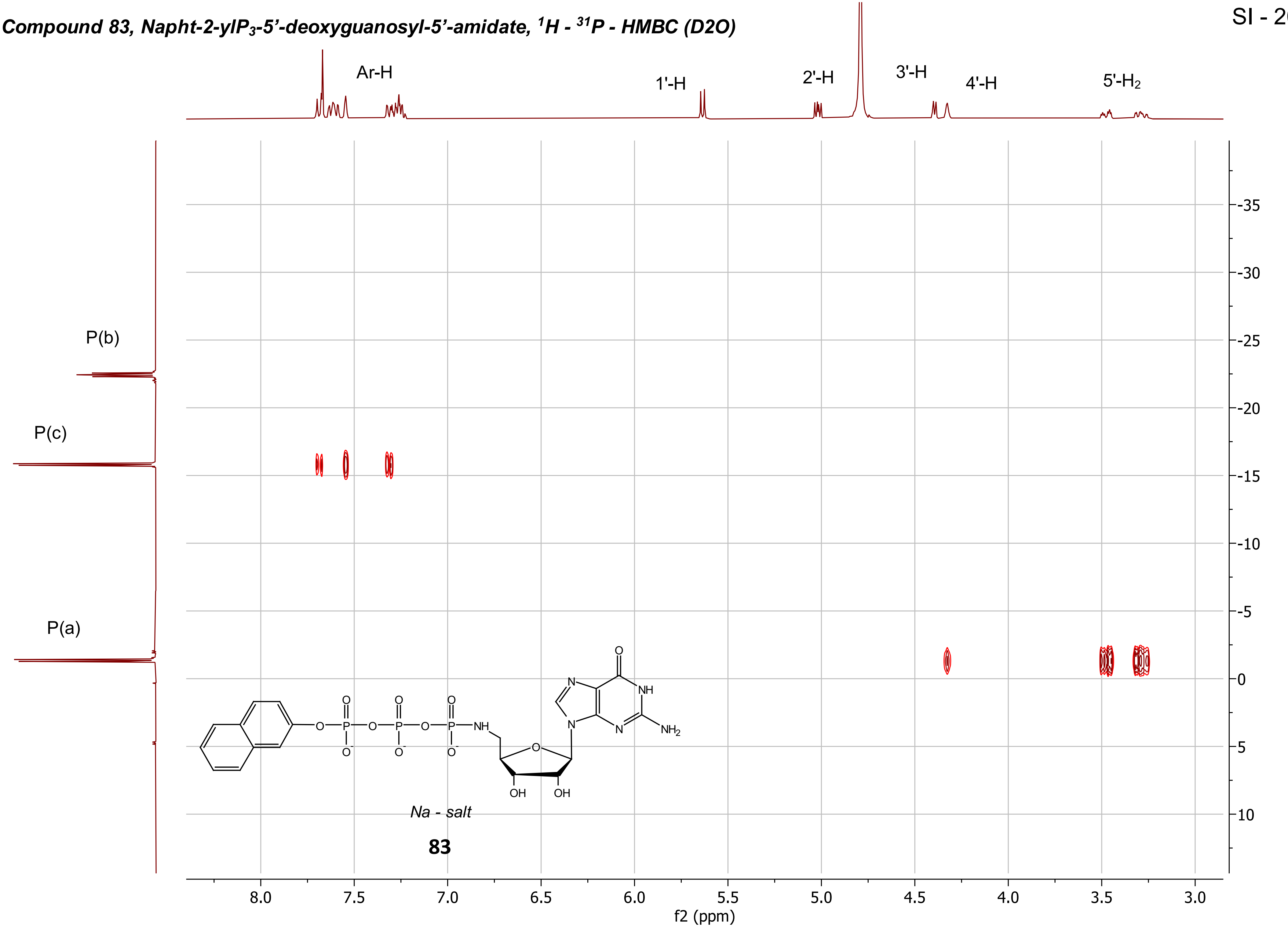

Compound 83, Napht-2-ylP$_3$-5'-deoxyguanosyl-5'-amidate, ^{13}C{^{1}H} - NMR (D2O, 101 MHz)

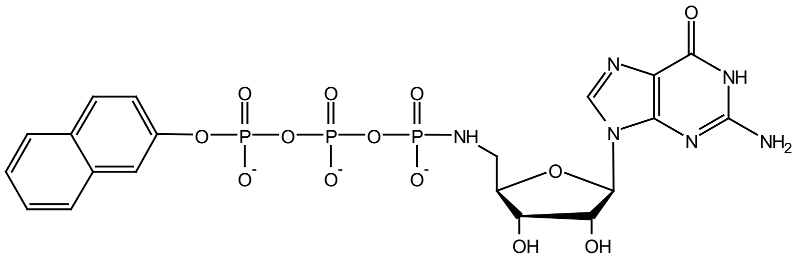

Na - salt

83

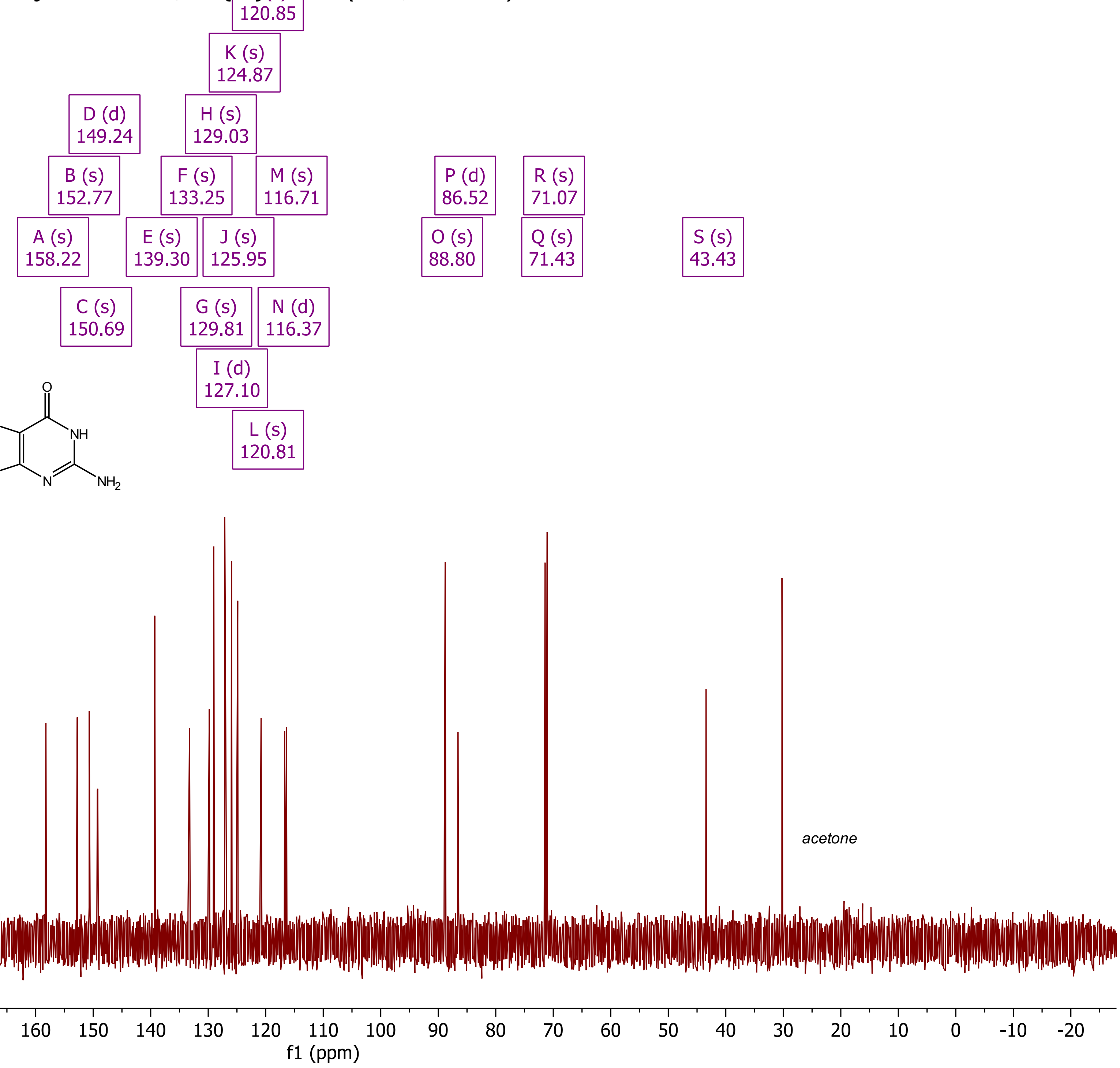

Compound 84, PyrenylP$_3$-5'-deoxyadenosyl-5'-amidate, ^{1}H - NMR (D2O, 400 MHz)

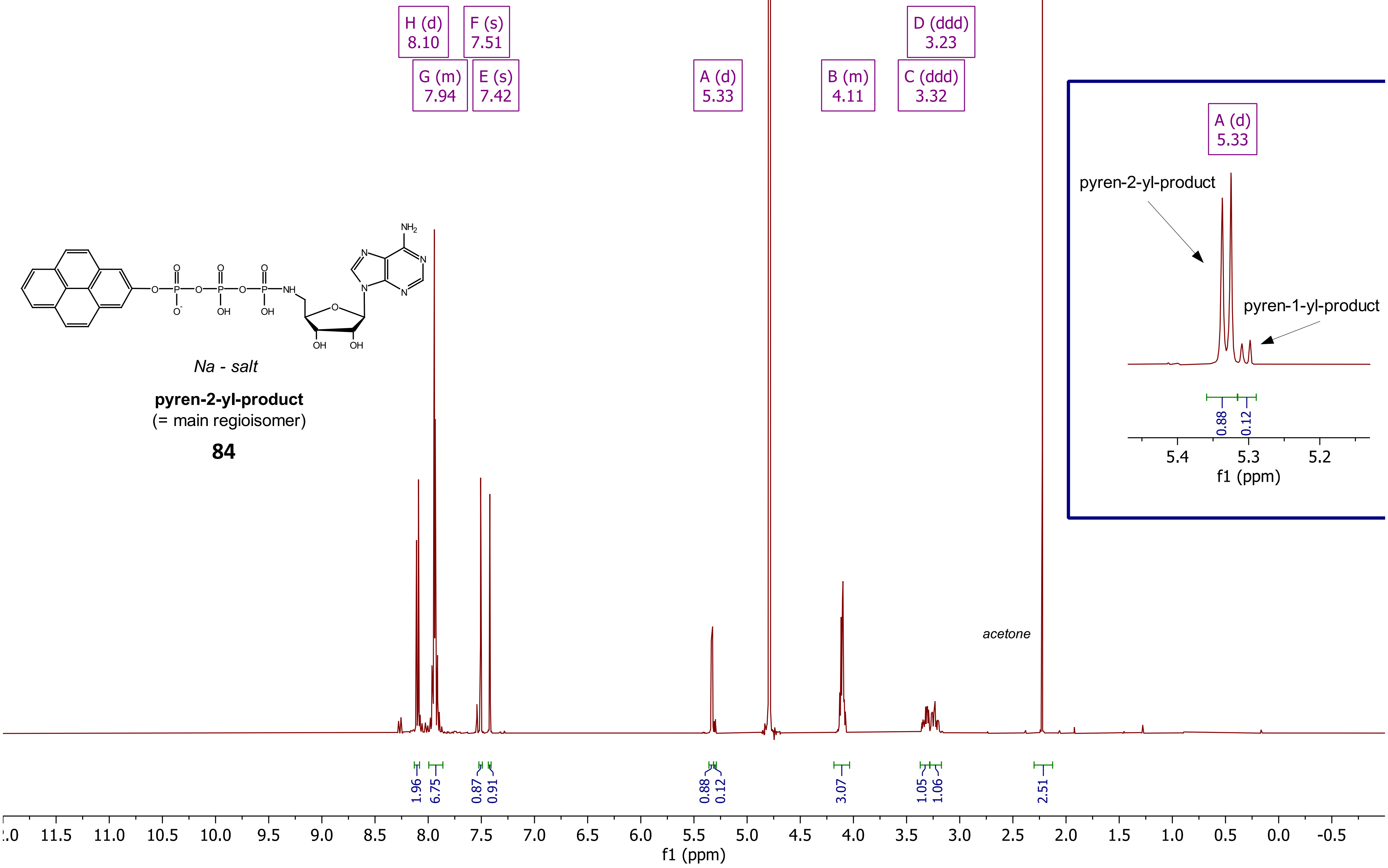

Compound 84, PyrenylP$_3$-5'-deoxyadenosyl-5'-amidate, ^{31}P {^{1}H} - NMR (D2O, 162 MHz)

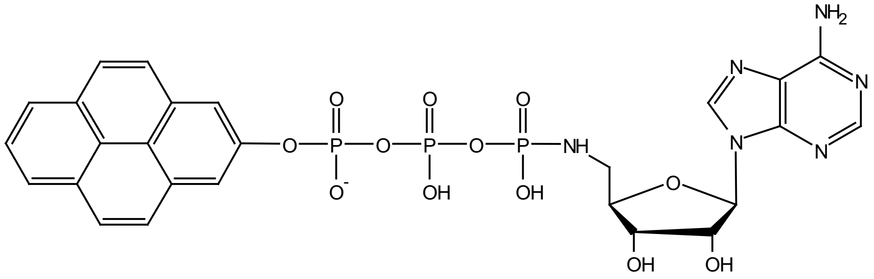

Na - salt

pyren-2-yl-product
(= main regioisomer)

84

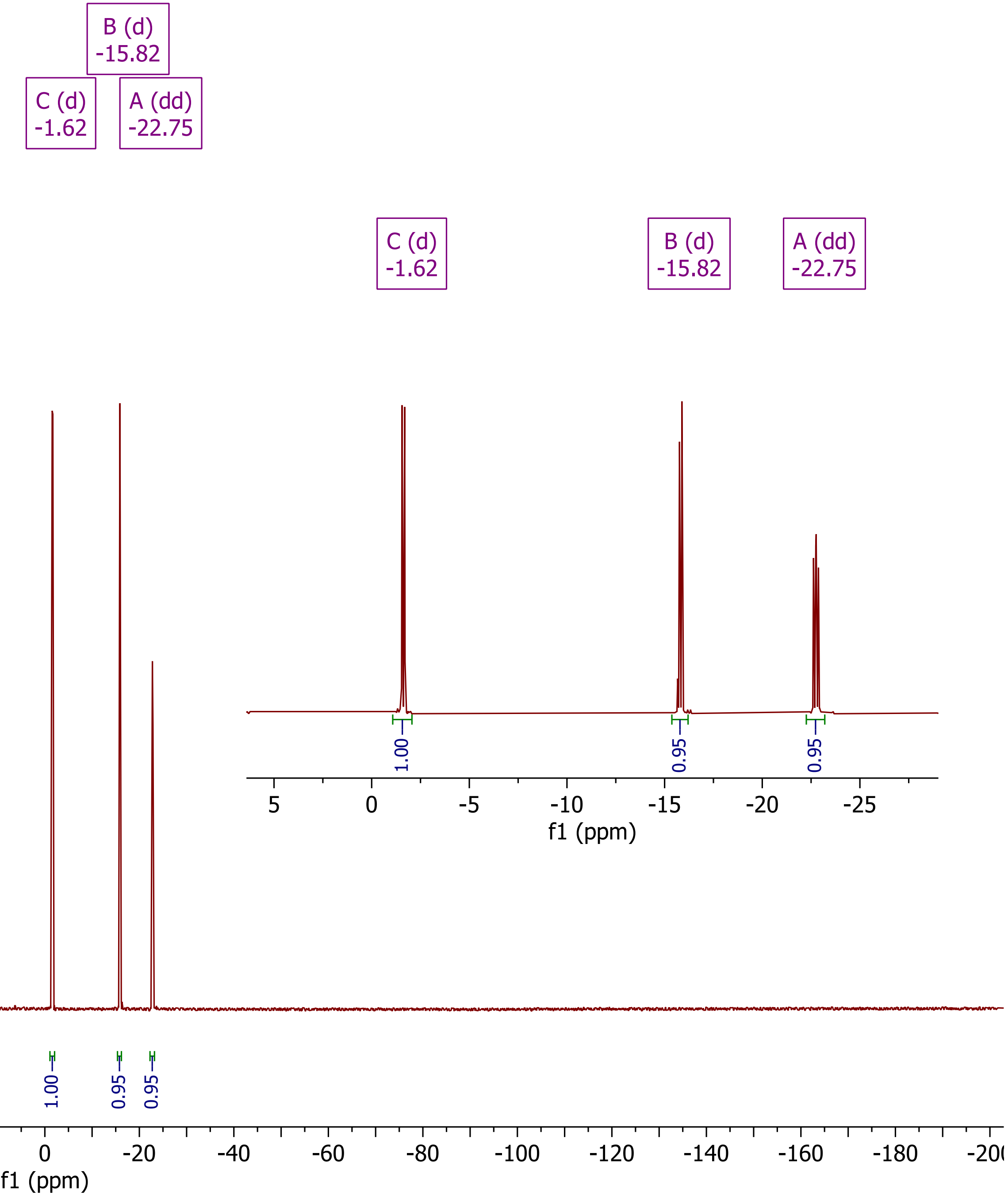

Compound 84, PyrenylP$_3$-5'-deoxyadenosyl-5'-amidate, DQF-COSY (D2O)

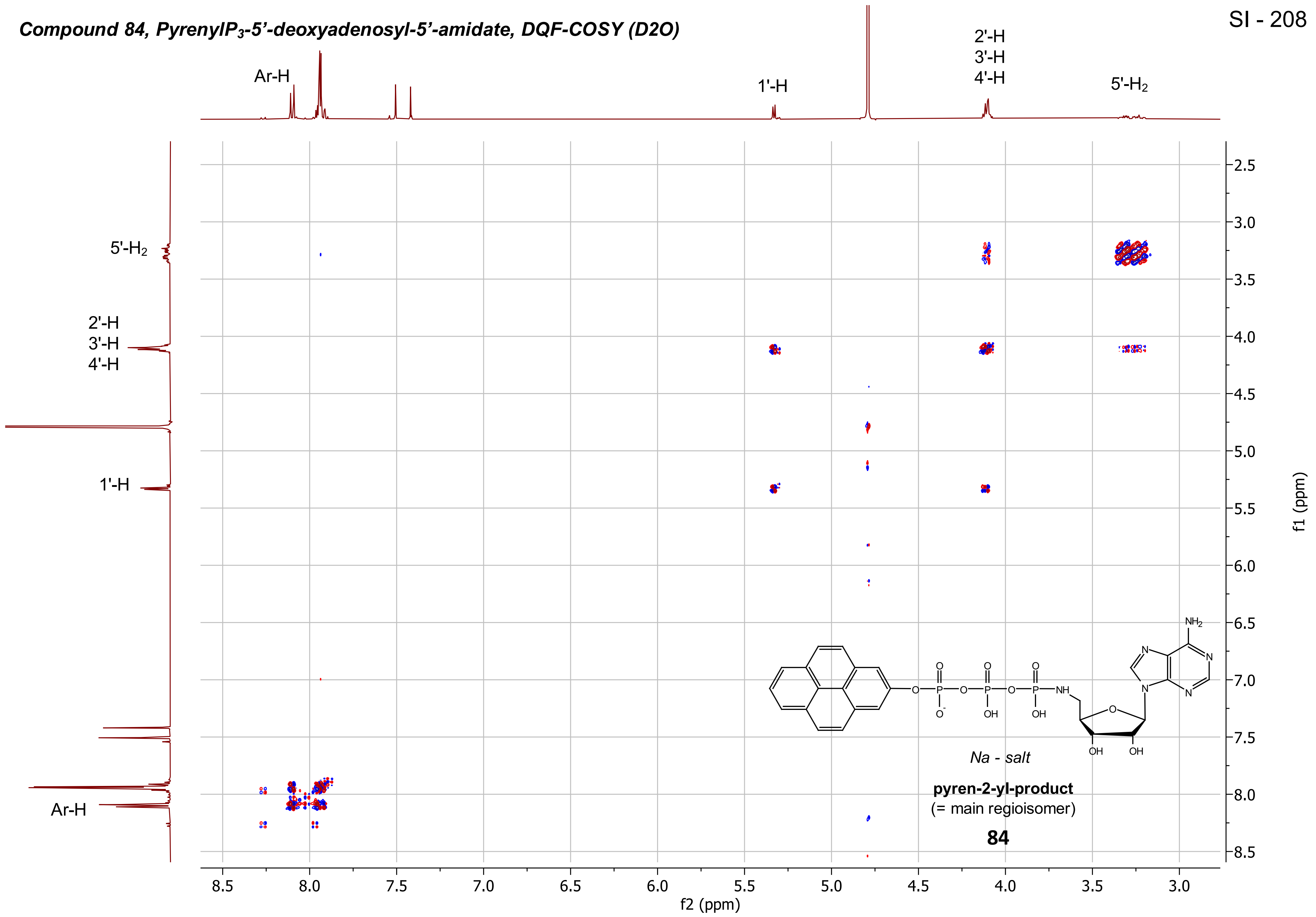

Compound 84, PyrenylP$_3$-5'-deoxyadenosyl-5'-amidate, HSQC (D2O)

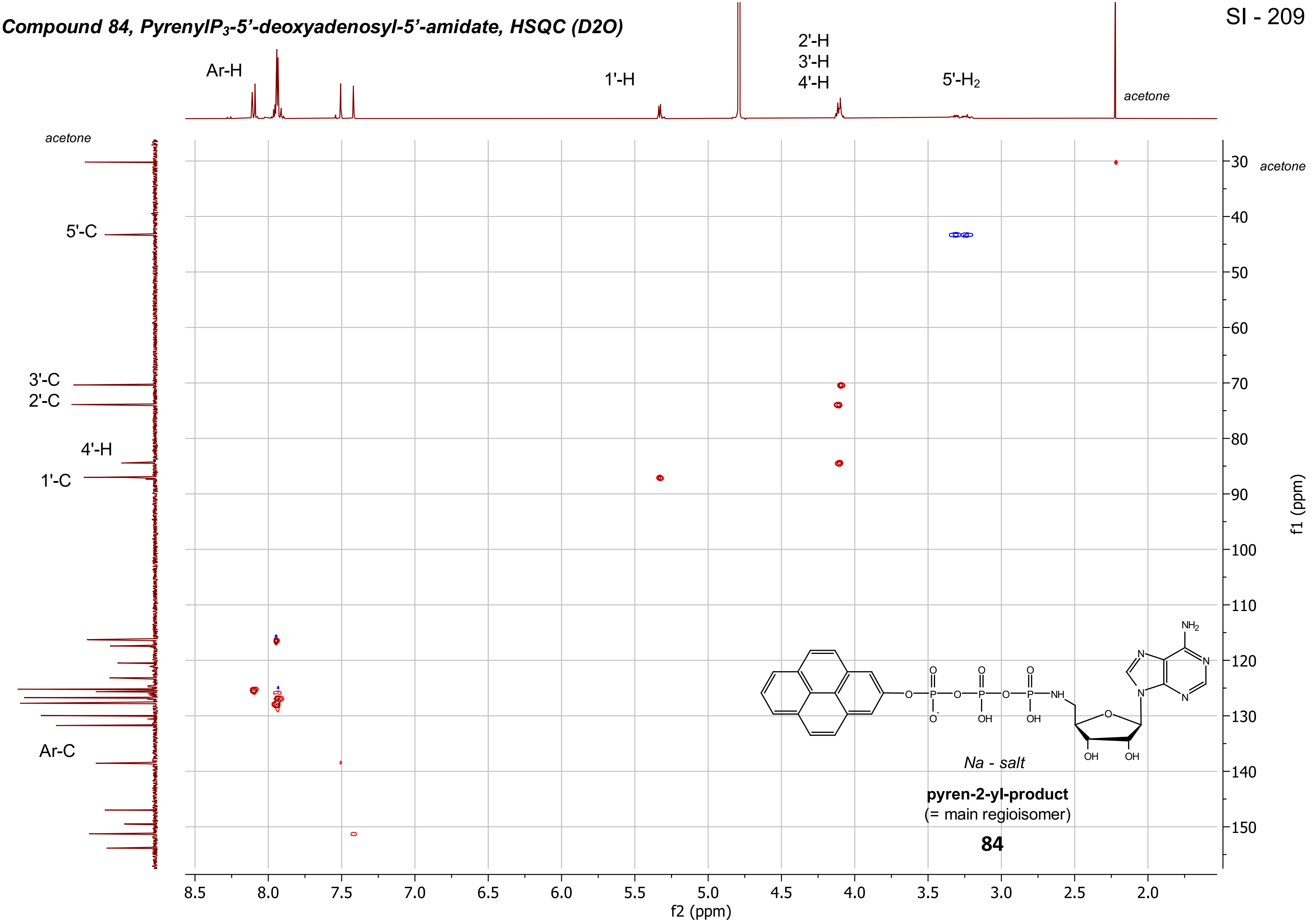

Compound 84, PyrenylP$_3$-5'-deoxyadenosyl-5'-amidate, ^{1}H - ^{31}P - HMBC (D2O)

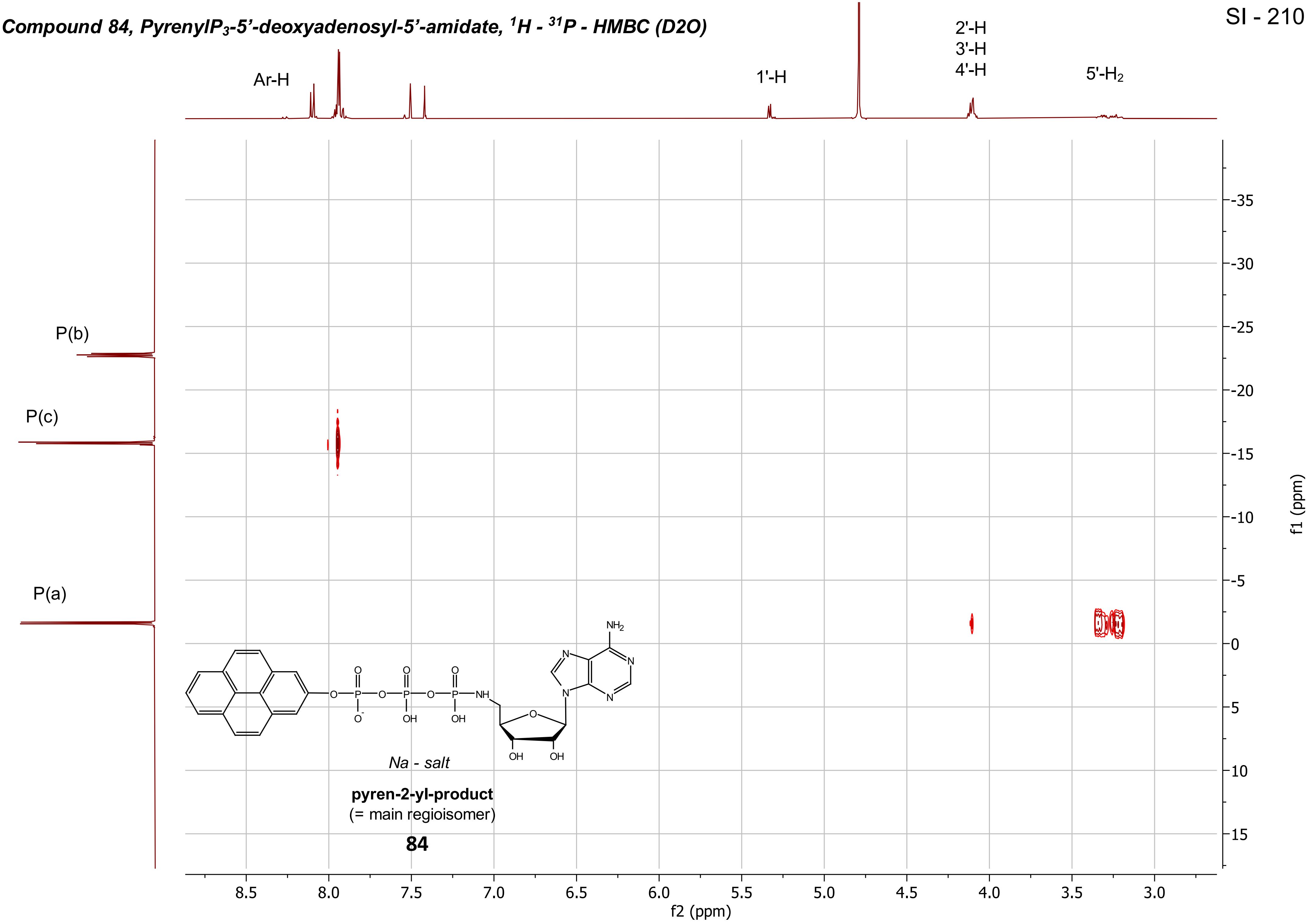

Compound 84, PyrenylP$_3$-5'-deoxyadenosyl-5'-amidate, $^{13}C\{^1H\}$ - NMR (D2O, 101 MHz)

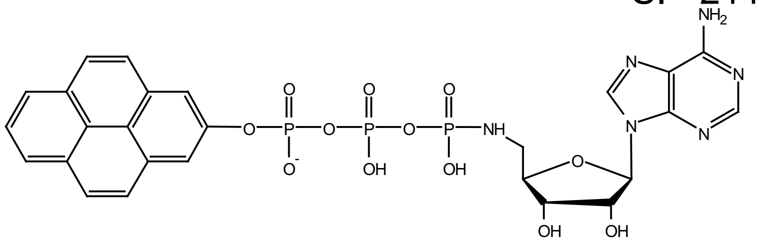

Na - salt

pyren-2-yl-product
(= main regioisomer)

84

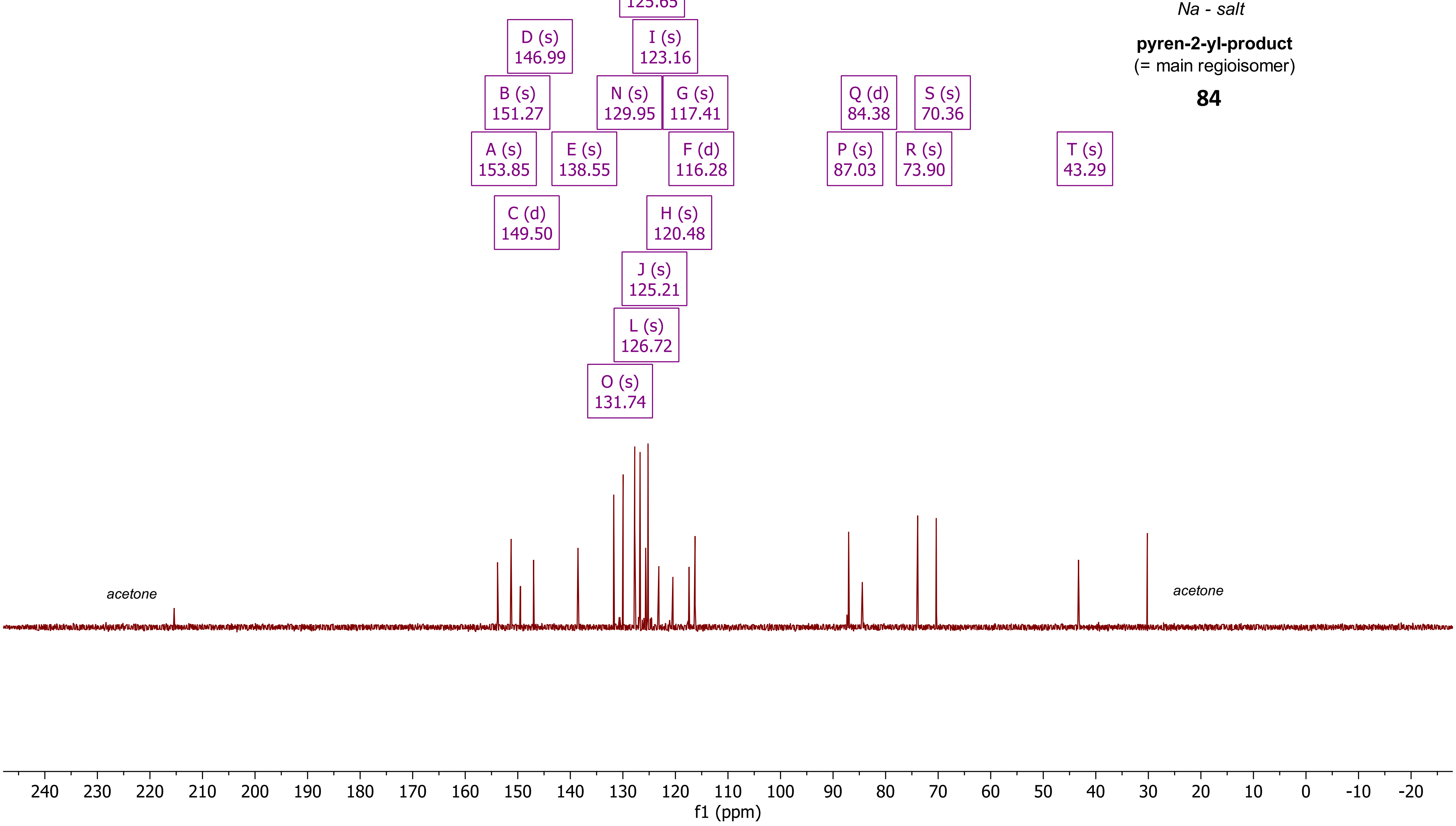

Compound 85, Benzo[1,3]dioxol-5-ylP$_4$-((7-(diethylamino)-2-oxo-2H-chromen-4-yl)methylamidate , ^{1}H - NMR (D2O, 400 MHz)

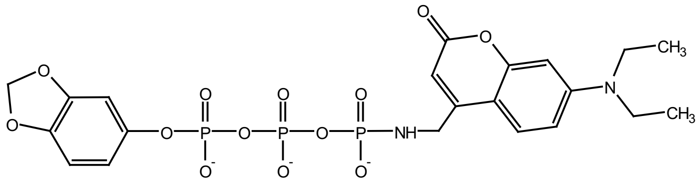

Na -salt

85

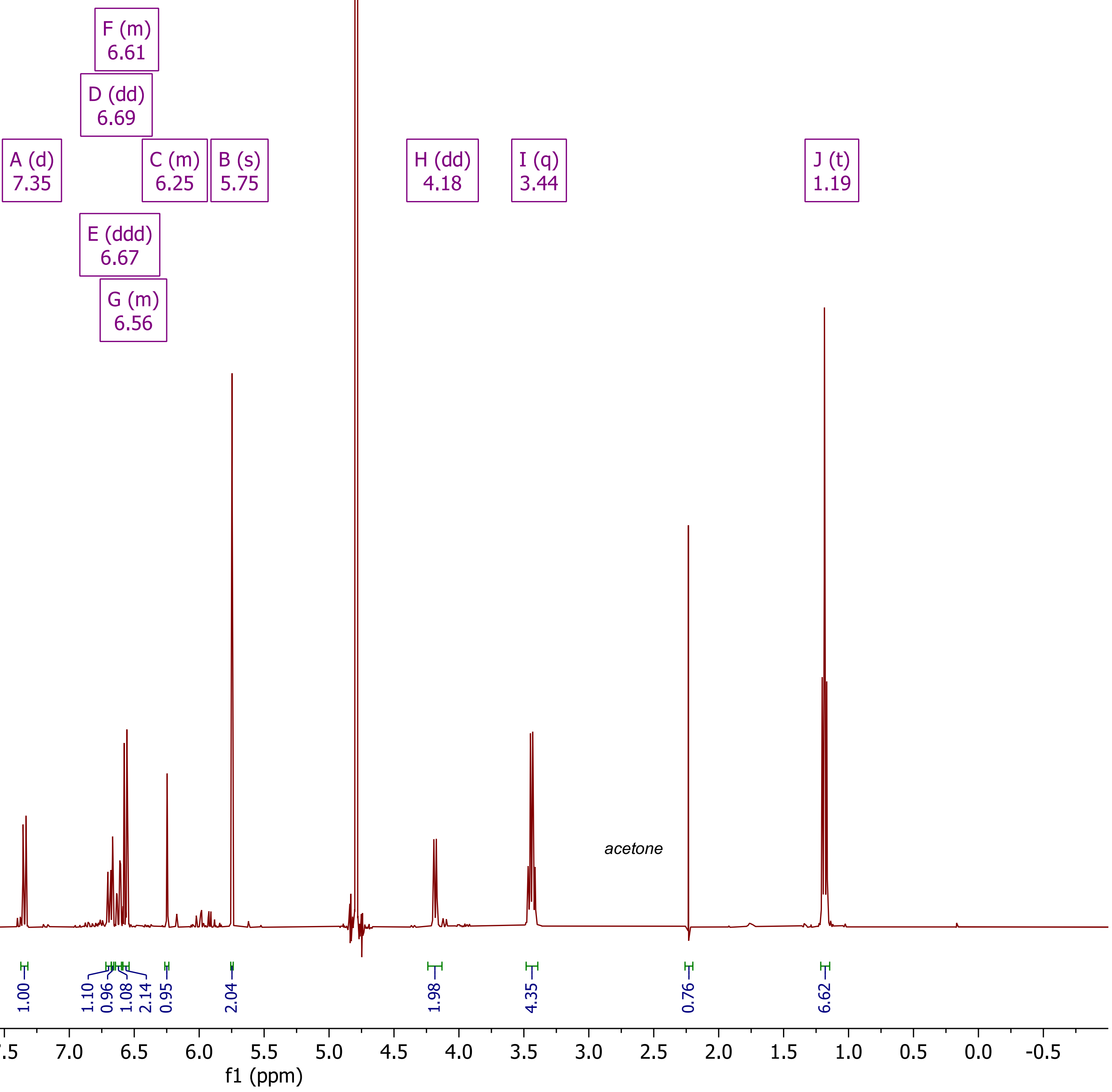

Compound 85, Benzo[1,3]dioxol-5-ylP$_4$-((7-(diethylamino)-2-oxo-2H-chromen-4-yl)methylamidate, ^{31}P {^1H} - NMR (D2O, 162 MHz)

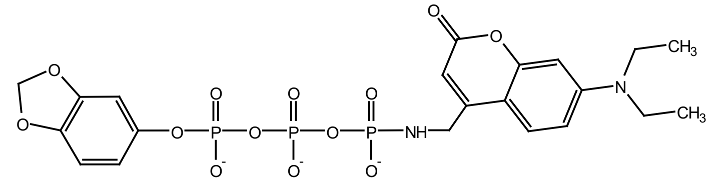

Na -salt

85

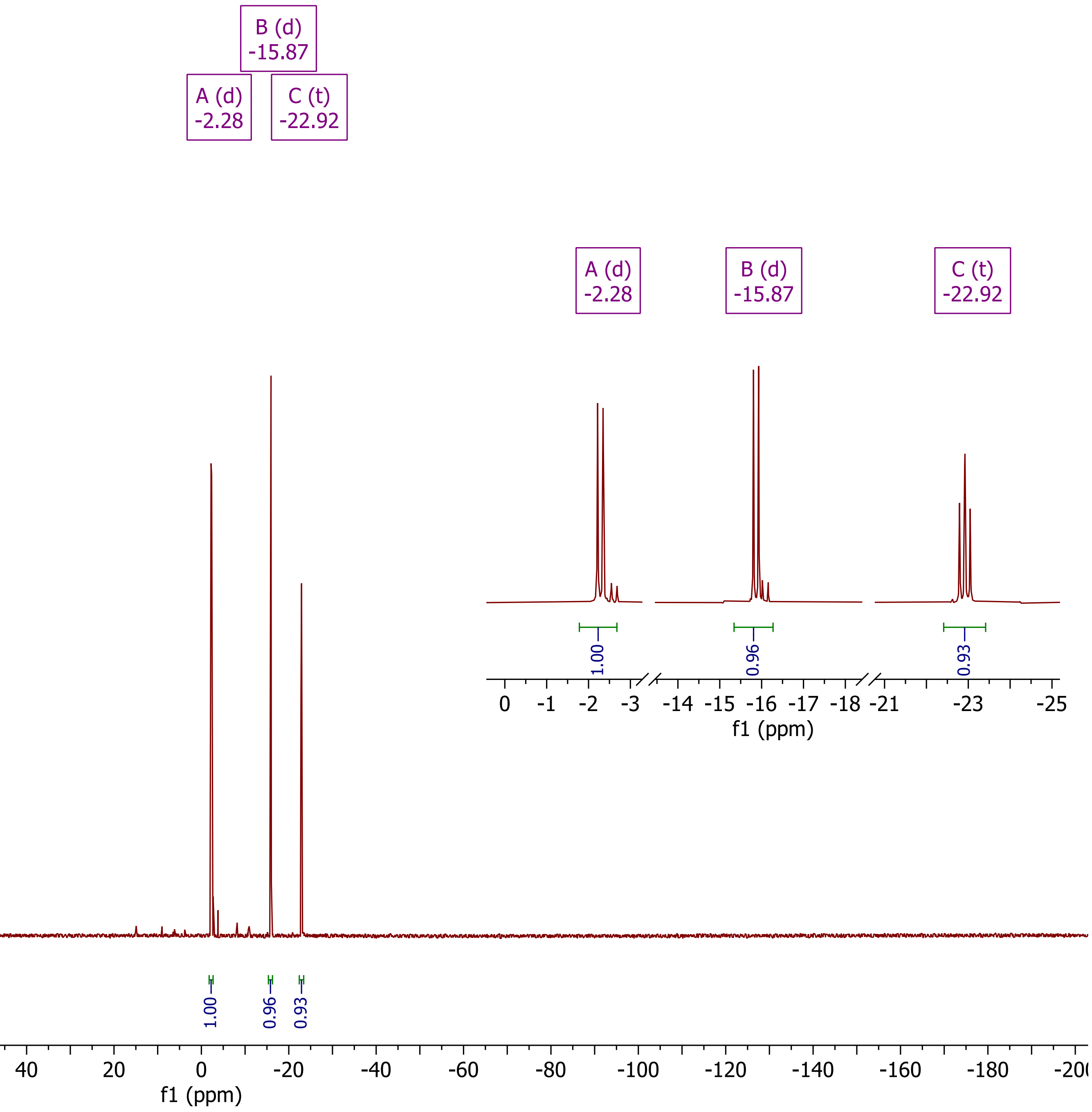

Compound 85, Benzo[1,3]dioxol-5-ylP$_4$-((7-(diethylamino)-2-oxo-2H-chromen-4-yl)methylamidate, DQF-COSY (D2O)

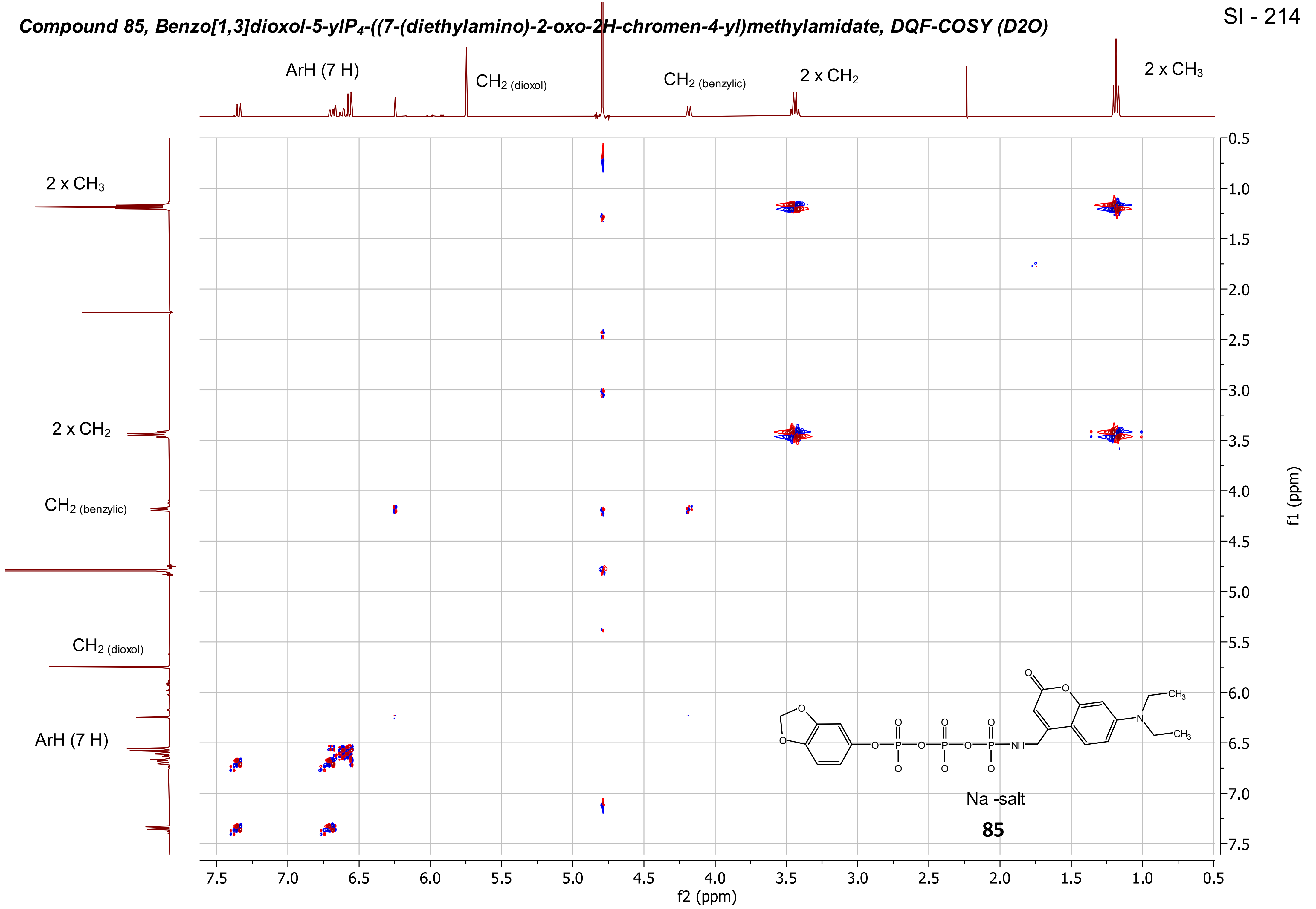

Compound 85, Benzo[1,3]dioxol-5-ylP4-((7-(diethylamino)-2-oxo-2H-chromen-4-yl)methylamidate, HSQC (D2O)

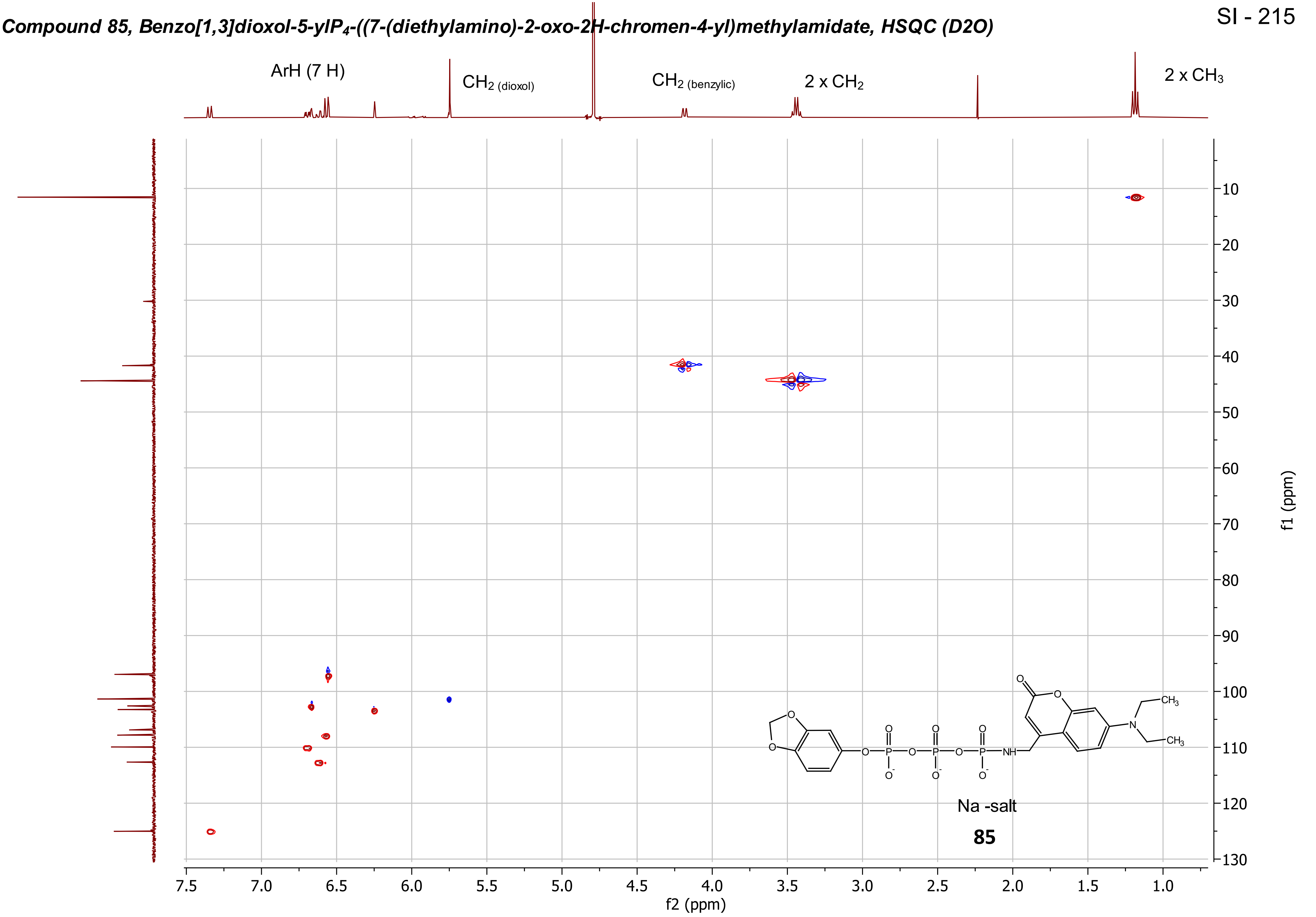

Compound 85, Benzo[1,3]dioxol-5-ylP$_4$-((7-(diethylamino)-2-oxo-2H-chromen-4-yl)methylamidate, ^{1}H - ^{31}P - HMBC (D2O)

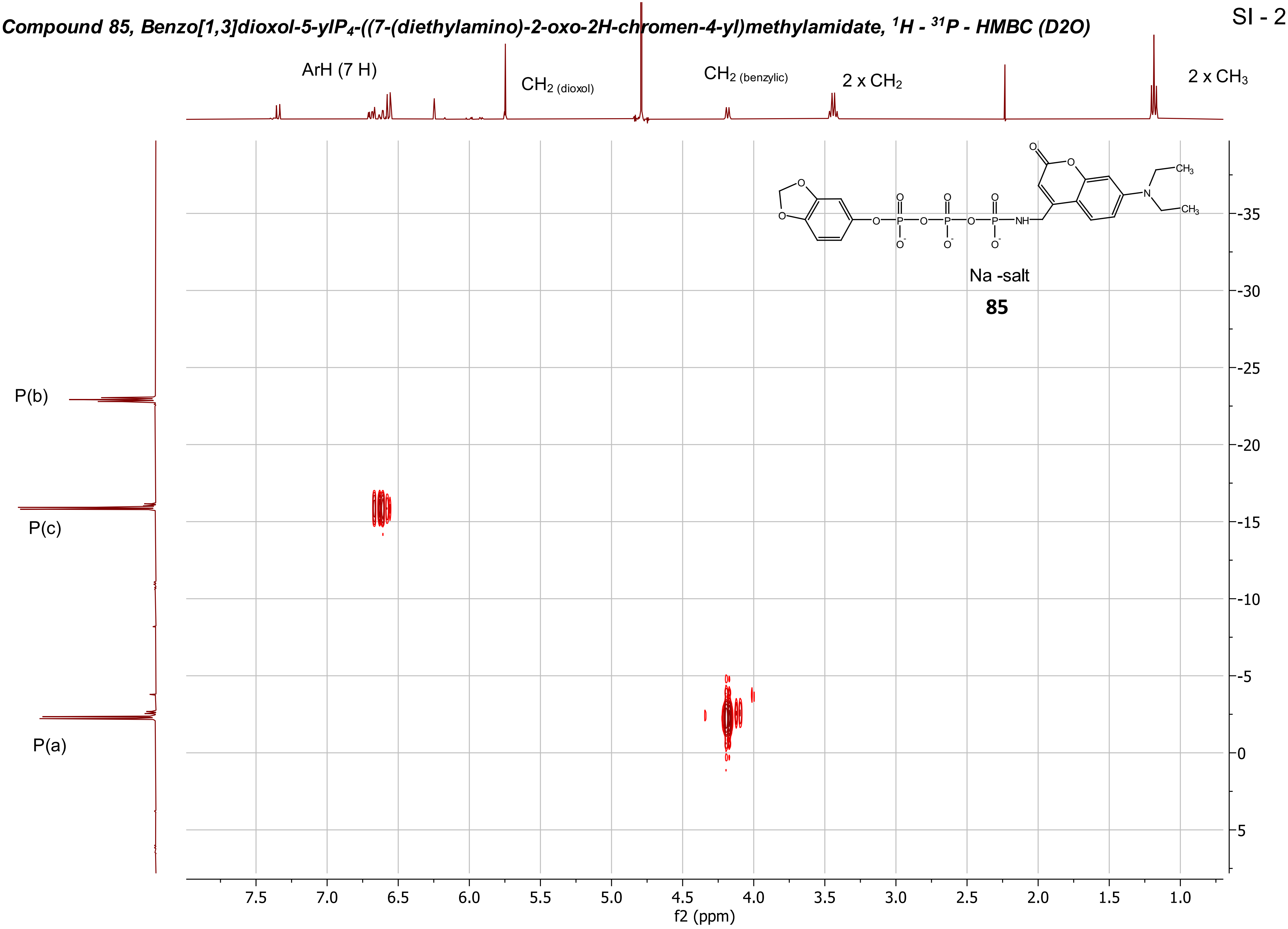

Compound 85, Benzo[1,3]dioxol-5-ylP$_4$-((7-(diethylamino)-2-oxo-2H-chromen-4-yl)methylamidate, $^{13}C\{^1H\}$ - NMR (D2O, 101 MHz)

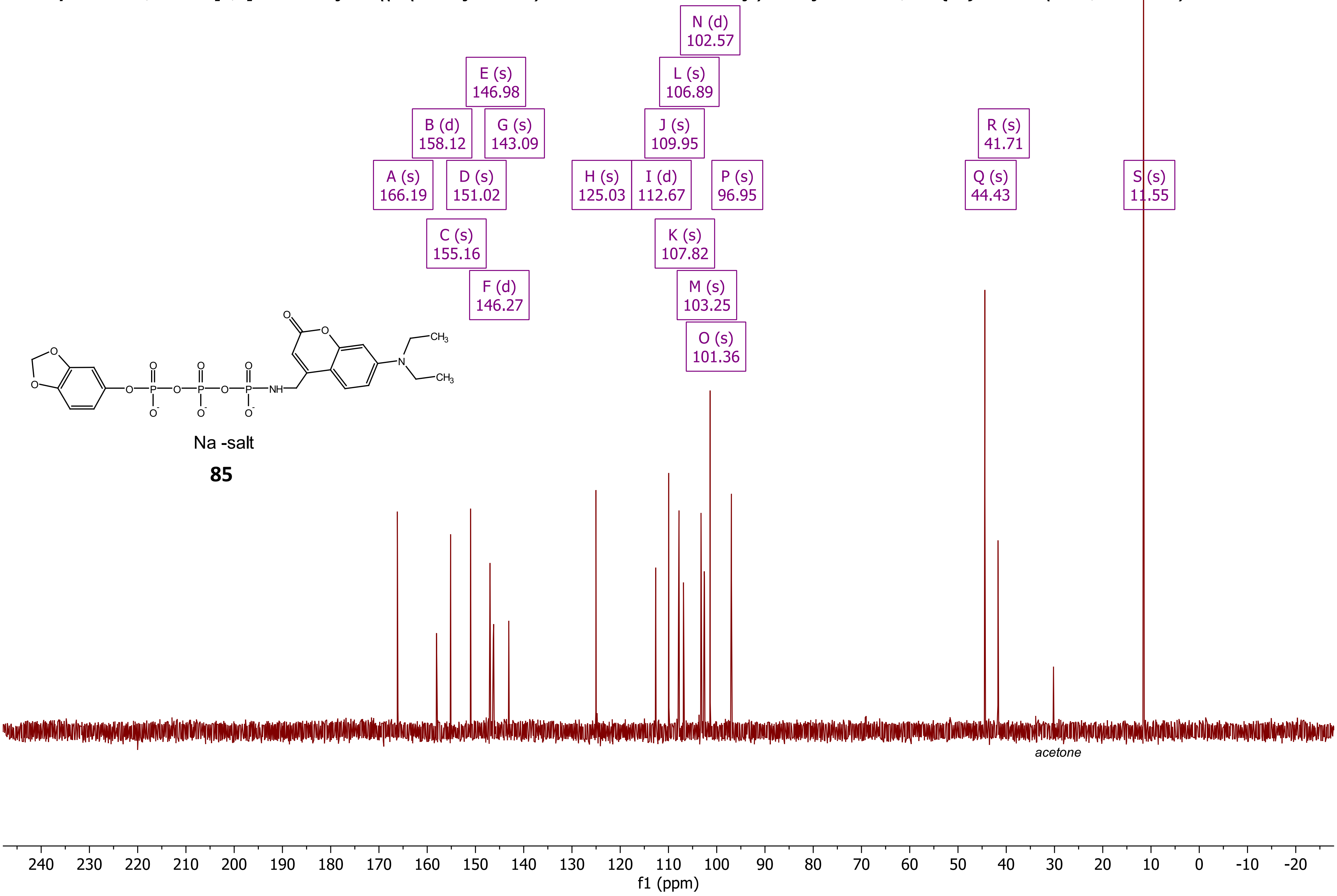

Compound 86, PhenylP$_4$-propargylamidate, ^{1}H - NMR (D2O, 400 MHz)

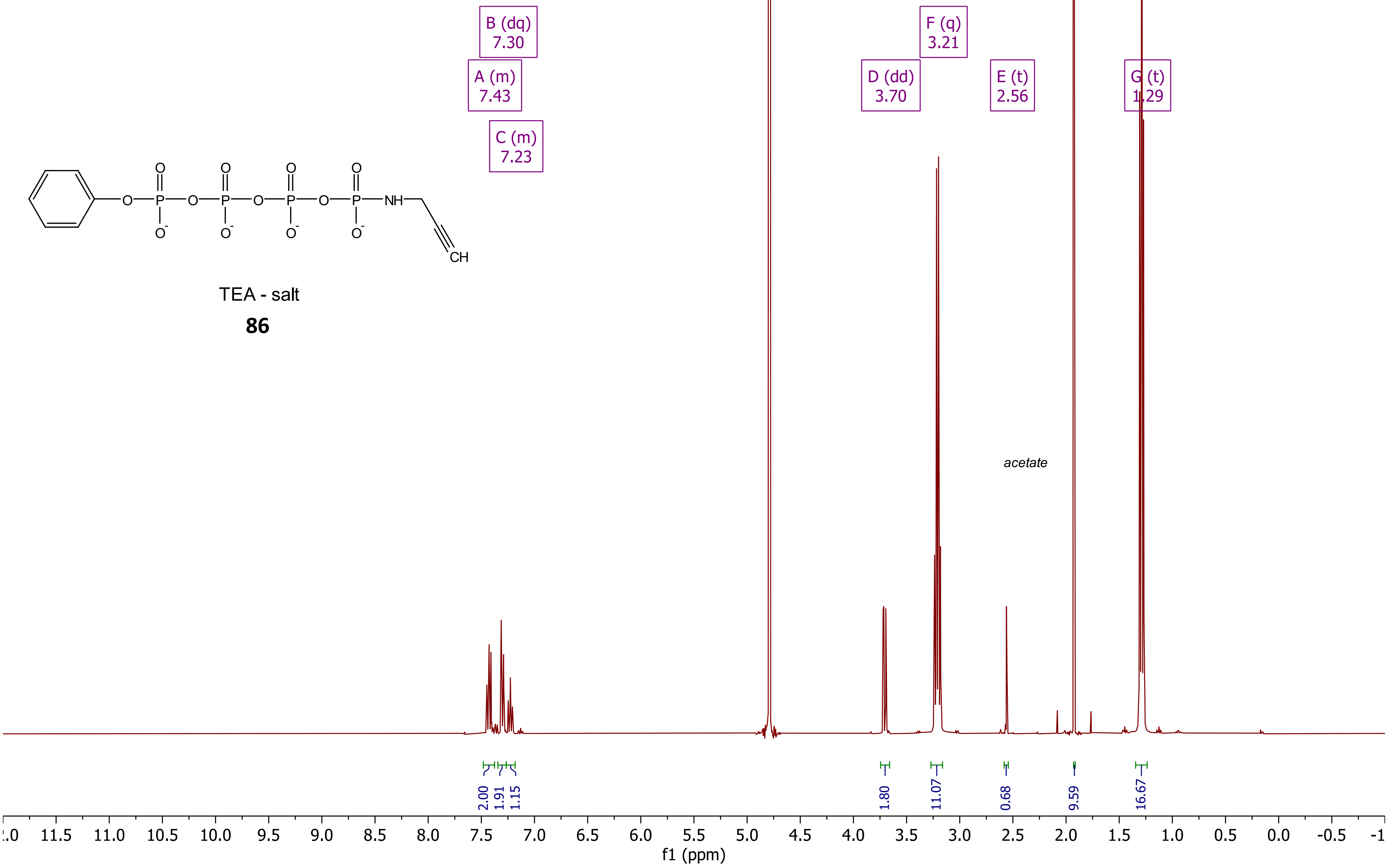

Compound 86, PhenylP$_4$-propargylamidate, ^{31}P {^{1}H} - NMR (D2O, 162 MHz)

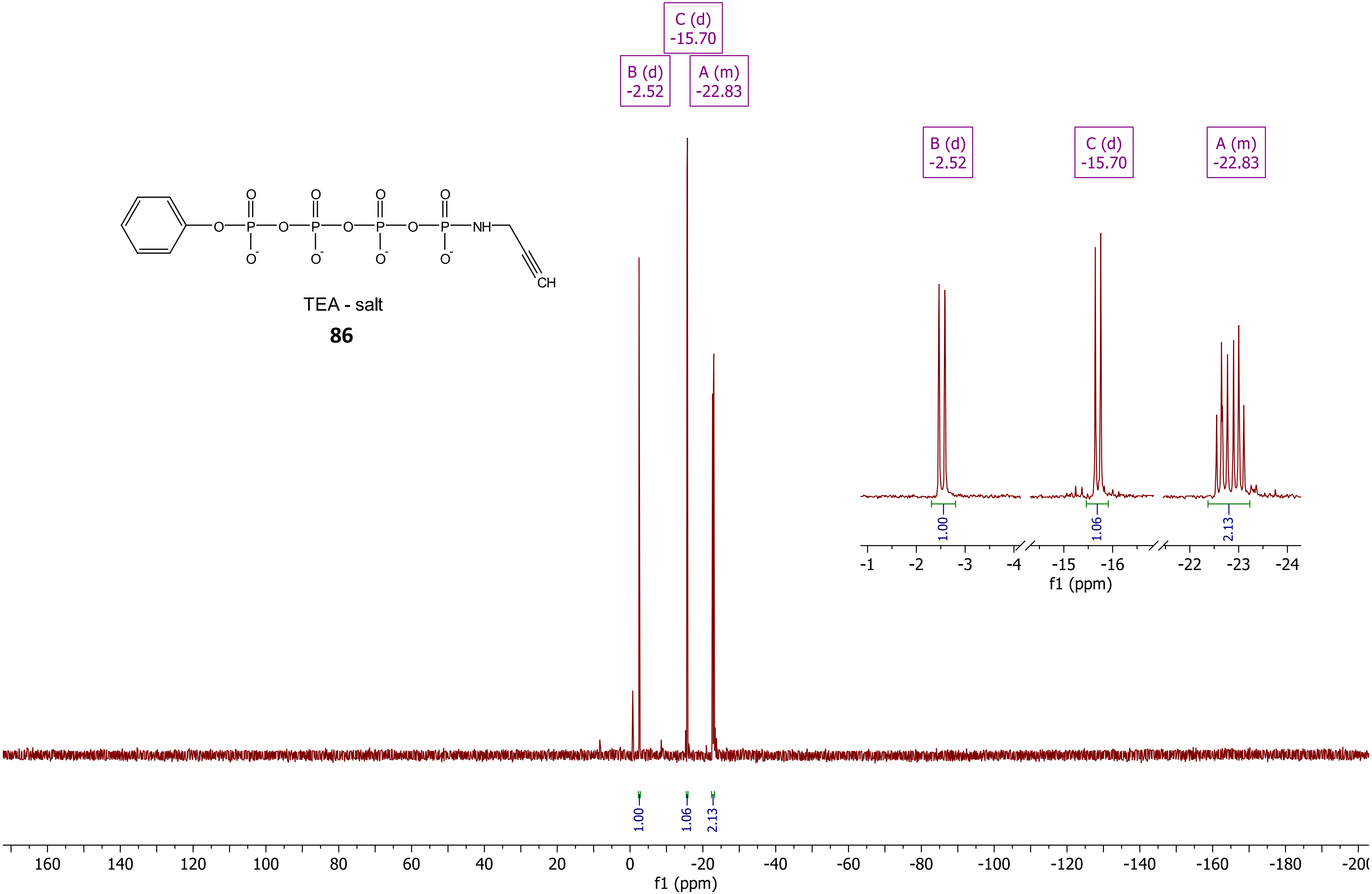

Compound 86, PhenylP$_4$-propargylamidate, ^1H - ^{31}P - HMBC (D2O)

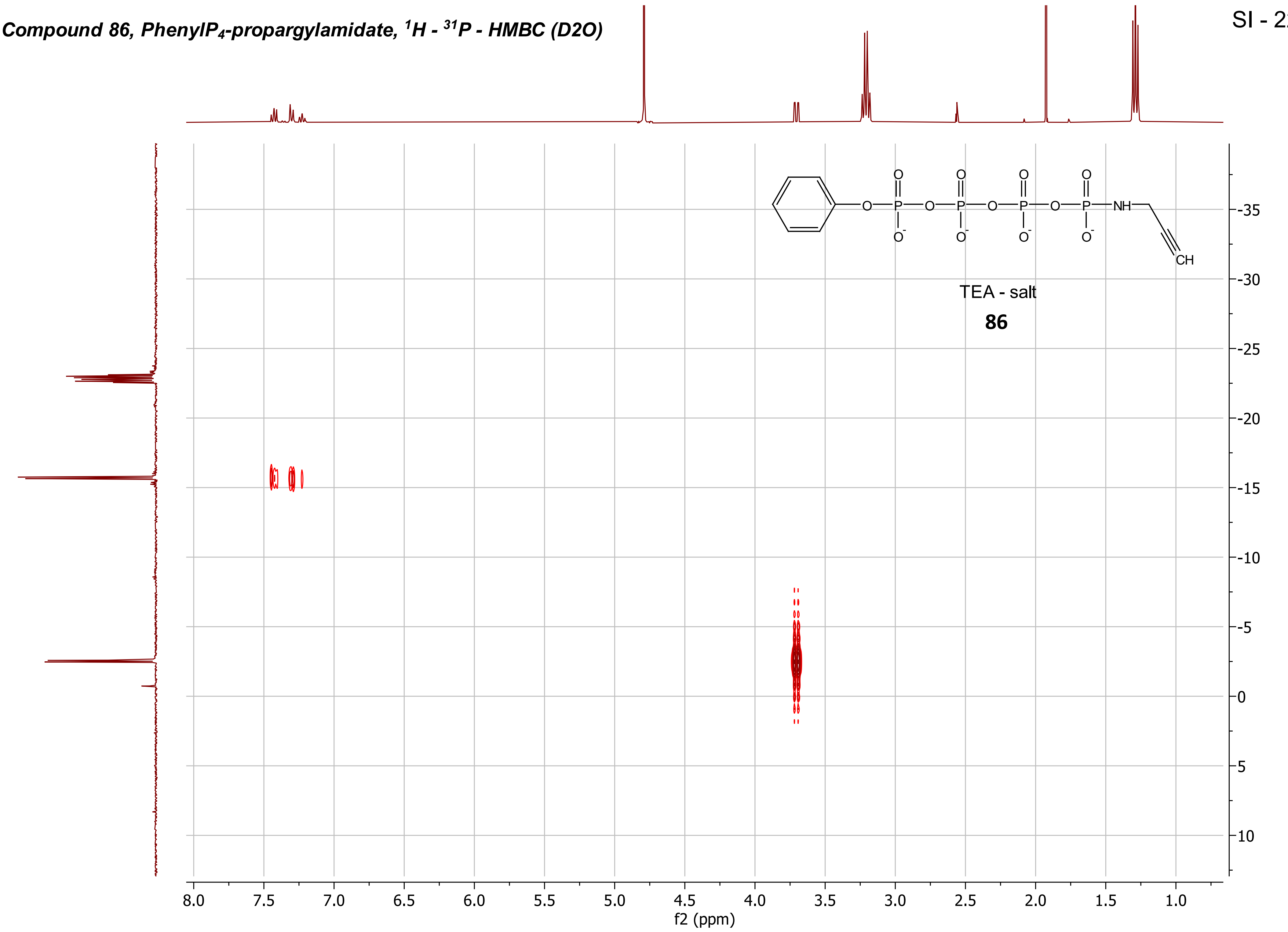

Compound 86, PhenylP$_4$-propargylamidate, $^{13}C\{^1H\}$ - NMR (D2O, 101 MHz)

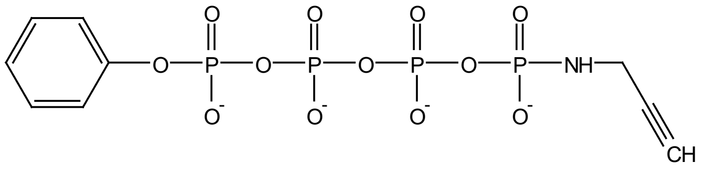

TEA - salt

86

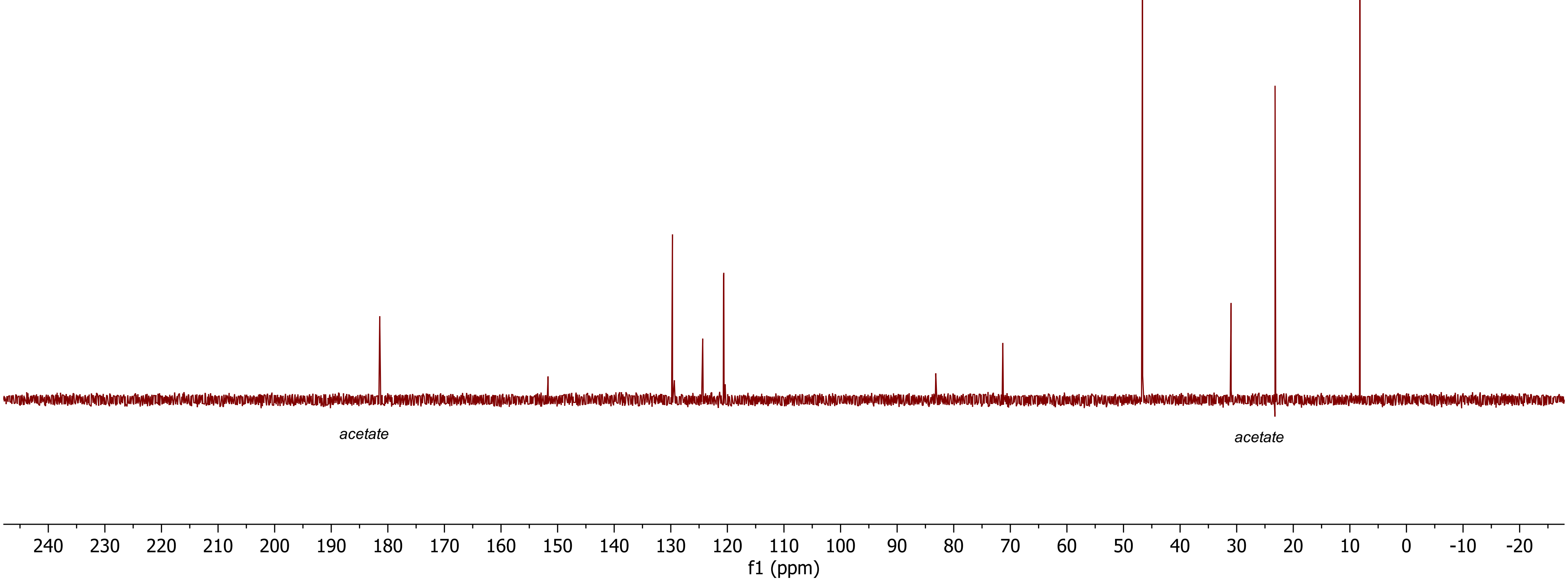

Compound 87, PyrenylP$_4$-5'-deoxyguanosyl-5'-amidate, ^{1}H - NMR (D2O, 400 MHz)

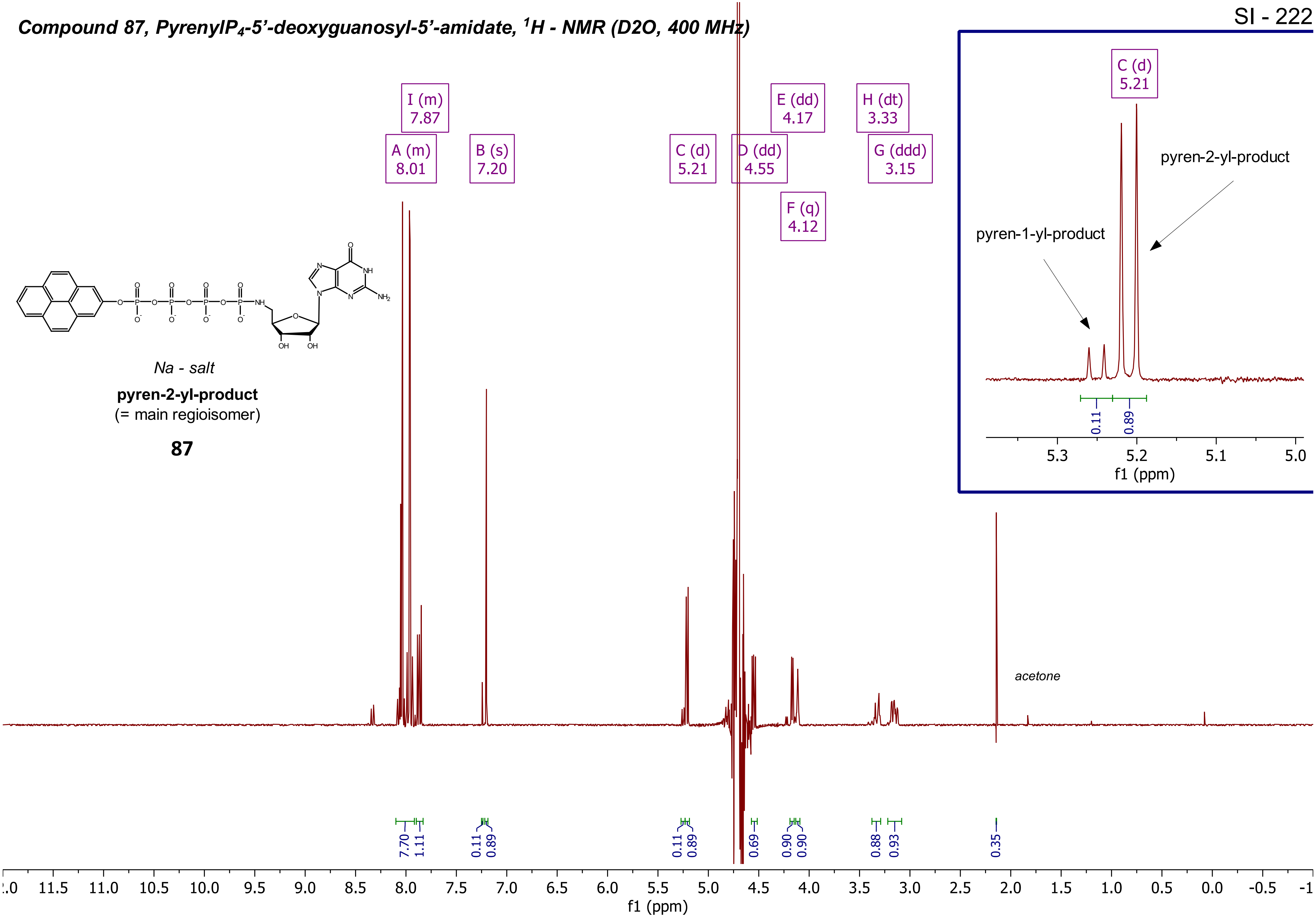

Compound 87, PyrenylP$_4$-5'-deoxyguanosyl-5'-amidate, $^{31}P\{^1H\}$ - NMR (D2O, 162 MHz)

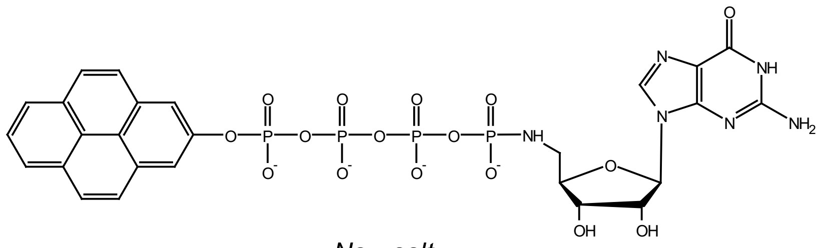

Na - salt

pyren-2-yl-product
(= main regioisomer)

87

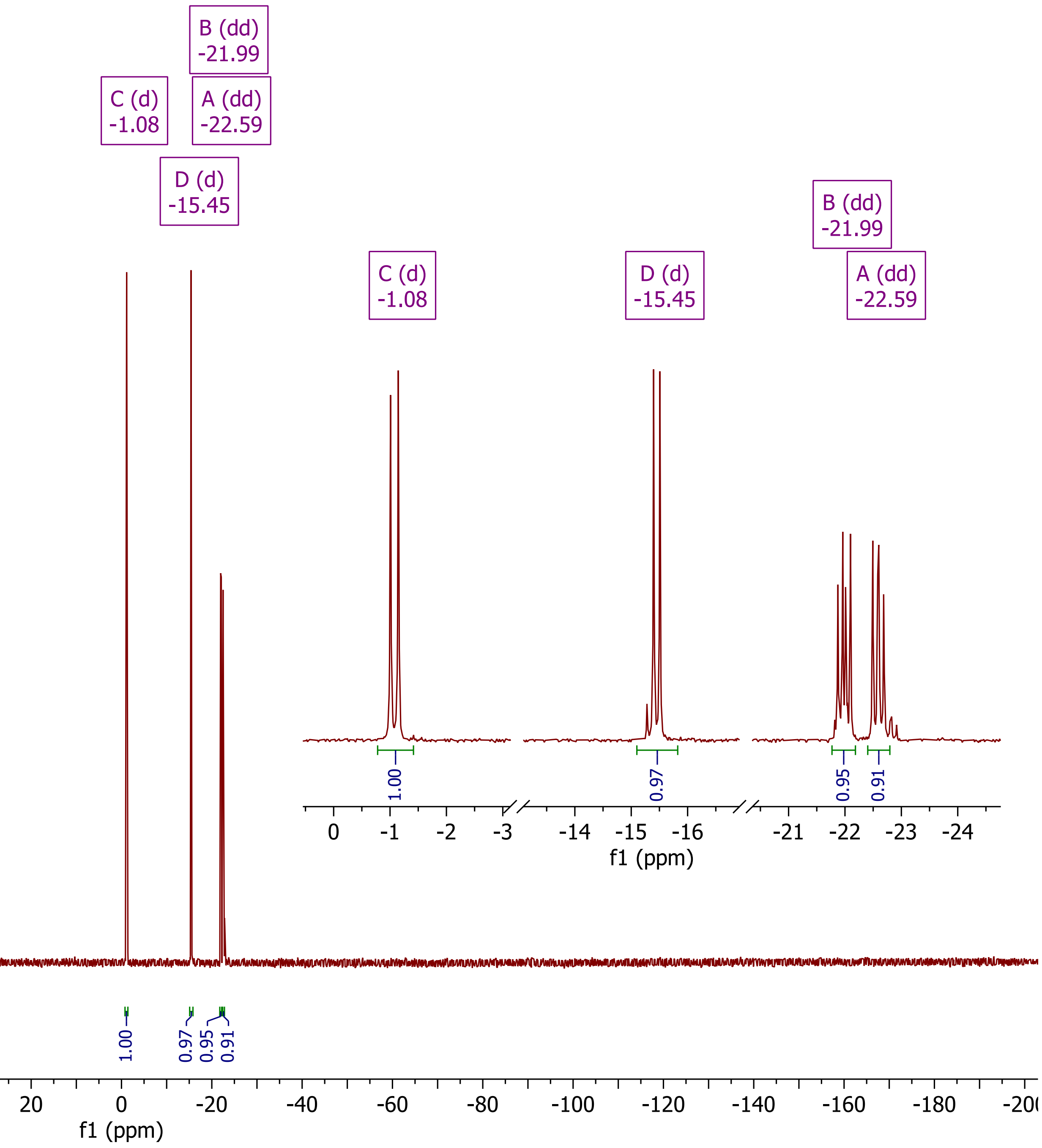

Compound 87, PyrenylP$_4$-5'-deoxyguanosyl-5'-amidate, DQF-COSY (D2O)

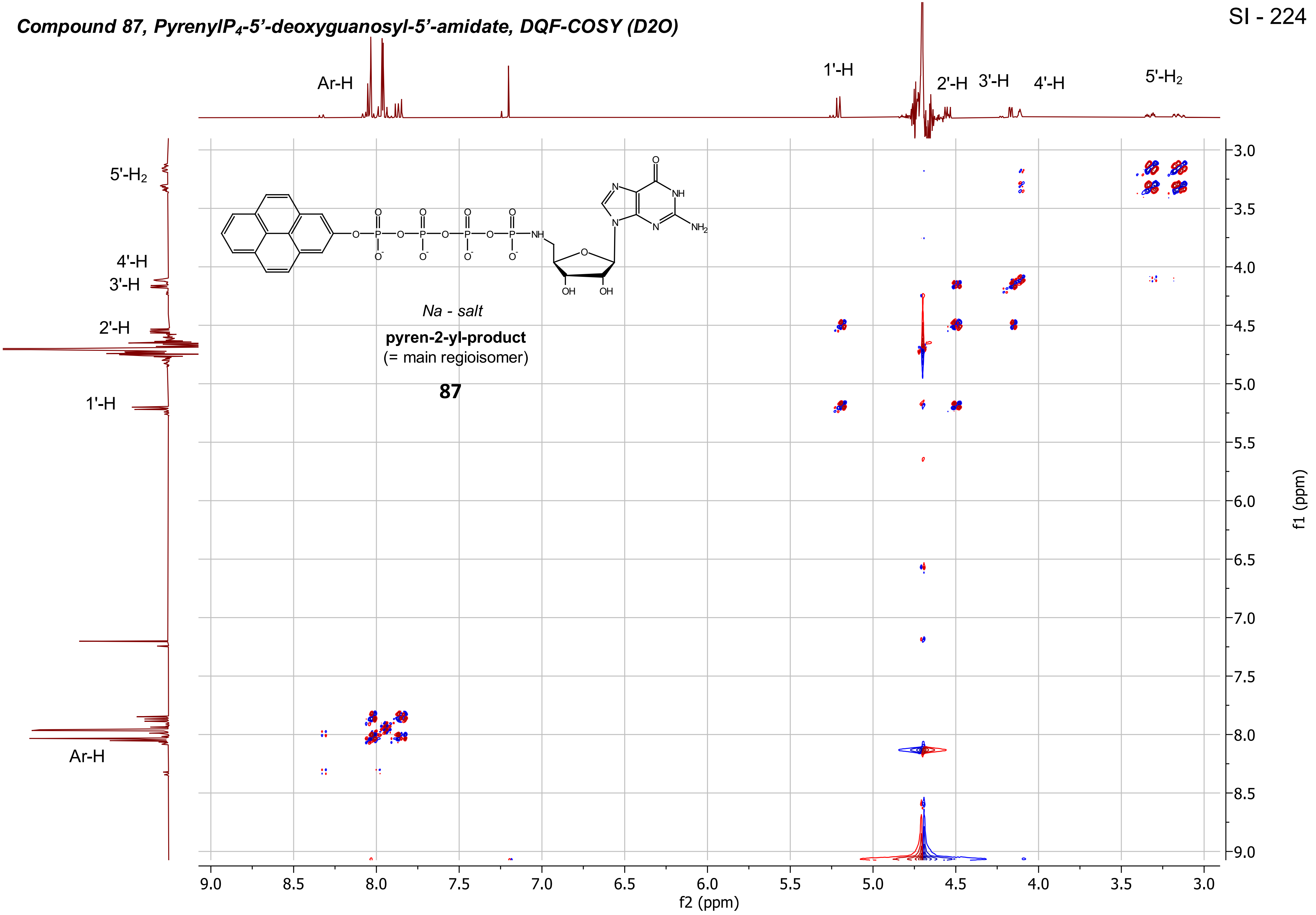

Compound 87, PyrenylP$_4$-5’-deoxyguanosyl-5’-amidate, HSQC (D2O)

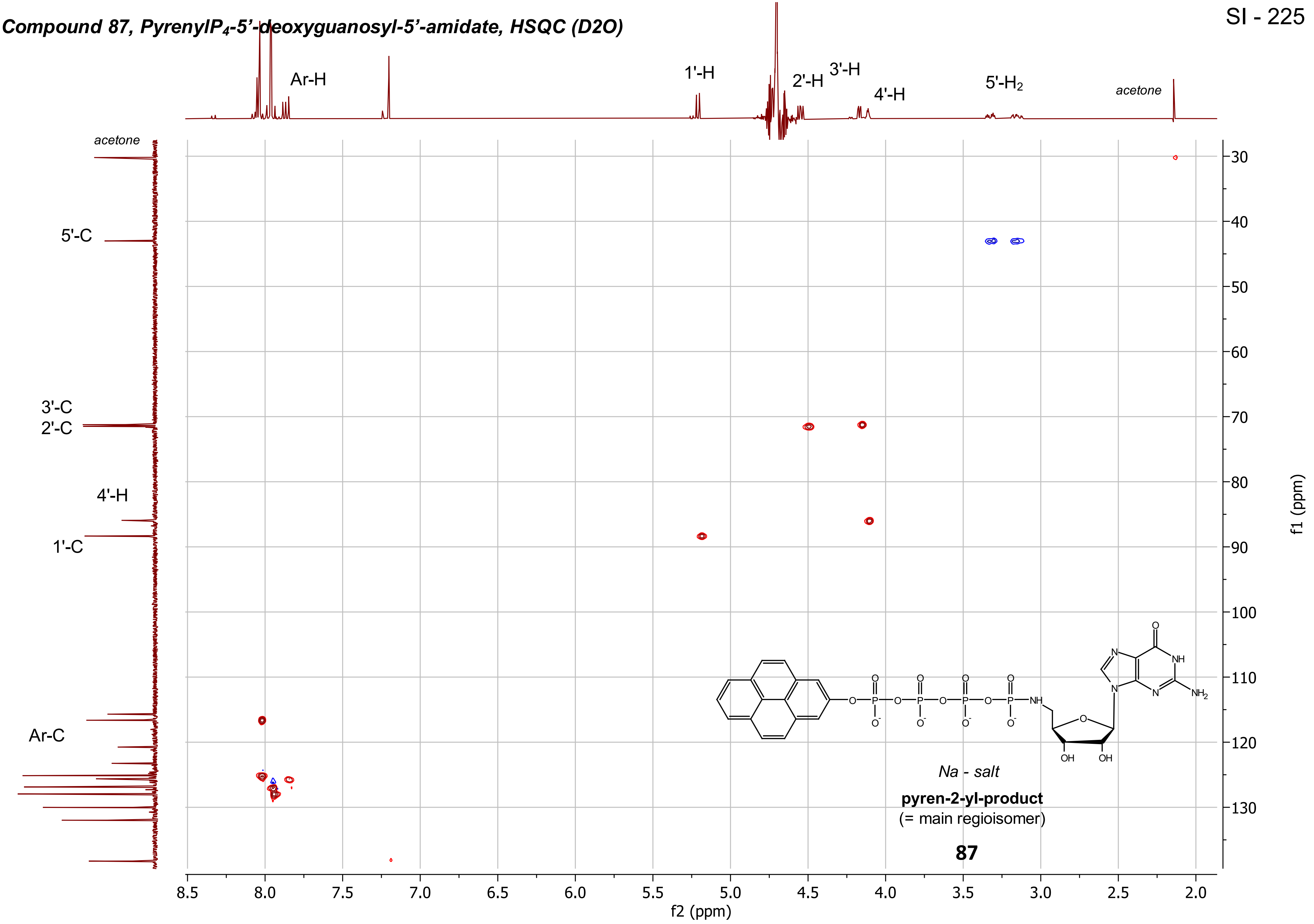

Compound 87, PyrenylP$_4$-5'-deoxyguanosyl-5'-amidate, ^{1}H - ^{31}P - HMBC (D2O)

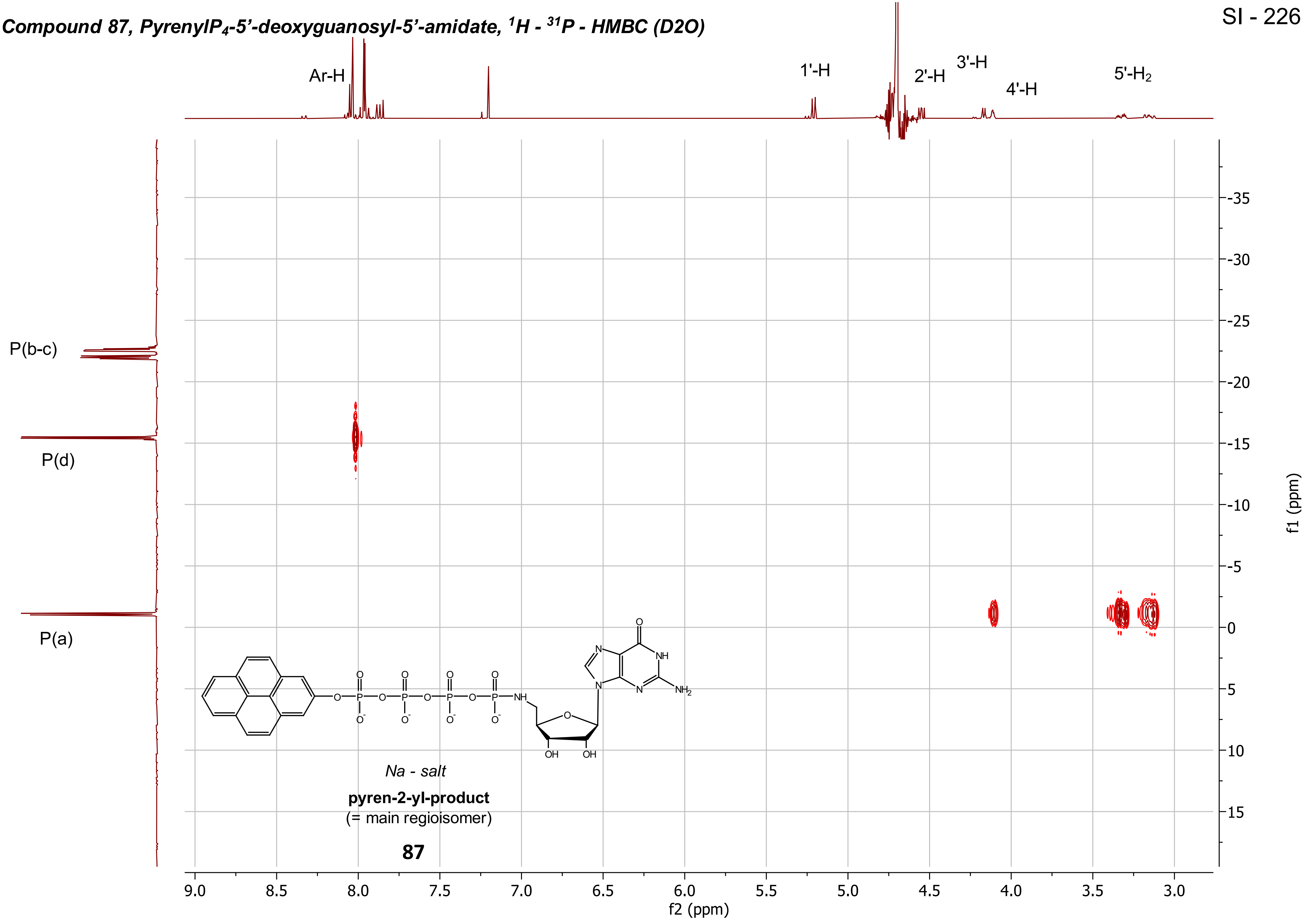

Compound 87, PyrenylP$_4$-5'-deoxyguanosyl-5'-amidate, $^{13}C\{^1H\}$ - NMR (D2O, 101 MHz)

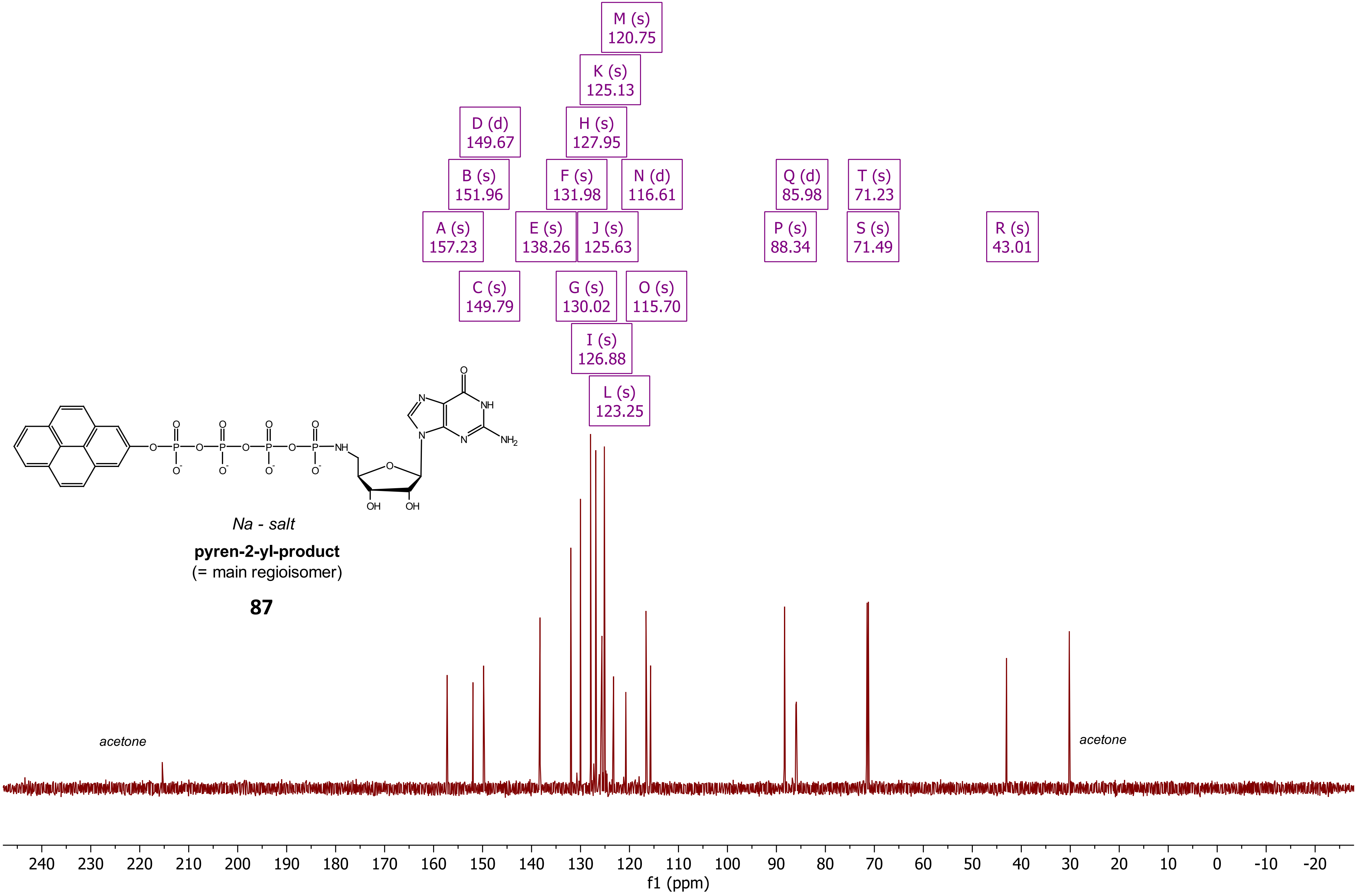

Compound 88, PhenylP$_4$-5'-deoxyguanosyl-5'-amidate, ^{1}H - NMR (D2O, 400 MHz)

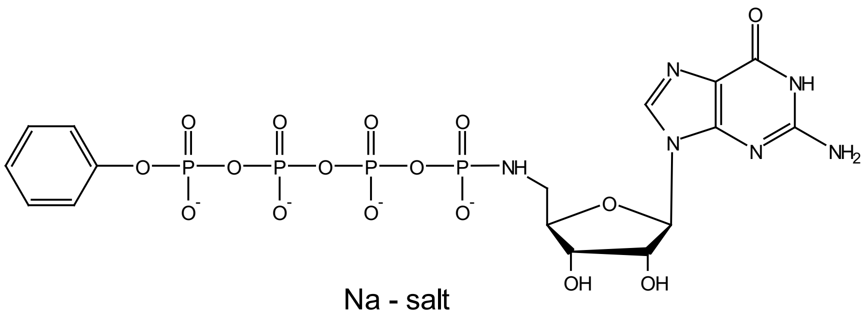

Na - salt

88

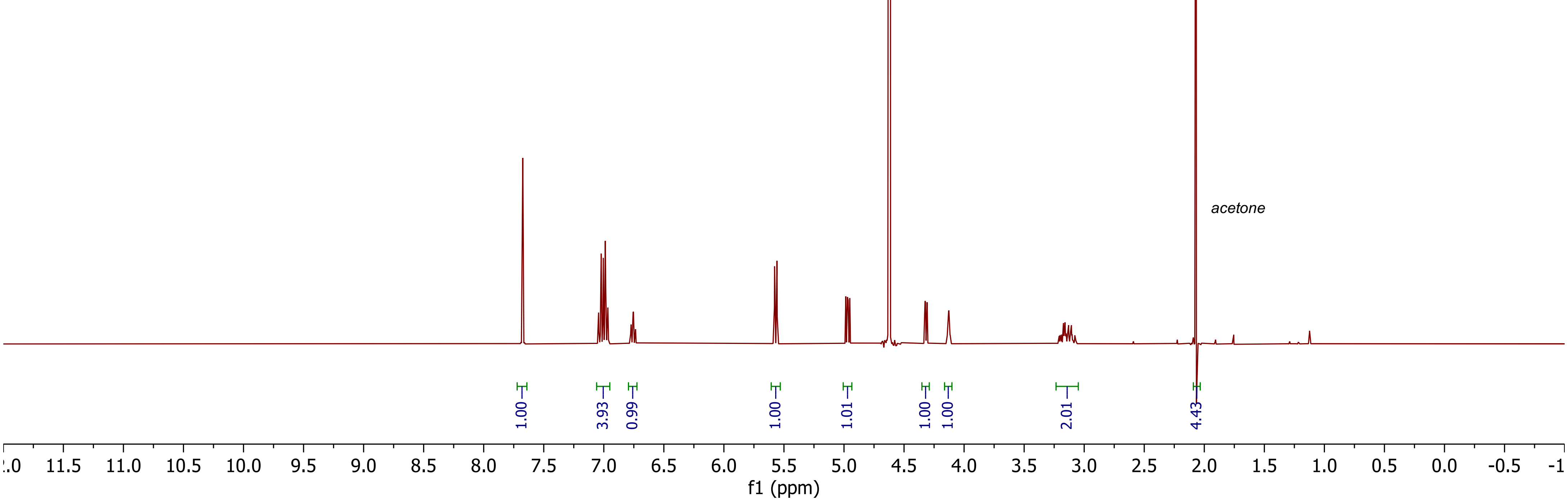

Compound 88, PhenylP$_4$-5'-deoxyguanosyl-5'-amidate, $^{31}P\{^1H\}$ - NMR (D2O, 162 MHz)

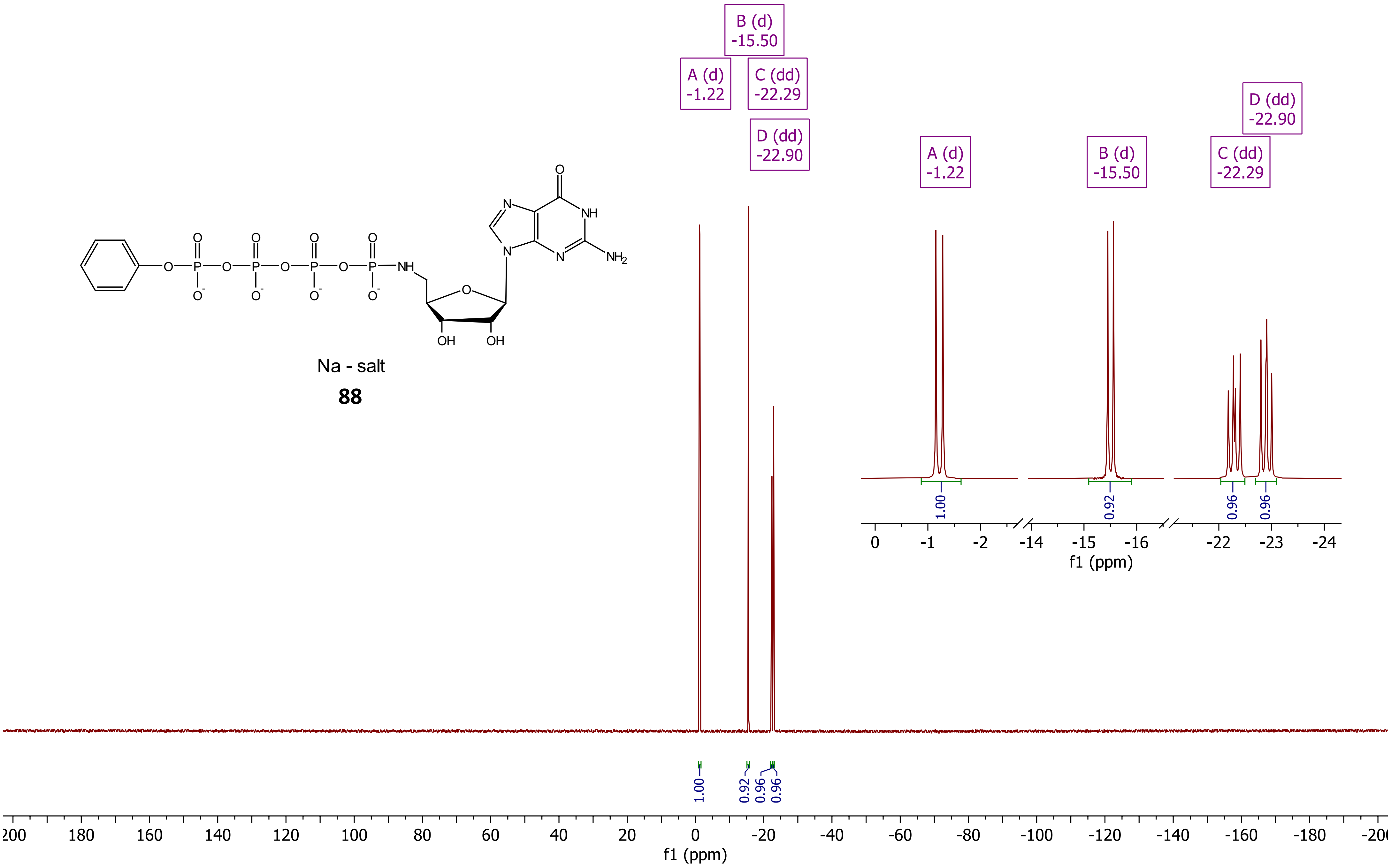

Compound 88, PhenylP$_4$-5'-deoxyguanosyl-5'-amidate, DQF-COSY (D2O)

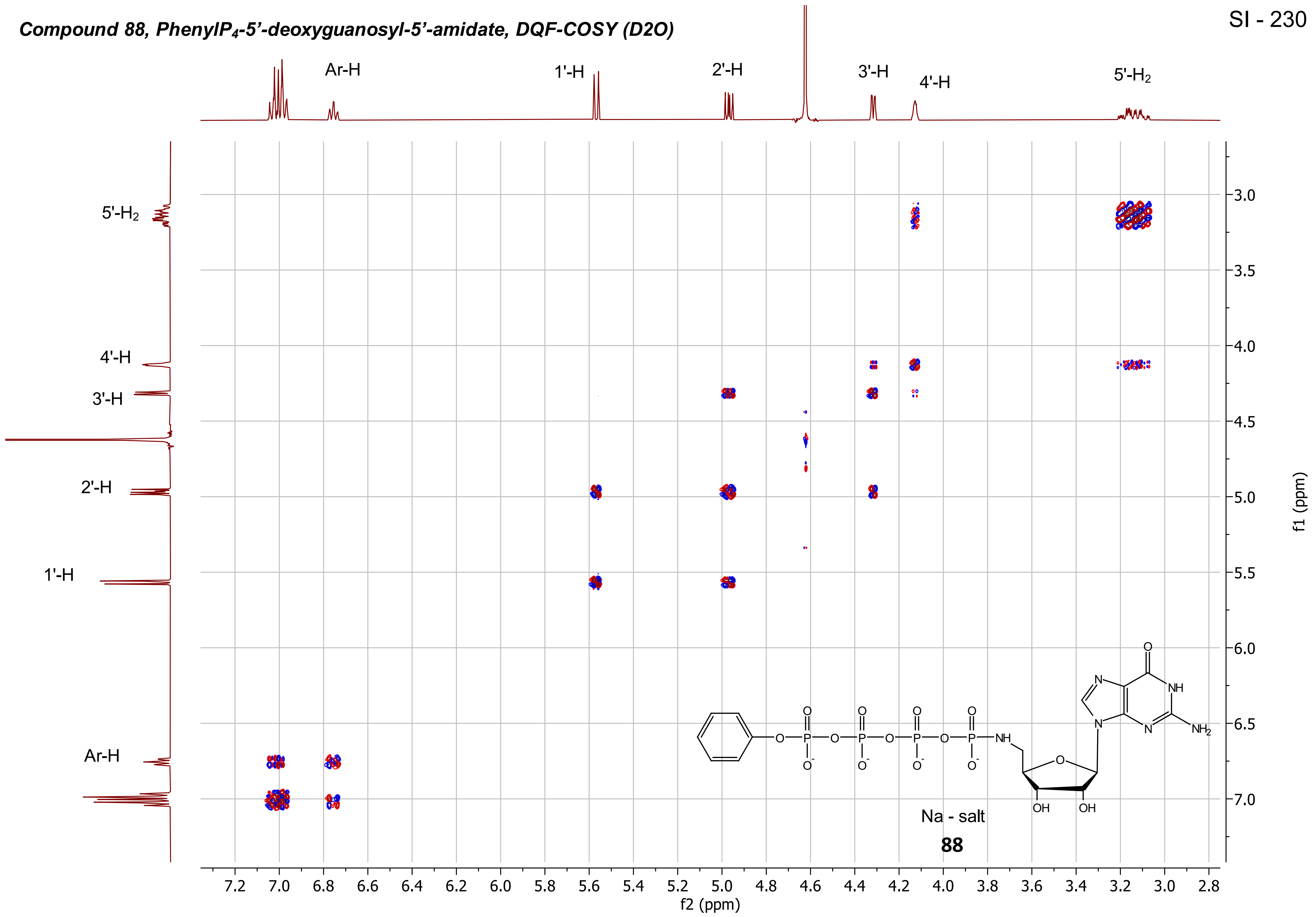

Compound 88, PhenylP$_4$-5'-deoxyguanosyl-5'-amidate, HSQC (D2O)

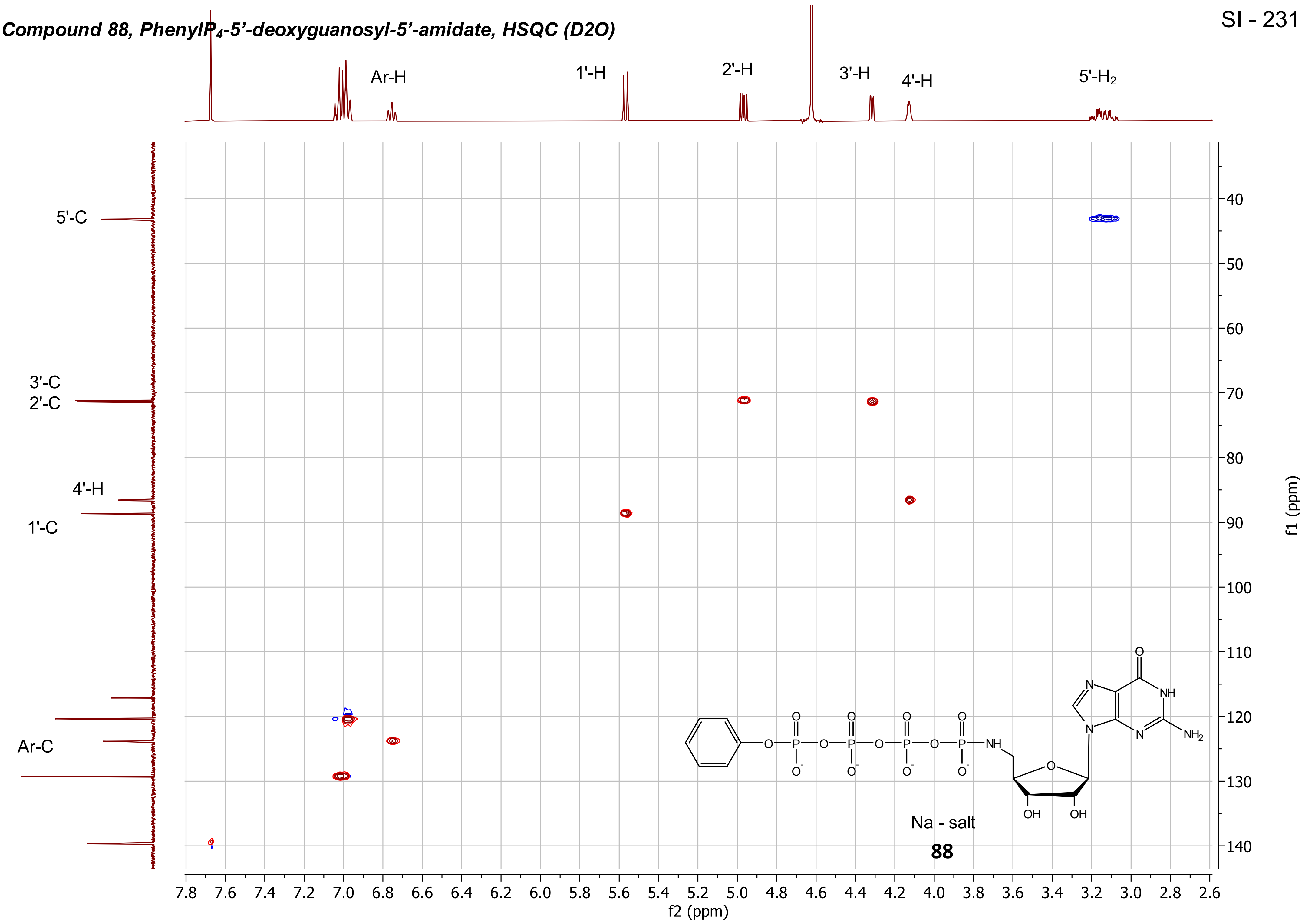

Compound 88, PhenylP$_4$-5'-deoxyguanosyl-5'-amidate, ^1H - ^{31}P - HMBC (D2O)

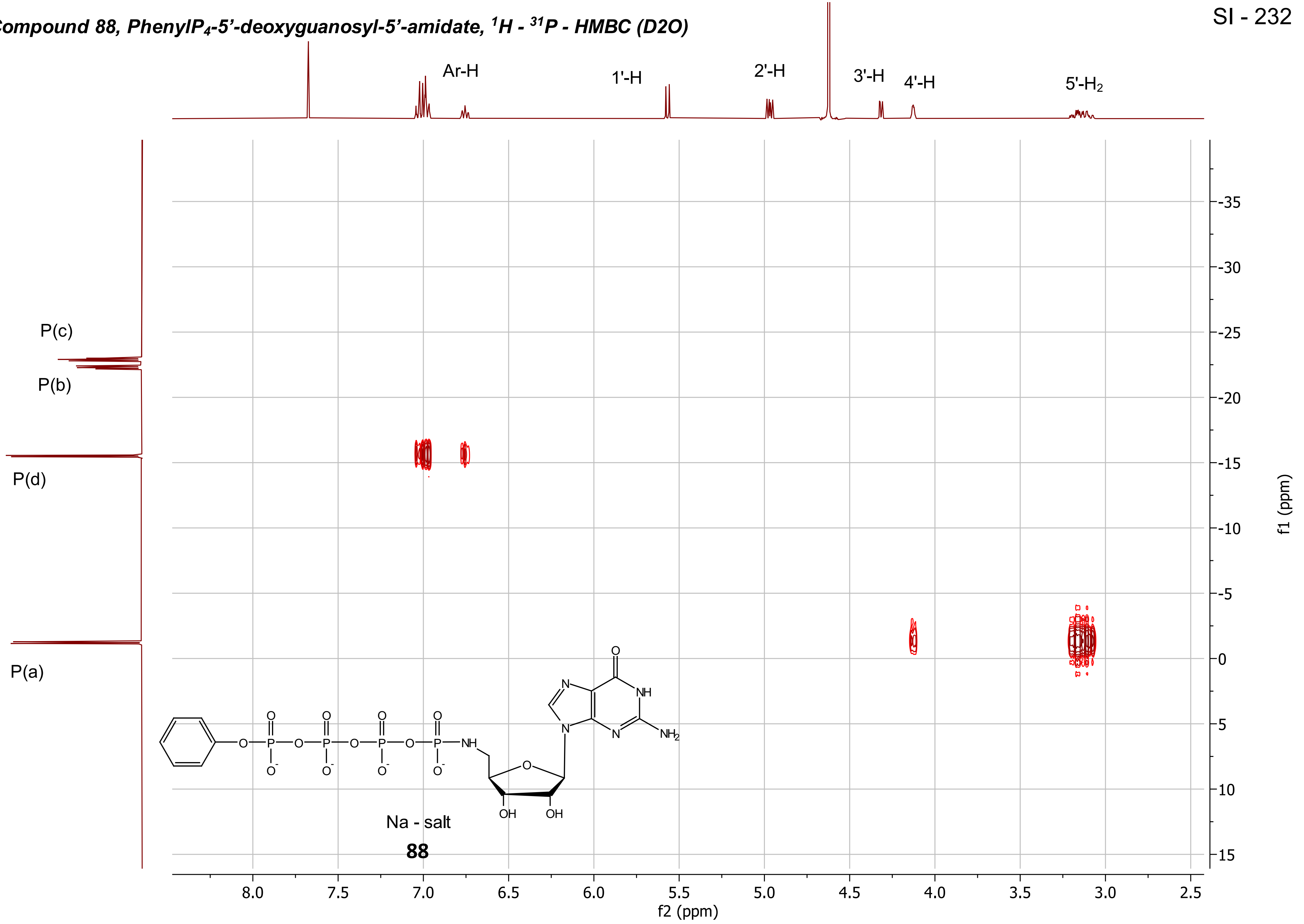

Compound 88, PhenylP$_4$-5'-deoxyguanosyl-5'-amidate, ^{31}P - ^{31}P - COSY (D2O)

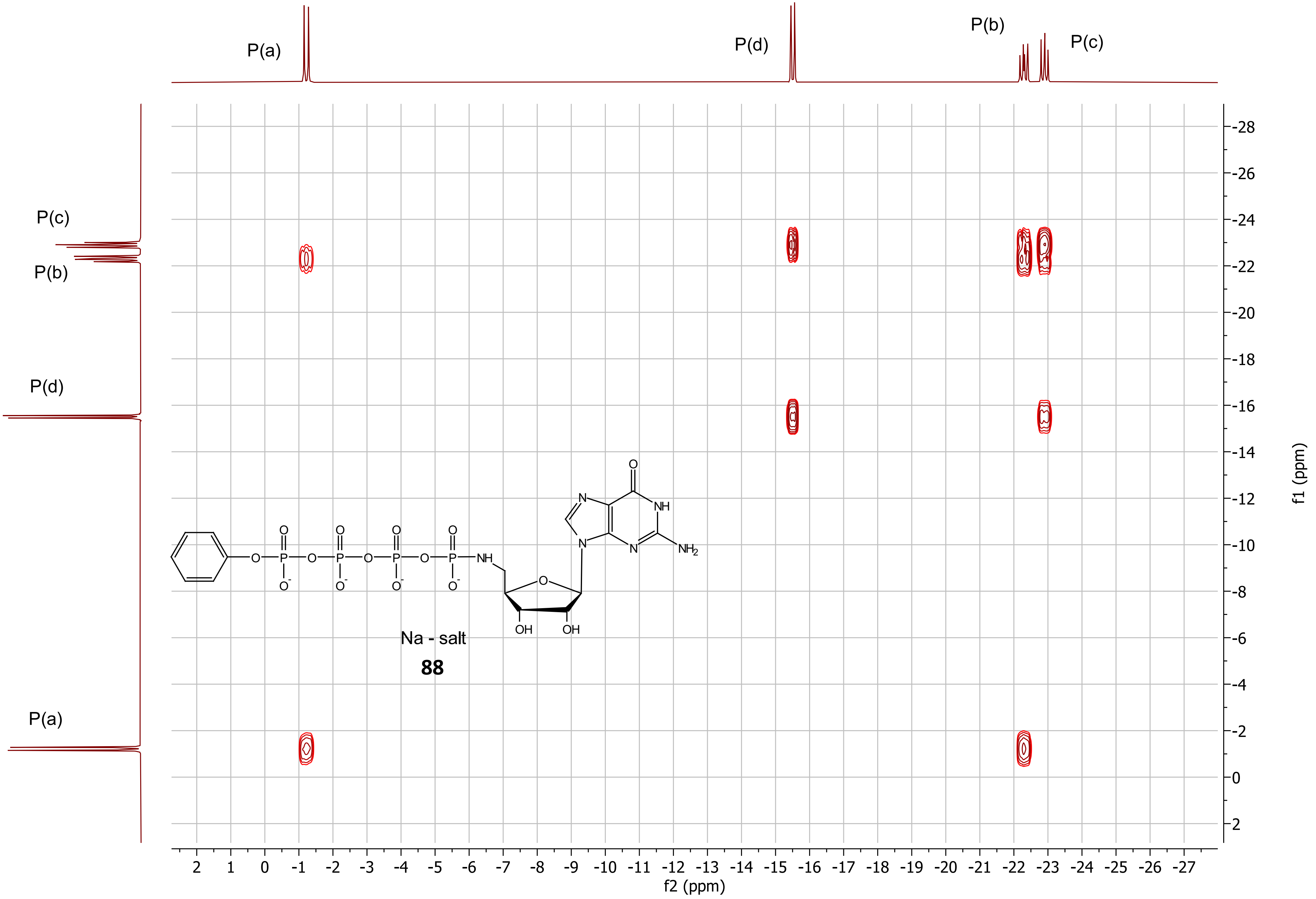

Compound 88, PhenylP$_4$-5'-deoxyguanosyl-5'-amidate, $^{13}C\{^1H\}$ - NMR (D2O, 101 MHz)

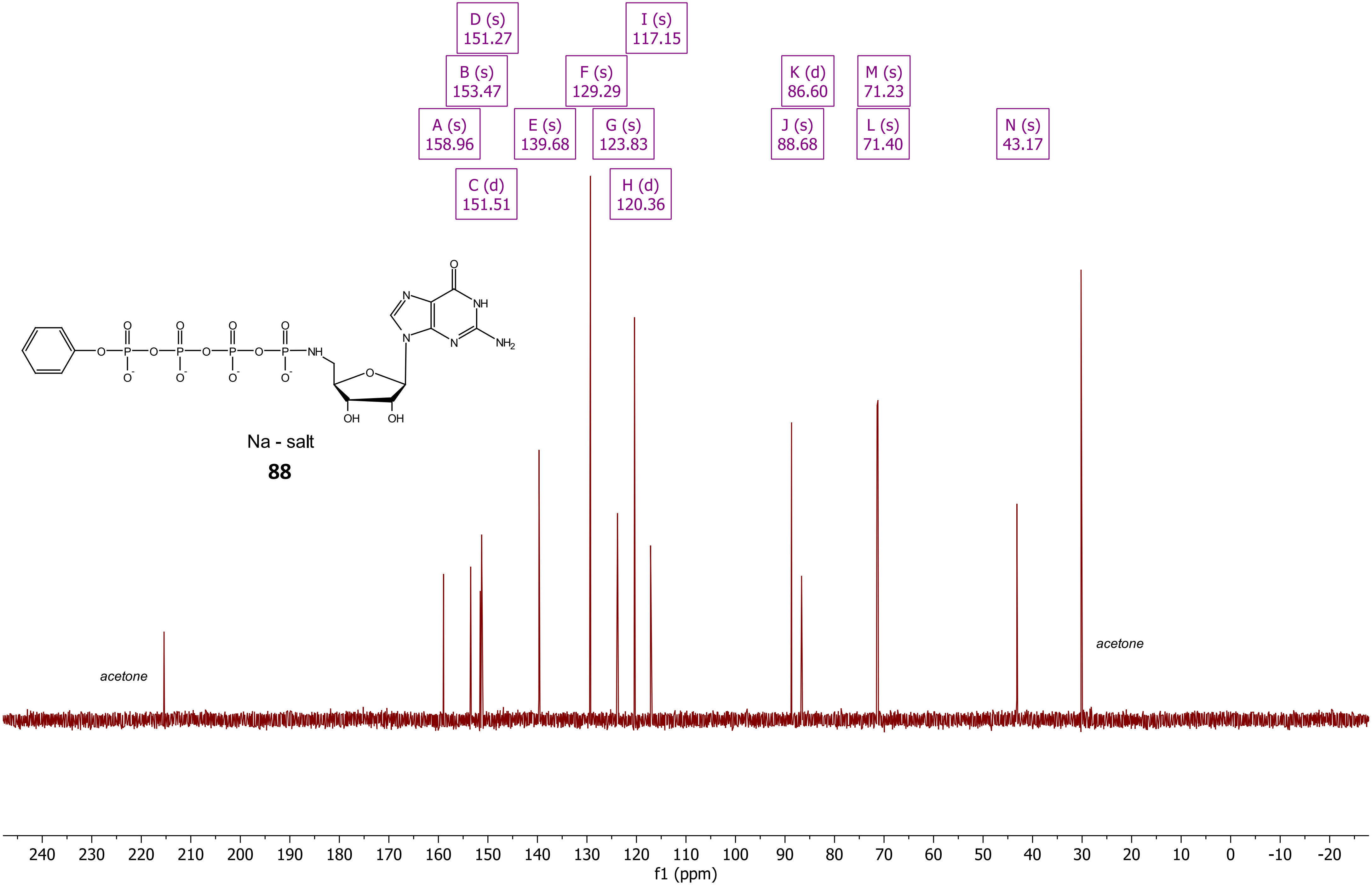

Compound 89, 3,4-DimethylphenylP$_4$-(7-(diethylamino)-2-oxo-2H-chromen-4-yl)methylamidate, ^{1}H - NMR (D2O, 400 MHz)

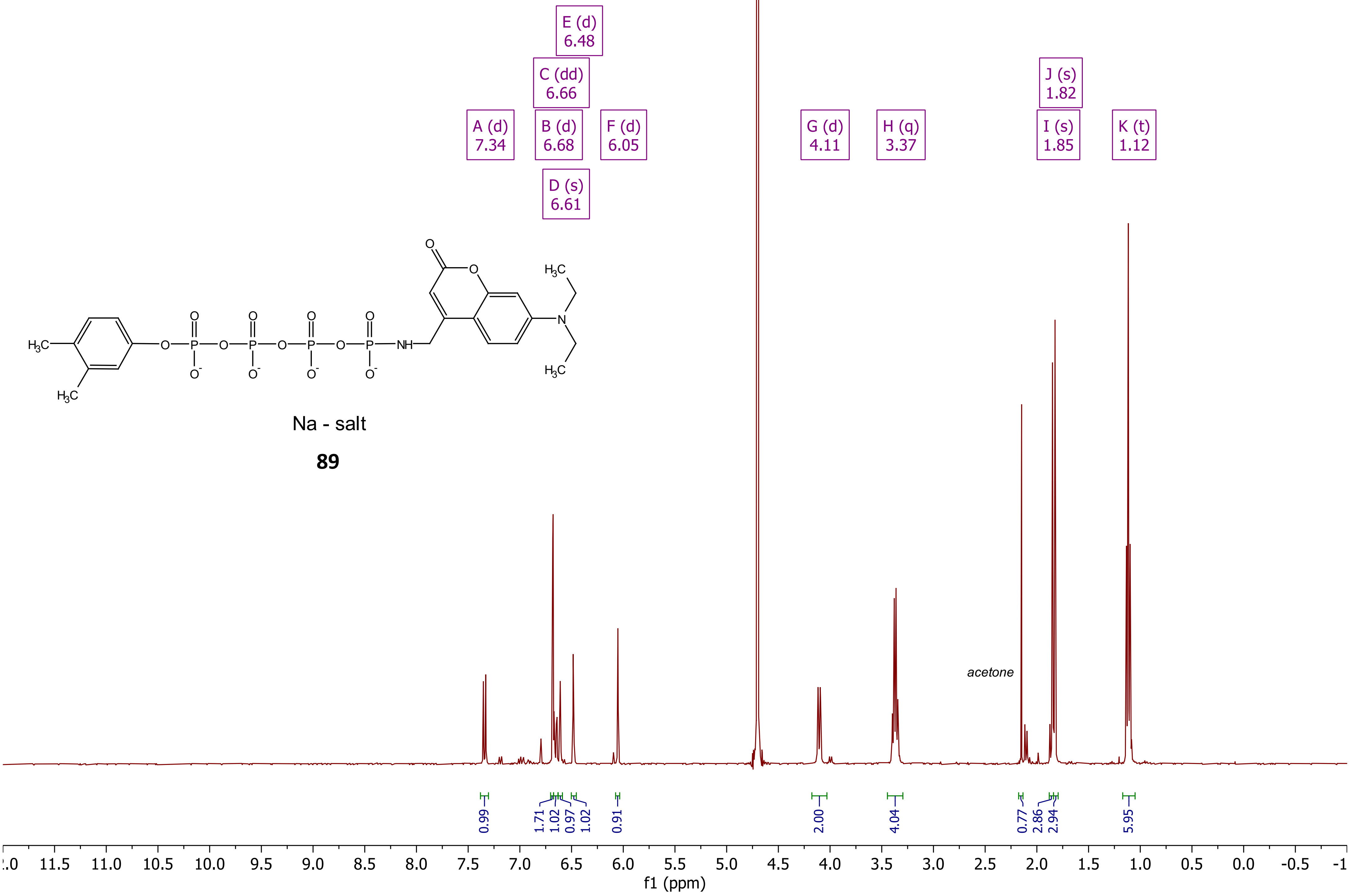

Compound 89, 3,4-DimethylphenylP$_4$-(7-(diethylamino)-2-oxo-2H-chromen-4-yl)methylamidate, ^{31}P {^{1}H} - NMR (D2O, 162 MHz)

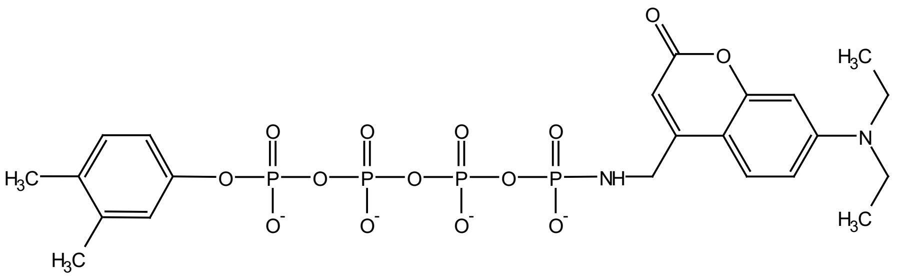

Na - salt

89

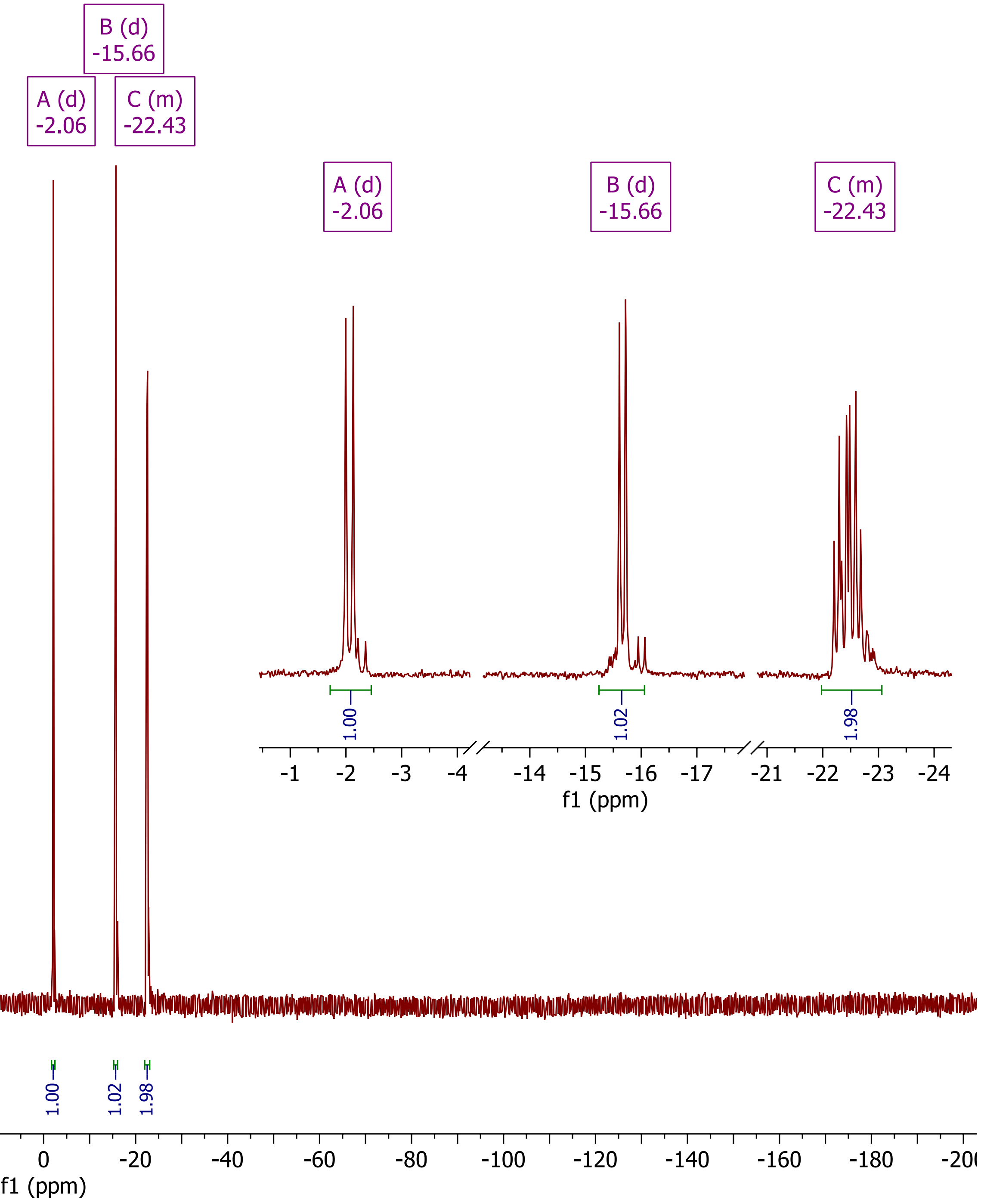

Compound 89, 3,4-DimethylphenylP$_4$-(7-(diethylamino)-2-oxo-2H-chromen-4-yl)methylamidate, ^1H - ^{31}P - HMBC (D2O)

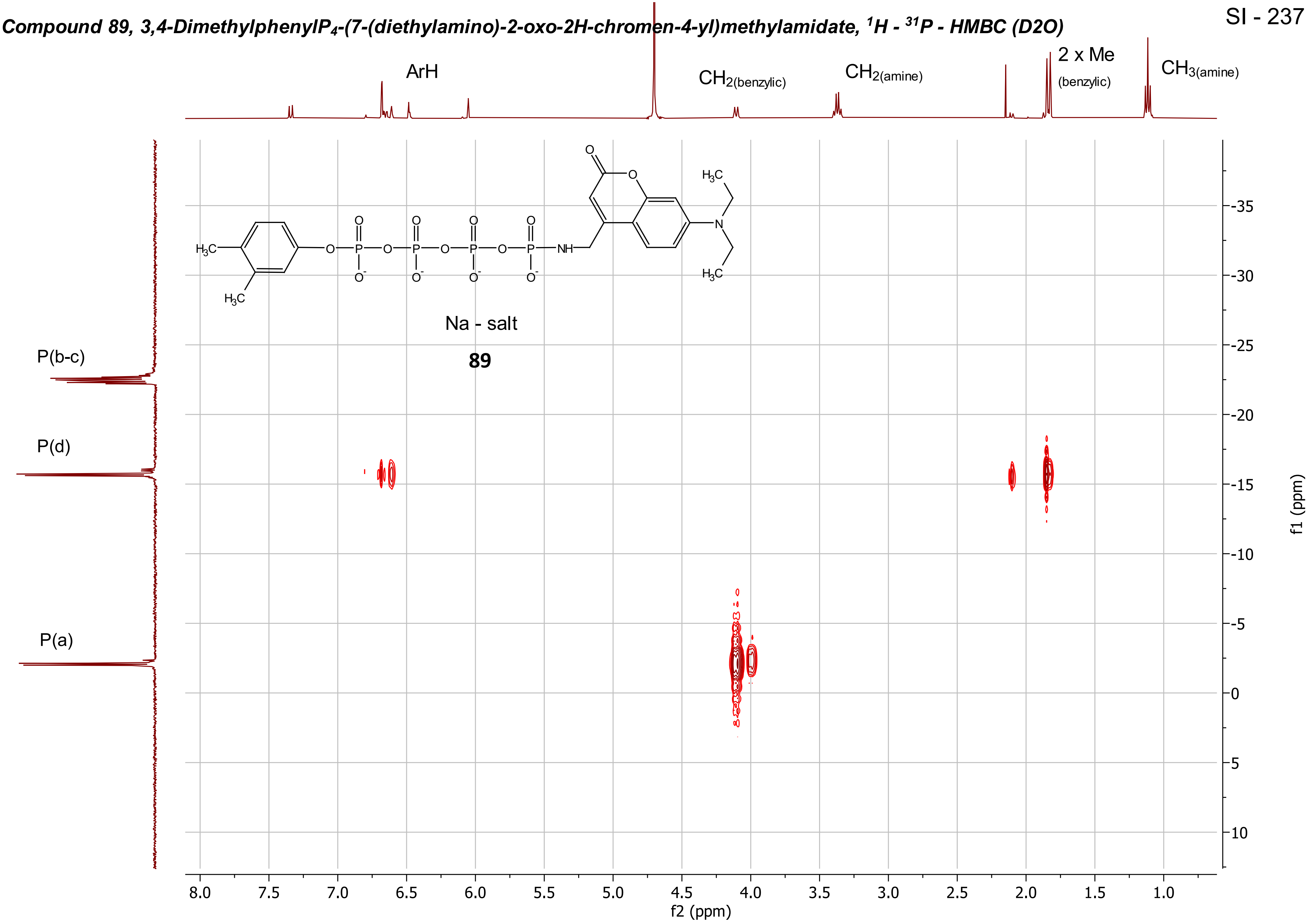

Compound 89, 3,4-DimethylphenylP$_4$-(7-(diethylamino)-2-oxo-2H-chromen-4-yl)methylamidate, $^{13}C\{^1H\}$ - NMR (D2O, 101 MHz)

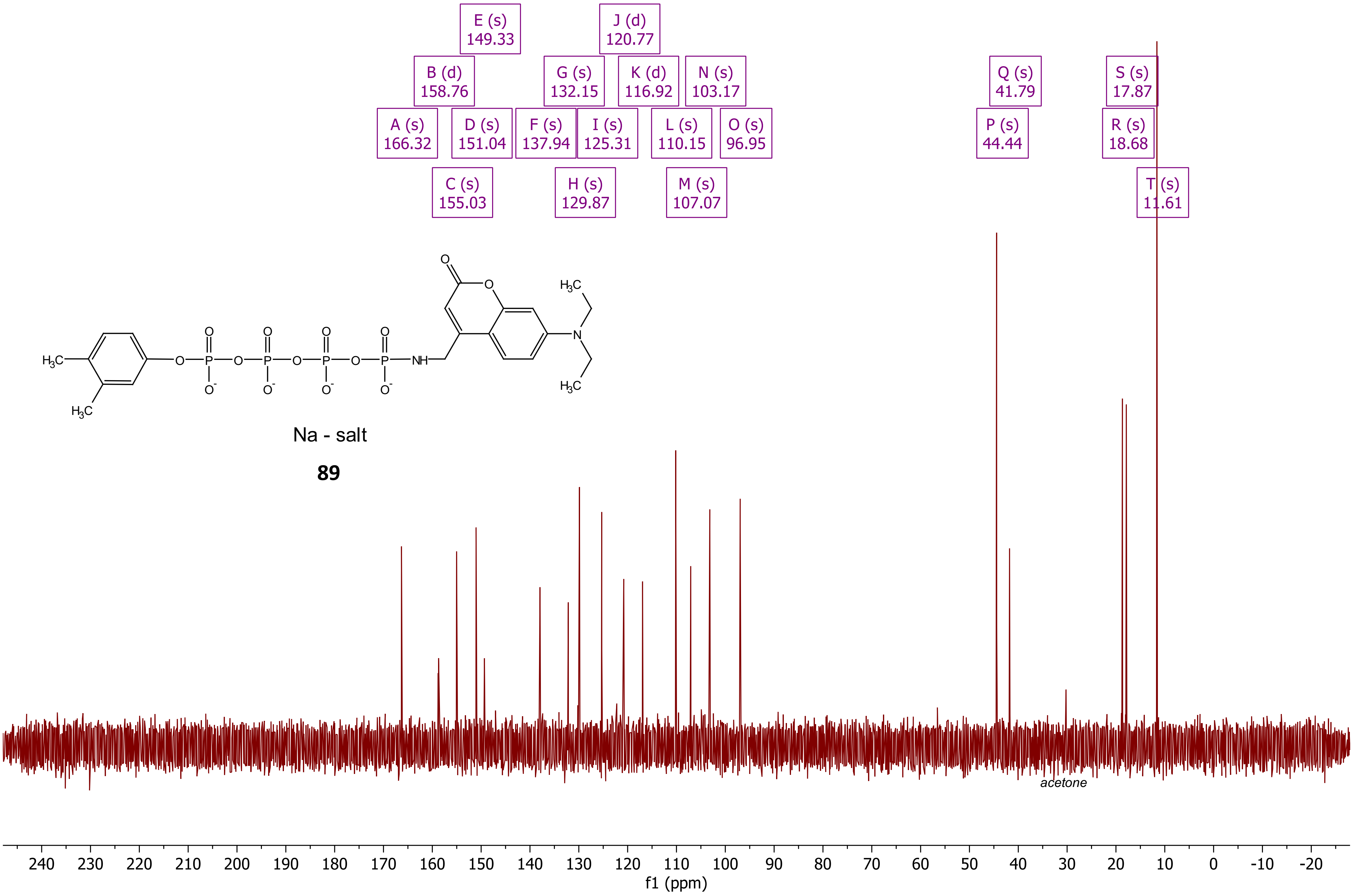

Compound 90, PhenylIP$_5$-propargylamidate, ^{1}H - NMR (D2O, 400 MHz)

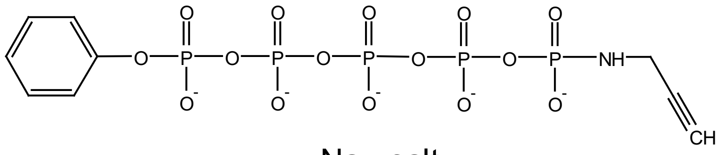

Na - salt

90

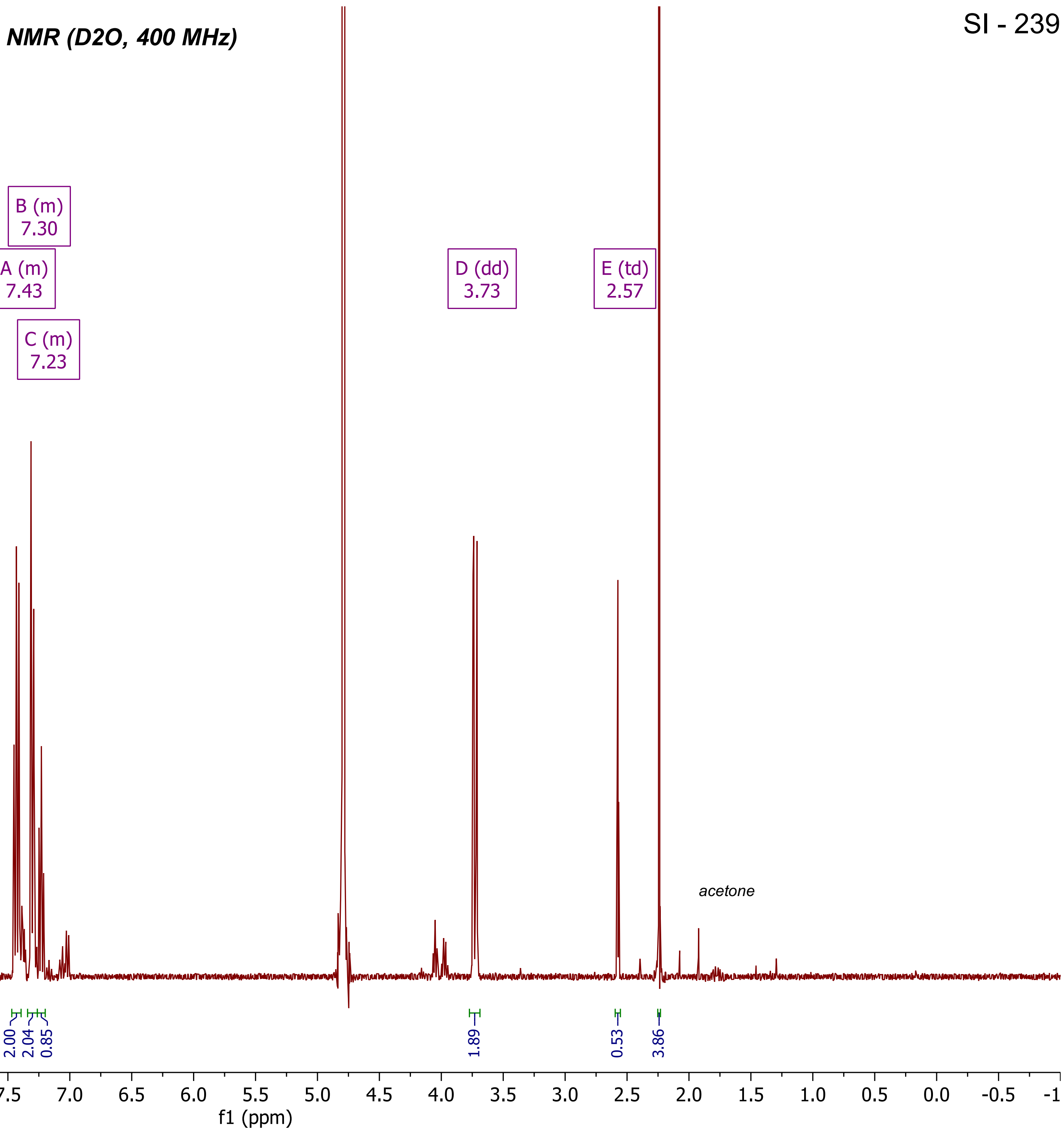

Compound 90, PhenylP$_5$-propargylamidate, ^{31}P {^{1}H} - NMR (D2O, 162 MHz)

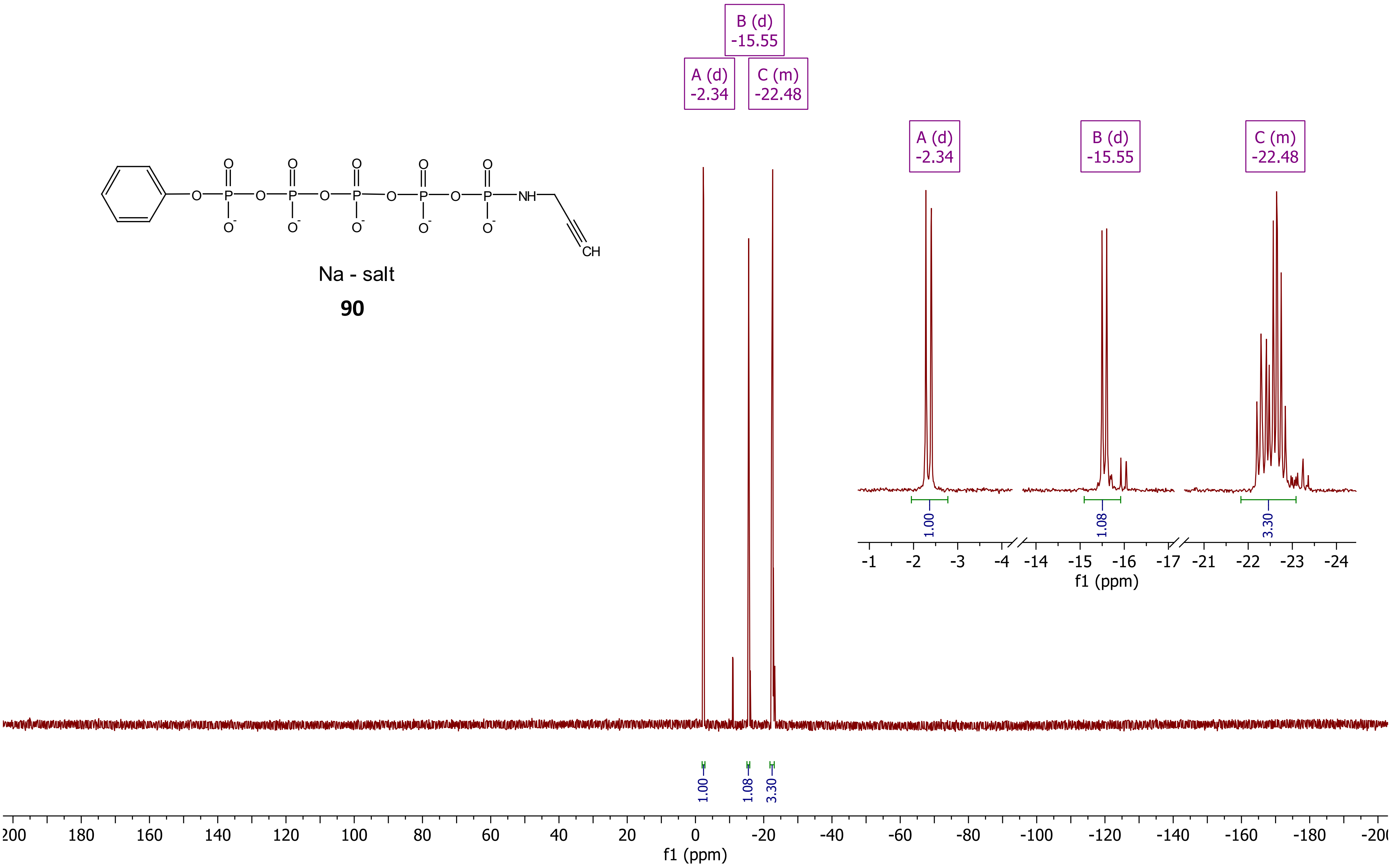

Compound 90, PhenylP$_5$-propargylamidate, ^1H - ^{31}P - HMBC (D2O)

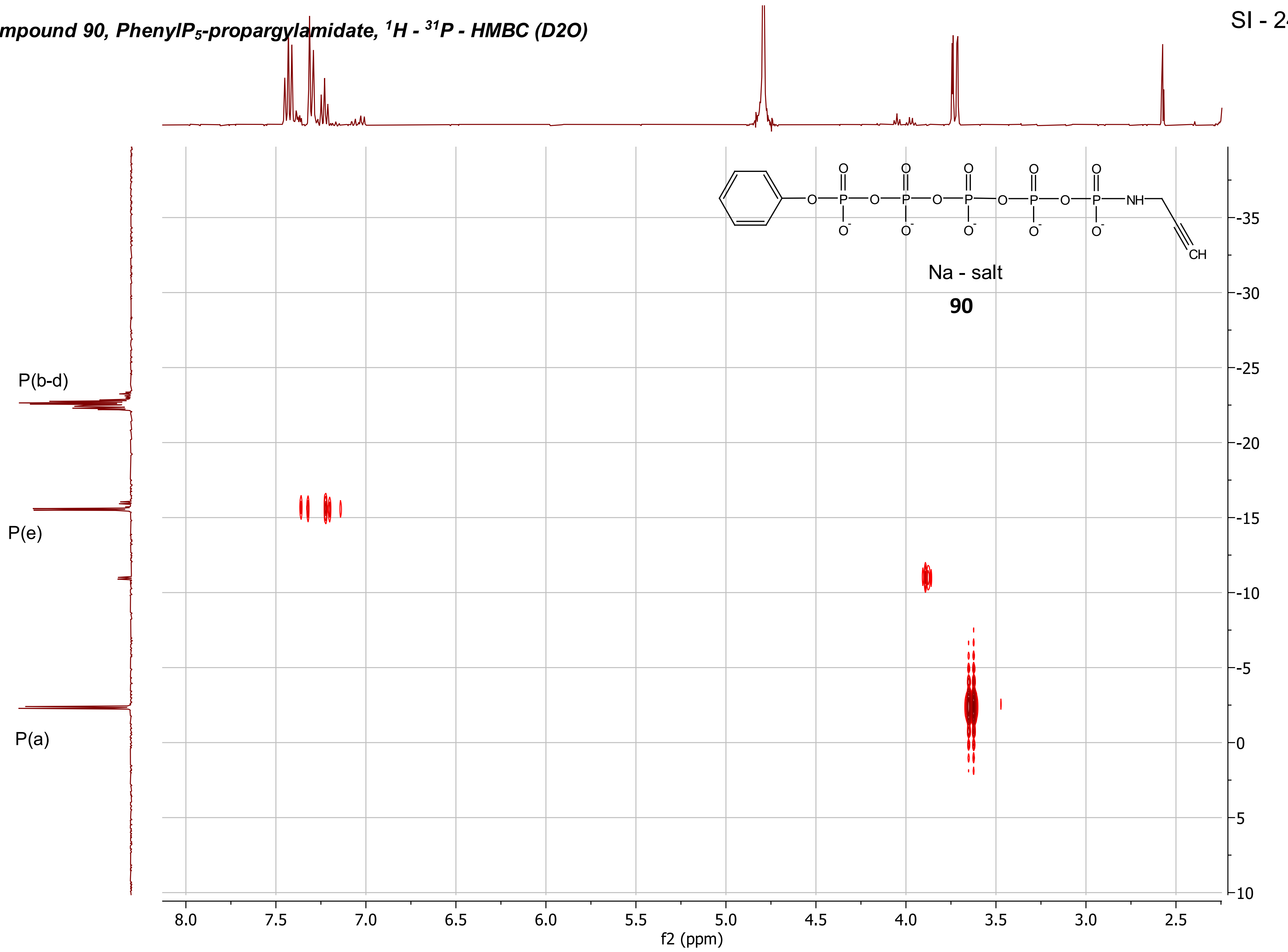

Compound 90, PhenylIP$_5$-propargylamidate, $^{13}C\{^1H\}$ - NMR (D2O, 101 MHz)

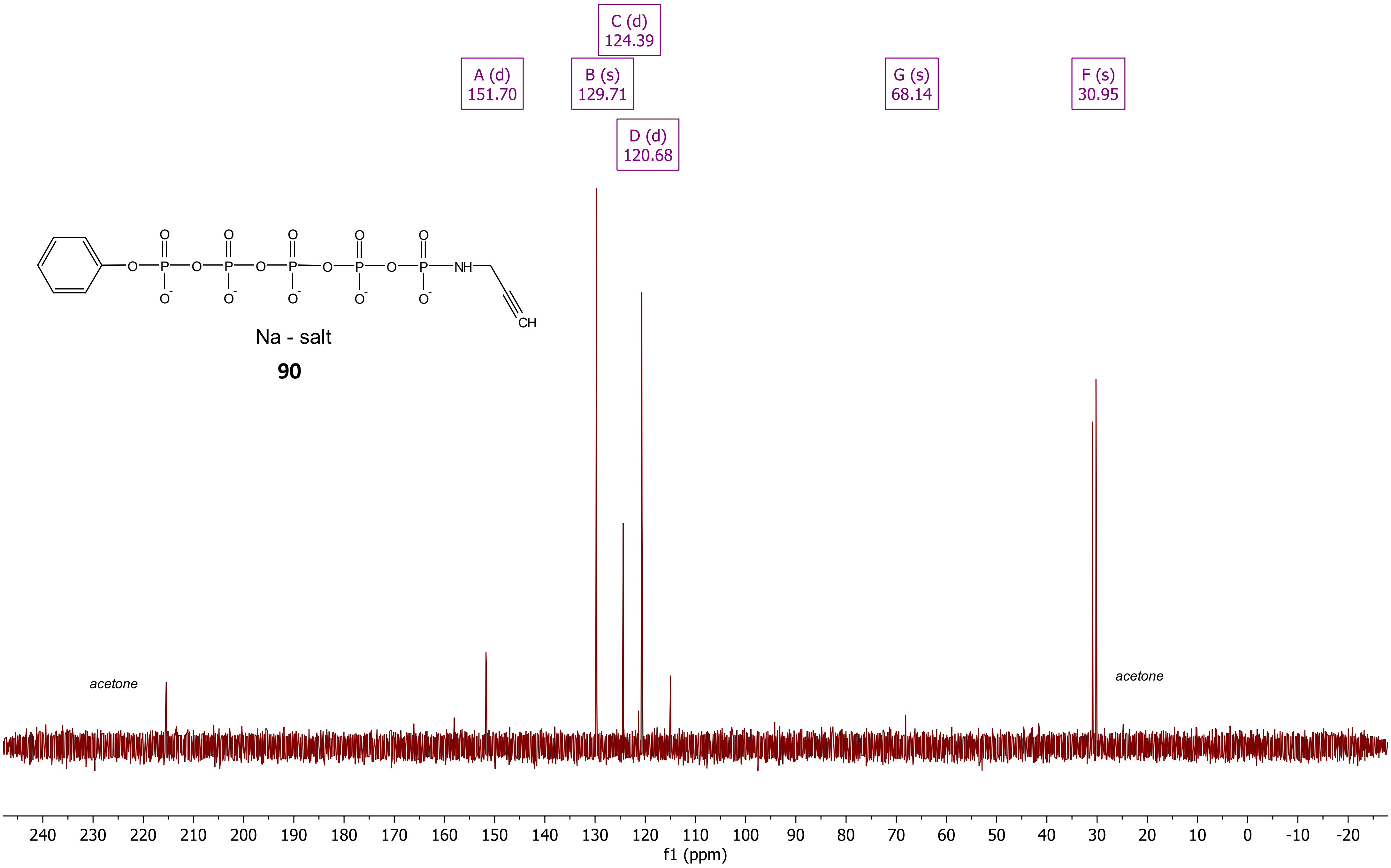

Compound 91, PhenylP$_7$-propargylamidate, ^{1}H - NMR (D2O, 400 MHz)

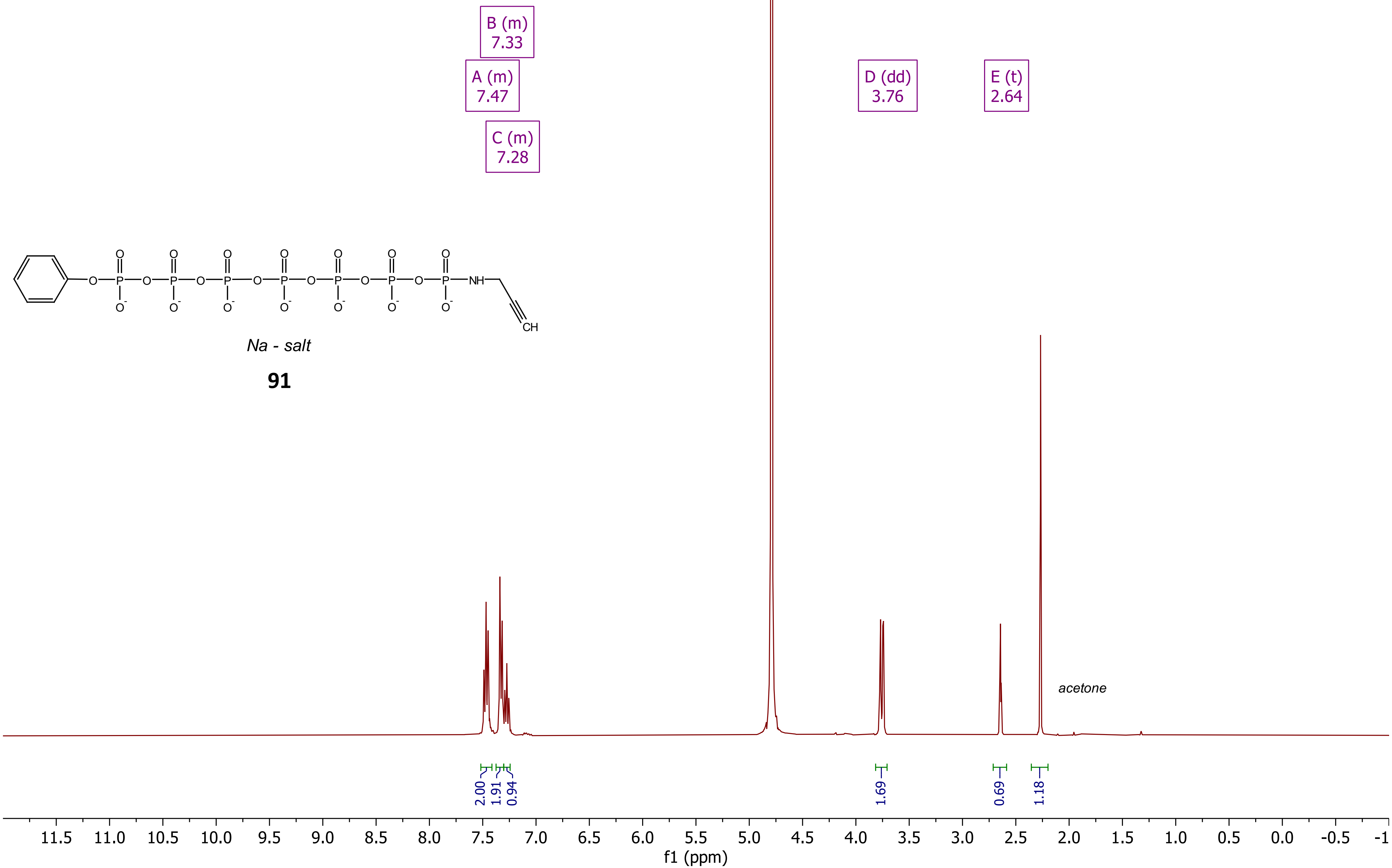

Compound 91, PhenylP$_7$-propargylamidate, ^{31}P {^{1}H} - NMR (D2O, 162 MHz)

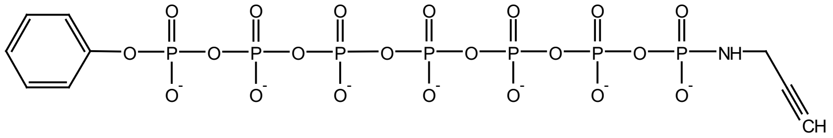

Na - salt

91

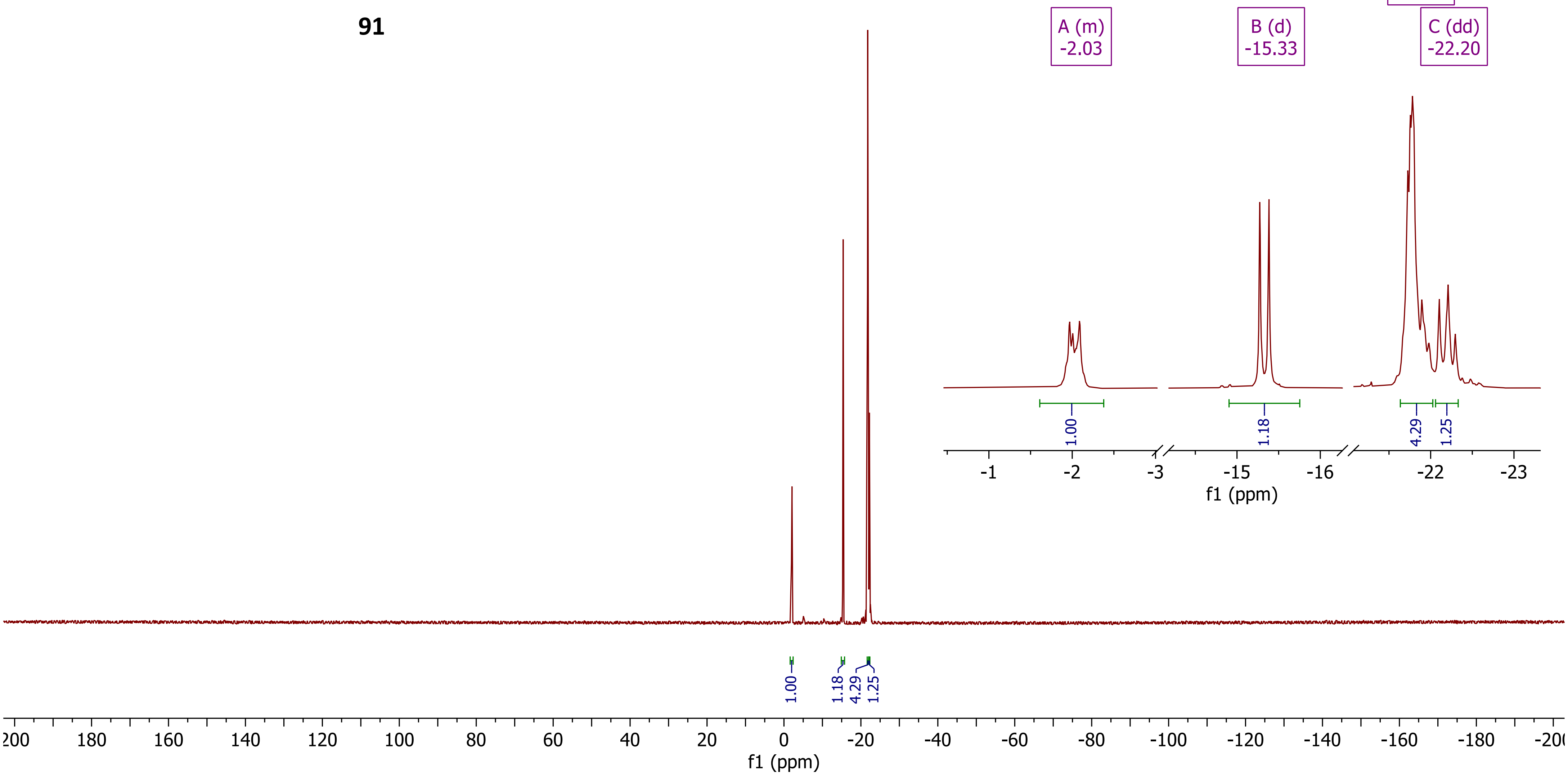

Compound 91, PhenylP$_7$-propargylamidate, ^{1}H - ^{31}P - HMBC (D2O)

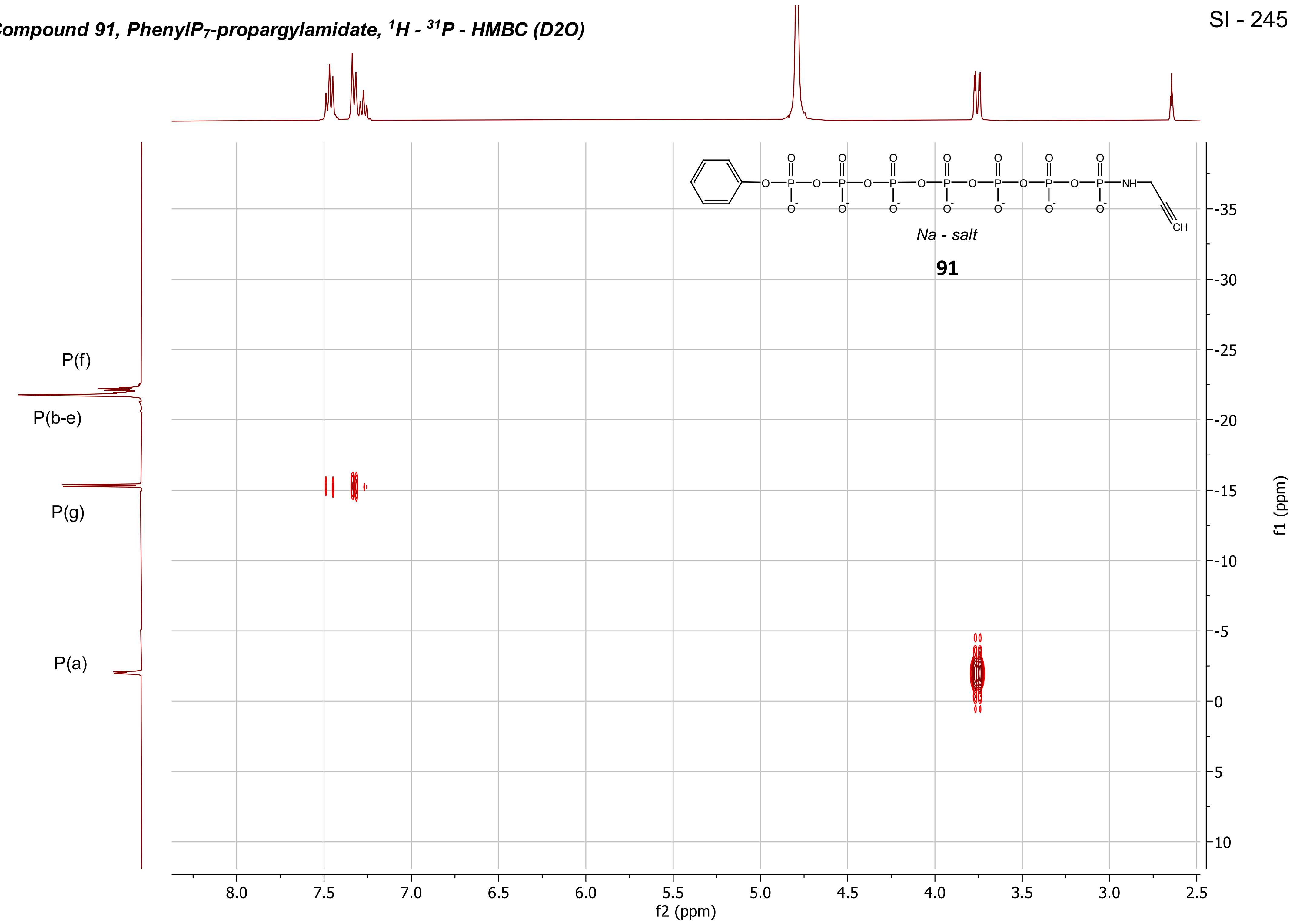

Compound 91, PhenylP$_7$-propargylamidate, PP-COSY (D2O)

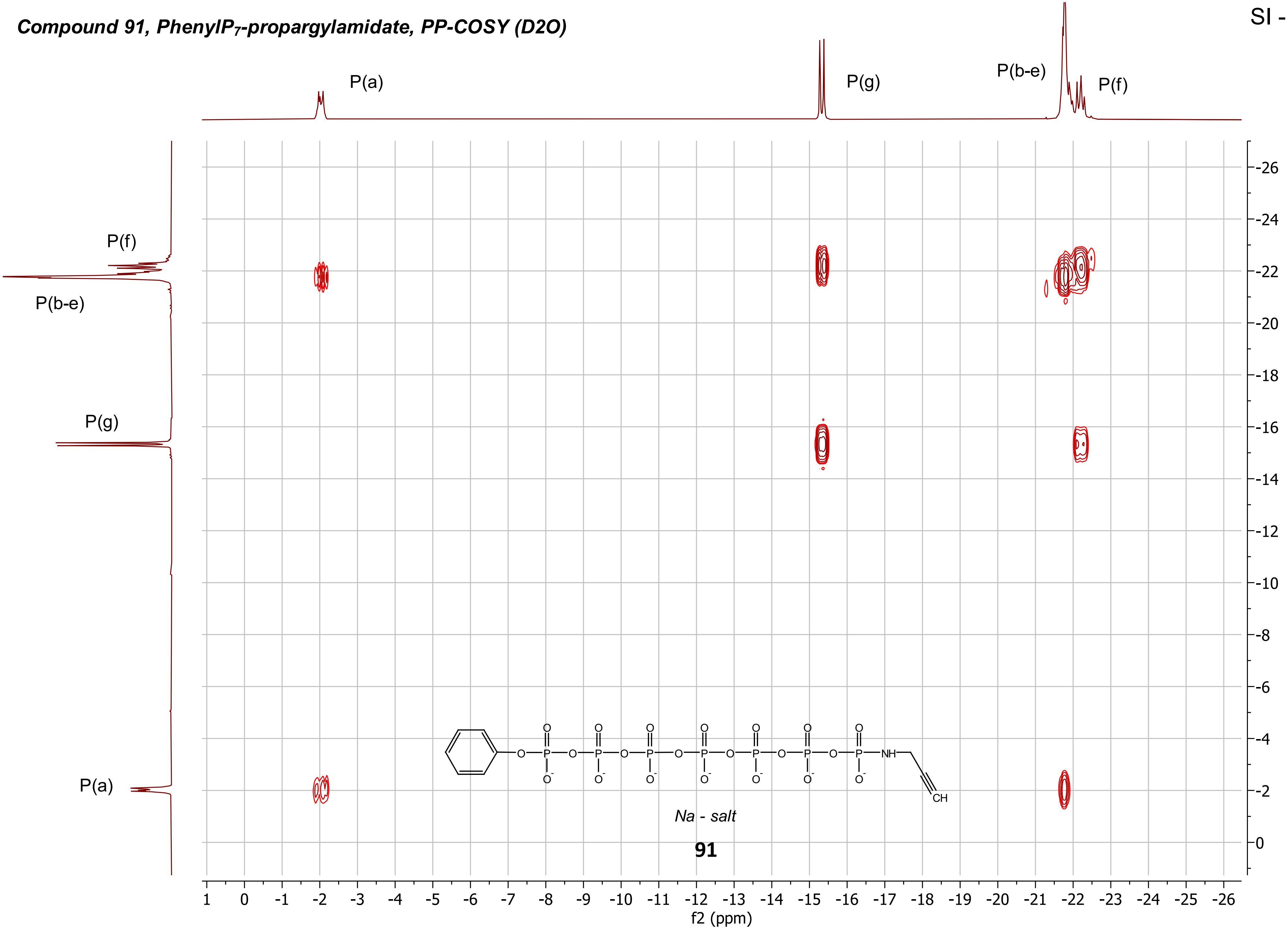

Compound 91, PhenylP$_7$-propargylamidate, $^{13}C\{^1H\}$ - NMR (D2O, 101 MHz)

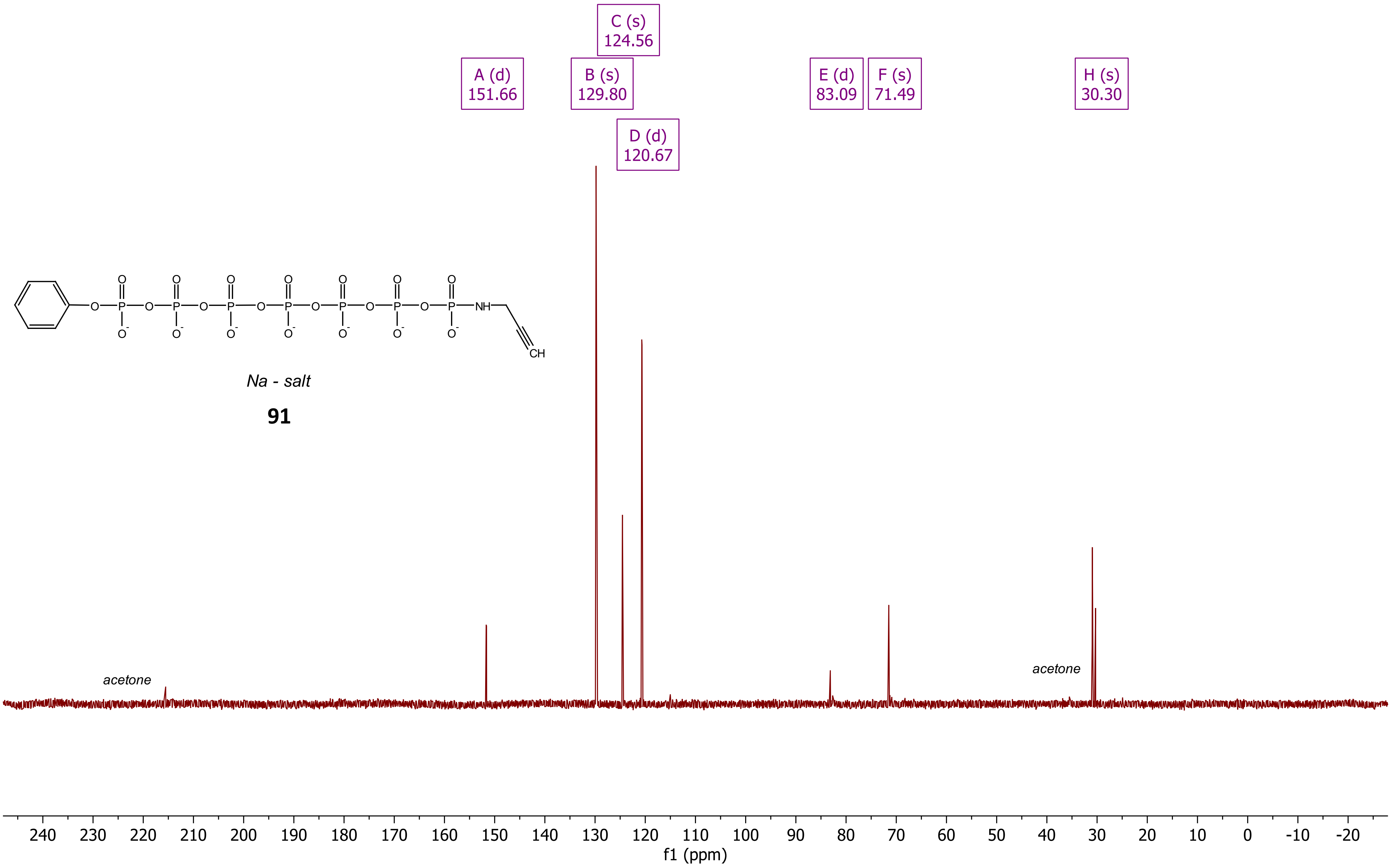

Compound 92, PhenylIP$_8$-propargylamidate, ^{1}H - NMR (D2O, 400 MHz)

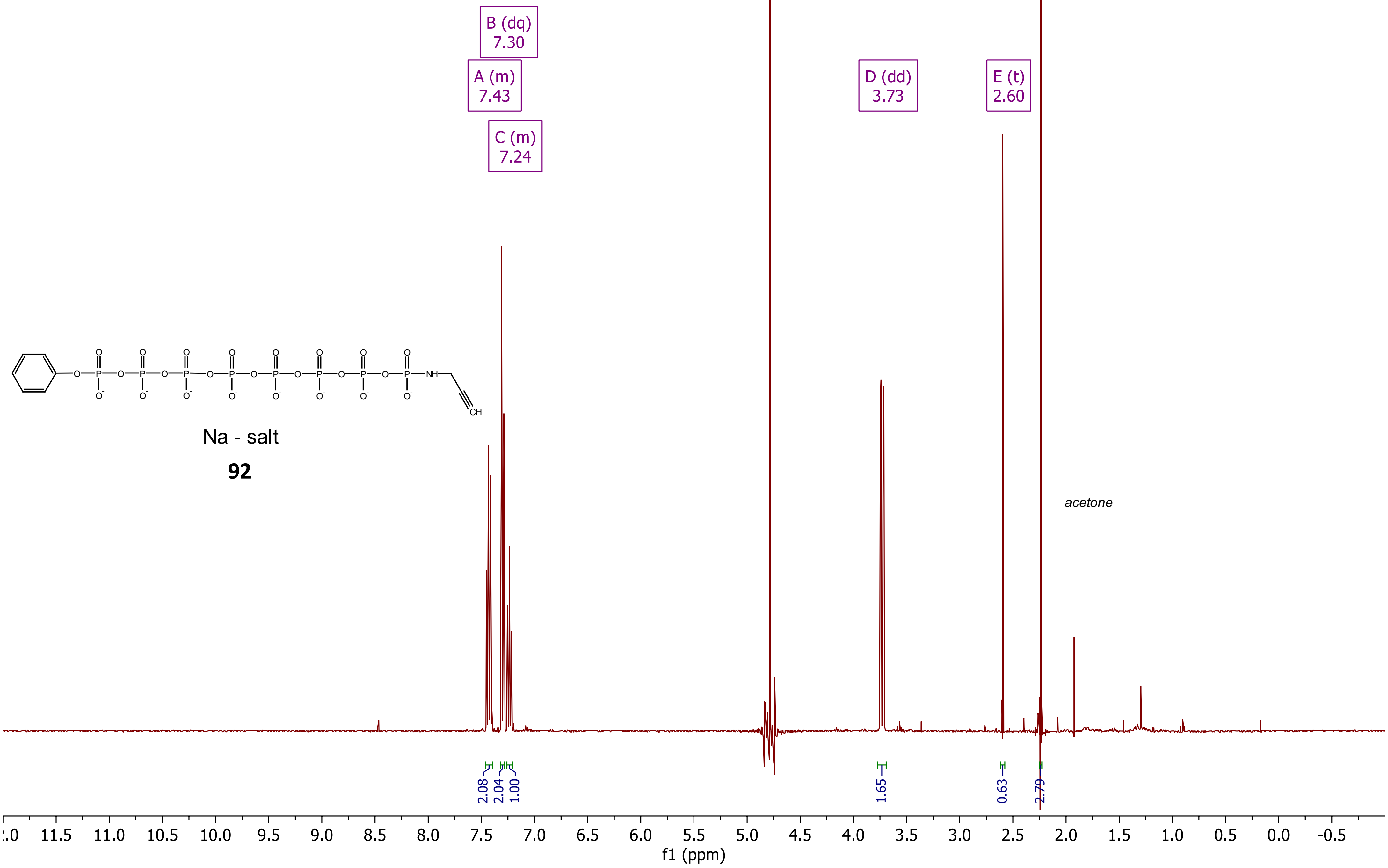

Compound 92, PhenylP$_8$-propargylamidate, $^{31}P\{^1H\}$ - NMR (D2O, 162 MHz)

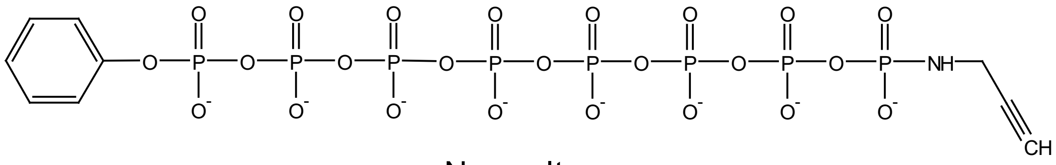

Na - salt

92

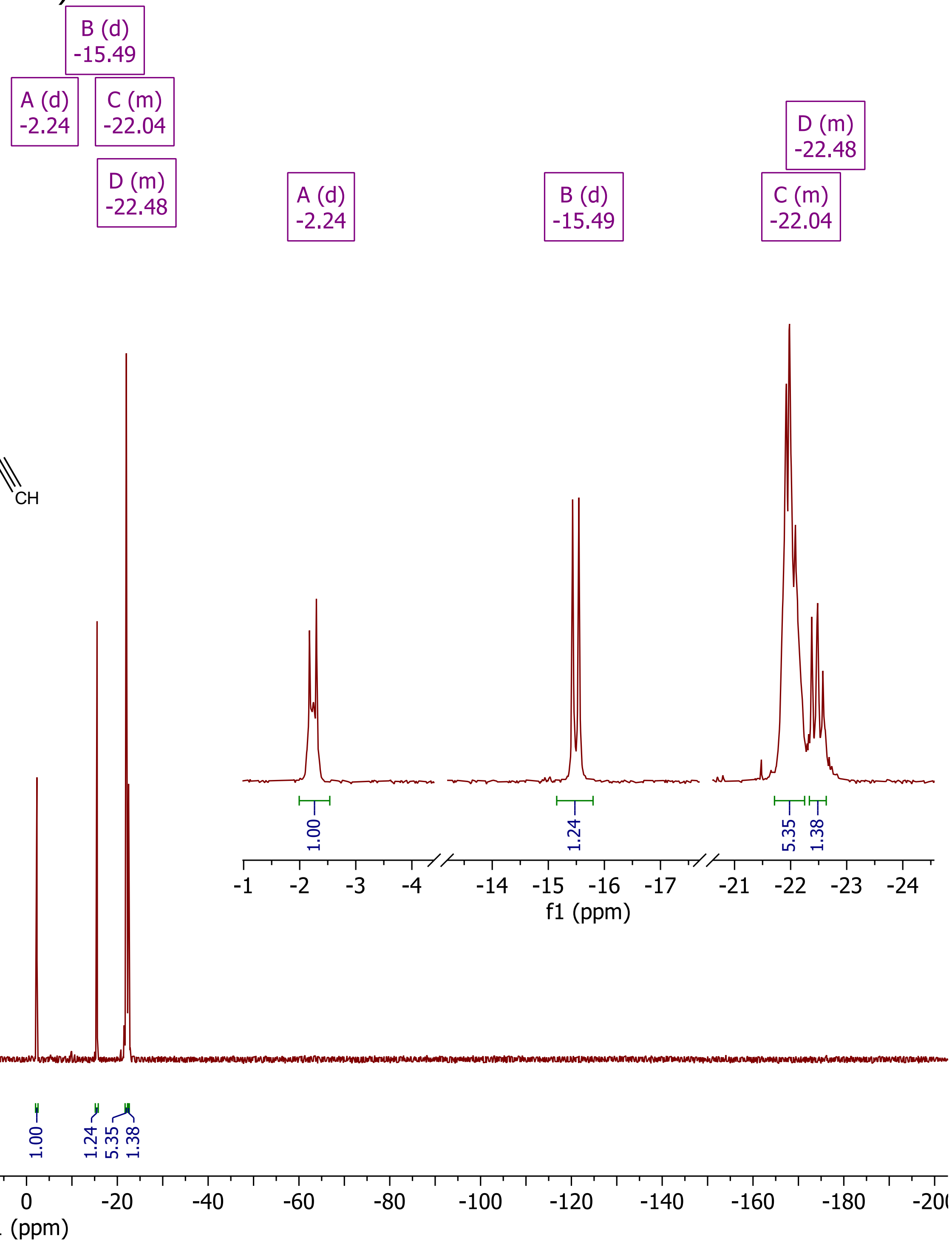

Compound 92, PhenylP$_8$-propargylamidate, ^1H - ^{31}P - HMBC (D2O)

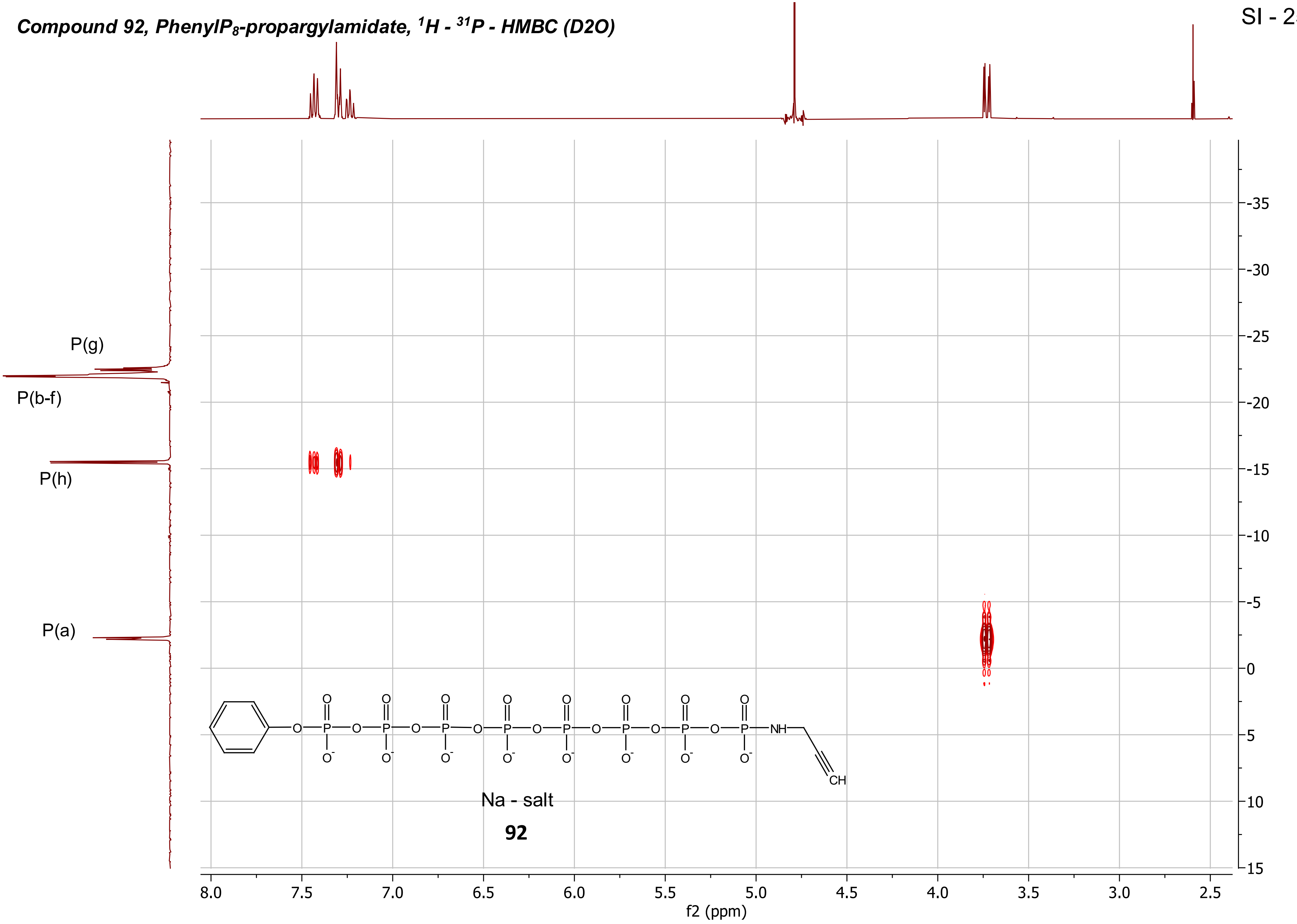

Compound 92, PhenylP$_8$-propargylamidate, PP-COSY (D2O)

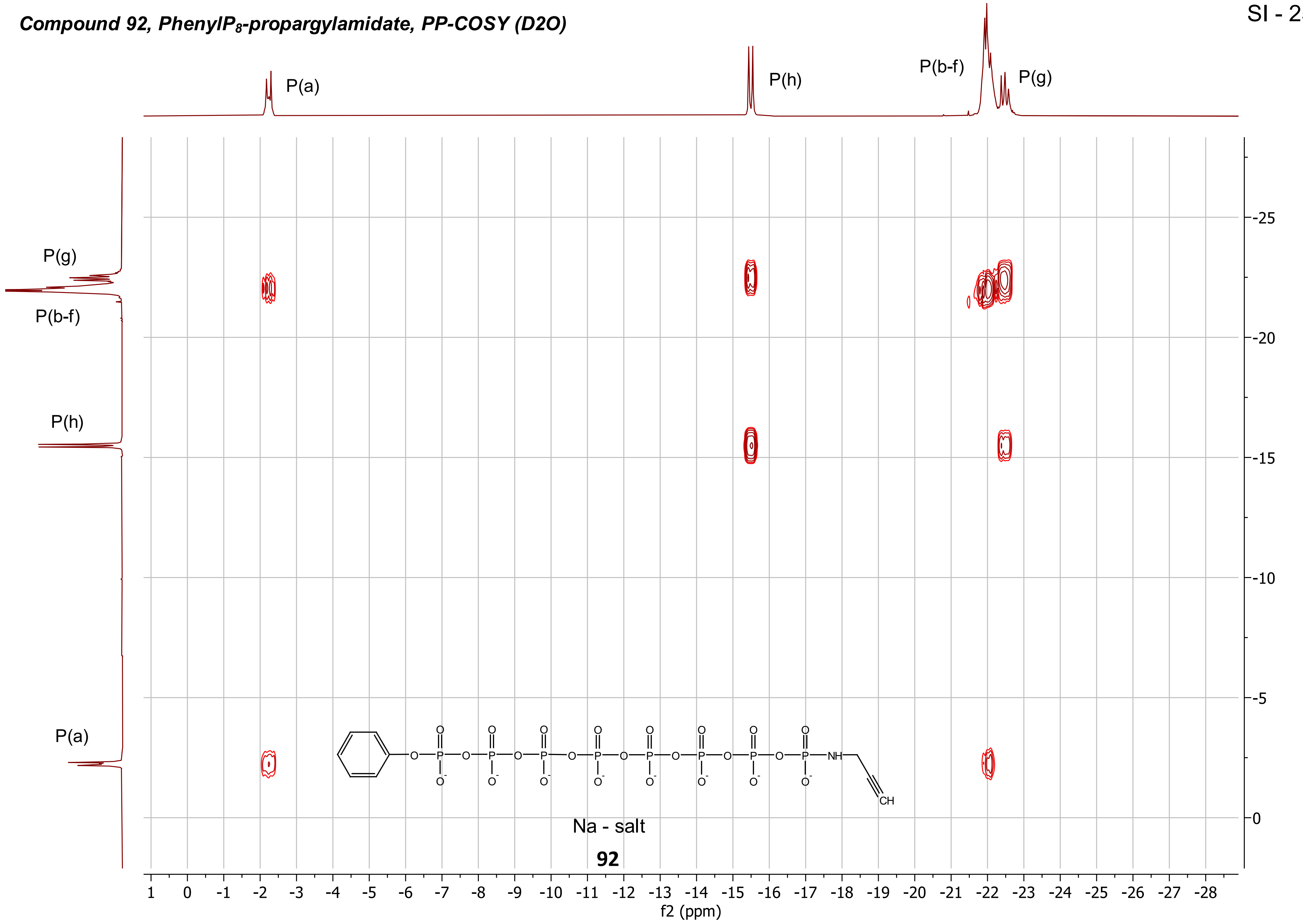

Compound 92, PhenylIP$_8$-propargylamidate, $^{13}C\{^1H\}$ - NMR (D2O, 101 MHz)

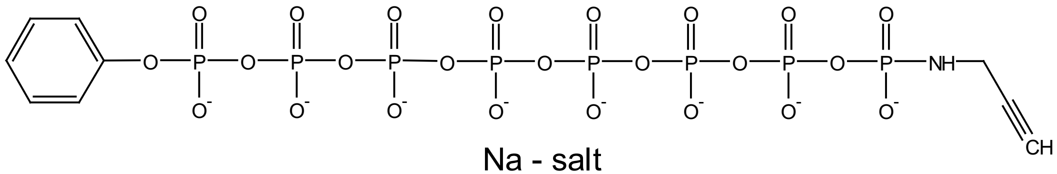

Na - salt

92

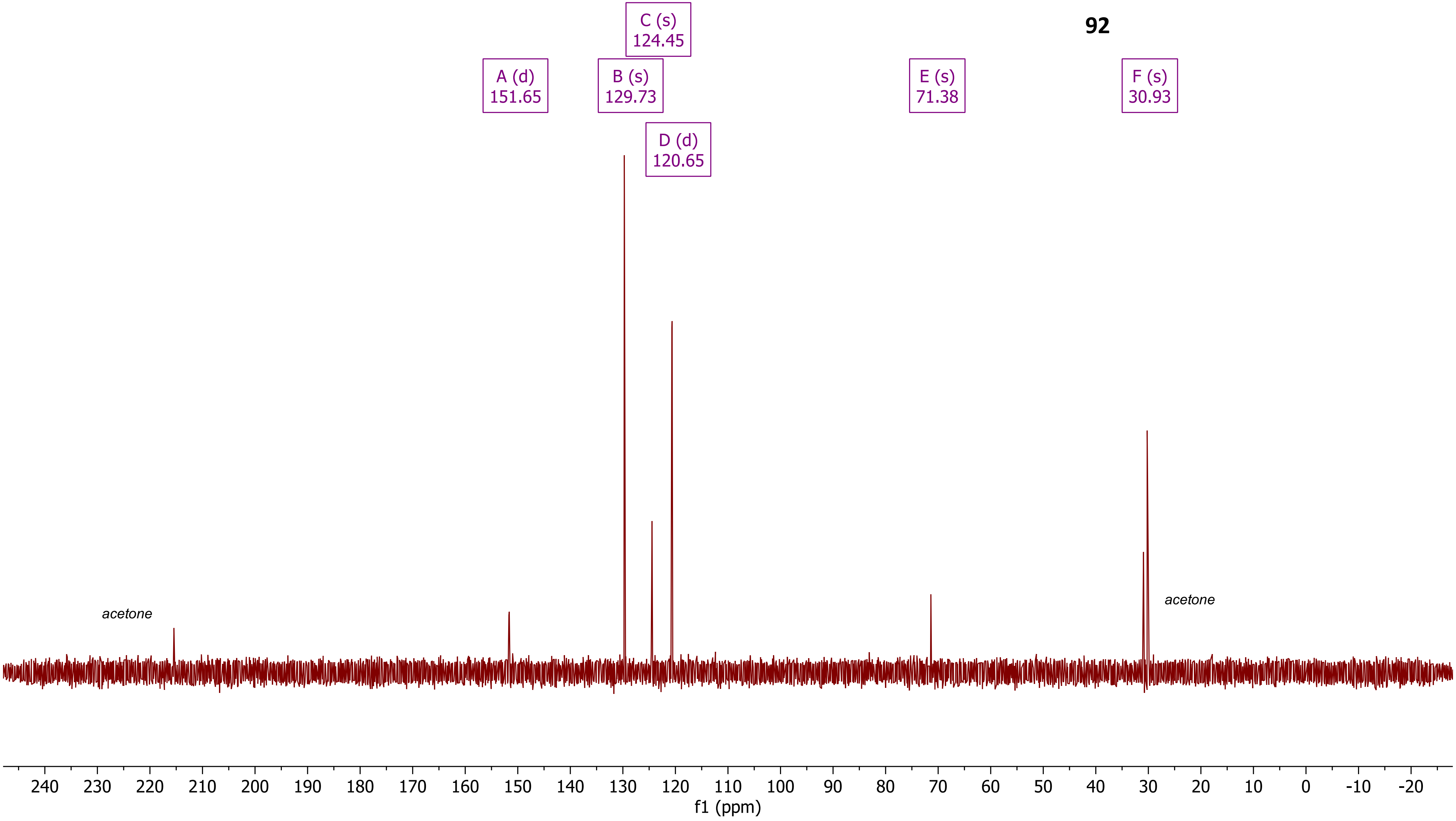

Compound SI-5, D4T-monophosphate, ¹H - NMR (D_2O, 400 MHz)

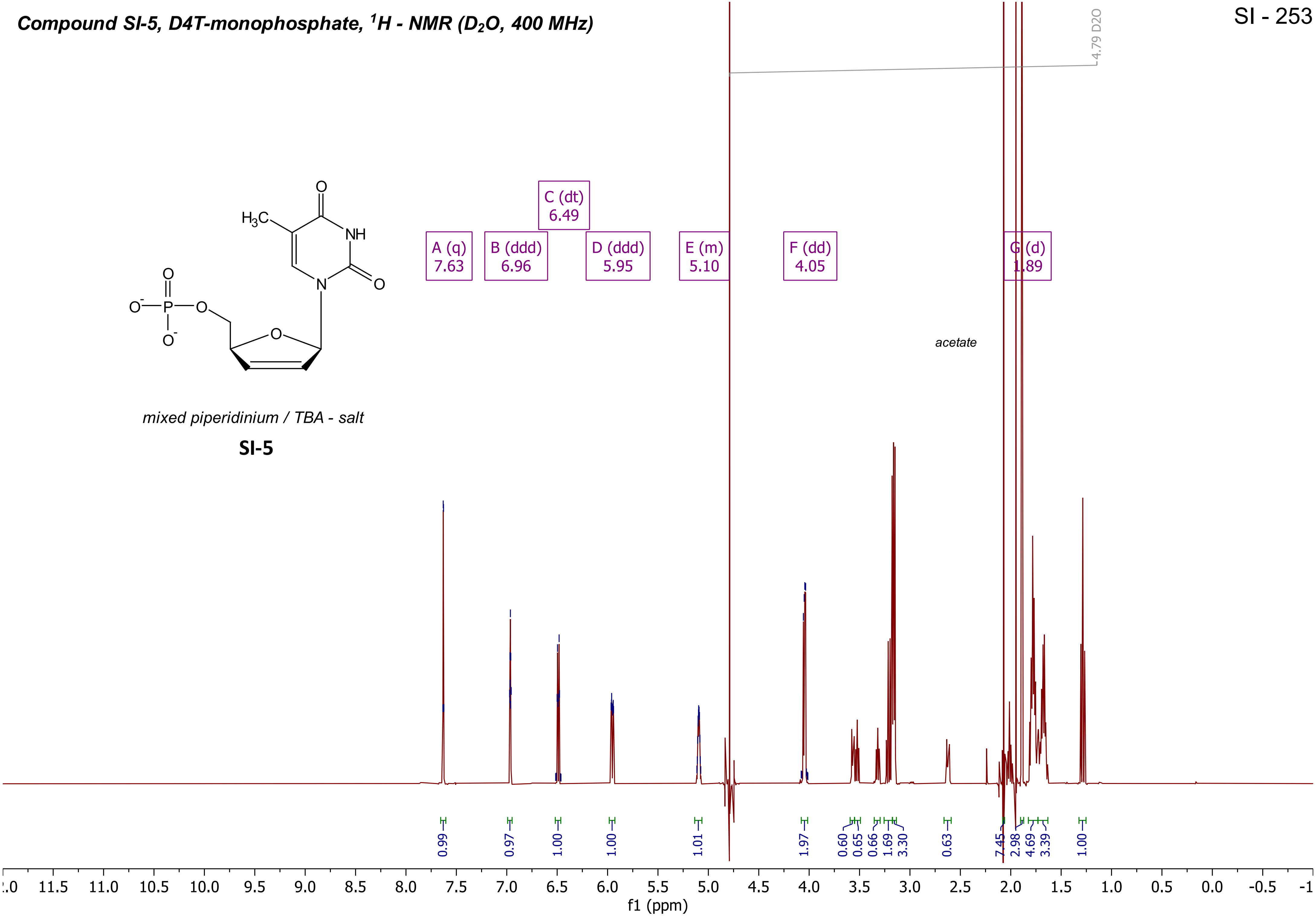

mixed piperidinium / TBA - salt

SI-5

Compound SI-5, D4T-monophosphate, $^{31}P\{^{1}H\}$ - NMR (D2O, 162 MHz)

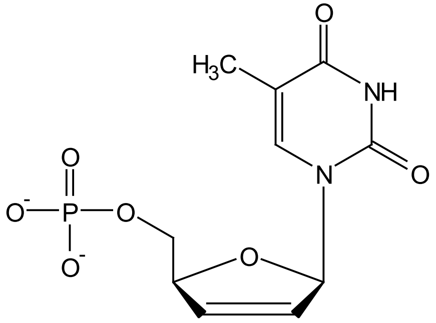

mixed piperidinium / TBA - salt

SI-5

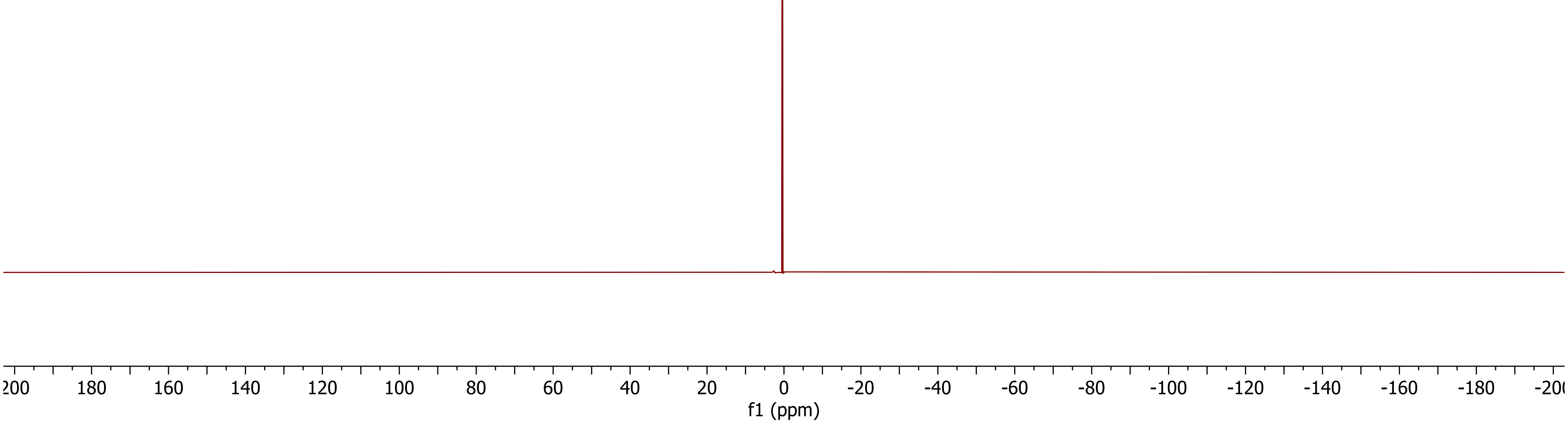

Compound SI-5, D4T-monophosphate, ^{31}P - NMR (D2O, 162 MHz)

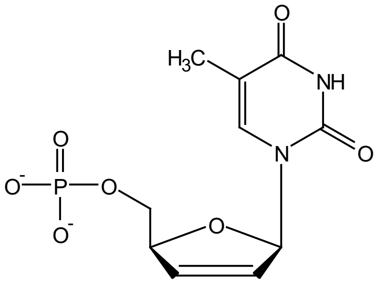

mixed piperidinium / TBA - salt

SI-5

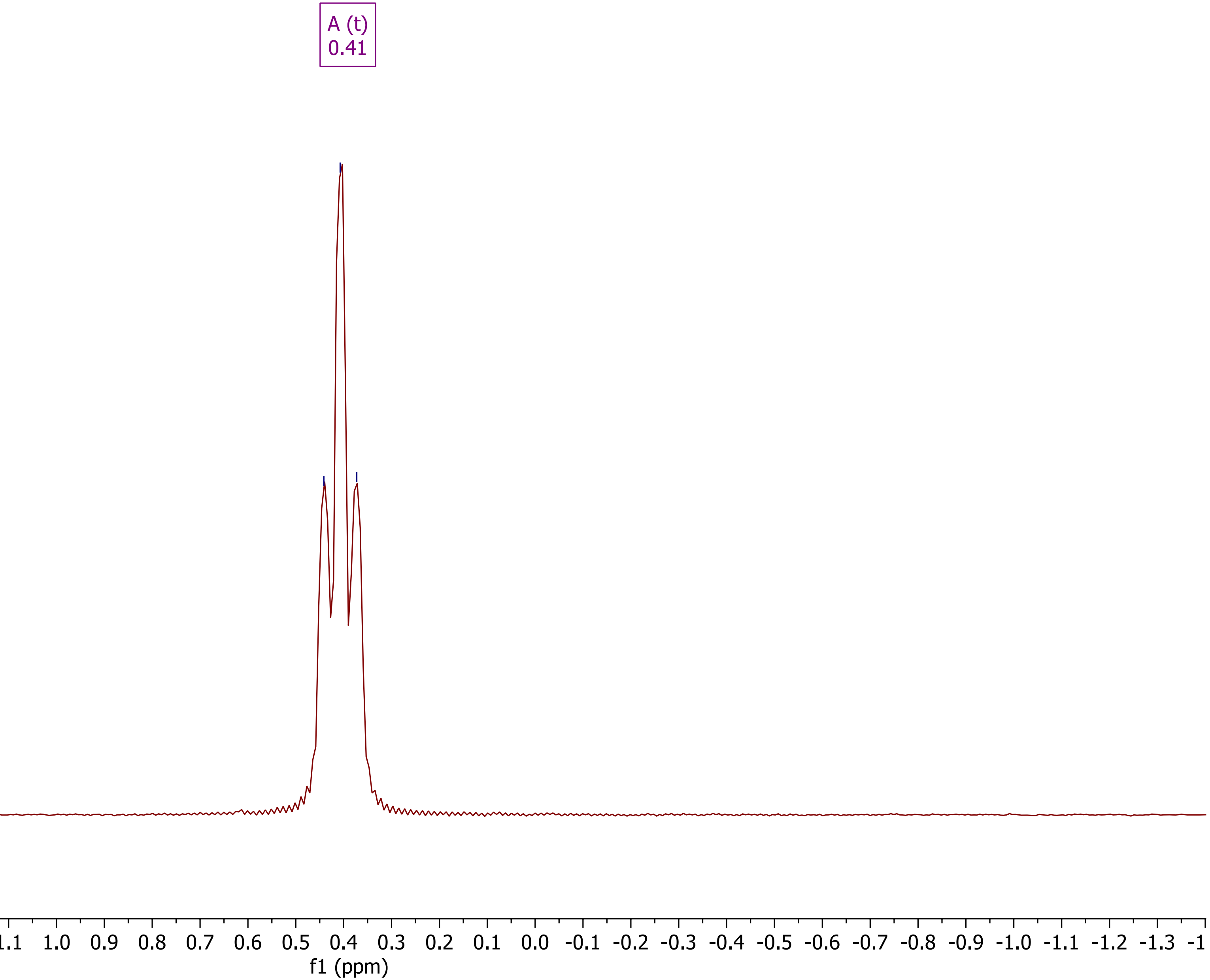

Compound SI-5, D4T-monophosphate, $^{13}C\{^1H\}$ - NMR (D2O, 101 MHz)

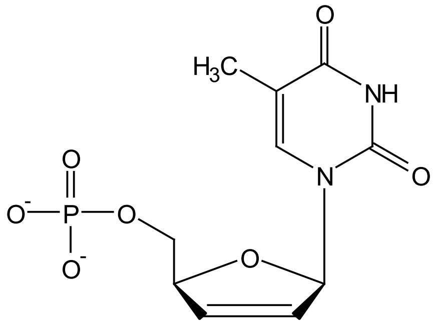

mixed piperidinium / TBA - salt

SI-5

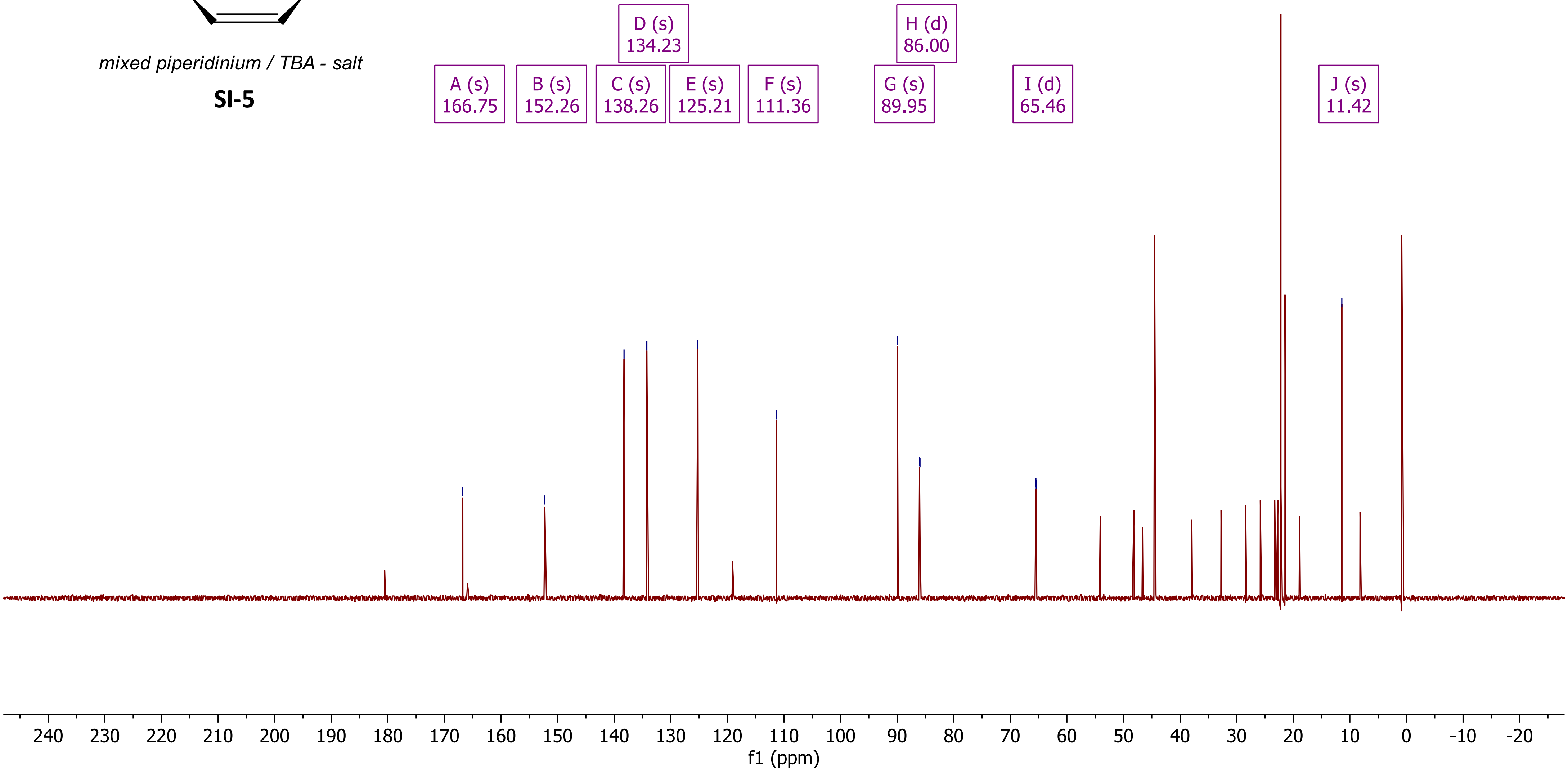

Compound SI-13, Hydroxyhexylphosphate, ^{1}H - NMR (D_2O, 400 MHz)

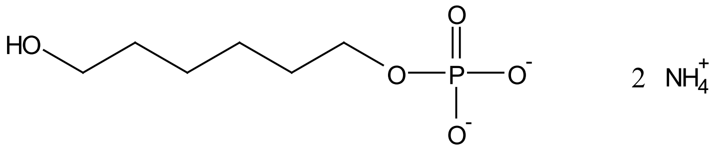

SI-13

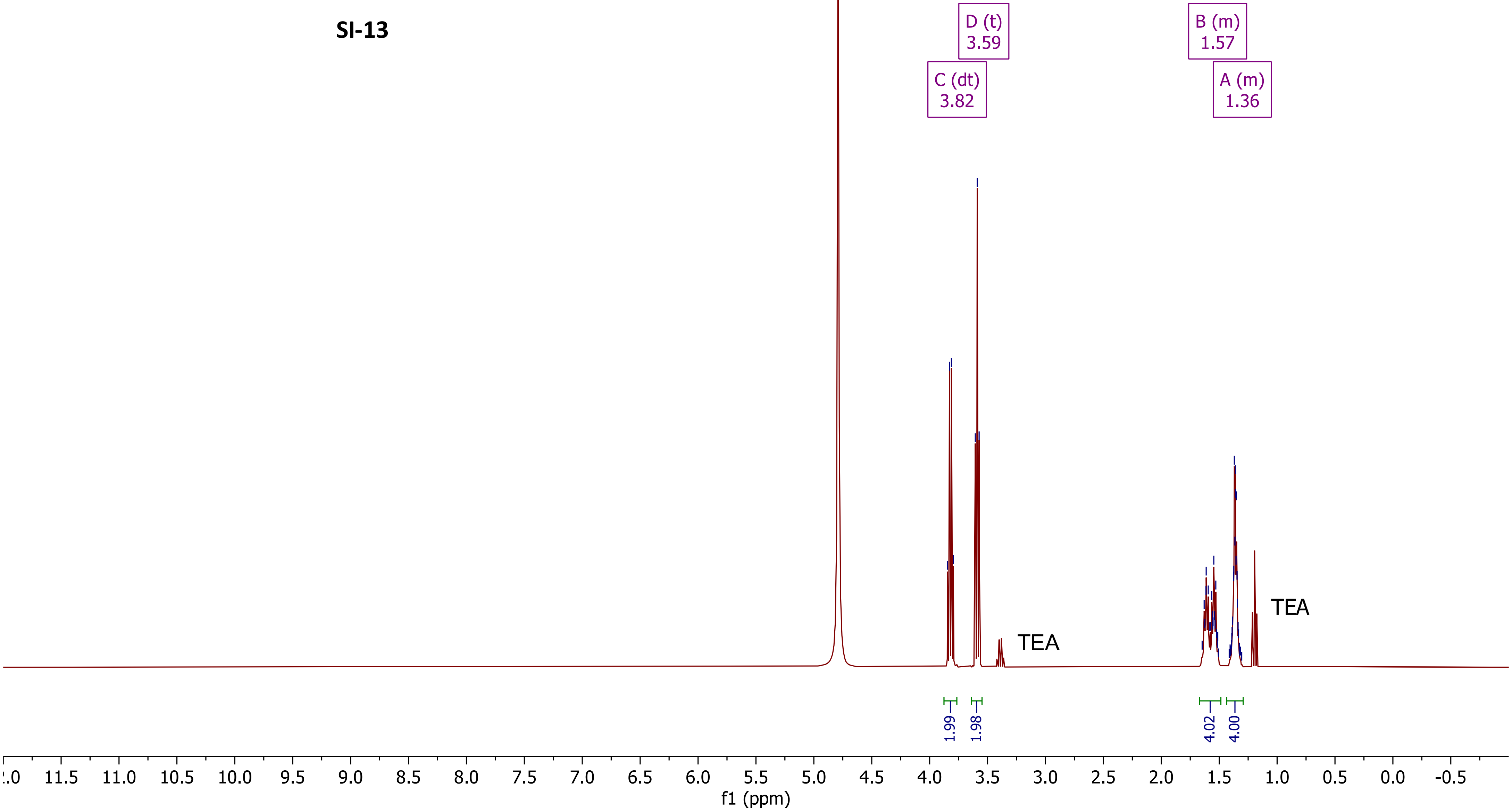

Compound SI-13, Hydroxyhexylphosphate, $^{31}P\ \{^{1}H\}$ - NMR (D2O, 162 MHz)

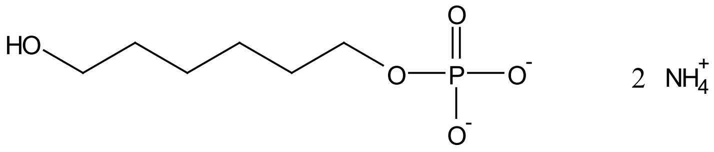

SI-13

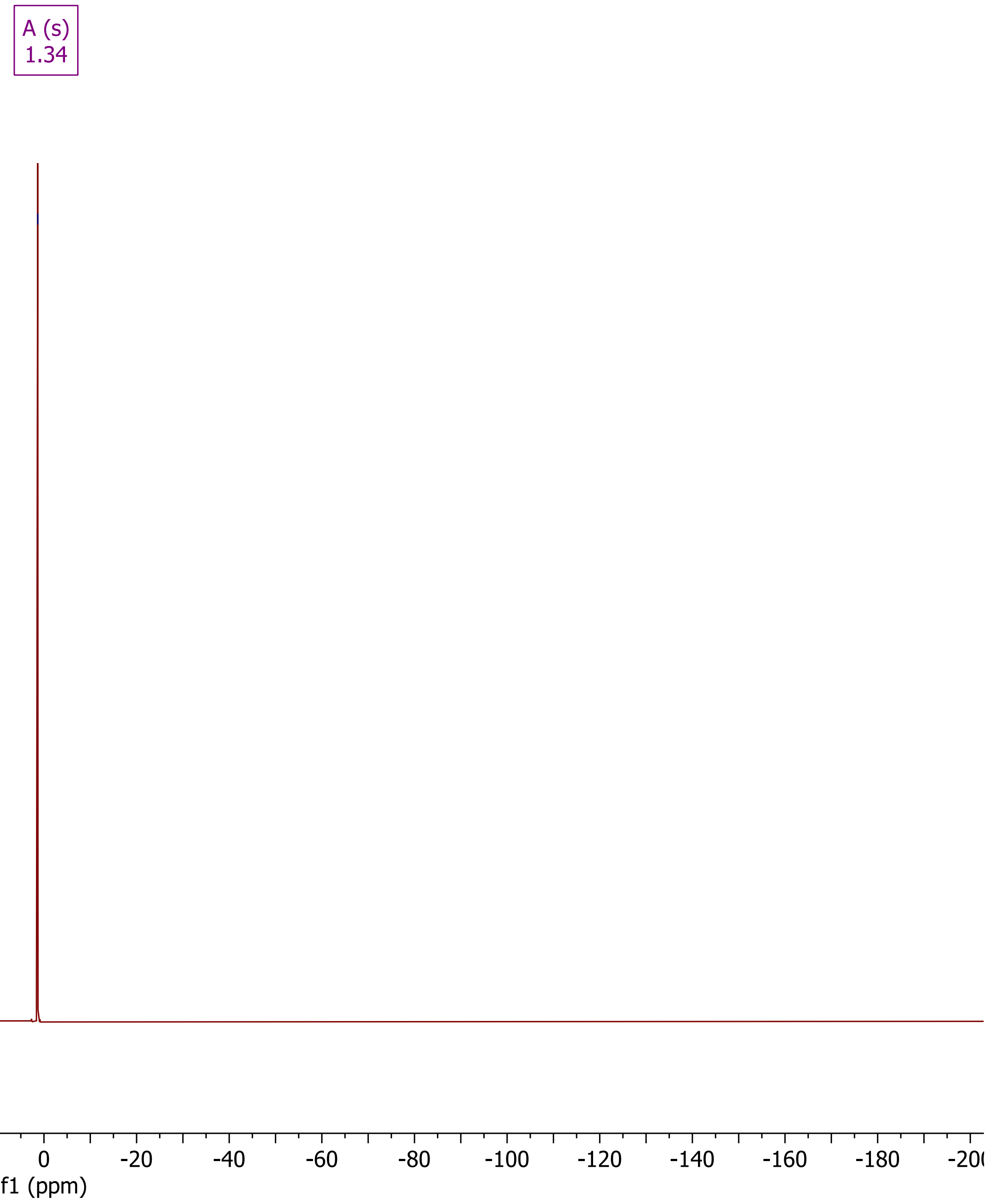

Compound SI-13, Hydroxyhexylphosphate, ^{31}P - NMR (D2O, 162 MHz)

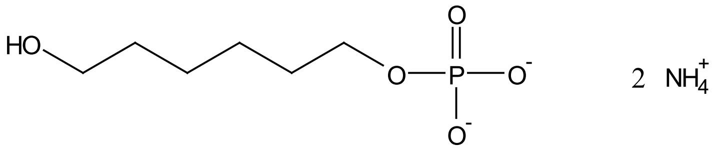

SI-13

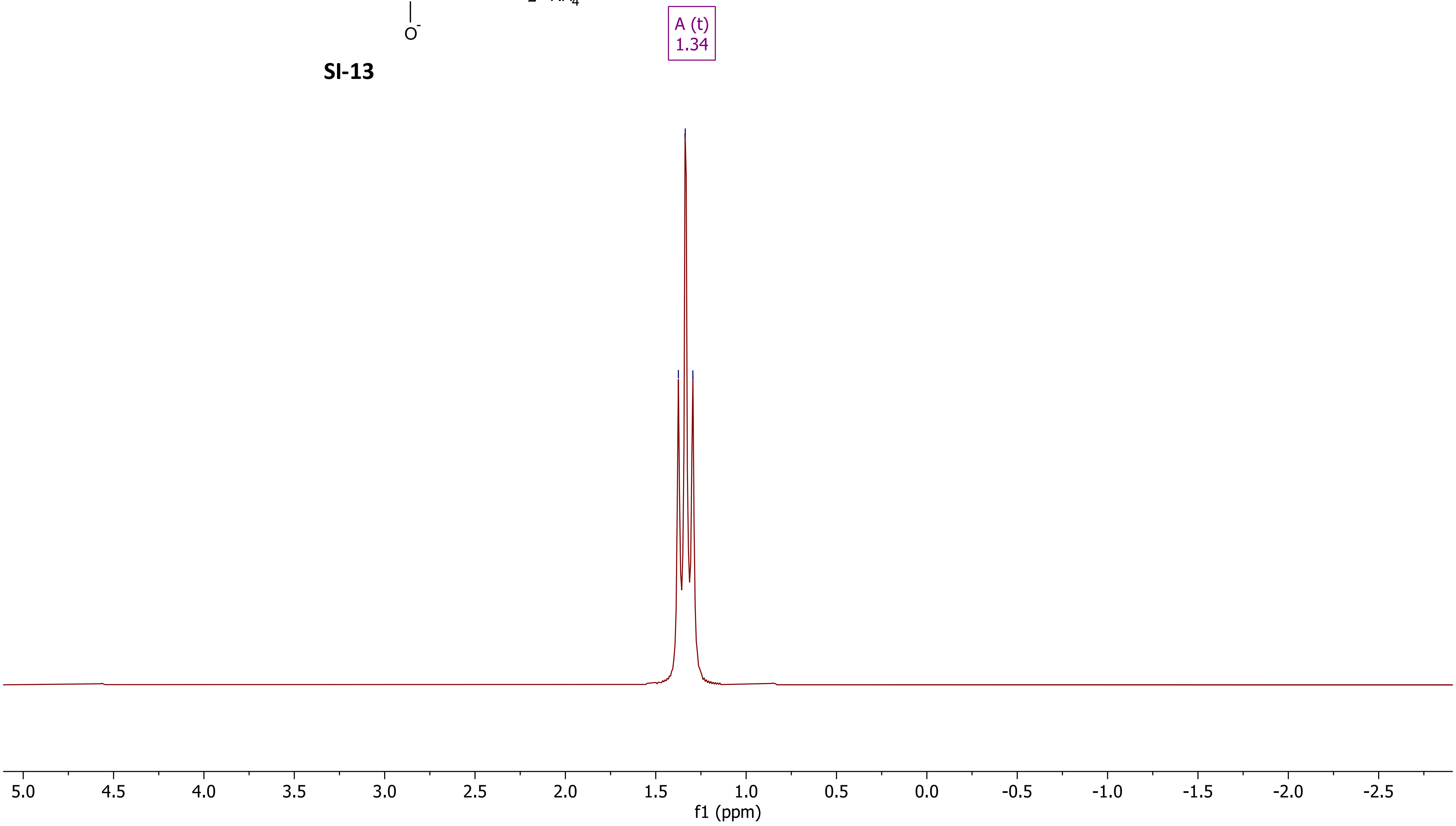

Compound SI-13, Hydroxyhexylphosphate, ^{13}C - NMR (D2O, 101 MHz)

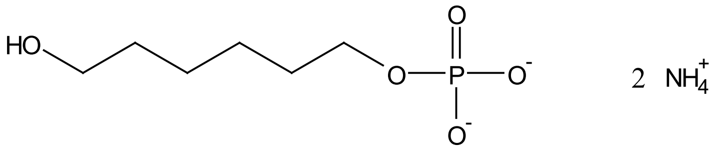

SI-13

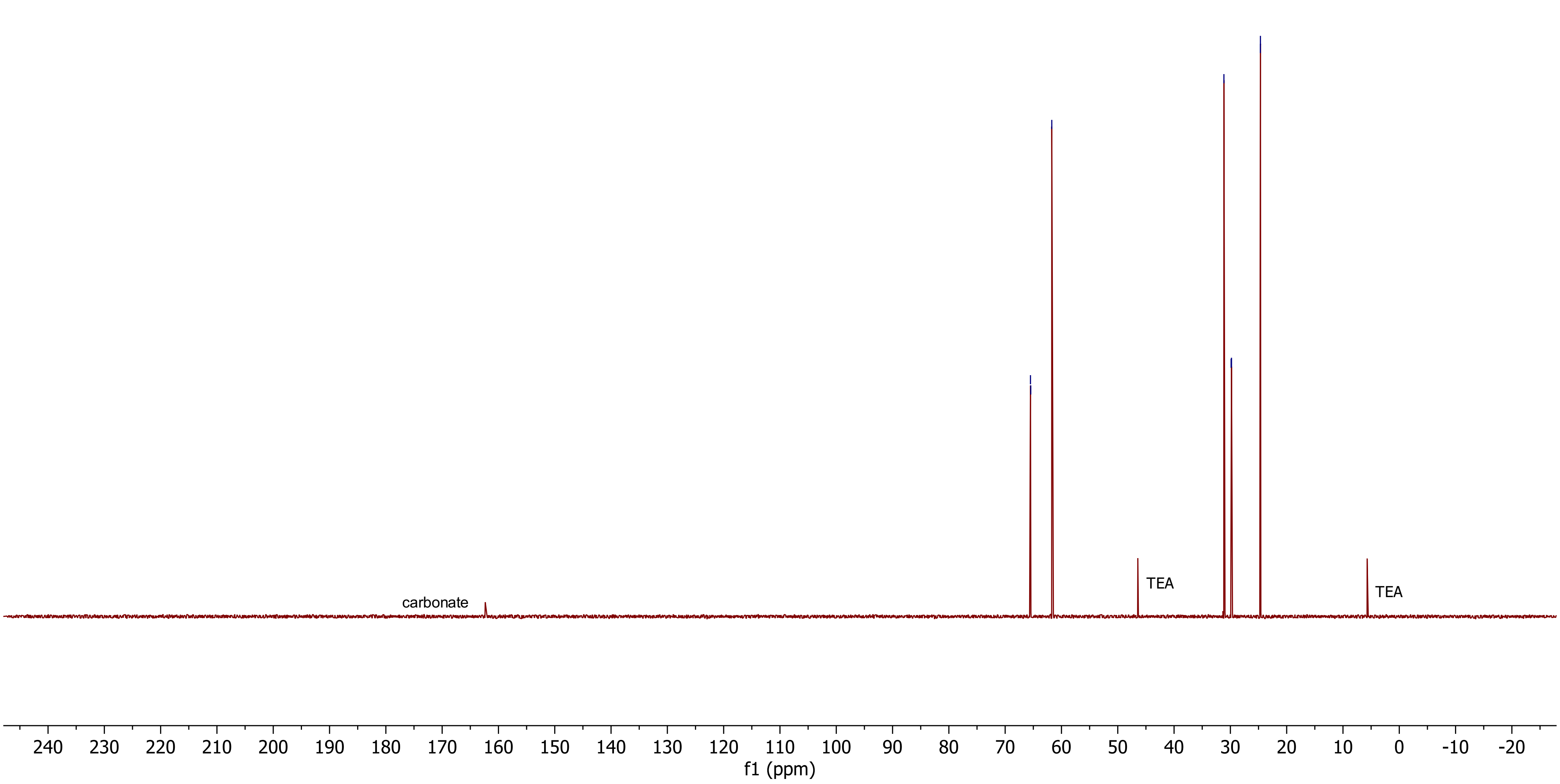

Compound SI-14, Geranylphosphate, ¹H - NMR (D_2O, 400 MHz)

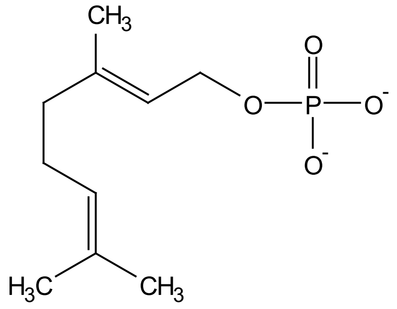

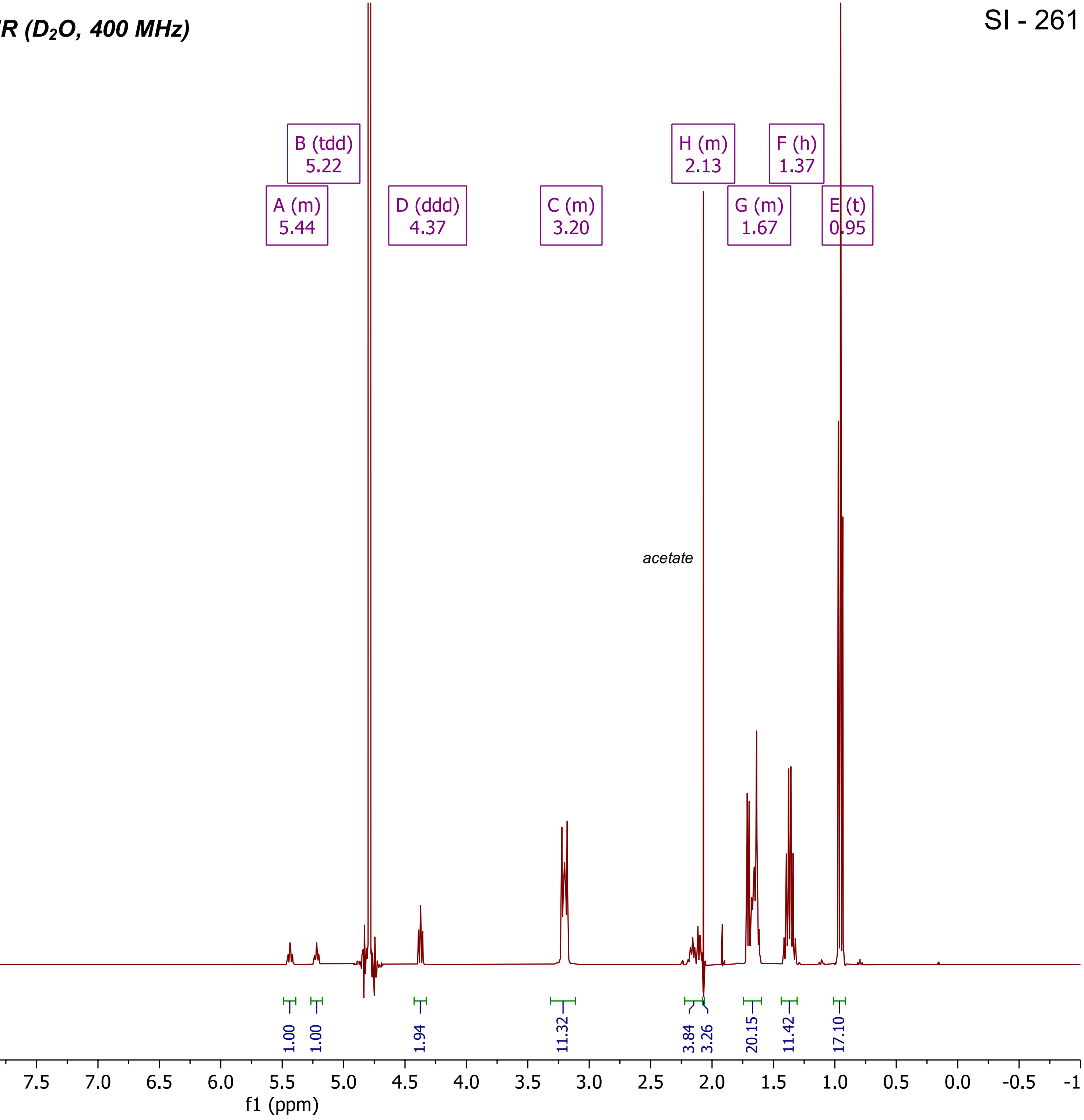

Compound SI-14, Geranylphosphate, $^{31}P\{^{1}H\}$ - NMR (D2O, 162 MHz)

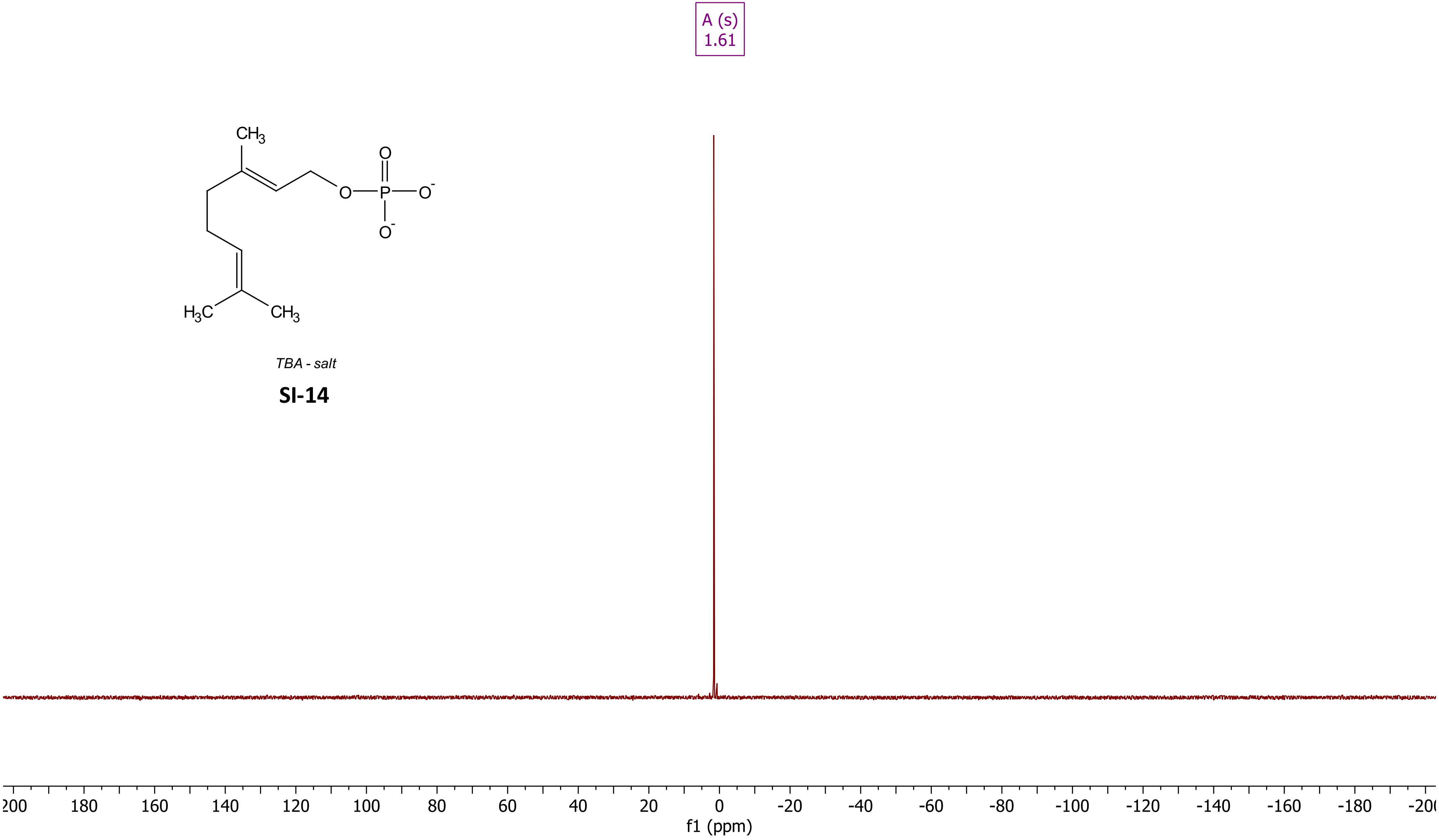

TBA - salt

SI-14

Compound SI-14, Geranylphosphate, $^{13}C\{^1H\}$ - NMR (D2O, 101 MHz)

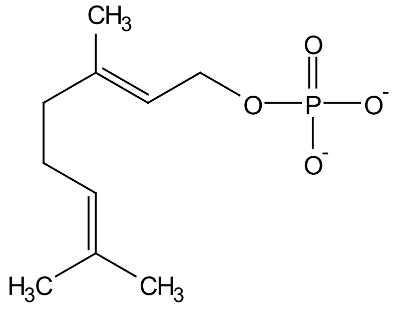

TBA - salt

SI-14

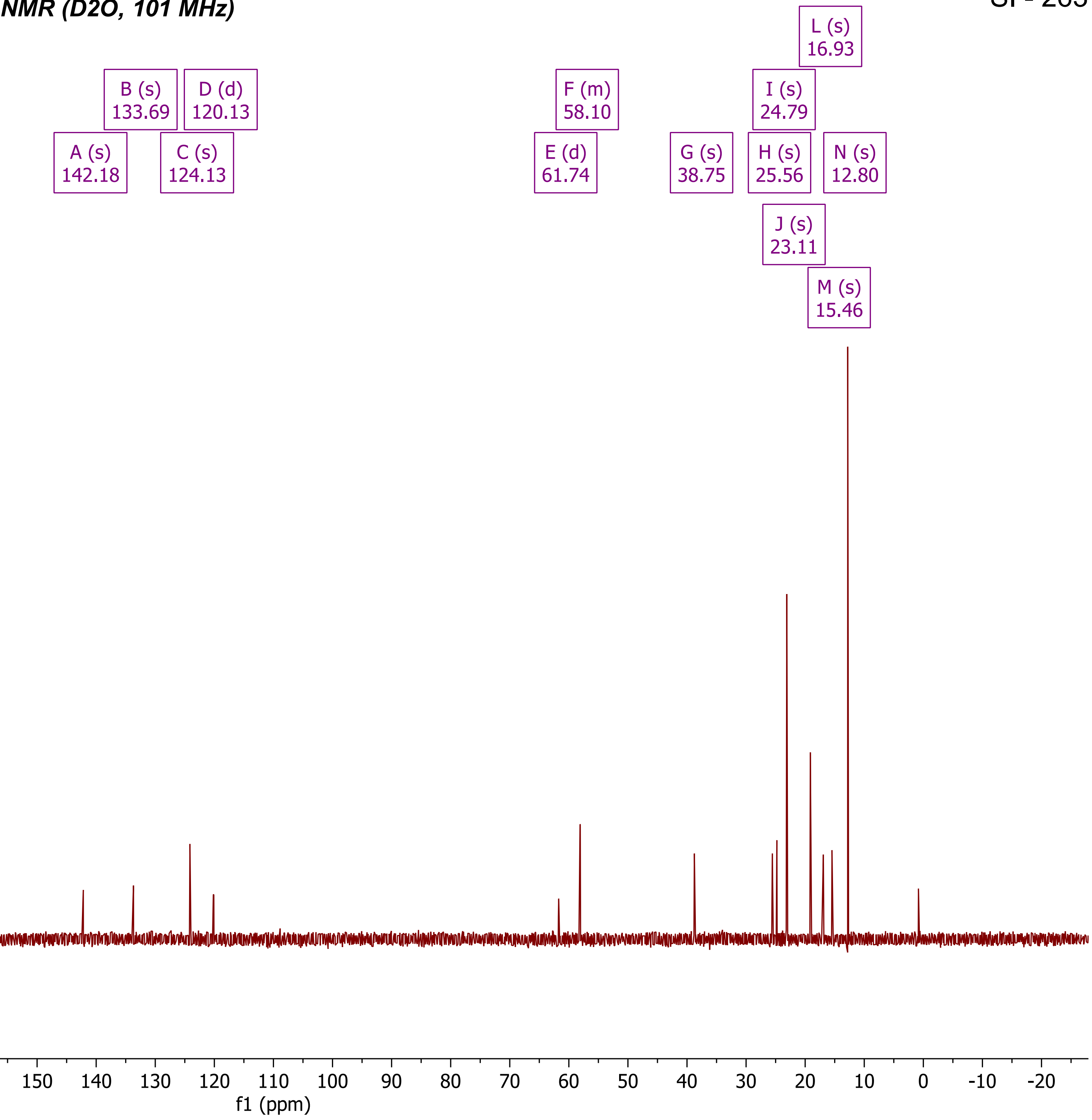

Compound SI-15, Isoprenylphosphate, ^{1}H - NMR (D_2O, 400 MHz)

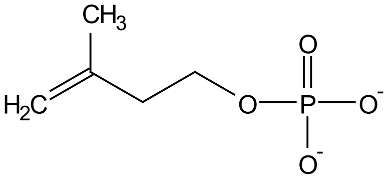

piperidinium/ TEAA salt

SI-15

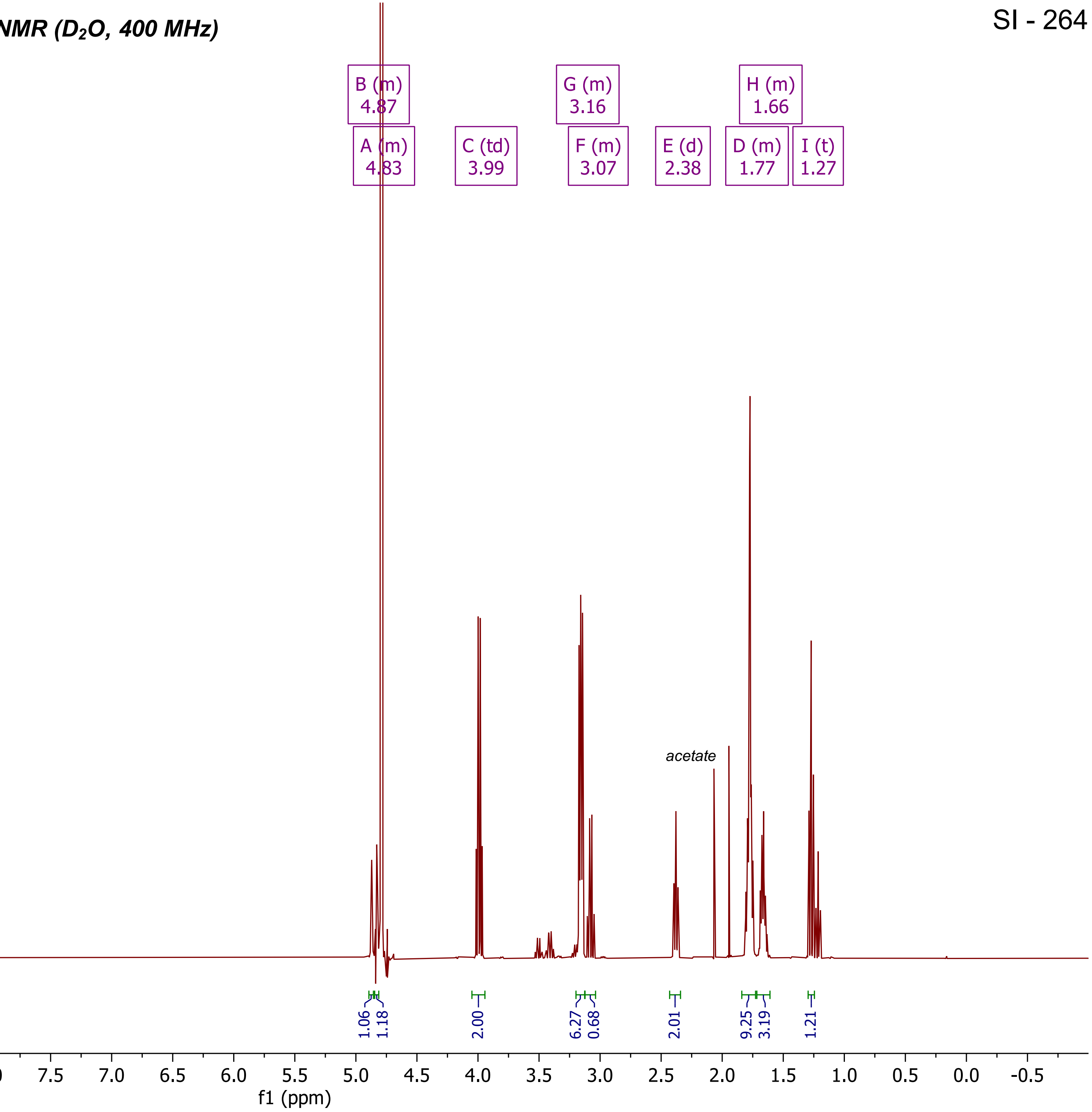

Compound SI-15, Isoprenylphosphate, $^{31}P\{^{1}H\}$ - NMR (D2O, 162 MHz)

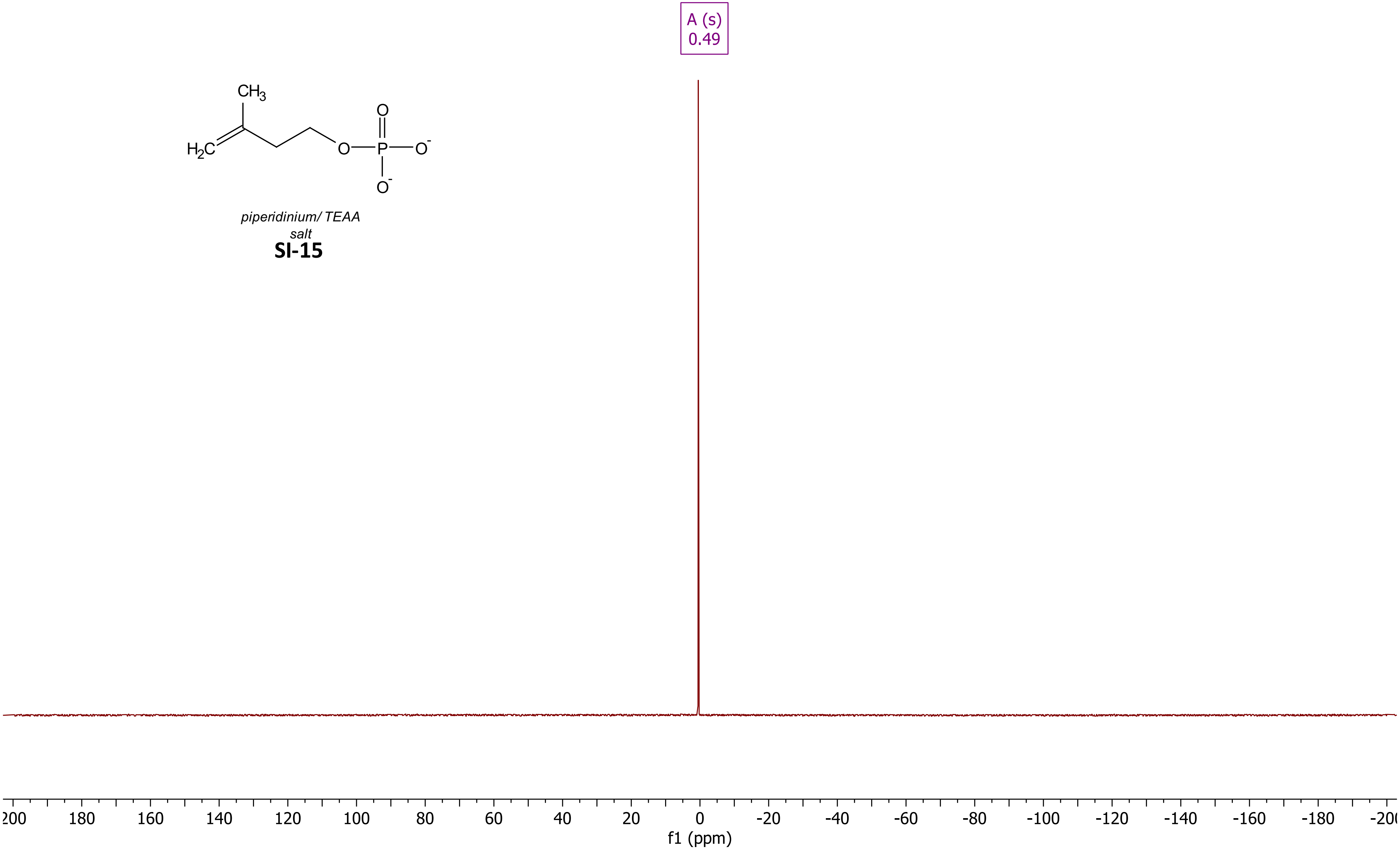

Compound SI-15, Isoprenylphosphate, ^{31}P - NMR (D2O, 162 MHz)

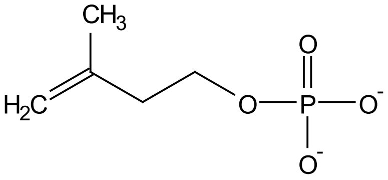

piperidinium/ TEAA salt

SI-15

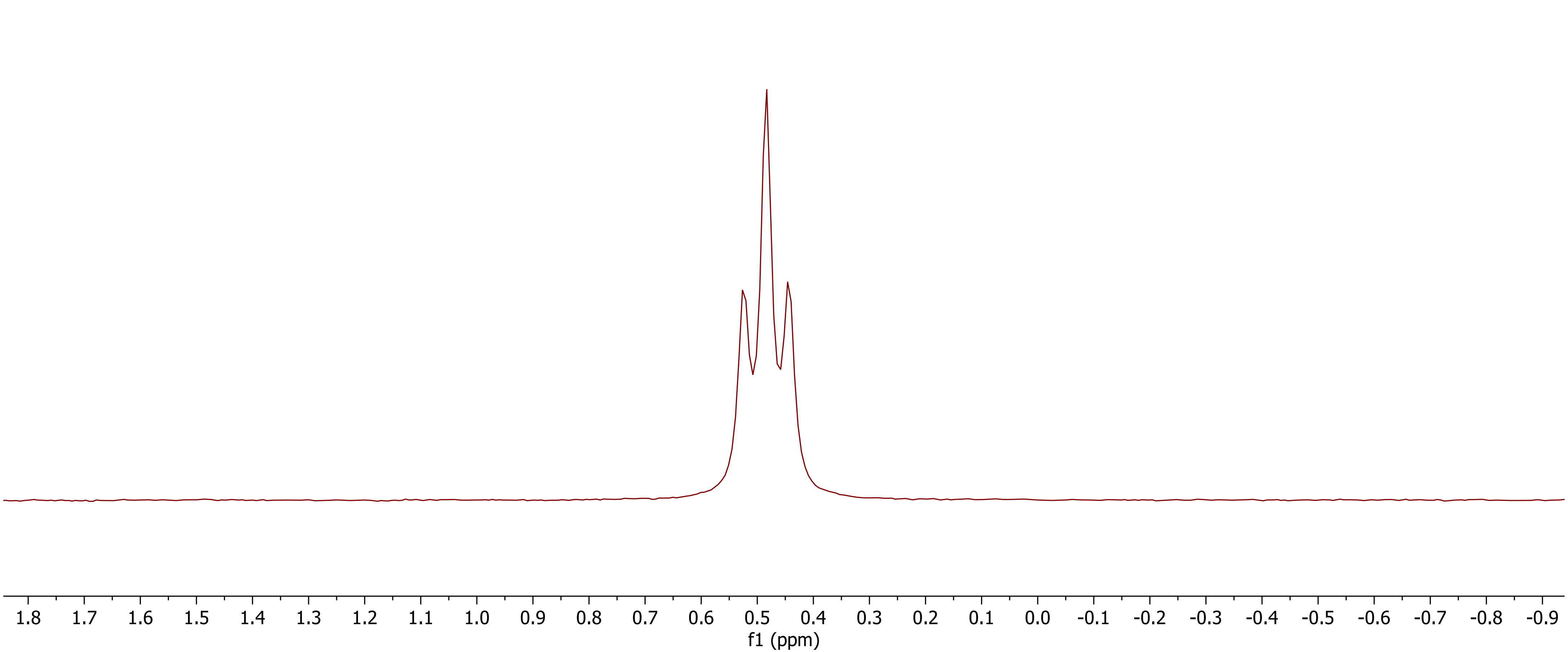

Compound SI-15, Isoprenylphosphate, $^{13}C\{^{1}H\}$ - NMR (D2O, 101 MHz)

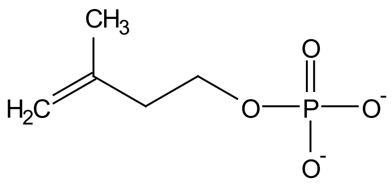

piperidinium/ TEAA salt
SI-15

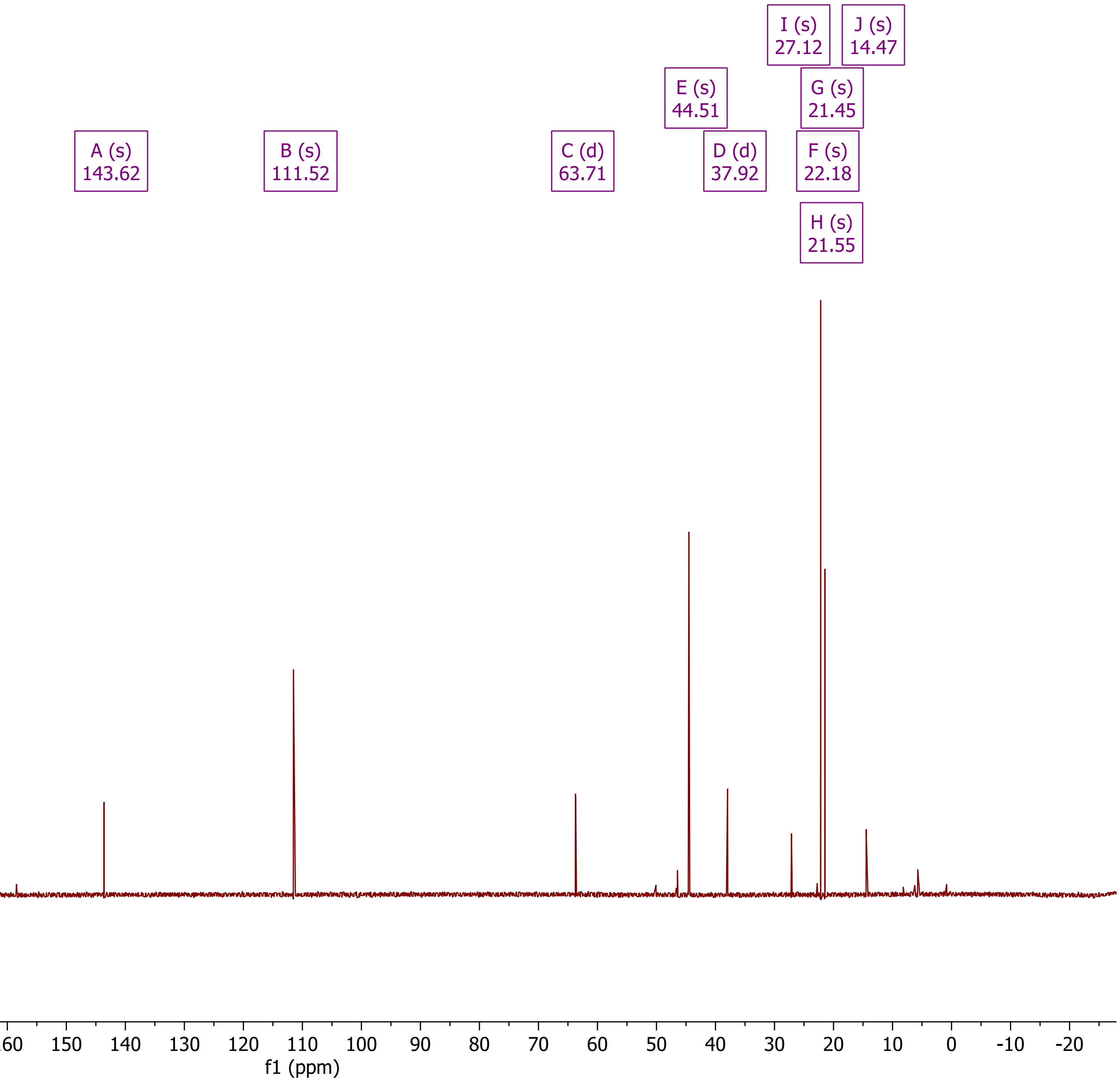

Compound SI-16, Phenyldiphosphate, ^{1}H - NMR (D_2O, 400 MHz)

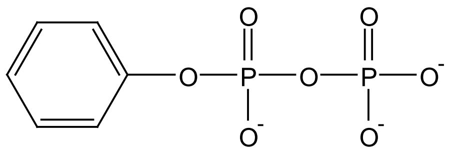

piperidinium salt

SI-16

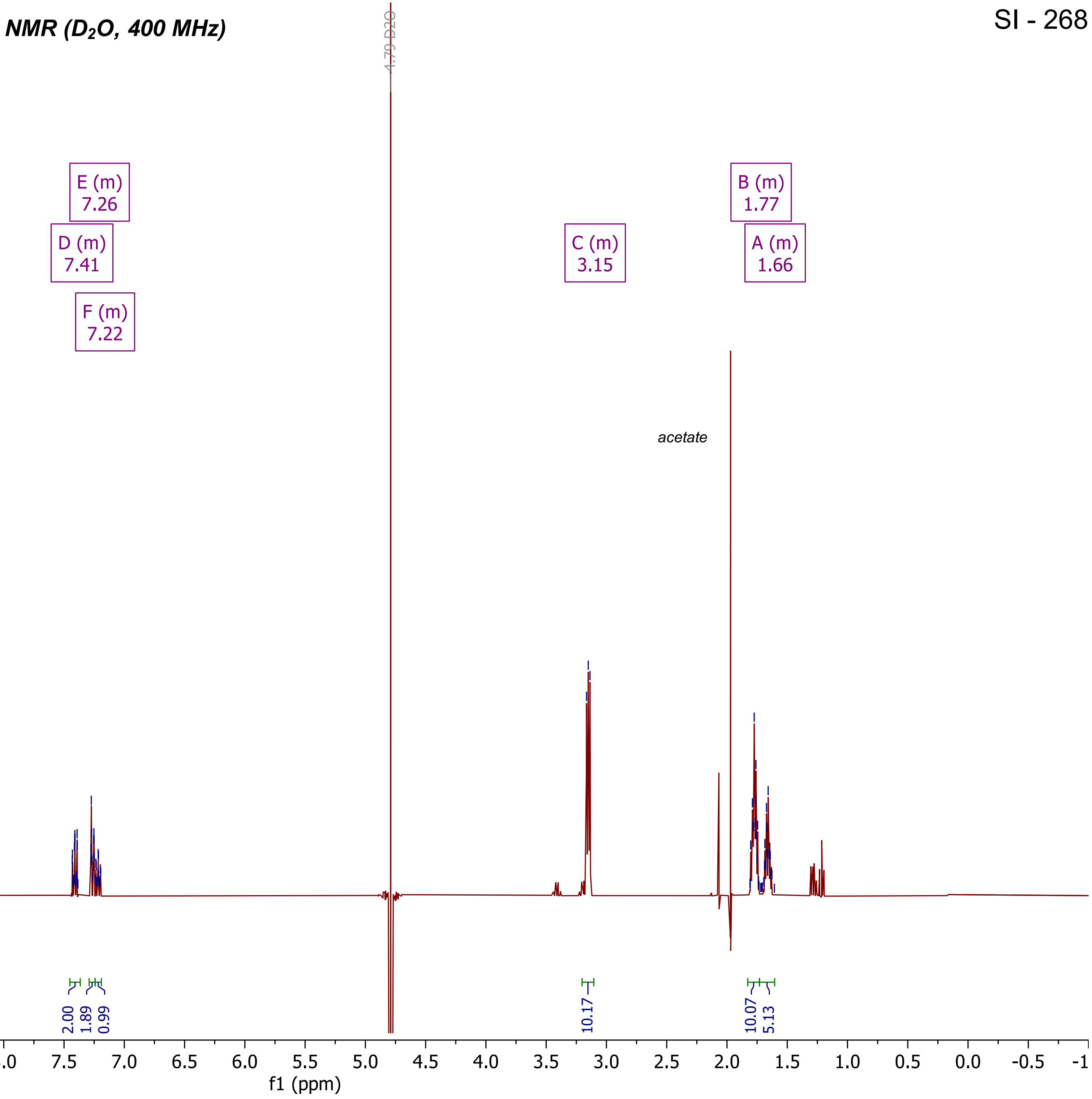

Compound SI-16, Phenyldiphosphate, $^{31}P\{^{1}H\}$ - NMR (D2O, 162 MHz)

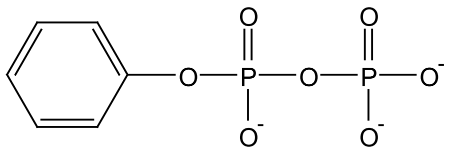

piperidinium salt

SI-16

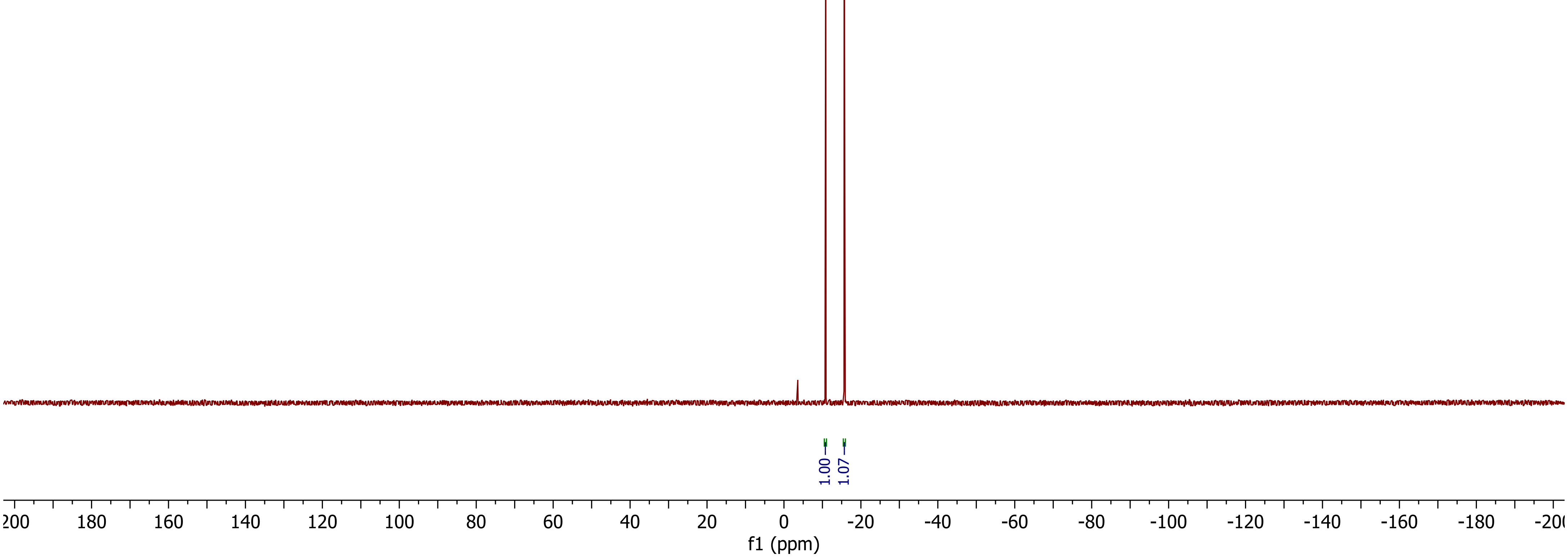

Compound SI-16, Phenyldiphosphate, $^{13}C\{^1H\}$ - NMR (D2O, 101 MHz)

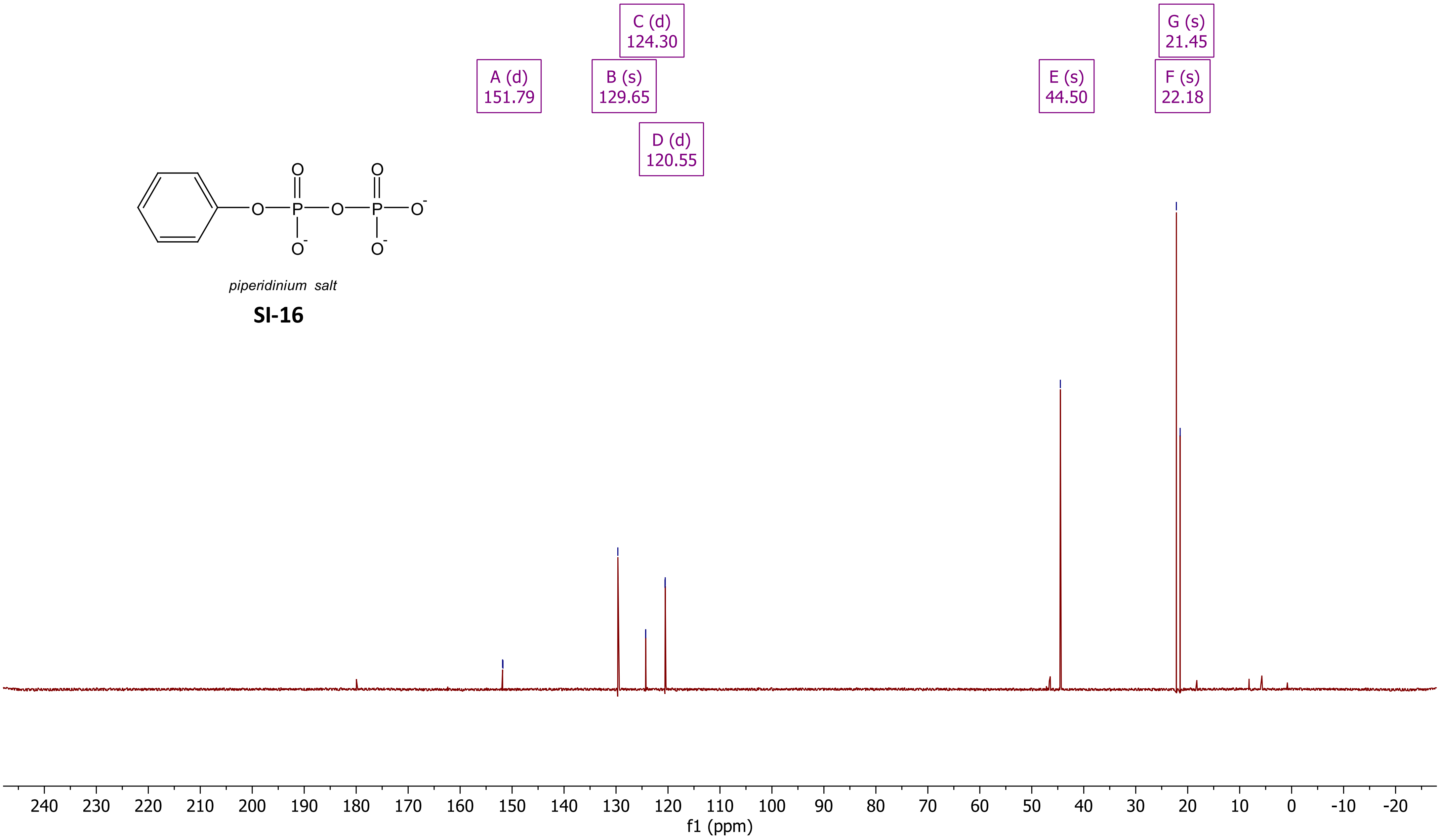

Compound SI-17, D4T-diphosphate, ^1H - NMR (D_2O, 400 MHz)

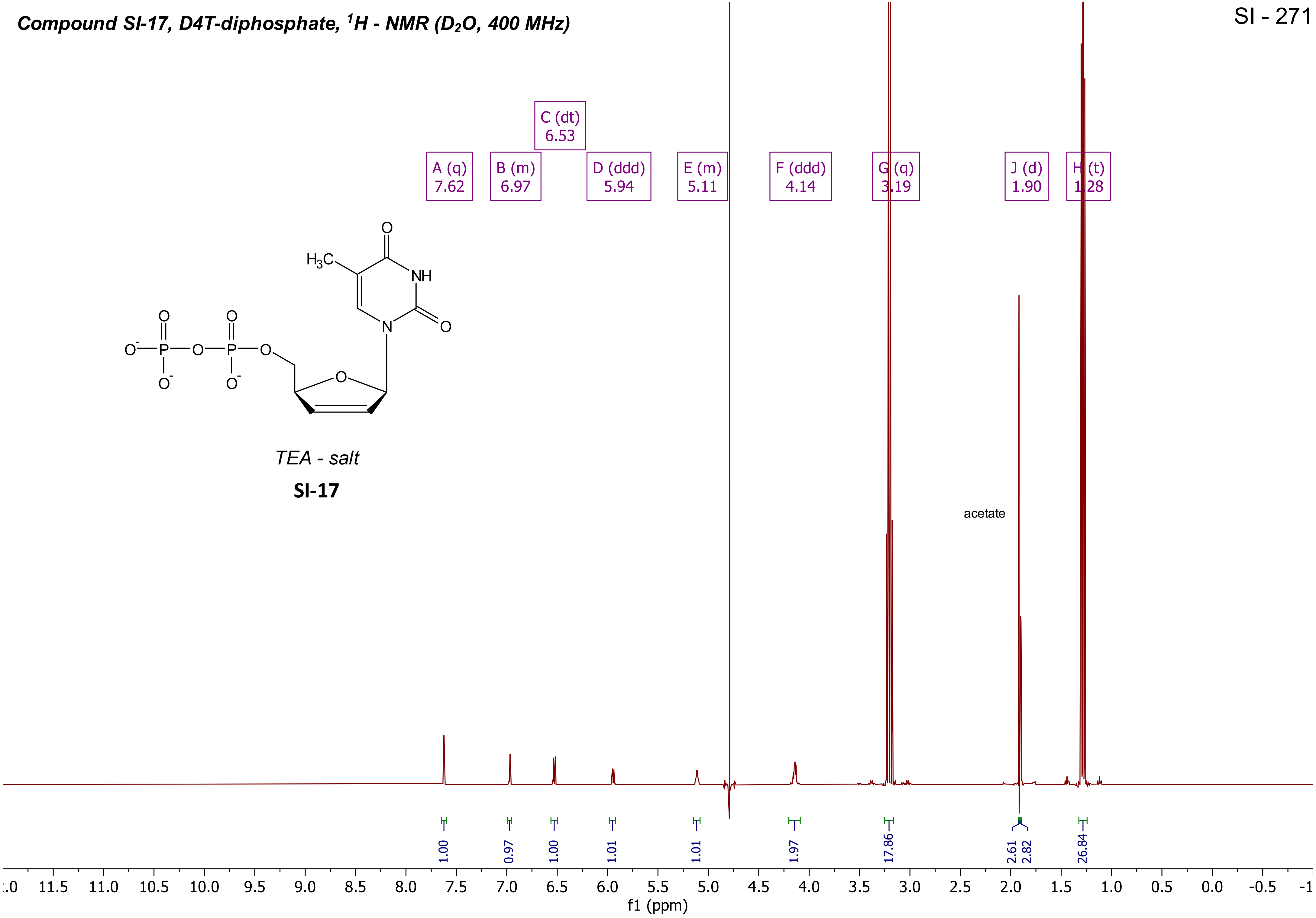

Compound SI-17, D4T-diphosphate, $^{31}P\{^{1}H\}$ - NMR (D2O, 162 MHz)

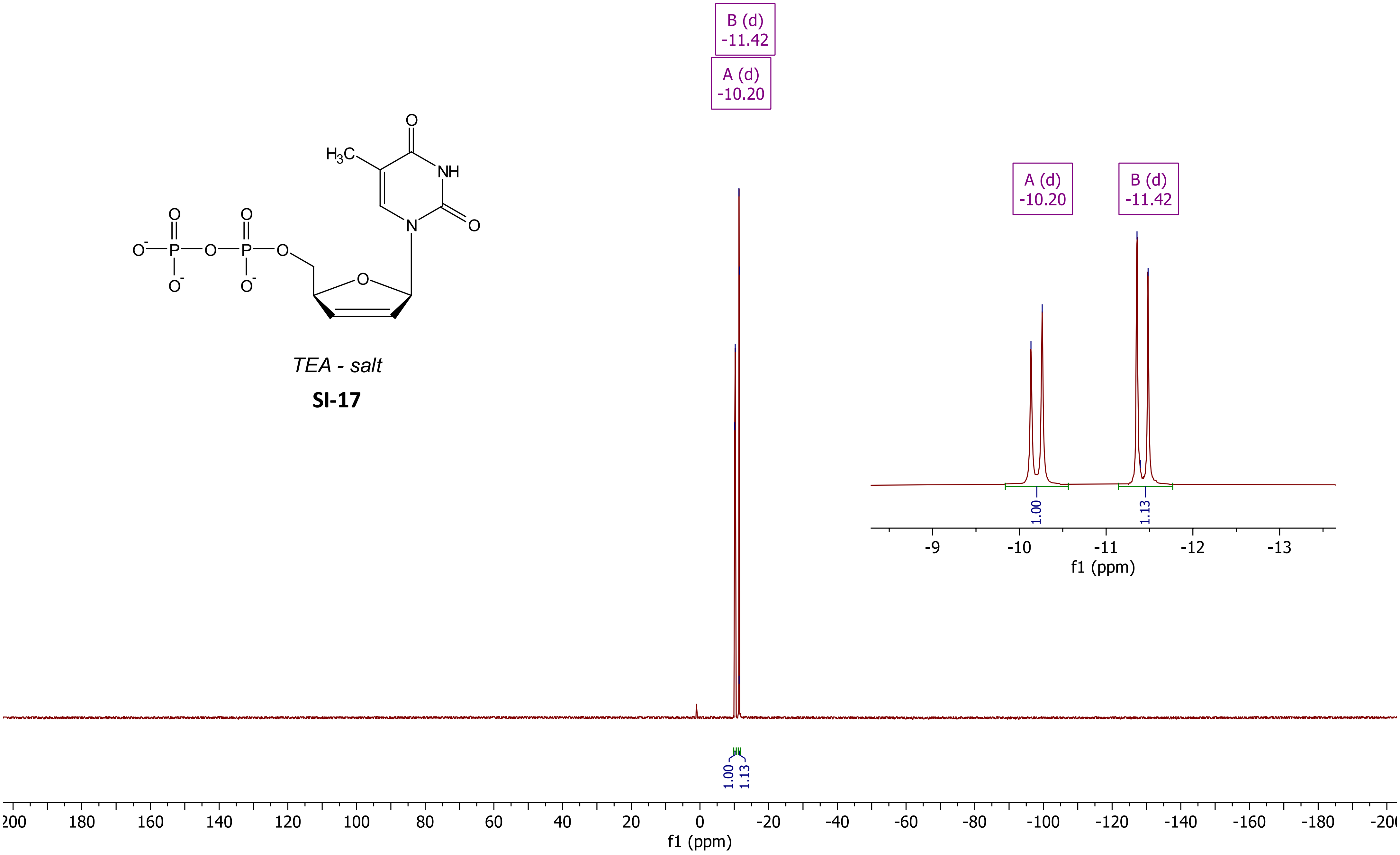

Compound SI-17, D4T-diphosphate, ^{31}P - NMR (D2O, 162 MHz)

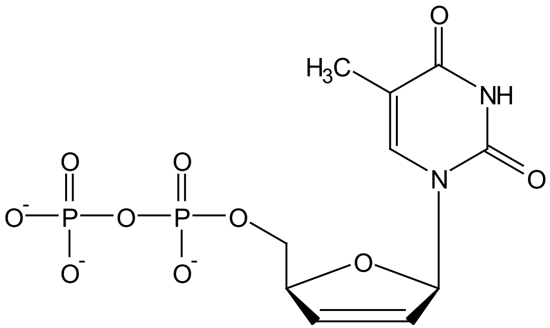

TEA - salt

SI-17

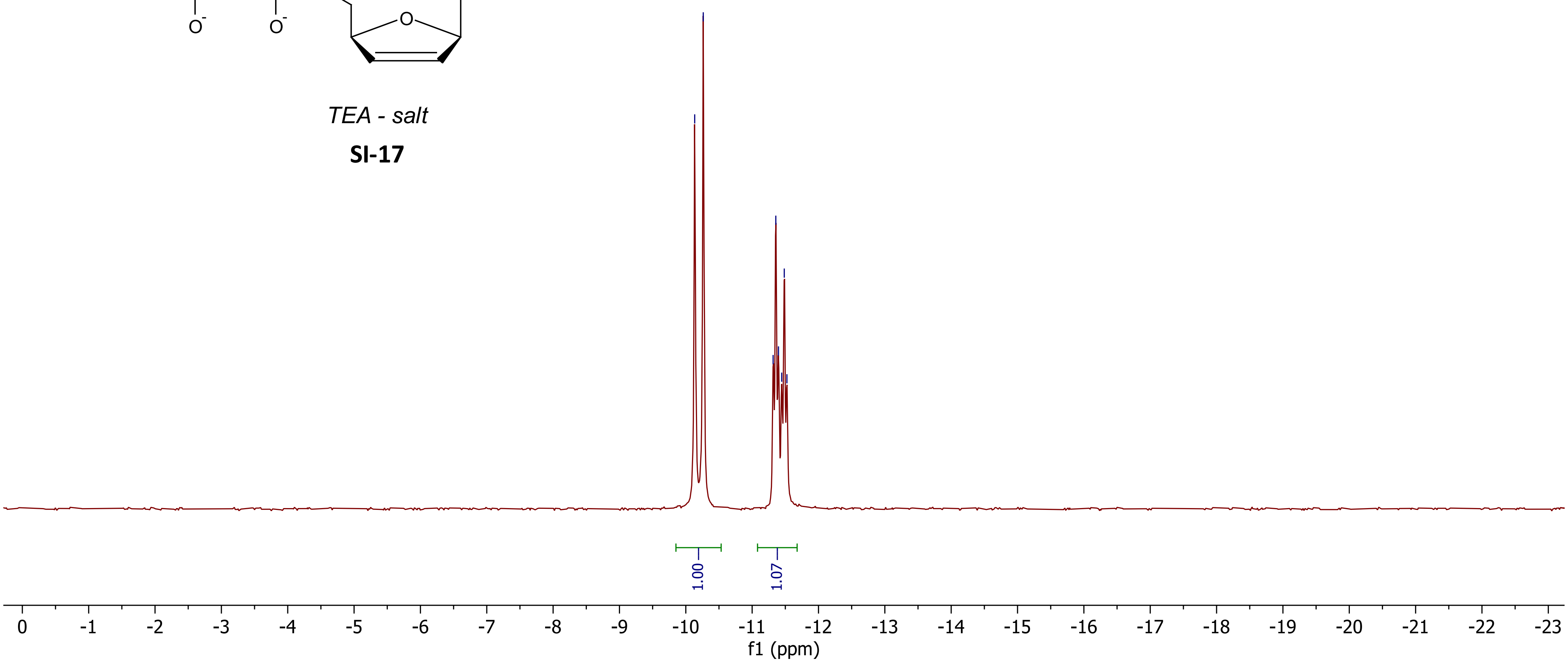

Compound SI-17, D4T-diphosphate, $^{13}C\{^1H\}$ - NMR (D2O, 101 MHz)

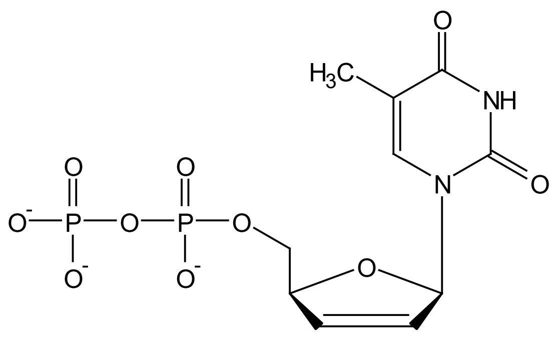

TEA - salt

SI-17

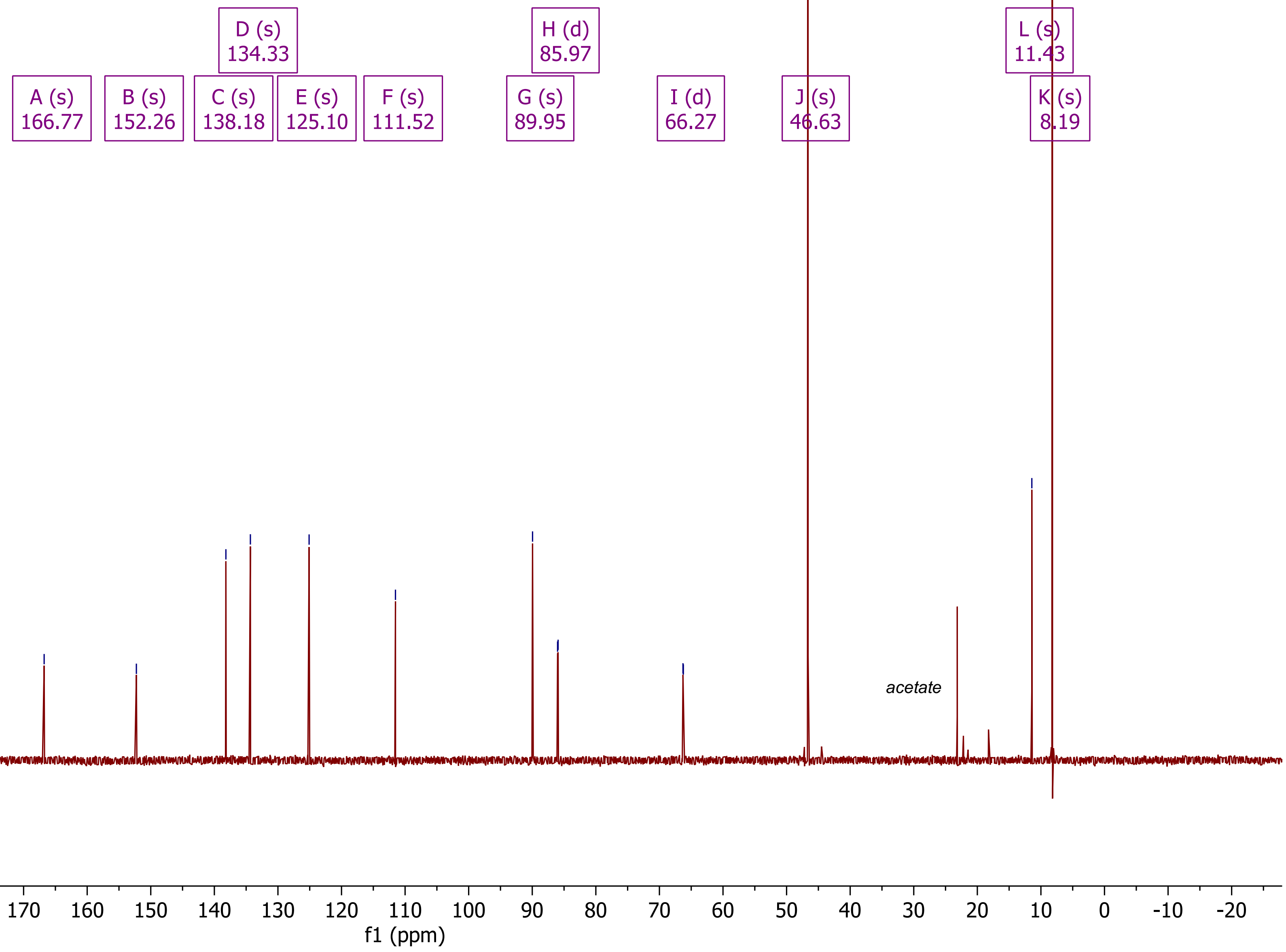

Compound SI-19, 2-(trimethylsilyl)pyren-1-yl trifluoromethanesulfonate, ^{1}H - NMR ($CDCl_3$, 400 MHz)

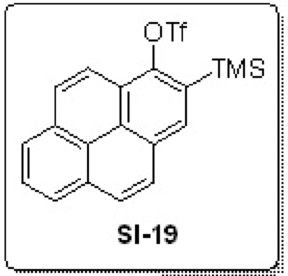

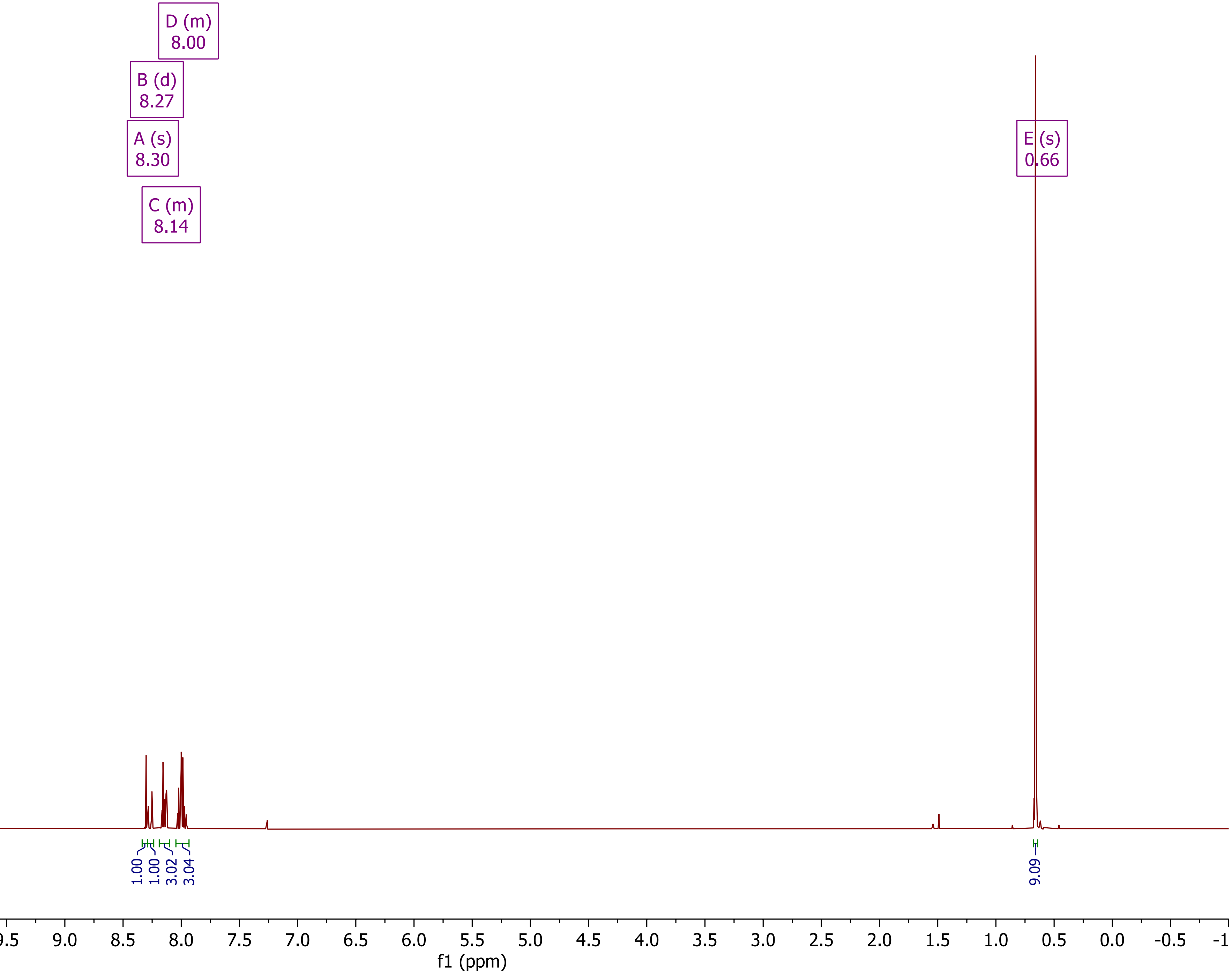

Compound SI-19, 2-(trimethylsilyl)pyren-1-yl trifluoromethanesulfonate, ^{19}F - NMR ($CDCl_3$, 377 MHz)

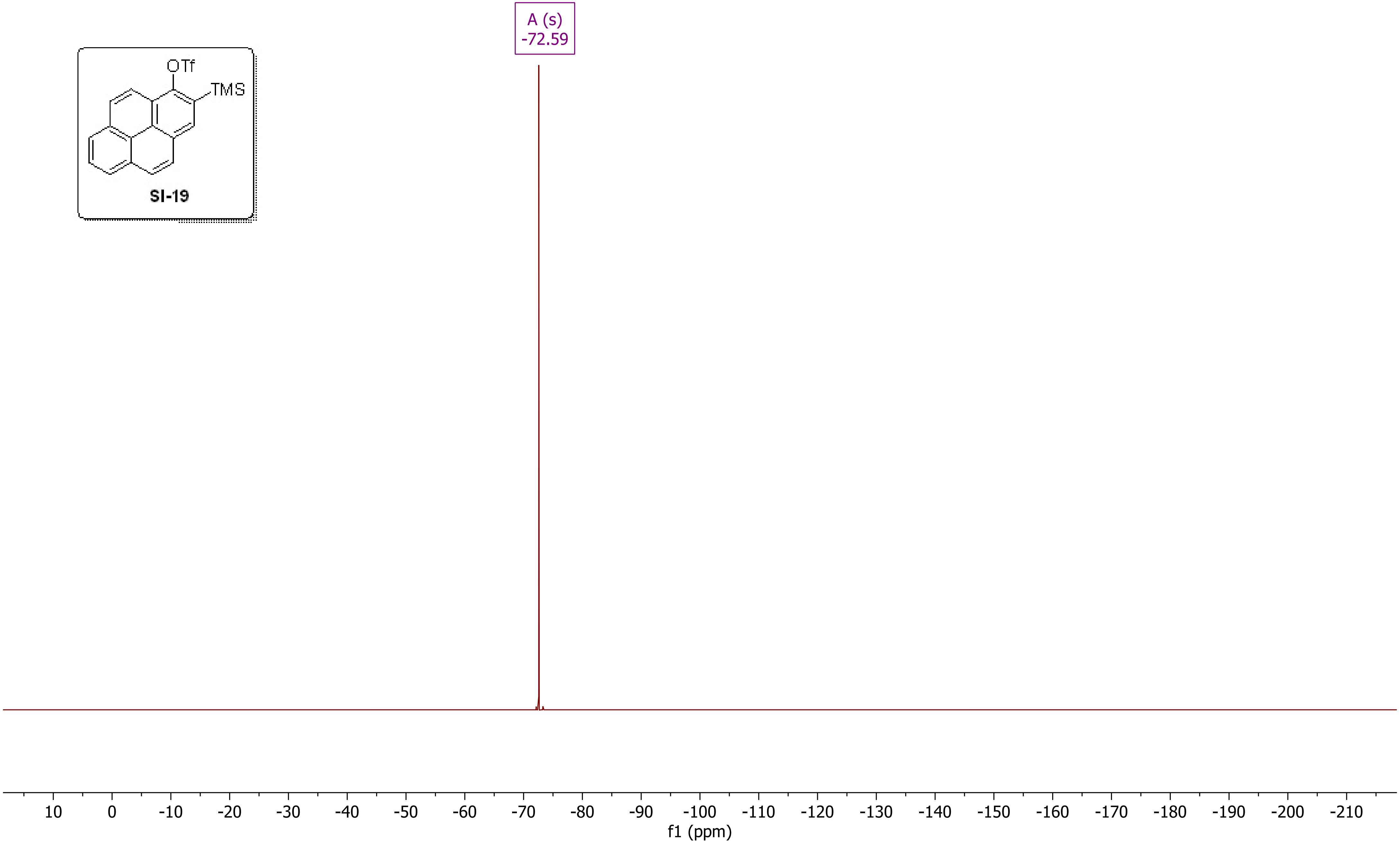

Compound SI-19, 2-(trimethylsilyl)pyren-1-yl trifluoromethanesulfonate, ^{13}C - NMR ($CDCl_3$, 101 MHz)

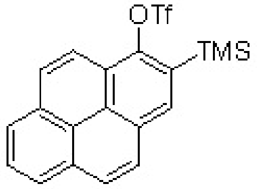

SI-19

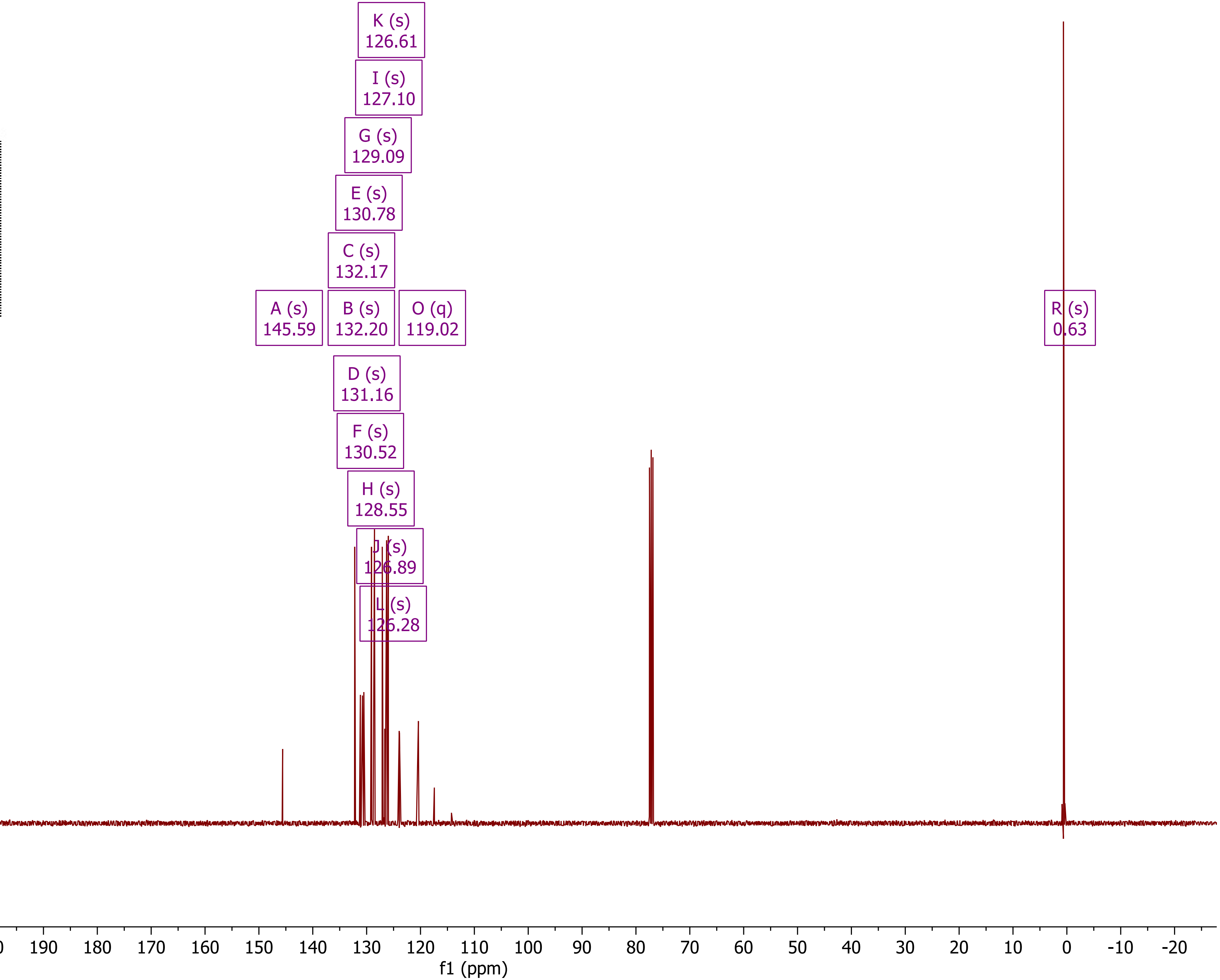

Compound SI-21, 4-(trimethylsilyl)ethynyl)phenyl isopropylcarbamate, ^{1}H - NMR ($CDCl_3$, 400 MHz)

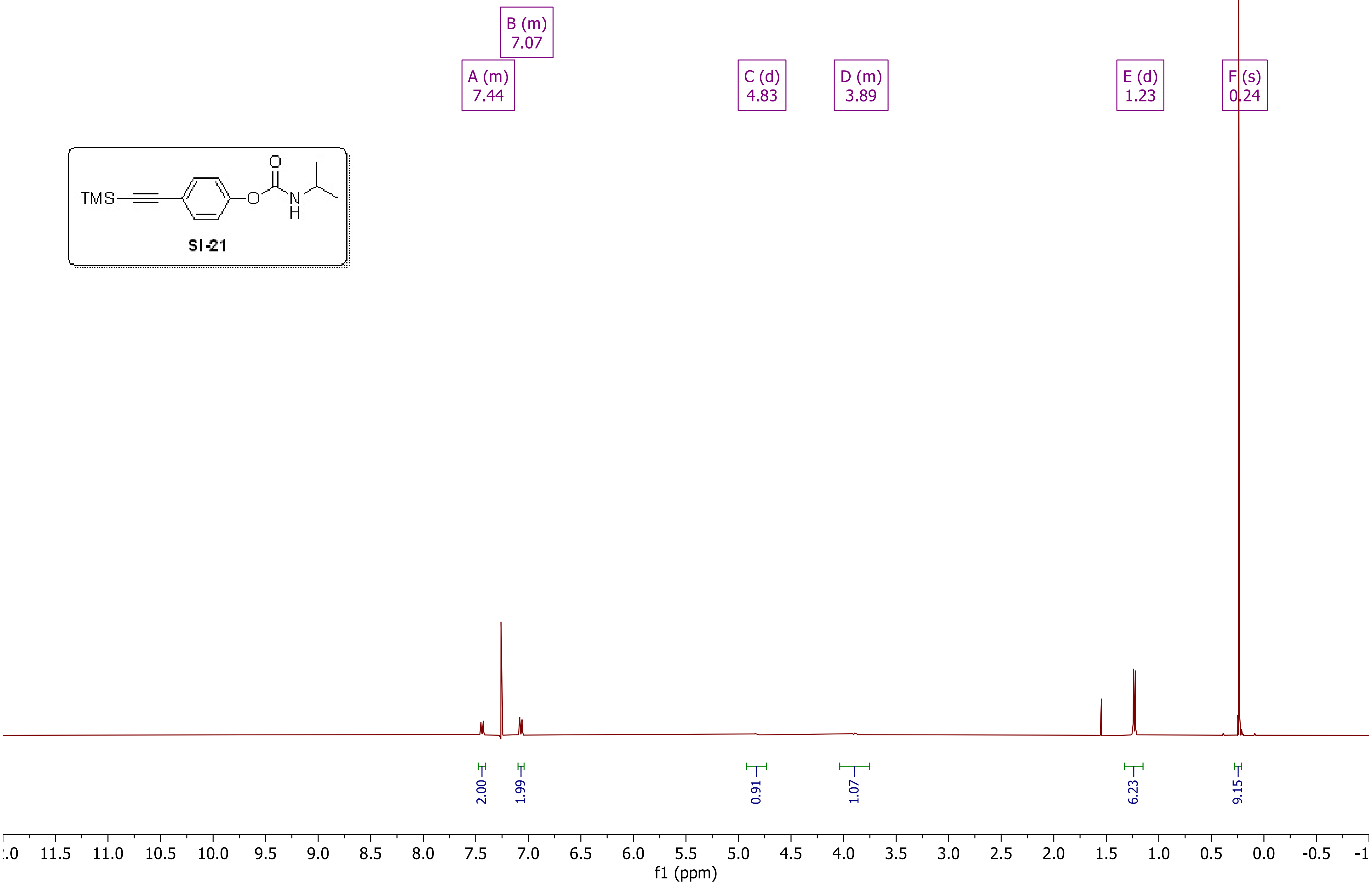

Compound SI-21, 4-(trimethylsilyl)ethynyl)phenyl isopropylcarbamate, ^{13}C - NMR ($CDCl_3$, 101 MHz)

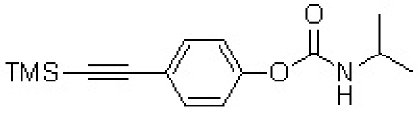

SI-21

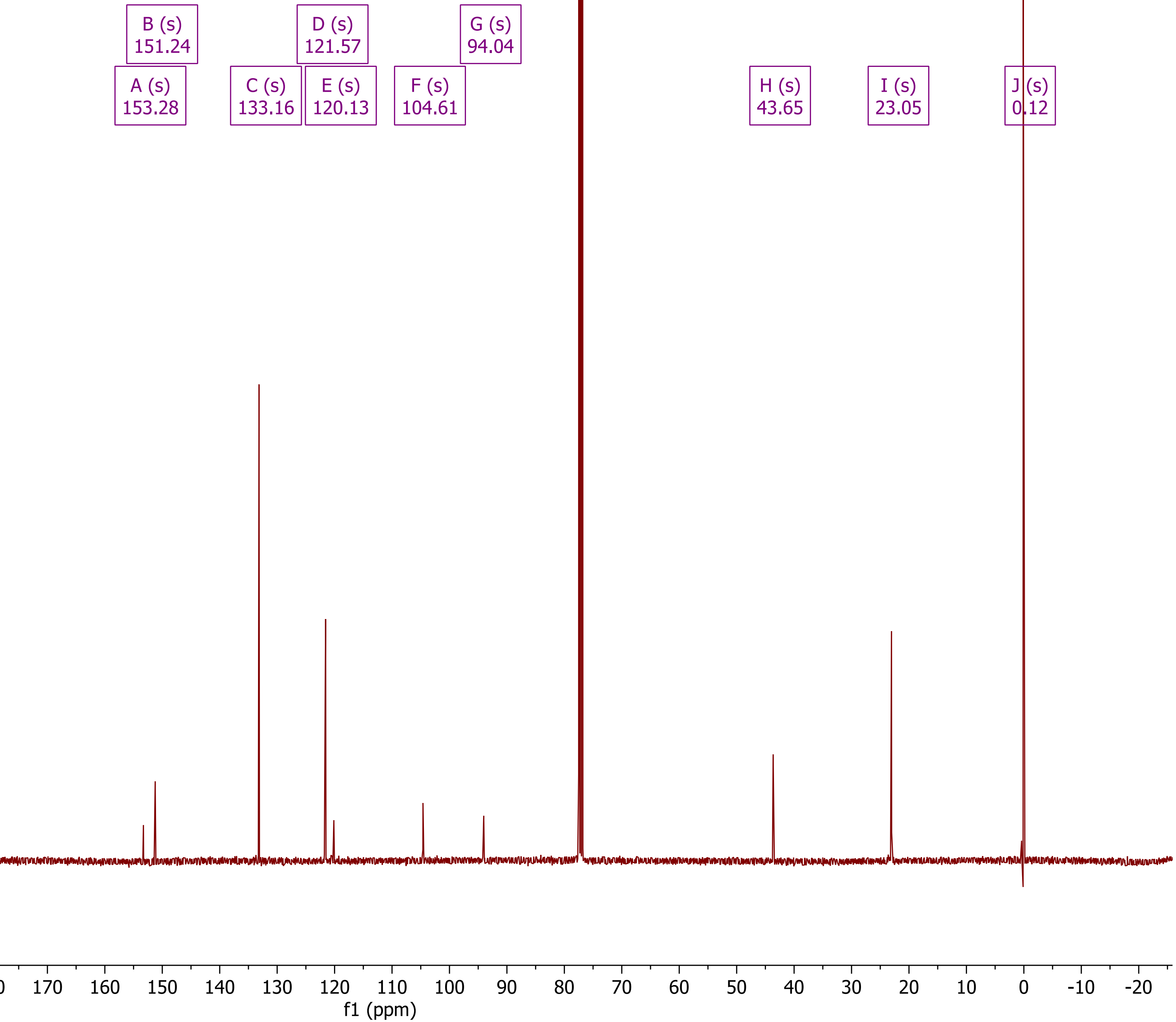

Compound SI-22, 2-(trimethylsilyl)-4-((trimethylsilyl)ethynyl)phenyl isopropylcarbamate, ^{1}H - NMR ($CDCl_3$, 400 MHz)

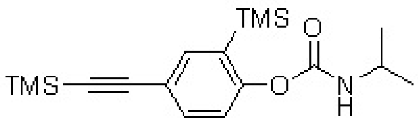

SI-22

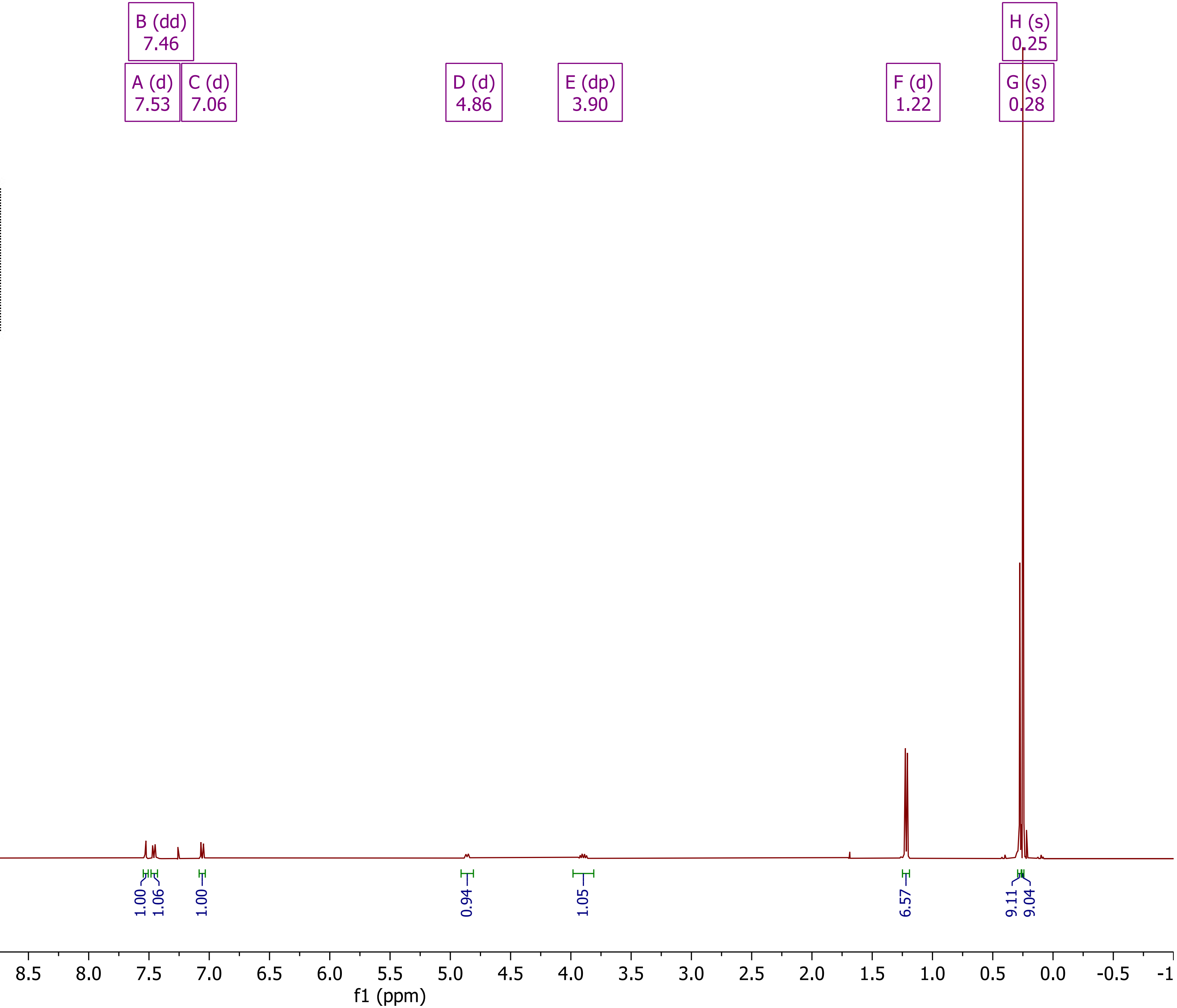

Compound SI-22, 2-(trimethylsilyl)-4-((trimethylsilyl)ethynyl)phenyl isopropylcarbamate, ^{13}C - NMR (CDCl$_3$, 101 MHz)

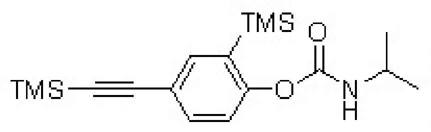

SI-22

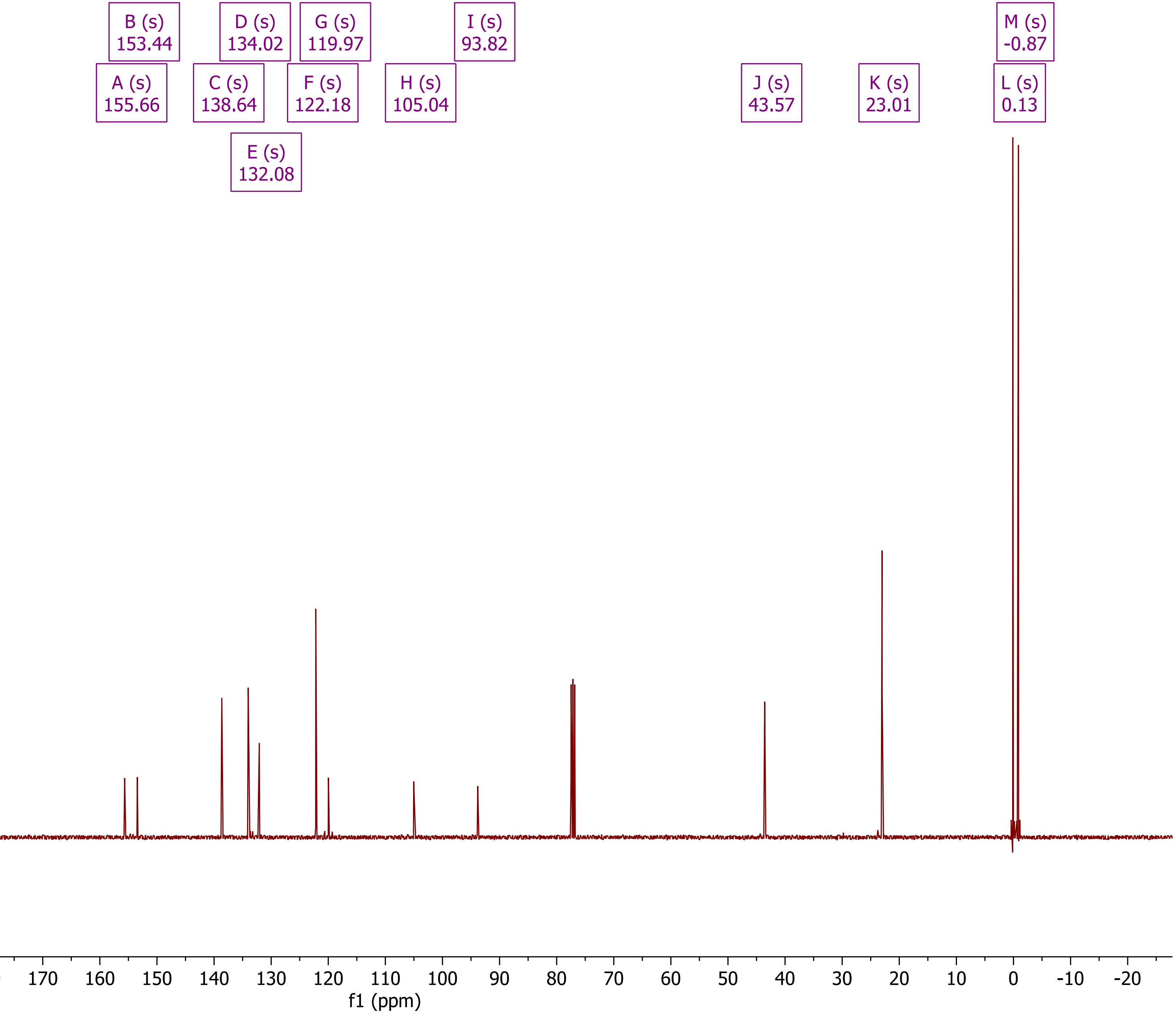

Compound SI-23, 2-(trimethylsilyl)-4-((trimethylsilyl)ethynyl)phenyl trifluoromethanesulfonate, ^{1}H - NMR ($CDCl_3$, 400 MHz)

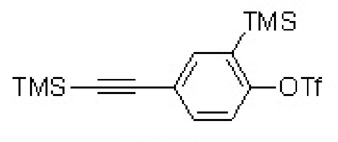

SI-23

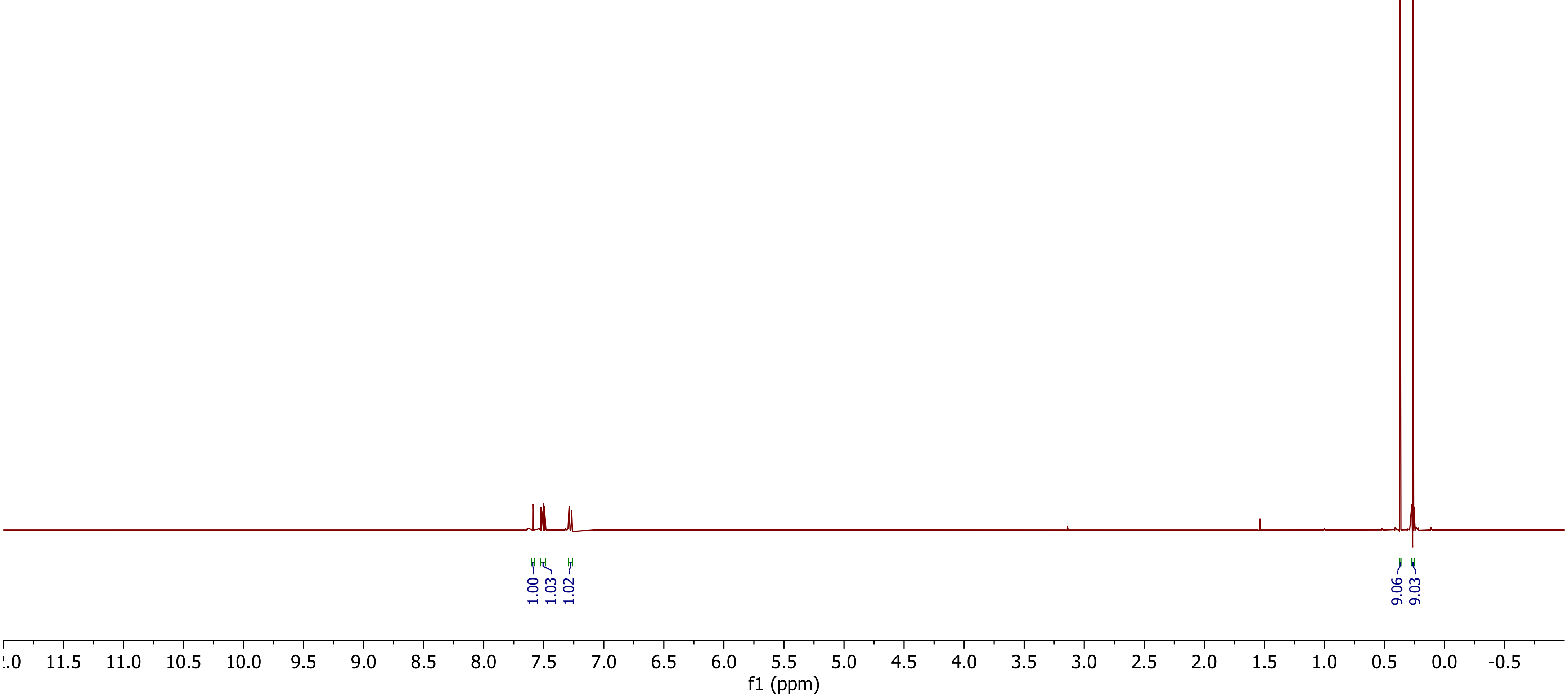

Compound SI-23, 2-(trimethylsilyl)-4-((trimethylsilyl)ethynyl)phenyl trifluoromethanesulfonate, ^{19}F - NMR ($CDCl_3$, 377 MHz)

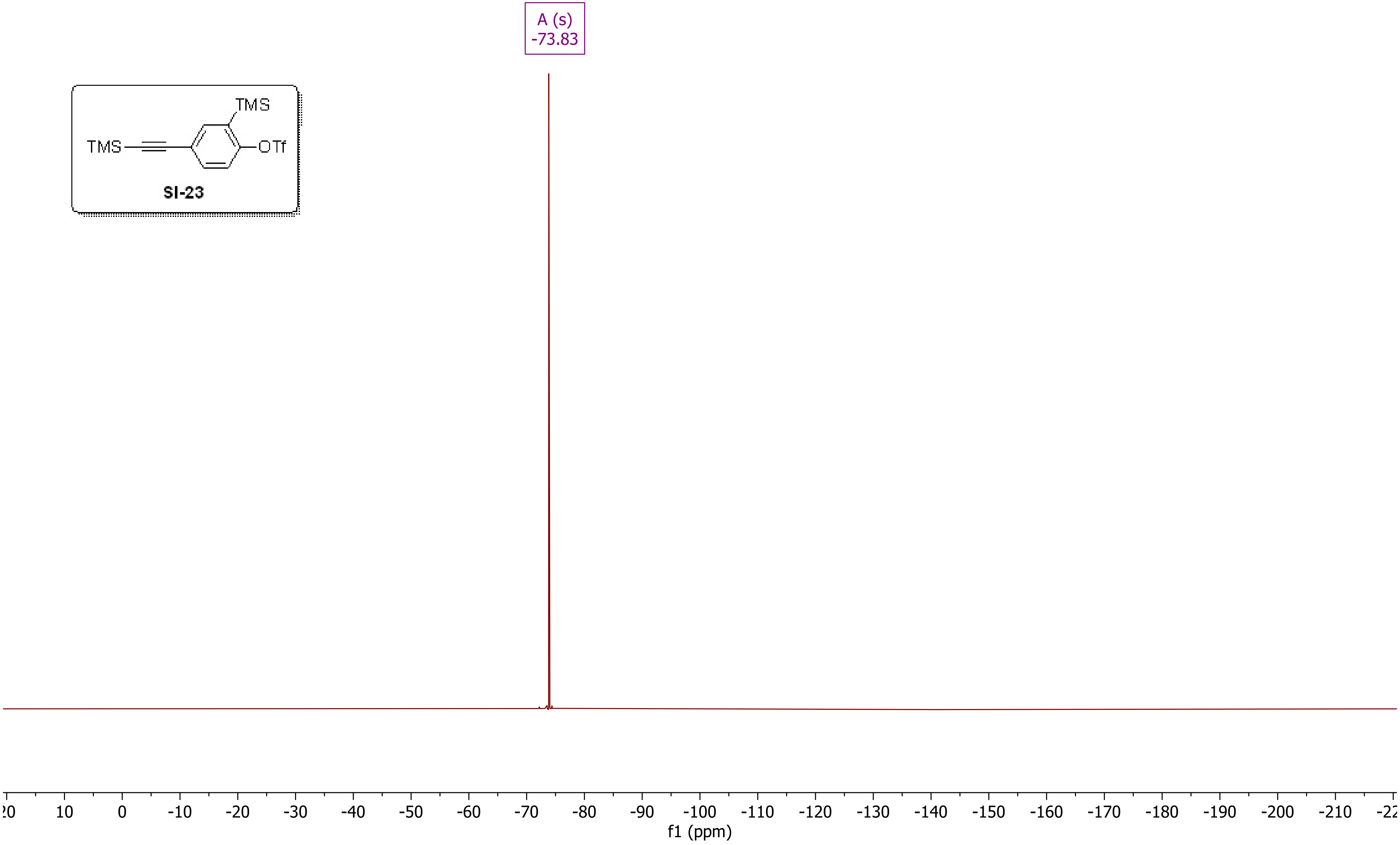

Compound SI-23, 2-(trimethylsilyl)-4-((trimethylsilyl)ethynyl)phenyl trifluoromethanesulfonate, ^{13}C - NMR ($CDCl_3$, 101 MHz)

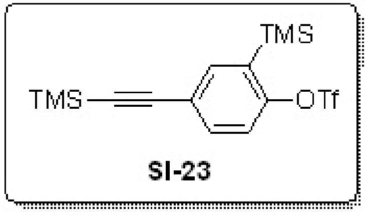

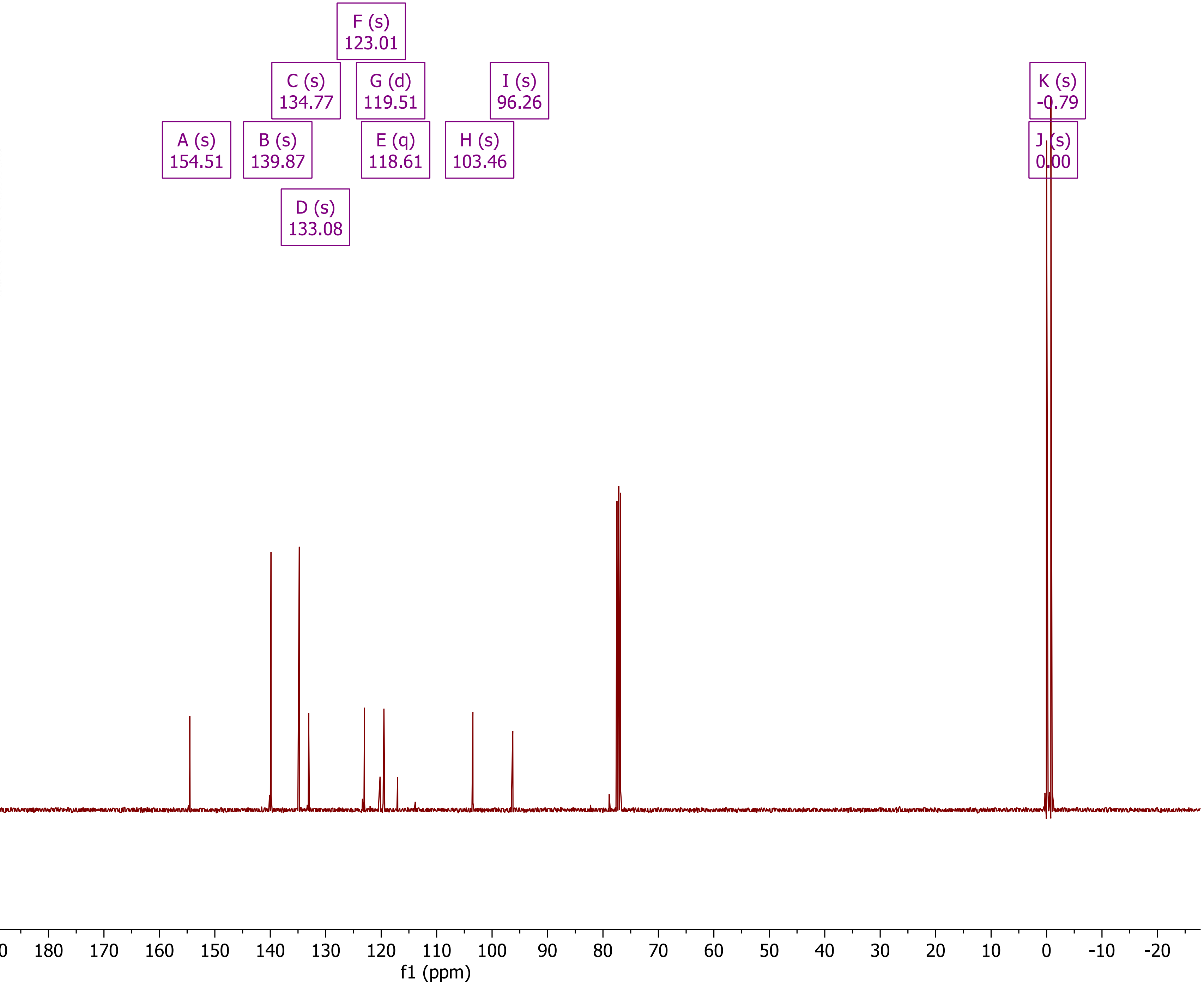

Compound SI-25, 2-((7-(diethylamino)-2-oxo-2H-chromen-4-yl)methyl)isoindoline-1,3-dione, ¹H - NMR (D_2O, 400 MHz)

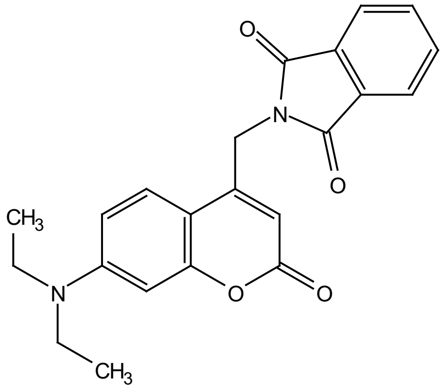

SI-25

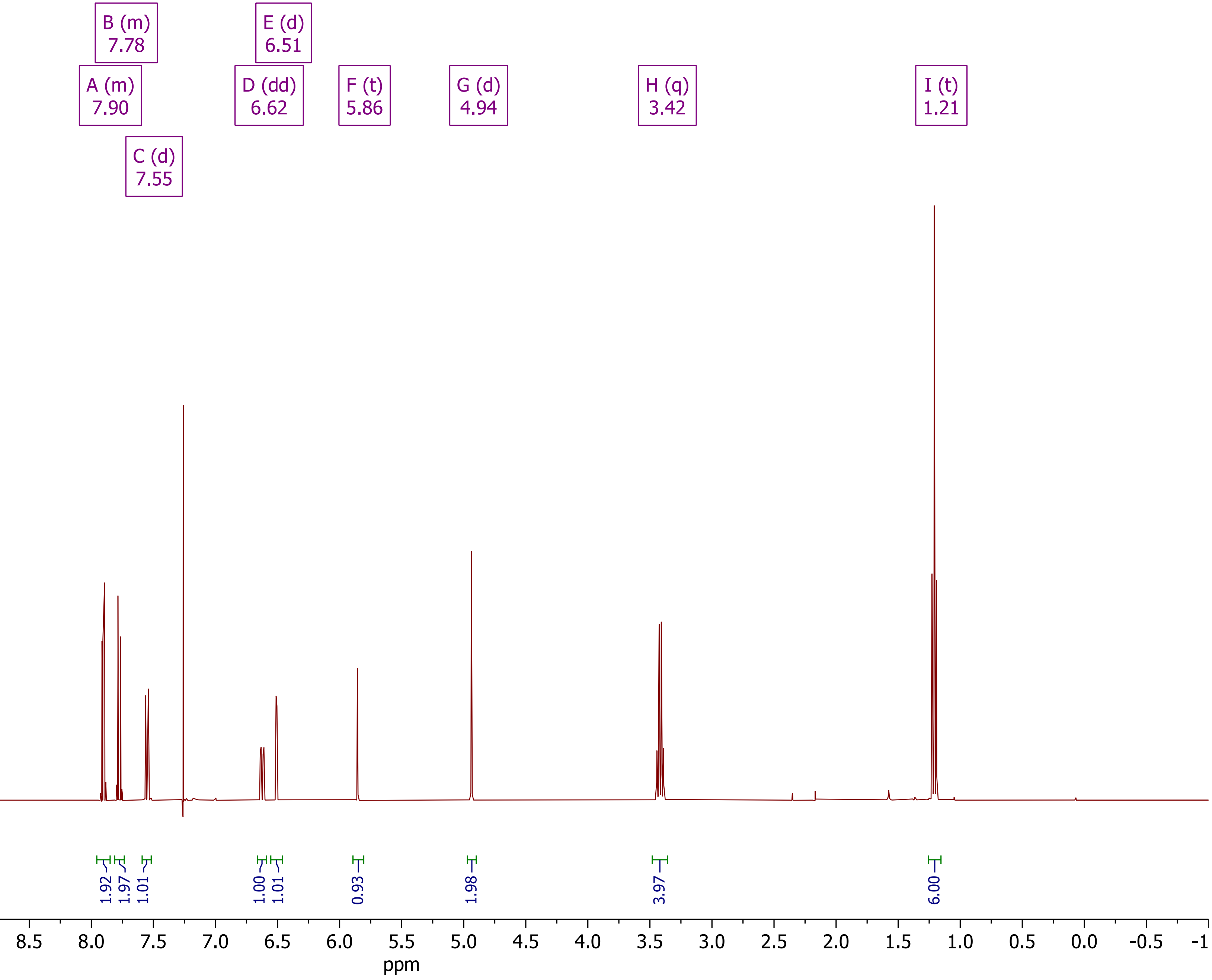

Compound SI-25, 2-((7-(diethylamino)-2-oxo-2H-chromen-4-yl)methyl)isoindoline-1,3-dione, $^{13}C\{^{1}H\}$ - NMR (D2O, 101 MHz)

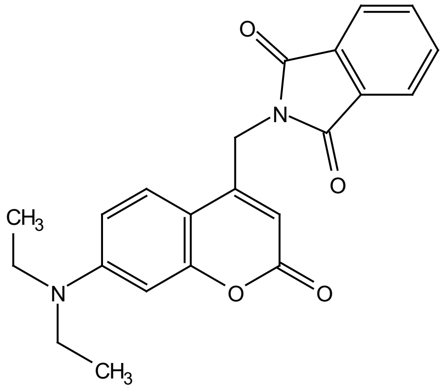

SI-25

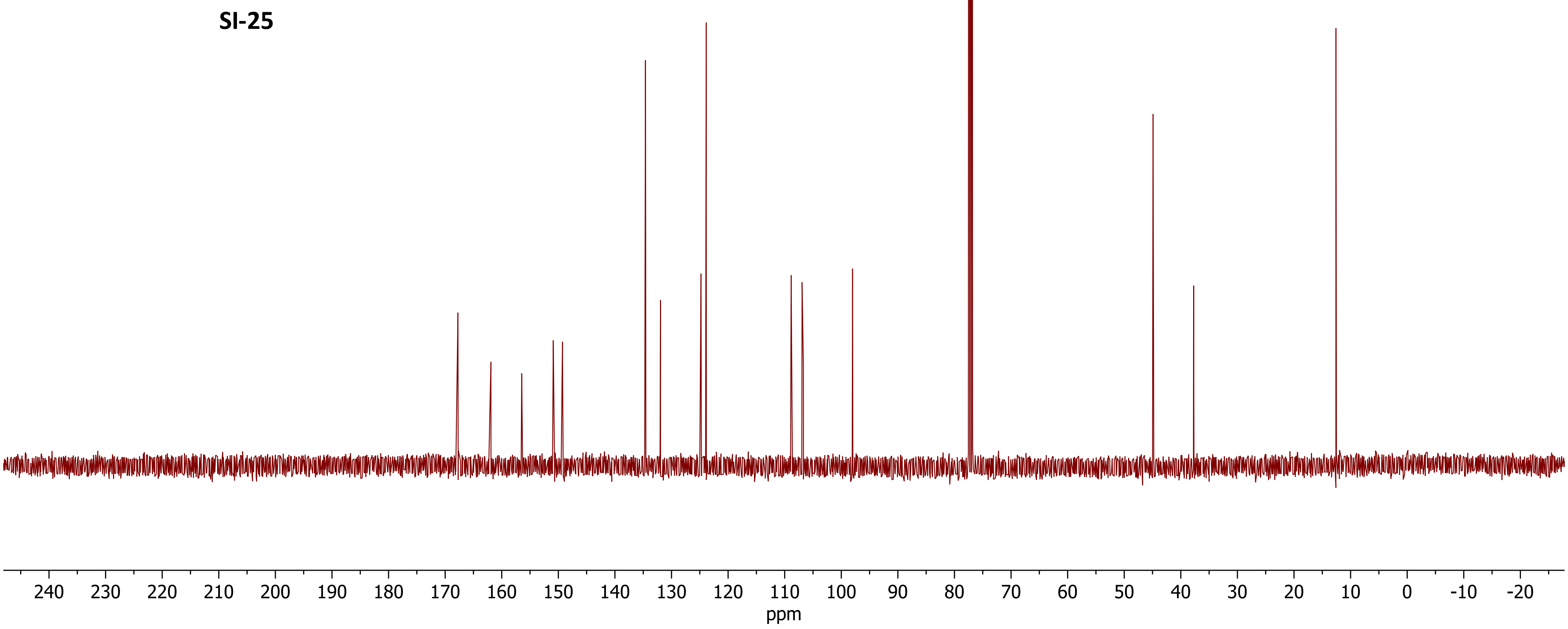

Compound SI-26, 4-(aminomethyl)-7-(diethylamino)-2H-chromen-2-one, ^{1}H - NMR (D_2O, 400 MHz)

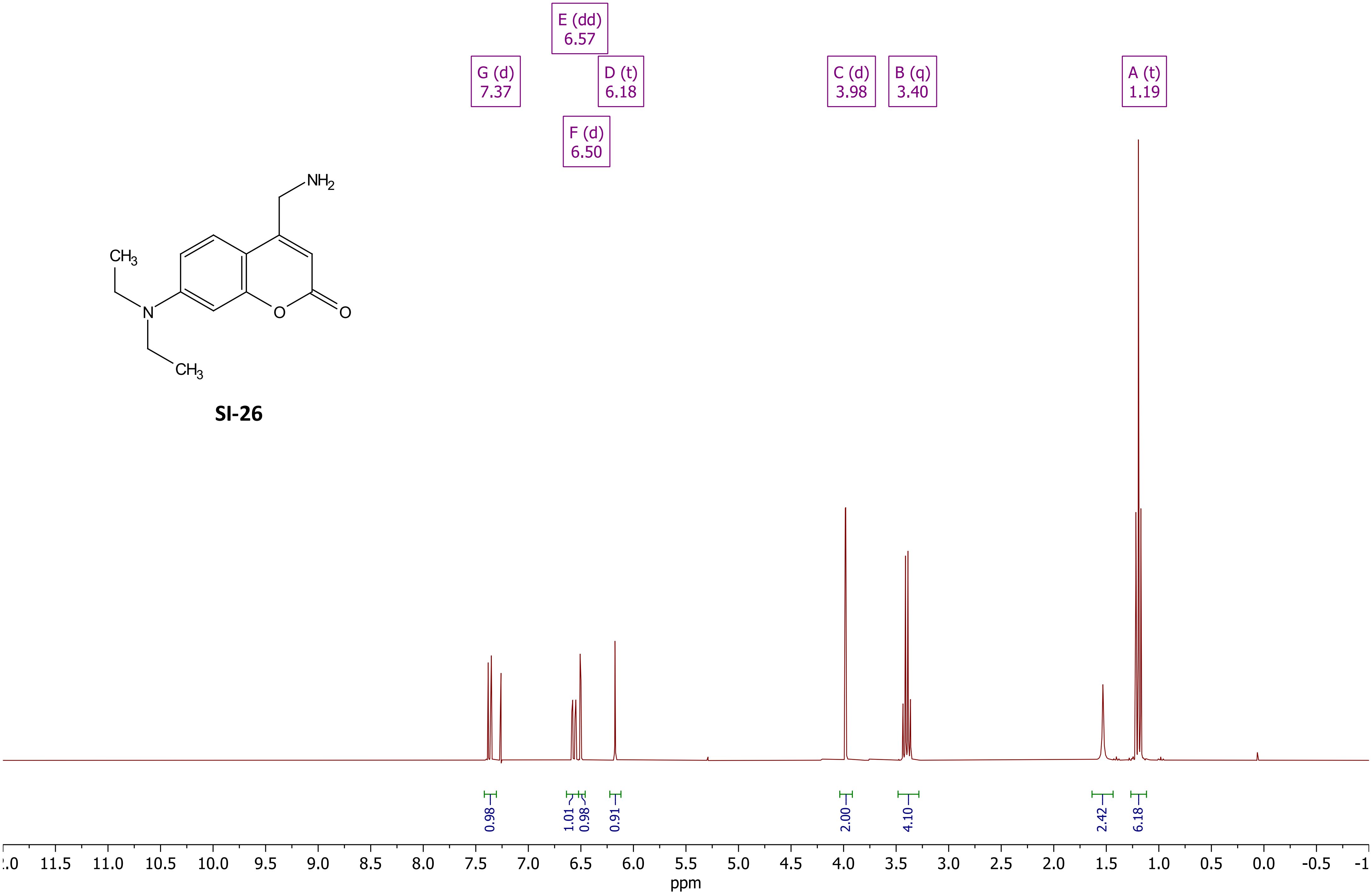

Compound SI-26, 4-(aminomethyl)-7-(diethylamino)-2H-chromen-2-one, $^{13}C\{^1H\}$ - NMR (D2O, 101 MHz)

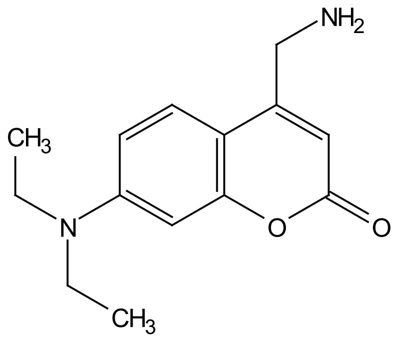

SI-26

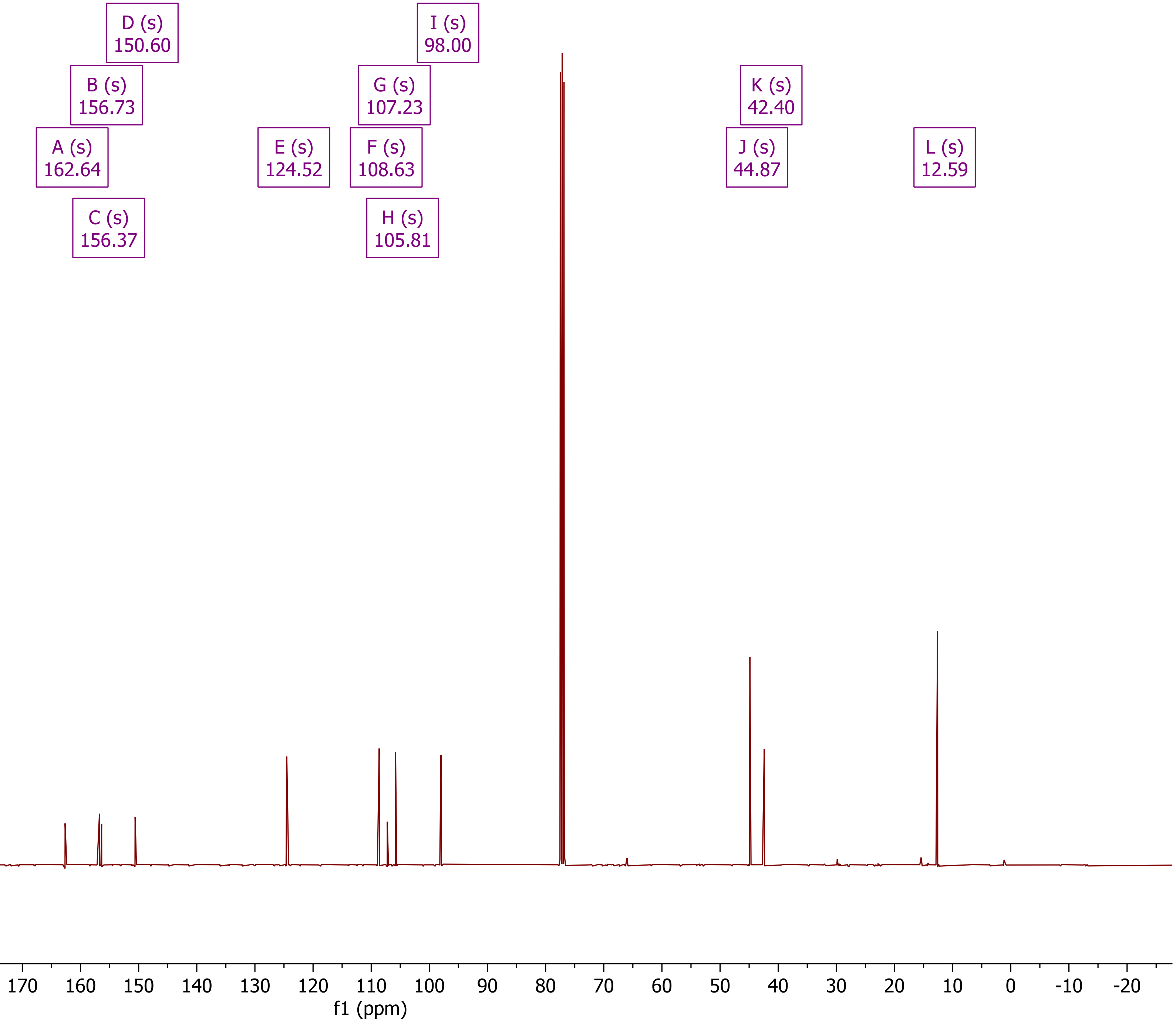

13. MS – spectra

(aligned according to molecule numbering)

HRMS (ESI) Analysis of compound 27: phenyl phosphate

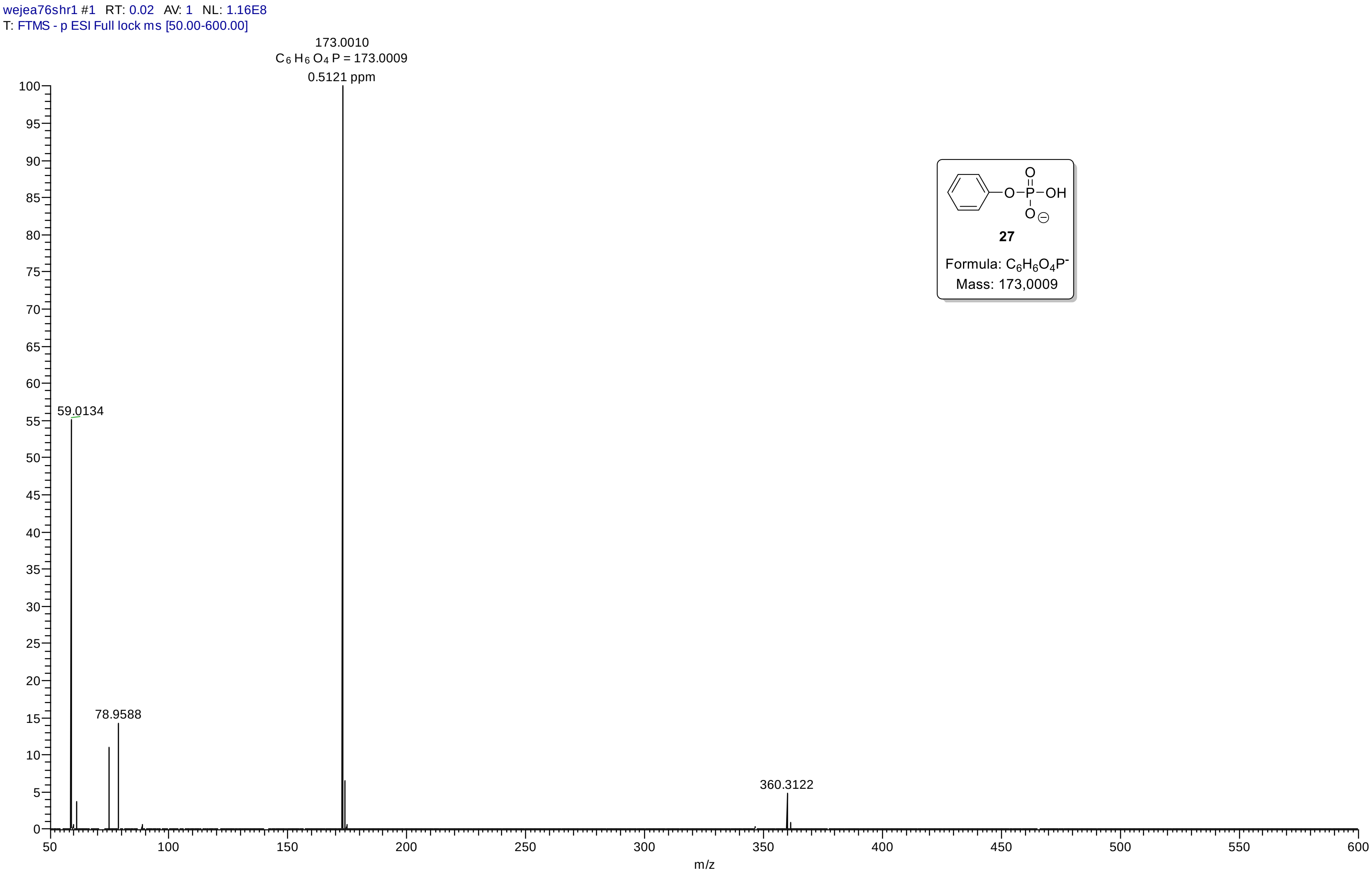

HRMS (APCI) Analysis of compound 28: 2-naphthalen-2-yl phosphate

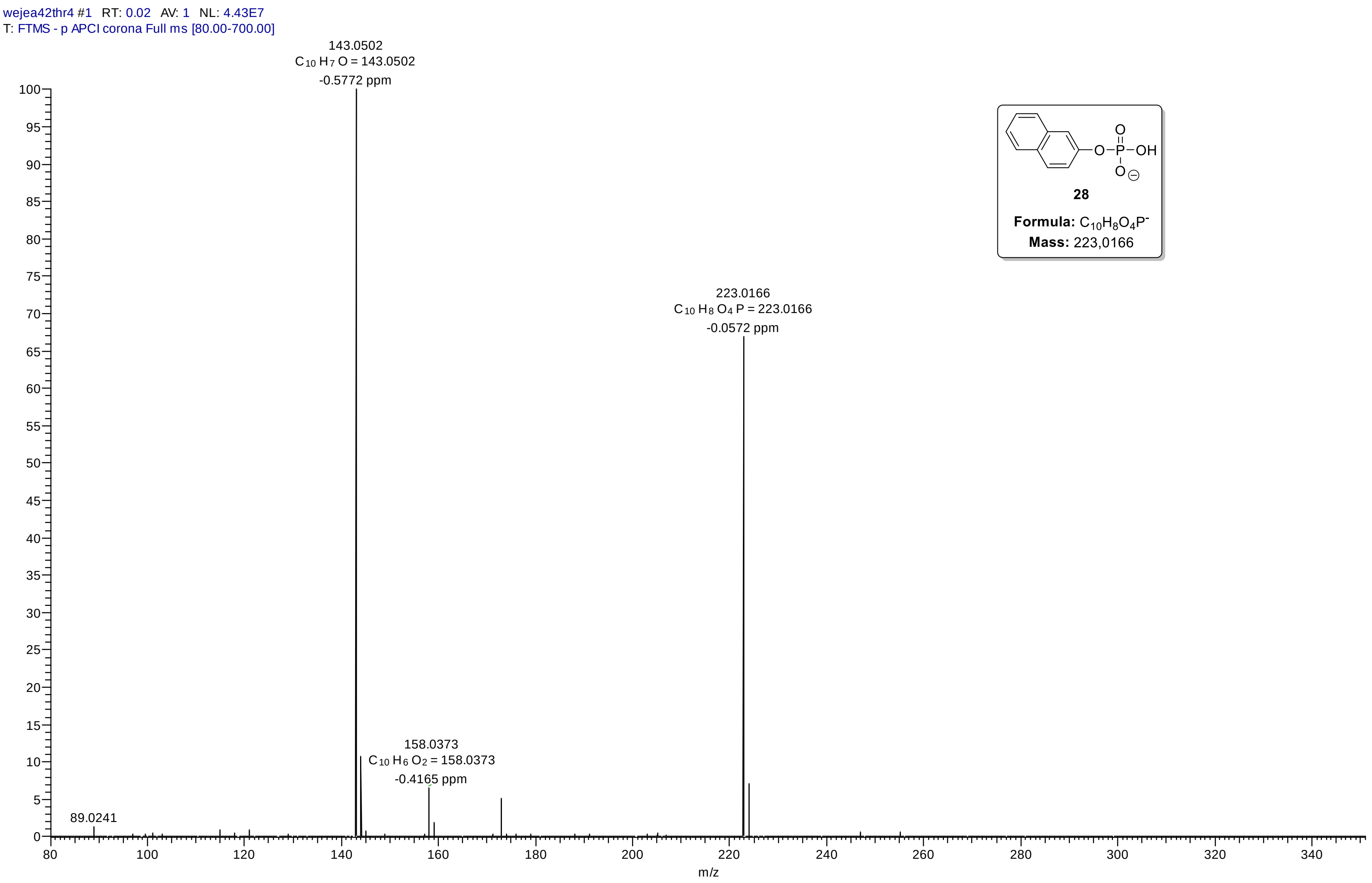

HRMS (APCI) Analysis of compound 29: 3,4-dimethylphenyl phosphate

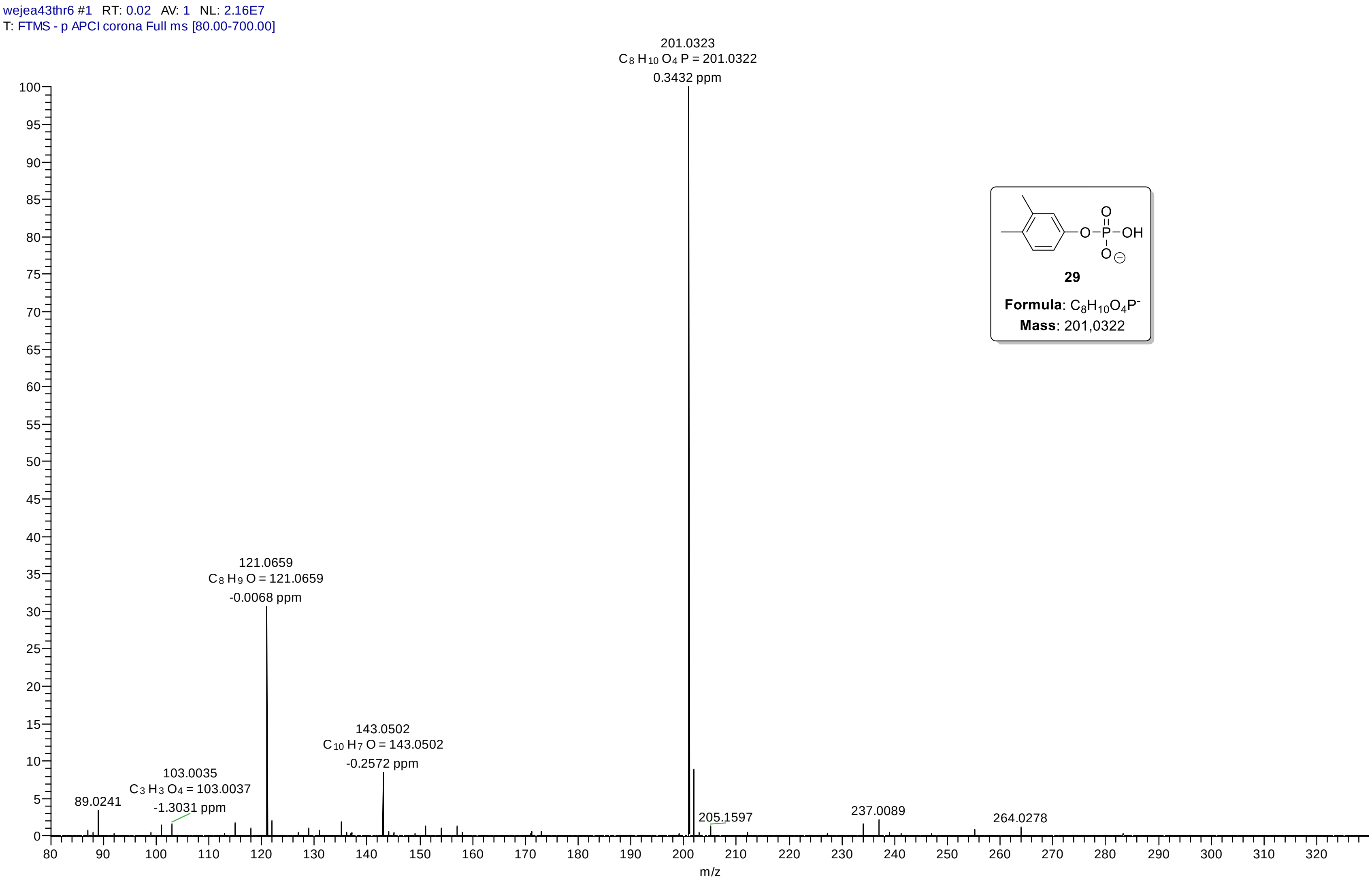

29

Formula: $C_8H_{10}O_4P^-$
Mass: 201,0322

HRMS (ESI) Analysis of compound 30: 1H-indol-5-yl phosphate

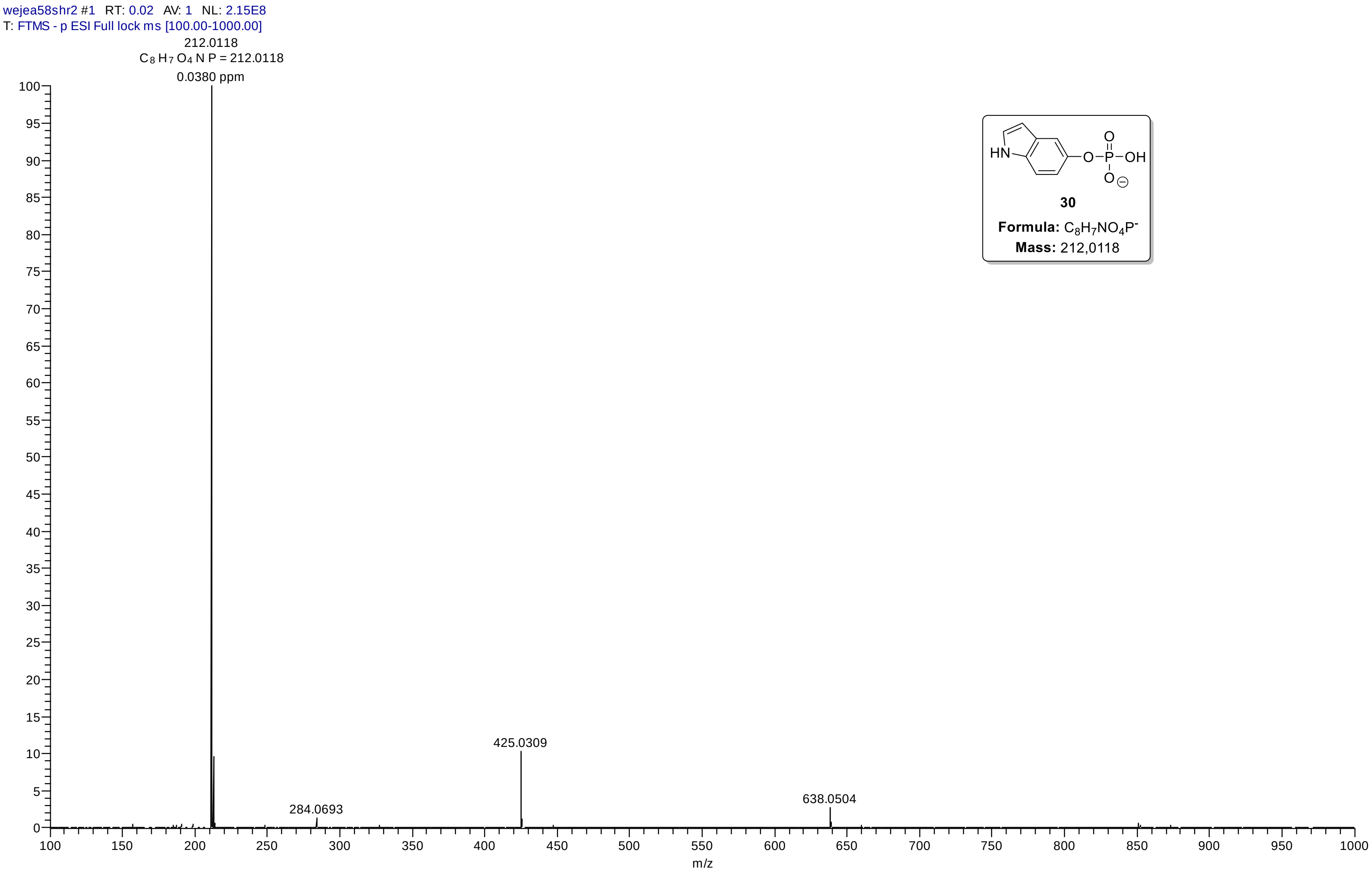

HRMS (ESI) Analysis of compound 31: pyren-2-yl phosphate

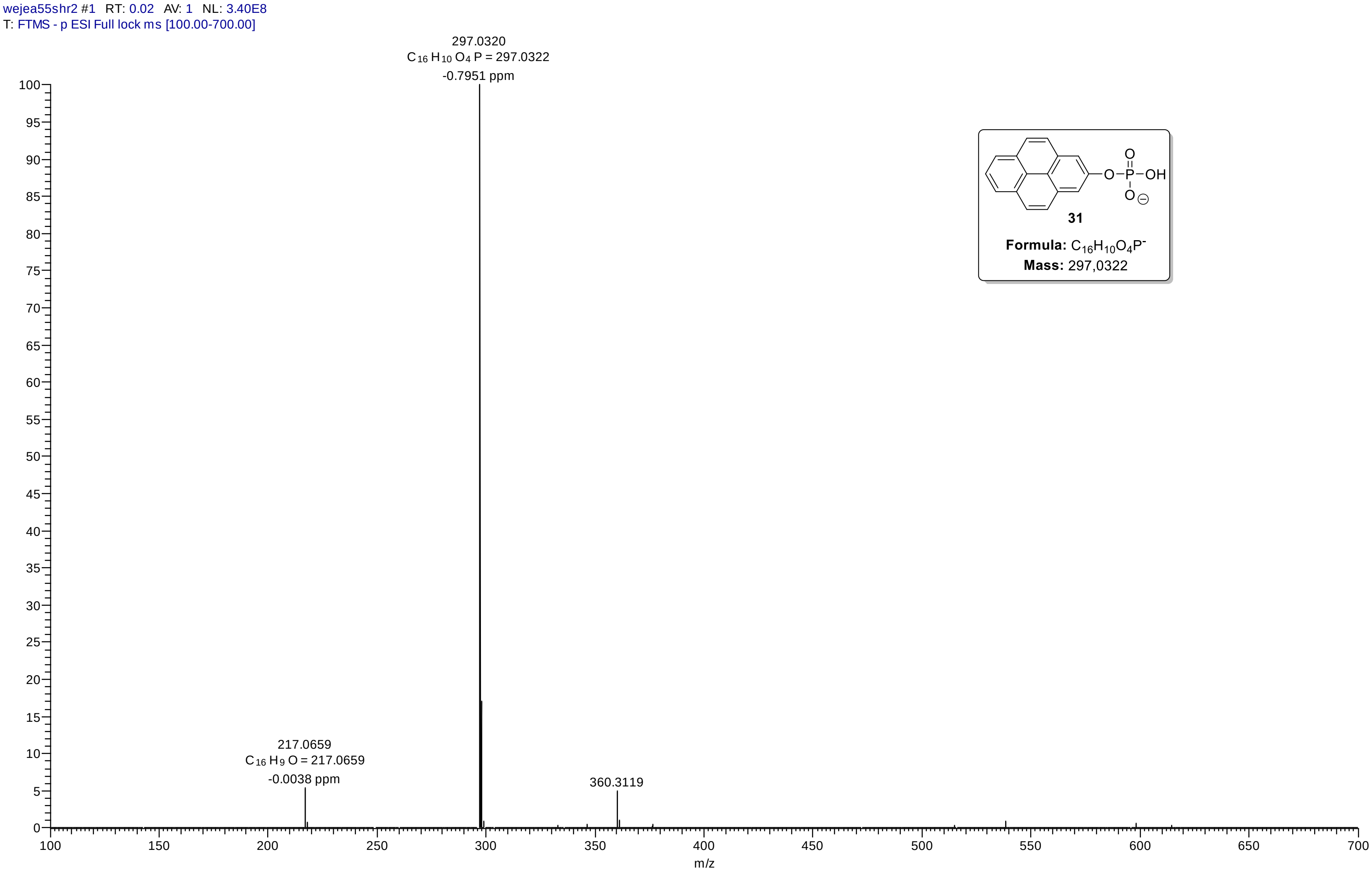

31

Formula: $C_{16}H_{10}O_4P^-$

Mass: 297,0322

HRMS (ESI) Analysis of compound 32: phenyl diphosphate

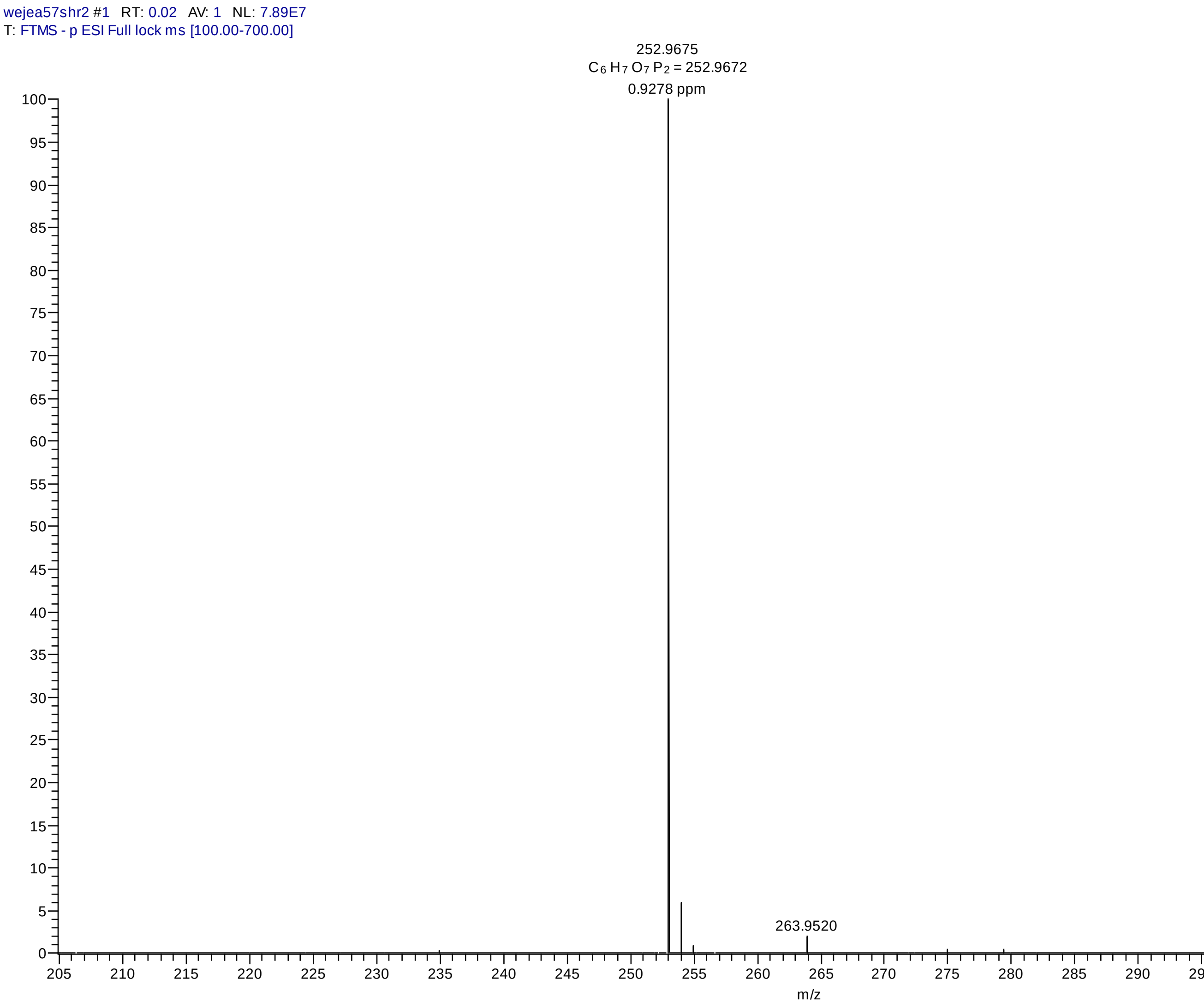

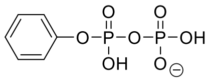

32

Formula: $C_6H_7O_7P_2^-$

Mass: 252,9672

HRMS (ESI) Analysis of compound 33: 2-naphthalen-2-yl diphosphate

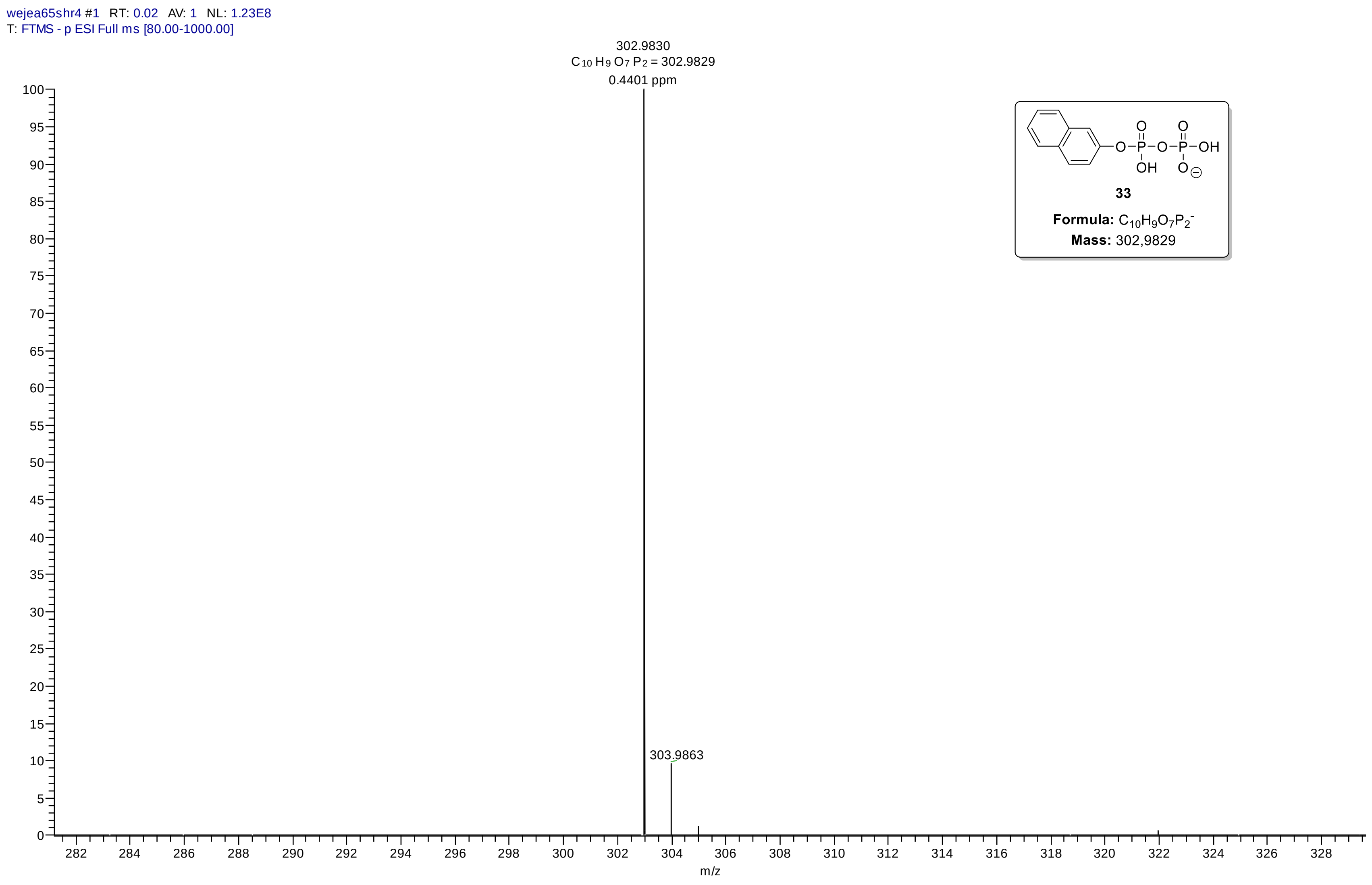

HRMS (ESI) Analysis of compound 34: 3,4-dimethylphenyl diphosphate

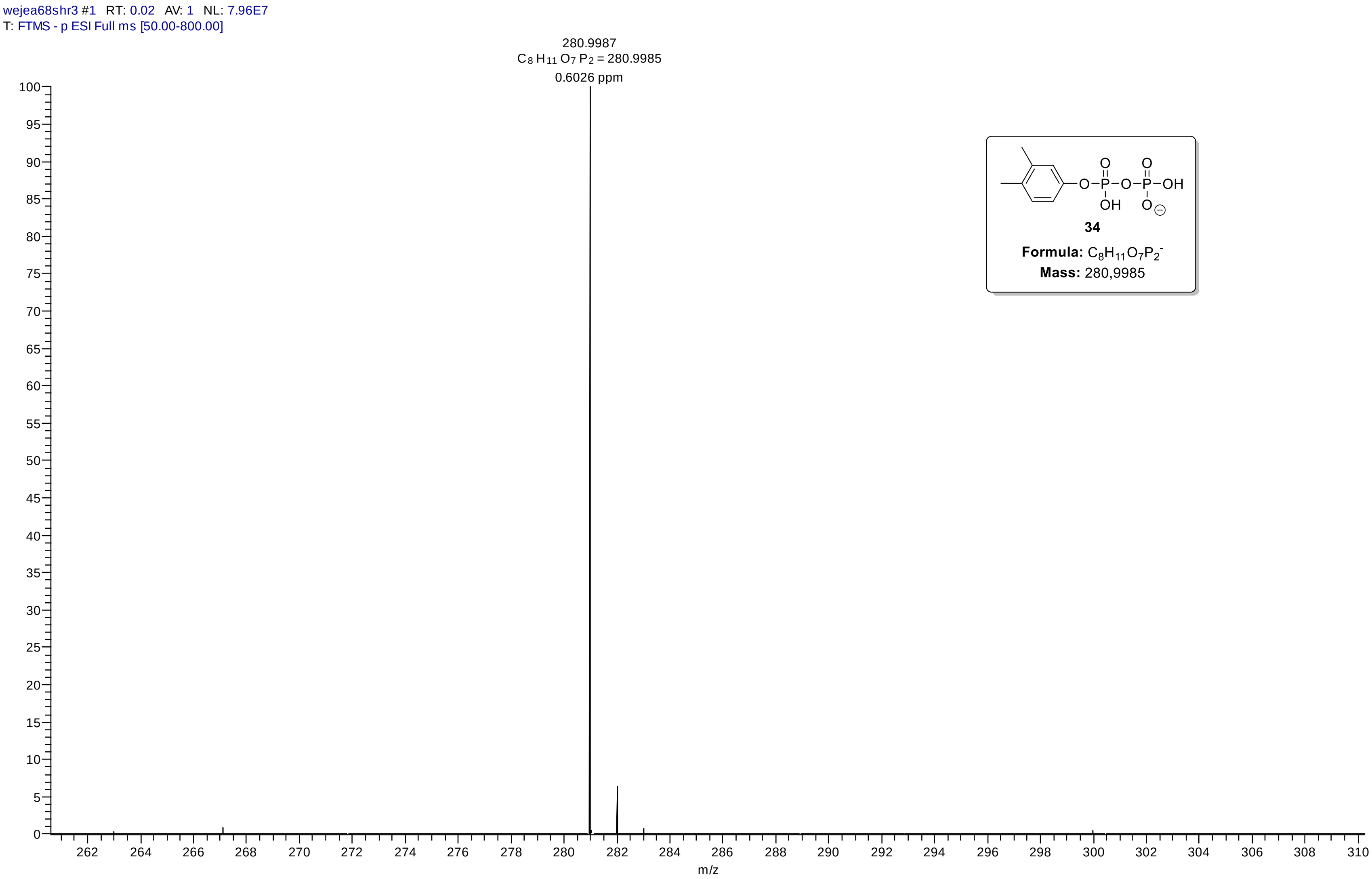

34

Formula: $C_8H_{11}O_7P_2^-$

Mass: 280,9985

HRMS (ESI) Analysis of compound 35: 1H-indol-5-yl diphosphate

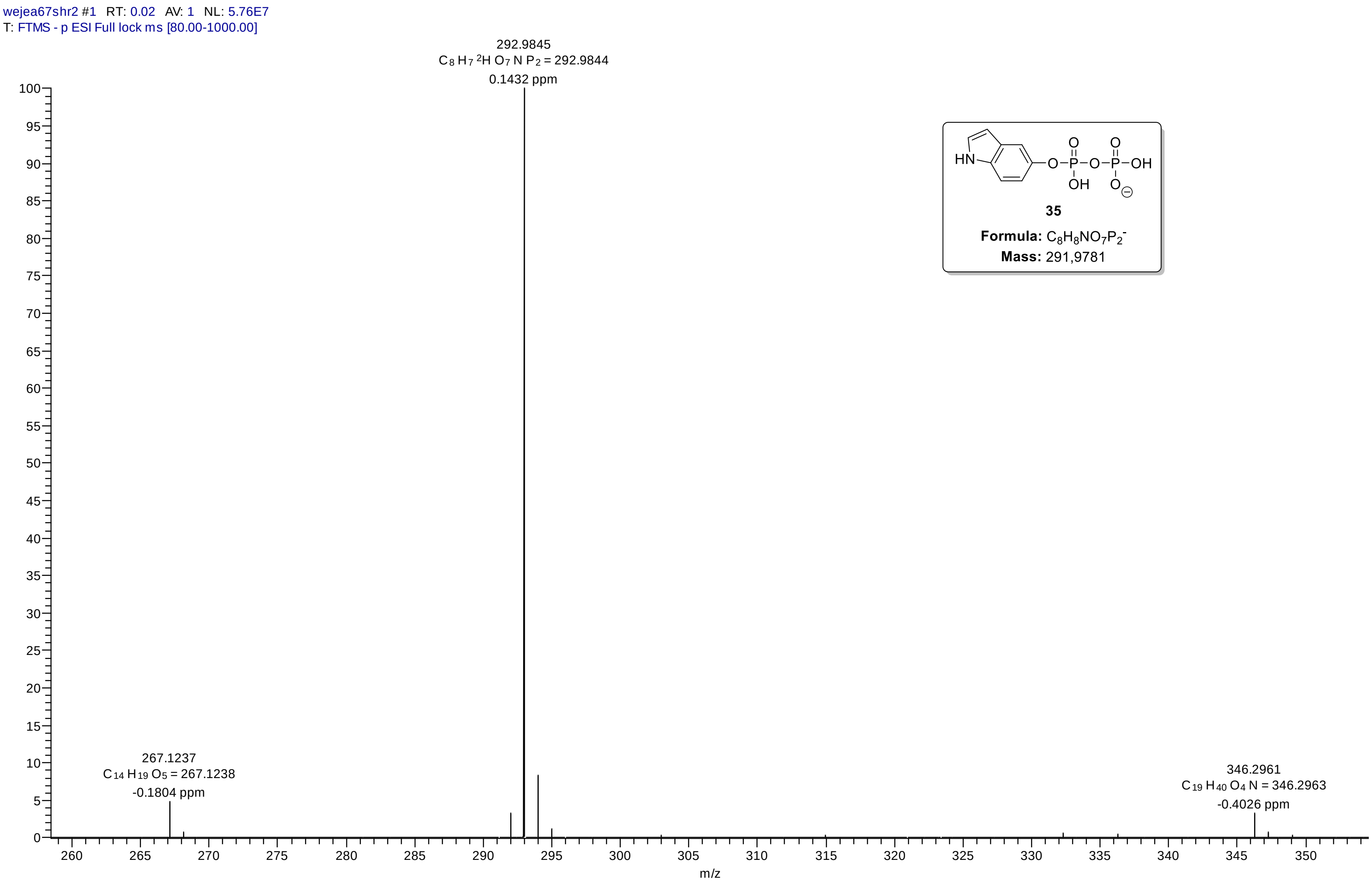

35

Formula: $C_8H_8NO_7P_2^-$

Mass: 291,9781

HRMS (ESI) Analysis of compound 36: pyren-2-yl diphosphate

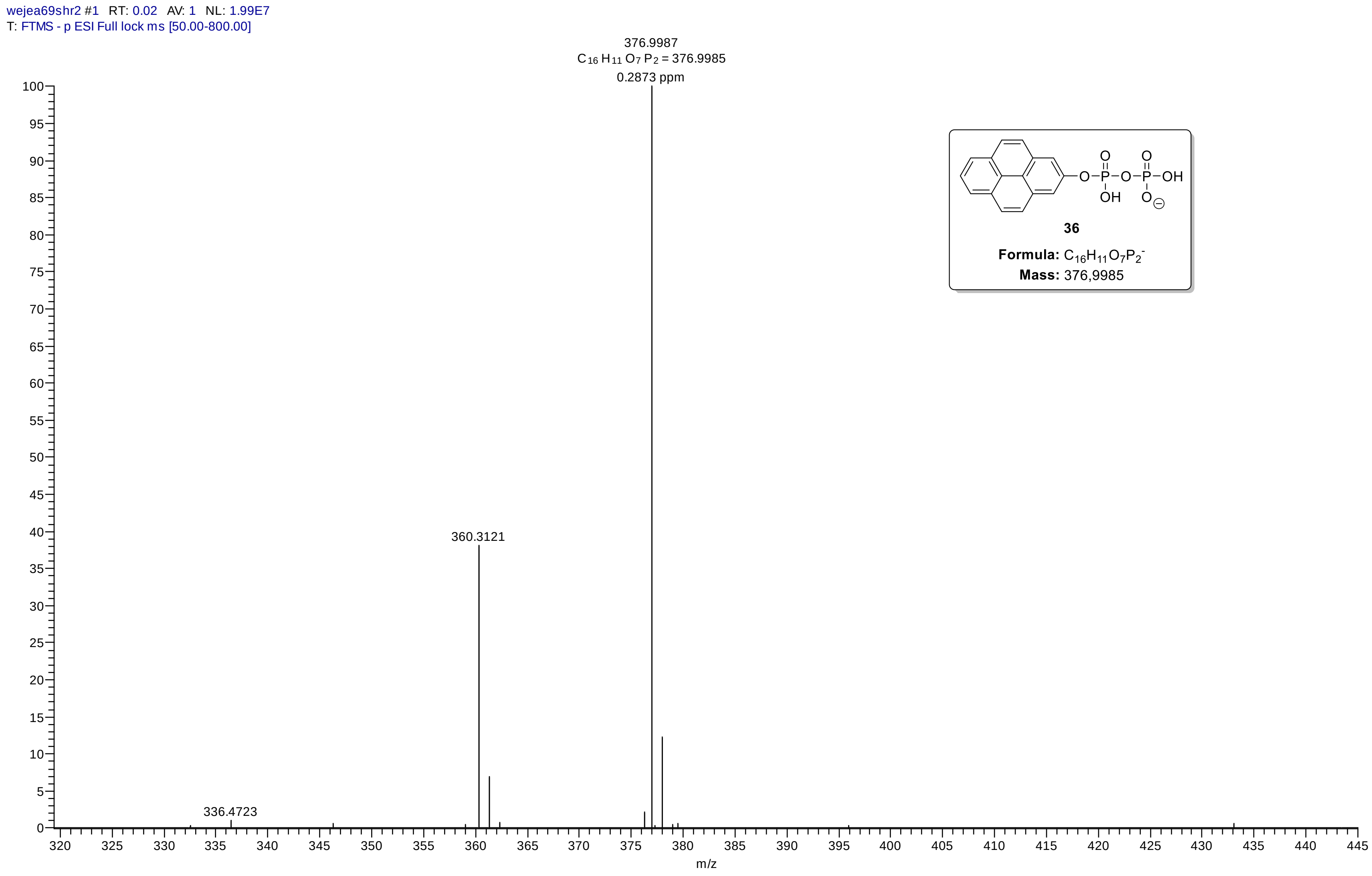

HRMS (ESI) Analysis of compound 37: deutero-phenylphosphate

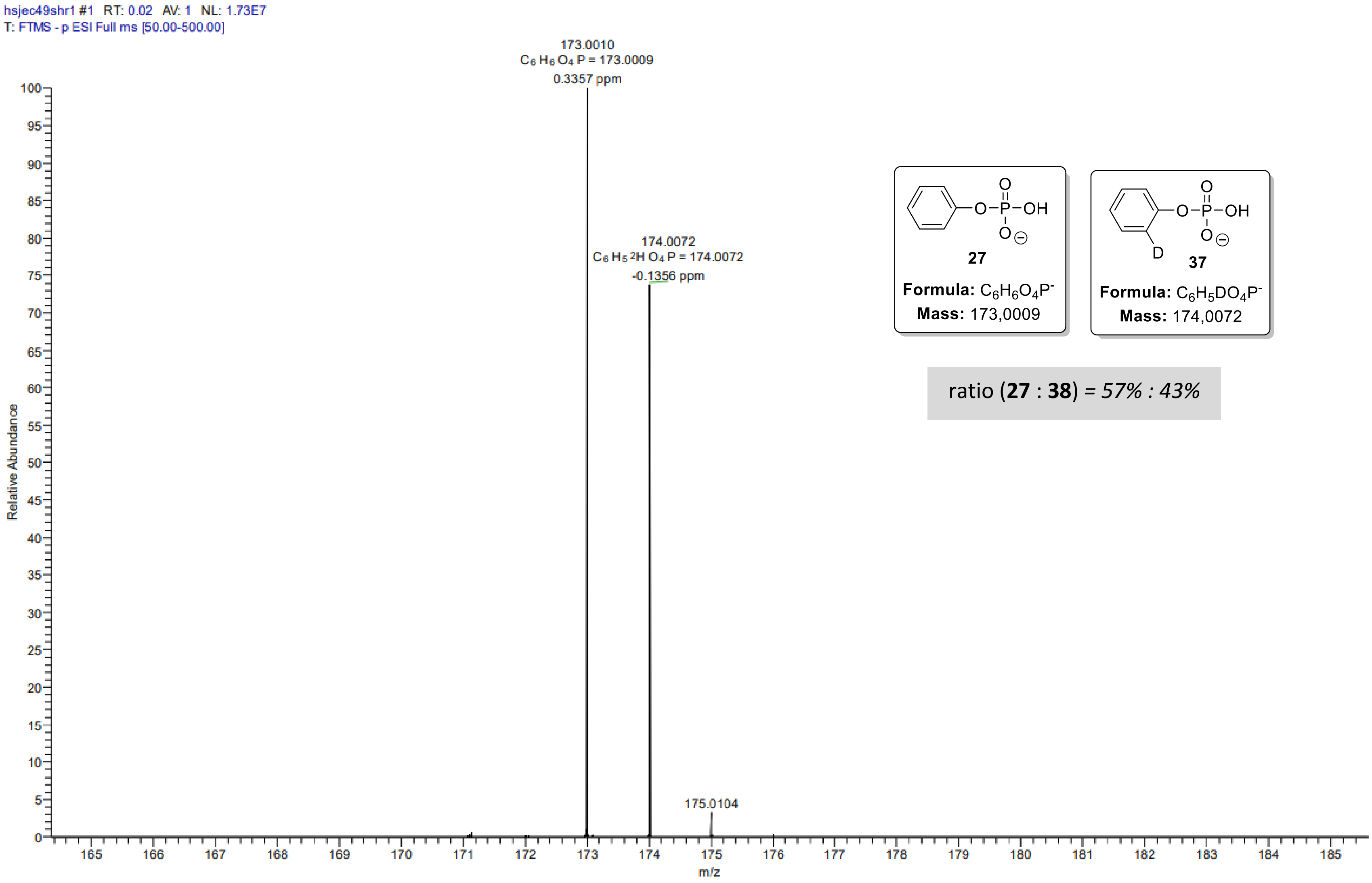

27

Formula: $C_6H_6O_4P^-$
Mass: 173,0009

37

Formula: $C_6H_5DO_4P^-$
Mass: 174,0072

ratio (**27** : **38**) = *57% : 43%*

HRMS (ESI) Analysis of compound 38: 5,5,5-trifluoropentyl phosphate

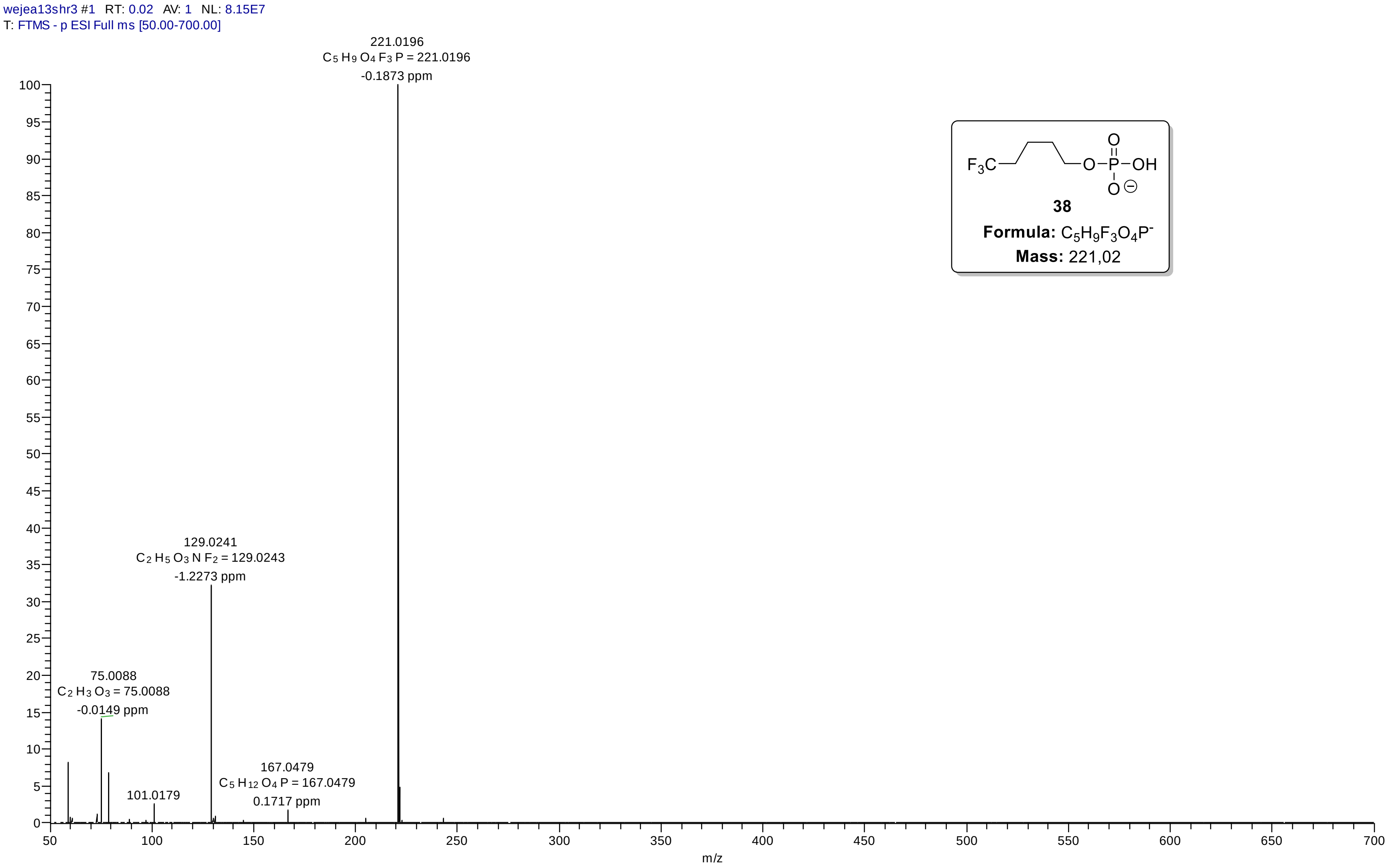

38

Formula: $C_5H_9F_3O_4P^-$

Mass: 221,02

HRMS (ESI) Analysis of compound 40: phenyl (5,5,5-trifluoropentyl) phosphate

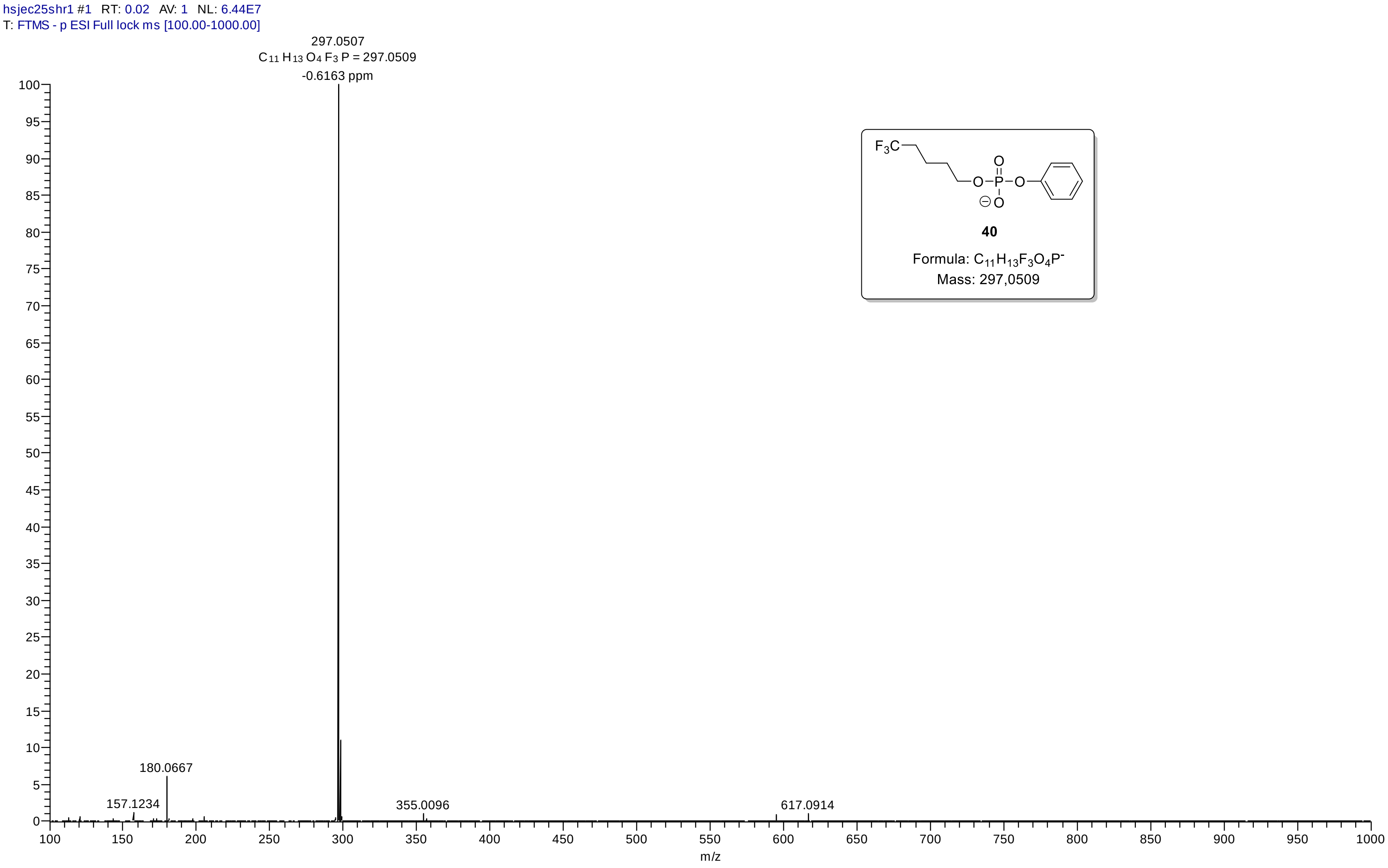

HRMS (ESI) Analysis of compound 41: 3,4-dimethylphenyl (5,5,5-trifluoropentyl) phosphate

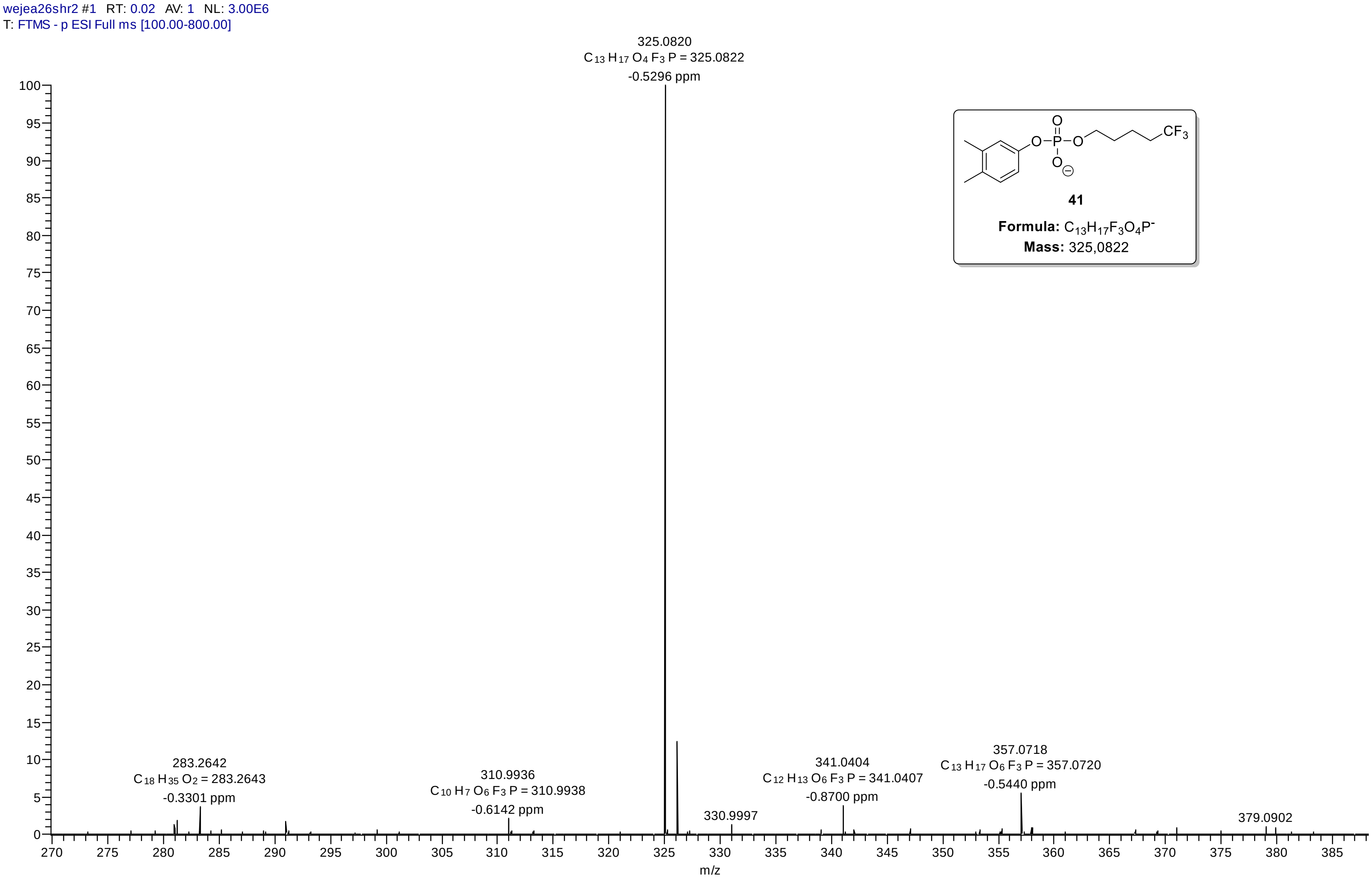

41

Formula: $C_{13}H_{17}F_3O_4P^-$

Mass: 325,0822

HRMS (ESI) Analysis of compound 42: 2,5-dimethylphenyl (5,5,5-trifluoropentyl) phosphate

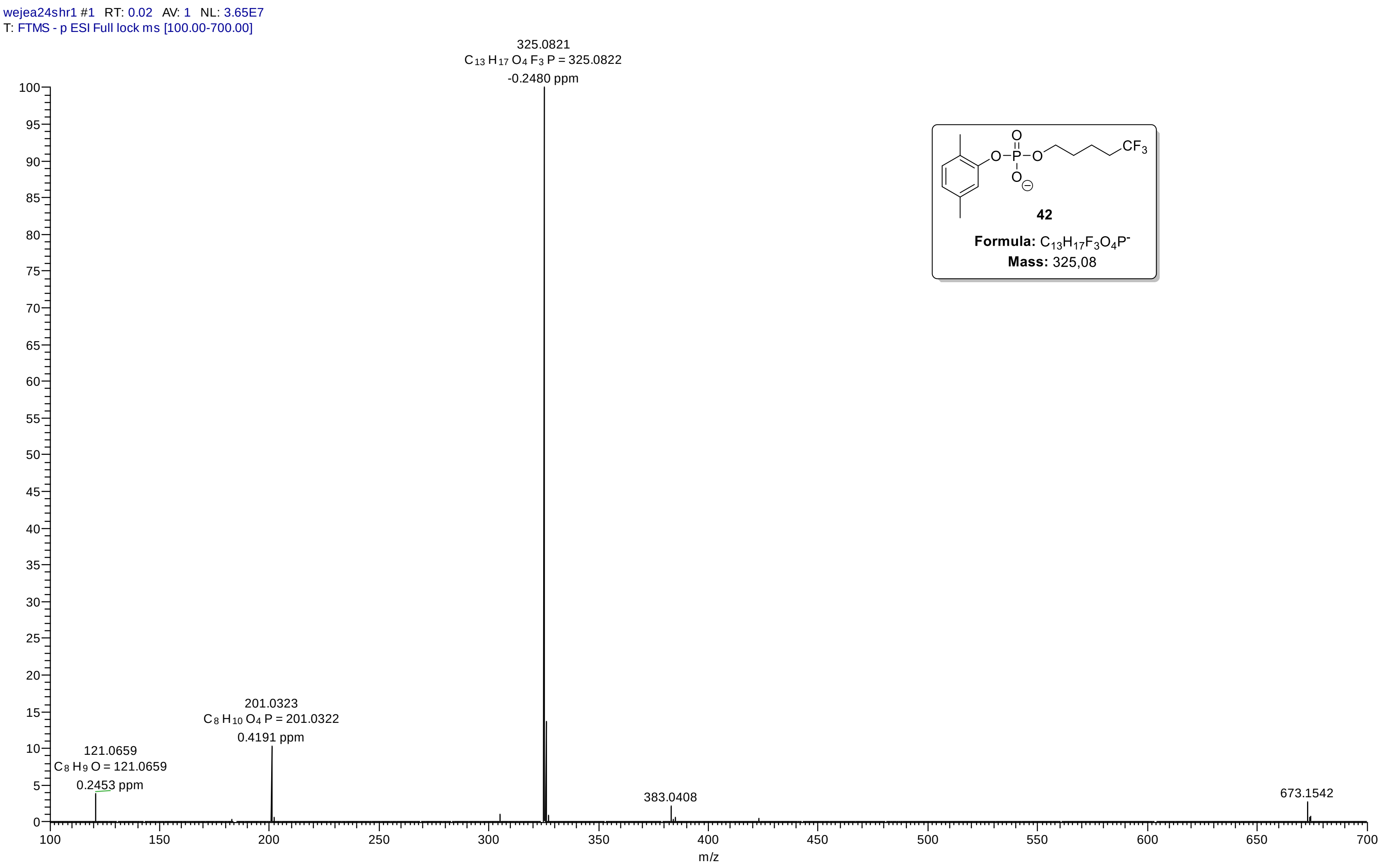

42

Formula: $C_{13}H_{17}F_3O_4P^-$
Mass: 325,08

HRMS (ESI) Analysis of compound 43: 3,4-dimethoxyphenyl (5,5,5-trifluoropentyl) phosphate

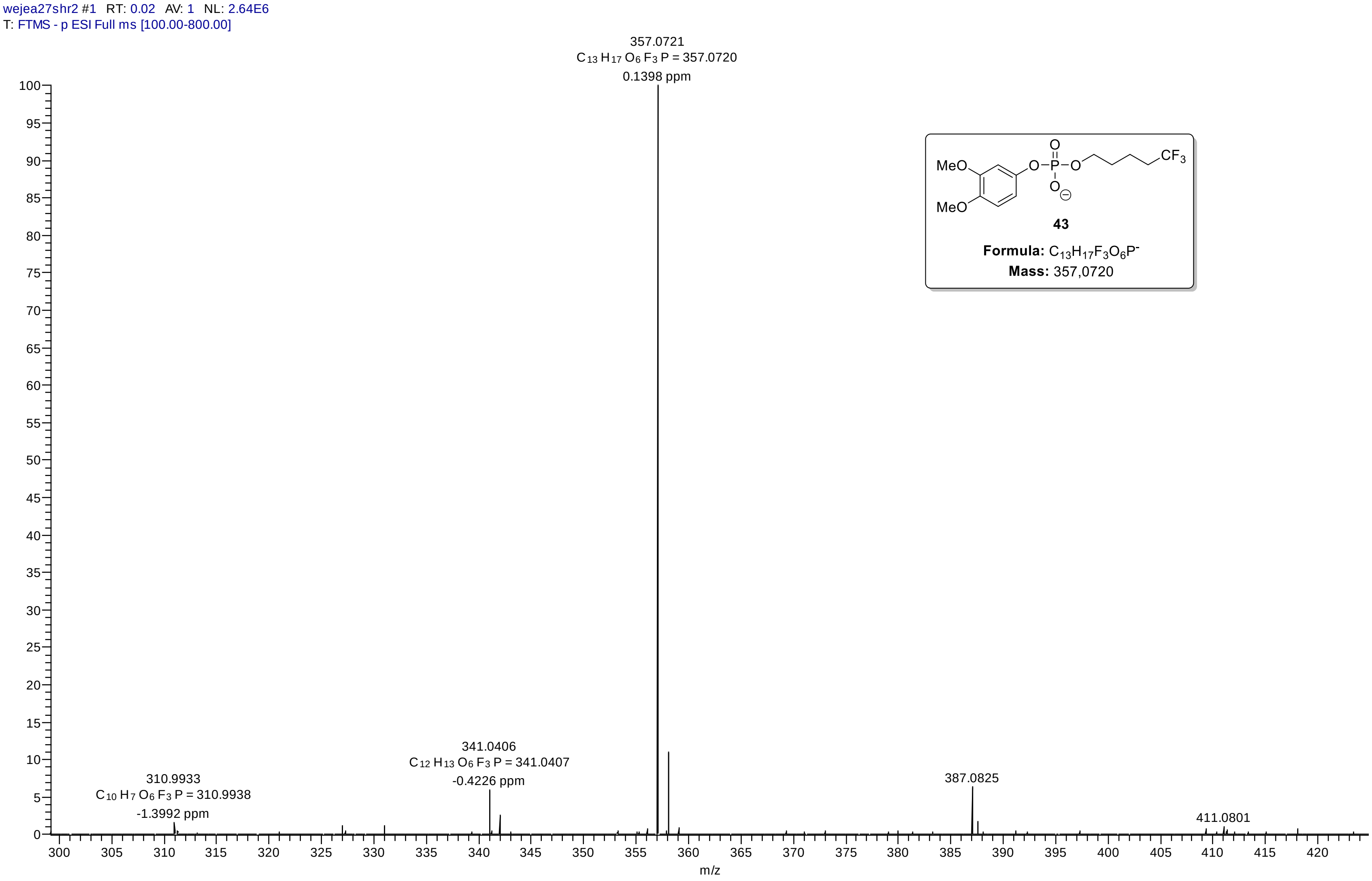

HRMS (ESI) Analysis of compound 44: 4-(trifluoromethyl)phenyl (5,5,5-trifluoropentyl) phosphate

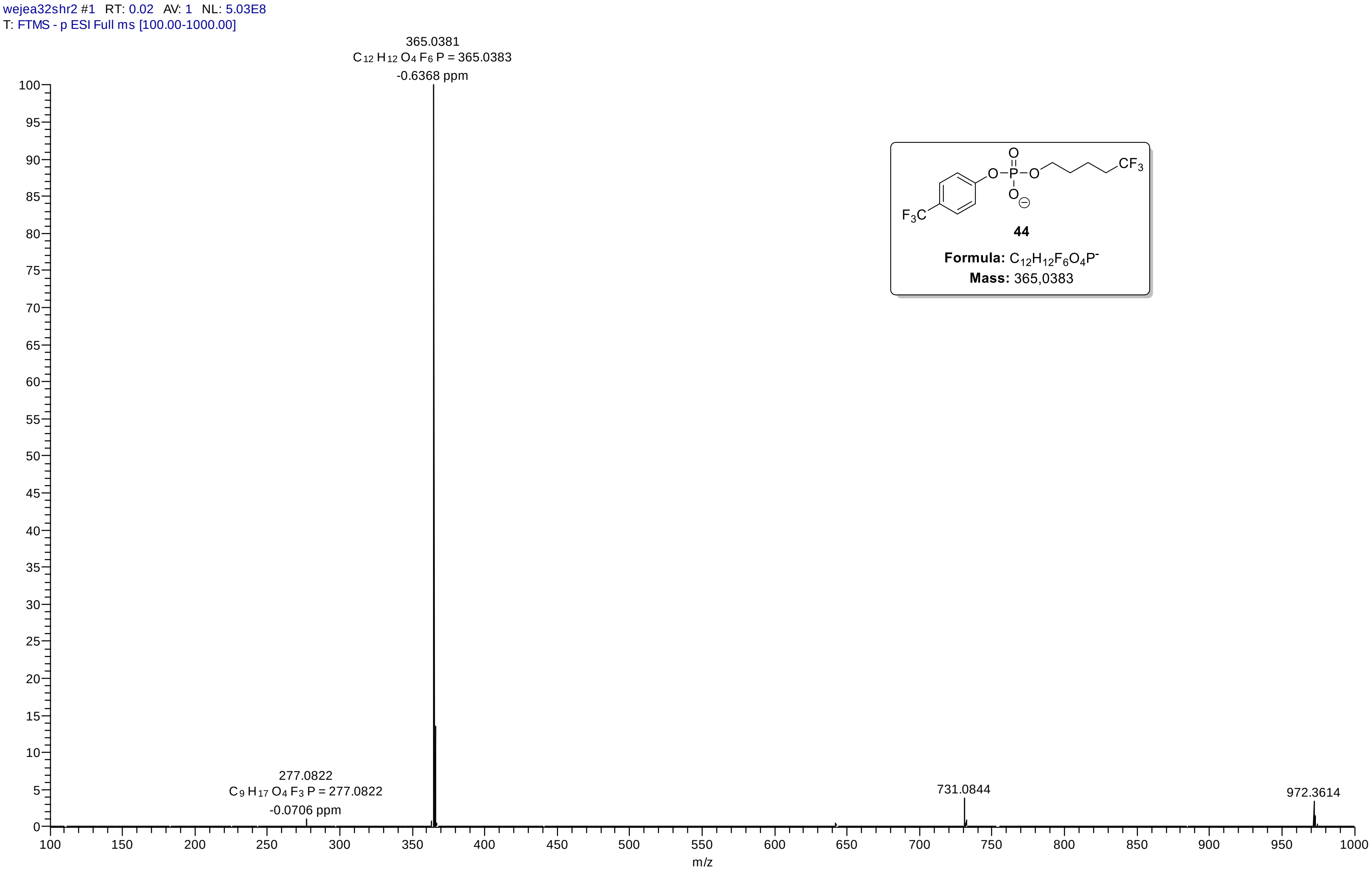

HRMS (ESI) Analysis of compound 45: benzo[d][1,3]dioxol-5-yl (5,5,5-trifluoropentyl) phosphate

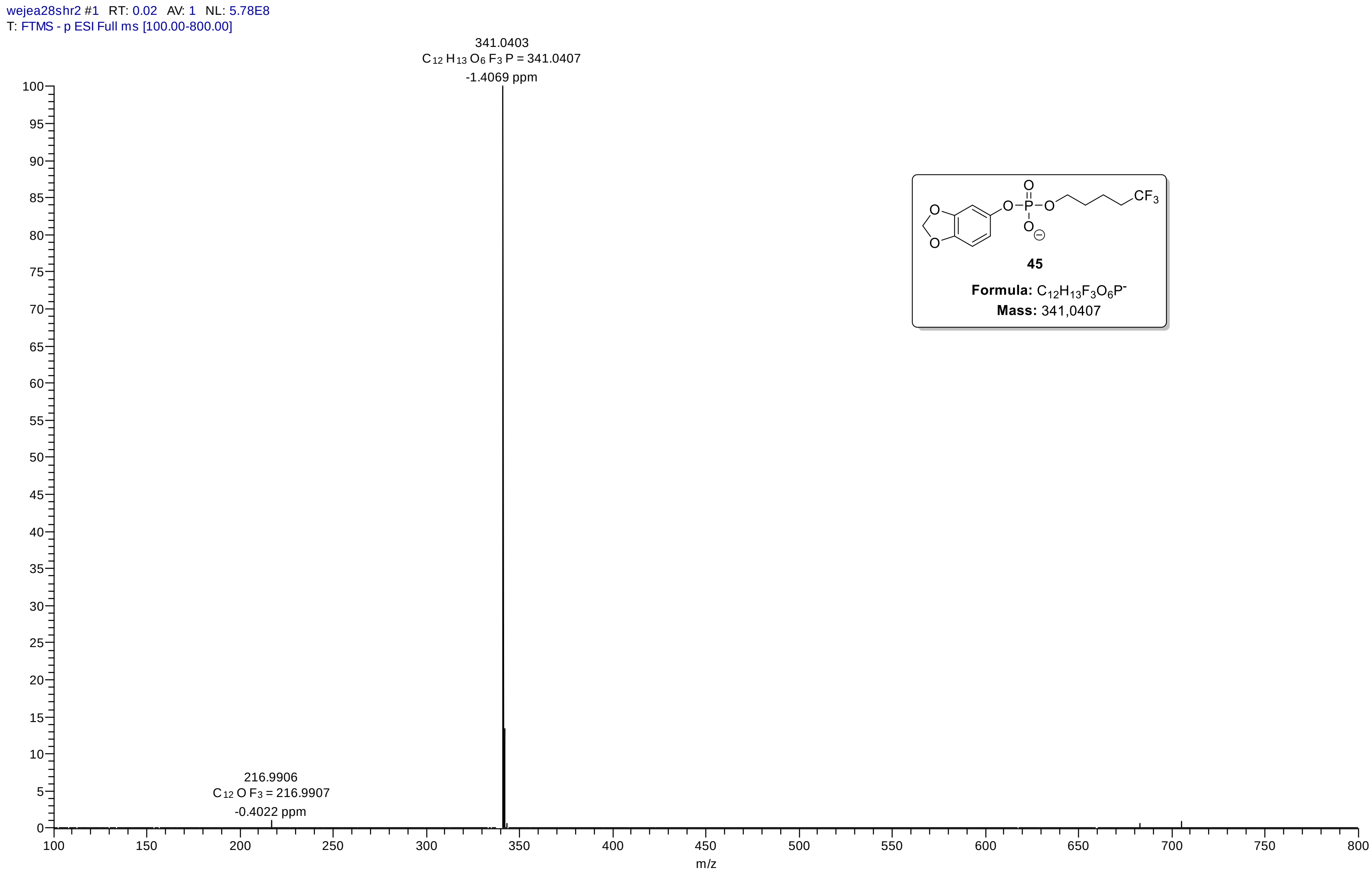

45

Formula: $C_{12}H_{13}F_3O_6P^-$
Mass: 341,0407

HRMS (ESI) Analysis of compound 46: 4-ethynylphenyl (5,5,5-trifluoropentyl) phosphate

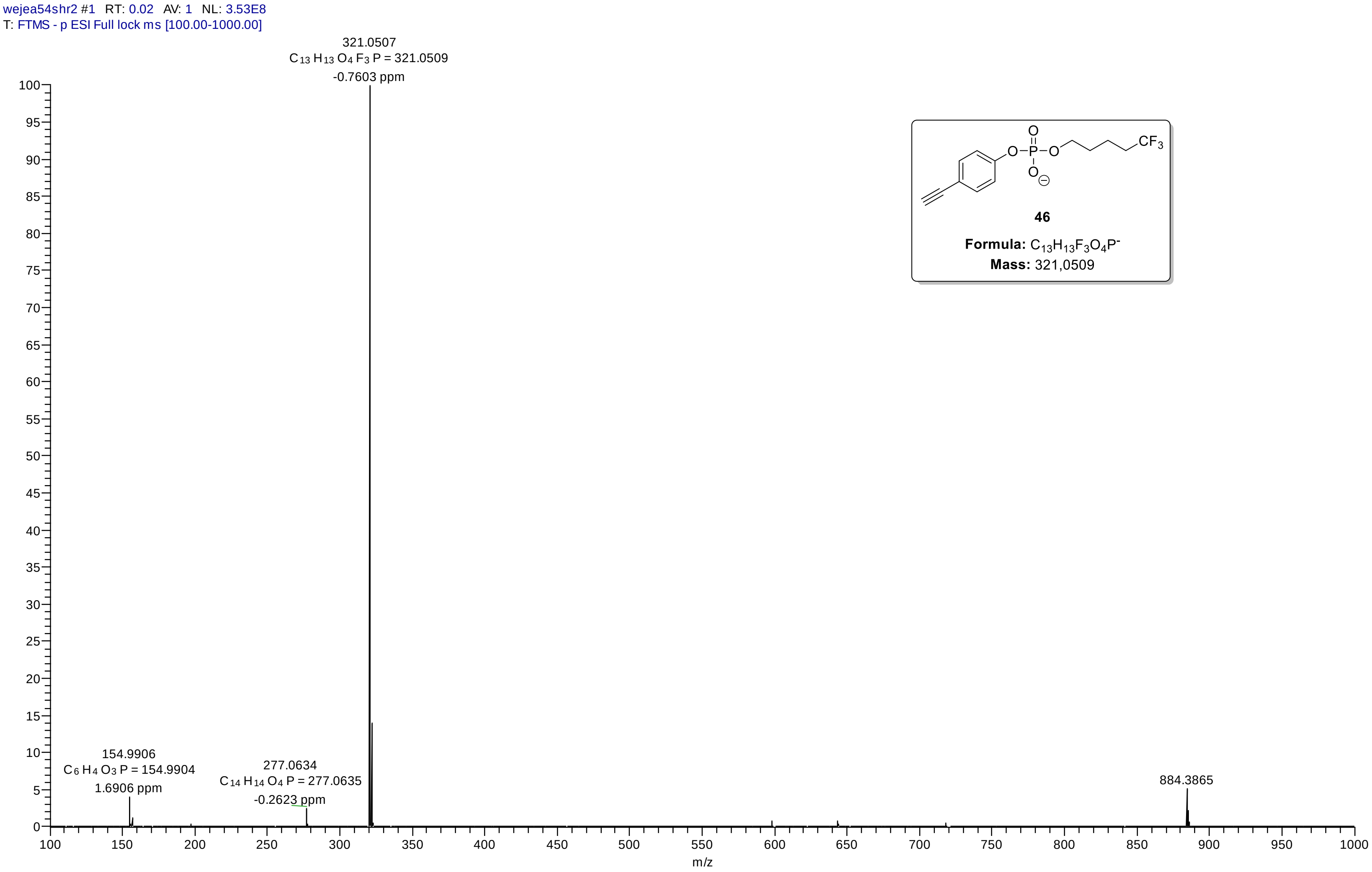

HRMS (ESI) Analysis of compound 47: 3-bromophenyl (5,5,5-trifluoropentyl) phosphate

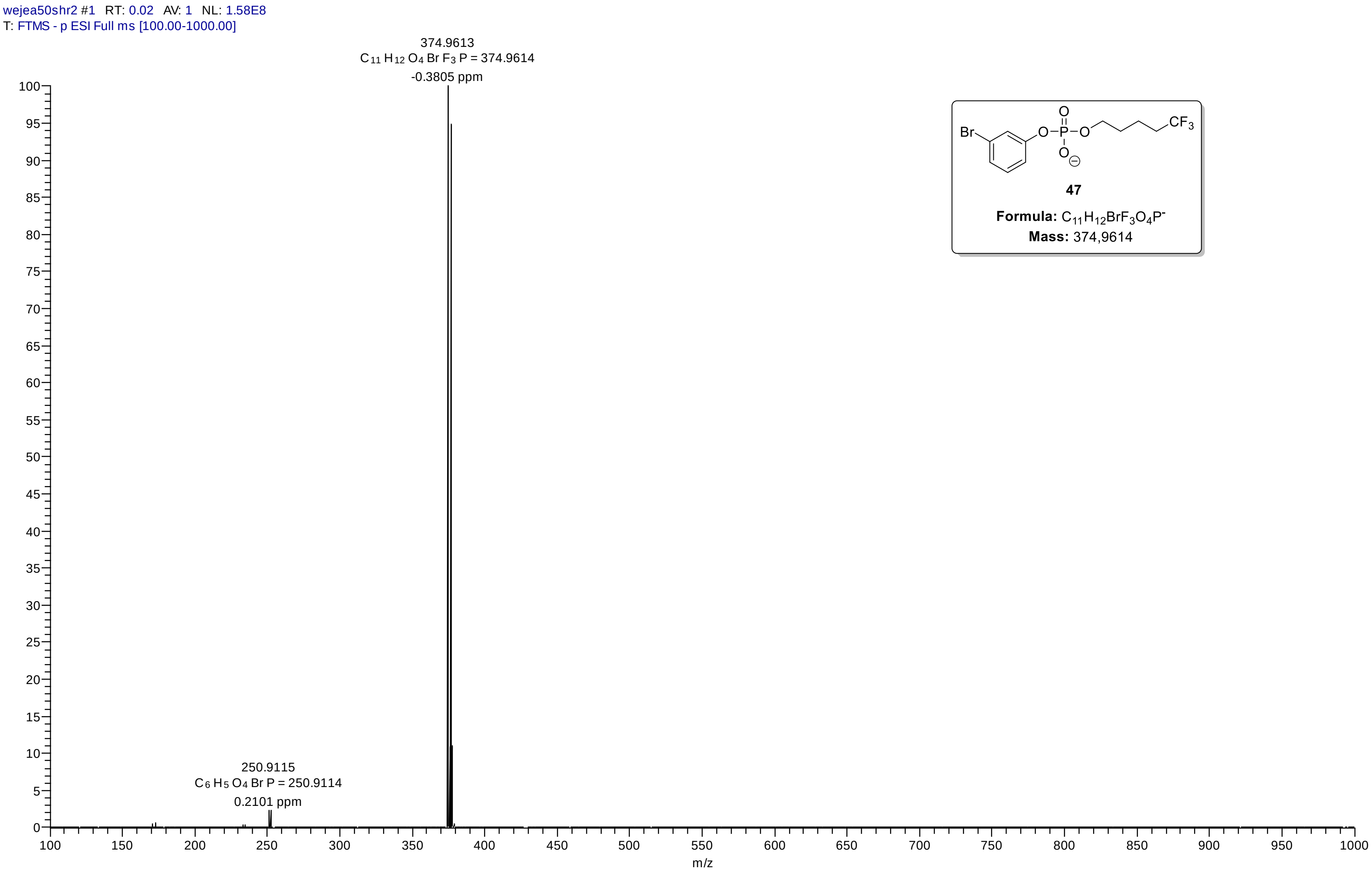

HRMS (ESI) Analysis of compound 48:4-chlorophenyl (5,5,5-trifluoropentyl) phosphate

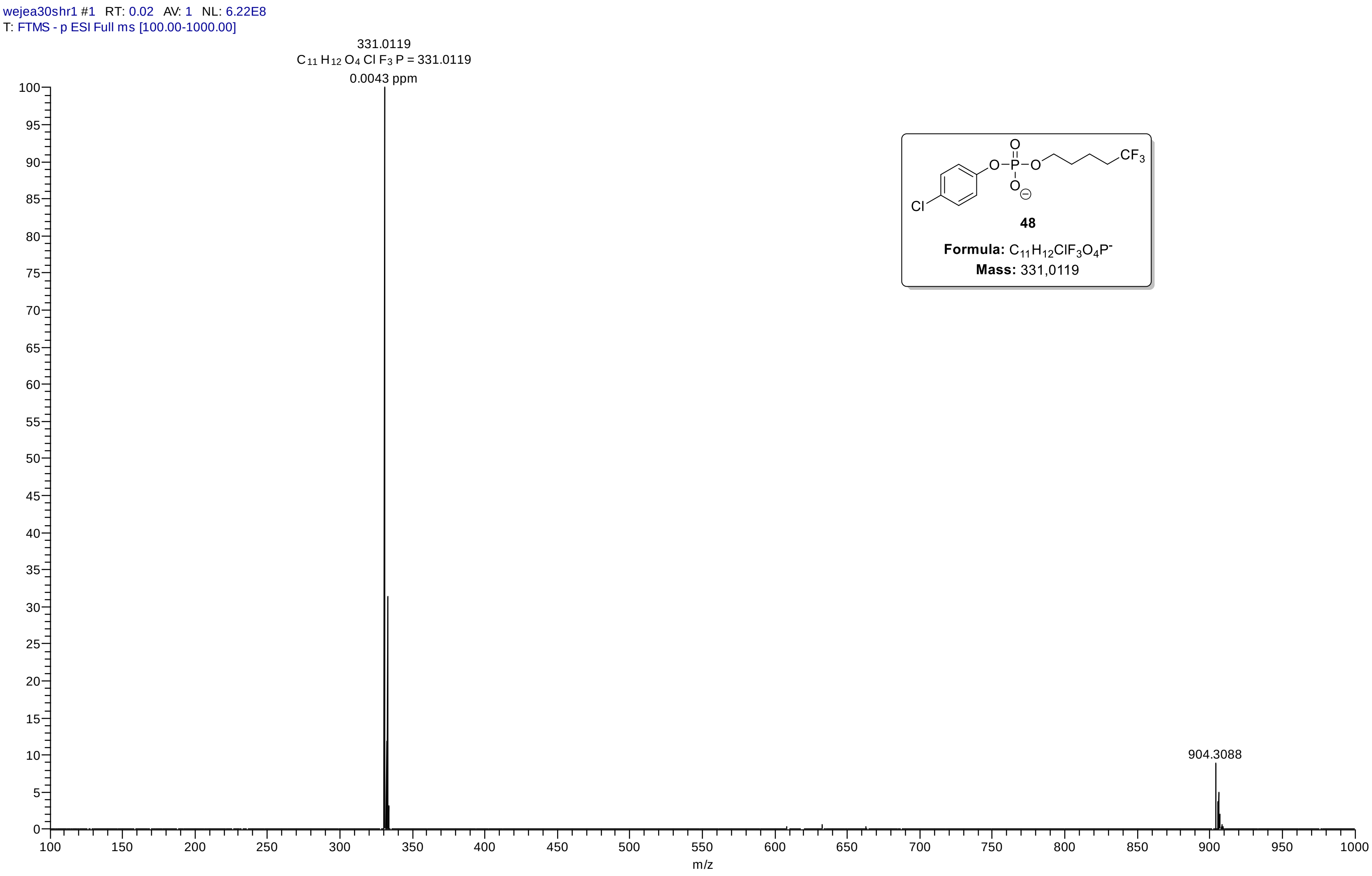

HRMS (ESI) Analysis of compound 49, naphthalen-2-yl (5,5,5-trifluoropentyl) phosphate

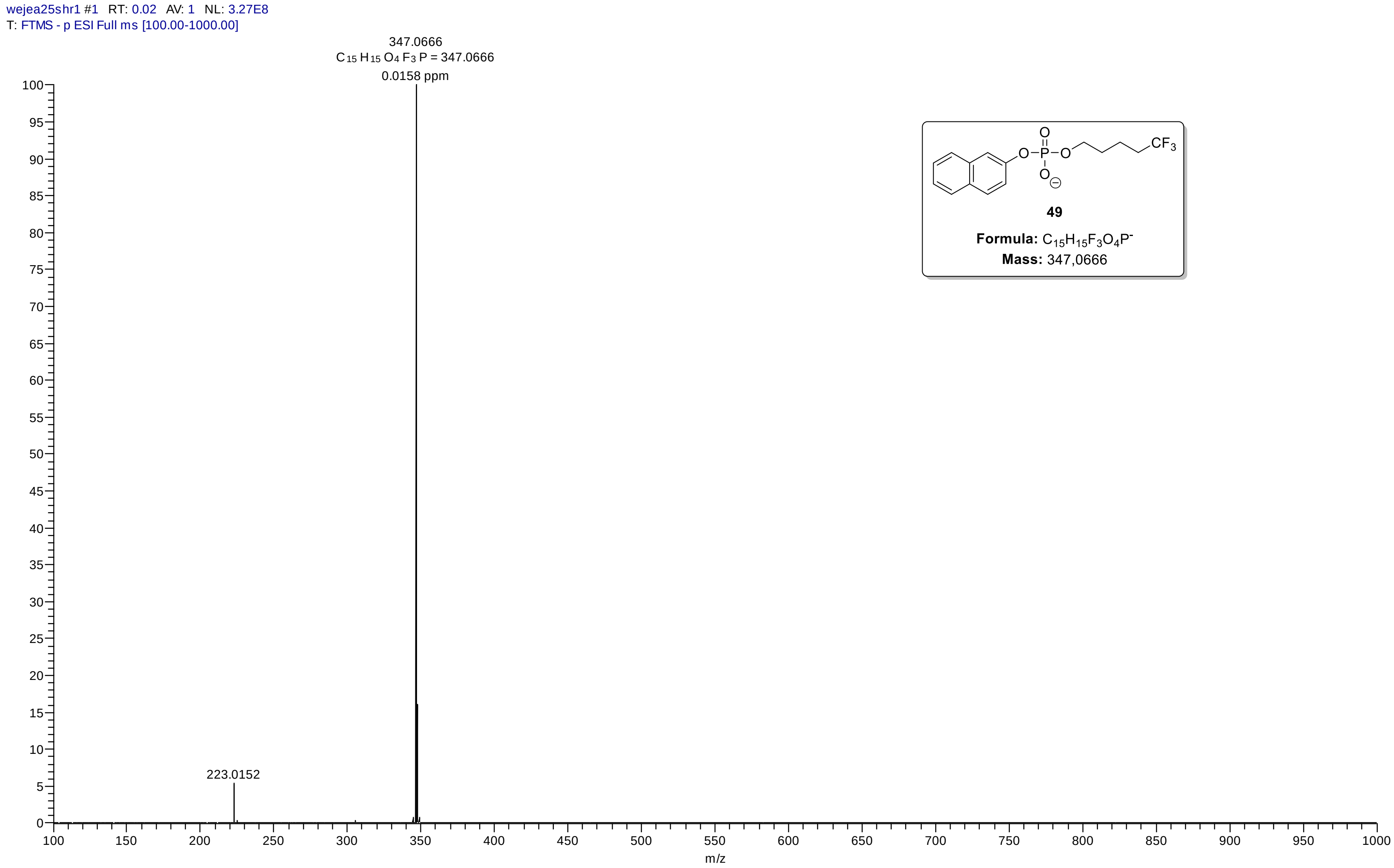

49

Formula: $C_{15}H_{15}F_3O_4P^-$

Mass: 347,0666

HRMS (ESI) Analysis of compound 50: 1H-indol-5-yl (5,5,5-trifluoropentyl) phosphate

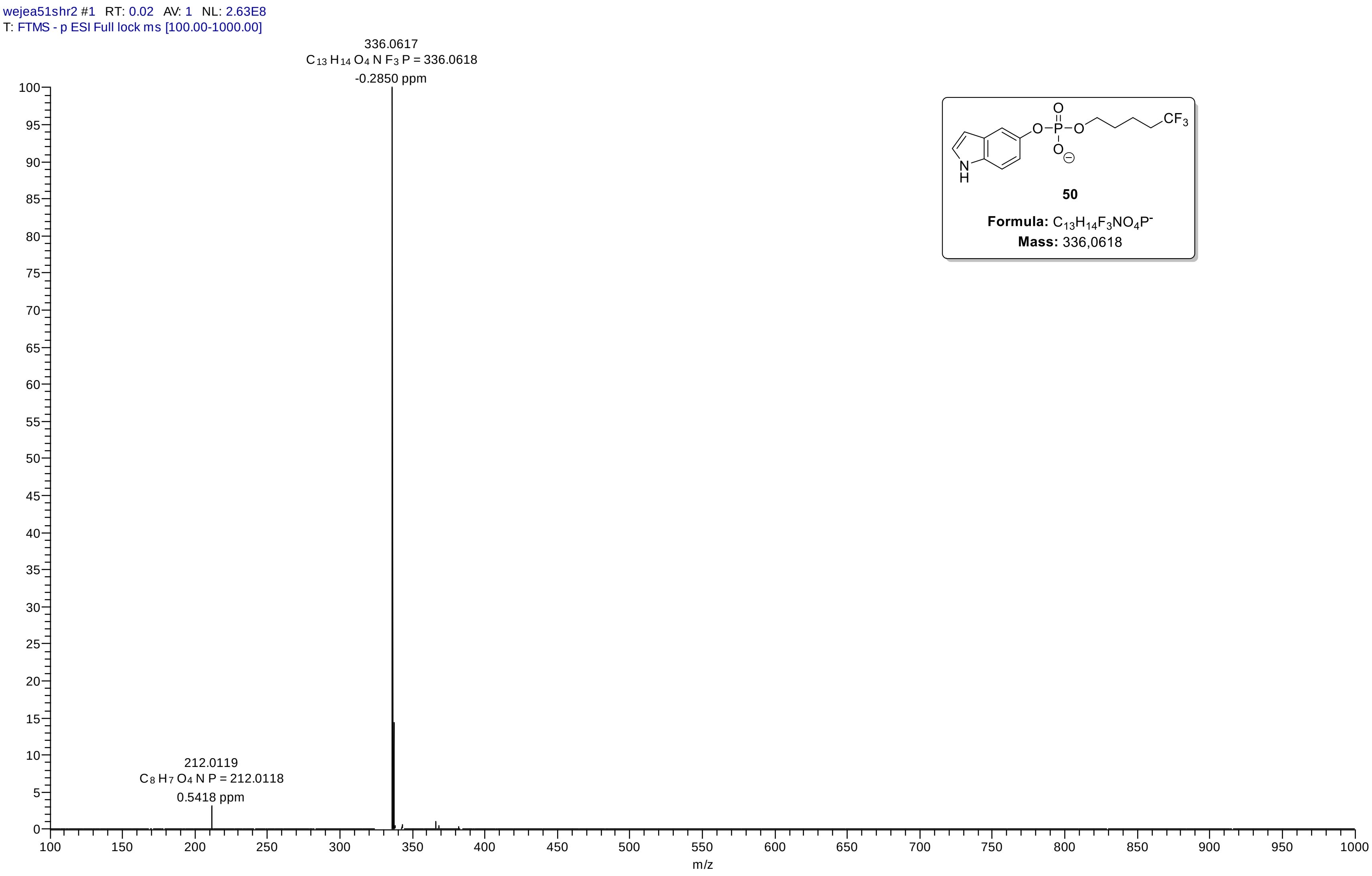

50

Formula: $C_{13}H_{14}F_3NO_4P^-$
Mass: 336,0618

HRMS (ESI) Analysis of compound 51: pyren-2-yl (5,5,5-trifluoropentyl) phosphate

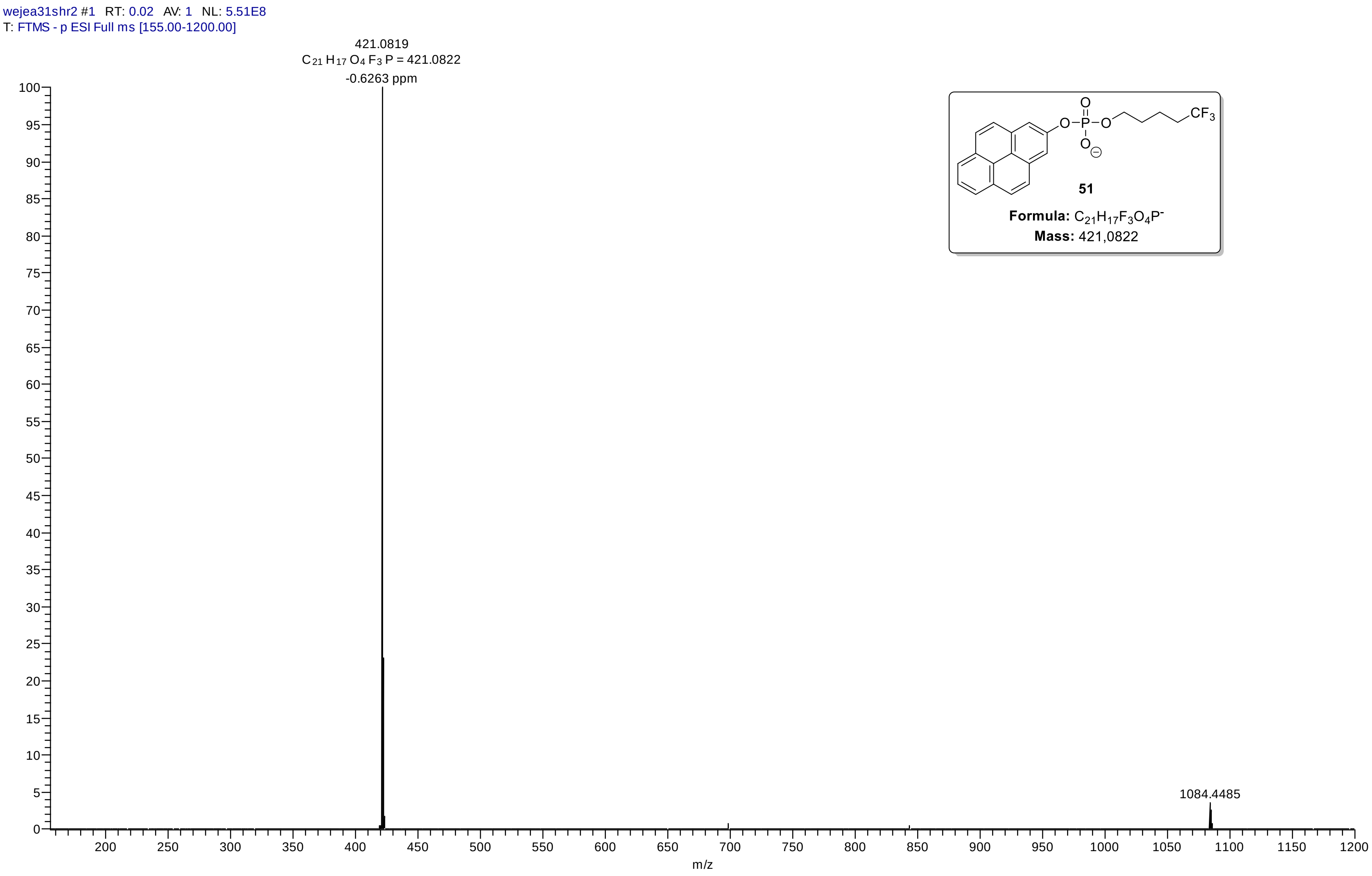

HRMS (ESI) Analysis of compound 52: Diphenylphosphate

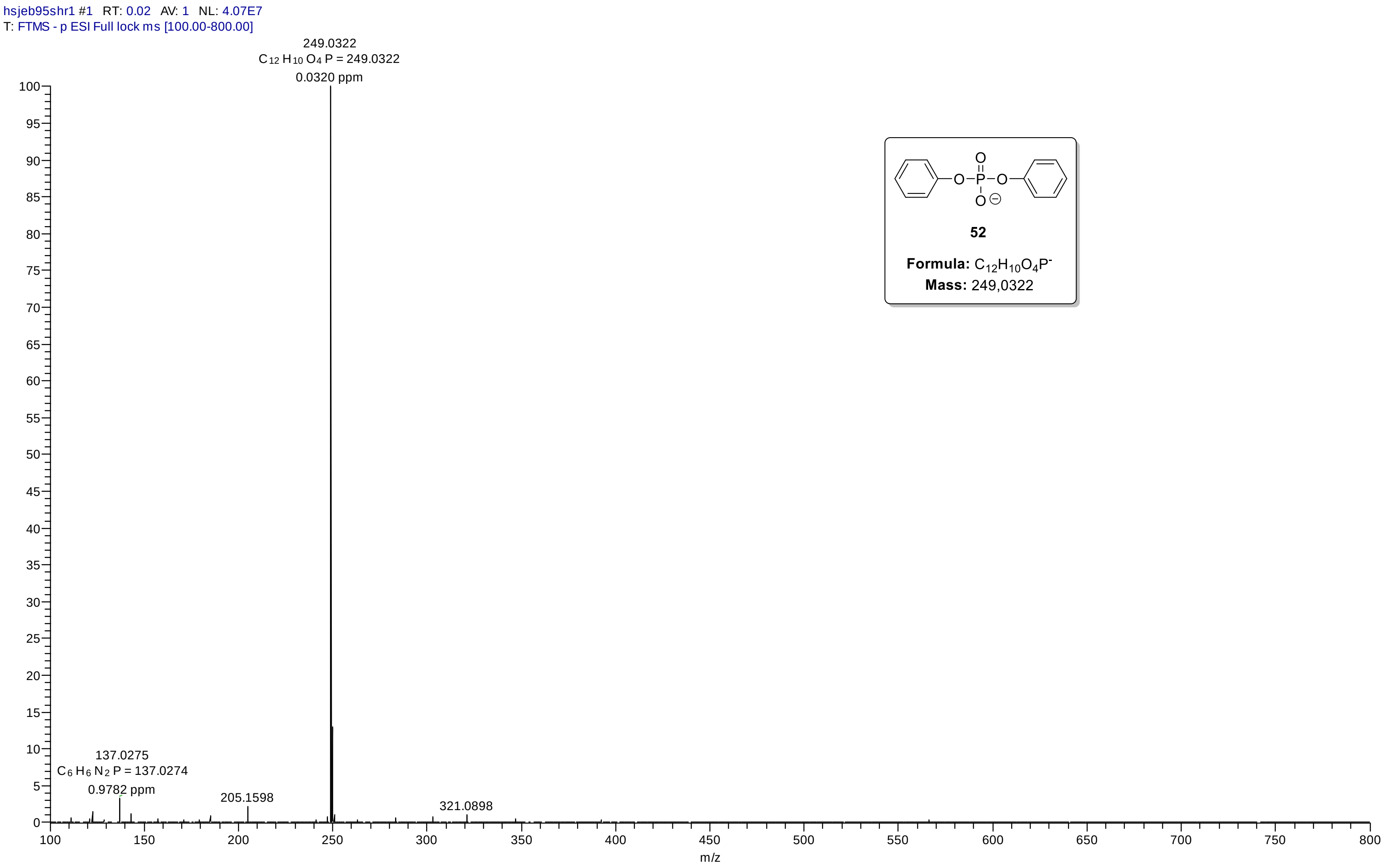

52

Formula: $C_{12}H_{10}O_4P^-$
Mass: 249,0322

HRMS (ESI) Analysis of compound 53: Pentyl-phenylphosphate

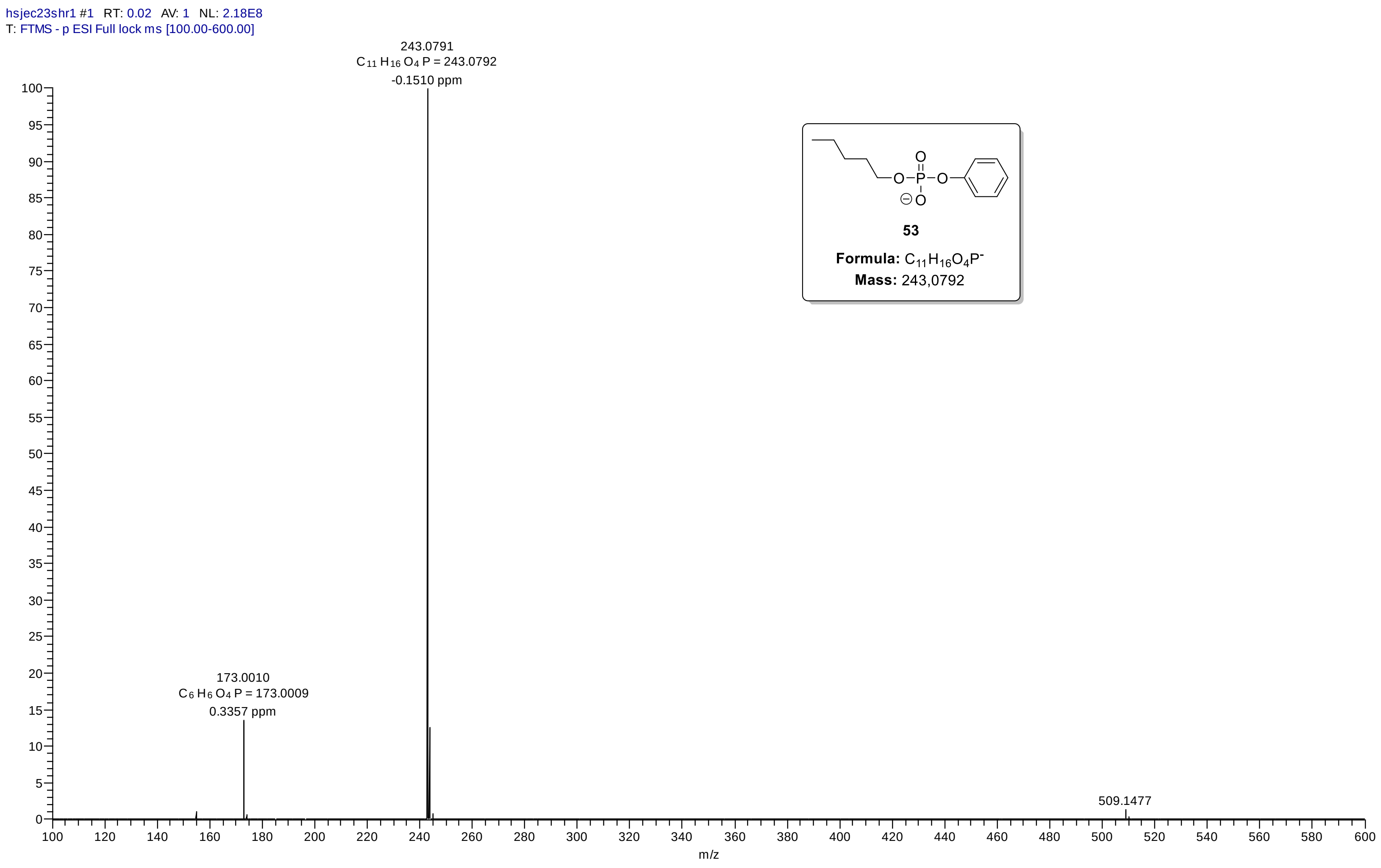

53

Formula: $C_{11}H_{16}O_4P^-$
Mass: 243,0792

HRMS (ESI) Analysis of compound 54: Phenyl-phenylphosphonate

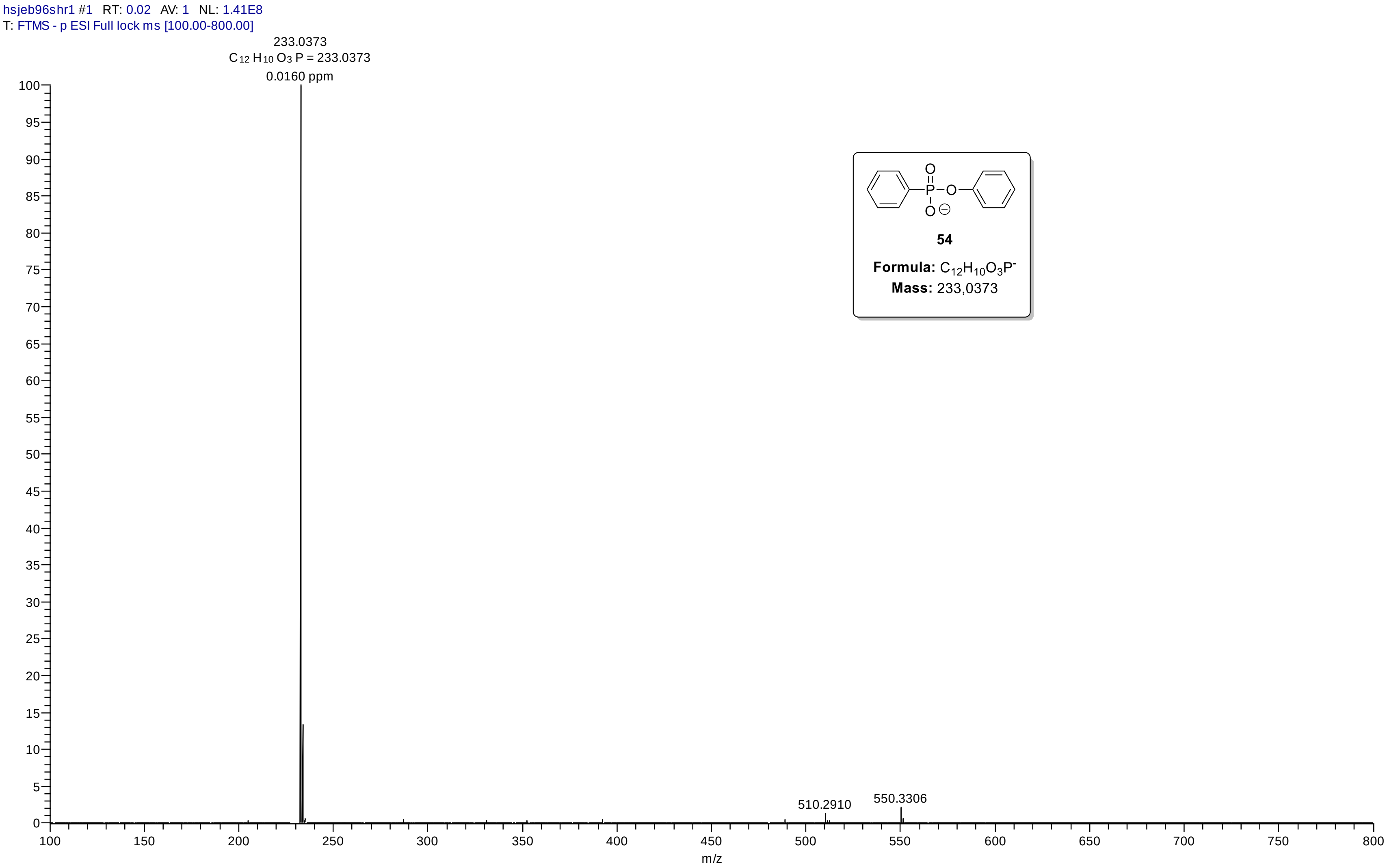

HRMS (ESI) Analysis of compound 55: Pent-4-yn-1-yl-phenylphosphate

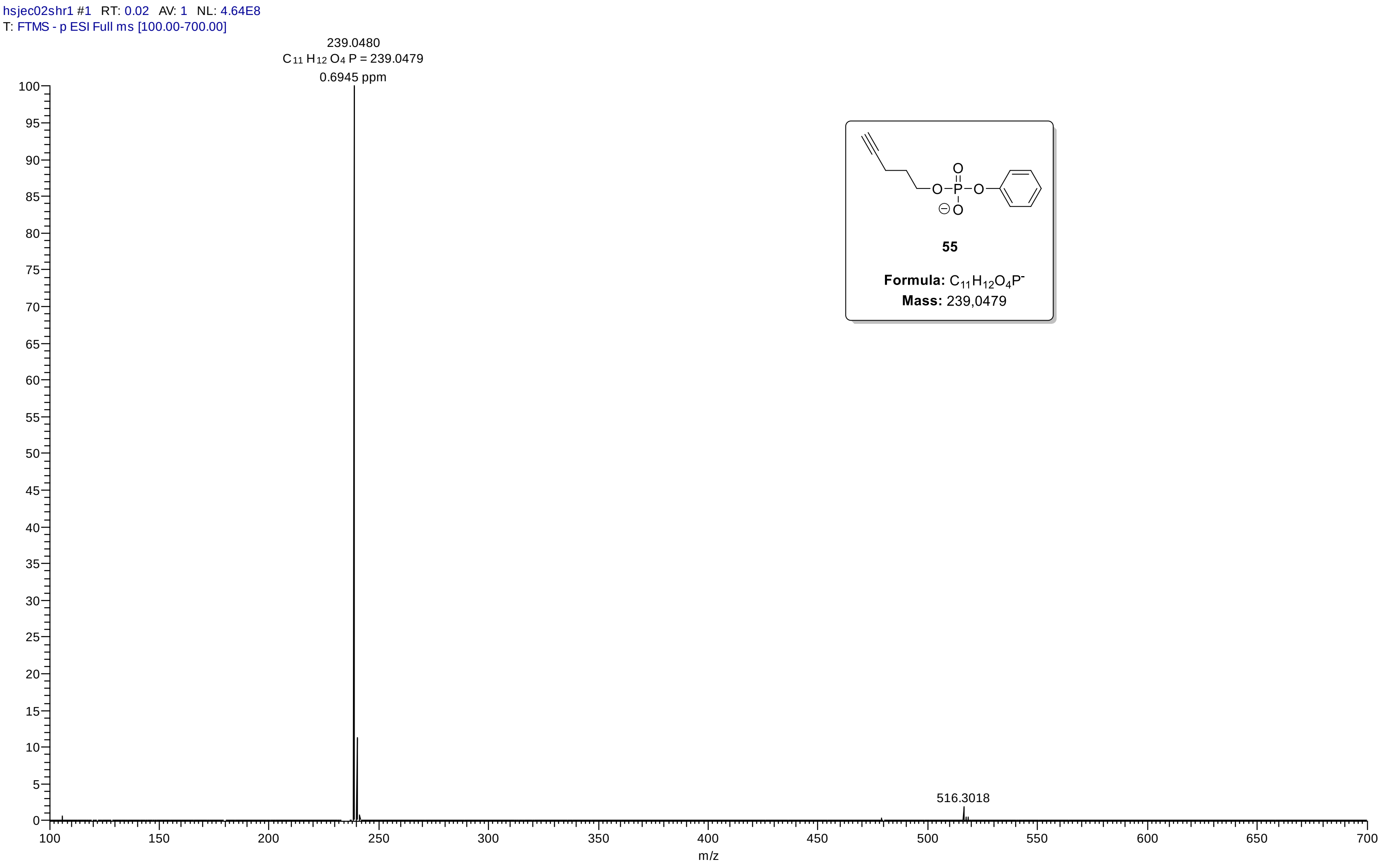

55

Formula: $C_{11}H_{12}O_4P^-$
Mass: 239,0479

HRMS (ESI) Analysis of compound 56: Isoprenol-phenylphosphate

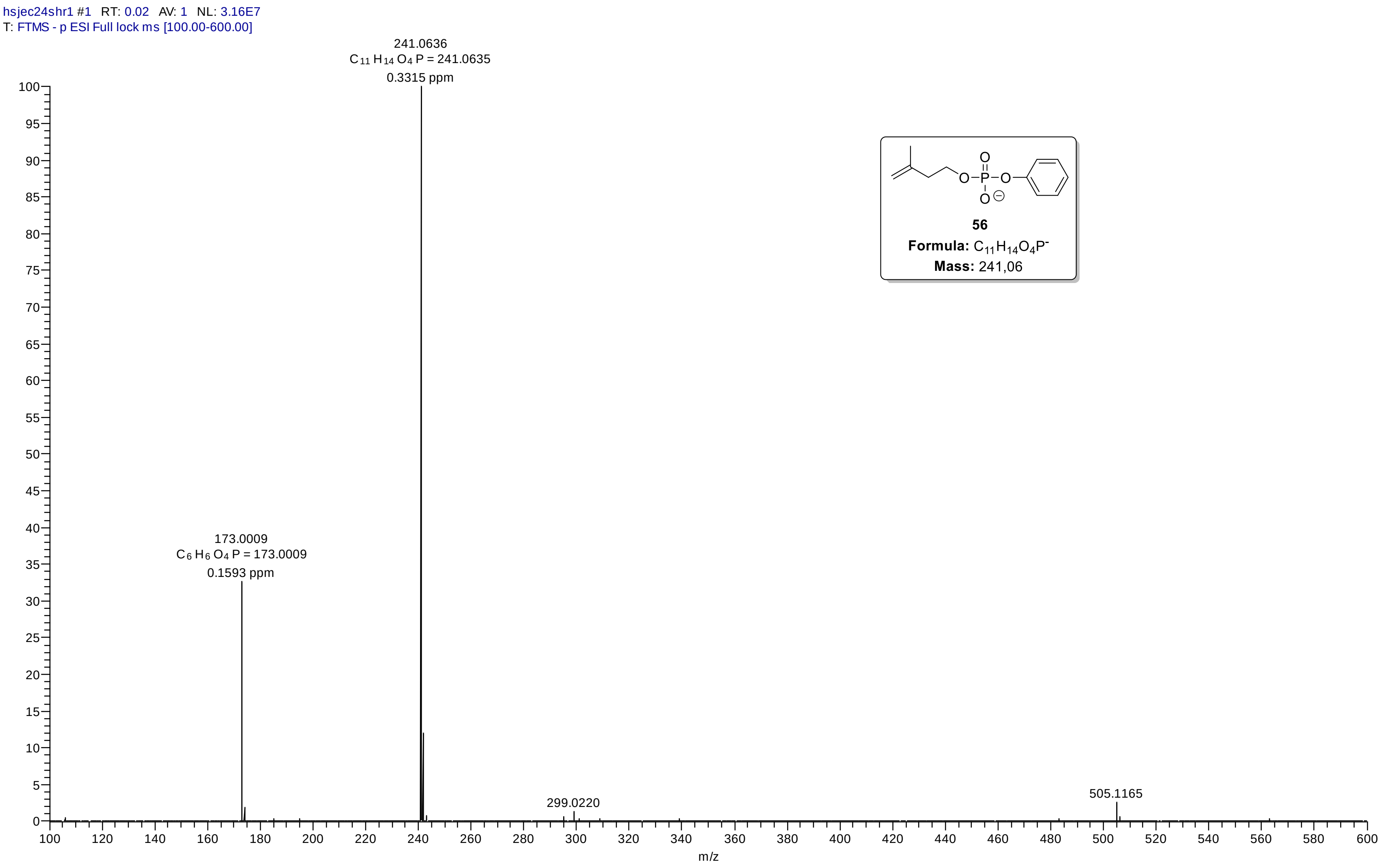

HRMS (ESI) Analysis of compound 57: Geranyl-phenylphosphate

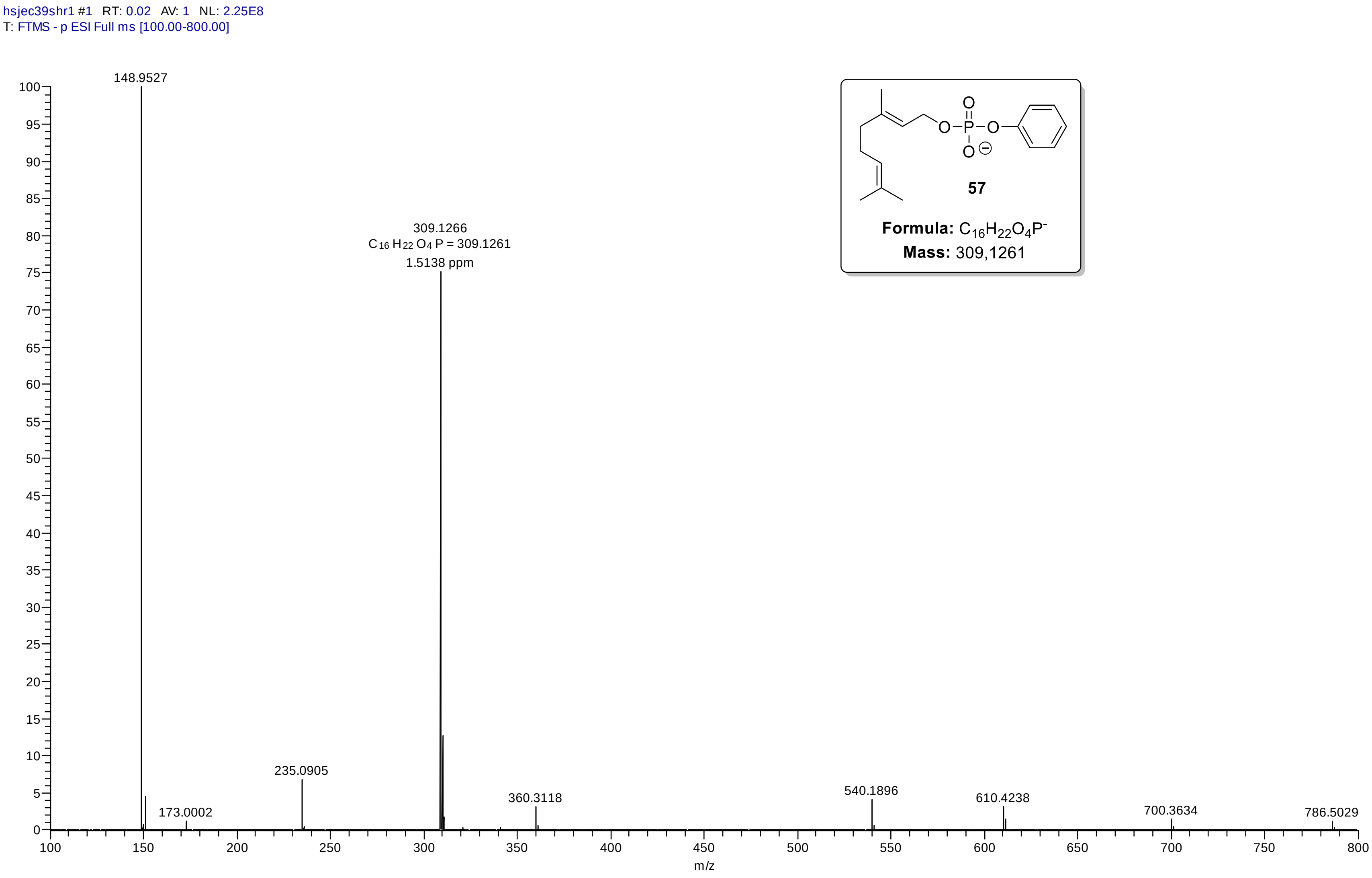

57

Formula: $C_{16}H_{22}O_4P^-$
Mass: 309,1261

HRMS (ESI) Analysis of compound 58: D4T-phenylpyrophosphate

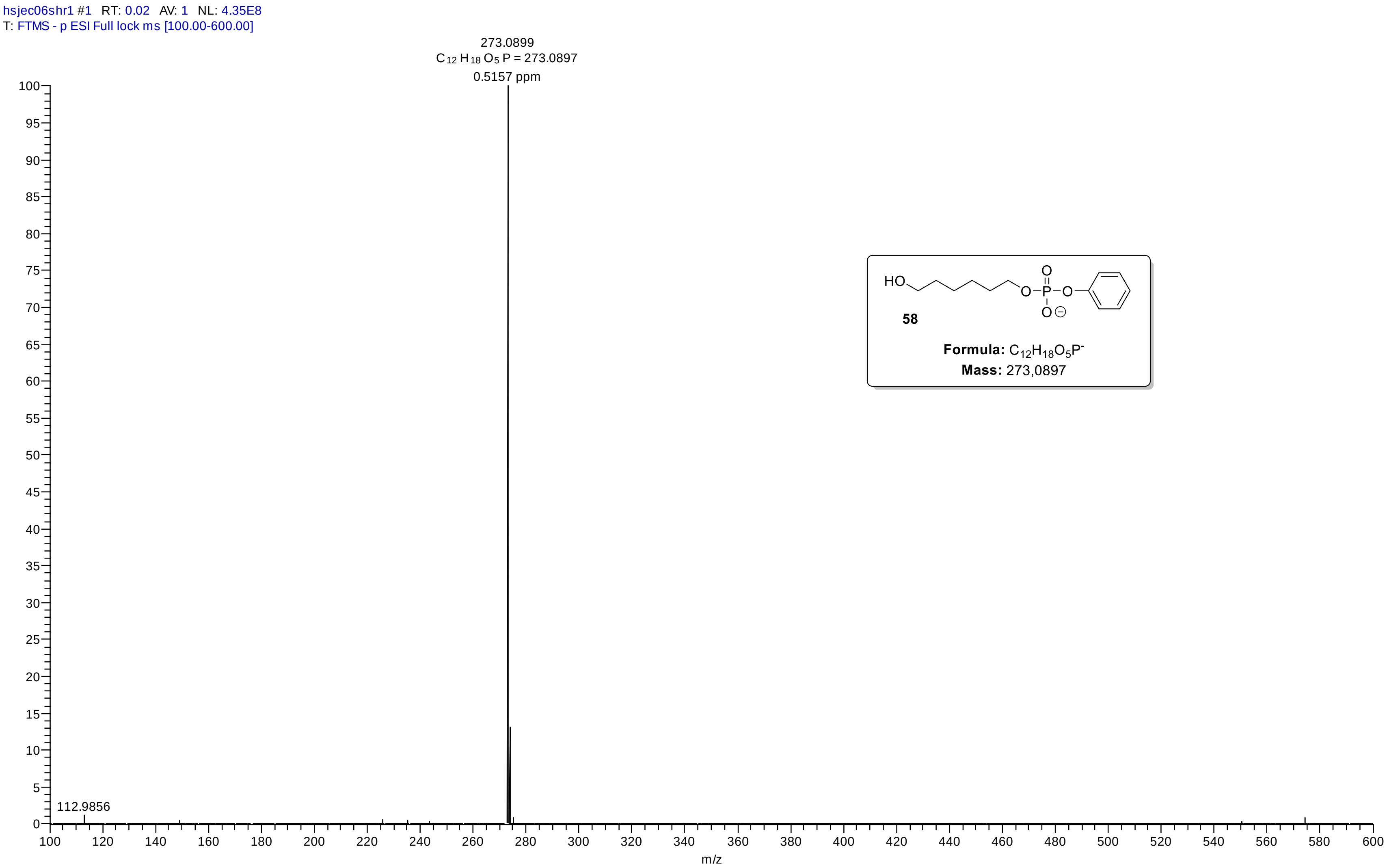

Formula: $C_{12}H_{18}O_5P^-$
Mass: 273,0897

HRMS (ESI) Analysis of compound 59: D4T-phenylphosphate

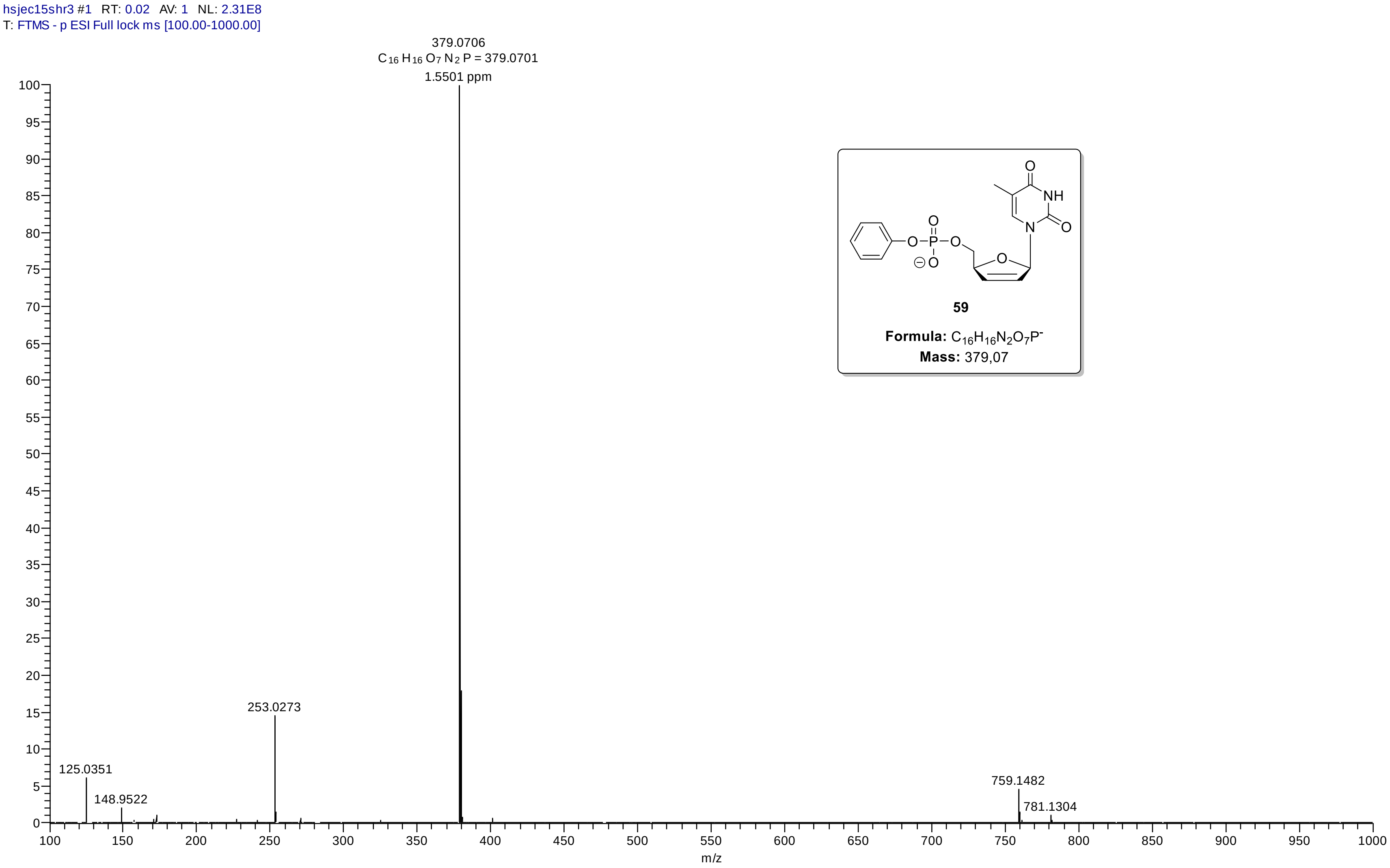

59

Formula: $C_{16}H_{16}N_2O_7P^-$

Mass: 379,07

HRMS (ESI) Analysis of compound 60: D4T-phenylpyrophosphate

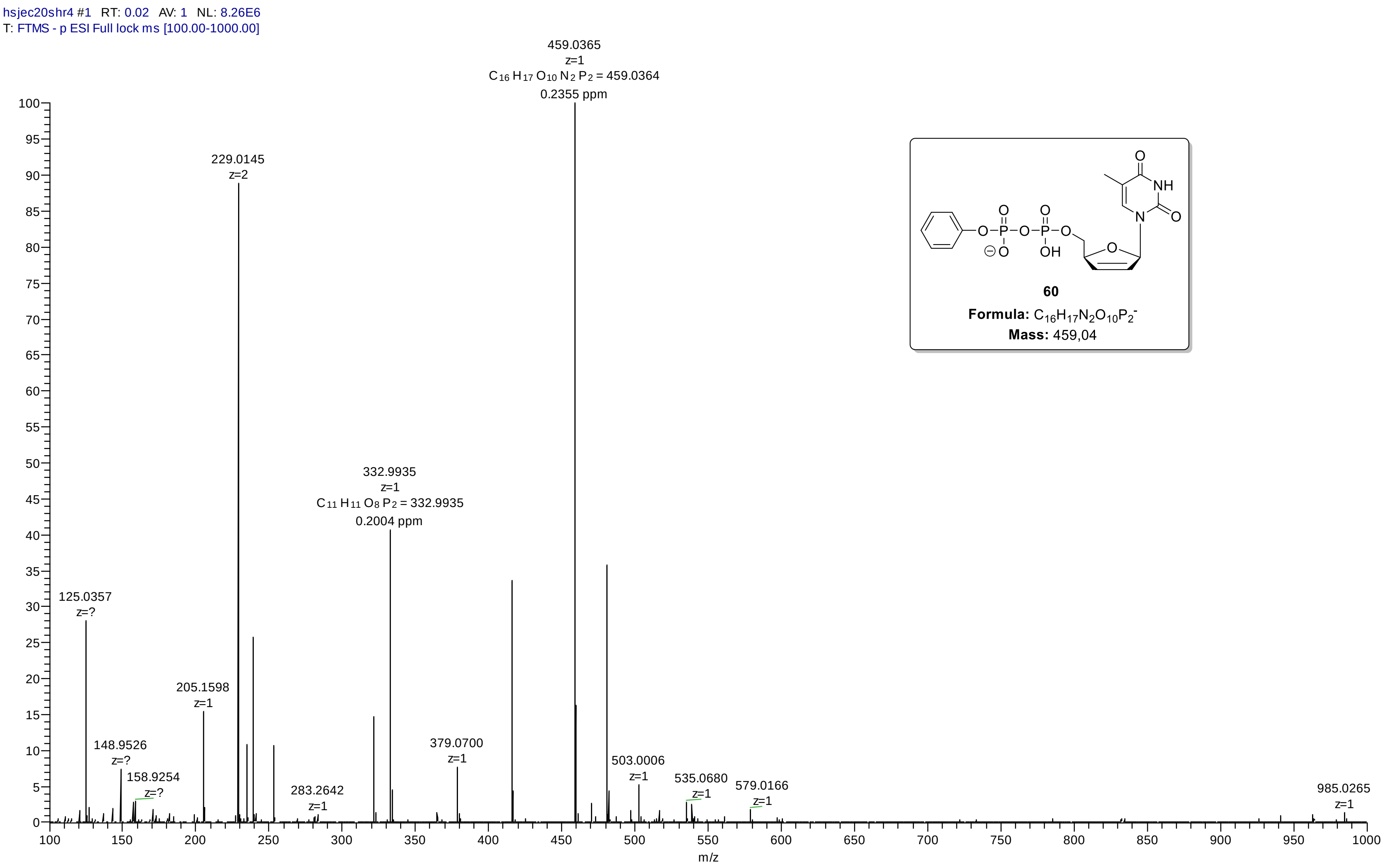

HRMS (ESI) Analysis of compound 61: Diphenylpyrophosphate

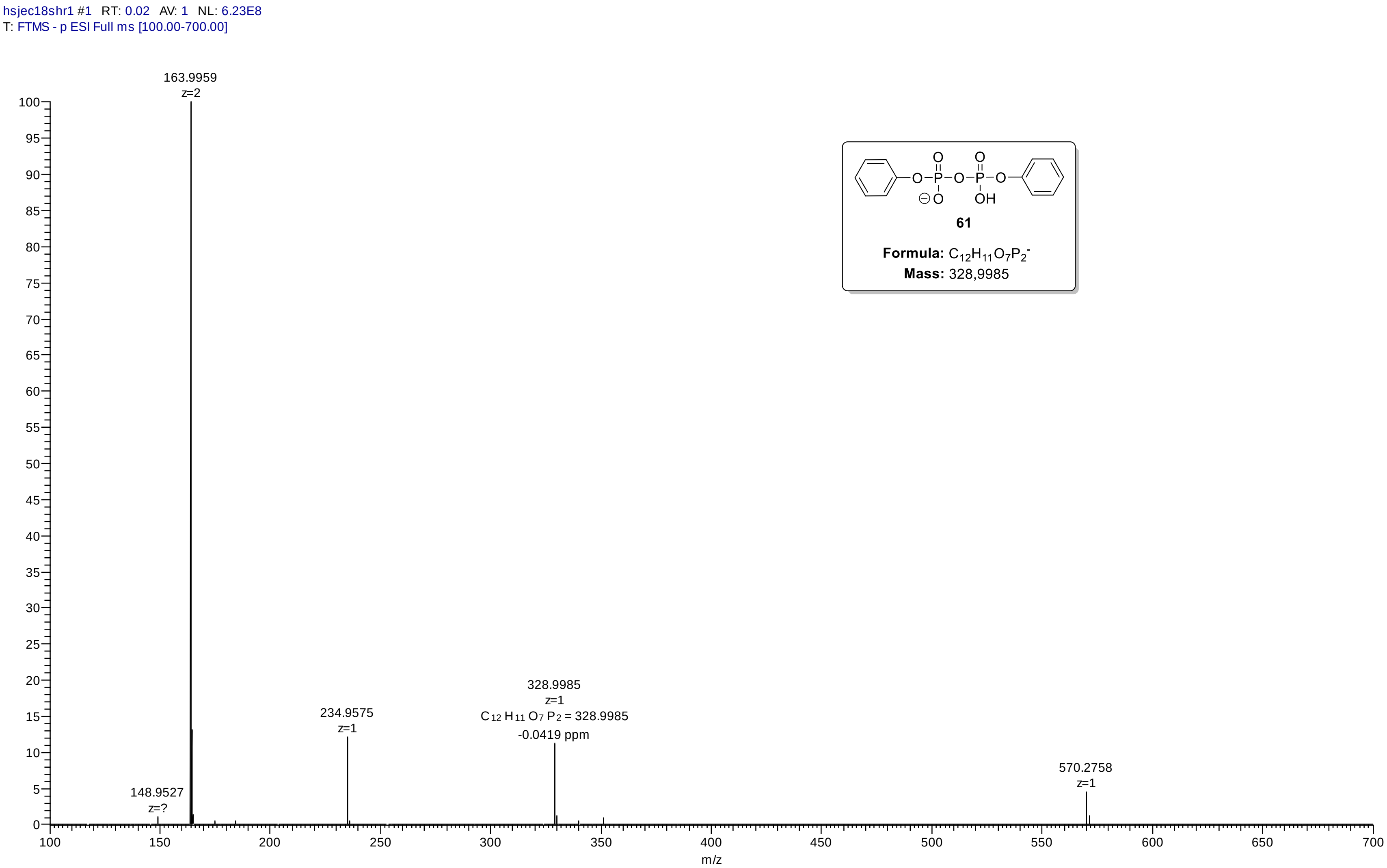

61

Formula: $C_{12}H_{11}O_7P_2^-$
Mass: 328,9985

HRMS (ESI) Analysis of compound 67: Pentylphosphate

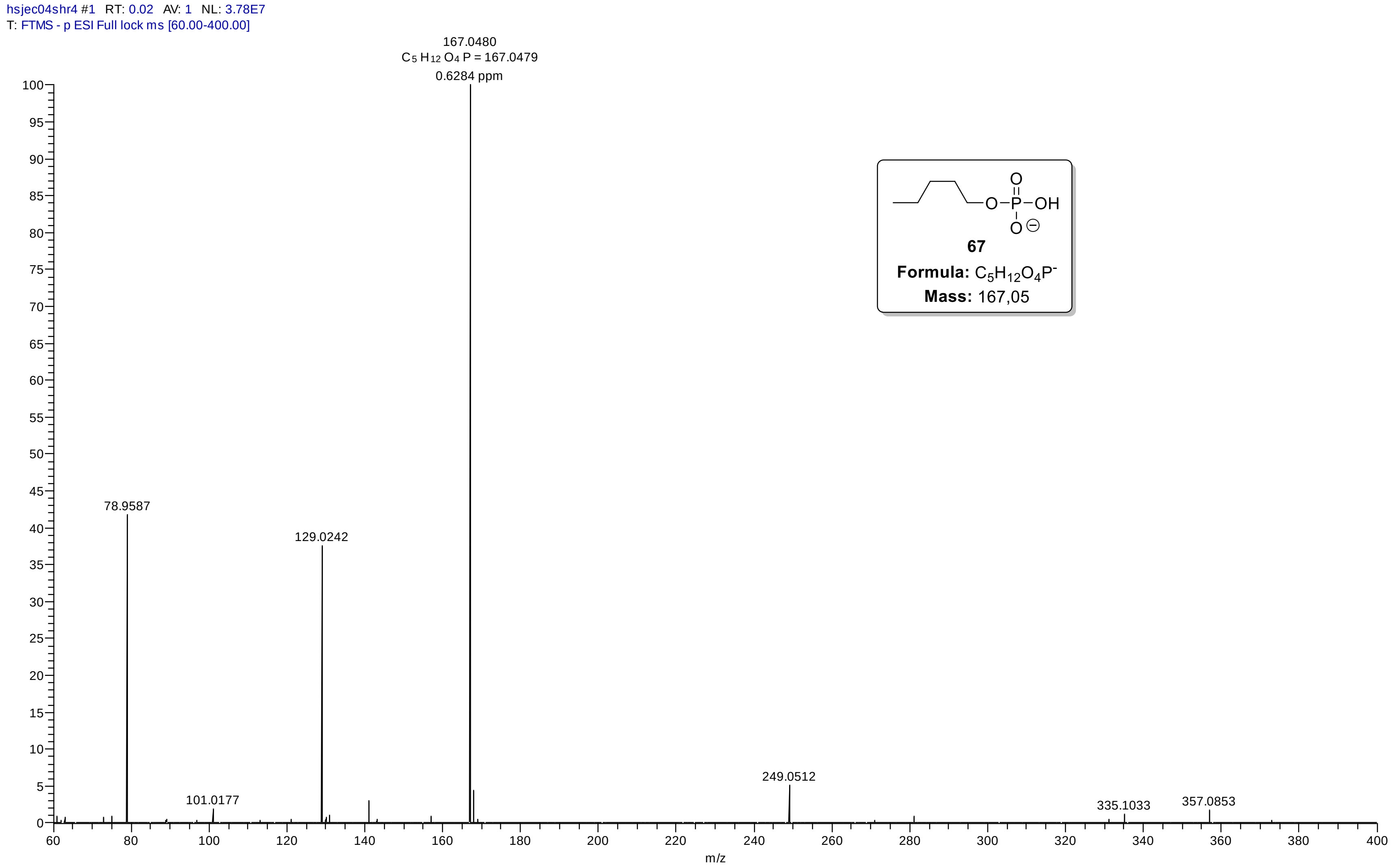

HRMS (ESI) Analysis of compound 70: Pentametaphosphate

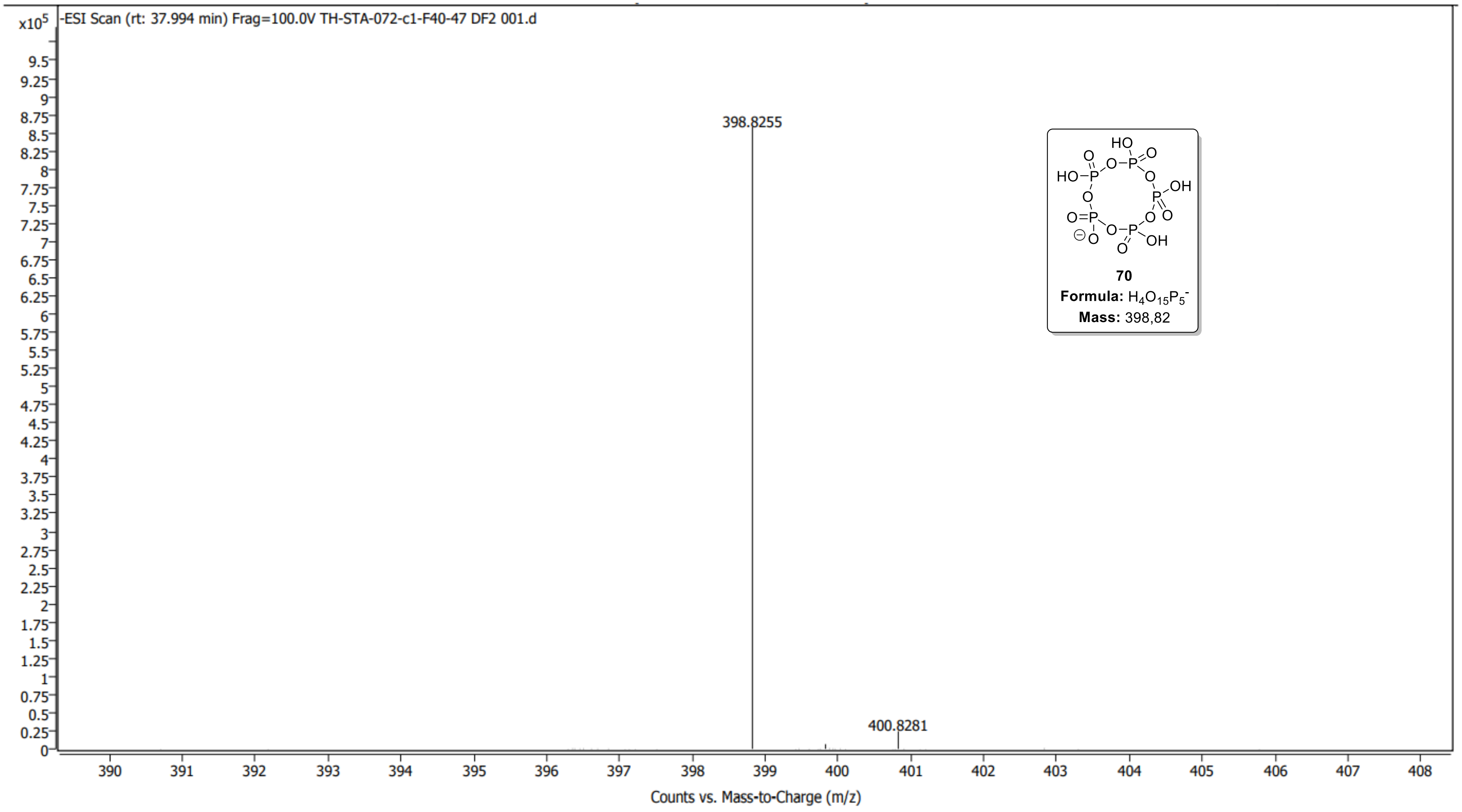

HRMS (ESI) Analysis of compound 71: Heptametaphosphate

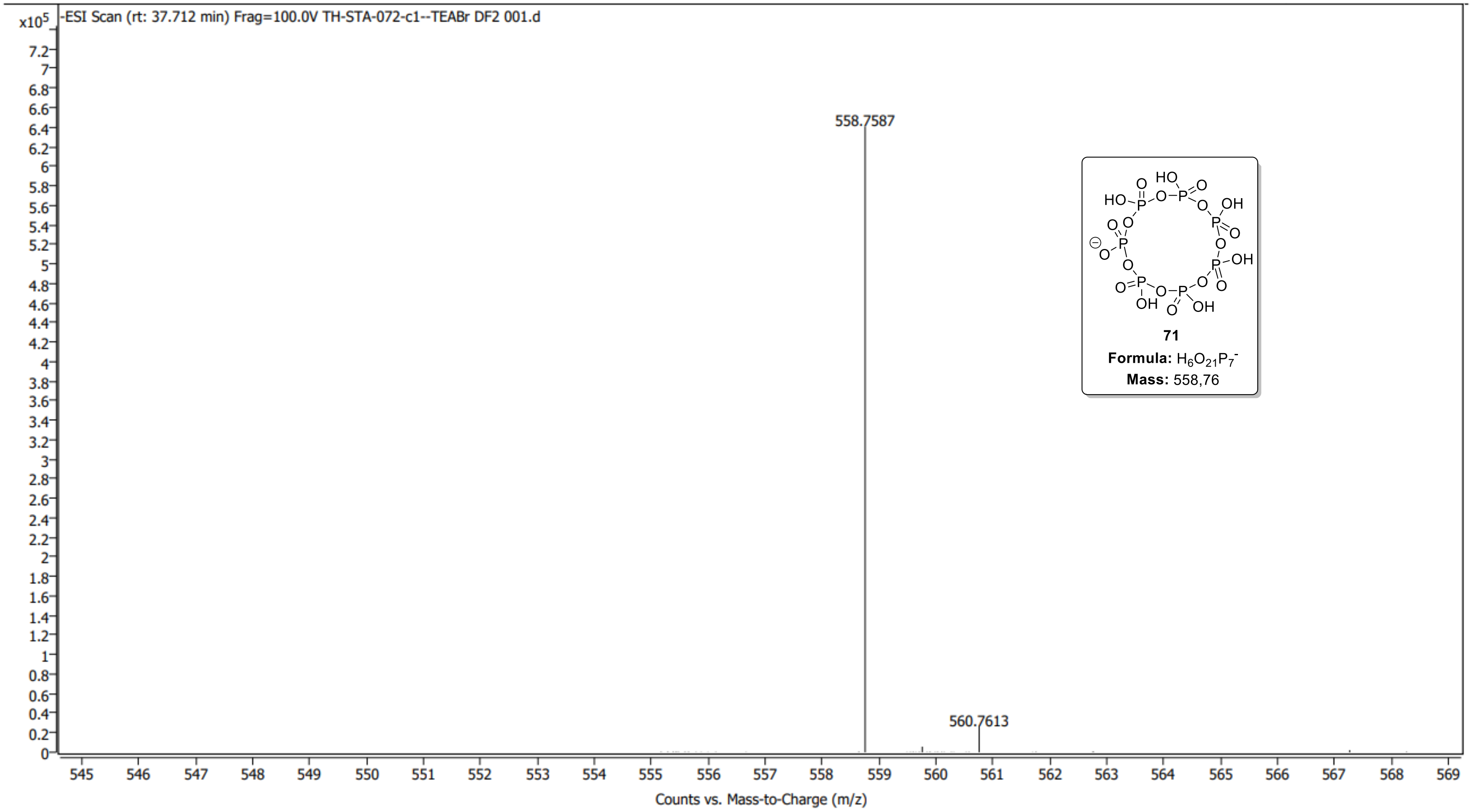

HRMS (ESI) Analysis of compound 72: Octametaphosphate

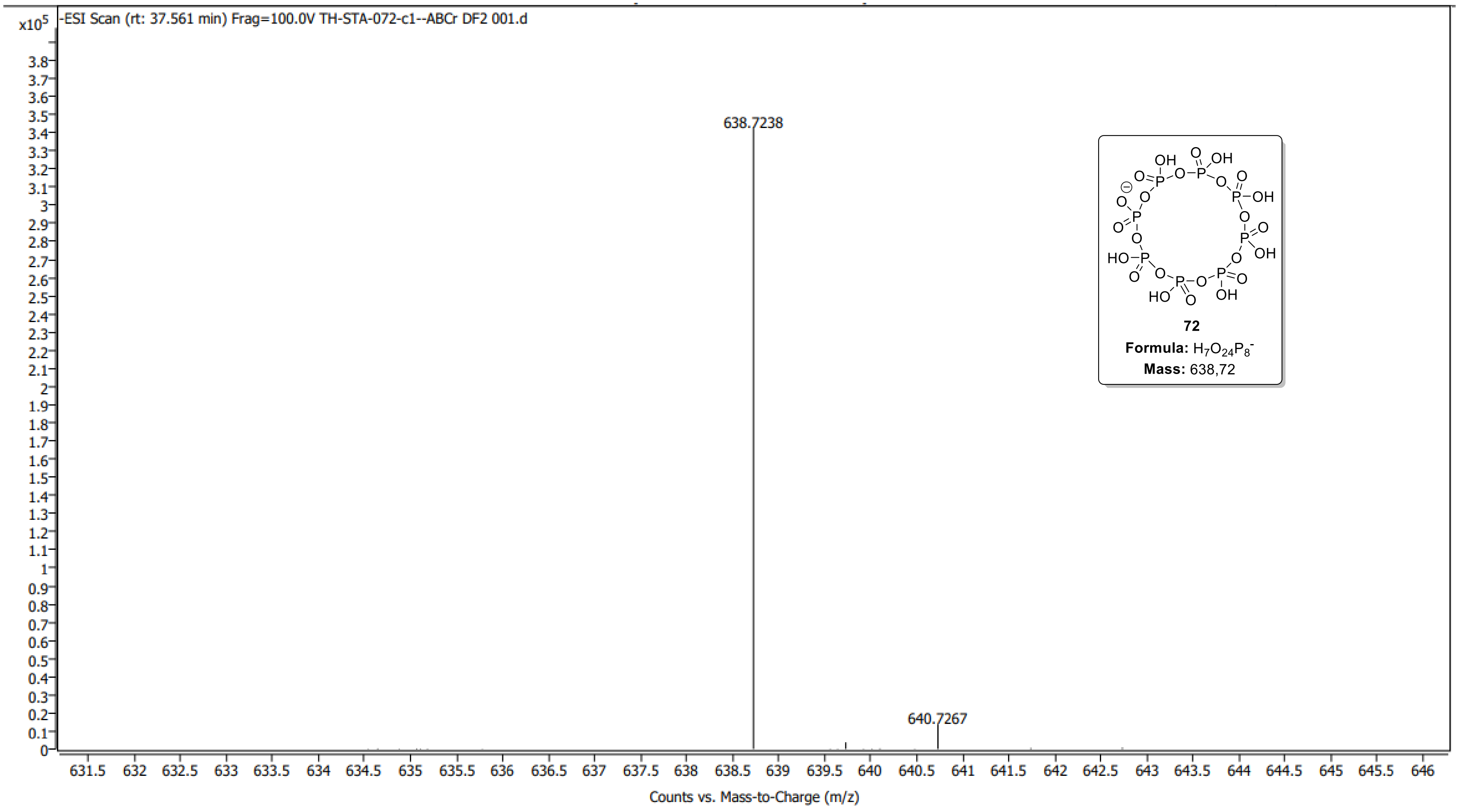

HRMS (ESI) Analysis of compound 73: Phenylcyclotriphosphate

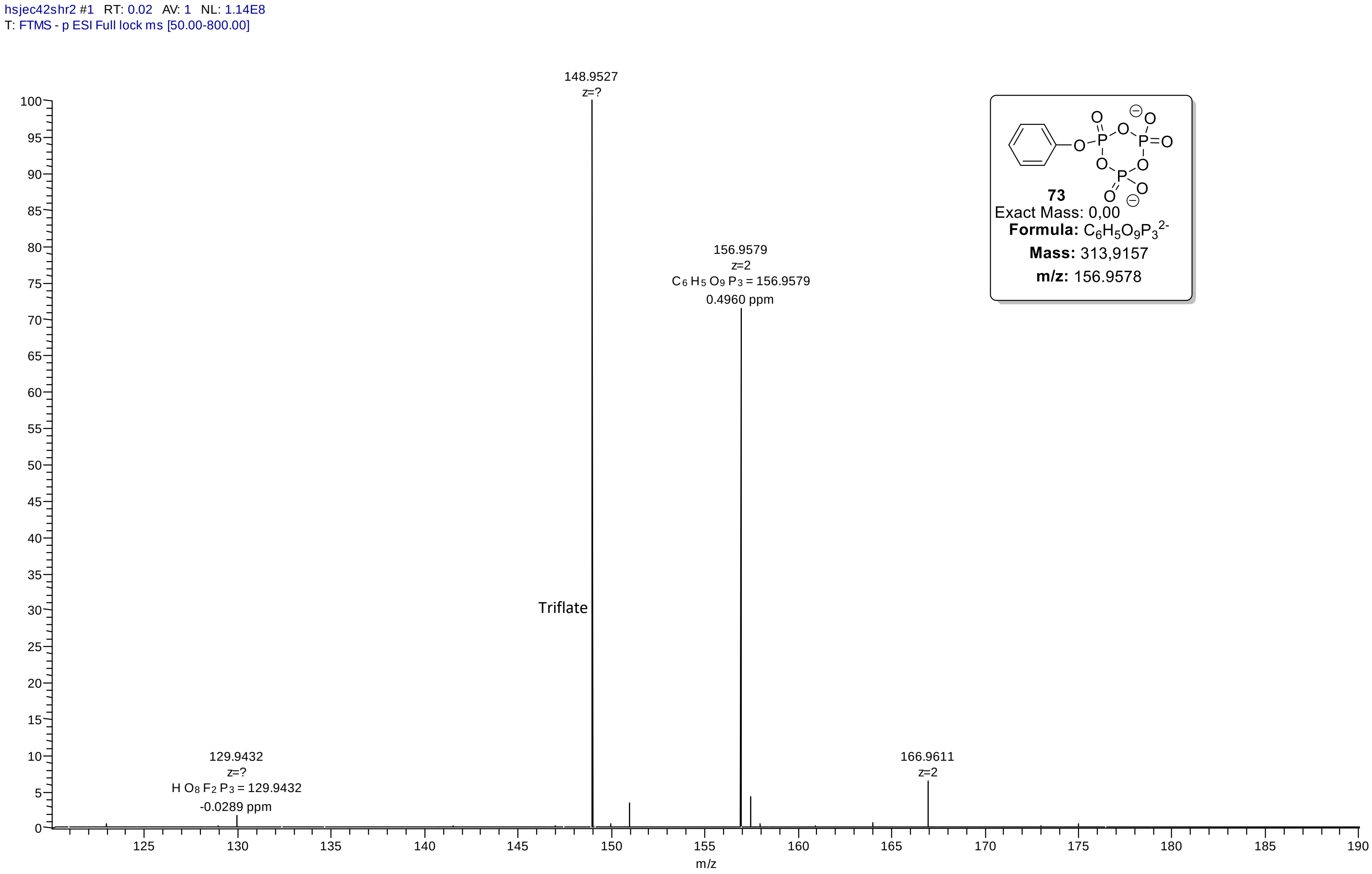

73

Exact Mass: 0,00

Formula: $C_6H_5O_9P_3^{2-}$

Mass: 313,9157

m/z: 156.9578

HRMS (ESI) Analysis of compound 74: Phenylcyclotetraphosphate

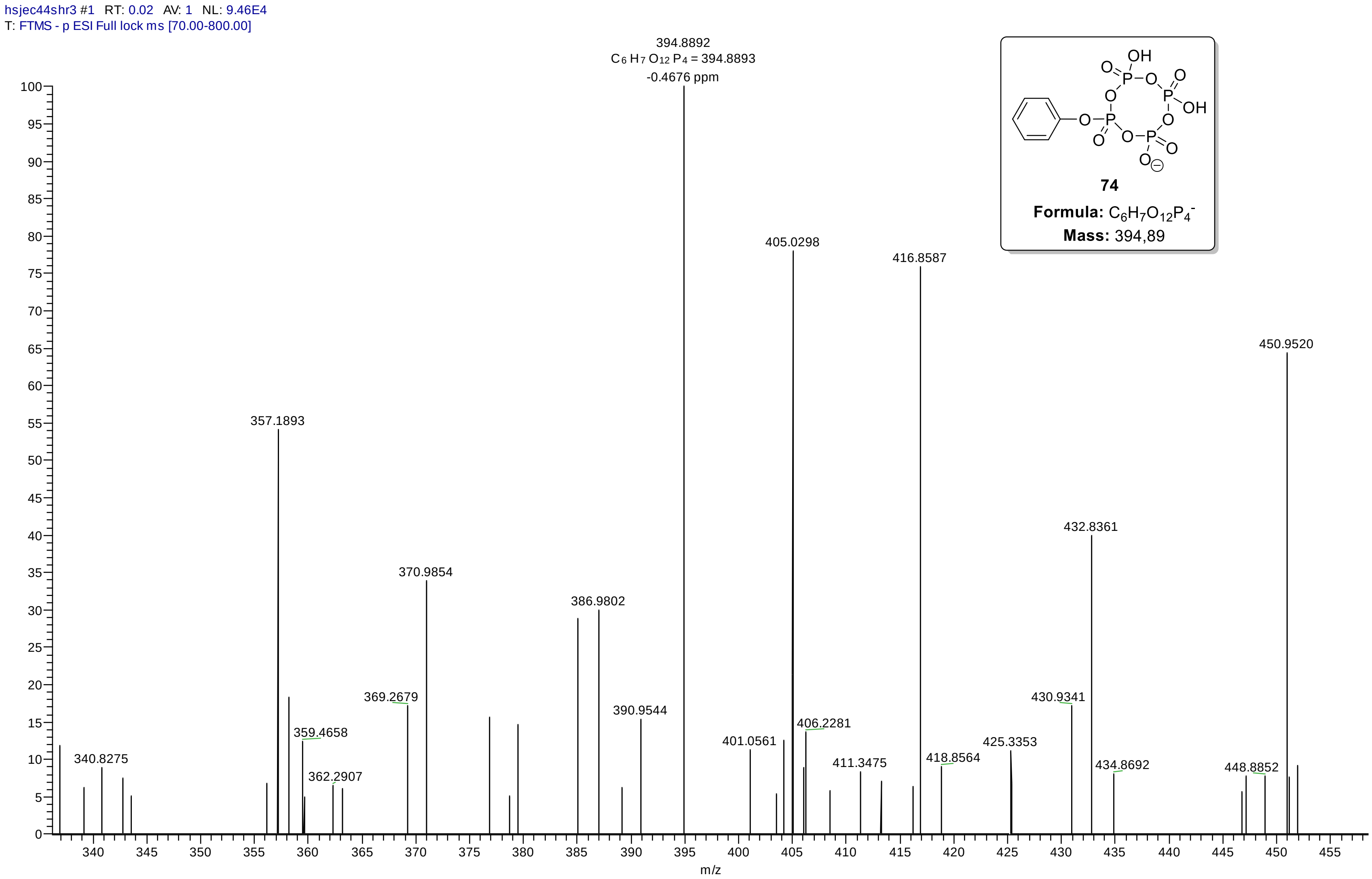

74

Formula: $C_6H_7O_{12}P_4^-$

Mass: 394,89

HRMS (ESI) Analysis of compound 78: PhenylP₃-propargylamidate

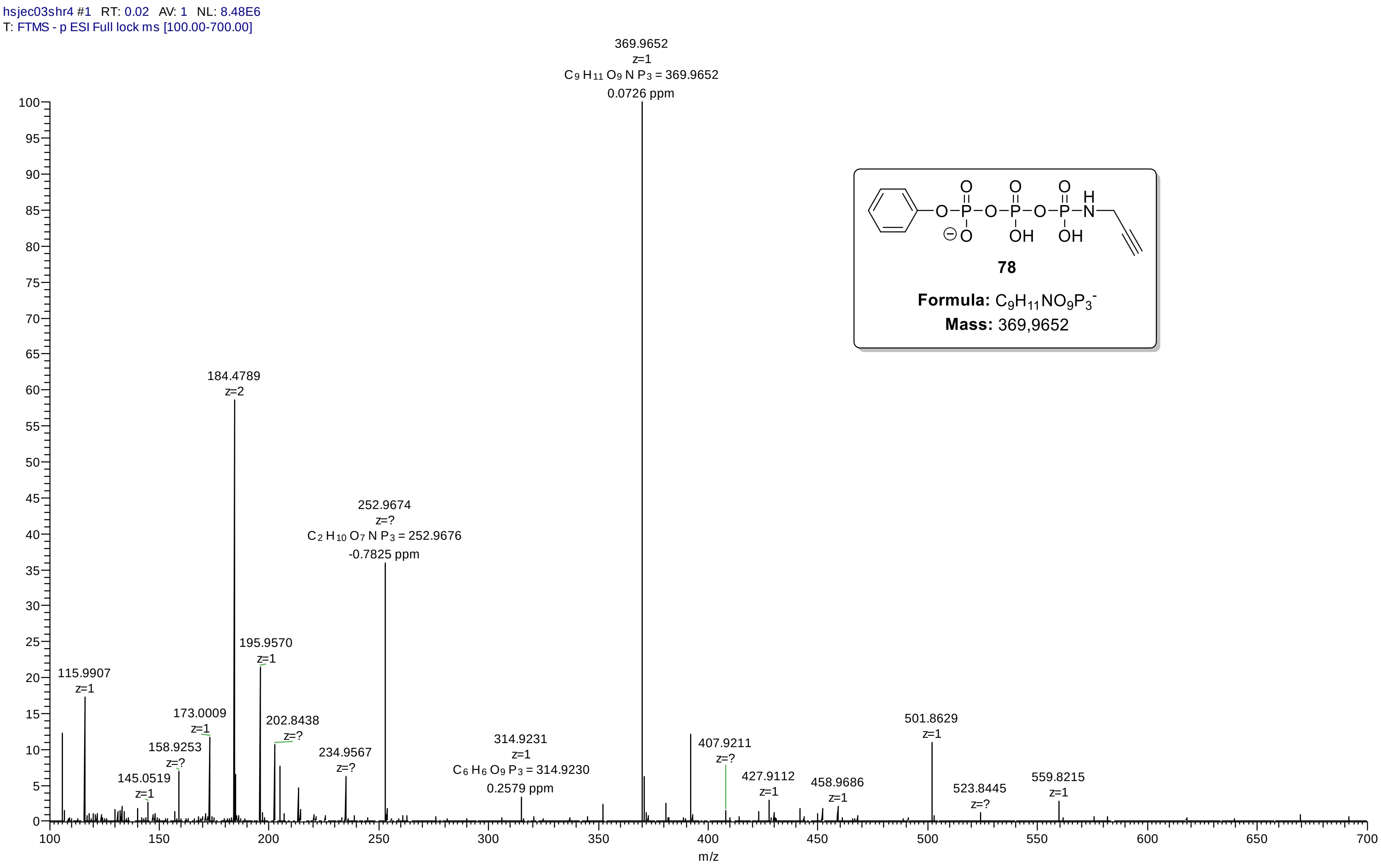

78

Formula: $C_9H_{11}NO_9P_3^-$

Mass: 369,9652

HRMS (ESI) Analysis of compound 79: PhenylP$_3$-amidate

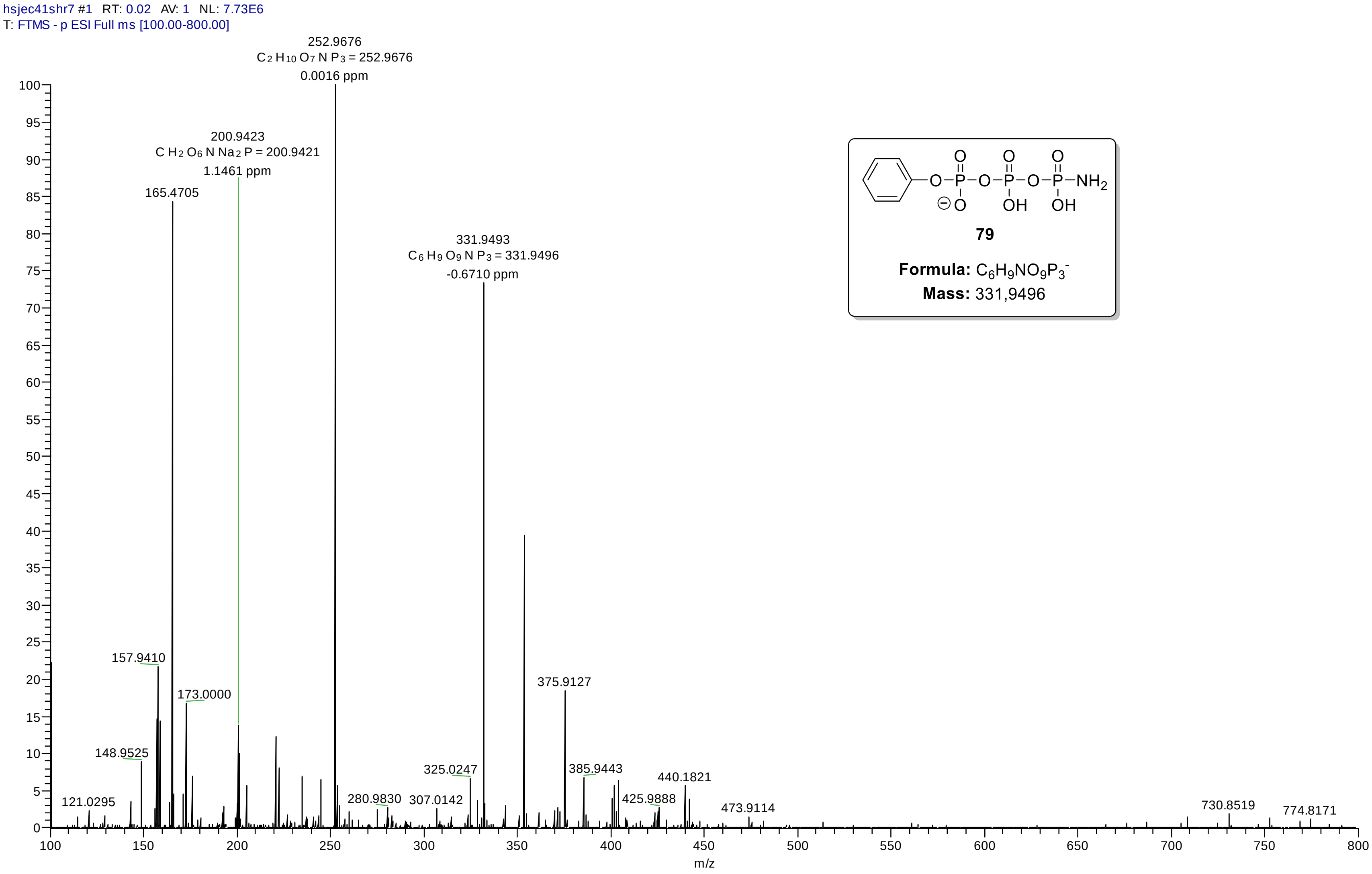

HRMS (ESI) Analysis of compound 80: PhenylP$_3$-anthracen-9-ylmethanamidate

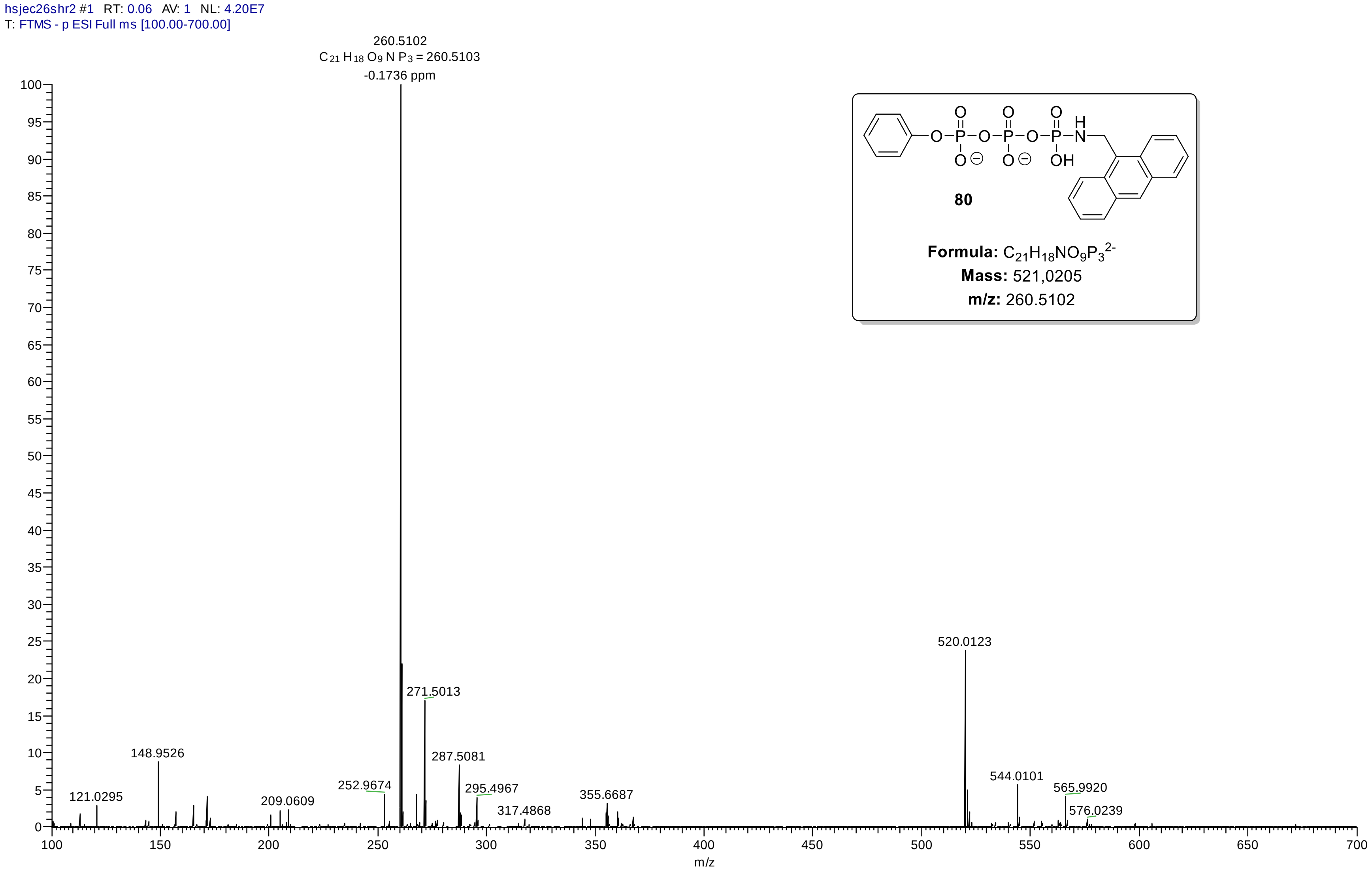

HRMS (ESI) Analysis of compound 81: PhenylP₃-5'-deoxyadenosyl-5'-amidate

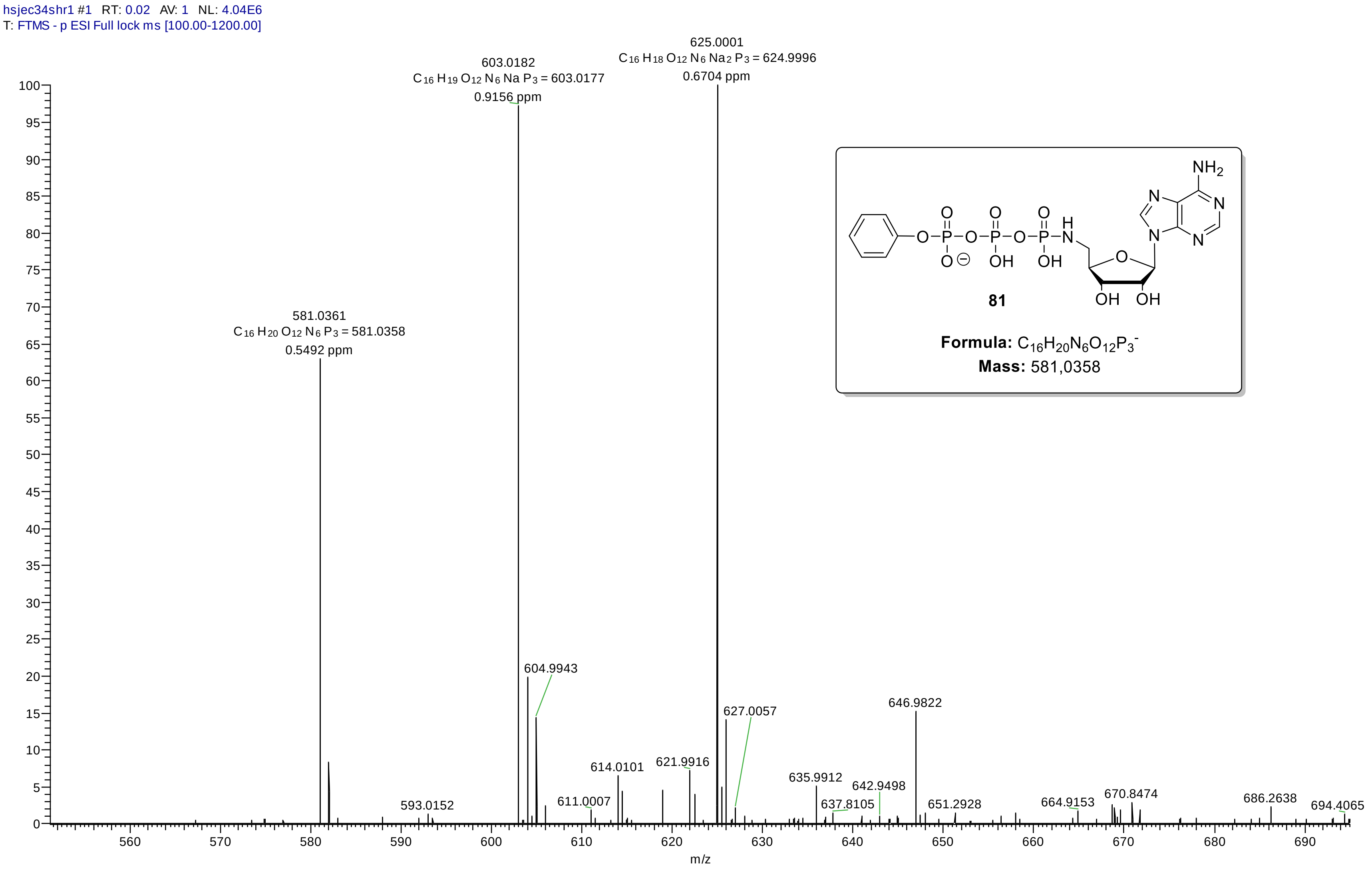

HRMS (ESI) Analysis of compound 82: PhenylP₃-5'-deoxyguanosyl-5'-amidate

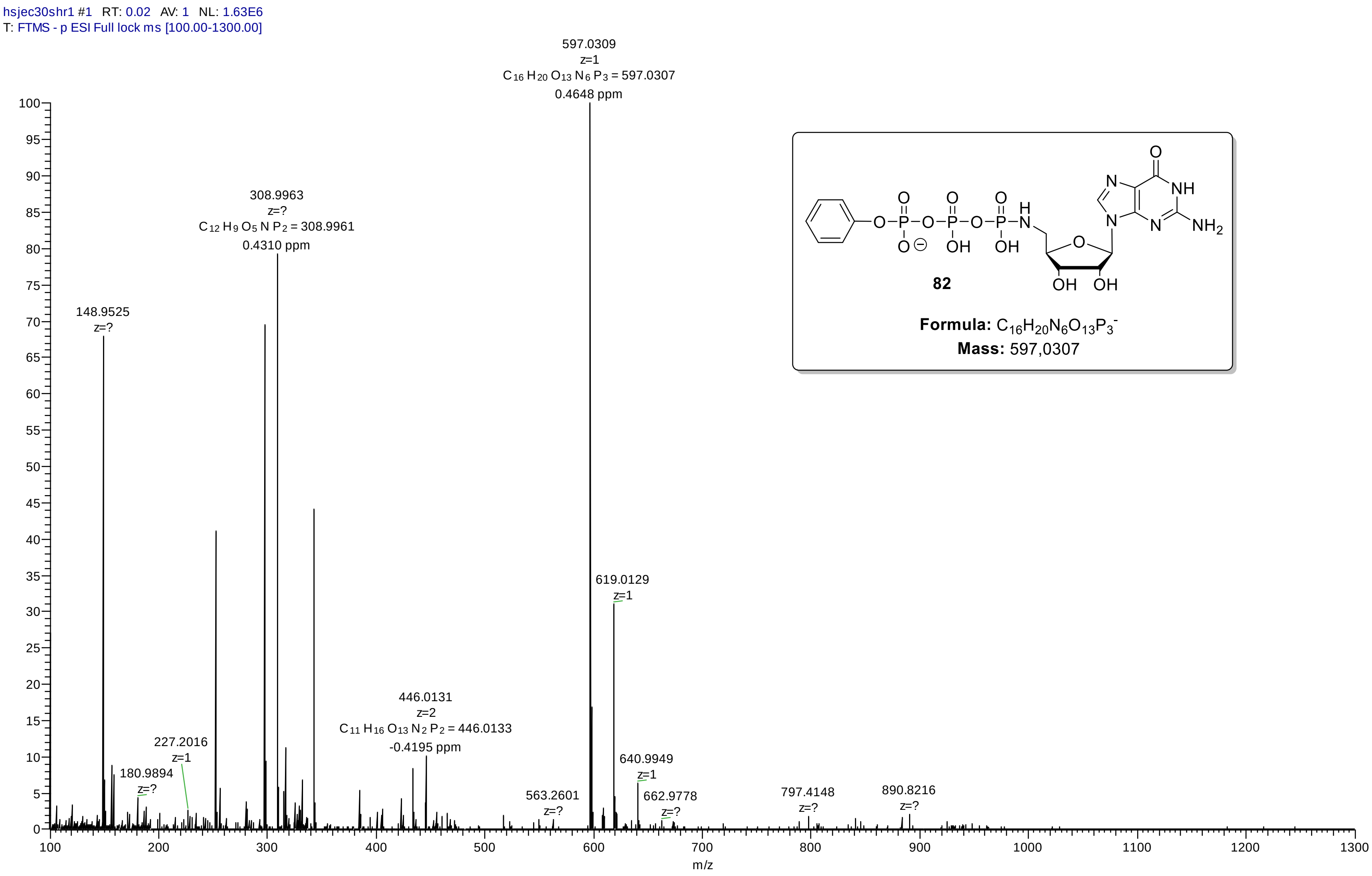

82

Formula: $C_{16}H_{20}N_6O_{13}P_3^-$
Mass: 597,0307

HRMS (ESI) Analysis of compound 83: Napht-2-ylP$_3$-5’-deoxyguanosyl-5’-amidate

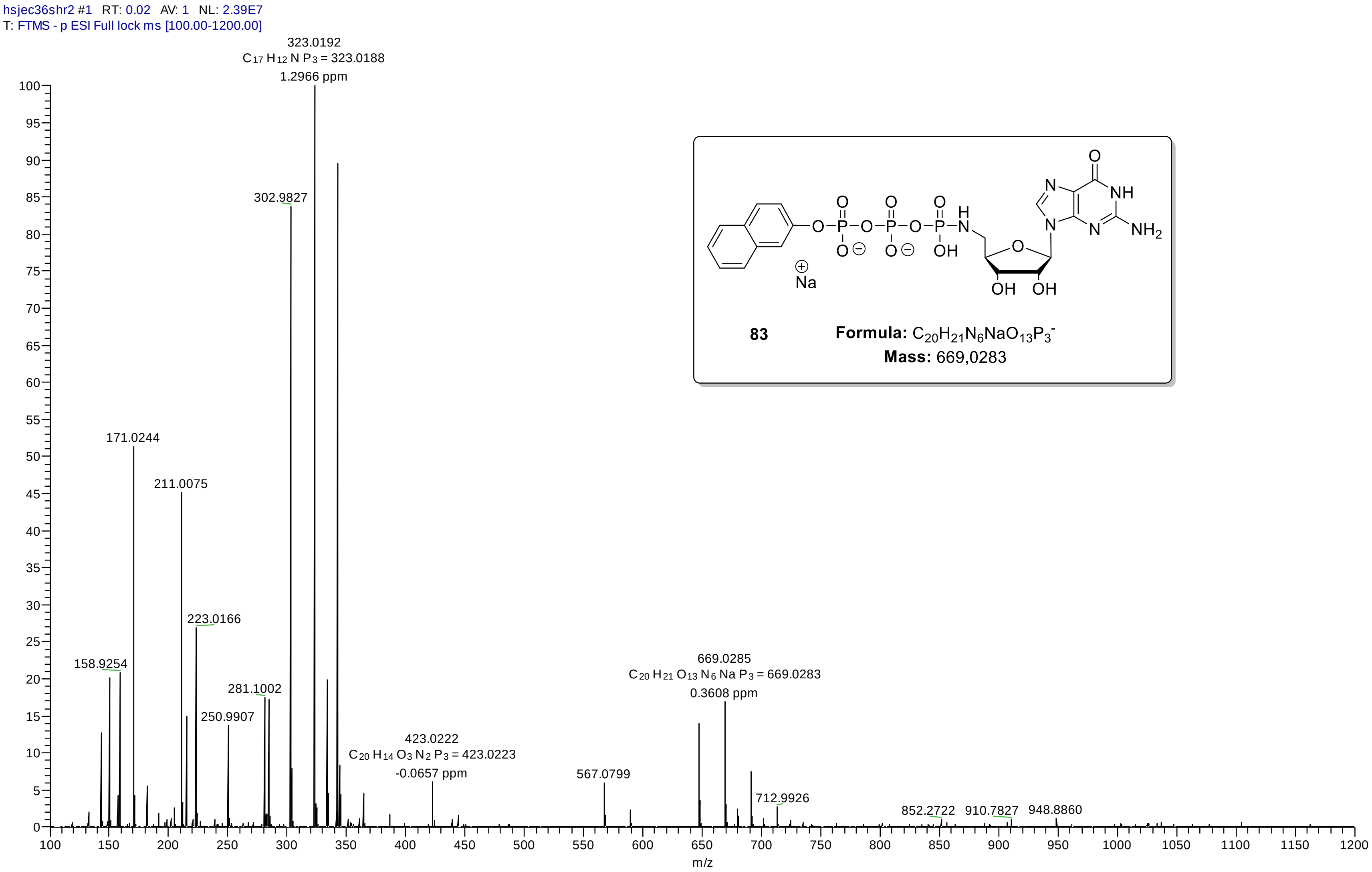

HRMS (ESI) Analysis of compound 84: Pyren-2-ylP$_3$-5'-deoxyadenosyl-5'-amidate

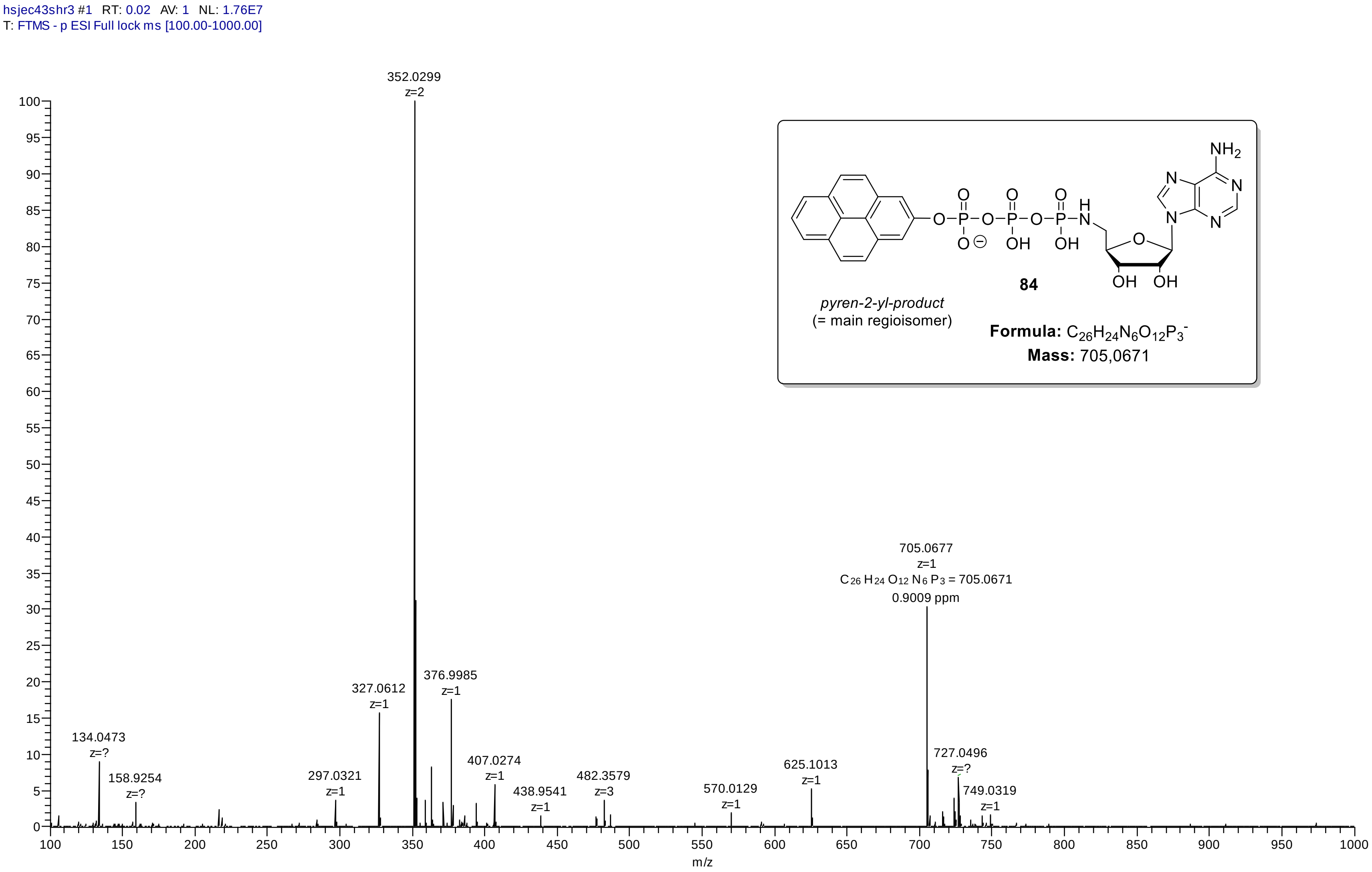

HRMS (ESI) Analysis of compound 85: Benzo[1,3]dioxol-5-ylP3-((7-(diethylamino)-2-oxo-2H-chromen-4-yl) methylamidate

hsjec31shr2 #1 RT: 0.02 AV: 1 NL: 1.21E8
T: FTMS - p ESI Full lock ms [100.00-1300.00]

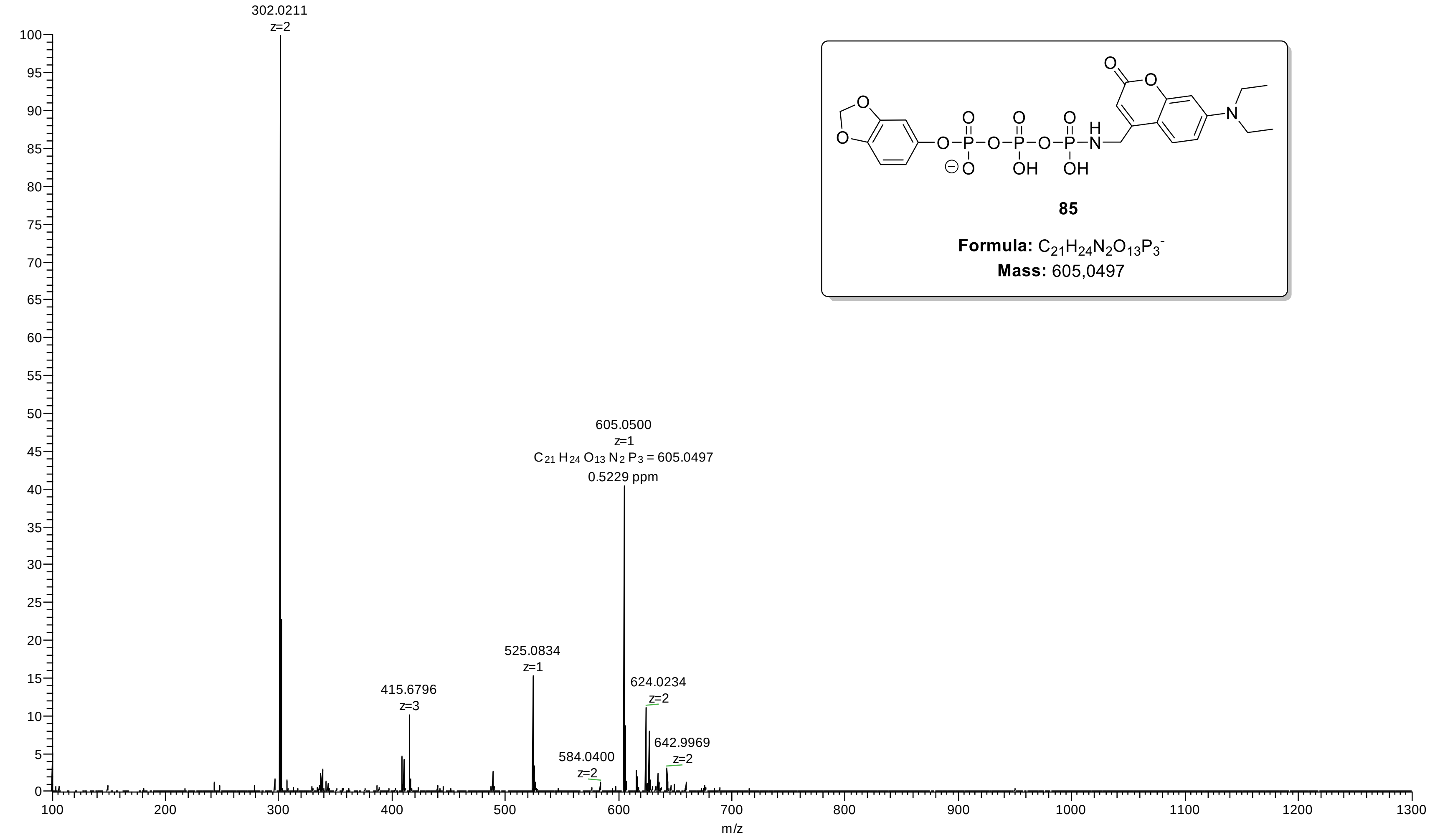

85

Formula: $C_{21}H_{24}N_2O_{13}P_3^-$
Mass: 605,0497

HRMS (ESI) Analysis of compound 86: PhenylP$_4$-propargylamidate

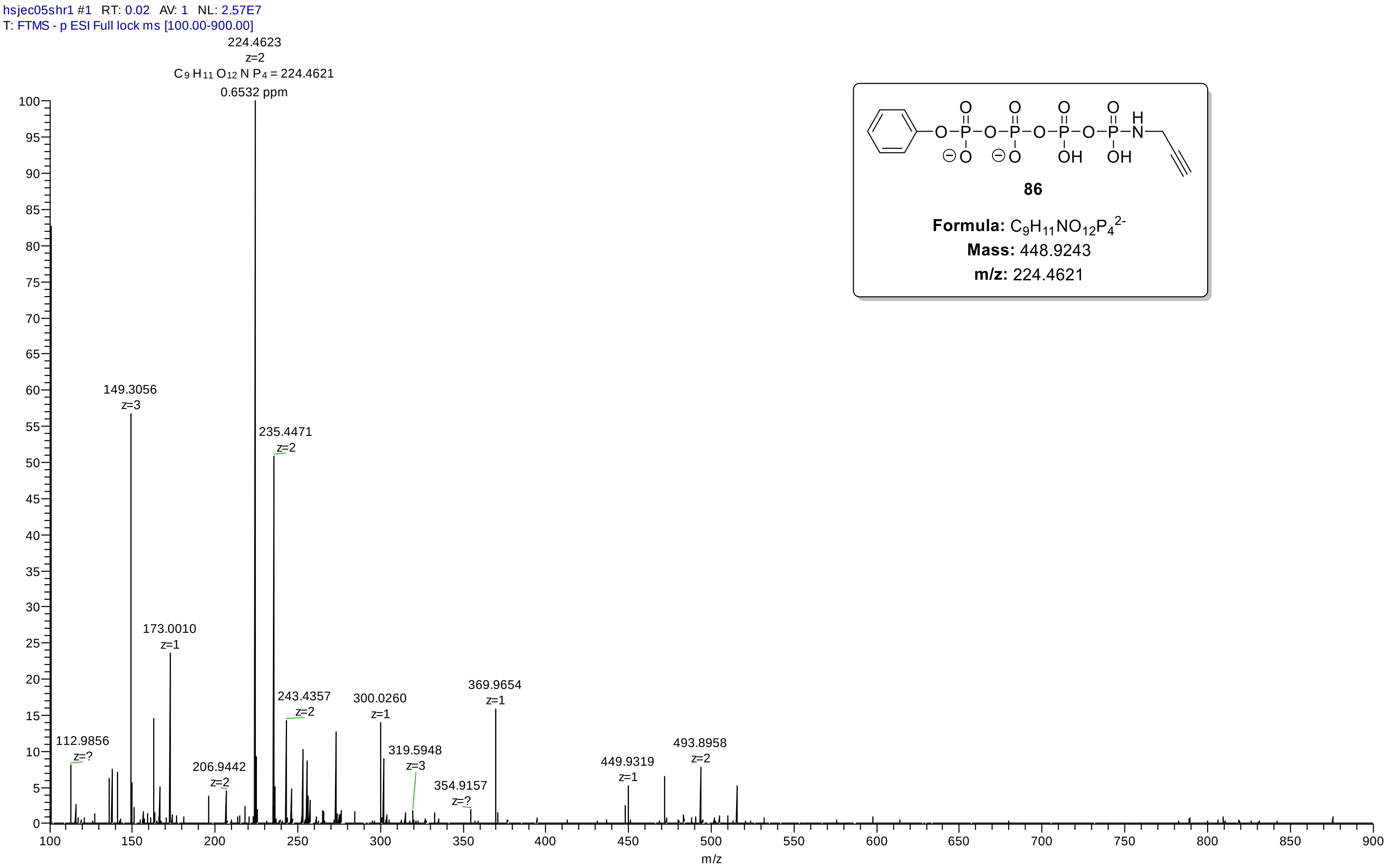

86

Formula: $C_9H_{11}NO_{12}P_4^{2-}$

Mass: 448.9243

m/z: 224.4621

HRMS (ESI) Analysis of compound 87: Pyren-2-ylP4-5'-deoxyguanosyl-5'-amidate

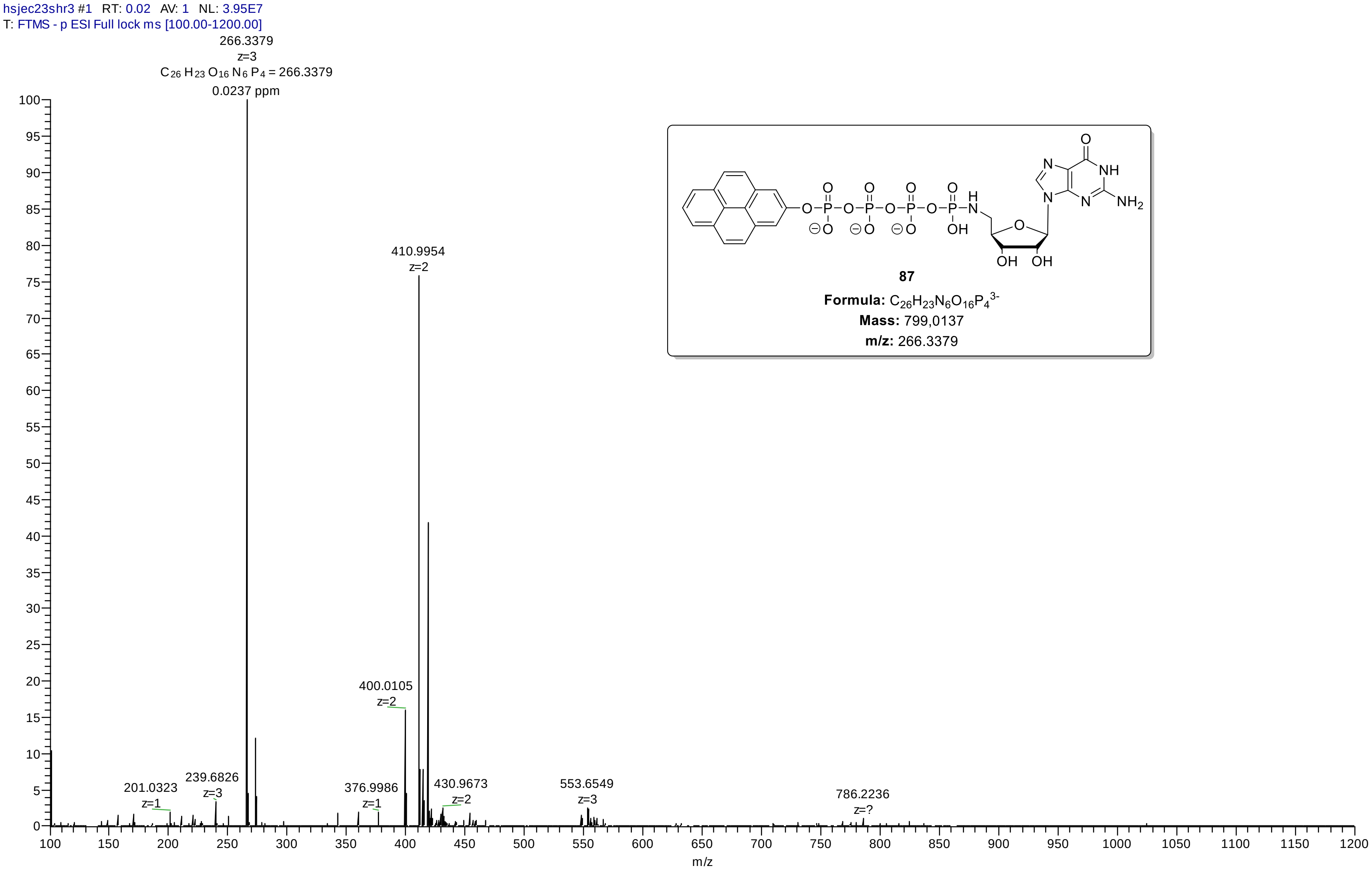

HRMS (ESI) Analysis of compound 88: PhenylP4-5'-deoxyguanosyl-5'-amidate

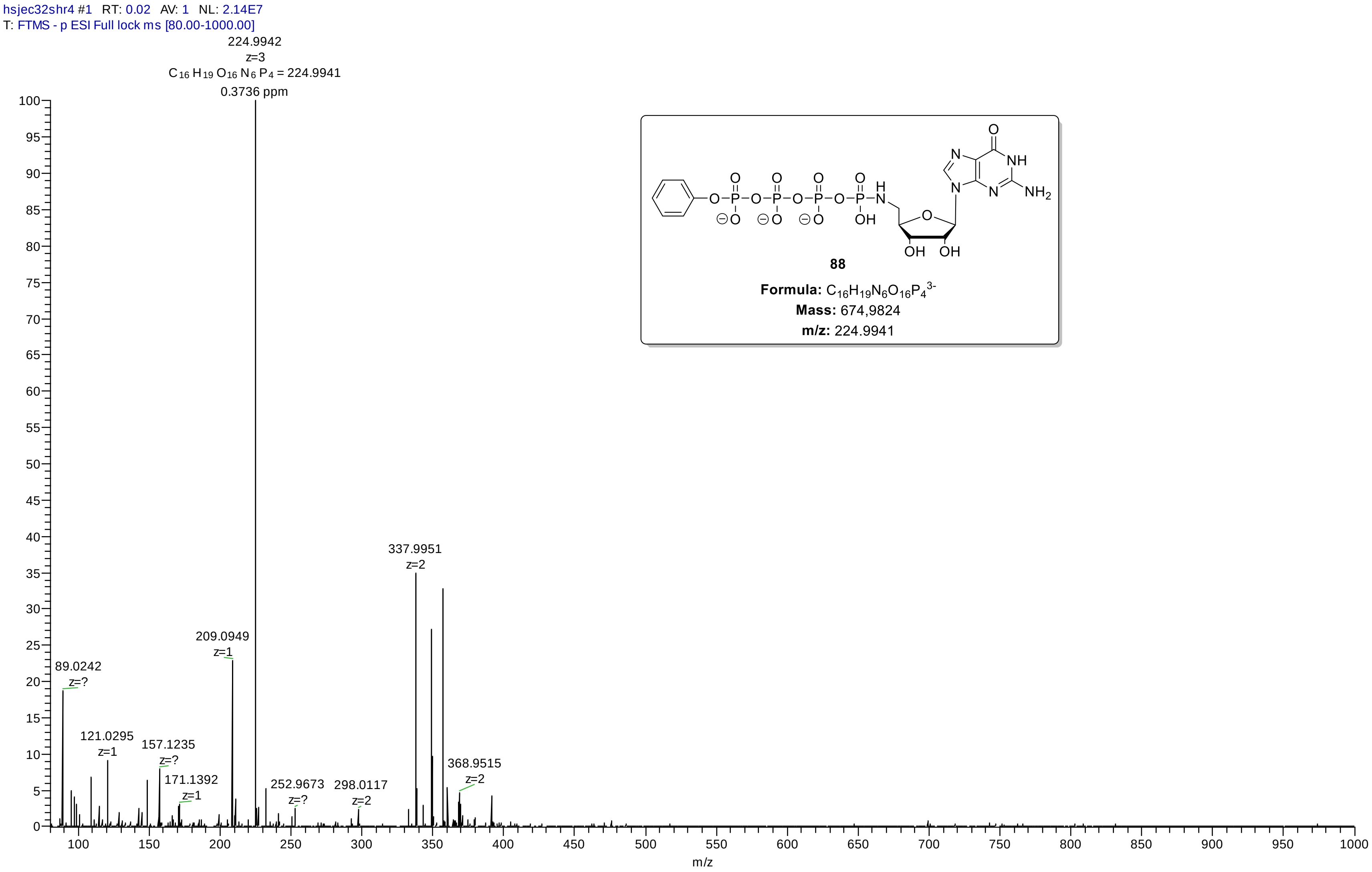

HRMS (ESI) Analysis of compound 89: 3,4-DimethylphenylP₄-(7-(diethylamino)-2-oxo-2H-chromen-4-yl) methylamidate

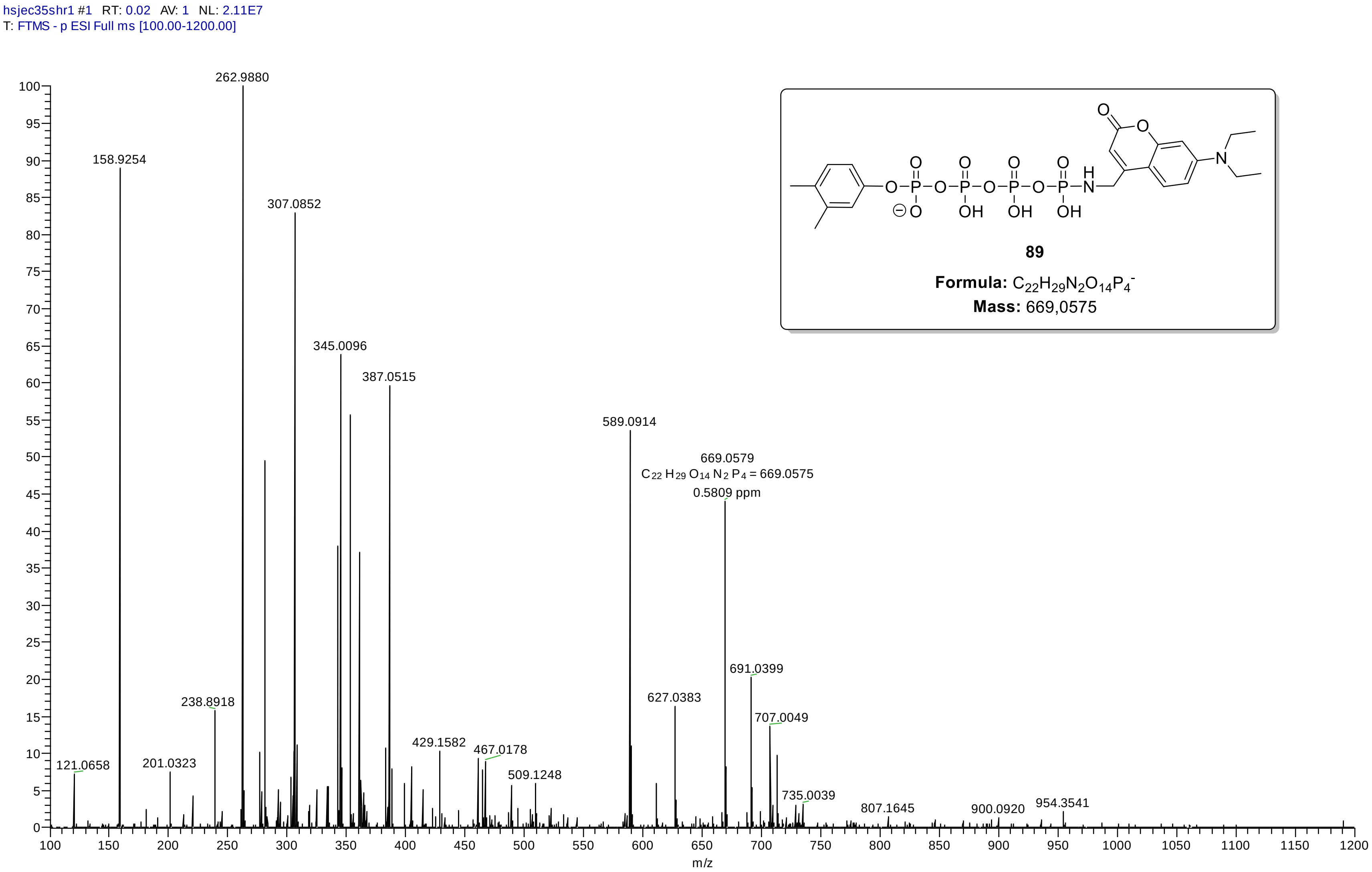

89

Formula: $C_{22}H_{29}N_2O_{14}P_4^-$

Mass: 669,0575

HRMS (ESI) Analysis of compound 90: PhenylIP₅-propargylamidate

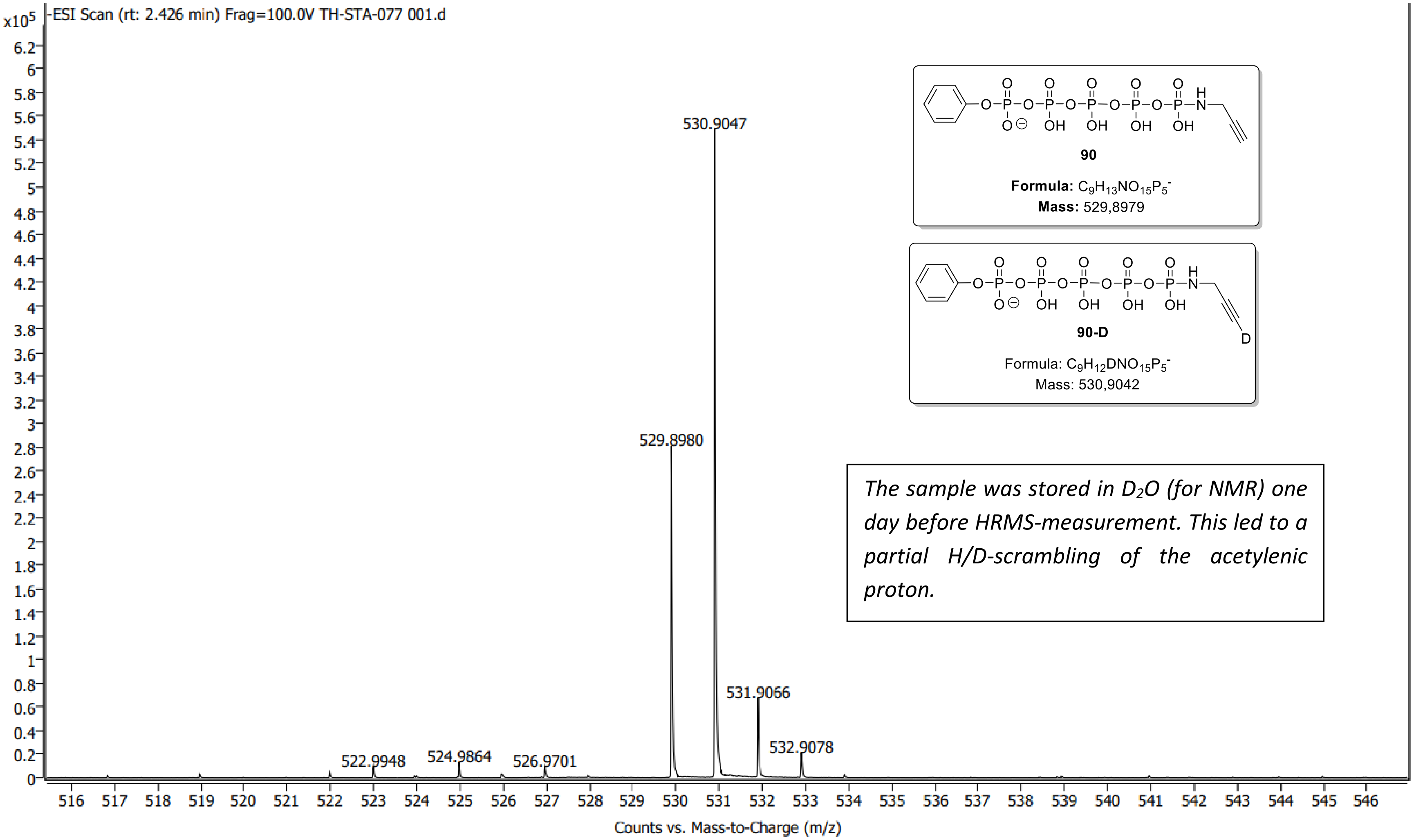

The sample was stored in D_2O (for NMR) one day before HRMS-measurement. This led to a partial H/D-scrambling of the acetylenic proton.

HRMS (ESI) Analysis of compound 91: PhenylP₇-propargylamidate

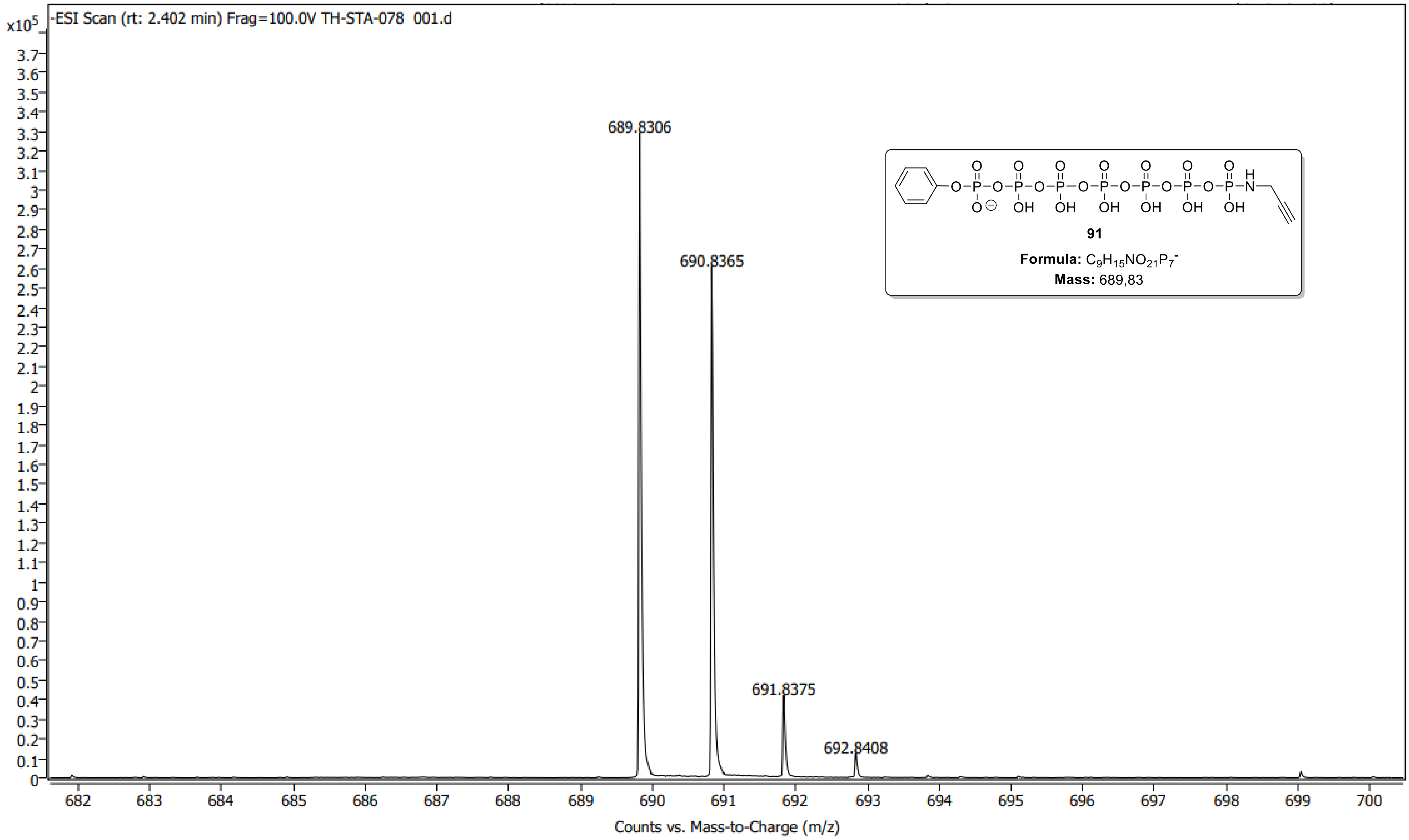

HRMS (ESI) Analysis of compound 92: PhenylP8-propargylamidate

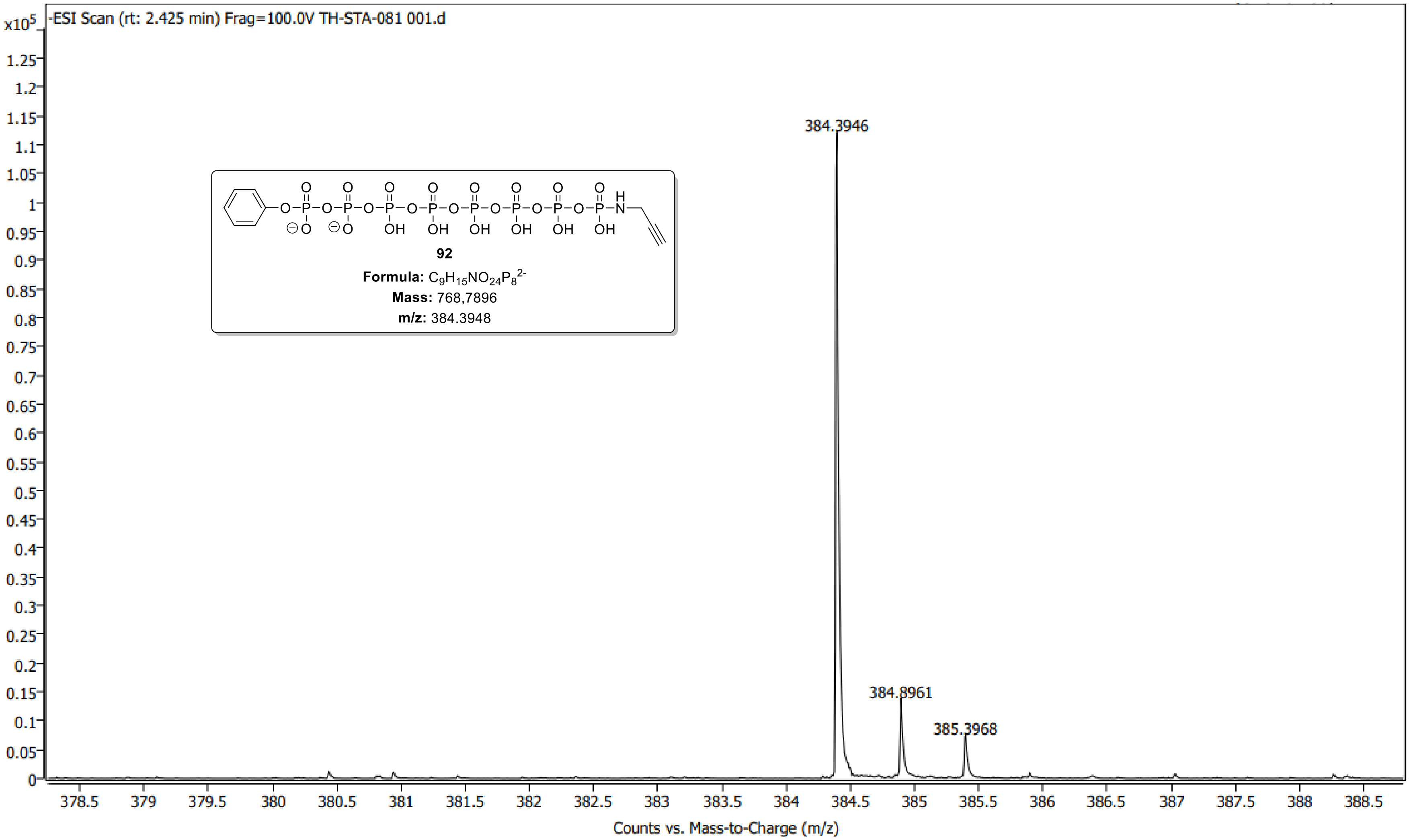

HRMS (ESI) Analysis of compound SI-5: D4T - monophosphate

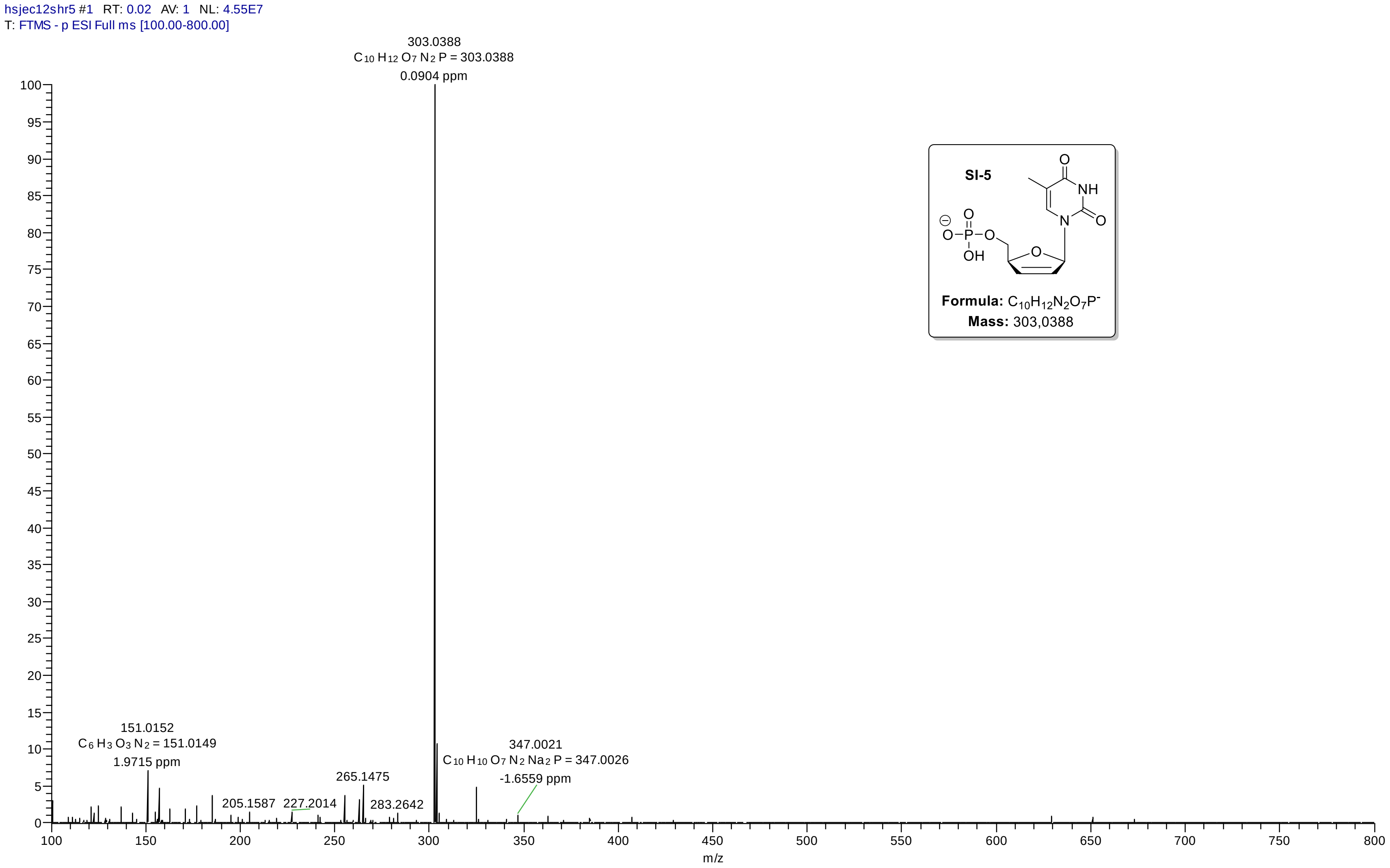

HRMS (ESI) Analysis of compound SI-13: 6-Hydroxyhexylphosphate

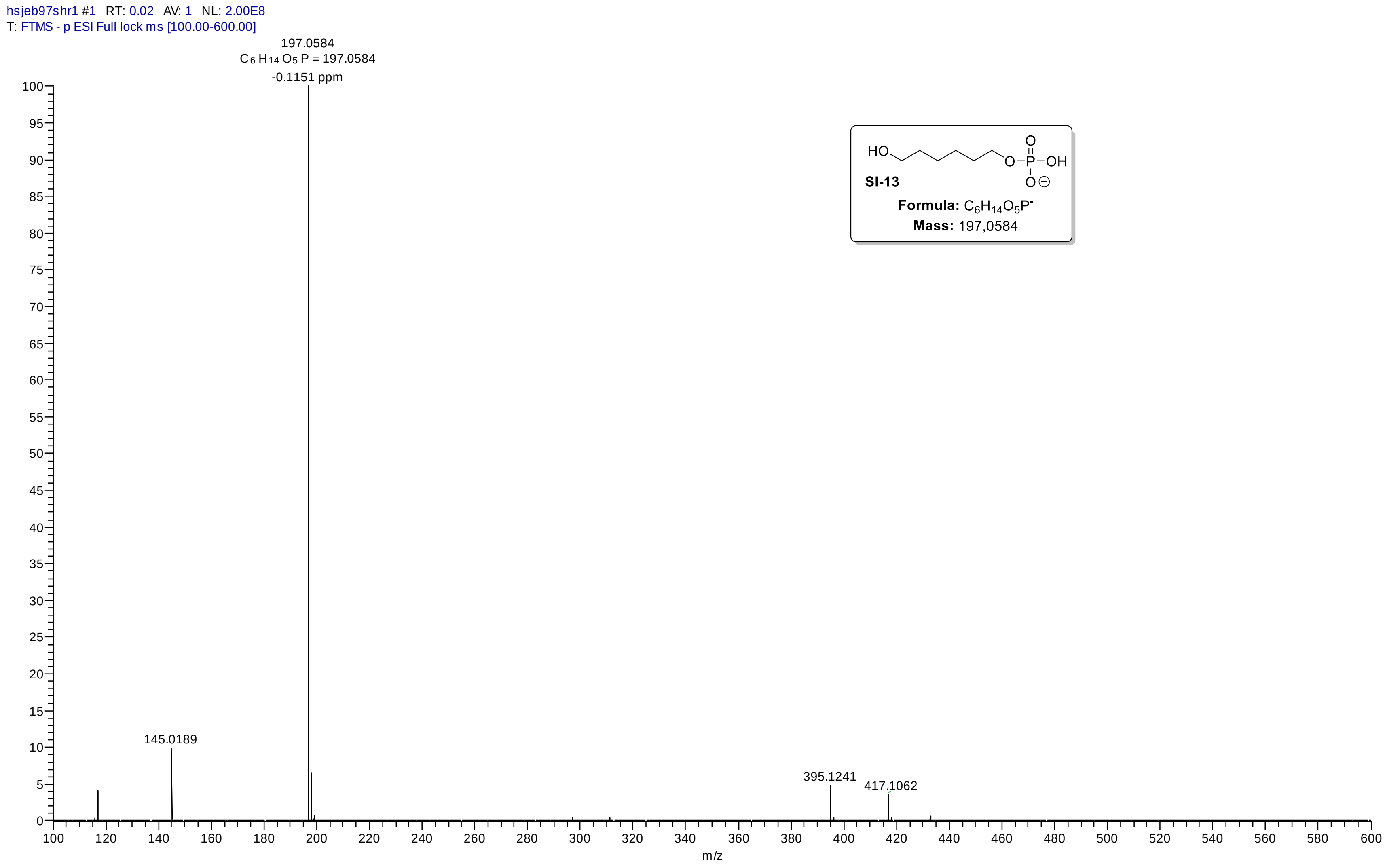

HRMS (ESI) Analysis of compound SI-14: Geranylmonophosphate

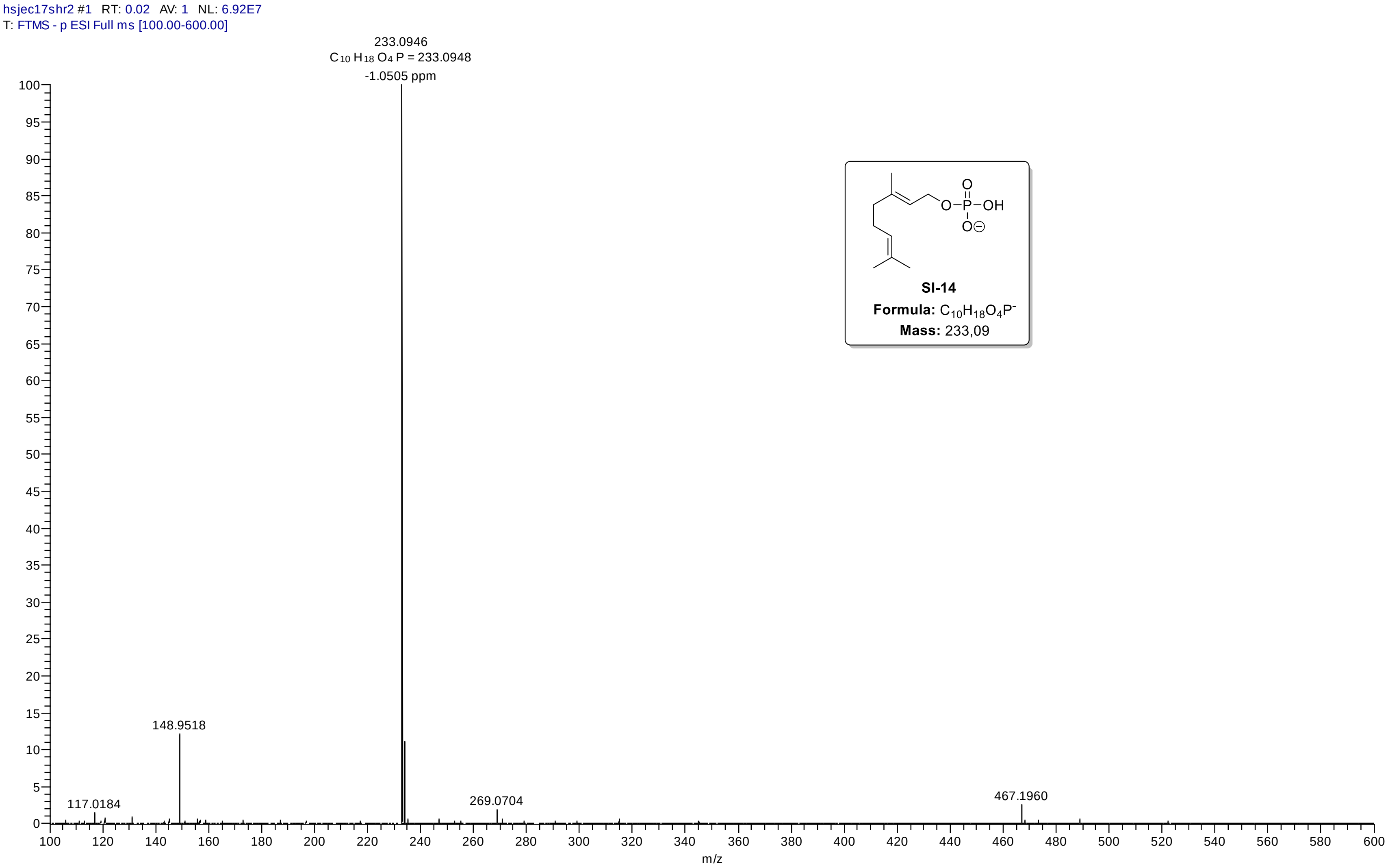

SI-14

Formula: $C_{10}H_{18}O_4P^-$

Mass: 233,09

HRMS (ESI) Analysis of compound SI-15: Isoprenylmonophosphate

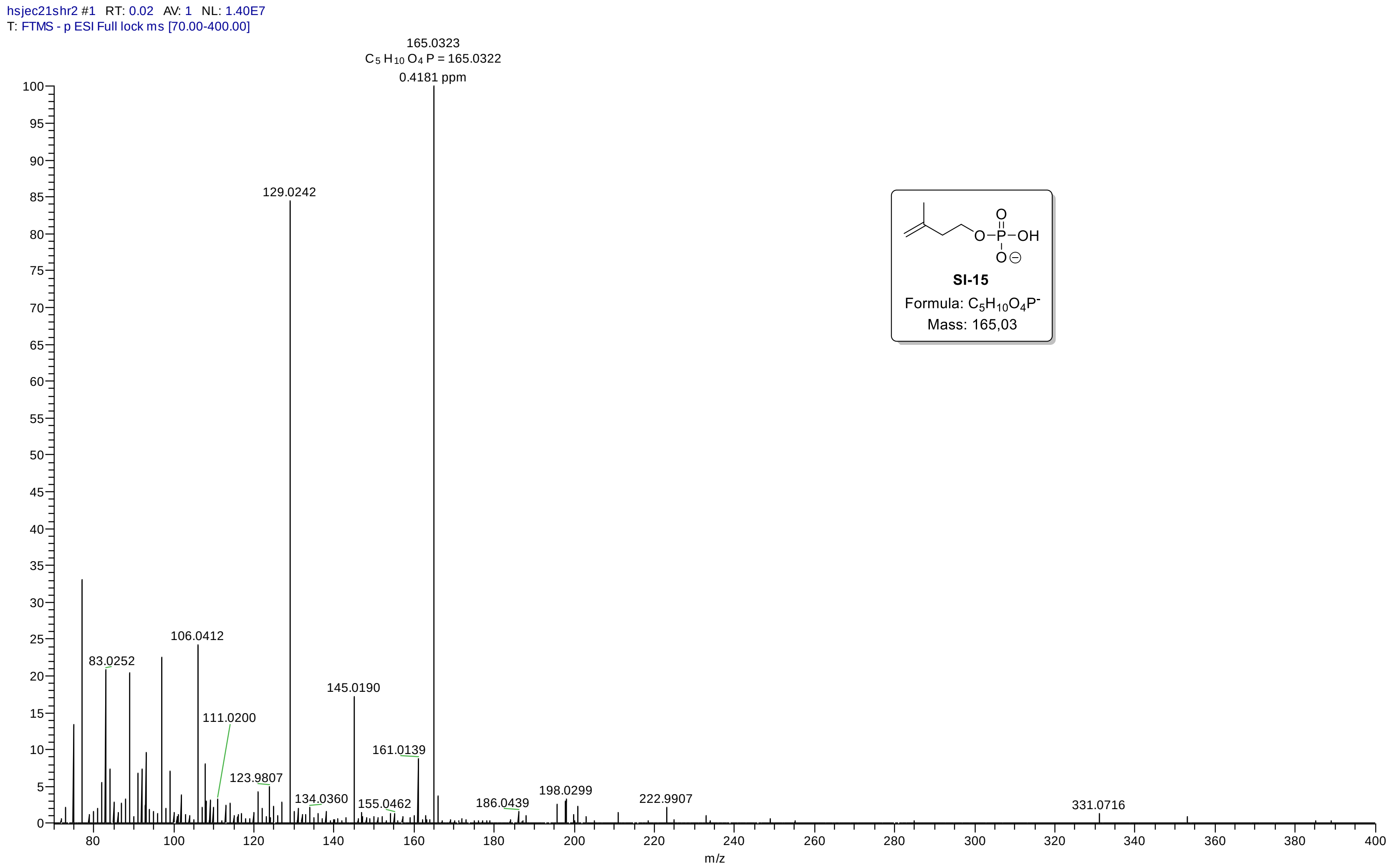

SI-15

Formula: $C_5H_{10}O_4P^-$

Mass: 165,03

HRMS (ESI) Analysis of compound SI-16: phenyldiphosphate

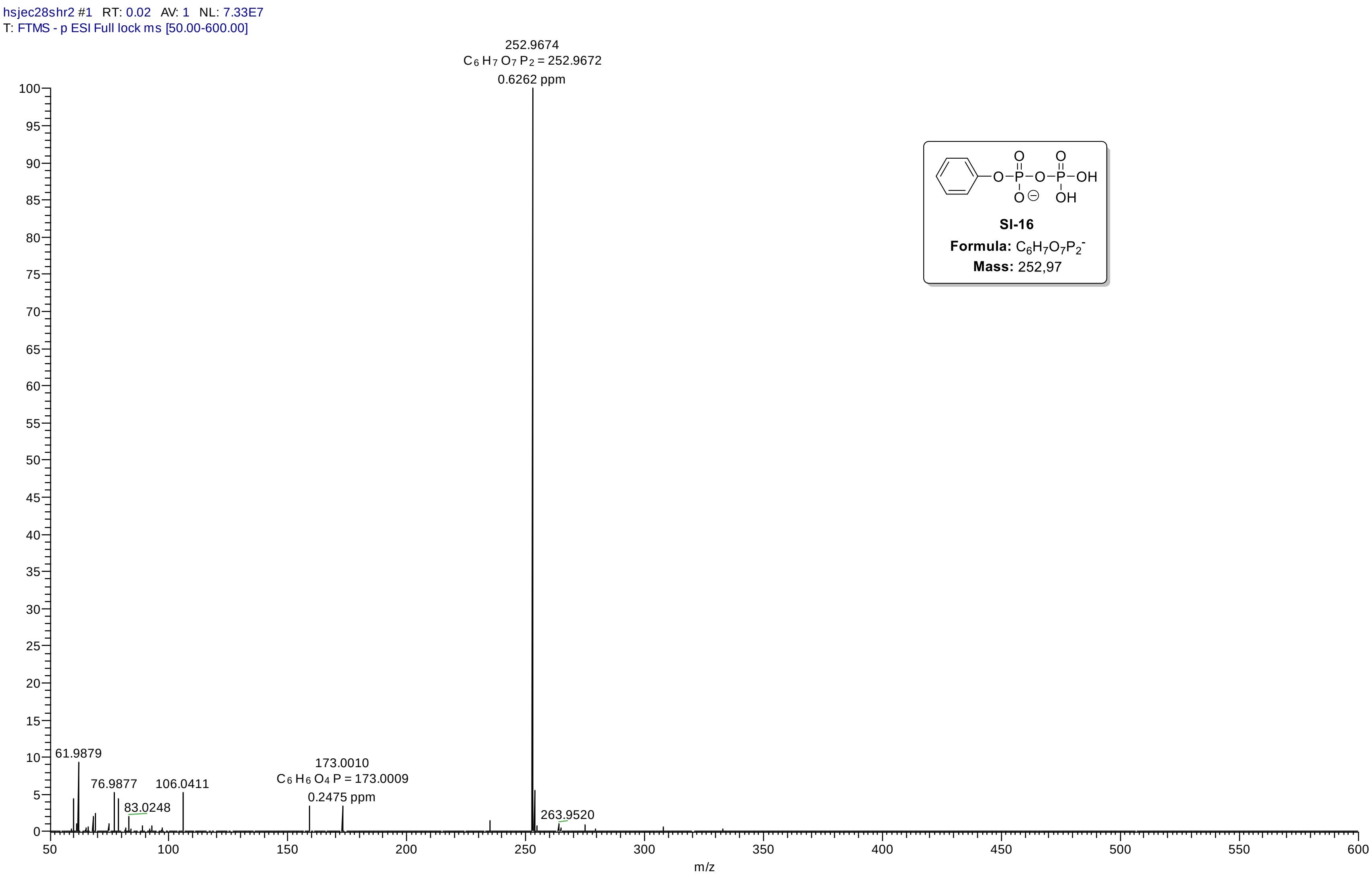

HRMS (ESI) Analysis of compound SI-17: D4T - diphosphate

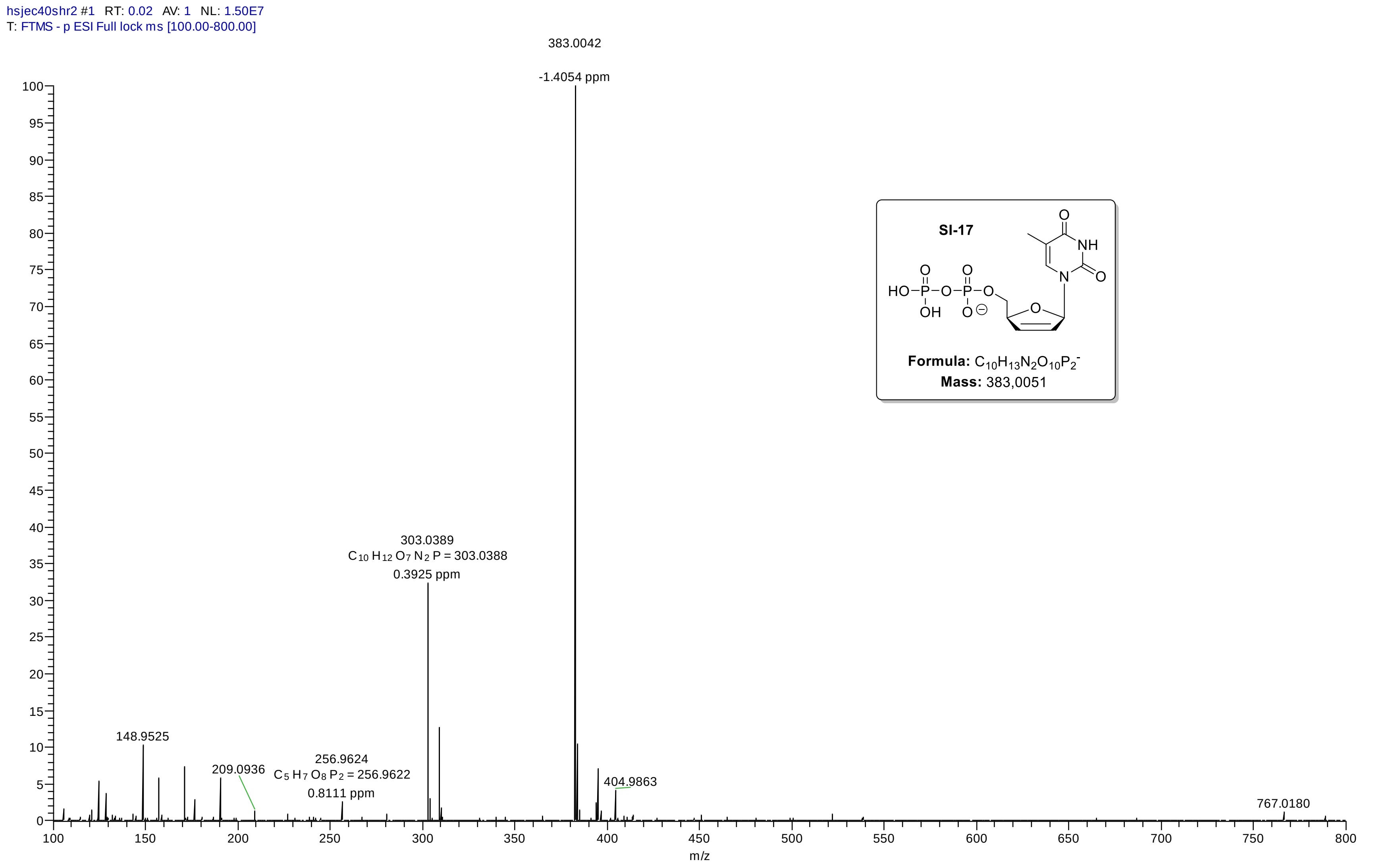

HRMS (APCI) Analysis of compound SI-19: 2-(trimethylsilyl)pyren-1-yl trifluoromethanesulfonate

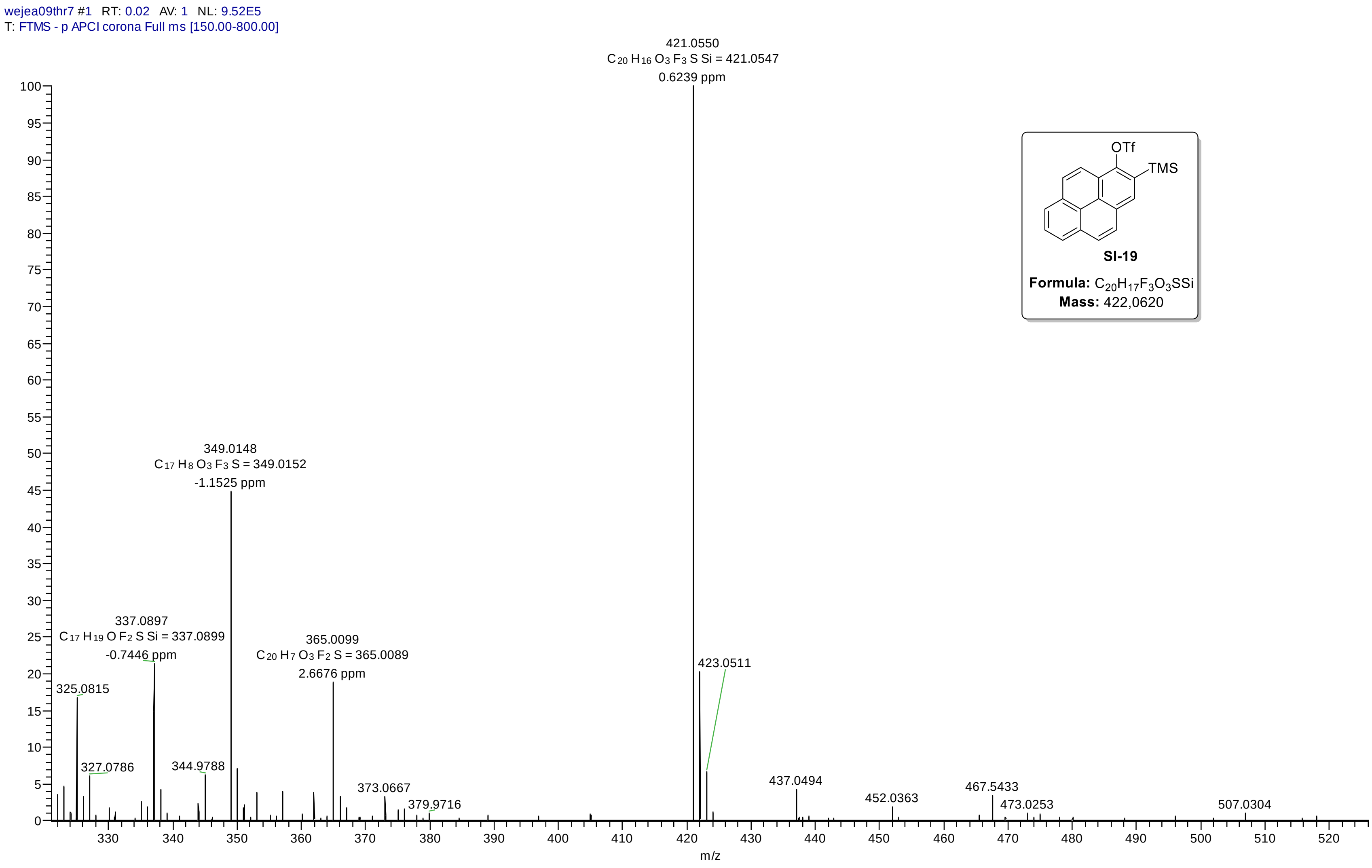

SI-19

Formula: $C_{20}H_{17}F_3O_3SSi$
Mass: 422,0620

HRMS (ESI) Analysis of compound SI-21: 4-(trimethylsilyl)ethynyl)phenyl isopropylcarbamate

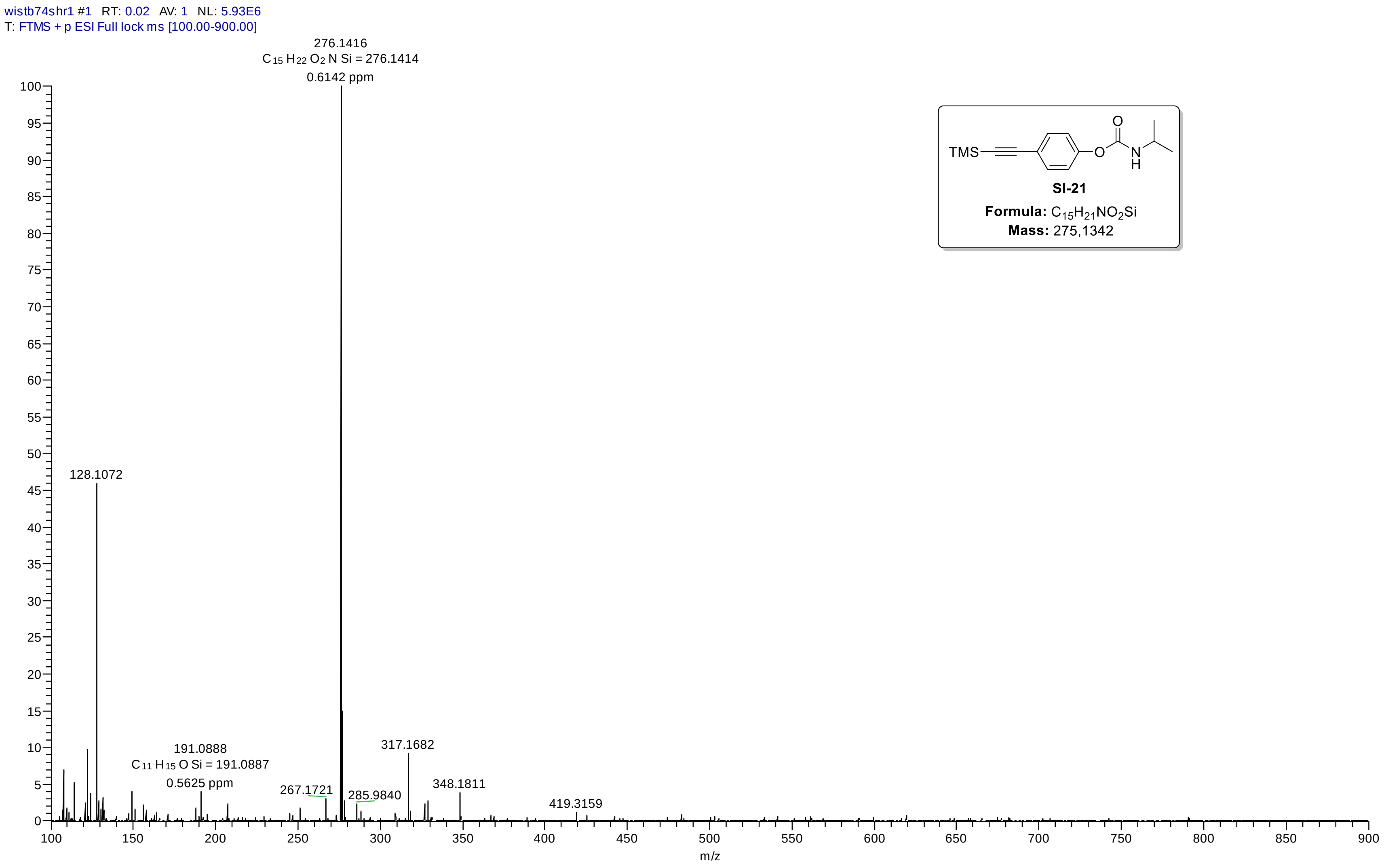

SI-21

Formula: $C_{15}H_{21}NO_2Si$

Mass: 275,1342

HRMS (ESI) Analysis of compound SI-22: 2-(trimethylsilyl)-4-((trimethylsilyl)ethynyl)phenyl isopropylcarbamate

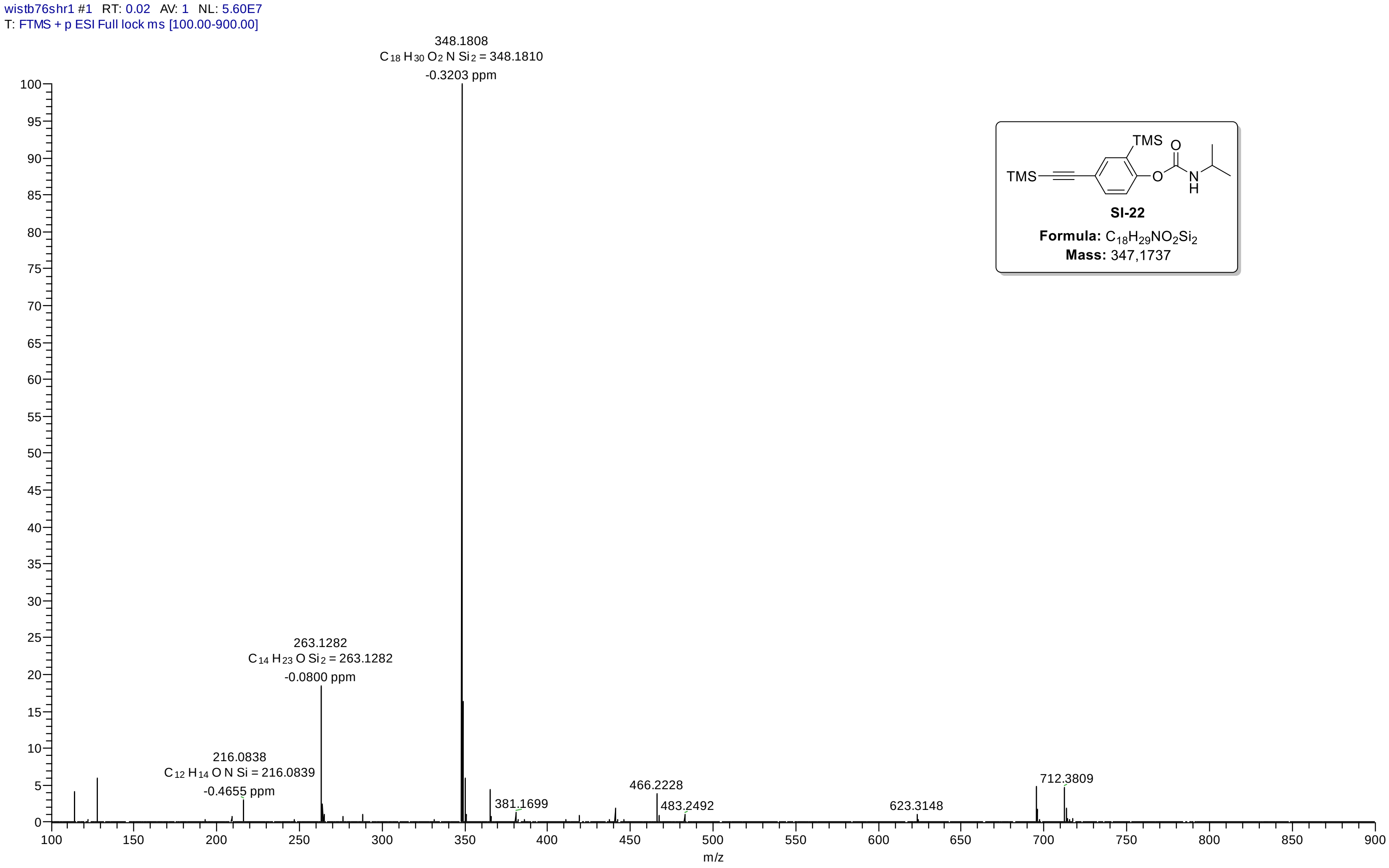

SI-22

Formula: $C_{18}H_{29}NO_2Si_2$

Mass: 347,1737

HRMS (APCI) Analysis of compound SI-23: 2-(trimethylsilyl)-4-((trimethylsilyl)ethynyl)phenyl trifluoromethanesulfonate

Analysis Report

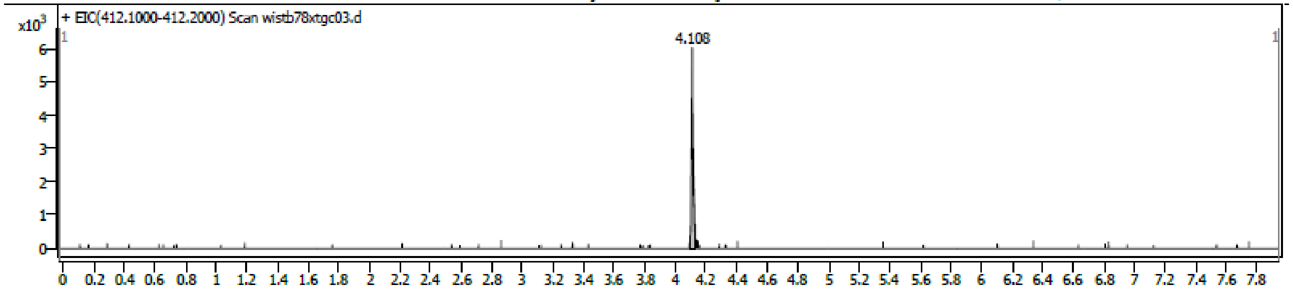

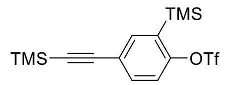

SI-23

Formula: $C_{15}H_{21}F_3O_3SSi_2$

Mass: 394,0702

Chromatogram Peaks

Peak	Start	RT	End	Height	Area	Area %	SNR
1	4.092	4.108	4.125	6027	4360	100.00	

Sample Spectra

+ Scan (rt: 3.984-4.319 min) Sub (2)

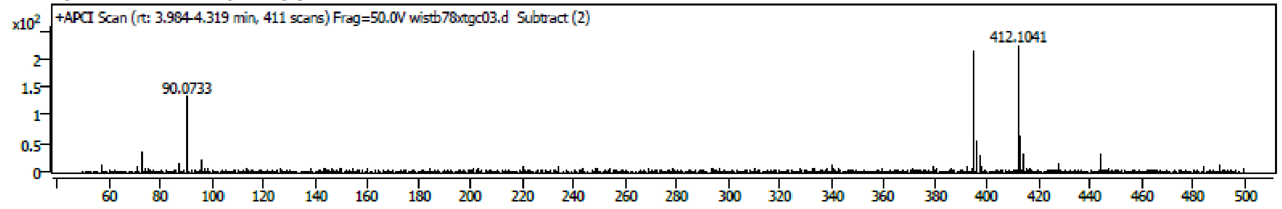

Spectrum Peaks

m/z	Z	Abund	Abund %	m/z (Calc)	Diff (ppm)	Ion Species	Formula	Ion Type
395.0775	1	213	95.44	395.0775	0.10	(M+H)+	C15 H21 F3 O3 S Si2	
412.1041	1	223	100.00	412.1040	0.07	(M+NH4)+	C15 H21 F3 O3 S Si2	
90.0733		133	59.71					

Spectrum Identification Table

Best ID	Source	Name	Formula	Species	m/z	Diff (ppm)	CAS	Score	Score (Lib)	Score (DB)	Score (MFG)	Lib/DB
No	MFG		C15 H21 F3 O3 S Si2	(M+H)+	395.0775	-0.19		96.82			96.82	
No	MFG		C15 H21 F3 O3 S Si2	(M+NH4)+	412.1041	0.07		96.58			96.58	

MassHunter Qual 10.0
(End of Report)

HRMS (ESI) Analysis of compound SI-25: 2-((7-(diethylamino)-2-oxo-2H-chromen-4-yl)methyl)isoindoline-1,3-dione

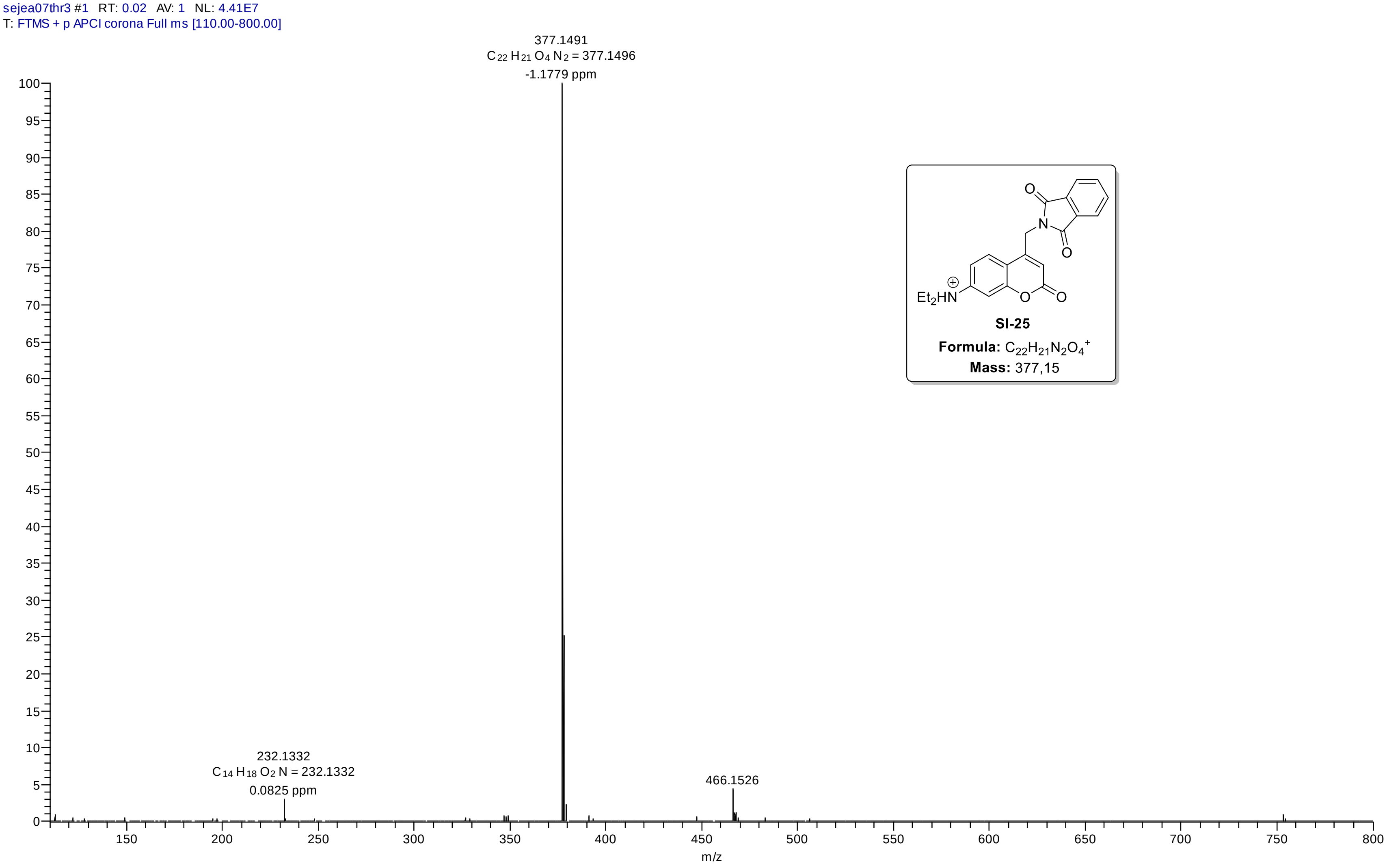

HRMS (ESI) Analysis of compound SI-26: 4-(aminomethyl)-7-(diethylamino)-2H-chromen-2-one (Amino-DEACM)

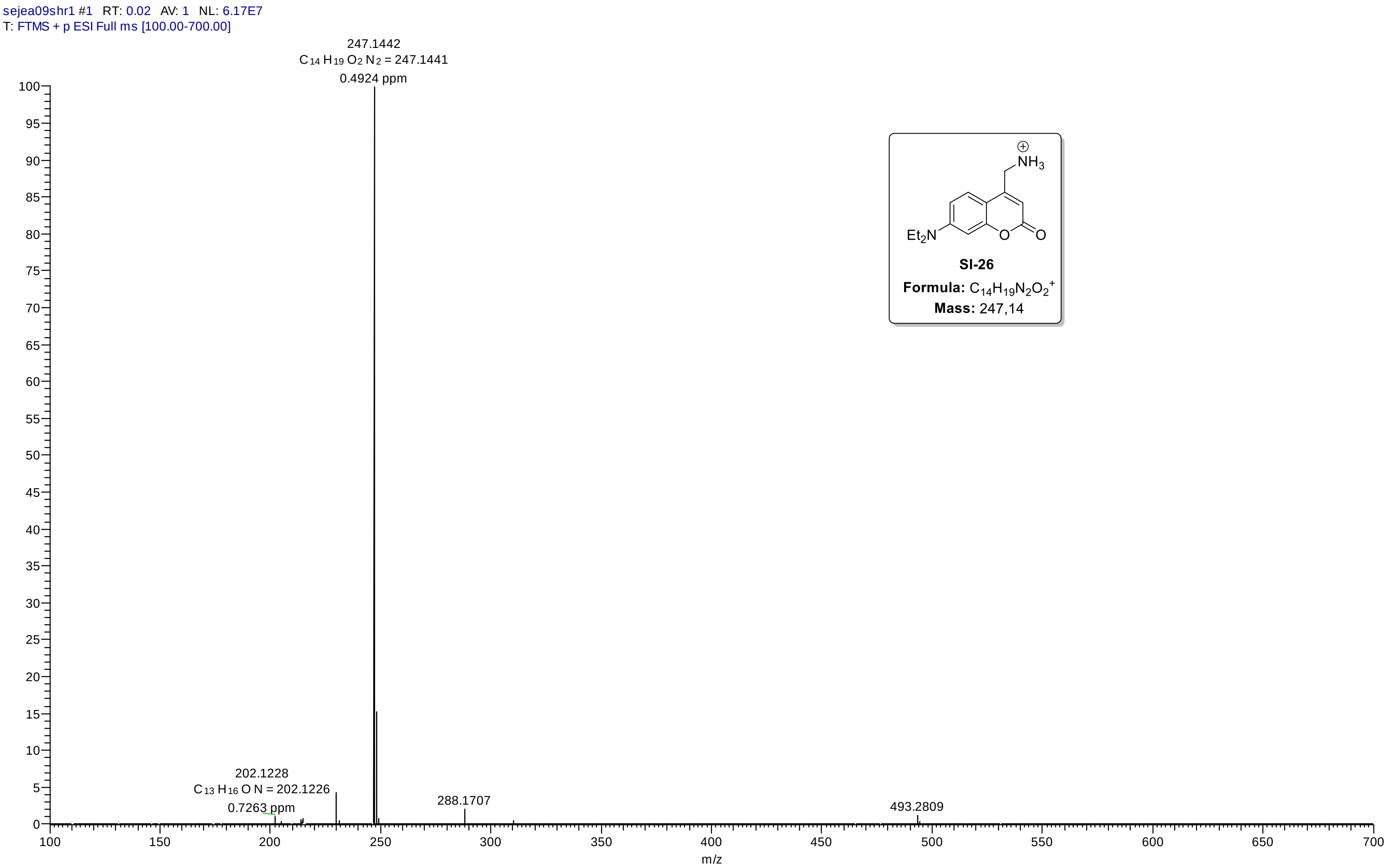

SI-26

Formula: $C_{14}H_{19}N_2O_2^+$

Mass: 247,14

14. Structure Tables (X-ray data)

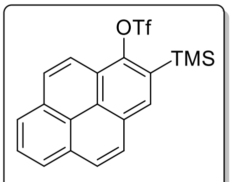

SI-19

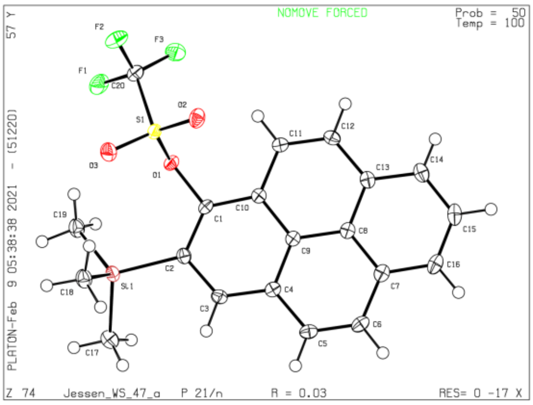

Crystals were obtained from a solvent mixture of dichloromethane and chloroform in which the compound was dissolved at 40 °C. The solution was first cooled down to room temperature, and slow evaoration of the solvent caused the formation of crystals. The data for Jessen_WS_47_a were collected from a shock-cooled single crystal at 100(2) K on a Bruker SMART APEX2 QUAZAR three-circle diffractometer with a microfocus sealed X-ray tube using mirror optics as monochromator and a Bruker APEXII detector. The diffractometer was equipped with an Oxford Cryostream 800 low temperature device and used MoK_α radiation (λ = 0.71073 Å). All data were integrated with SAINT and a multi-scan absorption correction using SADABS was applied.[1,2] The structure were solved by direct methods using SHELXT and refined by full-matrix least-squares methods against F^2 by SHELXL-2018/3.[3,4] All non-hydrogen atoms were refined with anisotropic displacement parameters. The hydrogen atoms were refined isotropically on calculated positions using a riding model with their U_{iso} values constrained to 1.5 times the U_{eq} of their pivot atoms for terminal sp^3 carbon atoms and 1.2 times for all other carbon atoms. Crystallographic data for the structures reported in this paper have been deposited with the Cambridge Crystallographic Data Centre.[5] CCDC 2062089 contain the supplementary crystallographic data for this paper. These data can be obtained free of charge from The Cambridge Crystallographic Data Centre via www.ccdc.cam.ac.uk/structures. This report and the CIF file were generated using FinalCif.[6]

Table 1. Crystal data and structure refinement for Jessen_WS_47_a

CCDC number	2062089
Empirical formula	$C_{20}H_{17}F_3O_3SSi$
Formula weight	422.48
Temperature [K]	100(2)
Crystal system	monoclinic
Space group (number)	$P2_1/n$ (14)
a [Å]	8.9023(7)
b [Å]	16.4839(12)
c [Å]	13.5475(10)
α [°]	90
β [°]	107.9570(10)
γ [°]	90
Volume [Å^3]	1891.2(2)
Z	4
ρ_{calc} [gcm^{-3}]	1.484
μ [mm^{-1}]	0.282
F(000)	872
Crystal size [mm^3]	0.170×0.150×0.100
Crystal colour	colourless
Crystal shape	block
Radiation	MoK_α (λ=0.71073 Å)
2Θ range [°]	4.01 to 55.11 (0.77 Å)
Index ranges	$-11 \leq h \leq 11$<br>$-21 \leq k \leq 21$<br>$-17 \leq l \leq 17$
Reflections collected	39111
Independent reflections	4368<br>R_{int} = 0.0298<br>R_{sigma} = 0.0160
Completeness to Θ = 25.242°	99.9 %
Data / Restraints / Parameters	4368/0/256
Goodness-of-fit on F^2	1.062
Final *R* indexes [$I \geq 2\sigma(I)$]	R_1 = 0.0290<br>wR_2 = 0.0780
Final *R* indexes [all data]	R_1 = 0.0341<br>wR_2 = 0.0814
Largest peak/hole [eÅ^{-3}]	0.47/-0.37

Table 2. Atomic coordinates and U_{eq} [Å^2] for Jessen_WS_47_a

Atom	*x*	*y*	*z*	U_{eq}
S1	0.30474(4)	0.35973(2)	0.81748(2)	0.01550(9)
Si1	0.29667(4)	0.17974(2)	0.61769(3)	0.01555(9)
F1	0.06123(11)	0.30635(6)	0.86700(7)	0.0295(2)
F2	0.22234(11)	0.38224(6)	0.98151(7)	0.0311(2)
F3	0.06171(11)	0.43591(6)	0.84399(7)	0.0285(2)
O1	0.18751(11)	0.34506(6)	0.70625(7)	0.01582(19)
O2	0.38573(12)	0.43487(6)	0.82526(8)	0.0215(2)
O3	0.38844(12)	0.28781(6)	0.85899(8)	0.0230(2)
C1	0.24254(15)	0.35737(8)	0.61689(10)	0.0144(3)
C2	0.29429(15)	0.28991(8)	0.57493(10)	0.0152(3)
C3	0.34438(15)	0.30589(8)	0.48816(10)	0.0163(3)
H3	0.382620	0.261986	0.457278	0.020
C4	0.34077(15)	0.38317(8)	0.44499(10)	0.0150(3)
C5	0.39467(15)	0.39853(9)	0.35686(10)	0.0178(3)
H5	0.437703	0.355154	0.328064	0.021
C6	0.38552(15)	0.47321(9)	0.31410(11)	0.0188(3)
H6	0.422691	0.481374	0.256218	0.023
C7	0.32021(15)	0.54065(8)	0.35500(10)	0.0169(3)
C8	0.26746(14)	0.52759(8)	0.44262(10)	0.0147(3)
C9	0.27939(14)	0.44887(8)	0.48850(10)	0.0136(2)
C10	0.22802(14)	0.43633(8)	0.57686(10)	0.0140(2)
C11	0.16150(15)	0.50339(8)	0.61727(10)	0.0161(3)
H11	0.125846	0.495479	0.675832	0.019
C12	0.14924(15)	0.57750(8)	0.57274(10)	0.0168(3)
H12	0.104224	0.620686	0.600560	0.020
C13	0.20222(15)	0.59273(8)	0.48479(10)	0.0161(3)
C14	0.18988(16)	0.66940(8)	0.43789(11)	0.0198(3)
H14	0.145873	0.713327	0.465080	0.024
C15	0.24139(17)	0.68170(9)	0.35211(11)	0.0222(3)
H15	0.231951	0.733909	0.321101	0.027
C16	0.30659(16)	0.61836(9)	0.31123(11)	0.0207(3)
H16	0.342294	0.627859	0.253003	0.025
C17	0.26384(18)	0.11607(9)	0.49861(12)	0.0239(3)
H17A	0.171902	0.136479	0.443748	0.036
H17B	0.245014	0.059708	0.514593	0.036
H17C	0.357394	0.118652	0.475107	0.036
C18	0.49555(16)	0.15408(9)	0.70861(11)	0.0207(3)
H18A	0.574641	0.158348	0.672075	0.031
H18B	0.494352	0.098537	0.734161	0.031
H18C	0.522058	0.191870	0.767300	0.031
C19	0.13349(16)	0.15804(9)	0.67359(12)	0.0213(3)
H19A	0.033441	0.177887	0.626151	0.032
H19B	0.155093	0.185437	0.740723	0.032
H19C	0.126339	0.099400	0.683205	0.032
C20	0.15111(17)	0.37193(9)	0.88066(11)	0.0209(3)

U_{eq} is defined as 1/3 of the trace of the orthogonalized U_{ij} tensor.

Table 3. Bond lengths and angles for Jessen_WS_47_a

Atom–Atom	Length [Å]
S1–O2	1.4206(10)
S1–O3	1.4208(10)
S1–O1	1.5648(10)

S1–C20	1.8345(15)
Si1–C18	1.8671(14)
Si1–C19	1.8682(14)
Si1–C17	1.8705(15)
Si1–C2	1.9041(14)
F1–C20	1.3235(17)
F2–C20	1.3279(17)
F3–C20	1.3224(17)
O1–C1	1.4534(15)
C1–C2	1.3905(18)
C1–C10	1.4005(18)
C2–C3	1.4048(18)
C3–C4	1.3980(18)
C3–H3	0.9500
C4–C9	1.4204(18)
C4–C5	1.4398(18)
C5–C6	1.352(2)
C5–H5	0.9500
C6–C7	1.4421(19)
C6–H6	0.9500
C7–C16	1.4010(19)
C7–C8	1.4210(18)
C8–C13	1.4218(18)
C8–C9	1.4285(18)
C9–C10	1.4220(18)
C10–C11	1.4395(18)
C11–C12	1.3520(19)
C11–H11	0.9500
C12–C13	1.4324(19)
C12–H12	0.9500
C13–C14	1.4035(19)
C14–C15	1.389(2)
C14–H14	0.9500
C15–C16	1.390(2)
C15–H15	0.9500
C16–H16	0.9500
C17–H17A	0.9800
C17–H17B	0.9800
C17–H17C	0.9800
C18–H18A	0.9800
C18–H18B	0.9800
C18–H18C	0.9800
C19–H19A	0.9800
C19–H19B	0.9800
C19–H19C	0.9800

**Atom–Atom–Atom**	**Angle [°]**
O2–S1–O3	120.39(6)
O2–S1–O1	112.26(6)
O3–S1–O1	111.39(6)
O2–S1–C20	107.71(6)
O3–S1–C20	106.39(6)
O1–S1–C20	95.42(6)
C18–Si1–C19	112.76(7)
C18–Si1–C17	107.82(7)
C19–Si1–C17	107.93(7)
C18–Si1–C2	109.64(6)
C19–Si1–C2	111.64(6)
C17–Si1–C2	106.78(6)
C1–O1–S1	118.98(8)
C2–C1–C10	125.99(12)
C2–C1–O1	117.90(11)
C10–C1–O1	115.96(11)
C1–C2–C3	115.01(12)
C1–C2–Si1	127.76(10)
C3–C2–Si1	117.18(10)
C4–C3–C2	123.28(12)
C4–C3–H3	118.4
C2–C3–H3	118.4
C3–C4–C9	118.90(12)
C3–C4–C5	122.46(12)
C9–C4–C5	118.63(12)
C6–C5–C4	121.60(13)
C6–C5–H5	119.2
C4–C5–H5	119.2
C5–C6–C7	121.01(12)
C5–C6–H6	119.5
C7–C6–H6	119.5
C16–C7–C8	118.97(13)
C16–C7–C6	122.32(13)
C8–C7–C6	118.71(12)
C7–C8–C13	120.15(12)
C7–C8–C9	120.12(12)
C13–C8–C9	119.73(12)
C4–C9–C10	120.21(12)
C4–C9–C8	119.90(12)
C10–C9–C8	119.89(12)
C1–C10–C9	116.48(11)
C1–C10–C11	124.47(12)
C9–C10–C11	119.03(12)
C12–C11–C10	120.69(12)
C12–C11–H11	119.7
C10–C11–H11	119.7
C11–C12–C13	121.84(12)
C11–C12–H12	119.1
C13–C12–H12	119.1
C14–C13–C8	118.87(13)
C14–C13–C12	122.31(13)
C8–C13–C12	118.81(12)
C15–C14–C13	120.67(13)
C15–C14–H14	119.7
C13–C14–H14	119.7
C14–C15–C16	120.64(13)
C14–C15–H15	119.7
C16–C15–H15	119.7
C15–C16–C7	120.69(13)
C15–C16–H16	119.7
C7–C16–H16	119.7

Si1–C17–H17A	109.5	Si1–C19–H19A	109.5
Si1–C17–H17B	109.5	Si1–C19–H19B	109.5
H17A–C17–H17B	109.5	H19A–C19–H19B	109.5
Si1–C17–H17C	109.5	Si1–C19–H19C	109.5
H17A–C17–H17C	109.5	H19A–C19–H19C	109.5
H17B–C17–H17C	109.5	H19B–C19–H19C	109.5
Si1–C18–H18A	109.5	F3–C20–F1	109.33(12)
Si1–C18–H18B	109.5	F3–C20–F2	109.00(12)
H18A–C18–H18B	109.5	F1–C20–F2	108.96(11)
Si1–C18–H18C	109.5	F3–C20–S1	111.16(9)
H18A–C18–H18C	109.5	F1–C20–S1	110.53(10)
H18B–C18–H18C	109.5	F2–C20–S1	107.81(10)

Table 4. Torsion angles for Jessen_WS_47_a

**Atom–Atom–Atom–Atom**	**Torsion Angle [°]**		
O2–S1–O1–C1	48.43(11)	C2–C1–C10–C9	−3.7(2)
O3–S1–O1–C1	−89.94(10)	O1–C1–C10–C9	−179.13(10)
C20–S1–O1–C1	160.08(10)	C2–C1–C10–C11	174.50(12)
S1–O1–C1–C2	96.23(12)	O1–C1–C10–C11	−0.95(18)
S1–O1–C1–C10	−87.93(12)	C4–C9–C10–C1	0.32(18)
C10–C1–C2–C3	4.0(2)	C8–C9–C10–C1	179.57(11)
O1–C1–C2–C3	179.40(11)	C4–C9–C10–C11	−177.97(11)
C10–C1–C2–Si1	−173.22(10)	C8–C9–C10–C11	1.28(18)
O1–C1–C2–Si1	2.16(18)	C1–C10–C11–C12	−178.78(12)
C1–C2–C3–C4	−1.08(19)	C9–C10–C11–C12	−0.64(19)
Si1–C2–C3–C4	176.47(10)	C10–C11–C12–C13	−0.4(2)
C2–C3–C4–C9	−1.9(2)	C7–C8–C13–C14	0.53(19)
C2–C3–C4–C5	179.13(12)	C9–C8–C13–C14	−179.30(12)
C3–C4–C5–C6	177.83(13)	C7–C8–C13–C12	179.62(12)
C9–C4–C5–C6	−1.16(19)	C9–C8–C13–C12	−0.21(18)
C4–C5–C6–C7	−0.4(2)	C11–C12–C13–C14	179.92(13)
C5–C6–C7–C16	−178.84(13)	C11–C12–C13–C8	0.9(2)
C5–C6–C7–C8	1.0(2)	C8–C13–C14–C15	−0.4(2)
C16–C7–C8–C13	−0.08(19)	C12–C13–C14–C15	−179.46(13)
C6–C7–C8–C13	−179.90(12)	C13–C14–C15–C16	−0.2(2)
C16–C7–C8–C9	179.75(12)	C14–C15–C16–C7	0.7(2)
C6–C7–C8–C9	−0.07(18)	C8–C7–C16–C15	−0.5(2)
C3–C4–C9–C10	2.25(19)	C6–C7–C16–C15	179.29(13)
C5–C4–C9–C10	−178.72(11)	O2–S1–C20–F3	52.21(12)
C3–C4–C9–C8	−176.99(12)	O3–S1–C20–F3	−177.44(10)
C5–C4–C9–C8	2.03(18)	O1–S1–C20–F3	−63.24(11)
C7–C8–C9–C4	−1.44(18)	O2–S1–C20–F1	173.81(10)
C13–C8–C9–C4	178.39(11)	O3–S1–C20–F1	−55.84(11)
C7–C8–C9–C10	179.32(12)	O1–S1–C20–F1	58.36(11)
C13–C8–C9–C10	−0.85(18)	O2–S1–C20–F2	−67.20(11)
		O3–S1–C20–F2	63.16(11)
		O1–S1–C20–F2	177.35(10)

Bibliography (x-ray part)

[1] Bruker, *SAINT*, *V8.38A*, Bruker AXS Inc., Madison, Wisconsin, USA.

[2] L. Krause, R. Herbst-Irmer, G. M. Sheldrick, D. Stalke, *J. Appl. Cryst.* **2015**, *48*, 3–10, doi:10.1107/S1600576714022985.
[3] G. M. Sheldrick, *Acta Cryst.* **2015**, *A71*, 3–8, doi:10.1107/S2053273314026370.
[4] G. M. Sheldrick, *Acta Cryst.* **2015**, *C71*, 3–8, doi:10.1107/S2053229614024218.
[5] C. R. Groom, I. J. Bruno, M. P. Lightfoot, S. C. Ward, *Acta Cryst.* **2016**, *B72*, 171–179, doi:10.1107/S2052520616003954.
[6] D. Kratzert, *FinalCif*, *V84*, https://www.xs3.uni-freiburg.de/research/finalcif.
